# Supplementary material for: Livestock Informatics Toolkit: A Case Study in Visually Characterizing Complex Behavioral Patterns across Multiple Sensor Platforms, Using Novel Unsupervised Machine Learning and Information Theoretic Approaches
Source: Sensors (Basel). 2021 Dec 21;22(1):1. doi: 10.3390/s22010001 (PMC8747447; doi:10.3390/s22010001)
Supplement: Supplementary file 1 [file sensors-22-00001-s001.zip › sensors-1463895-supplementary/OverallTB/Analysis_of_Overall_Time_Budgets.html]

Heirachical Analysis of Time Budget Data: Overall Time Budget


# Heirachical Analysis of Time Budget Data: Overall Time Budget

```
library('LIT')
library('tidyverse')
```

```
## Warning: package 'tidyverse' was built under R version 3.5.2
```

```
## ── Attaching packages ────────────────────────────────── tidyverse 1.3.0 ──
```

```
## ✓ ggplot2 3.3.2     ✓ purrr   0.3.4
## ✓ tibble  3.0.1     ✓ dplyr   1.0.2
## ✓ tidyr   1.1.0     ✓ stringr 1.4.0
## ✓ readr   1.3.1     ✓ forcats 0.5.0
```

```
## Warning: package 'stringr' was built under R version 3.5.2
```

```
## Warning: package 'forcats' was built under R version 3.5.2
```

```
## ── Conflicts ───────────────────────────────────── tidyverse_conflicts() ──
## x dplyr::filter() masks stats::filter()
## x dplyr::lag()    masks stats::lag()
```

```
library("RColorBrewer")
library('pheatmap')
```

```
## Warning: package 'pheatmap' was built under R version 3.5.2
```

```
library('lattice')
library('dendextend')
```

```
## Warning: package 'dendextend' was built under R version 3.5.2
```

```
## 
## ---------------------
## Welcome to dendextend version 1.13.4
## Type citation('dendextend') for how to cite the package.
## 
## Type browseVignettes(package = 'dendextend') for the package vignette.
## The github page is: https://github.com/talgalili/dendextend/
## 
## Suggestions and bug-reports can be submitted at: https://github.com/talgalili/dendextend/issues
## Or contact: <tal.galili@gmail.com>
## 
##  To suppress this message use:  suppressPackageStartupMessages(library(dendextend))
## ---------------------
```

```
## 
## Attaching package: 'dendextend'
```

```
## The following object is masked from 'package:stats':
## 
##     cutree
```

# Data Wrangling

## Data Wrangling - Cow Attributes

First, I need to pull together a bunch of the cow attribute fields from various data sets.

### Health Status

I’ll begin by looking at the sick lists.

```
startdate <- 17214 #17241 #min(as.numeric(names(milkquantdat_all)[-1]))
pasturedate <- 17280


sickdat <- read.csv('../Round_2/Data/sick_cows_organilac.csv', stringsAsFactors = F)
sickdat$DIGNOSIS[sickdat$DIGNOSIS == ''] <- NA
sickdat$DATE.SICK[sickdat$DATE.SICK == ''] <- NA 

sickdat$Date <- as.Date(sickdat$DATE.SICK, format = '%m/%d/%y')
sickdat$DateN <- as.numeric(sickdat$Date)  

sickdat$Sick_Pasture <- sickdat$Sick  &  sickdat$DateN > pasturedate
sickdat$Sick_Pen <- sickdat$Sick  &  sickdat$DateN < pasturedate  &  sickdat$DateN > startdate
sickdat$Sick_BurnIn <-  sickdat$Sick  &  sickdat$DateN < startdate
sickdat$Sick_Prepasture <- sickdat$Sick_Pen | sickdat$Sick_BurnIn


cowlist_healthy_all <- sickdat$ID[sickdat$Sick == 0]
length(cowlist_healthy_all)
```

```
## [1] 136
```

### Birth Date

```
birthdatedat_master <- read.csv('../Round_2/Data/Birthdates-aurora.CSV', stringsAsFactors = F)
birthdatedat_master$Date <- as.Date(birthdatedat_master$BrthDate, format = '%m/%d/%y')
names(birthdatedat_master)[1] <- 'CowID' 
birthdatedat_master$CowID <- gsub(' ', '', birthdatedat_master$CowID)

birthdatedat <- merge(data.frame(CowID = sickdat$ID), birthdatedat_master, by = 'CowID', all.x = T)

for(i in 1:nrow(birthdatedat)){
  
  if(is.na(birthdatedat$Date[i])){
    temp <- which(paste(birthdatedat$CowID[i],'*', sep='') == birthdatedat_master$CowID)
    if(length(temp) > 0){
      birthdatedat$BrthDate[i] <- birthdatedat_master$BrthDate[temp]
      birthdatedat$Date[i] <- birthdatedat_master$Date[temp]
    }
  }
  
}
 
birthdatedat$DateN <- 17241 - as.numeric(birthdatedat$Date) # age of cow on the first day the herd was fully established, which I set to be 3/16/17
```

### Calving Date

Now I want to get the date that cows entered the herd using their calving date records. I’ll also pull their experimental treatment group.

```
milk150 <- read.csv('../Round_2/Data/Milk Yeild 150 DIM.csv', stringsAsFactors = F, na.strings = '.')
experdat <- unique(milk150[,-c(2,3,4, 6, 8)])
names(experdat) <- c('CowID','Group','CalvingDate')

experdat$CalvingDateF <- as.Date(experdat$CalvingDate, format = '%m/%d/%y', tz = 'MST')
experdat$CalvingDateN <- as.numeric(experdat$CalvingDateF) - min(as.numeric(experdat$CalvingDateF), na.rm = T)
```

And now to check the enrollement rate

```
temp <- milk150[,c('ID', 'calv_date')]
temp <- unique(temp)

table(temp$calv_date)
```

```
## 
##         1/13/17 1/14/17 1/15/17 1/16/17 1/17/17 1/18/17 1/19/17 1/20/17 1/21/17 
##       1       5      13       9      11       9      15      14       8      11 
## 1/22/17 1/23/17 1/24/17 1/26/17 1/27/17 1/29/17 1/30/17 1/31/17 2/13/17  2/2/17 
##       9      21      11      21       6      11       4       8       5       5 
##  2/3/17  2/5/17  2/7/17  2/8/17 
##       2       1       1       1
```

```
cumsum(table(temp$calv_date))
```

```
##         1/13/17 1/14/17 1/15/17 1/16/17 1/17/17 1/18/17 1/19/17 1/20/17 1/21/17 
##       1       6      19      28      39      48      63      77      85      96 
## 1/22/17 1/23/17 1/24/17 1/26/17 1/27/17 1/29/17 1/30/17 1/31/17 2/13/17  2/2/17 
##     105     126     137     158     164     175     179     187     192     197 
##  2/3/17  2/5/17  2/7/17  2/8/17 
##     199     200     201     202
```

So, most of our cows are into the herd by the middle of Feb.

### Milk Yield

And now, finally, I want to look at their milk yield. This data set is challenging to work with, as it is not validated test day records but noisy parlor records. There are a lot of missing entries from failed readers, and also a lot of low-ball yields when cows knock their claws off one or more times on a given day. That will make fitting the actual curve to the data dubious at best. So I think I am just simply going to pull off the 95% quantile milking record and use that as a robust estimate of peak yield.

```
milk150dat <- data.frame(CowID = sickdat$ID, MilkYield95 = NA)

for(i in 1:nrow(milk150dat)){
  
  temp <- subset(milk150$MILKDAY, milk150$ID == milk150dat$CowID[i])
  milk150dat$MilkYield95[i] <- quantile(temp, probs = 0.95, na.rm = T)
  
}
```

## Data Wrangling - Sensor Data

The first sensor data set I want to bring in is the herd manager data set, and get the columns formatted.

```
HerdMan_master <- read.csv('../Round_2/Data/Sensor Data/HerdManager_Masterlist.csv', header = T, stringsAsFactors = F)
HerdMan_master$TimeStampRaw <- strptime(HerdMan_master$TimeStamp, format = '%Y%m%d %H:%M:%S', tz = 'GMT')
HerdMan_master$TimeStampF <- lubridate::with_tz(HerdMan_master$TimeStampRaw, 'MST')

HerdMan_master$Date <- as.Date(HerdMan_master$TimeStampF)
head(HerdMan_master)
```

Next, I want to cull down the datelist so that I only look at dates after the herd has been fully established. In my milk order analyses, I waited for the cows to not only finish entering the herd but also gave them time to establish a stable heirarchy, and so I didn’t start the data until 3/12/17 (17182+55).

Here, however, I want to recover inconsistencies in herd behavior, so I don’t need to cut off as much data on the front end. So I’m going to start looking at data a few days after the final cow enters the herd on 2/17/17 (17214). I’ll cut the data off after the herd gaiened access to pasture on April 24 (17280), at which point there were reader problems.

```
# peddat <- subset(HerdMan_master, 
#                  as.numeric(HerdMan_master$Date) >= (17182+55) &
#                    as.numeric(HerdMan_master$Date) <  17363 - 7)
peddat <- subset(HerdMan_master, 
                 as.numeric(HerdMan_master$Date) >= startdate &
                   as.numeric(HerdMan_master$Date) <  pasturedate)

#table(peddat$Id)
length(unique(peddat$Date))
```

```
## [1] 66
```

```
table(as.numeric(peddat$Date))
```

```
## 
## 17214 17215 17216 17217 17218 17219 17220 17221 17222 17223 17224 17225 17226 
##  4560  4560  4560  4560  4560  4560  4560  4560  4560  4560  4560  4559  4526 
## 17227 17228 17229 17230 17231 17232 17233 17234 17235 17236 17237 17238 17239 
##  4512  4480  4464  4464  4464  4464  4464  4464  4463  4440  4440  4440  4440 
## 17240 17241 17242 17243 17244 17245 17246 17247 17248 17249 17250 17251 17252 
##  4422  4440  4440  4440  4440  4440  4440  4425  4416  4416  4416  4416  4416 
## 17253 17254 17255 17256 17257 17258 17259 17260 17261 17262 17263 17264 17265 
##  4407  4408  4416  4416  4416  4416  4416  4416  4398  4392  4382  4368  4368 
## 17266 17267 17268 17269 17270 17271 17272 17273 17274 17275 17276 17277 17278 
##  4368  4368  4368  4368  4360  4344  4344  4346  4368  4368  4368  4368  4368 
## 17279 
##  2548
```

That leaves us with 66 days of observations. But it looks like we’ve got one day where several hundred observations are missing, which coincidentally is the day before pasture access, so we’ll drop that one to be safe as well. Leaving 65 observation days

```
peddat <- subset(peddat, as.numeric(peddat$Date) != 17279 )
length(unique(peddat$Date))
```

```
## [1] 65
```

```
table(peddat$Date)
```

```
## 
## 2017-02-17 2017-02-18 2017-02-19 2017-02-20 2017-02-21 2017-02-22 2017-02-23 
##       4560       4560       4560       4560       4560       4560       4560 
## 2017-02-24 2017-02-25 2017-02-26 2017-02-27 2017-02-28 2017-03-01 2017-03-02 
##       4560       4560       4560       4560       4559       4526       4512 
## 2017-03-03 2017-03-04 2017-03-05 2017-03-06 2017-03-07 2017-03-08 2017-03-09 
##       4480       4464       4464       4464       4464       4464       4464 
## 2017-03-10 2017-03-11 2017-03-12 2017-03-13 2017-03-14 2017-03-15 2017-03-16 
##       4463       4440       4440       4440       4440       4422       4440 
## 2017-03-17 2017-03-18 2017-03-19 2017-03-20 2017-03-21 2017-03-22 2017-03-23 
##       4440       4440       4440       4440       4440       4425       4416 
## 2017-03-24 2017-03-25 2017-03-26 2017-03-27 2017-03-28 2017-03-29 2017-03-30 
##       4416       4416       4416       4416       4407       4408       4416 
## 2017-03-31 2017-04-01 2017-04-02 2017-04-03 2017-04-04 2017-04-05 2017-04-06 
##       4416       4416       4416       4416       4416       4398       4392 
## 2017-04-07 2017-04-08 2017-04-09 2017-04-10 2017-04-11 2017-04-12 2017-04-13 
##       4382       4368       4368       4368       4368       4368       4368 
## 2017-04-14 2017-04-15 2017-04-16 2017-04-17 2017-04-18 2017-04-19 2017-04-20 
##       4360       4344       4344       4346       4368       4368       4368 
## 2017-04-21 2017-04-22 
##       4368       4368
```

That means all my cows should have 1560 hours of observations. It looks like I have only a handful of cows with any missing values. As this may mean that these cows left the experimental pen outside the range of the router for some period of time, perhaps for health treatments, I’m just going to go ahead and drop them.

```
#table(peddat$Id)
table(peddat$Id)[table(peddat$Id)<max(table(peddat$Id))]
```

```
## 
##   594  1393  1839 10500 13344 13907 13920 19484 21906 31889 63106 
##   344   527  1542  1543   302   287   801  1134  1482  1190   344
```

```
#peddat <- subset(peddat, !(peddat$Id %in% c(1839, 10500, 13920, 19484, 21906, 31889)))
peddat <- subset(peddat, !(peddat$Id %in% c(594, 1393, 1839, 10500, 13344, 13907, 13920, 19484, 21906, 31889, 63106 , 97539, 13960))) # last two I'm pulling bc I'm missing all their auxdata

length(unique(peddat$Id))
```

```
## [1] 177
```

That leaves me 179 focal cows out of the original 202.

Now I need to format the hour field, and correct for the time zone

```
#peddat$adjtemperdat_night$TimeStampC <- <- as.character(peddat$TimeStampF)
peddat$TimeDiff <- peddat$TimeStampF -  peddat$TimeStampF[1]

peddat$TimeStampC <- as.character(peddat$TimeStampF)
peddat$Hour  <- as.numeric(substring(peddat$TimeStampC, 12,13))

# peddat$HourCorrected <- NA
# peddat$HourCorrected[peddat$Hour == 0] <- 19
# peddat$HourCorrected[peddat$Hour == 1] <- 20
# peddat$HourCorrected[peddat$Hour == 2] <- 21
# peddat$HourCorrected[peddat$Hour == 3] <- 22
# peddat$HourCorrected[peddat$Hour == 4] <- 23
# peddat$HourCorrected[peddat$Hour == 5] <- 0
# peddat$HourCorrected[peddat$Hour == 6] <- 1
# peddat$HourCorrected[peddat$Hour == 7] <- 2
# peddat$HourCorrected[peddat$Hour == 8] <- 3
# peddat$HourCorrected[peddat$Hour == 9] <- 4
# peddat$HourCorrected[peddat$Hour == 10] <- 5
# peddat$HourCorrected[peddat$Hour == 11] <- 6
# peddat$HourCorrected[peddat$Hour == 12] <- 7
# peddat$HourCorrected[peddat$Hour == 13] <- 8
# peddat$HourCorrected[peddat$Hour == 14] <- 9
# peddat$HourCorrected[peddat$Hour == 15] <- 10
# peddat$HourCorrected[peddat$Hour == 16] <- 11
# peddat$HourCorrected[peddat$Hour == 17] <- 12
# peddat$HourCorrected[peddat$Hour == 18] <- 13
# peddat$HourCorrected[peddat$Hour == 19] <- 14
# peddat$HourCorrected[peddat$Hour == 20] <- 15
# peddat$HourCorrected[peddat$Hour == 21] <- 16
# peddat$HourCorrected[peddat$Hour == 22] <- 17
# peddat$HourCorrected[peddat$Hour == 23] <- 18
# peddat$Hourc <- as.factor(peddat$HourCorrected)

peddat$DateN <- as.numeric(peddat$Date)
peddat$DayOnTrial <- peddat$DateN - 17182 # official start date 1/16/17
peddat$Datec <- as.factor(peddat$DayOnTrial)
```

Ok, now I want to isolate the cows that I know had no recorded health events from the larger herd

```
# healthy cow data

#peddat_sickhealthy <- peddat
peddat_healthy <- subset(peddat, peddat$Id %in% cowlist_healthy_all)
cowlist_peddat_healthy <- unique(peddat_healthy$Id)
length(cowlist_peddat_healthy)
```

```
## [1] 124
```

```
# all cow data

cowlist_peddat <- unique(peddat$Id)
length(cowlist_peddat)
```

```
## [1] 177
```

Next, in order to caluculate a distance matrix, I need to align this data by the time stamps assigned to each observation across cows. In order to encourage the merges to run faster, I’m going to create an interger column that represent the difference in seconds from the earliest time stamp in this dataset (2-17-2017 00:00:00)

```
uniquedatelist <- unique(peddat[,c(which(names(peddat)=='TimeStampF'),
                                   which(names(peddat)=='TimeDiff'),
                                   which(names(peddat)=='Date'),
                                   which(names(peddat)=='Hour')
                                   )])


# initialize data frames

nonactivedat <- uniquedatelist
nonactivedat <- cbind(nonactivedat, matrix(NA, ncol = length(cowlist_peddat), nrow = length(unique(peddat$TimeDiff))))
names(nonactivedat)[-c(1,2,3,4)] <- cowlist_peddat

activedat <- uniquedatelist
activedat <- cbind(activedat, matrix(NA, ncol = length(cowlist_peddat), nrow = length(unique(peddat$TimeDiff))))
names(activedat)[-c(1,2,3,4)] <- cowlist_peddat

hiactivedat <- uniquedatelist
hiactivedat <- cbind(hiactivedat, matrix(NA, ncol = length(cowlist_peddat), nrow = length(unique(peddat$TimeDiff))))
names(hiactivedat)[-c(1,2,3,4)] <- cowlist_peddat

totactivedat <- uniquedatelist
totactivedat <- cbind(totactivedat, matrix(NA, ncol = length(cowlist_peddat), nrow = length(unique(peddat$TimeDiff))))
names(totactivedat)[-c(1,2,3,4)] <- cowlist_peddat

rumdat <- uniquedatelist
rumdat <- cbind(rumdat, matrix(NA, ncol = length(cowlist_peddat), nrow = length(unique(peddat$TimeDiff))))
names(rumdat)[-c(1,2,3,4)] <- cowlist_peddat

eatdat <- uniquedatelist
eatdat <- cbind(eatdat, matrix(NA, ncol = length(cowlist_peddat), nrow = length(unique(peddat$TimeDiff))))
names(eatdat)[-c(1,2,3,4)] <- cowlist_peddat

temperdat <- uniquedatelist
temperdat <- cbind(temperdat, matrix(NA, ncol = length(cowlist_peddat), nrow = length(unique(peddat$TimeDiff))))
names(temperdat)[-c(1,2,3,4)] <- cowlist_peddat

# fill in data to structured time dataset


for(i in 1:length(cowlist_peddat)){
  
  # join cow's data with structured timestamps data
  
  cowdat <- subset(peddat, peddat$Id == cowlist_peddat[i])
  tempdat <- merge(cowdat, nonactivedat[,c(1,2)], by = 'TimeDiff', all.y = T)
  
  # add cow data to its respective rows in each response set
  
  nonactivedat[ , which(names(nonactivedat)==cowlist_peddat[i])] <- tempdat$NotActive
  activedat[ , which(names(activedat)==cowlist_peddat[i])] <- tempdat$Active
  hiactivedat[ , which(names(hiactivedat)==cowlist_peddat[i])] <- tempdat$HighActive
  rumdat[ , which(names(rumdat)==cowlist_peddat[i])] <- tempdat$Ruminating
  eatdat[ , which(names(eatdat)==cowlist_peddat[i])] <- tempdat$Eating
  temperdat[ , which(names(temperdat)==cowlist_peddat[i])] <- tempdat$Temperature
  totactivedat[ , which(names(totactivedat)==cowlist_peddat[i])]<- tempdat$TotalActivity
  
}
```

## Data Wrangling - Calculating Time Budgets

### Calculate Overall Time Budget

Alright, first thing to do is sum up the times logged for each cow in each of the behavioral axes. Here I’ll look at all the data points sampled over the entire pen and pasture study periods.

```
tbdat_cow_all <- data.frame(CowID = names(activedat[,-c(1,2,3,4)]))
tbdat_cow_all$active <- apply(activedat[,-c(1,2,3,4)], 2, function(x) sum(x, na.rm = T) )
tbdat_cow_all$highactive <- apply(hiactivedat[,-c(1,2,3,4)], 2, function(x) sum(x, na.rm = T) )
tbdat_cow_all$nonactive <- apply(nonactivedat[,-c(1,2,3,4)], 2, function(x) sum(x, na.rm = T) )
tbdat_cow_all$eat <- apply(eatdat[,-c(1,2,3,4)], 2, function(x) sum(x, na.rm = T) )
tbdat_cow_all$rumination <- apply(rumdat[,-c(1,2,3,4)], 2, function(x) sum(x, na.rm = T) )

Nobs_cow <- apply(tbdat_cow_all[,-1], 1, sum)

qplot(Nobs_cow, main = 'Distribution of Observation Counts Per Cow', xlab = 'Total Observed Minutes', col=I("black"), fill=I("blue"), alpha=I(.5)) + geom_vline(xintercept  = 1560*60)
```

```
## `stat_bin()` using `bins = 30`. Pick better value with `binwidth`.
```

This variation in total minutes is well within the bounds of being explained by the rounding error, which causes some hours to log only 59 or 61 minutes insstead of 60

Alright, and now I need to scale these sums to account for missing data points. I’ll do this by simply scaling the data to proportion of total minutes logged.

```
tbdat_cow_all$totmin <- apply(tbdat_cow_all[,-1], 1, sum)
rownames(tbdat_cow_all) <- tbdat_cow_all$CowID

tbdat_cow_all$pact <- tbdat_cow_all$active / tbdat_cow_all$totmin
tbdat_cow_all$phiact <- tbdat_cow_all$highactive / tbdat_cow_all$totmin
tbdat_cow_all$pnoact <- tbdat_cow_all$nonactive / tbdat_cow_all$totmin
tbdat_cow_all$prum <- tbdat_cow_all$rumination / tbdat_cow_all$totmin
tbdat_cow_all$peat <- tbdat_cow_all$eat / tbdat_cow_all$totmin

# make overall TB count matrix
Co <- tbdat_cow_all[ , c('active', 'highactive', 'nonactive',
                         'rumination', 'eat')]
rownames(Co) <- tbdat_cow_all$CowID
colnames(Co) <- c('Active', 'HighlyActive', 'Nonactive', 'Rumination', 'Eating')

# make overall TB proportion matrix
Po <- tbdat_cow_all[ , c('pact','phiact','pnoact','prum','peat')]
rownames(Po) <- tbdat_cow_all$CowID
colnames(Po) <- c('Active', 'HighlyActive', 'Nonactive', 'Rumination', 'Eating')
```

### Calculate Daily Time Budgets

I’ve calculated the time budget for my cows while housed in their experimental pen over 65 days, but I’ll also want to look at how the daily time budget evolves over time.

```
datelist <- unique(as.numeric(peddat$Date))
tbdat_day <- array(0, dim = c(length(cowlist_peddat), 
                              length(datelist),
                              5),
                   dimnames = list(cowlist_peddat, 
                                   unique(peddat$DayOnTrial),
                                   names(Po)))

for(day in 1:length(datelist)){
  
  temp <- subset(activedat, as.numeric(activedat$Date) == datelist[day])
  tbdat_day[ , day, 'Active'] <- apply(temp[-c(1,2,3,4)], 2, sum)
  
  temp <- subset(hiactivedat, as.numeric(hiactivedat$Date) == datelist[day])
  tbdat_day[ , day, 'HighlyActive'] <- apply(temp[-c(1,2,3,4)], 2, sum)
  
  temp <- subset(nonactivedat, as.numeric(nonactivedat$Date) == datelist[day])
  tbdat_day[ , day, 'Nonactive'] <- apply(temp[-c(1,2,3,4)], 2, sum)
  
  temp <- subset(rumdat, as.numeric(rumdat$Date) == datelist[day])
  tbdat_day[ , day, 'Rumination'] <- apply(temp[-c(1,2,3,4)], 2, sum)
  
  temp <- subset(eatdat, as.numeric(eatdat$Date) == datelist[day])
  tbdat_day[ , day, 'Eating'] <- apply(temp[-c(1,2,3,4)], 2, sum)
  
}

Po_day <- tbdat_day
daymargin <- apply(tbdat_day, c(1,2), sum)
Po_day[,,'Active'] <- tbdat_day[,,'Active']/daymargin 
Po_day[,,'HighlyActive'] <- tbdat_day[,,'HighlyActive']/daymargin 
Po_day[,,'Nonactive'] <- tbdat_day[,,'Nonactive']/daymargin 
Po_day[,,'Rumination'] <- tbdat_day[,,'Rumination']/daymargin 
Po_day[,,'Eating'] <- tbdat_day[,,'Eating']/daymargin 

#apply(Po_day, c(1,2), sum)
```

## Data Wrangling - Auxiliary Data for Heatmap Margins

### Compiling Longitudinal Variability in Daily Time budgets

```
auxmat_tbvar <- as.data.frame(matrix(NA, 
                                     nrow = length(cowlist_peddat),
                                     ncol = 5))
names(auxmat_tbvar) <- paste('Var', names(Po), sep = '_')
rownames(auxmat_tbvar) <- cowlist_peddat

auxmat_tbvar$Var_Active <- apply(Po_day[, , 'Active'], 1,
                                 function(x) log(var(x)))
auxmat_tbvar$Var_HighlyActive <- apply(Po_day[, , 'HighlyActive'], 1, function(x) log(var(x)))
auxmat_tbvar$Var_Nonactive <- apply(Po_day[, , 'Nonactive'], 1, function(x) log(var(x)))
auxmat_tbvar$Var_Rumination <- apply(Po_day[, , 'Rumination'], 1, function(x) log(var(x)))
auxmat_tbvar$Var_Eating <- apply(Po_day[, , 'Eating'], 1, function(x) log(var(x)))

auxcol_var <- list(
  Var_Active = brewer.pal(auxmat_tbvar$Var_Active, "Purples"),
  Var_HighlyActive = brewer.pal(auxmat_tbvar$Var_HighlyActive, "Purples"),
  Var_Nonactive = brewer.pal(auxmat_tbvar$Var_Nonactive, "Purples"),
  Var_Rumination = brewer.pal(auxmat_tbvar$Var_Rumination, "Purples"),
  Var_Eating = brewer.pal(auxmat_tbvar$Var_Eating, "Purples")
               )
```

```
## Warning in if (n < 3) {: the condition has length > 1 and only the first element
## will be used
```

```
## Warning in brewer.pal(auxmat_tbvar$Var_Active, "Purples"): minimal value for n is 3, returning requested palette with 3 different levels
```

```
## Warning in if (n < 3) {: the condition has length > 1 and only the first element
## will be used
```

```
## Warning in brewer.pal(auxmat_tbvar$Var_HighlyActive, "Purples"): minimal value for n is 3, returning requested palette with 3 different levels
```

```
## Warning in if (n < 3) {: the condition has length > 1 and only the first element
## will be used
```

```
## Warning in brewer.pal(auxmat_tbvar$Var_Nonactive, "Purples"): minimal value for n is 3, returning requested palette with 3 different levels
```

```
## Warning in if (n < 3) {: the condition has length > 1 and only the first element
## will be used
```

```
## Warning in brewer.pal(auxmat_tbvar$Var_Rumination, "Purples"): minimal value for n is 3, returning requested palette with 3 different levels
```

```
## Warning in if (n < 3) {: the condition has length > 1 and only the first element
## will be used
```

```
## Warning in brewer.pal(auxmat_tbvar$Var_Eating, "Purples"): minimal value for n is 3, returning requested palette with 3 different levels
```

```
auxmat_tbiqr <- as.data.frame(matrix(NA, 
                                     nrow = length(cowlist_peddat),
                                     ncol = 5))
names(auxmat_tbiqr) <- paste('IQR', names(Po), sep = '_')
rownames(auxmat_tbiqr) <- cowlist_peddat

auxmat_tbiqr$IQR_Active <- apply(Po_day[, , 'Active'], 1, var)
auxmat_tbiqr$IQR_HighlyActive <- apply(Po_day[, , 'HighlyActive'], 1, var)
auxmat_tbiqr$IQR_Nonactive <- apply(Po_day[, , 'Nonactive'], 1, var)
auxmat_tbiqr$IQR_Rumination <- apply(Po_day[, , 'Rumination'], 1, var)
auxmat_tbiqr$IQR_Eating <- apply(Po_day[, , 'Eating'], 1, var)

auxcol_var <- list(
  IQR_Active = brewer.pal(auxmat_tbiqr$IQR_Active, "Purples"),
  IQR_HighlyActive = brewer.pal(auxmat_tbiqr$IQR_HighlyActive, "Purples"),
  IQR_Nonactive = brewer.pal(auxmat_tbiqr$IQR_Nonactive, "Purples"),
  IQR_Rumination = brewer.pal(auxmat_tbiqr$IQR_Rumination, "Purples"),
  IQR_Eating = brewer.pal(auxmat_tbiqr$IQR_Eating, "Purples")
)
```

```
## Warning in if (n < 3) {: the condition has length > 1 and only the first element
## will be used
```

```
## Warning in brewer.pal(auxmat_tbiqr$IQR_Active, "Purples"): minimal value for n is 3, returning requested palette with 3 different levels
```

```
## Warning in if (n < 3) {: the condition has length > 1 and only the first element
## will be used
```

```
## Warning in brewer.pal(auxmat_tbiqr$IQR_HighlyActive, "Purples"): minimal value for n is 3, returning requested palette with 3 different levels
```

```
## Warning in if (n < 3) {: the condition has length > 1 and only the first element
## will be used
```

```
## Warning in brewer.pal(auxmat_tbiqr$IQR_Nonactive, "Purples"): minimal value for n is 3, returning requested palette with 3 different levels
```

```
## Warning in if (n < 3) {: the condition has length > 1 and only the first element
## will be used
```

```
## Warning in brewer.pal(auxmat_tbiqr$IQR_Rumination, "Purples"): minimal value for n is 3, returning requested palette with 3 different levels
```

```
## Warning in if (n < 3) {: the condition has length > 1 and only the first element
## will be used
```

```
## Warning in brewer.pal(auxmat_tbiqr$IQR_Eating, "Purples"): minimal value for n is 3, returning requested palette with 3 different levels
```

### Compiling Cow Attributes

```
cowattribdat <- birthdatedat[ ,c('CowID', 'BrthDate', 'DateN')]
names(cowattribdat)[3] <- 'AgeDaysOld'

cowattribdat <- merge(cowattribdat, milk150dat, by = 'CowID', all.x = T)

temp <- experdat[ ,c('CowID', 'CalvingDate', 'CalvingDateN')]
cowattribdat <- merge(cowattribdat, temp, by = 'CowID', all.x = T)

temp <- sickdat[ , c('ID' ,'group', "Sick", "Sick_BurnIn", "Sick_Pen", "Sick_Pasture", "DIGNOSIS", "Sick_Prepasture")]
names(temp)[1] <- 'CowID' 
cowattribdat <- merge(cowattribdat, temp, by = 'CowID', all.x = T)
cowattribdat$Sick <- ifelse(cowattribdat$Sick == 1, TRUE, FALSE)
cowattribdat$Sick_Prepasture <- ifelse(cowattribdat$Sick_Prepasture, 'Sick', 'Healthy')
```

Now I need to discretized these values for my heatmap attribute visualizations

```
DMdat <- data.frame(CowID = cowattribdat$CowID)
rownames(DMdat) <- DMdat$CowID

# Calving Date

tempvec <- cowattribdat$CalvingDateN
names(tempvec) <- cowattribdat$CowID
tempvec <- tempvec[!is.na(tempvec)]
d <- dist(tempvec)

h.cdate <- hclust(d, method = 'ward.D2') 
plot(h.cdate, xlab = 'Cow ID', labels = tempvec, main = 'Dendrogram of Calving Date')
```

```
temp <- cowattribdat[ , c('CowID','CalvingDate','CalvingDateN')]
rownames(temp) <- temp$CowID
temp <- temp[!is.na(temp$CalvingDateN),]

nclust = 4
temp$CalvingDateC <- cutree(h.cdate, nclust)
temp$CalvingDateT <- temp$CalvingDateC 

for(i in 1:nclust){
  
  temp2 <- subset(temp, temp$CalvingDateC == i)
  stringtemp <- paste(temp2$CalvingDate[which.min(temp2$CalvingDateN)], '-', 
                      temp2$CalvingDate[which.max(temp2$CalvingDateN)], sep = '')
  
  temp$CalvingDateC[temp$CalvingDateC == i] <- stringtemp
  
}

DMdat <- merge(DMdat, temp[,c('CowID','CalvingDateC')], by = 'CowID', all.x = T)

temp2 <- temp[order(temp$CalvingDateN),]
DMdat$CalvingDateC <- factor(DMdat$CalvingDateC, levels = unique(temp2$CalvingDateC), ordered = T)


# Cow Age

tempvec <- cowattribdat$AgeDaysOld
names(tempvec) <- cowattribdat$CowID
tempvec <- tempvec[!is.na(tempvec)]
d <- dist(tempvec)

h.age <- hclust(d, method = 'ward.D2') 
plot(h.age, xlab = 'Cow ID', labels = tempvec, main = 'Dendrogram of Cow Age')
```

```
temp <- cowattribdat[ , c('CowID', 'BrthDate', 'AgeDaysOld')]
rownames(temp) <- temp$CowID
temp <- temp[!is.na(temp$AgeDaysOld),]

nclust = 5
temp$AgeC<- cutree(h.age, nclust)

for(i in unique(temp$AgeC)){
  
  temp2 <- subset(temp, temp$AgeC == i)
  temp2 <- temp2[!is.na(temp2$BrthDate),]
  stringtemp <- paste(temp2$BrthDate[which.min(temp2$AgeDaysOld)], '-', 
                      temp2$BrthDate[which.max(temp2$AgeDaysOld)], sep = '')
  
  temp$AgeC[temp$AgeC == i] <- stringtemp
  
}

DMdat <- merge(DMdat, temp[,c('CowID','AgeC')], by = 'CowID', all.x = T)

temp2 <- temp[order(temp$AgeDaysOld),] 
DMdat$AgeC <- factor(DMdat$AgeC, levels = unique(temp2$AgeC), ordered = T)


# Milking Yield

tempvec <- cowattribdat$MilkYield95
names(tempvec) <- cowattribdat$CowID
tempvec <- tempvec[!is.na(tempvec)]
d <- dist(tempvec)

h.milkyield <- hclust(d, method = 'ward.D2') 
plot(h.milkyield, xlab = 'Cow ID', labels = round(tempvec,1), main = 'Dendrogram of Peak Milk Yield')
```

```
temp <- cowattribdat[ , c('CowID','MilkYield95')]
rownames(temp) <- temp$CowID
temp <- temp[!is.na(temp$MilkYield95),]

nclust = 5
temp$MilkYield95C <- cutree(h.milkyield, nclust)

for(i in 1:nclust){
  
  temp2 <- subset(temp, temp$MilkYield95C == i)
  stringtemp <- paste(round(min(temp2$MilkYield95),1), 'lb-', 
                      round(max(temp2$MilkYield95),1), 'lb', sep = '')
  
  temp$MilkYield95C[temp$MilkYield95C == i] <- stringtemp
  
}

DMdat <- merge(DMdat, temp[,c('CowID','MilkYield95C')], by = 'CowID', all.x = T)

temp2 <- temp[order(temp$MilkYield95),] 
DMdat$MilkYield95C <- factor(DMdat$MilkYield95C, levels = unique(temp2$MilkYield95C), ordered = T)


# health attribs

temp <- cowattribdat[,c('CowID','group','Sick','Sick_BurnIn','Sick_Pen','Sick_Pasture','DIGNOSIS', "Sick_Prepasture")]
DMdat <- merge(DMdat, temp, by = 'CowID', all.x = T)
DMdat$group <- as.factor(DMdat$group)

# Finalize Cow aux

rownames(DMdat) <- DMdat$CowID

auxdata <- DMdat[ , c('group', 'AgeC','CalvingDateC','MilkYield95C', "Sick_Prepasture")]
names(auxdata) <- c('Treatment', 'Age', 'CalvingDate', 'MilkYield', 'HealthStatus')


auxcol_attrib <- list(
  CalvingDate = setNames(brewer.pal(nlevels(auxdata$CalvingDate), 
                         "Blues"), levels(auxdata$CalvingDate)),
  Age = setNames(brewer.pal(nlevels(auxdata$Age), "Purples"),
                 levels(auxdata$Age)),
  MilkYield = setNames(brewer.pal(nlevels(auxdata$MilkYield), "Reds"),
                       levels(auxdata$MilkYield)),
  Treatment  = c('Control' = 'cornflowerblue', 'Organilac' = 
                  'darkolivegreen1'),
  HealthStatus = c('Sick' = 'darkorange', 'Healthy' = 'cornflowerblue')
                       )
```

## Data Wrangling - Entry Order Data

First I’ll encode all cows with complete records

```
load('../MilkOrderData.RData')
set.seed(61916)

# all cows

data.norm <- milkquantdat_sickhealthy
colnames(data.norm) <- paste('Day', as.numeric(names(data.norm)) - min(as.numeric(names(data.norm))) + 1)
data.norm <- data.norm[, !colnames(data.norm) %in% c('Day 89', 'Day 90', 'Day 91', 'Day 85') ] # drop herd-level outlier days
temp <- ifelse(as.numeric(names(milkquantdat_sickhealthy)) > 17280, 'Pasture', 'Pen')
colauxdat <- data.frame(Period = as.factor(temp))
colauxdat$DaysOnTrial <- as.numeric(names(milkquantdat_sickhealthy)) - min(as.numeric(names(milkquantdat_sickhealthy))) + 1
rownames(colauxdat) <- paste('Day', colauxdat$DaysOnTrial)


dmplot.out <- dmplot(data.norm, 
                     n_rowclusters = 10, n_colclusters = 3, 
                     maxreps = 15, 
                     annotation_row = auxdata, 
                     annotation_col = colauxdat,
                     annotation_colors = list(
                       CalvingDate = setNames(brewer.pal(nlevels(auxdata$CalvingDate), "Blues"),
                                              levels(auxdata$CalvingDate)),
                       Age = setNames(brewer.pal(nlevels(auxdata$Age), "Purples"),
                                              levels(auxdata$Age)),
                       MilkYield = setNames(brewer.pal(nlevels(auxdata$MilkYield), "Reds"),
                                              levels(auxdata$MilkYield)),
                       Treatment  = c('Control' = 'cornflowerblue', 'Organilac' = 'darkolivegreen1'),
                       Period = c('Pen' = 'cornflowerblue', 'Pasture' = 'darkolivegreen1'),
                       DaysOnTrial  = brewer.pal(colauxdat$DaysOnTrial, "Purples"),
                       HealthStatus = c('Sick' = 'darkorange', 'Healthy' = 'cornflowerblue')
                       ),
                     export_plot_final = T,
                     filename = 'Viz/OverallTB/MilkOrder/DMPlot_EntryOrder_all',
                     showplot_final = T,
                     plot_title = "Data Mechanics Plot: Parlor Entry Order of All Cows",
                     imwidth = 13,
                     imheight = 21
                     )
```

```
## Warning in if (n < 3) {: the condition has length > 1 and only the first element
## will be used
```

```
## Warning in brewer.pal(colauxdat$DaysOnTrial, "Purples"): minimal value for n is 3, returning requested palette with 3 different levels
```

```
#htree_entorder_all <- dmplot.out.all$rowtree


dmgridout_all <- dmplotGrid(data.norm, 
                     n_rowclusters = 2:10, n_colclusters = 1:6, 
                     maxreps = 15, 
                     export_plots = F,
                     showplots = F,
                     plot_title = "Data Mechanics Plot: Parlor Entry Order of All Cows",
                     imwidth = 12,
                     imheight = 21,
                     annotation_legend = F,
                     annotation_col = colauxdat,
                     annotation_colors = list(
         Period = c('Pen' = 'cornflowerblue', 
                    'Pasture' = 'darkolivegreen1'),
         DaysOnTrial  = brewer.pal(colauxdat$DaysOnTrial, 
                                   "Purples")
                     )
        )
```

```
## Warning in if (n < 3) {: the condition has length > 1 and only the first element will be used

## Warning in if (n < 3) {: minimal value for n is 3, returning requested palette with 3 different levels
```

```
# healthy cows

data.norm <- milkquantdat_all2  # milkquantdat_sickhealthy
colnames(data.norm) <- paste('Day', as.numeric(names(data.norm)) - min(as.numeric(names(data.norm))) + 1)
data.norm <- data.norm[, !colnames(data.norm) %in% c('Day 89', 'Day 90', 'Day 91', 'Day 85') ] # drop herd-level outlier days
temp <- ifelse(as.numeric(names(milkquantdat_all2)) > 17280, 'Pasture', 'Pen')
colauxdat <- data.frame(Period = as.factor(temp))
colauxdat$DaysOnTrial <- as.numeric(names(milkquantdat_all2)) - min(as.numeric(names(milkquantdat_all2))) + 1
rownames(colauxdat) <- paste('Day', colauxdat$DaysOnTrial)


dmplot.out <- dmplot(data.norm, 
                     n_rowclusters = 10, n_colclusters = 4, 
                     maxreps = 15, 
                     #verbose = T,
                     annotation_row = auxdata[,-which(names(auxdata) == "HealthStatus")], 
                     annotation_col = colauxdat,
                     annotation_colors = list(
                       CalvingDate = setNames(brewer.pal(nlevels(auxdata$CalvingDate), "Blues"),
                                              levels(auxdata$CalvingDate)),
                       Age = setNames(brewer.pal(nlevels(auxdata$Age), "Purples"),
                                              levels(auxdata$Age)),
                       MilkYield = setNames(brewer.pal(nlevels(auxdata$MilkYield), "Reds"),
                                              levels(auxdata$MilkYield)),
                       Treatment  = c('Control' = 'cornflowerblue', 'Organilac' = 'darkolivegreen1'),
                       Period = c('Pen' = 'cornflowerblue', 'Pasture' = 'darkolivegreen1'),
                       DaysOnTrial  = brewer.pal(colauxdat$DaysOnTrial, "Purples"),
                       HealthStatus = c('Sick' = 'darkorange', 'Healthy' = 'cornflowerblue')
                       ),
                     export_plot_final = T,
                     filename = 'Viz/OverallTB/MilkOrder/DMPlot_EntryOrder_healthy',
                     showplot_final = T,
                     plot_title = "Data Mechanics Plot: Parlor Entry Order of Healthy Cows",
                     imwidth = 13,
                     imheight = 15
                     )
```

```
## Warning in if (n < 3) {: the condition has length > 1 and only the first element will be used

## Warning in if (n < 3) {: minimal value for n is 3, returning requested palette with 3 different levels
```

```
#htree_entorder_healthy <- dmplot.out.healthy$rowtree


dmgridout_healthy <- dmplotGrid(data.norm, 
                     n_rowclusters = 2:10, n_colclusters = 1:6, 
                     maxreps = 15, 
                     export_plots = F,
                     showplots = F,
                     imwidth = 11,
                     imheight = 15,
                     plot_title = "Data Mechanics Plot: Parlor Entry Order of Healthy Cows",
                     annotation_col = colauxdat,
                     annotation_colors = list(
               Period = c('Pen' = 'cornflowerblue', 
                          'Pasture' = 'darkolivegreen1'),
               DaysOnTrial  = brewer.pal(colauxdat$DaysOnTrial, 
                                         "Purples")
                     )

                     )
```

```
## Warning in if (n < 3) {: the condition has length > 1 and only the first element will be used

## Warning in if (n < 3) {: minimal value for n is 3, returning requested palette with 3 different levels
```

```
save.image("HSensorData.RData")
```

# Analysis of OTB - Dissimilarity Estimators

## HClustering OTB Data

First, I want to take a look at the observed data (Po) as a heatmap with rows and columns organized by hclust trees. To do so I’ll go ahead and make a helper function that will take a distance matrix and kick back visuals and clusters that will match the order of said visual. I’ll also provide options to spit out a larger dendrogram visualization for closer inspection.

```
encodeplot <- function(datmat,
                       distrow,
                       distcol,
                       hclust_method_r = "ward.D2",
                       hclust_method_c = "ward.D2",
                       n_rowclusters = 1, 
                       n_colclusters = 1,
                       plot_title = 'Encoding Visualization',
                       showplot = TRUE, 
                       exportplot = FALSE,
                       imwidth = 10,
                       imheight = 17,
                       imres = 300,
                       filename = "EncodePlotOut",
                       rorderdend = TRUE,
                       corderdend = TRUE,
                       exportdendrogram = FALSE,
                       dendheight = 8,
                       dendwidth = 18,
                       ...){
  
  if(length(n_rowclusters) == 1 & length(n_colclusters) == 1){
      
    # create dendrograms
    
    hclustr <- hclust(distrow, method = hclust_method_r)
    hclustc <- hclust(distcol, method = hclust_method_c)
    
    # creat plots
    
    if(n_colclusters == 0){ # if no clusters, keep order of original data
      plotout <- pheatmap::pheatmap(
        mat = datmat,
        cluster_cols = FALSE,
        cluster_rows = hclustr,
        #clustering_method = "ward.D2",
        cutree_rows = n_rowclusters , 
        #cutree_cols = n_colclusters ,
        silent = T,
        main = plot_title,
        ...)$gtable
    }else{
      plotout <- pheatmap::pheatmap(
        mat = datmat,
        cluster_cols = hclustc,
        cluster_rows = hclustr,
        #clustering_method = "ward.D2",
        cutree_rows = n_rowclusters , 
        cutree_cols = n_colclusters ,
        silent = T,
        main = plot_title,
        ...)$gtable
    }
    
    
    
    if(showplot){
      grid::grid.newpage()
      grid::grid.draw(plotout)
    }
    
    if(exportplot){
      jpeg(paste(filename,'_R',n_rowclusters,'_C',n_colclusters, '.jpeg', sep = ''), 
           width = imwidth, height = imheight, units = 'in', 
           res = imres)
        grid::grid.newpage()
        grid::grid.draw(plotout)
      dev.off()
    }
    
    # return row clusters
    
    if (rorderdend){
      finaltree <- hclustr
      cutout <- dendextend::cutree(as.dendrogram(finaltree),
                                   n_rowclusters, 
                                   order_clusters_as_data = F)
      names(cutout) <- rownames(datmat)[dendextend::order.hclust(finaltree)]
      finalrowcluster <- cutout[match(rownames(datmat), names(cutout))]
    }
    else {
      finalrowcluster <- cutree(hclustr, n_rowclusters)
    }
    
    rowclustout <- finalrowcluster
  
    
    # return column clusters
    
    if(n_colclusters == 0){
      finalcolcluster <- NA
    }else if(corderdend) {
      finaltree <- hclustc
      cutout <- dendextend::cutree(as.dendrogram(finaltree),
                                   n_colclusters, order_clusters_as_data = F)
      names(cutout) <- colnames(datmat)[dendextend::order.hclust(finaltree)]
      finalcolcluster <- cutout[match(colnames(datmat), names(cutout))]
    }
    else {
      finalcolcluster <- cutree(hclustc, n_colclusters)
    }
    
    colclustout <- finalcolcluster
    
    
    listout <- list()
    listout[[paste('nrow',n_rowclusters,
                   'ncol',n_colclusters,sep ='_')]] <- list(
              rowtree = hclustr, 
              coltree = hclustc,
              plot = plotout,
              rowclust = rowclustout,
              colclust = colclustout
              )
    
    # return(list(rowtree = hclustr, 
    #           coltree = hclustc,
    #           plot = plotout,
    #           rowclust = rowclustout,
    #           colclust = colclustout
    #           ))
  
  }else{
    
    listout <- list()
    for(i in n_rowclusters){
      for(j in n_colclusters){
        
        # create dendrograms
        
        hclustr <- hclust(distrow, method = hclust_method_r)
        hclustc <- hclust(distcol, method = hclust_method_c)
        
        # create plots
        
        if(j == 0){
          plotout <- pheatmap::pheatmap(
            mat = datmat,
            cluster_cols = FALSE,
            cluster_rows = hclustr,
            #clustering_method = "ward.D2",
            cutree_rows = i , 
            #cutree_cols = j ,
            #annotation_row = auxmat_tbvar,
            #annotation_colors  = auxcol_var,
            silent = T,
            main = plot_title,
            ...)$gtable
        }else{
          plotout <- pheatmap::pheatmap(
            mat = datmat,
            cluster_cols = hclustc,
            cluster_rows = hclustr,
            #clustering_method = "ward.D2",
            cutree_rows = i , 
            cutree_cols = j ,
            #annotation_row = auxmat_tbvar,
            #annotation_colors  = auxcol_var,
            silent = T,
            main = plot_title,
            ...)$gtable
        }
        
        
        
        if(showplot){
          grid::grid.newpage()
          grid::grid.draw(plotout)
        }
        
        if(exportplot){
          jpeg(paste(filename,'_R',i,'_C',j, '.jpeg', sep = ''), 
               width = imwidth, height = imheight, units = 'in', 
               res = imres)
            grid::grid.newpage()
            grid::grid.draw(plotout)
          dev.off()
        }
        
        # return row clusters
        
        if (rorderdend) {
          finaltree <- hclustr
          cutout <- dendextend::cutree(as.dendrogram(finaltree),
                                       i, 
                                       order_clusters_as_data = F)
          names(cutout) <- rownames(datmat)[dendextend::order.hclust(finaltree)]
          finalrowcluster <- cutout[match(rownames(datmat), names(cutout))]
        }
        else {
          finalrowcluster <- cutree(hclustr, i)
        }
        
        rowclustout <- finalrowcluster
        
        
        # return column clusters
        
        if(n_colclusters == 0){
          finalcolcluster <- NA
        }else if (corderdend) {
          finaltree <- hclustc
          cutout <- dendextend::cutree(as.dendrogram(finaltree),
                                       j, 
                                       order_clusters_as_data = F)
          names(cutout) <- colnames(datmat)[dendextend::order.hclust(finaltree)]
          finalcolcluster <- cutout[match(colnames(datmat), names(cutout))]
        }
        else {
          finalcolcluster <- cutree(hclustc, j)
        }
        
        colclustout <- finalcolcluster
        
        listout[[paste('nrow',i,'ncol',j,sep ='_')]] <- list(
              rowtree = hclustr, 
              coltree = hclustc,
              plot = plotout,
              rowclust = rowclustout,
              colclust = colclustout
              )
        
      }
    }
    
  }
  
  if(exportdendrogram){
        temp <- ggdendro::ggdendrogram(hclustr, 
                                       rotate = FALSE, 
                                       label.size = 5)
        ggpubr::ggexport(temp,
                         width = dendwidth, 
                         height = dendheight, 
                         res = imres,
                         filename = paste(filename,
                                          'Dendrogram',
                                          '.pdf', sep = '_'))
    }
    
    return(listout)
  
}
```

### Euclidean Distance

I’ll start out using regular Euclidean dustance, looking at row cuts on a grid from 1:10

```
rowdisto <- dist(Po)
coldisto <- dist(t(Po))

encodeout_otb_euc <- encodeplot(Po,
                       rowdisto,
                       coldisto,
                       n_rowclusters = 10, 
                       n_colclusters = 0,
                       plot_title = 'Encoding of Overall Time Budget: Unweighted Euclidean Distance',
                       showplot = T, 
                       exportplot = T,
                       imwidth = 12,
                       imheight = 20,
                       imres = 300,
                       filename = "Viz/OverallTB/OTBEncodings/Euclidean/*OverallTB_Eucidean_TBVar",
                       exportdendrogram = T,
                       annotation_row = auxmat_tbvar,
                       annotation_colors  = auxcol_var
                       )
```

```
## file saved to Viz/OverallTB/OTBEncodings/Euclidean/*OverallTB_Eucidean_TBVar_Dendrogram_.pdf
```

```
# grid::grid.newpage()
# grid::grid.draw(encodeout_otb_euc$plot$R1_C1)


encodeout_otb_euc1 <- encodeplot(Po,
                       rowdisto,
                       coldisto,
                       n_rowclusters = 10, 
                       n_colclusters = 0,
                       plot_title = 'Encoding of Overall Time Budget: Unweighted Euclidean Distance',
                       showplot = T, 
                       exportplot = T,
                       imwidth = 12,
                       imheight = 20,
                       imres = 300,
                       filename = "Viz/OverallTB/OTBEncodings/Euclidean/*OverallTB_Eucidean_AuxVar",
                       annotation_row = auxdata,
                       annotation_colors  = auxcol_attrib)
```

```
# grid::grid.newpage()
# grid::grid.draw(encodeout_otb_euc$plot$R1_C1)

encodeout_otb_euc <- encodeplot(Po,
                       rowdisto,
                       coldisto,
                       n_rowclusters = 1:10, 
                       n_colclusters = 0,
                       plot_title = 'Encoding of Overall Time Budget: Unweighted Euclidean Distance',
                       showplot = F, 
                       exportplot = T,
                       imwidth = 12,
                       imheight = 20,
                       imres = 300,
                       filename = "Viz/OverallTB/OTBEncodings/Euclidean/OverallTB_Eucidean")

#htree_otb_euc <- encodeout_otb_euc$rowtree
```

Heatmaps are definitely a superior visualization technique for identifying paterns in overall time budgets than conventional bar charts or pie charts. These plots still clearly show the tradeoffs between behavioral investments without obscuring the individual variability. We can see from this heatmap that there is quite a bit of heterogeneity in time budgets across this herd, even within the relatively short time interval that they have been observed. While the dendrogram on the row margin helps to bring out this heterogeneity between cows, there are some clear limitations to the clusterings produced by standard euclidean dissimilarity values.

Moving down the tree helps to convey what features are the most distinctive within the herd, and in doing reveals what behaviors are having the most influence on the clustering. In doing so we see that time spent eating, which is both high in magnitude and heterogeneity, is alsost exlclusively driving the divisions between cows clusters. Time spent ruminating, which is high in magnitude but fairly homogenous, and time spent nonactive with is also quite variable between cows but somewhat lower in magnitude, only come into play in much farther down the tree. And the two activity axes, which are quite low in magnitude, are completely ignored.

This is dynamic attributable to two notable shortcomings of simple euclidean dissimilarity metrics. The first is that there is no intrinsc scaling between axes that differ in magnitude. A difference of 5 minutes spent highly active between two cows, where the average cow seldom spends more than 10 minutes a day highly active, and a difference in 5 minutes spent ruminating, where cows might commonly spend 500+ minutes a day ruminating, are going to be given equal weight in calculating the geometric distance between animals in this “behavioral space”. We could get around this issue by rescaling each behavioral axis indpendently to have equivalent magnitudes of variance and thus receive equal weights. But there are several drawbacks to this approach. The most obvious is that the time budgts will no longer scale to 1, making it more difficult to interpret the behavioral tradeoffs. More philisophically, while we shouldn’t entirely ignore a behavior just because it occupies a relatively smaller portion of a cow’s daily time budget, does it make any more sense to give such a behavior the same weight as a behavior that occupies the majority of a cow’s time?

The second drawback is that a euclidean distance cannot “see” the domain constraints intrinsic to multinomial-distributed data. That means that differences between observations are given equal weight no matter where they lie on the space. Why does that matter. Lets say within a large herd I have to dominant cows that get their pick of bunk spaces. One cow spends 14 hours a day lying down and the other spends 12. Since both cows are getting plenty of rest, we’d probably say intutively that there really isn’t any meaningful difference between these two cows, at least not for this behavioral axis. But lets say within this same over-stocked herd I have two heifers, one who only gets to lie down for 6 hours a day and the other for 8. Because these two gals are getting so little opportunity to get off their feet, we’d intutively say that the two hour difference here might now have a pretty big impact on their welfare. But with euclidean distance, and hour is an hour, and so is as likely to divide our two senior ladies as it is our two footsore heifers.

### Kullback-Leibler Divergence

Now, instead of using something a genertic as Euclidean Distance, I want to try a distance measure that is specificially made to compare probability distributions. For any two probability distributions defined over the same alphabet (ie - the same set of discrete behaviors), we can calculate the relative entropy (or Kullback-Leibler divergence).

The benefit of this metric is intrinsically designed for working with multinomial data (ie - proportions that sum to 1). And so, it can see and account for how close two points are to the domain constraints. By utilizing logarithms we are effectively going to “blow up” the distances as they approach the boundary of the space to give them a bit more room to breath. So if cows typically spend 50% of their day doing one behavior and it drops down to 25% of their time, that will have a more equitable impact as if cows typically spent 10% of their time active and that suddenly drops to 5%. This will help to scale magnitudes not only between but also within a behavioral axis.

First I need to define the distance function.

```
relativeEntropy <- function(dist1, dist2, logbase = 2){
   
     if( !1%in%dim(dist1) | !1%in%dim(dist2)){
       stop('Error: Distributions must be provided as vectors')
     }

     if(sum(dist1) != 1  |  sum(dist2) != 1){
       stop('Error: Distributions do not sum to 1')
     }
    
    return( sum(dist1 * log(dist1/dist2, base = logbase)) )
  
}

KLDdist <- function(dist1, dist2 = NULL, returnSymetric = T, runfast = F, logbase = 2){
  
  if(!is.null(dist2)){ #distance betwee two vectors
    
    return( sum(dist1 * log(dist1/dist2, base = logbase)) )
  
  }else{ # compute distance matrix for rows (obs) of data matrix
    
    if(sum(is.na(dist1)) > 0){
      stop('Error: Data contains missing NA values')
    }
    
    if( sum(round(apply(dist1, 1, sum), 5) != 1) > 0 ){
      #print(apply(dist1, 1, sum)[apply(dist1, 1, sum) != 1])
      stop('Error: All row must sum to 1 to be a legitimate probability distribution')
    }

    if(runfast){
      dout <- Rfast::Dist(dist1, method = 'kullback_leibler')
      rownames(dout) <- rownames(dist1)
      colnames(dout) <- rownames(dist1)
      #NOTE: Only returns symmetric KL dist, and can't set the log base
    }else{
      dout <- apply(dist1, 1, function(x_i) sapply(1:nrow(dist1), function(j) KLDdist(x_i, dist1[j, ], logbase = logbase))) 

    }
    
    #dout <- matrix(0, nrow = nrow(dist1), ncol = nrow(dist1))
    
    
    if(returnSymetric){
      return(as.dist(dout + t(dout)))
    }else{
      return(as.dist(dout))
    }
    
  }
  
  
  
}

dist1 <- Po[1,]
dist2 <- Po[2,]

relativeEntropy(dist1, dist2)
```

```
## [1] 0.041078
```

```
relativeEntropy(dist2, dist1)
```

```
## [1] 0.04284475
```

```
start_time <- Sys.time()
temp <- KLDdist(Po)
as.matrix(temp)[1:5,1:5]
```

```
##              5        443        449       645        677
## 5   0.00000000 0.08392276 0.06419955 0.2924512 0.10863894
## 443 0.08392276 0.00000000 0.01601282 0.5292662 0.09200215
## 449 0.06419955 0.01601282 0.00000000 0.5670085 0.03877881
## 645 0.29245119 0.52926624 0.56700846 0.0000000 0.76831561
## 677 0.10863894 0.09200215 0.03877881 0.7683156 0.00000000
```

```
end_time <- Sys.time()
end_time - start_time
```

```
## Time difference of 37.81266 secs
```

```
start_time <- Sys.time()
temp <- KLDdist(Po, runfast = T)
as.matrix(temp)[1:5,1:5]
```

```
##              5        443        449       645        677
## 5   0.00000000 0.11634165 0.08899948 0.4054234 0.15060555
## 443 0.11634165 0.00000000 0.02219848 0.7337188 0.12754206
## 449 0.08899948 0.02219848 0.00000000 0.7860406 0.05375884
## 645 0.40542344 0.73371881 0.78604063 0.0000000 1.06511160
## 677 0.15060555 0.12754206 0.05375884 1.0651116 0.00000000
```

```
end_time <- Sys.time()
end_time - start_time
```

```
## Time difference of 0.08006501 secs
```

And now now lets try clustering with KLD

```
rowdisto <- KLDdist(Po, runfast = T)
coldisto <- dist(t(Po))

encodeout_otb_kld1 <- encodeplot(Po,
                       rowdisto,
                       coldisto,
                       n_rowclusters = 10, 
                       n_colclusters = 0,
                       plot_title = 'Encoding of Overall Time Budget: KL Distance',
                       showplot = T, 
                       exportplot = T,
                       imwidth = 12,
                       imheight = 20,
                       imres = 300,
                       filename = "Viz/OverallTB/OTBEncodings/KLDivergence/*OverallTB_KLD_TBVar",
                       annotation_row = auxmat_tbvar,
                       annotation_colors  = auxcol_var,
                       exportdendrogram = T)
```

```
## file saved to Viz/OverallTB/OTBEncodings/KLDivergence/*OverallTB_KLD_TBVar_Dendrogram_.pdf
```

```
# grid::grid.newpage()
# grid::grid.draw(encodeout_otb_kld$plot$R1_C1)


encodeout_otb_kld <- encodeplot(Po,
                       rowdisto,
                       coldisto,
                       n_rowclusters = 10, 
                       n_colclusters = 0,
                       plot_title = 'Encoding of Overall Time Budget: KL Distance',
                       showplot = T, 
                       exportplot = T,
                       imwidth = 12,
                       imheight = 20,
                       imres = 300,
                       filename = "Viz/OverallTB/OTBEncodings/KLDivergence/*OverallTB_KLD_AuxVar",
                       annotation_row = auxdata,
                       annotation_colors  = auxcol_attrib)
```

```
# grid::grid.newpage()
# grid::grid.draw(encodeout_otb_kld$plot$R1_C1)

encodeout_otb_kld <- encodeplot(Po,
                       rowdisto,
                       coldisto,
                       n_rowclusters = 1:10, 
                       n_colclusters = 0,
                       plot_title = 'Encoding of Overall Time Budget: KL Distance',
                       showplot = F, 
                       exportplot = T,
                       imwidth = 12,
                       imheight = 20,
                       imres = 300,
                       filename = "Viz/OverallTB/OTBEncodings/KLDivergence/OverallTB_KLD")

#htree_otb_kld <- encodeout_otb_kld$rowtree
```

As we might have expected, KL distance is providing more distinctive clussters by more equally leveraging information across all five behavioral axes, including the low magnitude activity axes. In particular we see its prioritizing differences between cows with more extreme time budgets, and is placing far less emphasis on differences between the cows with relatively balanced tradeoffs between eating/ruminating/nonactive. This can bee seen perhaps the most clearly between the euclidean and kl encodings at R6.

One potential shortcoming of this metric, however, is that while it can take into account better the nuances of multinomial-distributed data, it cannot account for any heterogeneity in variability attributable to the sensor. We see in this example that it is producing a very fine encoding of animals at the extreme ends of the time budget distribution, but just because these animals have more extreme values does not necessarily mean we are more confident in the accuracy of their measurements. So is this finer stratification at the extremes of the sample capturing authentic differences between cows, or is it finding fluctuations in the stochastic weeds below the accuracy threshold of the sensor.

### Ensemble Weighted Euclidean Distance

By simulating the original data, we can rescale the dissimilarity between cows by their respective ensemble variances (analogous to an anova). This should accomplish three desireable effects. First, each behavioral axis should be scaled to a unitless value with similar magnitudes and weights. Unlike with standard scaling, however, axes that are intrinsically more vairable with a less resilient/replicable biological signal will be penalizded, so a behavioral axis with weaker behavioral signal will not be artificially inflated in weight in calculating overall similarity. Finally, because these values are calculated for each cow, this should also serve to adjust the scaling relative the magnitude of the behavior in that individuals time budget - so while an ensemble euclidean distance will not be able to “see” the boundary constraints formally we will be able to approximate the rescaling effect at the boundary as in the KL Distances.

Alright, first I need a function that will allow me to simulate this data set at each hour. Obs (cows) will go on the rows. Time points will go on the columns. Behaviors axes (distributed multinomial) will go on the last axis. And finally each simulation will be indexed on the fourth and final axis.

Seting the time dimension on the second dimension will allow be to acomodate any dimensionality of data. I will simulate using the granularity at which the data was logged. I’ll then add in post-processing options to output aggregated time budgets. Options will either be 1) ‘NA’ for no aggreggation, and the sim will ouput data identical in format to the input tensor; 2) ‘Overall’ to completely collapse the time axis, so that the data is output as only a 3D tensor with simulations still on the final axis; 3) aggregate the time axis using a grouping variable, which is provided as a vector of length equal to that of the input time dimesion.

Note: Resimulating the sample from the MVB/MN give use some idea of uncertainty in over-all time budget due to sampling. If we record an extremely large number of samples, howver, central limit theorem will eventually kick in, and OTB will not vary much at all, which might give use OTB that are significantly distinct statistically, but not practically different. For such large datasets where this comes into effect, we may have sufficient replications to also explore the behavioral consitency. By subsampling over the time interval with each new ensemble, we can explore how consistent the time budget of each cow is temporally. If a cow does not show a distinct time budget across days, we will be be more conservative (less inclined) to say she is distinct from other cows with similar overall time budgets.

```
B = 100 # number of simulated datasets
m = 5 # number of behavioral axes

# format data as tensor

sensordat <- array(NA, 
                   dim = c(ncol(activedat[,-c(1,2,3,4)]), 
                           nrow(activedat[,-c(1,2,3,4)]),
                           m),
                   dimnames = list(names(activedat[,-c(1,2,3,4)]), 
                                activedat$TimeDiff,
                                names(Po)
                                )
                   )

sensordat[ , , 'Active'] <- t(activedat[ , -c(1,2,3,4) ])
sensordat[ , , 'HighlyActive'] <- t(hiactivedat[ , -c(1,2,3,4) ])
sensordat[ , , 'Nonactive'] <- t(nonactivedat[ , -c(1,2,3,4) ])
sensordat[ , , 'Rumination'] <- t(rumdat[ , -c(1,2,3,4) ])
sensordat[ , , 'Eating'] <- t(eatdat[ , -c(1,2,3,4) ])

sensordatcol_day <- activedat$Date
sensordatcol_hour <- activedat$Hour

# simulate data

# simMethod = 'DUAL'
# simMethod = 'MN'
# simMethod = 'MVB'

# dat <- Co
# dat <- sensordat
# agVar <- sensordatcol_day

# NTempSubsamp = 10
# subsampVar <- sensordatcol_day

simTimeBudget <- function(dat, B,
                          simMethod = 'DUAL',
                          NTempSubsamp = NULL,
                          subsampVar = NULL,
                          aggregateTB = NULL,
                          agVar = NULL,
                          verbose = T){
  
  if(length(dim(dat)) == 2){ # simulate overall time budget
    
    matsim <- matrix(NA, nrow = nrow(dat), ncol = ncol(dat))
    rownames(matsim) <- rownames(dat)
    
    
    if(simMethod == 'DUAL'){
      matsim <- t(apply(dat, 1, function(x) 
        rmultinom(1, 
                  size = sum(x), 
                  prob = DirichletReg::rdirichlet(1,
                                                  alpha = (x+1) 
                  ))
      ))
      colnames(matsim) <- colnames(dat)
    }else if(simMethod == 'MN'){
      matsim <- t(apply(dat, 1, function(x) 
        rmultinom(1, 
                  size = sum(x), 
                  prob = x/sum(x) 
                  )
      ))
      colnames(matsim) <- colnames(dat)
    }else if(simMethod == 'MVB'){
      matsim <- t(apply(dat, 1, function(x)
        DirichletReg::rdirichlet(1, alpha = (x+1) )
      ))
      colnames(matsim) <- colnames(dat)
    }else{
      stop('Error: simMethod option not recognized')
    }
    
    
    
    tbsim <- t(apply(matsim, 1, function(x) x/sum(x)))
    return(DataSim = matsim, TBSim = tbsim)

    
  }else{ #simulate time budget tensor
    
    tensorsim <- array(NA, 
                       dim = c(dim(dat)[1], 
                               dim(dat)[2],
                               dim(dat)[3],
                               B),
                       dimnames = list(unlist(dimnames(dat)[1]), 
                                       unlist(dimnames(dat)[2]),
                                       unlist(dimnames(dat)[3]),
                                       1:B)
    )
    
    if(is.null(aggregateTB) | verbose){ # skip creating a second large matrix if we won't ever return it
      tbsim <- array(NA, 
                     dim = c(dim(dat)[1], 
                             dim(dat)[2],
                             dim(dat)[3],
                             B),
                     dimnames = list(unlist(dimnames(dat)[1]), 
                                     unlist(dimnames(dat)[2]),
                                     unlist(dimnames(dat)[3]),
                                     1:B)  )
    }
    
    
    for(b in 1:B){
      
      ### subsample time interval ###
      if(is.null(NTempSubsamp)){
        
        myT <- unlist(dimnames(dat)[2])
        
      }else if(!is.null(NTempSubsamp) & is.null(subsampVar)){
        
        # subsample from time dimension unconditionally
        temp <- sample(unlist(dimnames(dat)[2]), 
                       NTempSubsamp,
                       replace = F)
        myT <- unlist(dimnames(dat)[2])[unlist(dimnames(dat)[2]) 
                                        %in% temp]
        
      }else{
        
        if(length(subsampVar) != dim(dat)[2]){
          stop('Error: Dimension of subsample indexing variable must match length of temporal dimension')
        }
        
        temp <- sample(unique(subsampVar), 
                       NTempSubsamp,
                       replace = F)
        myT <- unlist(dimnames(dat)[2])[subsampVar %in% temp]
        
      }
      
        
      ### resample obs data ###  
        
      if(simMethod == 'DUAL'){ 
      
        #tensorsim <- tensorsim[,myT,,]
        tensorsim[,myT,,b] <- aperm(apply(dat[,myT,], c(1,2), 
             function(x) rmultinom(1,
                size = sum(x),
                prob = DirichletReg::rdirichlet(1,
                                                alpha = (x+1) ))),
                       # convert to dierchlets mean
          perm = c(2,3,1))
      
      }else if(simMethod == 'MN'){
        
        #tensorsim <- tensorsim[,myT,,]
        tensorsim[,myT,,b] <- aperm(apply(dat[,myT,], c(1,2), 
             function(x) rmultinom(1,
                size = sum(x),
                prob = x/sum(x) )),
                       # convert to dierchlets mean
          perm = c(2,3,1))
      
      }else if(simMethod == 'MVB'){
      
        #tensorsim <- tensorsim[,myT,,]
        tensorsim[,myT,,b] <- aperm(apply(dat[,myT,], c(1,2), 
               function(x)
                    DirichletReg::rdirichlet(1, alpha = (x+1) )),
              perm = c(2,3,1))
      
      }else{
        stop('Error: simMethod option not recognized')
      }
      
      
      if(is.null(aggregateTB) | verbose){
        tbsim[,myT,,b] <- aperm(apply(tensorsim[,myT,,b], c(1,2), 
                               function(x) x/sum(x)),
                          perm = c(2,3,1))
      }
      
    }
    
    
    
    
    # View(dat[,1,,b])
    # View(tensorsim[,1,,b])
    # View(tbsim[,1,,b])
    # apply(tbsim[,1,,b], 1, sum)
    
    
    # for(i in 1:dim(tensorsim)[1]){
    #   for(j in 1:dim(tensorsim)[2]){
    #     for(b in 1:B){
    #       
    #       temp <- dat[i,j, ]
    #       tensorsim[i,j, ,b] <- rmultinom(1,
    #                              size = sum(temp),
    #                              prob = DirichletReg::rdirichlet(1,
    #                                        alpha = (temp+1) )
    #                                      )
    #       tbsim[i,j, ,b] <- tensorsim[i,j, ,b]/sum(temp)
    #       
    #          
    #     }
    #   }
    # }
    
    
    
  }
  
  if(is.null(aggregateTB)){ # do not aggregate data by any time dimension
    
    return(list(DataSim = tensorsim, 
                TBSim = tbsim, 
                SimSpecs = list(simMethod = simMethod,
                          NTempSubsamp = NTempSubsamp,
                          subsampVar = subsampVar,
                          aggregateTB = aggregateTB,
                          agVar = agVar)))
    
  }else if(aggregateTB == 'overall'){
    
    tempc <- apply(tensorsim, c(1,3,4), 
                   function(x) sum(x, na.rm = T)) 
    tempp <- aperm(apply(tempc, c(1,3), 
                         function(x) x/sum(x, na.rm = T)),
                   perm = c(2,1,3))
    #View(Po)
    #View(tempp)
    
    if(verbose){
      return(list(DataSim = tempc, 
                  TBSim = tempp,
                  FullSimResults = list(DataSim = tensorsim, 
                                        TBSim = tbsim),
                  SimSpecs = list(simMethod = simMethod,
                          NTempSubsamp = NTempSubsamp,
                          subsampVar = subsampVar,
                          aggregateTB = aggregateTB,
                          agVar = agVar)
                ))
    }else{
      return(list(DataSim = tempc, 
                  TBSim = tempp,
                  SimSpecs = list(simMethod = simMethod,
                          NTempSubsamp = NTempSubsamp,
                          subsampVar = subsampVar,
                          aggregateTB = aggregateTB,
                          agVar = agVar)))
    }
    
    
  }else if(aggregateTB == 'byvar'){
    
    if(is.null(agVar)){
      stop('Error: Provide vector with grouping variable for column indeces')
    }
    if(length(agVar) != dim(dat)[2]){
      stop('Error: AgVar vector does not match length of time dimension for data set provided')
    }
    
    tempc <- aperm(apply(tensorsim, c(1,3,4), function(x)
                                              tapply(x,agVar, sum)),
                   perm = c(2,1,3,4))
    tempp <- aperm(apply(tempc, c(1,2,4), function(x) x/sum(x)),
                   perm = c(2,3,1,4))

    
    if(verbose){
      return(list(DataSim = tempc, 
                  TBSim = tempp,
                  FullSimResults = list(DataSim = tensorsim, 
                                        TBSim = tbsim),
                  SimSpecs = list(simMethod = simMethod,
                          NTempSubsamp = NTempSubsamp,
                          subsampVar = subsampVar,
                          aggregateTB = aggregateTB,
                          agVar = agVar)
                ))
    }else{
      return(list(DataSim = tempc, 
                  TBSim = tempp,
                  SimSpecs = list(simMethod = simMethod,
                          NTempSubsamp = NTempSubsamp,
                          subsampVar = subsampVar,
                          aggregateTB = aggregateTB,
                          agVar = agVar)))
    }
    
    
  } 
  
    
}


# sim full data set and compress to overall TB

temp <- simTimeBudget(sensordat, B = 50, aggregateTB = 'overall')

hist(temp$FullSimResults$TBSim[1,1,1,])
abline(v = sensordat[1,1,1]/sum(sensordat[1,1,]), col = 'red')
```

```
hist(temp$TBSim[1,1,])
abline(v = Po[1,1])
```

```
max(temp$TBSim[1,1,]) - min(temp$TBSim[1,1,])
```

```
## [1] 0.004130783
```

```
max(temp$TBSim[1,4,]) - min(temp$TBSim[1,4,])
```

```
## [1] 0.006966051
```

```
plot(Po[,1], temp$TBSim[,1,1], main = 'Activity')
```

```
plot(Po[,2], temp$TBSim[,2,2], main = 'High Activity')
```

```
plot(Po[,3], temp$TBSim[,3,3], main = 'Nonactivity')
```

```
plot(Po[,4], temp$TBSim[,4,4], main = 'Rumination')
```

```
plot(Po[,5], temp$TBSim[,5,5], main = 'Eating')
```

```
pheatmap(Po)
```

```
pheatmap(temp$TBSim[,,1])
```

```
# sim subset of data set sampled by day to overallTB
 

temp2 <- simTimeBudget(sensordat, B = 50, 
                      NTempSubsamp = 30,
                      subsampVar = sensordatcol_day,
                      aggregateTB = 'overall')

hist(temp2$FullSimResults$TBSim[1,1,1,])
abline(v = sensordat[1,1,1]/sum(sensordat[1,1,]), col = 'red')
```

```
hist(temp2$TBSim[1,4,])
abline(v = Po[1,4])
```

```
max(temp2$TBSim[1,1,]) - min(temp2$TBSim[1,1,])
```

```
## [1] 0.009111975
```

```
max(temp2$TBSim[1,4,]) - min(temp2$TBSim[1,4,])
```

```
## [1] 0.02344176
```

```
# check if the overall averages match the observed

plot(Po[,1], temp2$TBSim[,1,1], main = 'Activity')
```

```
plot(Po[,2], temp2$TBSim[,2,2], main = 'High Activity')
```

```
plot(Po[,3], temp2$TBSim[,3,3], main = 'Nonactivity')
```

```
plot(Po[,4], temp2$TBSim[,4,4], main = 'Rumination')
```

```
plot(Po[,5], temp2$TBSim[,5,5], main = 'Eating')
```

```
pheatmap(Po)
```

```
pheatmap(temp2$TBSim[,,1])
```

Righto, so now lets create the simulations I want to use to create my ensemble distances

```
# simulate all observations

set.seed(61916)

simotb <- simTimeBudget(sensordat, 
                        B = 500, 
                        aggregateTB = 'overall', 
                        verbose = F)

pryr::object_size(simotb)
```

```
## 5.36 MB
```

```
# ensembleByHour = simotb$DataSim
# ensemblePmat_nth <- simotb$TBSim

# pheatmaps

set.seed(61916)

simotb_tsub <- simTimeBudget(sensordat, 
                        B = 500, 
                        NTempSubsamp = 14,
                        subsampVar = sensordatcol_day,
                        aggregateTB = 'overall', 
                        verbose = F)
```

And finally I need a function to calculate distances reweighted by the ensemble variance.

Note: As a speed up option, the ensemble variances used to scale each index in the dist matrix can be provided to avoid recalculation. If these values are being calculated for the first time, and you want to pass them into subsequent function, the verbose arguement will return a list with both the ensemble dist matrix and the matrix of scaling factors. If not verbose, only the distmat is returned.

```
## need to add check that dim names match
## need to fix weightdist calf for daily tb

#  po <- Po
#  pb <- simotb$TBSim

# po <- Po_day
# pb <- Pb_day

ensembledist <- function(po, pb, weightdist = NULL , verbose = F){
  #po = matrix/tensor of observed values
  #pb = matrix/tensor of simulated values
  
  B = dim(pb)[length(dim(pb))] # size of ensemble 
  
  distout <- matrix(0, nrow = nrow(po), ncol = nrow(po))
  rownames(distout) <- rownames(po)
  colnames(distout) <- rownames(po)
  
  if(length(dim(po)) == 2){
    
    if(is.null(weightdist)){
      
    #   weightdist <- po
    #   weightdist[,] <- NA 
    #   
    #   for(i in 1:nrow(po)){
    #     for(j in 1:ncol(po)){
    #       weightdist[i,j] <- sum((po[i,j] - pb[i,j, ])^2)/B
    #     }
    #   }
    #   
    
      weightdist <- apply(pb, c(1,2), var) 
      
    }
      
    
    
    # start_time <- Sys.time()
    # for(i in 1:nrow(po)){
    #   for(j in i:nrow(po)){
    #     
    #     distout[i,j] <- sum((po[i,] - po[j,])^2/
    #                           (weightdist[i,] + weightdist[j,])
    #                         )
    #     distout[j,i] <- distout[i,j]
    #    
    #   }
    # }
    # end_time <- Sys.time()
    # end_time - start_time
    # dist1 <- distout
    
    distout <- matrix(0, nrow = nrow(po), ncol = nrow(po))
    rownames(distout) <- rownames(po)
    colnames(distout) <- rownames(po)
  
    #start_time <- Sys.time()
    for(j in 1:ncol(po)){
      distout <- distout + 
        as.matrix(Rfast::Dist(matrix(po[,j], ncol = 1), square = T))/
        outer(weightdist[,j], weightdist[,j], FUN = '+')
    }
    # end_time <- Sys.time()
    # end_time - start_time
    # dist2 <- distout
    
    
  }else if(length(dim(po)) == 3){
    
    if(is.null(weightdist)){
      
      weightdist <- po
      weightdist[,,] <- NA 
      
      for(i in 1:nrow(po)){
        for(j in 1:ncol(po)){
          for(k in 1:dim(po)[3]){
            weightdist[i,j,k] <- sum((po[i,j,k] - pb[i,j,k, ])^2)/B
          }
        }
      }
      
    }
    
    for(i in 1:nrow(po)){
      for(j in i:nrow(po)){
        
        distout[i,j] <- sum((po[i,,] - po[j,,])^2/
                              (weightdist[i,,] + weightdist[j,,])
                            )
        distout[j,i] <- distout[i,j]
       
      }
    }
    
  }else{
    stop('Error: Incorrect number if dimensions')
  }
  
  
  if(verbose){
    return(list(dist = as.dist(distout), weights = weightdist))
  }else{
    return(as.dist(distout))
  }

}

start_time <- Sys.time()
temp <- ensembledist(Po, simotb$TBSim, verbose = T)
end_time <- Sys.time() 
end_time - start_time
```

```
## Time difference of 0.2392251 secs
```

```
as.matrix(temp$dist)[1:5, 1:5]
```

```
##            5        443        449       645       677
## 5      0.000  2191.4241  1796.2528  7471.914  3286.252
## 443 2191.424     0.0000   432.5322 13417.065  2333.736
## 449 1796.253   432.5322     0.0000 15077.543  1011.874
## 645 7471.914 13417.0654 15077.5427     0.000 19279.495
## 677 3286.252  2333.7362  1011.8737 19279.495     0.000
```

```
hist(sqrt(temp$weights[,1]), main = names(Po)[1])
```

```
hist(sqrt(temp$weights[,2]), main = names(Po)[2])
```

```
hist(sqrt(temp$weights[,3]), main = names(Po)[3])
```

```
hist(sqrt(temp$weights[,4]), main = names(Po)[4])
```

```
hist(sqrt(temp$weights[,5]), main = names(Po)[5])
```

And now I’ll visualize using the ensemble weighted distance from the temporally heterogenous simulation of the complete sample

```
ensembleweightOTB <- ensembledist(Po, simotb$TBSim, verbose = T)
rowdisto <- ensembleweightOTB$dist
coldisto <- dist(t(Po))

encodeout_otb_ew <- encodeplot(Po,
                       rowdisto,
                       coldisto,
                       n_rowclusters = 10, 
                       n_colclusters = 0,
                       plot_title = 'Encoding of Overall Time Budget: Noise-Penalized Ensemble Weighted Distance',
                       showplot = T, 
                       exportplot = T,
                       imwidth = 12,
                       imheight = 20,
                       imres = 300,
                       filename = "Viz/OverallTB/OTBEncodings/NoisePenalized/*OverallTB_EW_TBVAR",
                       annotation_row = auxmat_tbvar,
                       annotation_colors  = auxcol_var,
                       exportdendrogram = T)
```

```
## file saved to Viz/OverallTB/OTBEncodings/NoisePenalized/*OverallTB_EW_TBVAR_Dendrogram_.pdf
```

```
# grid::grid.newpage()
# grid::grid.draw(encodeout_otb_ew$plot$R1_C1)

npweights <- as.data.frame(log(ensembleweightOTB$weights))
names(npweights) <- paste('EW_',names(npweights), sep = '')
auxcol_np <- list(
  EW_Active = brewer.pal(npweights$EW_Active, "Purples"),
  EW_HighlyActive = brewer.pal(npweights$EW_HighlyActive, "Purples"),
  EW_Nonactive = brewer.pal(npweights$EW_Nonactive, "Purples"),
  EW_Rumination = brewer.pal(npweights$EW_Rumination, "Purples"),
  EW_Eating = brewer.pal(npweights$EW_Eating, "Purples")
)
```

```
## Warning in if (n < 3) {: the condition has length > 1 and only the first element
## will be used
```

```
## Warning in brewer.pal(npweights$EW_Active, "Purples"): minimal value for n is 3, returning requested palette with 3 different levels
```

```
## Warning in if (n < 3) {: the condition has length > 1 and only the first element
## will be used
```

```
## Warning in brewer.pal(npweights$EW_HighlyActive, "Purples"): minimal value for n is 3, returning requested palette with 3 different levels
```

```
## Warning in if (n < 3) {: the condition has length > 1 and only the first element
## will be used
```

```
## Warning in brewer.pal(npweights$EW_Nonactive, "Purples"): minimal value for n is 3, returning requested palette with 3 different levels
```

```
## Warning in if (n < 3) {: the condition has length > 1 and only the first element
## will be used
```

```
## Warning in brewer.pal(npweights$EW_Rumination, "Purples"): minimal value for n is 3, returning requested palette with 3 different levels
```

```
## Warning in if (n < 3) {: the condition has length > 1 and only the first element
## will be used
```

```
## Warning in brewer.pal(npweights$EW_Eating, "Purples"): minimal value for n is 3, returning requested palette with 3 different levels
```

```
encodeout_otb_ew1 <- encodeplot(Po,
                       rowdisto,
                       coldisto,
                       n_rowclusters = 10, 
                       n_colclusters = 0,
                       plot_title = 'Encoding of Overall Time Budget: Noise-Penalized Ensemble Weighted Distance',
                       showplot = T, 
                       exportplot = T,
                       imwidth = 12,
                       imheight = 20,
                       imres = 300,
                       filename = "Viz/OverallTB/OTBEncodings/NoisePenalized/*OverallTB_EW_EWVAR",
                       annotation_row = npweights,
                       annotation_colors  = auxcol_np)
```

```
encodeout_otb_ew <- encodeplot(Po,
                       rowdisto,
                       coldisto,
                       n_rowclusters = 10, 
                       n_colclusters = 0,
                       plot_title = 'Encoding of Overall Time Budget: Noise-Penalized Ensemble Weighted Distance',
                       showplot = T, 
                       exportplot = T,
                       imwidth = 12,
                       imheight = 20,
                       imres = 300,
                       filename = "Viz/OverallTB/OTBEncodings/NoisePenalized/*OverallTB_EW_AuxVar",
                       annotation_row = auxdata,
                       annotation_colors  = auxcol_attrib)
```

```
# grid::grid.newpage()
# grid::grid.draw(encodeout_otb_ew$plot$R1_C1)

encodeout_otb_ew <- encodeplot(Po,
                       rowdisto,
                       coldisto,
                       n_rowclusters = 1:10, 
                       n_colclusters = 0,
                       plot_title = 'Encoding of Overall Time Budget: Noise-Penalized Ensemble Weighted Distance',
                       showplot = F, 
                       exportplot = T,
                       imwidth = 12,
                       imheight = 20,
                       imres = 300,
                       filename = "Viz/OverallTB/OTBEncodings/NoisePenalized/OverallTB_EW")

#htree_otb_ew <- encodeout_otb_ew$rowtree
```

This has produced quite a nice encoding. Whereas both euclidean and kld are both almost entirely pre-occupied with heterogenity in eating times for the first handful of cuts, the ensemble reweighting seems to have produce a more behaviorally balanced encoding.

As before, the first cut seperates the cows with low eating times from high, but the next few cuts then turn to extremes in nonactivity patterns. Nor do we get the over-stratification of the extreme groups, like we saw with kld, but instead get a finer striations within the intermediate group that seem reasonably well balanced between eating, rumination, and nonactivity, with the high activity axis coming into play in later cuts.

And finally I’ll visualize using the ensemble weighted distance from the temporally heterogenous simulation of the temporally subsampled data, penalizing for plasticity in OTB

```
plastweightOTB <- ensembledist(Po, simotb_tsub$TBSim, verbose = T)
rowdisto <- plastweightOTB$dist
coldisto <- dist(t(Po))

encodeout_otb_pw <- encodeplot(Po,
                       rowdisto,
                       coldisto,
                       n_rowclusters = 10, 
                       n_colclusters = 0,
                       plot_title = 'Encoding of Overall Time Budget: Plasticity-Penalized Ensemble Weighted Distance',
                       showplot = T, 
                       exportplot = T,
                       imwidth = 12,
                       imheight = 20,
                       imres = 300,
                       filename = "Viz/OverallTB/OTBEncodings/PlasticityPenalized/*OverallTB_PW_TBVAR",
                       annotation_row = auxmat_tbvar,
                       annotation_colors  = auxcol_var,
                       exportdendrogram = T)
```

```
## file saved to Viz/OverallTB/OTBEncodings/PlasticityPenalized/*OverallTB_PW_TBVAR_Dendrogram_.pdf
```

```
# grid::grid.newpage()
# grid::grid.draw(encodeout_otb_ew$plot$R1_C1)

ppweights <- as.data.frame(log(plastweightOTB$weights))
names(ppweights) <- paste('EW_',names(ppweights), sep = '')
auxcol_pp <- list(
  EW_Active = brewer.pal(ppweights$EW_Active, "Purples"),
  EW_HighlyActive = brewer.pal(ppweights$EW_HighlyActive, "Purples"),
  EW_Nonactive = brewer.pal(ppweights$EW_Nonactive, "Purples"),
  EW_Rumination = brewer.pal(ppweights$EW_Rumination, "Purples"),
  EW_Eating = brewer.pal(ppweights$EW_Eating, "Purples")
)
```

```
## Warning in if (n < 3) {: the condition has length > 1 and only the first element
## will be used
```

```
## Warning in brewer.pal(ppweights$EW_Active, "Purples"): minimal value for n is 3, returning requested palette with 3 different levels
```

```
## Warning in if (n < 3) {: the condition has length > 1 and only the first element
## will be used
```

```
## Warning in brewer.pal(ppweights$EW_HighlyActive, "Purples"): minimal value for n is 3, returning requested palette with 3 different levels
```

```
## Warning in if (n < 3) {: the condition has length > 1 and only the first element
## will be used
```

```
## Warning in brewer.pal(ppweights$EW_Nonactive, "Purples"): minimal value for n is 3, returning requested palette with 3 different levels
```

```
## Warning in if (n < 3) {: the condition has length > 1 and only the first element
## will be used
```

```
## Warning in brewer.pal(ppweights$EW_Rumination, "Purples"): minimal value for n is 3, returning requested palette with 3 different levels
```

```
## Warning in if (n < 3) {: the condition has length > 1 and only the first element
## will be used
```

```
## Warning in brewer.pal(ppweights$EW_Eating, "Purples"): minimal value for n is 3, returning requested palette with 3 different levels
```

```
encodeout_otb_pw1 <- encodeplot(Po,
                       rowdisto,
                       coldisto,
                       n_rowclusters = 10, 
                       n_colclusters = 0,
                       plot_title = 'Encoding of Overall Time Budget: Plasticity-Penalized Ensemble Weighted Distance',
                       showplot = T, 
                       exportplot = T,
                       imwidth = 12,
                       imheight = 20,
                       imres = 300,
                       filename = "Viz/OverallTB/OTBEncodings/PlasticityPenalized/*OverallTB_PW_EWVAR",
                       annotation_row = ppweights,
                       annotation_colors  = auxcol_pp)
```

```
encodeout_otb_pw <- encodeplot(Po,
                       rowdisto,
                       coldisto,
                       n_rowclusters = 10, 
                       n_colclusters = 0,
                       plot_title = 'Encoding of Overall Time Budget: Plasticity-Penalized Ensemble Weighted Distance',
                       showplot = T, 
                       exportplot = T,
                       imwidth = 12,
                       imheight = 20,
                       imres = 300,
                       filename = "Viz/OverallTB/OTBEncodings/PlasticityPenalized/*OverallTB_PW_AuxVar",
                       annotation_row = auxdata,
                       annotation_colors  = auxcol_attrib)
```

```
# grid::grid.newpage()
# grid::grid.draw(encodeout_otb_ew$plot$R1_C1)


encodeout_otb_pw <- encodeplot(Po,
                       rowdisto,
                       coldisto,
                       n_rowclusters = 1:10, 
                       n_colclusters = 0,
                       plot_title = 'Encoding of Overall Time Budget: Plasticity-Penalized Ensemble Weighted Distance',
                       showplot = F, 
                       exportplot = T,
                       imwidth = 12,
                       imheight = 20,
                       imres = 300,
                       filename = "Viz/OverallTB/OTBEncodings/PlasticityPenalized/OverallTB_PW")
```

#### Comparisons of Ensemble Weights

First I want to compare variance estimates extracted from the noise-penalized and plasticity-penalized simulations.

```
qplot(log(ensembleweightOTB$weights[,1]), 
      log(plastweightOTB$weights[,1]), 
      main = names(Po)[1], 
      xlab = 'Noise-Penalized Variance (log-scale)', 
      ylab = 'Plasticity-Penalized Variance (log-scale)')
```

```
qplot(log(ensembleweightOTB$weights[,2]), 
      log(plastweightOTB$weights[,2]), 
      main = names(Po)[2], 
      xlab = 'Noise-Penalized Variance (log-scale)', 
      ylab = 'Plasticity-Penalized Variance (log-scale)')
```

```
qplot(log(ensembleweightOTB$weights[,3]),
      log(plastweightOTB$weights[,3]), 
      main = names(Po)[3], 
      xlab = 'Noise-Penalized Variance (log-scale)', 
      ylab = 'Plasticity-Penalized Variance (log-scale)')
```

```
qplot(log(ensembleweightOTB$weights[,4]), 
      log(plastweightOTB$weights[,4]), 
      main = names(Po)[4], 
      xlab = 'Noise-Penalized Variance (log-scale)', 
      ylab = 'Plasticity-Penalized Variance (log-scale)')
```

```
qplot(log(ensembleweightOTB$weights[,5]), 
      log(plastweightOTB$weights[,5]), 
      main = names(Po)[5], 
      xlab = 'Noise-Penalized Variance (log-scale)', 
      ylab = 'Plasticity Weight (log-scale)')
```

Ok, and now I’m curius to compare the simulation-based variances to the observed variance in daily vales

```
temp <- merge(auxmat_tbvar, plastweightOTB$weights, by = 0)

qplot(log(temp$Active), 
      temp$Var_Active, 
      main = 'Active', 
      xlab = 'Observed Variance in Daily Time Budget (log-scale)', 
      ylab = 'Plasticity-Penalized Variance (log-scale)')
```

```
cor(temp$Active, exp(temp$Var_Active))
```

```
## [1] 0.9960189
```

```
qplot(log(temp$HighlyActive), 
      temp$Var_HighlyActive, 
      main = 'Highly Active', 
      xlab = 'Observed Variance in Daily Time Budget (log-scale)', 
      ylab = 'Plasticity-Penalized Variance (log-scale)')
```

```
cor(temp$HighlyActive, exp(temp$Var_HighlyActive))
```

```
## [1] 0.9953798
```

```
qplot(log(temp$Rumination), 
      temp$Var_Rumination, 
      main = 'Rumination', 
      xlab = 'Observed Variance in Daily Time Budget (log-scale)', 
      ylab = 'Plasticity-Penalized Variance (log-scale)')
```

```
cor(temp$Rumination, exp(temp$Var_Rumination))
```

```
## [1] 0.9899404
```

```
qplot(log(temp$Nonactive), 
      temp$Var_Nonactive, 
      main = 'Nonactive', 
      xlab = 'Observed Variance in Daily Time Budget (log-scale)', 
      ylab = 'Plasticity-Penalized Variance (log-scale)')
```

```
cor(temp$Nonactive, exp(temp$Var_Nonactive))
```

```
## [1] 0.9950559
```

```
qplot(log(temp$Eating), 
      temp$Var_Eating, 
      main = 'Eating', 
      xlab = 'Observed Variance in Daily Time Budget (log-scale)', 
      ylab = 'Plasticity-Penalized Variance (log-scale)')
```

```
cor(temp$Eating, exp(temp$Var_Eating))
```

```
## [1] 0.9912937
```

### Summary Plot

```
plotout <- ggpubr::ggarrange(
            ggplotify::as.ggplot(encodeout_otb_euc1$nrow_10_ncol_0$plot), 
            ggplotify::as.ggplot(encodeout_otb_kld1$nrow_10_ncol_0$plot), 
            ggplotify::as.ggplot(encodeout_otb_ew1$nrow_10_ncol_0$plot),
            ggplotify::as.ggplot(encodeout_otb_pw1$nrow_10_ncol_0$plot),
            ncol = 2, nrow = 2)

ggpubr::ggexport(plotout,
                         width = 30, 
                         height = 45, 
                         res = 300,
                         filename = 'Viz/OverallTB/OTBEncodings/EncodingSummary.pdf')
```

```
## file saved to Viz/OverallTB/OTBEncodings/EncodingSummary.pdf
```

## Comparisons of Clusterings

https://cran.r-project.org/web/packages/dendextend/vignettes/dendextend.html#comparing-two-dendrograms

https://en.wikipedia.org/wiki/Pointwise\_mutual\_information?fbclid=IwAR1EMGU9uBnlpclCWd\_HQ025J\_NvTjENlZvWmEzMv0Ds6d\_cFeihQ8vaOKc

Alright, now I want to make a ploting utility that will help me visually compare my encodings

Checking Inverse Beta estimates: https://keisan.casio.com/exec/system/1180573395

```
NBThetaCI <- function(y, k, N){
  # Calculate confidence intervale for theta of negative binomial data as in Johnson 1999
  a = k 
  b = N - k + 1
  theta_low  <- zipfR::Rbeta.inv(y,a,b, log = F)
  
  a = N - k
  b = k + 1
  theta_high  <- 1 - zipfR::Rbeta.inv(y,a,b, log = F)
  
  return(list(LowerBound = theta_low, UpperBound = theta_high))
}

#dendlist(as.dendrogram(htree_otb_euc), as.dendrogram(htree_otb_ew)) %>%
#  untangle(method = "step1side") %>% # Find the best alignment layout
#  tanglegram(k_branches = 8)  
 
# encodeout1 <- encodeout_otb_euc$nrow_5_ncol_0
# encodeout2 <- dmgridout_all$nrow_6_ncol_3

 
#encodeout2 <- encodeout_otb_ew$nrow_10_ncol_1
#encodeout2 <- dmgridout_all$nrow_8_ncol_6

encodeout1 <- data.frame(SickPen = cowattribdat$Sick_Prepasture)
rownames(encodeout1) <- cowattribdat$CowID

#encodeout1 <- encodeout_otb_euc$nrow_2_ncol_1
#encodeout2 <- encodeout_ageyeild_h$nrow_3_ncol_0

#encodeout1 <- dmgridout_all$nrow_4_ncol_3
#encodeout2 <- encodeout_otb_ew$nrow_5_ncol_0

compareEncodings <- function(encodeout1, encodeout2, 
                             #varnames = c('X','Y'),
                             colorby = 'count', #PMI
                             simPMI = NULL, 
                             #NullPerm, NullParam, CIParam
                             nsim = 1000,
                             alpha = 0.05,
                             logbase = 2,
                             gradient_color_low = 'white',
                             gradient_color_high = 'steelblue',
                             gridcolor = 'black',
                             nacolor = NA,
                             confmat_xlab = '',
                             confmat_ylab = '',
                             fontsize = 25,
                             imheight = 30,
                             imwidth = 30,
                             imres = 300,
                             filename = 'ClusterComparison', 
                             extraplot = NULL,
                             returnplots = FALSE,
                             verbose = FALSE
                             ){ 
  
  
  ### preprocess data frames ###
  
  if(is.data.frame(encodeout1)){
    
    temp <- list()
    temp$rowclust <- as.data.frame(encodeout1)
    names(temp$rowclust) <- 'X'
    temp$plot <- ggplot(na.omit(temp$rowclust), aes(X)) + 
               geom_bar() + 
               xlab('') + ylab('Count')
    encodeout1 <- temp
    
  } 
  
  if(is.data.frame(encodeout2)){
    
    temp <- list()
    temp$rowclust <- as.data.frame(encodeout2)
    names(temp$rowclust) <- 'Y'
    temp$plot <- ggplot(na.omit(temp$rowclust), aes(Y)) + 
               geom_bar() + 
               xlab('') + ylab('Count')
    encodeout2 <- temp
    
  } 
  
  ### calc confusion matrix ###
  
  rowclust1 <- as.data.frame(encodeout1$rowclust)
  names(rowclust1) <- 'X'
  rowclust2 <- as.data.frame(encodeout2$rowclust)
  names(rowclust2) <- 'Y'
  
  mergedat <- transform(merge(rowclust1, rowclust2, by = 0),
                            row.names=Row.names, Row.names=NULL)
  mergedat <- mergedat[complete.cases(mergedat), ]
  confmat <- table(mergedat[,1], mergedat[,2])
  
  pmat <- (confmat)/sum(confmat) # empirical ML frequencies
  dmmat <- (confmat + 1)/sum(confmat + 1) # convert to dierchlets mean to account for counts of zero
  
  
  ### Calculate PMI ###
  
  px <- apply(dmmat, 1, sum)
  py <- apply(dmmat, 2, sum)
  
  exptmat <- outer(px, py, FUN = '*') # null expected joint frequency
  
  PMI <- log(dmmat/exptmat, base = logbase)
  
  # PMI[is.infinite(PMI)] <- 0
  # PMI <- (confmat/sum(confmat)) - exptmat
  
#  # Calculate penalized PMI values
# 
#   penPMI <- matrix(NA, nrow = nrow(pmat), ncol = ncol(pmat))
#   #ibeta <- function(x,a,b){ pbeta(x,a,b)*beta(a,b) }
#   N <- sum(confmat)
#   n_row <- apply(confmat,1,sum)
#   n_col <- apply(confmat,2,sum)
#   s <- 1 - alpha
#   y <- (1-s)/2
#   
#   for(i in 1:nrow(pmat)){
#     for(j in 1:ncol(pmat)){
#       
#       mynumerator <- thetaCI(y, 
#                              confmat[i,j],
#                              n_row[i])$LowerBound
#       mydenom <- thetaCI(y, 
#                              n_col[j],
#                              N)$UpperBound
#       
#       penPMI[i,j] <- log(mynumerator/mydenom, base = logbase) 
#       penPMI[is.infinite(penPMI)] <- 0
#      
#     } 
#   }
#   
#   penPMI 
  
  
  
  ### Simulations  ###
  
  # obsexptmat <- outer(apply(confmat/sum(confmat), 1, sum), 
  #                     apply(confmat/sum(confmat), 2, sum), FUN = '*')
  

  if(is.null(simPMI)){
    PMI_thresh <- PMI
    qlower = NULL
    qupper = NULL
  }else{
    
    simout <- array(NA, dim= c(length(px),length(py),nsim))
    
    if(simPMI == 'NullParam'){
      
      for(i in 1:nsim){

        confmat_temp <- matrix(rmultinom(n = 1,
                                         size = sum(confmat),
                                         prob = exptmat),
                               #prob = obsexptmat),
                               nrow = length(px),
                               ncol = length(py))
        
        dmmat_temp <- (confmat_temp + 1)/sum(confmat_temp + 1)
        px_temp <- apply(dmmat_temp, 1, sum)
        py_temp <- apply(dmmat_temp, 2, sum)
        exptmat_temp <- outer(px_temp, py_temp, FUN = '*')
        simout[ , ,i] <- log(dmmat_temp/exptmat_temp, base = logbase)
        
      }
      
      qlower <- apply(simout, c(1,2),
                      function(x) quantile(x,alpha/2))
      qupper <- apply(simout, c(1,2),
                      function(x) quantile(x,1-alpha/2))
      
      PMI_thresh <- PMI *  !(PMI <= qupper & PMI >= qlower)
      
      
    }else if(simPMI == 'NullPerm'){  
      
      for(i in 1:nsim){
        
        tempdat <- mergedat
        tempdat[,1] <- sample(tempdat[,1]) # permute over row index to break any bivariate associations
        
        confmat_temp <- table(tempdat[,1], tempdat[,2])
        dmmat_temp <- (confmat_temp + 1)/sum(confmat_temp + 1)
        px_temp <- apply(dmmat_temp, 1, sum)
        py_temp <- apply(dmmat_temp, 2, sum)
        exptmat_temp <- outer(px_temp, py_temp, FUN = '*')
        simout[ , ,i] <- log(dmmat_temp/exptmat_temp, base = logbase)

      }
      
      qlower <- apply(simout, c(1,2),
                      function(x) quantile(x,alpha/2))
      qupper <- apply(simout, c(1,2),
                      function(x) quantile(x,1-alpha/2))
      
      PMI_thresh <- PMI *  !(PMI <= qupper & PMI >= qlower)
      
      
    }else if(simPMI == 'CIParam'){
      
      for(i in 1:nsim){
        confmat_temp <- matrix(rmultinom(n = 1,
                                         size = sum(confmat),
                                         prob = dmmat),
                               #prob = obsexptmat),
                               nrow = length(px),
                               ncol = length(py))
        
        dmmat_temp <- (confmat_temp + 1)/sum(confmat_temp + 1)
        px_temp <- apply(dmmat_temp, 1, sum)
        py_temp <- apply(dmmat_temp, 2, sum)
        exptmat_temp <- outer(px_temp, py_temp, FUN = '*')
        simout[ , ,i] <- log(dmmat_temp/exptmat_temp, base = logbase)
        
      }

      qlower <- apply(simout, c(1,2),
                      function(x) quantile(x,alpha/2))
      qupper <- apply(simout, c(1,2),
                      function(x) quantile(x,1-alpha/2))
      
      PMI_thresh <- PMI *  !(qlower <= 0  & qupper >= 0)
      PMI_thresh[PMI_thresh != 0 & qlower > 0] <- qlower[PMI_thresh != 0 & qlower > 0]
      PMI_thresh[PMI_thresh != 0 & qupper < 0] <- qupper[PMI_thresh != 0 & qupper < 0]
      
      
      
    }else{
      stop('Error: PMI simulation method not recognized')
    }
    
  }
  
  
  
  
  # Setup Matrix Viz
  
  temp1 <- reshape2::melt(confmat)
  rownames(temp1) <- paste(temp1$Var1, temp1$Var2, sep = '_')
  names(temp1)[which(names(temp1) == 'value')] <- 'Count'
  
  temp2 <- reshape2::melt(PMI_thresh)
  rownames(temp2) <- paste(temp2$Var1, temp2$Var2, sep = '_')
  names(temp2)[which(names(temp2) == 'value')] <- 'PMI'
  
  temp <- merge(temp1, temp2, by = 0)
  names(temp)[which(names(temp) == 'Var1.x')] <- 'Var1'
  names(temp)[which(names(temp) == 'Var2.x')] <- 'Var2'
  
  if(is.integer(temp$Var1)){
    temp$Var1 <- factor(temp$Var1, 
                        levels = sort(unique(temp$Var1)),
                        ordered = T)
  }
  
  if(is.integer(temp$Var2)){
    temp$Var2 <- factor(temp$Var2, 
                        levels = sort(unique(temp$Var2)),
                        ordered = T)
  }
  
  
  
  
  if(colorby == 'count'){
    
    matplot <- ggplot(temp, aes(x = Var1, y = Var2, fill = Count)) +
    geom_tile(colour = gridcolor) + 
    #geom_text(aes(label = sprintf("%1.2f", Count)), vjust = 1) +
    geom_text(aes(label = Count), size = fontsize) + 
    scale_fill_gradient(low = gradient_color_low, 
                        high = gradient_color_high,
                        na.value = nacolor) +
    #xlim(mylevelsx) +
    ylim(rev(levels(temp$Var2))) +
    #scale_y_reverse() +
    xlab(confmat_xlab) + ylab(confmat_ylab) + theme_bw() +
    theme(panel.border = element_blank(), 
          panel.grid.major = element_blank(),
          panel.grid.minor = element_blank(),
          axis.ticks.x = element_blank(),
          #axis.text.x = element_blank(),
          axis.ticks.y = element_blank(),
          #axis.text.y = element_blank(),
          legend.position = "none",
          axis.text = element_text(size = fontsize))
    
  }else if(colorby == 'PMI'){
    
    matplot <- ggplot(temp, aes(x = Var1, y = Var2, fill = PMI)) +
    geom_tile(colour = gridcolor) + 
    #geom_text(aes(label = sprintf("%1.2f", Count)), vjust = 1) +
    geom_text(aes(label = Count), size = fontsize) + 
    scale_fill_gradient2(low = gradient_color_low, 
                        mid = 'white',
                        high = gradient_color_high,
                        na.value = nacolor,
                        midpoint = 0) +
    ylim(rev(levels(temp$Var2))) +
    #scale_y_reverse() +
    xlab(confmat_xlab) + ylab(confmat_ylab) +  theme_bw() +
    theme(panel.border = element_blank(), 
          panel.grid.major = element_blank(),
          panel.grid.minor = element_blank(),
          axis.ticks.x = element_blank(),
          #axis.text.x = element_blank(),
          axis.ticks.y = element_blank(),
          #axis.text.y = element_blank(),
          #legend.position = "none",
          axis.text = element_text(size = fontsize))
  }else{
    stop('Error: Option for colorby not recognized')
  }

  
  # Create joint viz

    
    if(sum(class(encodeout1$plot) == 'list') == 1){
      grid1 = 1:length(encodeout1$plot)
    }else{
      grid1 = 1
    }
    
    if(sum(class(encodeout2$plot) == 'list') == 1){
      grid2 = 1:length(encodeout2$plot)
    }else{
      grid2 = 1
    }
    
    for(i in grid1){
      for(j in grid2){
        
        if(sum(class(encodeout1$plot) == 'list') == 1){
           xheat <- ggplotify::as.ggplot(encodeout1$plot[[i]]) + 
             theme(legend.position = "none")
           xname <- names(encodeout1$plot)[i]
        }else{
           xheat <- ggplotify::as.ggplot(encodeout1$plot) + 
             theme(legend.position = "none")
           xname <-''
        }
        
        if(sum(class(encodeout2$plot) == 'list') == 1){
          yheat <- ggplotify::as.ggplot(encodeout2$plot[[j]]) + 
            theme(legend.position = "none")
          yname <- names(encodeout2$plot)[j]
        }else{
          yheat <- ggplotify::as.ggplot(encodeout2$plot) + 
            theme(legend.position = "none")
          yname <- ''
        }
        
        if(is.null(extraplot)){
          plotout <- ggpubr::ggarrange(NULL, 
                                       ggplotify::as.ggplot(xheat), 
                                       ggplotify::as.ggplot(yheat),
                                       matplot, 
                                       ncol = 2, nrow = 2) 
        }else{
          plotout <- ggpubr::ggarrange(
            ggplotify::as.ggplot(extraplot), 
            ggplotify::as.ggplot(xheat), 
            ggplotify::as.ggplot(yheat),
            matplot, 
            ncol = 2, nrow = 2) 
        }
        
        # don't export pdf if filename is set to NA or NULl
        if( !(is.na(filename) | is.null(filename)) ){
           ggpubr::ggexport(plotout,
                         width = imwidth, 
                         height = imheight, 
                         res = imres,
                         filename = paste(filename,
                                          xname,
                                          yname, 
                                          '.pdf', sep = ''))
        }
        
      }
    }
    
  if(returnplots){
    rplotlist <- list(
      fullplot = plotout, 
      encoding1plot = ggplotify::as.ggplot(xheat),
      encoding2plot = ggplotify::as.ggplot(yheat),
      confusionmatrix = matplot
    )
  }else{
    rplotlist <- NULL
  }
    
  if(verbose){
    return(list(PlotList = rplotlis,
                counts = confmat,
                obsfreq = pmat,
                dirchletmeans = dmmat,
                nullfreq = exptmat,
                PMI = PMI,
                PMIThresholded = PMI_thresh,
                simquantile_lowerbound = qlower,
                simquantile_upperbound = qupper
                ))
  }
  
   # xheat <- ggplotify::as.ggplot(encodeout1$plot) + 
   #   theme(legend.position = "none")
   # yheat <- ggplotify::as.ggplot(encodeout2$plot) + 
   #   theme(legend.position = "none")
   # 
  
   # plotout <- ggpubr::ggarrange(NULL, 
   #                              ggplotify::as.ggplot(xheat), 
   #                              ggplotify::as.ggplot(yheat), matplot, 
   #                              ncol = 2, nrow = 2) #,  align = "hv", widths = c(2, 1), heights = c(1, 2))
   # 
   # ggpubr::ggexport(plotout,
   #                  width = imwidth, height = imheight, res = imres,
   #                  filename = paste(filename, '.pdf', sep = ''))
   
}
```

### Euclidean vs KLD

```
compareEncodings(encodeout_otb_euc$nrow_5_ncol_0, 
                 encodeout_otb_kld$nrow_5_ncol_0, 
                             # n_rowcluster1 = 6, 
                             # n_rowcluster2 = 6,
                             # n_colcluster1 = 1,
                             # n_colcluster1 = 1,
                             filename = 'Viz/OverallTB/ContrastPlots/Euclid_vs_KLD_5x5',
                             imheight = 45,
                             imwidth = 30,
                             fontsize = 35
                             )
```

```
## file saved to Viz/OverallTB/ContrastPlots/Euclid_vs_KLD_5x5.pdf
```

The two most extreme clusters of high/low eating cows are the same, but there is a lot of wiggle in the cutoffs for the remainder of the herd with more moderate time budgets.

```
compareEncodings(encodeout_otb_euc$nrow_10_ncol_0, 
                 encodeout_otb_kld$nrow_10_ncol_0, 
                             # n_rowcluster1 = 6, 
                             # n_rowcluster2 = 6,
                             # n_colcluster1 = 1,
                             # n_colcluster1 = 1,
                             filename = 'Viz/OverallTB/ContrastPlots/Euclid_vs_KLD_10x10',
                             imheight = 45,
                             imwidth = 30,
                             fontsize = 25
                             )
```

```
## file saved to Viz/OverallTB/ContrastPlots/Euclid_vs_KLD_10x10.pdf
```

These clusterings are pretty close with respect to cows with extreme tradeoffs in eating vs rumination time. KLD did break down the high rumination/low eating cows smaller clusters. The cutoffs between cows with moderate time budgets are not very consistent between the two methods, however.

### Euclidean vs Ensemble-Weighted

```
compareEncodings(encodeout_otb_euc$nrow_5_ncol_0, 
                 encodeout_otb_ew$nrow_5_ncol_0, 
                             # n_rowcluster1 = 6, 
                             # n_rowcluster2 = 6,
                             # n_colcluster1 = 1,
                             # n_colcluster1 = 1,
                             filename = 'Viz/OverallTB/ContrastPlots/Euclid_vs_EW_5x5',
                             imheight = 45,
                             imwidth = 30,
                             fontsize = 35
                             )
```

```
## file saved to Viz/OverallTB/ContrastPlots/Euclid_vs_EW_5x5.pdf
```

The euclidean distance embedding is clearly placing more emphasis on extremes in the high magnitude eating and rumination, while the ensemble reweighting has placed more emphasis on heterogenity in nonactive time.

```
compareEncodings(encodeout_otb_euc$nrow_10_ncol_0, 
                 encodeout_otb_ew$nrow_10_ncol_0, 
                             # n_rowcluster1 = 6, 
                             # n_rowcluster2 = 6,
                             # n_colcluster1 = 1,
                             # n_colcluster1 = 1,
                             filename = 'Viz/OverallTB/ContrastPlots/Euclid_vs_EW_10x10',
                             imheight = 45,
                             imwidth = 30,
                             fontsize = 25
                             )
```

```
## file saved to Viz/OverallTB/ContrastPlots/Euclid_vs_EW_10x10.pdf
```

The ensemble reweighted distances seem to have produced a more conservative encoding of our more extreme cows, which seem to be over-cut in the regular euclidean encoding.

The cutoffs between more moderate cows are definitely quite different between the two encodings, with the activity axes not really coming into play in the unweighted encoding

### Euclidean vs Plasticity-Weighted

```
compareEncodings(encodeout_otb_euc$nrow_5_ncol_0, 
                 encodeout_otb_pw$nrow_5_ncol_0, 
                             # n_rowcluster1 = 6, 
                             # n_rowcluster2 = 6,
                             # n_colcluster1 = 1,
                             # n_colcluster1 = 1,
                             filename = 'Viz/OverallTB/ContrastPlots/Euclid_vs_PW_5x5',
                             imheight = 45,
                             imwidth = 30,
                             fontsize = 35
                             )
```

```
## file saved to Viz/OverallTB/ContrastPlots/Euclid_vs_PW_5x5.pdf
```

```
compareEncodings(encodeout_otb_euc$nrow_10_ncol_0, 
                 encodeout_otb_pw$nrow_10_ncol_0, 
                             # n_rowcluster1 = 6, 
                             # n_rowcluster2 = 6,
                             # n_colcluster1 = 1,
                             # n_colcluster1 = 1,
                             filename = 'Viz/OverallTB/ContrastPlots/Euclid_vs_PW_10x10',
                             imheight = 45,
                             imwidth = 30,
                             fontsize = 25
                             )
```

```
## file saved to Viz/OverallTB/ContrastPlots/Euclid_vs_PW_10x10.pdf
```

### KLD vs Ensemble

```
compareEncodings(encodeout_otb_kld$nrow_5_ncol_0, 
                 encodeout_otb_ew$nrow_5_ncol_0, 
                             # n_rowcluster1 = 6, 
                             # n_rowcluster2 = 6,
                             # n_colcluster1 = 1,
                             # n_colcluster1 = 1,
                             filename = 'Viz/OverallTB/ContrastPlots/KLD_vs_EW_5x5',
                             imheight = 45,
                             imwidth = 30,
                             fontsize = 35
                             )
```

```
## file saved to Viz/OverallTB/ContrastPlots/KLD_vs_EW_5x5.pdf
```

Here we can see that the KLD is really going for characterizing the most extreme time budgets first, namely the extremes in eating and rumination times, and has completely ignored the moderate cows. The ensemble encoding is also going after the extremes first, but may be doing so a bit more equitably across behavioral axes, as it seems to be giving the activity axes a bit more consideration.

```
compareEncodings(encodeout_otb_kld$nrow_10_ncol_0, 
                 encodeout_otb_ew$nrow_10_ncol_0, 
                             # n_rowcluster1 = 6, 
                             # n_rowcluster2 = 6,
                             # n_colcluster1 = 1,
                             # n_colcluster1 = 1,
                             filename = 'Viz/OverallTB/ContrastPlots/KLD_vs_EW_10x10',
                             imheight = 45,
                             imwidth = 30,
                             fontsize = 25
                             )
```

```
## file saved to Viz/OverallTB/ContrastPlots/KLD_vs_EW_10x10.pdf
```

A surprising amoung of difference really, for these two distance values to both be taking into account heterogeneity in scaling. The ensemble encoding was definitely far more conservative in determining divisions amongst cows time budgets on the extreme ends of the spectrum. The cutoffs between moderate cows also diverged quite a bit, with the ensemble encoding to be somewhat smoother, particularly for clusters along the nonactive axis.

### KLD vs Plasticity

```
compareEncodings(encodeout_otb_kld$nrow_5_ncol_0, 
                 encodeout_otb_pw$nrow_5_ncol_0, 
                             # n_rowcluster1 = 6, 
                             # n_rowcluster2 = 6,
                             # n_colcluster1 = 1,
                             # n_colcluster1 = 1,
                             filename = 'Viz/OverallTB/ContrastPlots/KLD_vs_PW_5x5',
                             imheight = 45,
                             imwidth = 30,
                             fontsize = 35
                             )
```

```
## file saved to Viz/OverallTB/ContrastPlots/KLD_vs_PW_5x5.pdf
```

```
compareEncodings(encodeout_otb_kld$nrow_10_ncol_0, 
                 encodeout_otb_pw$nrow_10_ncol_0, 
                             # n_rowcluster1 = 6, 
                             # n_rowcluster2 = 6,
                             # n_colcluster1 = 1,
                             # n_colcluster1 = 1,
                             imheight = 45,
                             imwidth = 30,
                             fontsize = 25,
                             filename = 'Viz/OverallTB/ContrastPlots/KLD_vs_PW_10x10'
                             )
```

```
## file saved to Viz/OverallTB/ContrastPlots/KLD_vs_PW_10x10.pdf
```

### Ensemble vs Plasticity

```
compareEncodings(encodeout_otb_pw$nrow_5_ncol_0, 
                 encodeout_otb_ew$nrow_5_ncol_0, 
                             # n_rowcluster1 = 6, 
                             # n_rowcluster2 = 6,
                             # n_colcluster1 = 1,
                             # n_colcluster1 = 1,
                             filename = 'Viz/OverallTB/ContrastPlots/EW_vs_PW_5x5',
                             imheight = 45,
                             imwidth = 30,
                             fontsize = 35
                             )
```

```
## file saved to Viz/OverallTB/ContrastPlots/EW_vs_PW_5x5.pdf
```

The low eating group is the same excluding one cow. The plasticity-weighted ensemble identifyied and isolated the low-eating group much earlier.

Among the moderate time budgets, the plasticity weighted ensemble is more heterogenous, but the three groups are more balanced in size.

```
compareEncodings(encodeout_otb_pw$nrow_10_ncol_0, 
                 encodeout_otb_ew$nrow_10_ncol_0, 
                             # n_rowcluster1 = 6, 
                             # n_rowcluster2 = 6,
                             # n_colcluster1 = 1,
                             # n_colcluster1 = 1,
                             imheight = 45,
                             imwidth = 30,
                             fontsize = 25,
                             filename = 'Viz/OverallTB/ContrastPlots/EW_vs_PW_10x10'
                             )
```

```
## file saved to Viz/OverallTB/ContrastPlots/EW_vs_PW_10x10.pdf
```

Interestingly, the plasticity-weighted encoding has appeared to put more emphasis on the high activity axis.

```
save.image("OverallTB.RData")
```

# Analysis of OTB - Ensemble Cut

Alright, we’ve come up with a few stratedgies to quantittively compare time budgets, and we can subsequently use these dissimilarity measures to represent the multi-dimensional structure in a 2D dendrogram. But how do we know which branches represent authentic systematic differences between cows in their behavioral investments and where we are just starting to get into noise? So now lets explore how we can leverage the multinomal distribution of this data to also help us determine the cluster cutoffs.

When cutting a tree to generate clusters the ultimate goal is to pick the point where where signal ends and we get into the stochastic weeds. When we do this by visual inspection, we look for a sudden drop off in branch length. There are two main problems with this approach. The first is that it is ultimately subjective, especially where the shift from signal to noise is not clear, which can create issues with reproducibility. The second is that, if the variance is non-uniform across the domain of support of the function, then raw branch lengths will not be comparible across the entire data set. This can happen with data sets that come from distributions that are intrinsically heterogenous, such as the multinomial. We could also expect this dynamic if there are multiple components of signal that may operate at different magnitudes within the system (expl - herd level vs clique level effects are both components of signal, but may have effects on observed behavioral values of different magnitudes), then cutting a tree at a fixed dissimilarity value may overlook signal present in some subpopulations.

My goal here is to create an algorithm that systematically moves through a tree branch by branch, cutting only where it senses there is a clear shift from signal to noise. It will do this by assessing the reliability of each proposed bifurcation in the data using two ensemble mimicries.

The first ensemble will be a simple mimickry of the observed data created by redrawing from a multinomial distribution using the observed proportion of time dedicated to each behavior in a given hour observation sample, which will contain the entirity of the observed signal. This is the same mimickry used to compute ensemble distances, so we will be able to speed up the code significantly if we repurpose these pre-computed simulation results.

I will then create a second ensemble mimickry, which I will call the “null mimickry”, where for the given branch under consideration, I will assume the bifurcation is simply just picking up noise and that all all behavioral signals are equivalent relative the resolution of the metric. If the null is true, then animals will not be any more likely to be bifurcated together in a simulation under the null than one generated with the observed hourly time budgets. I can contrast the reliability of this binarization across the two simulations using MI.

I will first measure MI of branch binarization on the observed data against the mimickry by hour, which will reflect my confidence under the alternative that the branch is picking up signal. I will then measure MI of the branch bifurcation of the observed data against the mimickry under the null. I will then assess the distribution of these two sets of entropy values. If they overlap significantly, then I will assume that branch is noise and not use it to create distinct clusters. If the two mimickries produce distinct MI values against the observed data, I will determine this branch contains signal, and use it to cut the data.

## Creating Functions

First lets start with a simple helper function to pull a discrete code off a data set using whatever clustering method is desired

```
getBinaryCode <- function(x, 
                          nclust = 2,
                          dist_method = 'Euclidean', 
                          hclust_method = 'ward.D2', 
                          simdata = NULL, 
                          weightdist = NULL,
                          returnTree = FALSE){
  
  ## Claculate Dissimilarity Matrix ##
  
  if(dist_method == 'Euclidean'){
    distmat <- dist(x)
  }
  if(dist_method == 'KLD'){
    distmat <- KLDdist(x, runfast = T)
    #distmat <- as.dist(Rfast::Dist(x, method='kullback_leibler'))
  }
  if(dist_method == 'Ensemble'){
    distmat <- ensembledist(x, simdata, weightdist = weightdist)
    wout <- ensembledist(x, simdata, weightdist = weightdist)
    #distmat2 <- j
  }
  
  
  ## Create Binary Code ##
  
  hout <- hclust(distmat, method = hclust_method)
  cout <- cutree(hout, nclust)
  
  if(returnTree){
    return(list(cluster = cout, hctree = hout , weightdist))
  }else{
    return(cout)
  }
  
}

start_time <- Sys.time() 
temp <- getBinaryCode(Po)
end_time <- Sys.time()
end_time - start_time
```

```
## Time difference of 0.002511024 secs
```

```
start_time <- Sys.time() 
temp <- getBinaryCode(Po, dist_method = 'KLD')
end_time <- Sys.time()
end_time - start_time
```

```
## Time difference of 0.016258 secs
```

```
start_time <- Sys.time() 
temp <- getBinaryCode(Po, dist_method = 'Ensemble', simdata =  simotb$TBSim)
end_time <- Sys.time()
end_time - start_time
```

```
## Time difference of 0.1715209 secs
```

```
start_time <- Sys.time() 
temp <- getBinaryCode(Po, dist_method = 'Ensemble', 
                      simdata = simotb$TBSim, 
                      weightdist = ensembleweightOTB$weights)
end_time <- Sys.time()
end_time - start_time
```

```
## Time difference of 0.03628612 secs
```

Righto, so all these cases appear to be running correctly. We do see however how much of a speedup we can get by initializing the ensemble weights instead of recaculating from the simulation each time.

And now I need a function to test the statistical significance of a given branch.

NOTE: We can significantly speed up the code if we pre-compute the simulation of the raw data used to estiamte the alternative MI hist. For larger data sets, however, there may be memory constraints on the number of simulations that can be stored. In the higher branches, there is typically enough of a gap between branches that an extremely large simulation would not be needed to resolve the difference between the alternative from the null MI distributions. For smaller branches branches farther down the tree that are approaching the stochastic weeds with lower magnitude biological signals, however, additional simulations likely will be needed to resolve these distributions, which shouldn’t be an undue burden on the computer memeory for smaller subsets of cows. To determine which branches will need additional simulations, we’ll start out testing with the presimulated data, and then estimate confidence intervals for the quantiles used to estimate the cutoffs. If the quantiles overlap, then we’ll allow the function to loop back through and add more simulations by calling the test function recursively on itself.

For the cutoff decision we’ll see if the 95-th quantile of the simulation of MI under the null is lower than the 5th quantile of simulated MI values under the alternative. I’ll estiamte the two-sided 95% confidence intervals for these quantile values using the approximate interval method using EnvStats.

https://staff.math.su.se/hoehle/blog/2016/10/23/quantileCI.html

I am going to add a maxiter option, which will allow me to set the maximum size for a simulation under the alternative. If we double the size of the simulation at each recursion, and we still can return a confident result by the time we reach our simulation threshold, then the function will just return that the brach cut under consideration is insignificant.

OLD NOTE: Mimicking the data under the alternative can and should be done at the level of the raw data, because to do so only requires us to assume the that each individual observation point is distributed dirchlet/multinomial, which is pretty self-apparent. With the null distribution, however, we are also assuming that cows have equivalent time budgets that can be represented by a mean value. That assumption can only really be asserted at the level of aggregation that the data is being clustered, not at the level of the raw data. So for example, it may be reasonable in clustering overall time budgets to assume cows have the same underlying time budget, but its not reasonable to assume that cows in the same OTB cluster have the same distribute of time budgets at the level of day or hour. So we’ll simulate the null using the obs data, which has already been aggregated, so that our null simulation matches the level of aggregation used to formulate the hypothesized group of equivalent cows. We’ll do this by redrawing from a multivariatbeta (MVB) distribution, fit to the complete complement of cows using MOM estimators as in Narayanan (1992). This should give us not only the center of the distribution but also our relative confidence in sampled vectors. If there is structure in a data set, the variance within branch should be estimated to be fairly high under the null, which should make this test reasonably conservative.

Narayanan A. A note on parameter estimation in the multivariate beta distribution. Comput Math with Appl. 1992;24(10):11–7.

https://rdrr.io/github/feralaes/dampack/man/dirichlet\_params.html

Note: After further testing, I’ve decided to remove the MVB sampling stratedgy for the null model. First, this parametric stratedgy is not generalizable to other data formats. Second, because the MOM estimators only consider the first and second moments of the distribution, the null simulations I’m getting have far less kurtosis than the raw data - in other words the MVB is generating data null data sets that are more centrally peaked and so are getting bifucated near the center too often, which may cause MI to be under-estimated. Instead, I’m switching to a bootstrapping stratedgy, wherein I create a mimicry under the null by subsampling from the alternative mimicry without conditioning on cow ID. This represents our null in that we assume all cows have the equivalent behavioral patterns up to the resolution of the sample, and so under this assumption the simulation under the alterative should be the same for each cow, such that they collectively provide a good empirical estimation of the distribution of observational uncertainty as simulated using the same sampling error models.

Note: It is possible, when testing a given branch in the observed data, that there can be another split very close in strength so that, when a bit of noise is added to the data via simulation, you get that the binary cut will swap back and forth between those two competing mechnism. This competition between splits will obscure (distract) the simple binary encoding, which could make a branch that represents an authentic signal that is in competition with another split look locally like a branch has hit the stochasitic weeds. To account for this, if a given branch is insignificant with a binary encoding, or the result is ambigous within confidence bounts, then I’ll enable the user to perform a test for significance allowing for multiple clusters. This will allow me, if a given split is insignificant, to “look down the branch” to check that there isn’t any significant structure beneath it. The nclustalt will allow the user to specify how far down the branch they want to look. I’ll never look down the branch if there are not at least twice the number of obs left in a branch as the number of encoding levels. If a user doesn’t want to check down the branch, then they can leave that option as the default null, which will allow the algorithm to only perform a binary test of the current branch under consideration, which should provide a be conservative result.

```
# to-do notes:
# axis I pull feature subset from needs to be adaptive (currently set to second axis for OTB where time axis is collapsed)
# need to fix the mimicky endoding under the null for tb tensor


#pmat <- Po
paramMVB <- function(pmat){
  # pmat where i is obs, and each row contains p-vector summing to 1
  # returns estimates for alpha-paramters for dirchlet (MVBeta)
  
  p1 <- apply(pmat, 2, function(x) mean(x, na.rm = T)) # first moment
  #p2 <- apply(Po, 2, function(x) mean(x, na.rm = T)) # second moment
  p2 <- apply(pmat, 2, function(x) sd(x, na.rm = T))
  p2 <- p1^2 + p2^2
  
  n <- length(p1)
  
  myalpha <- c()
  for(i in 1:(n-1)){
    myalpha[i] <- (p1[1] - p2[1]) * p1[i]/(p2[1] - 
            p1[1]^2)
  } 
  
  myalpha[n] <- ( (p1[1]-p2[1])*(1-sum(p1[-n])) ) / 
                      (p2[1] - p1[1]^2)
  
  return(myalpha)
                                    
} 
#paramMVB(Po)
# p1 <- apply(Po, 2, function(x) mean(x, na.rm = T))
# p2 <- apply(Po, 2, function(x) sd(x, na.rm = T))
# dampack::dirichlet_params(p1,p2)


testBranchTwo <- function(obsdat, simdata){
  
  if(length(dim(obsdat)) == 2){
    obsdiff <- sum((obsdat[1,] - obsdat[2,])^2)
    
    estvar2 <- apply(simdata, c(1,2), function(x) var(x^2, na.rm = T))
    estvar <- apply(simdata, c(1,2), function(x) var(x, na.rm = T))
    mysd <- sum(estvar2[1,] + 
                  estvar2[2,]^2 - 
                  2*estvar[1,]*estvar[2,])^0.5
    myci <- c(obsdiff - 3*mysd, obsdiff + 3*mysd)
    
    if(myci[1] <= 0 & myci[2] >= 0){ # if 0 in conf interval, fail branch test
      return(F)
    }else{
      return(T)
    }
  }else{
    # need to write code for temporal dim
  }
  
}

# temp <-  c("5","1454","1511","2129","2457","4965","10985","11298", "13429","13460","13470","13826","13946","13952","14256", "18175", "19185","23049","23314","23502","26068","26451","26934","27091", "27221","32607","33199","33513","45551","46769","46926","48576", "53683","55389","55454","55887","64584","65469","96023","97974", "98038", "98737","98771", "98777")

#temp <- c("677",  "4562", "13831", "23325")

#temp <- c("13485", "20531")

#temp <- c( "5" , "1038" , "1090" , "1424" , "1545" , "4407", "4965", "9363" , "13952" ,"14256", "18787" ,"25816", "31867", "32949","46769" ,"47122" ,"53683", "55454", "64584" ,"96539", "98739" )

#temp <- c('2219', '55482', '23461', '55516', '97444', '49670', '55391', '645', '31904', '6580', '97907', '757', '22179', '1421', '55785', '45757', '1135', '13482')

# obsdat <- Po
# obsdat <- Po[temp, ]
# obsdat <- Po_day
# simdata <- simotb$TBSim
# simdata <- simotb$TBSim[temp,,]
# rawdata <- sensordat[temp, , ]
# rawdata <- Co

testBranch <- function(obsdat, simdata, rawdata, feature_subset = NULL, hclust_method = 'ward.D2', nclustalt = NULL, dist_method = 'Euclidean', weightdist = NULL, SimSpecs = NULL, logbase = 2, showplot = TRUE, verbose = F, B = 2000, maxiter = 2000){ 
  
  if(!is.null(feature_subset)){
    obsdat <- obsdat[ , feature_subset] # need to make dynamic
    simdata <- simdata[,feature_subset,]
    rawdata <- rawdata[ , , feature_subset]
    if(!is.null((weightdist))){
      weightdist <- weightdist[ , feature_subset]
    }
  }
  
  if(!is.null(nclustalt)){
    if(nrow(obsdat) < 2*nclustalt){nclustalt <- NULL}
    # if the user wants to look down the branch if the binary encoding is insignficant, but if there aren't at least twice as many obs as the number of encoding levels, then switch nclustalt to NULL so that we don't look down the branch with too few observations
  }
  

  
  ### get code for obs data ###
  
  encodeobs <- getBinaryCode(obsdat,
                             nclust = 2,
                             simdata = simdata,
                             hclust_method = hclust_method,
                             dist_method = dist_method,
                             weightdist = weightdist,
                             returnTree = T)
  
  # pheatmap(obsdat, cluster_rows = encodeobs$hctree) # check
  
  
  obscode <- encodeobs$cluster
  
  
  ###  Test for branch of n = 2 ###
  
  if(dim(obsdat)[1] == 2){ # if testing branch with only two obs
    return(list(sigdiff = testBranchTwo(obsdat, simdata),
                encoding = obscode))
  }
  
  ### get code for mimickry under alternative ###
  
  mimickcode_alt <- apply(simdata, 
                          length(dim(simdata)), 
                          function(x) 
                            getBinaryCode(x, 
                                      simdata = simdata,
                                      nclust = 2,
                                      hclust_method = hclust_method,
                                      dist_method = dist_method,
                                      #returnTree = T,
                                      weightdist = weightdist
                                      ))
  
  # check dist max distance of alt trees matches obs tree
  # temp <- apply(simdata,  
  #       length(dim(simdata)), 
  #       function(x) 
  #         max(getBinaryCode(x, 
  #                       simdata = simdata,
  #                       nclust = 2,
  #                       hclust_method = hclust_method,
  #                       dist_method = dist_method,
  #                       returnTree = T,
  #                       weightdist = weightdist
  #         )$hctree$height )
  #       ) 
  # hist(temp)
  # check alt clust
  # temp <- getBinaryCode(as.matrix(simdata[,,5]), 
  #                       simdata = simdata,
  #                       nclust = 2,
  #                       hclust_method = hclust_method,
  #                       dist_method = dist_method,
  #                       returnTree = T,
  #                       weightdist = weightdist)
  # plot(temp$hctree)
  # pheatmap(simdata[,,1], cluster_rows =  temp$hctree)
  
  
  ### make mimickry under null ###
  
    temp <- dimnames(obsdat)
    temp[[length(temp) + 1]] <- 1:B
    nullsim <- array(NA,
                     dim = c(dim(obsdat),B),
                     dimnames = temp)
  
  if(length(dim(obsdat))==2){ # simulate from overall time budget
    
    
    # fitdat <- rbind(as.matrix(obsdat), 
    #                 matrix(aperm(simdata, c(3,1,2)), 
    #                     ncol = dim(obsdat)[length(dim(obsdat))]))
    # myalpha <- paramMVB(fitdat) # MVB is fit to all observed and simulated otb vectors for this branch
    sampdat <- matrix(aperm(simdata, c(3,1,2)), 
                      ncol = dim(obsdat)[length(dim(obsdat))])
    for(b in 1:B){
      #nullsim[,,b] <- DirichletReg::rdirichlet(nrow(obsdat),
      #                                 alpha = myalpha)
      nullsim[,,b] <- sampdat[sample(1:nrow(sampdat),
                                     dim(obsdat)[1]), ]
      # sample randomly from the full collection of simulated overall time vectors from any animal in the ensemble data set 
      
      
      
      
    }

  }else{ # simulate from an obs data tensor with a time dimension
    
    # need to fix to also accept ensemble values
    # for(i in 1:dim(obsdat)[2]){
    #   myalpha <- paramMVB(obsdat[,i,])
    #   for(b in 1:B){
    #     nullsim[,i,,b] <- DirichletReg::rdirichlet(nrow(obsdat),
    #                                                alpha = myalpha)
    #   }
    # }
    
  }
 
  
  ### get code for null mimickry  ###

  if(length(dim(nullsim)) == 3){ # for analysis of overall TB matrix
    mimickcode_null <- apply(nullsim, 
                          length(dim(nullsim)), 
                          function(x) 
                          getBinaryCode(x, 
                                    nclust = 2,
                                    hclust_method = hclust_method,
                                    dist_method = dist_method,
                                    simdata = nullsim
                                    #weightdist = weightdist
                                    ) 
                             )
  }else{ # for analysis of tensor
    
  }
  
  
  
  ### get MI values ###
  
  mce_alt <- apply(mimickcode_alt, 2, function(x) 
    calcEntropy(table(as.factor(obscode), as.factor(x)), 
                   logbase = logbase)$MutualInformation)
  
  mce_null <- apply(mimickcode_null, 2, function(x) 
    calcEntropy(table(as.factor(obscode), as.factor(x)), 
                   logbase = logbase)$MutualInformation)
  
  nullquant <- EnvStats::eqnpar(mce_null, 0.95, ci = T, 
                                lb = 0, ub = 1)
  nullthreshold <- nullquant$quantiles
  nullci <- nullquant$interval$limits
  
  if(length(unique(mce_alt)) == 1){ # if all sims have same cut
    altquant <- NULL 
    altthreshold <- unique(mce_alt)
    altci <- c(unique(mce_alt), unique(mce_alt))
  }else{
    altquant <- EnvStats::eqnpar(mce_alt, 0.05, ci = T, 
                               lb = 0, ub = 1)
    altthreshold <- altquant$quantiles
    altci <- altquant$interval$limits
  }
  
  
  conftestfail <- sum(nullci >= altci[1] & nullci <= altci[2]) > 0 # check if quntile intervals overlap

  
  if(!conftestfail  & 
        (nullthreshold < altthreshold |
         nullthreshold >= altthreshold & is.null(nclustalt))){
    # if you've got a confident test result then function should return results if either 1) the branch is sig for the binary case or 2) its not significant for the binary case but user doesn't want to look forward with multilevel encoding
    
    # plot test distributions
    plotdat = data.frame(MCE = c(mce_alt, mce_null),
                         Mimickry = c(rep('Alt',
                                          length(mce_alt)),
                                      rep('Null', length(mce_null)))
                          )
    plotout <- ggplot(plotdat, aes(x = MCE, fill = Mimickry)) +
      geom_density(aes(y = ..scaled..), alpha = 0.75) + 
      xlim(c(-0.1,1.1)) + 
      ggtitle(paste('Result of Bifucation Simulation: N',
                    nrow(obsdat))) + 
      ylab('Scaled Density') + xlab('Mutual Information') +
      scale_fill_manual(breaks = c("Alt", "Null"), 
                       values=c("#00BFC4", "#F8766D"))
    if(showplot){  
      print(plotout)
      # ggpubr::ggexport(plotout, width = 8, height = 5, res = 300,
      #    filename = 'Viz/OverallTB/EnsembleCut/BifucationViz.pdf')
    } 
    
    # return output 
    
    if(verbose){
      return(list(sigdiff = nullthreshold < altthreshold,
                  pval = sum(mce_alt <= nullthreshold) /
                    length(mce_alt),
                  encoding = obscode,
                  dist_method = dist_method,
                  obsHTree = encodeobs$hctree,
                  thresholdnull = nullquant,
                  thresholdalt = altquant,
                  MCE_null = mce_null,
                  MCE_alt = mce_alt,
                  plot = plotout))
    }else{
      return(list(sigdiff = nullthreshold < altthreshold,
                  pval = sum(mce_alt <= nullthreshold) /
                    length(mce_alt),
                  encoding = obscode,
                  plot = plotout
      ))
    }
    
  }else{ # either result is inconclusive, or negative and user wants to check down branch
    

    if(is.null(nclustalt)){
      
      # if user doesn't want to look forward, skip this step
    
    # check is multi-cluster necoding is significant  
    }else{ # check is multi-cluster necoding is significant
    
      encodeobs2 <- getBinaryCode(obsdat,
                                 nclust = nclustalt,
                                 simdata = simdata,
                                 hclust_method = hclust_method,
                                 dist_method = dist_method,
                                 weightdist = weightdist,
                                 returnTree = T)
      obscode2 <- encodeobs2$cluster
      mimickcode_alt2 <- apply(simdata, 
                              length(dim(simdata)), 
                              function(x) 
                                getBinaryCode(x, 
                                      simdata = simdata,
                                      nclust = nclustalt,
                                      hclust_method = hclust_method,
                                      dist_method = dist_method,
                                      weightdist = weightdist 
                                      ))
      if(length(dim(nullsim)) == 3){ # analysis of overall TB matrix
        mimickcode_null2 <- apply(nullsim, 
                                 length(dim(nullsim)), 
                                 function(x) 
                                   getBinaryCode(x, 
                                      simdata = nullsim,
                                      nclust = nclustalt,
                                      hclust_method = hclust_method,
                                      dist_method = dist_method
                                      ))
      }else{ # for analysis of tensor
        
      }
      
      mce_alt2 <- apply(mimickcode_alt2, 2, function(x) 
        calcEntropy(table(as.factor(obscode2), as.factor(x)), 
                    logbase = logbase)$MutualInformation)
      mce_null2 <- apply(mimickcode_null2, 2, function(x) 
        calcEntropy(table(as.factor(obscode2), as.factor(x)), 
                    logbase = logbase)$MutualInformation)
      
      nullquant2 <- EnvStats::eqnpar(mce_null2, 0.95, ci = T)
      nullthreshold2 <- nullquant2$quantiles
      nullci2 <- nullquant2$interval$limits
      
      if(length(unique(mce_alt2)) == 1){ # if all sims have same cut
        altquant2 <- NULL 
        altthreshold2 <- unique(mce_alt2)
        altci2 <- c(unique(mce_alt2), unique(mce_alt2))
      }else{
        altquant2 <- EnvStats::eqnpar(mce_alt2, 0.05, ci = T)
        altthreshold2 <- altquant2$quantiles
        altci2 <- altquant2$interval$limits
      }
      
      conftestfail2 <- sum(nullci2 >= altci2[1] 
                           & nullci2 <= altci2[2]) > 0 
      
      # return conditions
      
      if(!conftestfail2 ){
        # return if a confident result is returned, report reslts multi-cluster encoding
        # plot test distributions
        plotdat = data.frame(MCE = c(mce_alt2, mce_null2),
                       Mimickry = c(rep('Observed',
                                              length(mce_alt2)),
                                    rep('Null', length(mce_null2)))
        )
        plotout <- ggplot(plotdat, aes(x = MCE, fill = Mimickry)) +
          geom_density(aes(y = ..scaled..), alpha = 0.75) + 
          ggtitle(paste('Result of Bifucation Simulation: N',
                        nrow(obsdat)))
        if(showplot){  
          print(plotout)
        } 
        
        # return output 
        
        if(verbose){
          return(list(sigdiff = nullthreshold2 < altthreshold2,
                      pval = sum(mce_alt2 <= nullthreshold2) /
                        length(mce_alt2),
                      encoding = obscode,
                      dist_method = dist_method,
                      obsHTree = encodeobs2$hctree,
                      thresholdnull = nullquant2,
                      thresholdalt = altquant2,
                      MCE_null = mce_null2,
                      MCE_alt = mce_alt2,
                      plot = plotout))
        }else{ 
          return(list(sigdiff = nullthreshold2 < altthreshold2,
                      pval = sum(mce_alt2 <= nullthreshold2) /
                        length(mce_alt2),
                      encoding = obscode,
                      plot = plotout
          ))
        }
        
      }else if(nullthreshold >= altthreshold & !conftestfail & conftestfail2){
        # if the original test failed decisively, and now this test is inconclusive, return the original results
        # plot test distributions
        plotdat = data.frame(MCE = c(mce_alt, mce_null),
                       Mimickry = c(rep('Observed',
                                              length(mce_alt)),
                                    rep('Null', length(mce_null)))
        )
        plotout <- ggplot(plotdat, aes(x = MCE, fill = Mimickry)) +
          geom_density(aes(y = ..scaled..), alpha = 0.75) + 
          xlim(c(-0.1,1.1)) + 
          ggtitle(paste('Result of Bifucation Simulation: N',
                        nrow(obsdat)))
        if(showplot){  
          print(plotout)
        } 
        
        # return output 
        
        if(verbose){
          return(list(sigdiff = nullthreshold < altthreshold,
                      pval = sum(mce_alt <= nullthreshold) /
                        length(mce_alt),
                      encoding = obscode,
                      dist_method = dist_method,
                      obsHTree = encodeobs$hctree,
                      thresholdnull = nullquant,
                      thresholdalt = altquant,
                      MCE_null = mce_null,
                      MCE_alt = mce_alt,
                      plot = plotout))
        }else{ 
          return(list(sigdiff = nullthreshold < altthreshold,
                      pval = sum(mce_alt <= nullthreshold) /
                        length(mce_alt),
                      encoding = obscode,
                      plot = plotout
          ))
        }
      }
    
    
  }
    
    # if we cont inconclusive results for binary and multi-clust case, then we must need to increase our sample size and try the test all over again
    

    altB <- dim(simdata)[length(dim(simdata))]
    if((2*altB) <= maxiter){ 
      if(length(dim(obsdat)) == 2){ # sim overal TB
        
        temp <- dim(simdata)
        temp[3] <- 2*altB
        temp2 <- dimnames(simdata)
        temp2[[3]] <- 1:temp[3]
        simdata2 <- array(NA, dim = temp, dimnames = temp2 )
        
        simdata2[ , , 1:altB] <- simdata 
        simdata2[ , , (altB+1):(2*altB)] <- simTimeBudget(rawdata, 
                               B = altB, # double size of sim
                               aggregateTB = 'overall',
                               simMethod = SimSpecs$simMethod,
                               NTempSubsamp = SimSpecs$NTempSubsamp,
                               subsampVar = SimSpecs$subsampVar,
                               agVar = SimSpecs$agVar,
                               verbose = F)$TBSim
        
        return(testBranch(obsdat, simdata2, rawdata, 
                          SimSpecs = SimSpecs,
                          feature_subset = feature_subset, 
                          hclust_method = hclust_method, 
                          dist_method = dist_method, 
                          weightdist = weightdist, 
                          logbase = logbase , 
                          showplot = showplot, 
                          verbose = verbose, 
                          B = (B + 500),
                          maxiter = maxiter))
      }
    }else{ 
      # if we max out iterations, then just return a insignificant result from t he final binary test
      
      # plot test distributions
      plotdat = data.frame(MCE = c(mce_alt, mce_null),
                           Mimickry = c(rep('Observed',
                                            length(mce_alt)),
                                    rep('Null', length(mce_null)))
      )
      plotout <- ggplot(plotdat, aes(x = MCE, fill = Mimickry)) +
        geom_density(aes(y = ..scaled..), alpha = 0.75) + 
        xlim(c(-0.1,1.1)) + 
        ggtitle(paste('Result of Bifucation Simulation: N',
                      nrow(obsdat)))
      if(showplot){  
        print(plotout)
      } 
      
      # return output 
      
      if(verbose){
        return(list(sigdiff = F,
                    pval = sum(mce_alt <= nullthreshold) /
                      length(mce_alt),
                    encoding = obscode,
                    dist_method = dist_method,
                    obsHTree = encodeobs$hctree,
                    thresholdnull = nullquant,
                    thresholdalt = altquant,
                    MCE_null = mce_null,
                    MCE_alt = mce_alt,
                    plot = plotout))
      }else{
        return(list(sigdiff = F,
                    pval = sum(mce_alt <= nullthreshold) /
                      length(mce_alt),
                    encoding = obscode,
                    plot = plotout
        ))
      }
      
    } 
   
    
  }
  
  print('Missed a condition... dammit')

}
  
  

# start_time <- Sys.time()
# testout <- testBranch(Po, simotb$TBSim, sensordat,
#            B = 1000,
#            dist_method = 'Ensemble',
#            SimSpecs = simotb$SimSpecs,
#            hclust_method = 'ward.D2', logbase = 2,
#            showplot = TRUE, verbose = T)
# end_time <- Sys.time()
# end_time - start_time
# 
# start_time <- Sys.time()
# testout <- testBranch(Po, simotb$TBSim, sensordat,
#            B = 1000,
#            dist_method = 'Euclidean',
#            SimSpecs = simotb$SimSpecs,
#            hclust_method = 'ward.D2', logbase = 2,
#            showplot = TRUE, verbose = T)
# end_time <- Sys.time()
# end_time - start_time
# 
# start_time <- Sys.time()
# testout <- testBranch(Po, simotb_tsub$TBSim, sensordat,
#            B = 100,
#            dist_method = 'Ensemble',
#            SimSpecs = simotb_tsub$SimSpecs,
#            hclust_method = 'ward.D2', logbase = 2,
#            showplot = TRUE, verbose = T)
# end_time <- Sys.time()
# end_time - start_time
# 
# start_time <- Sys.time()
# testout <- testBranch(Po[temp,], 
#                       simotb$TBSim[temp,,], 
#                       sensordat[temp,,],
#            B = 2000,
#            dist_method = 'Ensemble',
#            SimSpecs = simotb$SimSpecs,
#            hclust_method = 'ward.D2', logbase = 2,
#            showplot = TRUE, verbose = T)
# end_time <- Sys.time()
# end_time - start_time
```

Alright, now to apply this recursively o.O Everybody hold on to something.

```
# obsdat <- Po
# simdata <- simotb$TBSim
# rawdata <- sensordat
 
# temp <- c("5" , "1511" , "13460" ,"13946" ,"14256" ,"32607" ,"46926", "64584" ,"98737" ,"98771")

#temp <- encodeout_otb_ew$nrow_10_ncol_1$rowclust
#temp <- names(temp[temp == 8 | temp == 9 | temp == 10])

# obsdat <- Po[temp, ]
# simdata <- simotb$TBSim[temp,,]
# rawdata <- sensordat[temp,,]

 # obsdat <- Po
 # simdata <- simotb$TBSim
 # rawdata <- sensordat

recursiveTestBranch <-  function(obsdat, simdata, rawdata, resultout = NULL, nclustalt = NULL, maxiter = 2000, B = 2000, feature_subset = NULL, dist_method = 'Euclidean', weightdist = NULL, hclust_method = 'ward.D2', logbase = 2, SimSpecs = NULL, showplot = FALSE, verbose = F){
  
  # if(is.null(feature_subset)){
  #   feature_subset = names(obsdat)
  # }
  
  if(!is.null(feature_subset)){
    obsdat <- obsdat[ , feature_subset]
    simdata <- simdata[,feature_subset,]
    rawdata <- rawdata[ , , feature_subset]
    if(!is.null((weightdist))){
      weightdist <- weightdist[ , feature_subset]
    }
  }
  
  #print(rownames(obsdat))
  
  if(is.null(resultout)){
    resultout <- list()
  }
  
  if(nrow(obsdat) == 1){ # if node is a leaf, bottom out
    
    resultout[[length(resultout) + 1]] <- list(Type = 'Cluster',
                                          LeafID = rownames(obsdat))
    return(resultout)
    
  }
  
  # get subtree for branch under consideration
  hout <- getBinaryCode(obsdat,
                          simdata = simdata,
                          hclust_method = hclust_method,
                          dist_method = dist_method, 
                          weightdist = weightdist,
                          returnTree = T
                          )$hctree %>% as.dendrogram()
  nh <- hout %>% get_nodes_attr("height")
  

  #print(rownames(obsdat))
  
  if(sum(nh == 0) == length(nh)){ # if leaves are identical, bottom out
    
    resultout[[length(resultout) + 1]] <- list(Type = 'Cluster',
                                          LeafID = rownames(obsdat))
    return(resultout)
    
  }else{ # apply recursion
     
    #print(dim(obsdat[ , feature_subset]))
    #print(rownames(obsdat))
    #print(round(sqrt(Ntot/nrow(obsdat)) * B))
    
    branchresult <- testBranch(obsdat, # obsdat[temp,], 
                               simdata,# simdata[temp,,], 
                               rawdata,# rawdata[temp,,],
                               #B = round(sqrt(Ntot/nrow(obsdat)) * B), # rescale B to account for diminishing branch size (lower confidence MCE estimates) to maintain accuracy if emprical cdf's,
                               B = B,
                               maxiter = maxiter,
                               nclustalt = nclustalt,
                               feature_subset = feature_subset,
                               hclust_method = hclust_method,
                               dist_method = dist_method,
                               weightdist = weightdist,
                               SimSpecs = SimSpecs,
                               logbase = logbase,
                               showplot = showplot,
                               verbose = verbose)
    
      # tryCatch({
      # branchresult <- testBranch(obsdat, # obsdat[temp,], 
      #                          simdata,# simdata[temp,,], 
      #                          rawdata,# rawdata[temp,,],
      #                          #B = round(sqrt(Ntot/nrow(obsdat)) * B), # rescale B to account for diminishing branch size (lower confidence MCE estimates) to maintain accuracy if emprical cdf's,
      #                          B = B,
      #                          maxiter = maxiter,
      #                          nclustalt = nclustalt,
      #                          feature_subset = feature_subset,
      #                          hclust_method = hclust_method,
      #                          dist_method = dist_method,
      #                          weightdist = weightdist,
      #                          SimSpecs = SimSpecs,
      #                          logbase = logbase,
      #                          showplot = showplot,
      #                          verbose = verbose)
      # }, warning = function(w) { print(w) }  )
    #print(branchresult$pval)
    #print(branchresult)
    
    if(!branchresult$sigdiff){
      
      resultout[[length(resultout) + 1]] <- list(Type = 'Cluster',
                                          LeafID = rownames(obsdat))
      return(resultout)
      
    }else{
      
      resultout[[length(resultout) + 1]] <- list(Type = 'Branch',
                                            SimResult = branchresult)
      
      
      # obscode <- getBinaryCode(obsdat[ , feature_subset],
      #                          hclust_method = hclust_method)
      obscode <- branchresult$encoding
      resultout <- recursiveTestBranch(obsdat[obscode == 1, ],
                          simdata[obscode == 1, ,], 
                          rawdata[obscode == 1, ,],             
                          resultout = resultout,
                          nclustalt = nclustalt,
                          maxiter = maxiter,
                          B = B,
                          feature_subset = feature_subset,
                          hclust_method = hclust_method,
                          dist_method = dist_method,
                          weightdist = weightdist[obscode == 1,],
                          SimSpecs = SimSpecs,
                          logbase = logbase,
                          showplot = showplot,
                          verbose = verbose
                          )
      resultout <- recursiveTestBranch(obsdat[obscode == 2, ],
                          simdata[obscode == 2, ,], 
                          rawdata[obscode == 2, ,],             
                          resultout = resultout,
                          nclustalt = nclustalt,
                          maxiter = maxiter,
                          B = B,
                          feature_subset = feature_subset,
                          hclust_method = hclust_method,
                          dist_method = dist_method,
                          weightdist = weightdist[obscode == 2,],
                          SimSpecs = SimSpecs,
                          logbase = logbase,
                          showplot = showplot,
                          verbose = verbose
                          )
    }
  }
  
}

start_time <- Sys.time()
rout <- recursiveTestBranch(Po[temp,],
                      simotb$TBSim[temp,,],
                      sensordat[temp,,],
                            nclustalt = 3,
                            dist_method = 'Ensemble',
                            SimSpecs = simotb$SimSpecs,
                            verbose = T, showplot = T)
```

```
end_time <- Sys.time()
end_time - start_time
```

```
## Time difference of 8.103506 mins
```

```
start_time <- Sys.time()
rout <- recursiveTestBranch(Po[temp,],
                      simotb_tsub$TBSim[temp,,],
                      sensordat[temp,,],
                            nclustalt = 3,
                            dist_method = 'Ensemble',
                            SimSpecs = simotb_tsub$SimSpecs,
                            verbose = T, showplot = T)
```

```
end_time <- Sys.time()
end_time - start_time
```

```
## Time difference of 7.944622 mins
```

And now a wrapper to process the output nicely. I’ll use the dendextend for the visualization

https://cran.r-project.org/web/packages/dendextend/vignettes/dendextend.html#adding-extra-bars-and-rectangles

```
# cutoff = 0.01
# B = 50
# hclust_method = 'ward.D2'
# dist_method = 'Euclidean'
# logbase = 2
# showmSimPlots = T
# verbose = F
# showDendrogram = TRUE
# feature_subset = names(P)
# aggregateTB = 'overall'
# agVar = NULL

cutreeEnsemble <- function(obsdat, simdata, rawdata, nclustalt = NULL, B = 2000, maxiter = 2000, feature_subset = NULL, hclust_method = 'ward.D2', dist_method = 'Euclidean', weightdist = NULL, SimSpecs = NULL, logbase = 2, showmSimPlots = FALSE, verbose = F, showDendrogram = TRUE, showHeatmap = T, exportPlots = F, filename = 'EnsembleCutPlot', imSpecDend = list(imwidth = 10, imheight = 20, imres = 300), imSpecHeatmap = list(imwidth = 12, imheight = 18), imres = 300, ...){ # dendColorPalette ='Paired'
  
  require(dendextend)
  require(RColorBrewer)
  #require(DirichletReg)
  
  # if(is.null(feature_subset)){
  #   feature_subset = names(obsdat)
  # }
  
  if(!is.null(feature_subset)){
    obsdat <- obsdat[ , feature_subset]
    simdata <- simdata[,feature_subset,]
    rawdata <- rawdata[ , , feature_subset]
    if(!is.null((weightdist))){
      weightdist <- weightdist[ , feature_subset]
    }
  }
 
  rout <- recursiveTestBranch(obsdat,  # obsdat[temp,], 
                              simdata, # simdata[temp,,], 
                              rawdata, # rawdata[temp,,],
                              nclustalt = nclustalt,
                              maxiter = maxiter,
                              B = B, 
                              feature_subset = feature_subset,
                              hclust_method = hclust_method, 
                              logbase = logbase, 
                              showplot = showmSimPlots,
                              verbose = verbose,
                              dist_method = dist_method,
                              SimSpecs = SimSpecs,
                              weightdist = weightdist) 
  
  # return vector of cluster ID's numbered in order returned by recursive
  
  cutout <- data.frame(ObsID = NA, 
                       ClusterID = NA,
                       stringsAsFactors = F)
  mylevels <- 1
  if(length(rout) > 1){
    for(i in 1:length(rout)){
      
      if(rout[[i]]$Type == 'Cluster'){
        
       # if(sum(is.na(rout[[i]]$LeafID))){print(i)}
        
        cutout <- rbind(cutout,
                      data.frame(ObsID = rout[[i]]$LeafID, 
                                 ClusterID = rep(mylevels,
                                             length(rout[[i]]$LeafID)),
                                 stringsAsFactors = F)
                      )
        mylevels <- mylevels + 1
      }

    }
  }else{
    cutout$ClusterID <- 1
  } 
  

  
  cutout <- cutout[-1, ]
  rownames(cutout) <- cutout$ObsID
  #print(sum(is.na(rownames(cutout))))
  
  clusterout_data <- cutout[match(rownames(obsdat), rownames(cutout)), ]
  #print(sum(is.na(clusterout_data$ClusterID))) 
  

  
  # Reassign cluster ID values to match order of dendrogram
  
  htreeout <- getBinaryCode(obsdat,
                          simdata = simdata,
                          dist_method = dist_method,
                          hclust_method = hclust_method,
                          returnTree = T
                          )$hctree 
  hout <- as.dendrogram(htreeout)
  #print(labels(hout))
  #print(sum(is.na(labels(hout))))
  
  
  #print(unique(clusterout_data$ClusterID)) 
  temp <- clusterout_data[order.dendrogram(hout),] # data ordered to mach dendrogram lables
  ctemp <- unique(temp$ClusterID)
  #print(ctemp)
  
  clusterout_data$DendID <- NA
  
  for (i in 1:length(ctemp)){
    clusterout_data$DendID[clusterout_data$ClusterID == ctemp[i]] <- i
  }
  #print(sum(is.na(clusterout_data$DendID)))
  
  
  # Dendrogram Viz
  
  temp <- clusterout_data[order.dendrogram(hout),] # data ordered to mach dendrogram lables
  col <- rainbow(length(ctemp))
  #col <- brewer.pal(length(ctemp), dendColorPalette)
  
  for(i in 1:length(ctemp)){
    hout <- set(hout, 'by_labels_branches_col', 
                value = temp$ObsID[temp$DendID == i], 
                TF_values = c(col[i],Inf))
  }
  
  dendout <- as.ggdend(hout)
  dendout <- ggplot(dendout, theme = theme_minimal(), horiz = T)
  if(showDendrogram){  
    plot(hout)
  }
  
  
  # Heatmap Viz
  
  temp <- clusterout_data[order.dendrogram(hout),] # data ordered to mach dendrogram lable
  mygaps <- c()
  for(i in 1:(length(table(temp$DendID)) - 1) ){
    mygaps <- c(mygaps, max(which(temp$DendID == i)))
  }

  plotout <- pheatmap::pheatmap(
      mat = obsdat[order.dendrogram(hout),],
      cluster_rows = F,
      #clustering_method = "ward.D2",
      #annotation_row = auxmat_tbvar,
      #annotation_colors  = auxcol_var,
      gaps_row = mygaps,
      silent = T,
      ...)
  
  if(showHeatmap){
    plot.new()
    print(plotout)
  }
  
  
  # Export Plots
  
  if(exportPlots){
    ggpubr::ggexport(dendout ,
                         width = imSpecDend$imwidth, 
                         height = imSpecDend$imheight, 
                         res = imres,
                         filename = paste(filename,
                                          '_Dendrogram', 
                                          '.pdf', sep = ''))
    ggpubr::ggexport(plotout,
                         width = imSpecHeatmap$imwidth, 
                         height = imSpecHeatmap$imheight, 
                         res = imres,
                         filename = paste(filename,
                                          '_Heatmap', 
                                          '.pdf', sep = ''))
  }
  
 

  # Return Info
  
  outdat <- clusterout_data$DendID
  names(outdat) <- clusterout_data$ObsID
  
  return(list(SimResults = rout,
              FinalEncoding = outdat,
              Dendrogram = hout,
              DendrogramPlot = dendout,
              Heatmap = plotout))
  
}

# obsdat <- Po 
# simdata <- simotb$TBSim 
# rawdata <- sensordat
# 
# start_time <- Sys.time()
# CEout <- cutreeEnsemble( Po, simotb$TBSim, sensordat,
#   #Po[temp,], simotb$TBSim[temp,,], sensordat[temp,,],
#                         nclustalt = 3,
#                         dist_method = 'Euclidean',
#                         SimSpecs = simotb$SimSpecs,
#                         showmSimPlots = T, verbose = T)
# end_time <- Sys.time()
# end_time - start_time
# 
# 
# start_time <- Sys.time()
# CEout <- cutreeEnsemble( Po, simotb$TBSim, sensordat,
#   #Po[temp,], simotb$TBSim[temp,,], sensordat[temp,,],
#                         nclustalt = 3,
#                         dist_method = 'Ensemble',
#                         SimSpecs = simotb$SimSpecs,
#                         showmSimPlots = T, verbose = T)
# end_time <- Sys.time()
# end_time - start_time
```

Alright, and now finally, with the output from ensembleCut, I want to create a new tree object the contains only the validated branches. To give myself more flexibility downstream, I’ll create a difference matrix, wherein the distance between any two observations is the tree difference between validated branches, and all other distances are set to zero. By default, I’ll also normalize the distances so that the longest distances between validated branches is set to one (with an option also to return the raw dist)

https://cran.r-project.org/web/packages/dendextend/vignettes/dendextend.html#getting-nodes-attributes-in-a-depth-first-search

```
#cutout <- CEotb_subh_euc
normTreeDist <- function(cutout, normalize = T){
  
  
  roworder <- names(cutout$FinalEncoding)
  distmat <- as.matrix(cophenetic(cutout$Dendrogram))
  
  for(i in 1:nrow(distmat)){
    tempcow <- rownames(distmat)[i]
    temp <-names(cutout$FinalEncoding)[cutout$FinalEncoding[tempcow]
                                      == cutout$FinalEncoding]
    distmat[i, temp] <- 0
    
  }
  
  distmat <- distmat[roworder, roworder] # reorder dist mat to match order of orignal data
  
  if(normalize){
    distmat <- distmat/max(distmat)
  }
  
  return(as.dist(distmat))
  
}

#temp <- normTreeDist(CEotb_subh_euc, normalize = T)
#image(as.matrix(temp))

#plot(hclust(temp, method = 'single'))
#plot(CEotb_subh_euc$Dendrogram)
```

## Pruning Dendrograms

### Euclidean

First a null simulation using all data

```
set.seed(61916)

CEotb_allh_euc <- cutreeEnsemble(Po, simotb$TBSim, sensordat,
                            nclustalt = 3,
                            SimSpecs = simotb$SimSpecs,
                            showmSimPlots = F, verbose = T,
                            exportPlots = T, 
                            filename = 'Viz/OverallTB/EnsembleCut/Euclidean_NP',
                            main = 'Noise-Penalized Ensemble Cut Encoding: Euclidean Distance',
                            imSpecHeatmap = list(imwidth = 12,
                                                 imheight = 23)
                            )
```

```
## file saved to Viz/OverallTB/EnsembleCut/Euclidean_NP_Dendrogram.pdf
```

```
## file saved to Viz/OverallTB/EnsembleCut/Euclidean_NP_Heatmap.pdf
```

```
max(CEotb_allh_euc$FinalEncoding)
```

```
## [1] 39
```

And now the temporally subsetted ensemble

```
set.seed(61916)

CEotb_subh_euc <- cutreeEnsemble(Po, simotb_tsub$TBSim, sensordat,
                            nclustalt = 3,
                            SimSpecs = simotb_tsub$SimSpecs,
                            showmSimPlots = F, verbose = T,
                            exportPlots = T, 
                            filename = 'Viz/OverallTB/EnsembleCut/Euclidean_PP',
                            main = 'Plasticity-Penalized Ensemble Cut Encoding: Euclidean Distance',
                            imSpecHeatmap = list(imwidth = 12,
                                                 imheight = 22)
                            )
```

```
## file saved to Viz/OverallTB/EnsembleCut/Euclidean_PP_Dendrogram.pdf
```

```
## file saved to Viz/OverallTB/EnsembleCut/Euclidean_PP_Heatmap.pdf
```

```
max(CEotb_subh_euc$FinalEncoding)
```

```
## [1] 13
```

```
rowdisto <- normTreeDist(CEotb_subh_euc, normalize = T)
coldisto <- dist(t(Po))

encodeout_otb_euc <- encodeplot(Po,
                       rowdisto,
                       coldisto,
                       hclust_method_r = 'single',
                       n_rowclusters = 1:10, 
                       n_colclusters = 0,
                       showplot = F, 
                       exportplot = F,
                       imwidth = 12,
                       imheight = 22,
                       imres = 300,
                       plot_title = 'Plasticity-Penalized Ensemble Cut Encoding: Euclidean Distance'
                       )

plot(encodeout_otb_euc$nrow_10_ncol_0$plot)
```

### KLD

First a null simulation using all data

```
set.seed(61916)

CEotb_allh_kld <- cutreeEnsemble(Po, simotb$TBSim, sensordat,
                            dist_method = 'KLD',
                            nclustalt = 3,
                            SimSpecs = simotb$SimSpecs,
                            showmSimPlots = F, verbose = T,
                            exportPlots = T, 
                            filename = 'Viz/OverallTB/EnsembleCut/KLD_NP',
                            main = 'Noise-Penalized Ensemble Cut Encoding: KL Distance',
                            imSpecHeatmap = list(imwidth = 12,
                                                 imheight = 23)
                            )
```

```
## file saved to Viz/OverallTB/EnsembleCut/KLD_NP_Dendrogram.pdf
```

```
## file saved to Viz/OverallTB/EnsembleCut/KLD_NP_Heatmap.pdf
```

```
max(CEotb_allh_kld$FinalEncoding)
```

```
## [1] 31
```

And now the temporally subsetted ensemble

```
set.seed(61916)

CEotb_subh_kld <- cutreeEnsemble(Po, simotb_tsub$TBSim, sensordat,
                            dist_method = 'KLD',
                            nclustalt = 3,
                            SimSpecs = simotb_tsub$SimSpecs,
                            showmSimPlots = F, verbose = T,
                            exportPlots = T, 
                            filename = 'Viz/OverallTB/EnsembleCut/KLD_Sub',
                            main = 'Plasticity-Penalized Ensemble Cut Encoding: KL Distance',
                            imSpecHeatmap = list(imwidth = 12,
                                                 imheight = 22)
                            )
```

```
## file saved to Viz/OverallTB/EnsembleCut/KLD_Sub_Dendrogram.pdf
```

```
## file saved to Viz/OverallTB/EnsembleCut/KLD_Sub_Heatmap.pdf
```

```
max(CEotb_subh_kld$FinalEncoding)
```

```
## [1] 17
```

```
rowdisto <- normTreeDist(CEotb_subh_kld, normalize = T)
coldisto <- dist(t(Po))

encodeout_otb_kld <- encodeplot(Po,
                       rowdisto,
                       coldisto,
                       hclust_method_r = 'single',
                       n_rowclusters = 1:10, 
                       n_colclusters = 0,
                       plot_title = 'Plasticity-Penalized Ensemble Cut Encoding: KL Distance',
                       showplot = F, 
                       exportplot = F,
                       imwidth = 12,
                       imheight = 22,
                       imres = 300
                       )

plot(encodeout_otb_kld$nrow_10_ncol_0$plot)
```

### Noise Penalized

First I’ll run the noise-weighted dendrogram against the noise-penalized ensmble cut algorithm.

```
set.seed(61916)

CEotb_allh_ew <- cutreeEnsemble(Po, simotb$TBSim, sensordat,
                            dist_method = 'Ensemble',
                            nclustalt = 3,
                            SimSpecs = simotb$SimSpecs,
                            showmSimPlots = F, verbose = T,
                            exportPlots = T, 
                            filename = 'Viz/OverallTB/EnsembleCut/NoisePen_NP',
                            main = 'Noise-Penalized Ensemble Cut Encoding: Noise-Penalized Distance',
                            imSpecHeatmap = list(imwidth = 12,
                                                 imheight = 22)
                            )
```

```
## file saved to Viz/OverallTB/EnsembleCut/NoisePen_NP_Dendrogram.pdf
```

```
## file saved to Viz/OverallTB/EnsembleCut/NoisePen_NP_Heatmap.pdf
```

```
max(CEotb_allh_ew$FinalEncoding)
```

```
## [1] 38
```

```
rowdisto <- normTreeDist(CEotb_allh_ew, normalize = T)
coldisto <- dist(t(Po))

encodeout_otb_ew <- encodeplot(Po,
                       rowdisto,
                       coldisto,
                       hclust_method_r = 'single',
                       n_rowclusters = 1:18, 
                       n_colclusters = 0,
                       plot_title = 'Noise-Penalized Ensemble Cut Encoding: Noise-Penalized Distance',
                       showplot = F, 
                       exportplot = T,
                       filename = 'Viz/OverallTB/EnsembleCut/NPEncode/NPCut/NPEncode',
                       imwidth = 12,
                       imheight = 22,
                       imres = 300)

plot(encodeout_otb_ew$nrow_10_ncol_0$plot)
```

Now I’ll run the noise-weighted dendrogram against the plasticity-penalized ensmble cut algorithm.

```
set.seed(61916)

CEotb_subh_ew <- cutreeEnsemble(Po, simotb_tsub$TBSim, sensordat,
                            dist_method = 'Ensemble',
                            nclustalt = 3,
                            SimSpecs = simotb_tsub$SimSpecs,
                            weightdist = ensembleweightOTB$weights,
                            showmSimPlots = F, verbose = T,
                            exportPlots = T, 
                            filename = 'Viz/OverallTB/EnsembleCut/NPEncode/PPCut',
                            main = 'Plasticity-Penalized Ensemble Cut Encoding: Noise-Penalized Distance',
                            imSpecHeatmap = list(imwidth = 12,
                                                 imheight = 22)
                            )
```

```
## file saved to Viz/OverallTB/EnsembleCut/NPEncode/PPCut_Dendrogram.pdf
```

```
## file saved to Viz/OverallTB/EnsembleCut/NPEncode/PPCut_Heatmap.pdf
```

```
max(CEotb_subh_ew$FinalEncoding)
```

```
## [1] 14
```

```
rowdisto <- normTreeDist(CEotb_subh_ew, normalize = T)
coldisto <- dist(t(Po))

encodeout_otb_ewPP <- encodeplot(Po,
                       rowdisto,
                       coldisto,
                       hclust_method_r = 'single',
                       n_rowclusters = 1:14, 
                       n_colclusters = 0,
                       plot_title = 'Plasticity-Penalized Ensemble Cut Encoding: Noise-Penalized Distance',
                       showplot = F, 
                       exportplot = T,
                       filename = 'Viz/OverallTB/EnsembleCut/NPEncode/PPCut/NPEncode',
                       imwidth = 12,
                       imheight = 22,
                       imres = 300)

plot(encodeout_otb_ewPP$nrow_10_ncol_0$plot)
```

### Plasticity Penalized

Now I’ll run the plasticity-weighted dendrogram against the plasticity-weighted ensmble cut algorithm.

```
set.seed(61916)

CEotb_subh_pw <- cutreeEnsemble(Po, simotb_tsub$TBSim, sensordat,
                            dist_method = 'Ensemble',
                            nclustalt = 3,
                            SimSpecs = simotb_tsub$SimSpecs,
                            showmSimPlots = F, verbose = T,
                            exportPlots = T, 
                            filename = 'Viz/OverallTB/EnsembleCut/PlastPen_PP',
                            main = 'Plasticity-Penalized Ensemble Cut Encoding: Plasticity-Penalized Distance',
                            imSpecHeatmap = list(imwidth = 12,
                                                 imheight = 22)
                            )
```

```
## file saved to Viz/OverallTB/EnsembleCut/PlastPen_PP_Dendrogram.pdf
```

```
## file saved to Viz/OverallTB/EnsembleCut/PlastPen_PP_Heatmap.pdf
```

```
max(CEotb_subh_pw$FinalEncoding)
```

```
## [1] 14
```

```
rowdisto <- normTreeDist(CEotb_subh_pw, normalize = T)
coldisto <- dist(t(Po))

encodeout_otb_pw <- encodeplot(Po,
                       rowdisto,
                       coldisto,
                       hclust_method_r = 'single',
                       n_rowclusters = 1:14, 
                       n_colclusters = 0,
                       plot_title = 'Plasticity-Penalized Ensemble Cut Encoding: Plasticity-Penalized Distance',
                       filename = 'Viz/OverallTB/EnsembleCut/PPEncode/PWEncode',
                       showplot = F, 
                       exportplot = T,
                       imwidth = 12,
                       imheight = 22,
                       imres = 300
                       )

plot(encodeout_otb_pw$nrow_10_ncol_0$plot)
```

```
plotout <- ggpubr::ggarrange(
            ggplotify::as.ggplot(CEotb_allh_ew$Heatmap), #encodeout_otb_ew$nrow_18_ncol_0$plot
            ggplotify::as.ggplot(CEotb_subh_pw$Heatmap), #encodeout_otb_pw$nrow_13_ncol_0$plot
            ncol = 2, nrow = 1)

ggpubr::ggexport(plotout,
                         width = 20, 
                         height = 22, 
                         res = 300,
                         filename = 'Viz/OverallTB/EnsembleCut/EnsembleEncodings.pdf')
```

```
## file saved to Viz/OverallTB/EnsembleCut/EnsembleEncodings.pdf
```

```
EncodingsOTB <- list(Euclidean = encodeout_otb_euc,
                     KLD = encodeout_otb_kld,
                     Ensemble = encodeout_otb_ewPP,
                     Plasticity = encodeout_otb_pw
                     )
```

```
save.image("OverallTB.RData")
```

# Mutual Information - Bivariate Tests

## Define Functions

First I need to load up a function to calculate mutual information. This will take either a vector for univariate measures or a contingency table/array for multivariate systems

https://jmlr.csail.mit.edu/papers/volume10/hausser09a/hausser09a.pdf https://arxiv.org/pdf/physics/0108025.pdf https://memming.wordpress.com/2014/02/09/a-guide-to-discrete-entropy-estimators/

Note: This is going to over-ride the entropy function currently in LIT, as it will add the trivar estimators and allow for bias-corrections to be applied.

```
# dat <- matrix(c(1,2,3,4,2,3,4,5,3,4,5,6), nrow = 4)


calcEntropy <- function(counts, logbase = 2, estimator = 'ML', verbose = F, isfreq = F, ...){

  if(estimator == 'CS'){
    if(logbase == 2){
      myunit = 'log2'
    }else if(logbase == 10){
      myunit = 'log10'
    }else if(logbase == exp(1)){
      myunit = 'log'
    }else{
      if(estimator != 'ML'){
        stop('Error: CS estimator only supports log bases 2, 10, or e (natural log)')
      }
    }
  }

  # format as matrix/3d array
  if(!is.matrix(counts) & !is.array(counts)){
    if(length(dim(counts)) < 3){
      dat <- as.matrix(counts)
    }else{
      dat <- array(as.matrix(counts),
                   dim = dim(counts),
                   dimnames = dimnames(counts))
    }
  }else{
    dat <- counts
  }

  # convert to frequencies

  if(isfreq & estimator == 'ML'){
    p = dat
  }else if(estimator == 'ML'){
    p <- dat/sum(dat)
  }else if(estimator == 'shrink'){

    if( sum(dim(dat)>1) == 1 ){
      p <- as.numeric(entropy::freqs.shrink(dat, verbose = F)) # get rid of lambda attrib attached to vector
    }else{
      p <- entropy::freqs.shrink(dat, verbose = F)
    }

  }else if(estimator %in% c("Jeffreys","Laplace", "SG", "minmax")){

    if(estimator == "Jeffreys"){
      a = 1/2
    }else if(estimator == 'Laplace'){
      a = 1
    }else if(estimator == 'SG'){
      a = 1/length(as.vector(dat))
    }else if(estimator == "minmax"){
      a = sum(dat)^0.5 / length(as.vector(dat))
    }else{
      stop('Error: Estimator method not supported')
    }

    p <- entropy::freqs.Dirichlet(dat, a = a)

  }else{
    stop('Error: Estimator method not supported')
  }


  if( sum(dim(dat)>1) == 1 ){ # 1D entropy

    if(sum(dat) == 0){ # if count vector is empty, set H to 0
      H <- 0
    }else{

      if(estimator == 'CS'){
        H <- as.numeric(entropy::entropy(dat, unit = myunit, method = 'CS', verbose = F))
      }else{
        H <- sum(p[p!=0] * log(1/p[p!=0], base = logbase))
      }

    }

    maxH <- log(length(dat), base = logbase)

    if(verbose){
      pout = p
    }else{
      pout = NULL
    }

    return(list(Entropy = H,
                MaxEntropy = maxH,
                Redundancy = 1 - H/maxH,
                estimator = estimator,
                logbase = logbase,
                p = pout
                ))

  }else if( sum(dim(dat)>1) == 2 ){

    # marginal entropy
    margC <- calcEntropy( apply(p, 2, sum), logbase = logbase, isfreq = T)
    margR <- calcEntropy( apply(p, 1, sum), logbase = logbase, isfreq = T)

    # joint entropy
    jointH <- calcEntropy(as.vector(p), logbase = logbase, isfreq = T)$Entropy

    # conditional entropy
    pcol <- apply(p, 2, sum)
    chrow = sum(pcol * apply(p, 2, function(x) calcEntropy(x/ifelse(sum(x)==0,1,sum(x)), logbase = logbase, isfreq = T)$Entropy) )

    prow <- apply(p, 1, sum)
    chcol = sum(prow * apply(p, 1, function(x) calcEntropy(x/ifelse(sum(x)==0,1,sum(x)), logbase = logbase, isfreq = T)$Entropy) )

    if(verbose){
      pout = p
    }else{
      pout = NULL
    }

    return(list(MarginalEntropyRow = margR,
                MarginalEntropyCol = margC,
                JointEntropy = jointH,
                ConditionEntropyRow = chrow,
                ConditionEntropyCol = chcol,
                MutualInformation = margR$Entropy - chrow,
                RelMI = (margR$Entropy - chrow)/jointH,
                estimator = estimator,
                logbase = logbase,
                p = pout
                ))


  }else if( sum(dim(dat) > 1) == 3 ){

    ogdim <- dim(dat)

    MI_XY <- calcEntropy(apply(p,
                               c(1,2),
                               function(x) sum(x, na.rm = T)
                               ),
                         logbase = logbase,
                         isfreq = T)
    MI_XZ <- calcEntropy(apply(p,
                               c(1,3),
                               function(x) sum(x, na.rm = T)
                               ),
                         logbase = logbase,
                         isfreq = T
                         )
    MI_YZ <- calcEntropy(apply(p,
                               c(2,3),
                               function(x) sum(x, na.rm = T)
                               ),
                         logbase = logbase,
                         isfreq = T
                         )

    # Calculate H(X;Y:Z)

    H_XYZ <- calcEntropy(as.vector(p),
                         logbase = logbase,
                         isfreq = T)$Entropy

    # Calculate I(X;Y | Z)

    H_X_Z <- MI_XZ$ConditionEntropyRow #H(X|Z)

    datx_yz <- p
    dim(datx_yz) <- c(ogdim[1], ogdim[2] * ogdim[3])
    H_X_YZ <- calcEntropy(datx_yz,
                          logbase = logbase,
                          isfreq = T)$ConditionEntropyRow

    MCE_XY <- H_X_Z - H_X_YZ

    # Calculate I(X;Z | Y)

    H_X_Y <- MI_XY$ConditionEntropyRow

    MCE_XZ <- H_X_Y - H_X_YZ

    # Calculate I(Y;Z | X)

    datz_xy <- p
    dim(datz_xy) <- c(ogdim[1] * ogdim[2] , ogdim[3])
    H_Z_X <- MI_XZ$ConditionEntropyCol
    H_Z_XY <- calcEntropy(datz_xy,
                          logbase = logbase,
                          isfreq = T)$ConditionEntropyCol

    MCE_YZ <- H_Z_X - H_Z_XY

    # Mutual Information

    IXYZ <- MI_XY$MutualInformation - MCE_XY
    totcor <- MCE_XY + MCE_XZ + MCE_YZ + IXYZ

    return(list(JointEntropy = H_XYZ,
                MutualInformation = IXYZ,
                MCE_XY = MCE_XY,
                MCE_XZ = MCE_XZ,
                MCE_YZ = MCE_YZ,
                MarginalMI_XY = MI_XY,
                MarginalMI_XZ = MI_XZ,
                MarginalMI_YZ = MI_YZ,
                TotalCorrelation = totcor,
                MI_TotCor = ifelse(totcor == 0, 0, IXYZ/totcor)
                ))

  }else{
    print('Error: Sorry, more than 3 dimensions is above my pay grade')
  }

}
```

Now I want to derive a permutation test based on mutual information statistic to assess the statistical significance of the observed level of association between two encodings.

By default I’ll use the ML estimator. However, there is a risk with extremely heterogenous and/or small datasets that undersampled empty cells will cause the entropy estiamtes to be biased downwards. Since our observed data is more likely to contain empty cells than a purely randomized dataset, this bias may not be uniform, which could make our comparisons anti-conservative, so switching to a bias corrected entorpy estimator is recommended.

Such a test, on its own, can only tell me if distribution of obs between encodings appears to be non-random, it cannot tell me “how” its nonrandom. So I also want to build into my compareEncoding visualization tools a partial mutual conditional entropy option to tell be which crosses are significantly under- and over-populated when compared with purely randomized data.

Note: For bias-corrected estimators, the total entropy may change between null and alternative, so by default I’ll rescale my estimator to be proportion of total joint entropy attributed to mutual information

```
 #  codex <- encodeout_otb_euc$nrow_6_ncol_1$rowclust
 # codey <- encodeout_otb_ew$nrow_6_ncol_1$rowclust
 # table(codex,codey)

MITest <- function(codex, codey, 
                   nperm = 1000, logbase = 2, 
                   estimator = 'ML',
                   rescale = T,
                   verbose = F,
                   plotresults = F){
  # codex = data vector of row categorical variable
  # codey = dat vector of column categorical variable
  # nperm = number of permutations 
  # NOTE: x and y should by aligned by observed individuals
  
    if(is.data.frame(codex)){
      if(1 %in% dim(codex)){
        temp <- codex[,1]
        names(temp) <- rownames(codex)
        codex <- temp
      }else{
        stop('Error: Encoding X must be data vector')
      }
    }
  
    if(is.data.frame(codey)){
      if(1 %in% dim(codey)){
        temp <- codey[,1]
        names(temp) <- rownames(codey)
        codey <- temp
      }else{
        stop('Error: Encoding Y must be data vector')
      }
    }
  
  if( sum(names(codey) != names(codex)) > 0 ){
    stop('Error: Observations do not align between input vectors')
  }
    
    obsinformtation <- calcEntropy(table(codex,codey), 
                          logbase = logbase,
                          estimator = estimator)
    if(rescale){
      entobs <- obsinformtation$RelMI
    }else{
      entobs <- obsinformtation$MutualInformation
    }
    
    
    randent <- rep(NA, nperm)
    for(i in 1:nperm){
      xr <- sample(codex, replace = F)
      yr <- sample(codey, replace = F)
      
      if(rescale){
        randent[i] <- calcEntropy(table(xr, yr), 
                        logbase = logbase,
                        estimator = estimator)$RelMI
      }else{
        randent[i] <- calcEntropy(table(xr, yr), 
                        logbase = logbase,
                        estimator = estimator)$MutualInformation
      }
    }
    
    plotout <- NULL
    if(plotresults){
      xmin <- min(c(randent,entobs))-0.1*abs(min(c(randent,entobs)))
      xmax <-max(c(randent,entobs))+0.1*abs(max(c(randent,entobs)))
      plotout <- qplot(randent, 
                       main = 'Histogram of Randomized Mutual Information Values',
                       xlim = c(xmin,xmax)) +
                 geom_vline(xintercept=entobs, col = 'red')
      print(plotout)
    }   
        
    if(verbose){     
      return(list(pval = sum(randent>entobs)/nperm,
                  ObsCounts = table(codex,codey),
                  ObsEntropy = obsinformtation,
                  NullDist = randent,
                  Plot = plotout,
                  testSpecs = list(
                    logbase = logbase,
                    nperm = nperm,
                    rescale = rescale,
                    estimator = estimator
                  )
            ))
    }else{
      return(list(pval = sum(randent>entobs)/nperm,
                  ObsEntropy = obsinformtation,
                  testSpecs = list(
                    logbase = logbase,
                    nperm = nperm,
                    rescale = rescale,
                    estimator = estimator
                  )
            ))
    }
    
}

# MITest(encodeout_otb_euc$nrow_6_ncol_1$rowclust, 
#        encodeout_otb_ew$nrow_6_ncol_1$rowclust, 
#        plotresults = T)$pval
# 
# MITest(cutree(encodeout_otb_euc$nrow_6_ncol_1$rowtree, 5), 
#        cutree(encodeout_otb_ew$nrow_6_ncol_1$rowtree, 5), 
#        plotresults = T)$pval
```

Alright, now I want to provde a utility that will allow me to test bivariate associations on an grid of encoding granularities. I want to be able to either provide a tree, which i can cut using standard cutree() on a grid of values, or I want to be able to provide list output, formated as in encodePlot or dmplotGrid, that will pull out the encodings on the resulting grid.

Note - if the number of columns is not fixed, then bivarEncodeTest will for a given combination of row grid values, parse through all the column values and return the result for the column value with the best mutual information

Note: I’ll allow the user to input a vector with pre-determined encoding, and the corresponding grid option will just be over-ridden to the number of levels. But it must be formated as data frame with rownames

```
set.seed(61916)

# inputdatx <- encodeout_otb_kld
# inputdaty <- encodeout_otb_euc
# 
# inputdaty <- encodeout_otb_ew$nrow_1_ncol_0$rowtree
# inputdaty <- dmgridout_all

# inputdatx <- data.frame(SickPen = cowattribdat$Sick_Prepasture)
# rownames(inputdatx) <- cowattribdat$CowID

#inputdaty <- dmgridout_healthy


# inputdaty <- dmgridout_all #$nrow_4_ncol_3
# grid_y = 2:10
# inputdatx <- encodeout_otb_ew #$nrow_5_ncol_0
# grid_x = 2:10

bivarEncodeTest <- function(inputdatx, inputdaty, 
                        grid_x = 2:5, grid_y = 2:5,
                        logbase = 2, nperm = 1000, 
                        estimator = 'ML', rescaleMI = T,
                        verbose = F){
  
  
  if(1 %in% grid_x | 1 %in% grid_y ) {
    stop("Error: Cluster size must be >1")
  }

  
  ### Pre-process vector with fixed encoding
  
  if(is.data.frame(inputdatx)){
    
    if(.row_names_info(inputdatx)<0){stop('Error: Data frame must have rownames corresponding to observation ID')}
    if(dim(inputdatx)[2] > 1){stop('Error: If providing encoding vector, must be formated with ncol(inputdatx) = 1 ')}
    
    tempdat <- inputdatx[,1]
    names(tempdat) <- rownames(inputdatx)
    rownames_x <- rownames(inputdatx)
    
    grid_x <- sum(!is.na(unique(inputdatx)))
    
    temp <- list()
    temp[[paste('nrow', grid_x, 'ncol_0', sep='_')]] <- 
      list(rowclust = tempdat)
    inputdatx <- temp
    
    dimx <- "ncol_0"
    
    
  }
  
  
  if(is.data.frame(inputdaty)){
    
    if(.row_names_info(inputdaty)<0){stop('Error: Data frame must have rownames corresponding to observation ID')}
    if(dim(inputdaty)[2] > 1){stop('Error: If providing encoding vector, must be formated with ncol(inputdaty) = 1 ')}
    
    tempdat <- inputdaty[,1]
    names(tempdat) <- rownames(inputdaty)
    rownames_y <- rownames(inputdaty)
    
    grid_y <- sum(!is.na(unique(inputdaty)))
    
    temp <- list()
    temp[[paste('nrow', grid_y, 'ncol_0', sep='_')]] <- 
      list(rowclust = tempdat)
    inputdaty <- temp
    
    dimy <- "ncol_0"
    
  }
  
  ### Pre-process tree inputs ###
    
  if(dendextend::is.hclust(inputdatx)){ # format x tree as list
    
    rownames_x <- inputdatx$labels
    
    temp <- list()
    for(i in grid_x){
      temp[[paste('nrow',i,'ncol_0',sep='_')]] <- list(rowclust = cutree(inputdatx,i))
    }
    inputdatx <- temp
    
    dimx <- "ncol_0"
  }
  
  if(dendextend::is.hclust(inputdaty)){ # format y tree as list
    
    rownames_y <- inputdaty$labels
    
    temp <- list()
    for(i in grid_y){
      temp[[paste('nrow',i,'ncol_0',sep='_')]] <- list(rowclust = cutree(inputdaty,i))
    }
    inputdaty <- temp
    
    dimy <- "ncol_0"
  }
  
  
  ### Pre-process list inputs ###
  
  if(is.list(inputdatx)){
    
    temp <- names(inputdatx)[grep(paste('nrow_', grid_x[1], sep = ''), names(inputdatx))] 
    dimx <- sub(paste('nrow_', grid_x[1], '_', sep = ''), '', temp)
  
    rownames_x <- names(inputdatx[[temp[1]]]$rowclust)

    
  } 
  
  if(is.list(inputdaty)){
    
    temp <- names(inputdaty)[grep(paste('nrow_', grid_y[1], sep = ''), names(inputdaty))] 
    dimy <- sub(paste('nrow_', grid_y[1], '_', sep = ''), '', temp)
    
    rownames_y <- names(inputdaty[[temp[1]]]$rowclust)
    
  }
  
  
  
  ## initialize testing grid ##
  
  simout <- array(0, 
                  dim = c(length(grid_x), length(grid_y), 
                          length(dimx), length(dimy),
                          nperm),
                  dimnames = list(paste('Kx=', grid_x, sep=''),
                               paste('Ky=', grid_y, sep=''),
                               paste('XClust_', dimx, sep=''), 
                               paste('YClust_', dimy, sep=''), 
                               1:nperm)
                  )
  p_out <- simout
  
  mi_out <- array(0, 
                  dim = c(length(grid_x), length(grid_y), 
                          length(dimx), length(dimy) ),
                  dimnames = list(paste('Kx=', grid_x, sep=''),
                               paste('Ky=', grid_y, sep=''),
                               dimx, dimy)
                  )
  

  

  ## conduct sim ## 
  
  for(b in 1:nperm){
    
    # permute indices of tree (to be conserved across grid)
    
    permx <- data.frame(NewID = rownames_x, row.names = rownames_x)
    permx$NewID <- sample(rownames_x)
    
    permy <- data.frame(NewID = rownames_y, row.names = rownames_y)
    permy$NewID <- sample(rownames_y)
    
    
    
    for(i in 1:length(grid_x)){
      for(j in 1:length(grid_y)){
        
        index_x <- grep(paste('nrow_', grid_x[i], sep = ''),
                        names(inputdatx))
        index_y <- grep(paste('nrow_', grid_y[j], sep = ''),
                        names(inputdaty))
        
        # pull encoding values for dat x
        if(length(index_x) == 1){
          
          # pull encoding data frame from list
          datx <- as.data.frame(inputdatx[[index_x]]$rowclust)
          colnames(datx) <- names(inputdatx)[index_x]
          datx_obs <- datx
          
          # permute using the conserved tree permutation index
          tempdat <- merge(datx, permx, by = 0)
          tempdat$Row.names <- tempdat$NewID
          datx <- tempdat[ , -which(names(tempdat) == 'NewID') ]
          datx <- transform(datx,
                            row.names=Row.names, 
                            Row.names=NULL)
          
        }else{

          # initialize data frame
          datx <- as.data.frame(inputdatx[[index_x[1]]]$rowclust)
          colnames(datx) <- names(inputdatx)[index_x[1]]
          
          # merge additional columns 
          for(ix in 2:length(index_x)){
            temp <- as.data.frame(inputdatx[[index_x[ix]]]$rowclust)
            colnames(temp) <- names(inputdatx)[index_x[ix]]
            
            datx <- transform(merge(datx, temp, by = 0, all = T),
                              row.names=Row.names, Row.names=NULL)
          }
          datx_obs <- datx
          
          # permute using the conserved tree permutation index
          tempdat <- merge(datx, permx, by = 0)
          tempdat$Row.names <- tempdat$NewID
          datx <- tempdat[ , -which(names(tempdat) == 'NewID') ]
          datx <- transform(datx,
                            row.names=Row.names, 
                            Row.names=NULL)
          
        }
        
        
        # pull encoding values for dat y
        if(length(index_y) == 1){
          
          daty <- as.data.frame(inputdaty[[index_y]]$rowclust)
          colnames(daty) <- names(inputdaty)[index_y]
          daty_obs <- daty
          
          # permute using the conserved tree permutation index
          tempdat <- merge(daty, permy, by = 0)
          tempdat$Row.names <- tempdat$NewID
          daty <- tempdat[ , -which(names(tempdat) == 'NewID') ]
          daty <- transform(daty,
                            row.names=Row.names, 
                            Row.names=NULL)
          
        }else{
          
          # initialize data frame
          daty <- as.data.frame(inputdaty[[index_y[1]]]$rowclust)
          colnames(daty) <- names(inputdaty)[index_y[1]]
          
          # merge additional columns 
          for(iy in 2:length(index_y)){
            temp <- as.data.frame(inputdaty[[index_y[iy]]]$rowclust)
            colnames(temp) <- names(inputdaty)[index_y[iy]]
            
            daty <- transform(merge(daty, temp, by = 0, all = T),
                              row.names=Row.names, Row.names=NULL)
          }
          daty_obs <- daty
          
          # permute using the conserved tree permutation index
          tempdat <- merge(daty, permy, by = 0)
          tempdat$Row.names <- tempdat$NewID
          daty <- tempdat[ , -which(names(tempdat) == 'NewID') ]
          daty <- transform(daty,
                            row.names=Row.names, 
                            Row.names=NULL)
          
        }
        
        
        datall <- transform(merge(datx, daty, by = 0),
                            row.names=Row.names, Row.names=NULL)
        names(datall) <- c(paste('XCluster', names(datx)),
                               paste('YCluster',names(daty)))
        
        if(b == 1){
          datall_obs <- transform(merge(datx_obs, daty_obs, by = 0),
                            row.names=Row.names, Row.names=NULL)
          names(datall_obs) <- c(paste('XCluster', names(datx_obs)),
                               paste('YCluster',names(daty_obs)))
        }
        
        for(ci in 1:ncol(datx)){
          for(cj in (1:ncol(daty)) + ncol(datx) ){
            
            if(b == 1){
              entoutobs <- calcEntropy(table(datall_obs[,ci],
                                          datall_obs[,cj]),
                        logbase = logbase,
                        estimator = estimator)
              if(rescaleMI){
                mi_out[i,j,ci,cj-ncol(datx)] <- entoutobs$RelMI
              }else{
                mi_out[i,j,ci,cj-ncol(datx)] <- entoutobs$MutualInformation
              }
            }
            
            
           entout <- calcEntropy(table(datall[,ci],
                                       datall[,cj]),
                        logbase = logbase,
                        estimator = estimator)
           
           if(rescaleMI){
             simout[i,j,ci,cj-ncol(datx),b] <- entout$RelMI
           }else{
             simout[i,j,ci,cj-ncol(datx),b] <- entout$MutualInformation
           }
           
           
           p_out[i,j,ci,cj-ncol(datx),b] <- simout[i,j,ci,cj-ncol(datx),b] >= mi_out[i,j,ci,cj-ncol(datx)]
           
          }
        }
        
      }# for j
    }# for i
  }# for b
  
  # hist(simout[1,1,1,1,])
  # abline(v = mi_out[1,1,1,1], col = 'red')
  # sum(simout[1,1,1,1,] >= mi_out[1,1,1,1]) / length(simout[1,1,1,1,])
  
  
  
  ## Calculate Probabilities ##
  
  p_outp <- apply(p_out, c(1,2, 3, 4), 
                 function(x) sum(x)/dim(p_out)[5] )
  
  nullmed <- apply(simout, c(1,2,3,4), median)
  nullIQR <- apply(simout, c(1,2,3,4), IQR)
  
  z_out <- (mi_out - nullmed)/nullIQR
  
  plotout <- NULL
  if(verbose){
    
    annocol = NULL
    annorow = NULL
    mycolist = list()
    if(dim(mi_out)[3] > 1 | dim(mi_out)[4] > 1){
      
      temp2 <- array(NA,
                    dim = c(dim(z_out)[1], dim(z_out)[2], 2),
                    dimnames = list(dimnames(z_out)[[1]], 
                                    dimnames(z_out)[[2]],
                                    c('BestX','BestY')))
      for(t_kx in 1:dim(z_out)[1]){ 
        for(t_ky in 1:dim(z_out)[2]){ 
          
          temp <- z_out[t_kx, t_ky, , , drop = F]
          myindeces <- which(temp == max(temp), arr.ind = T)
          temp2[t_kx, t_ky, 'BestX']<-dimnames(z_out)[[3]][myindeces[3]]
          temp2[t_kx, t_ky, 'BestY']<-dimnames(z_out)[[4]][myindeces[4]]
       
        }
      }

      if(dim(mi_out)[3] > 1){
        
        if(dim(mi_out)[2] == 1){
          annorow <- as.data.frame(t(temp2[,,1]))
          names(annorow) <- dimnames(temp2)[[2]]
        }else{
          annorow <- as.data.frame(temp2[,,1])
        }
        
        
        for(i in 1:ncol(annorow)){
          annorow[,i] <- factor(annorow[,i], 
                                levels = dimnames(mi_out)[[3]], 
                                ordered = T)
          mycolist[as.character(names(annorow)[i])] <- list(setNames(brewer.pal(dim(mi_out)[3], "Greens"),dimnames(mi_out)[[3]]))
        } 
      }
      
      if(dim(mi_out)[4] > 1){
        
        if(dim(mi_out)[1] == 1){
          annocol <- as.data.frame(temp2[,,2])
          names(annocol) <- dimnames(temp2)[[1]]
        }else{
          annocol <- as.data.frame(t(temp2[,,2]))
        }
        
        
        for(i in 1:ncol(annocol)){
          annocol[,i] <- factor(annocol[,i], 
                                levels = dimnames(mi_out)[[4]], 
                                ordered = T)
          mycolist[as.character(names(annocol)[i])] <- list(setNames(brewer.pal(dim(mi_out)[4], "Purples"),dimnames(mi_out)[[4]]))
        } 
        #str(annocol)
      }
      
    }
    
    
    plotout <- pheatmap::pheatmap(apply(z_out, c(1,2), max),
                       cluster_rows = F, cluster_cols = F,
                       main = 'Simulated ZScore Estimates of Observed Relative Entropy Estimates',
                       annotation_col = annocol,
                       annotation_row = annorow,
                       annotation_colors = mycolist)  
    plotout
  }
  
  
  
  return(list(PVals = p_outp, 
              RelMIVals = mi_out,
              ZScores = z_out,
              PlotOut = plotout))  
  
}

# temp <- bivarEncodeTest(encodeout_otb_kld$nrow_1_ncol_1$rowtree,
#                     dmgridout$nrow_6_ncol_4$rowtree,
#                     grid_x = 2:10, grid_y = 2:10)
# temp$PVals
# 
# 
# temp2 <- bivarEncodeTest(encodeout_otb_kld,
#                     dmgridout,
#                     grid_x = 2:10, grid_y = 2:10)
# temp2$PVals
```

And finally I want to use my helper function to find the overall best row and column from the Pval matrix to select a row and cluster number combo to go visualize

```
getBVTPval <- function(gridout){
  
  rankinrow <- t(apply(gridout, 1, rank))
  avgcolrank <- apply(rankinrow, 2, mean)
  mincol <- which.min(avgcolrank)
  
  rankincol <- apply(gridout, 2, rank)
  avgrowrank <- apply(rankincol, 1, mean)
  minrow <- which.min(avgrowrank)
  
  return(list(pval = gridout[minrow, mincol], row = minrow, col = mincol))
  
}


#getBVTPval(temp2$PVals)
```

## Bivar Test - OTB vs Cow Attributes

First I need to create a joint encoding for age and peak milk yeild fields.

```
cowageyield <- cowattribdat[,c("AgeDaysOld", "MilkYield95")]
cowageyield <- as.data.frame(apply(cowageyield, 2, scale))
rownames(cowageyield) <- cowattribdat$CowID
cowageyield <- cowageyield[ !is.na(cowageyield$AgeDaysOld) |
                              !is.na(cowageyield$MilkYield95) , ]

encodeout_ageyeild_all <-  encodeplot(cowageyield, 
                       dist(cowageyield), 
                       dist(t(cowageyield)),
                       n_rowclusters = 1:10, 
                       n_colclusters = 0,
                       plot_title = 'Encoding of Cow Age & Productivity: All Cows',
                       showplot = F, 
                       exportplot = T,
                       imwidth = 12,
                       imheight = 24,
                       imres = 300,
                       filename = 'Viz/OverallTB/BivarTest_AgeYeild/AgeYeildEncoding/All/AgeYeild'
                       )

temp <- cowageyield[rownames(cowageyield) %in%
                 names(dmgridout_healthy$nrow_2_ncol_1$rowclust), ]
encodeout_ageyeild_h <-  encodeplot(temp,
                       dist(temp), 
                       dist(t(temp)),
                       n_rowclusters = 1:10, 
                       n_colclusters = 0,
                       plot_title = 'Encoding of Cow Age & Productivity: Healthy Cows',
                       showplot = F, 
                       exportplot = T,
                       filename = 'Viz/OverallTB/BivarTest_AgeYeild/AgeYeildEncoding/Healthy/AgeYeild',
                       imwidth = 12,
                       imheight = 20,
                       imres = 300
                       )
```

### Noise Penalized

#### Healthy Cows

First I want to look at just the healthy cows

```
set.seed(61916)

bvt_ay_ew <- bivarEncodeTest(encodeout_ageyeild_h,
                    EncodingsOTB$Ensemble,
                    grid_x = 2:10, grid_y = 2:10,
                    estimator = 'shrink',
                    rescaleMI = T,
                    verbose = T)
```

```
temp <- bvt_ay_ew
round(temp$PVals,3)
```

```
## , , XClust_ncol_0, YClust_ncol_0
## 
##        Ky=2  Ky=3  Ky=4  Ky=5  Ky=6  Ky=7  Ky=8  Ky=9 Ky=10
## Kx=2  0.625 0.911 0.488 0.336 0.370 0.317 0.413 0.507 0.465
## Kx=3  0.336 0.473 0.375 0.161 0.163 0.062 0.064 0.099 0.097
## Kx=4  0.271 0.544 0.556 0.380 0.327 0.088 0.111 0.090 0.069
## Kx=5  0.215 0.595 0.474 0.217 0.110 0.023 0.036 0.047 0.028
## Kx=6  0.537 0.402 0.307 0.148 0.079 0.017 0.036 0.036 0.016
## Kx=7  0.558 0.219 0.197 0.099 0.075 0.033 0.032 0.036 0.018
## Kx=8  0.217 0.127 0.150 0.094 0.061 0.039 0.042 0.056 0.026
## Kx=9  0.234 0.163 0.229 0.144 0.101 0.052 0.060 0.078 0.037
## Kx=10 0.349 0.239 0.337 0.265 0.186 0.093 0.074 0.132 0.069
```

```
set.seed(61916)

compareEncodings(encodeout_ageyeild_h$nrow_6_ncol_0,
                 EncodingsOTB$Ensemble$nrow_7_ncol_0,
                 colorby = 'PMI',
                 simPMI = 'NullParam',
                 alpha = 0.05,
                 nsim = 2000,
                 gradient_color_low = 'steelblue',
                 gradient_color_high = 'orange',
                 filename = 'Viz/OverallTB/BivarTest_AgeYeild/OTB_Healthy_NP',
                 fontsize = 30,
                 imheight = 38,
                 imwidth = 30)
```

```
## file saved to Viz/OverallTB/BivarTest_AgeYeild/OTB_Healthy_NP.pdf
```

High yeilding parity 2 cows are over-represented in the time budget characterized by relatively low time spent eating, relatively high time nonactive, and relatively high time ruminating.

Low yeilding parity 2 cows are over-represented in the time budget cluster characterized by extremely low time spent eating, high time ruminating, and relatively high time spent nonactive

Heifers were under-represented in the time budget cluster characterized by moderate time spent eating, low time spent nonactive, and relatively high time spent ruminating.

#### All Cows

```
set.seed(61916)

bvt_ay_ew_all <- bivarEncodeTest(encodeout_ageyeild_all,
                    EncodingsOTB$Ensemble, 
                    grid_x = 2:10, grid_y = 2:10,
                    estimator = 'shrink',
                    rescaleMI = T, 
                    verbose = T)
```

```
temp <- bvt_ay_ew_all
round(temp$PVals,3)
```

```
## , , XClust_ncol_0, YClust_ncol_0
## 
##        Ky=2  Ky=3  Ky=4  Ky=5  Ky=6  Ky=7  Ky=8  Ky=9 Ky=10
## Kx=2  0.239 0.045 0.135 0.211 0.287 0.168 0.198 0.249 0.281
## Kx=3  0.282 0.153 0.381 0.541 0.698 0.293 0.129 0.187 0.252
## Kx=4  0.238 0.191 0.317 0.570 0.698 0.365 0.229 0.290 0.397
## Kx=5  0.313 0.159 0.344 0.483 0.598 0.328 0.177 0.249 0.424
## Kx=6  0.414 0.248 0.520 0.670 0.778 0.551 0.388 0.510 0.692
## Kx=7  0.166 0.140 0.455 0.651 0.708 0.447 0.286 0.379 0.568
## Kx=8  0.143 0.234 0.533 0.685 0.800 0.655 0.453 0.538 0.667
## Kx=9  0.059 0.100 0.367 0.562 0.765 0.594 0.432 0.467 0.561
## Kx=10 0.149 0.165 0.568 0.654 0.795 0.632 0.534 0.578 0.640
```

```
#round(temp$RelMIVals,3)
#getBVTPval(bvt_ay_ew_all$PVals)
```

A weakly significant (margial) result.

```
set.seed(61916)


compareEncodings(encodeout_ageyeild_all$nrow_2_ncol_0,
                 EncodingsOTB$Ensemble$nrow_3_ncol_0,
                 colorby = 'PMI',
                 simPMI = 'NullParam',
                 alpha = 0.05,
                 nsim = 2000,
                 gradient_color_low = 'steelblue',
                 gradient_color_high = 'orange',
                 filename = 'Viz/OverallTB/BivarTest_AgeYeild/OTB_All_NP',
                 fontsize = 30,
                 imheight = 38,
                 imwidth = 30)
```

```
## file saved to Viz/OverallTB/BivarTest_AgeYeild/OTB_All_NP.pdf
```

Heifers are signficantly under-represented in the time budget cluster characterized by relatively low time spent eating, relatively high time nonactive, and relatively high time ruminating.

### Plasticity Penalized

#### Healthy

First I want to look at just the healthy cows

```
set.seed(61916)

bvt_ay_pw <- bivarEncodeTest(encodeout_ageyeild_h,
                    EncodingsOTB$Plasticity,
                    grid_x = 2:10, grid_y = 2:10,
                    estimator = 'shrink',
                    rescaleMI = T, 
                    verbose = T)
```

```
temp <- bvt_ay_pw
round(temp$PVals,3)
```

```
## , , XClust_ncol_0, YClust_ncol_0
## 
##        Ky=2  Ky=3  Ky=4  Ky=5  Ky=6  Ky=7  Ky=8  Ky=9 Ky=10
## Kx=2  0.251 0.422 0.086 0.261 0.392 0.377 0.366 0.519 0.832
## Kx=3  0.138 0.237 0.080 0.303 0.160 0.114 0.160 0.139 0.267
## Kx=4  0.376 0.339 0.257 0.499 0.296 0.254 0.258 0.162 0.308
## Kx=5  0.330 0.252 0.336 0.660 0.344 0.302 0.199 0.139 0.315
## Kx=6  0.269 0.462 0.155 0.460 0.167 0.124 0.122 0.104 0.410
## Kx=7  0.173 0.391 0.089 0.389 0.275 0.204 0.086 0.089 0.320
## Kx=8  0.224 0.271 0.078 0.324 0.259 0.193 0.085 0.081 0.324
## Kx=9  0.384 0.375 0.132 0.383 0.298 0.231 0.128 0.110 0.321
## Kx=10 0.534 0.556 0.281 0.596 0.413 0.336 0.176 0.138 0.372
```

```
#round(temp$RelMIVals,3)

#getBVTPval(bvt_ay_pw$PVals)
```

No significant result.

#### All

```
set.seed(61916)

bvt_ay_pw_all <- bivarEncodeTest(encodeout_ageyeild_all,
                    EncodingsOTB$Plasticity,
                    grid_x = 2:10, grid_y = 2:10, 
                    estimator = 'shrink', 
                    rescaleMI = T,
                    verbose = T)
```

```
temp <- bvt_ay_pw_all
round(temp$PVals,3)
```

```
## , , XClust_ncol_0, YClust_ncol_0
## 
##        Ky=2  Ky=3  Ky=4  Ky=5  Ky=6  Ky=7  Ky=8  Ky=9 Ky=10
## Kx=2  0.216 0.216 0.404 0.524 0.304 0.278 0.351 0.188 0.103
## Kx=3  0.389 0.441 0.723 0.845 0.728 0.666 0.722 0.452 0.338
## Kx=4  0.197 0.236 0.555 0.717 0.598 0.587 0.618 0.295 0.223
## Kx=5  0.241 0.369 0.344 0.562 0.470 0.482 0.527 0.438 0.476
## Kx=6  0.375 0.580 0.534 0.805 0.756 0.787 0.924 0.866 0.921
## Kx=7  0.517 0.426 0.460 0.760 0.668 0.689 0.870 0.750 0.802
## Kx=8  0.520 0.351 0.354 0.620 0.584 0.841 0.890 0.743 0.659
## Kx=9  0.442 0.178 0.260 0.489 0.455 0.709 0.754 0.538 0.480
## Kx=10 0.532 0.389 0.435 0.758 0.727 0.831 0.896 0.816 0.834
```

```
#round(temp$RelMIVals,3)

#getBVTPval(bvt_ay_pw_all$PVals)
```

No significant association.

## Bivar Test - Entry Position vs Cow Attributes

#### Healthy

```
set.seed(61916)

bvt_ay_eo_h <- bivarEncodeTest(encodeout_ageyeild_h,
                    dmgridout_healthy,
                    grid_x = 2:10, grid_y = 2:10, 
                    estimator = 'shrink',
                    rescaleMI = T,
                    verbose = T)
```

```
bvt_ay_eo_h$PVals
```

```
## , , XClust_ncol_0, YClust_ncol_1
## 
##        Ky=2  Ky=3  Ky=4  Ky=5  Ky=6  Ky=7  Ky=8  Ky=9 Ky=10
## Kx=2  1.000 1.000 0.637 0.753 1.000 0.891 0.466 0.463 0.508
## Kx=3  0.477 1.000 0.761 0.886 1.000 0.950 0.771 0.832 0.646
## Kx=4  0.178 0.676 0.417 0.533 0.708 0.558 0.550 0.706 0.550
## Kx=5  0.109 0.417 0.399 0.434 0.677 0.630 0.611 0.513 0.518
## Kx=6  0.253 0.619 0.604 0.590 0.631 0.444 0.434 0.394 0.420
## Kx=7  0.236 0.370 0.426 0.615 0.536 0.301 0.302 0.283 0.156
## Kx=8  0.381 0.460 0.591 0.745 0.685 0.420 0.395 0.308 0.185
## Kx=9  0.251 0.345 0.504 0.661 0.627 0.386 0.371 0.276 0.173
## Kx=10 0.293 0.440 0.495 0.713 0.566 0.366 0.264 0.210 0.121
## 
## , , XClust_ncol_0, YClust_ncol_2
## 
##        Ky=2  Ky=3  Ky=4  Ky=5  Ky=6  Ky=7  Ky=8  Ky=9 Ky=10
## Kx=2  0.861 0.559 0.576 0.745 1.000 0.610 0.945 0.634 0.717
## Kx=3  0.610 0.973 0.959 0.999 1.000 0.608 0.750 0.527 0.415
## Kx=4  0.326 0.643 0.608 0.738 0.749 0.321 0.574 0.319 0.376
## Kx=5  0.167 0.479 0.576 0.640 0.610 0.372 0.556 0.243 0.388
## Kx=6  0.250 0.518 0.563 0.627 0.453 0.113 0.495 0.261 0.199
## Kx=7  0.236 0.673 0.391 0.767 0.283 0.055 0.372 0.166 0.134
## Kx=8  0.431 0.663 0.455 0.810 0.386 0.089 0.395 0.245 0.160
## Kx=9  0.286 0.628 0.401 0.759 0.327 0.069 0.352 0.204 0.146
## Kx=10 0.313 0.689 0.366 0.767 0.345 0.103 0.382 0.238 0.207
## 
## , , XClust_ncol_0, YClust_ncol_3
## 
##        Ky=2  Ky=3  Ky=4  Ky=5  Ky=6  Ky=7  Ky=8  Ky=9 Ky=10
## Kx=2  0.691 0.519 0.588 0.640 0.811 0.785 0.954 0.983 0.729
## Kx=3  1.000 0.704 0.819 0.855 0.794 0.637 0.947 0.987 0.650
## Kx=4  0.959 0.236 0.496 0.583 0.295 0.259 0.851 0.913 0.558
## Kx=5  0.369 0.104 0.531 0.532 0.401 0.193 0.878 0.873 0.386
## Kx=6  0.440 0.183 0.653 0.592 0.353 0.103 0.900 0.737 0.194
## Kx=7  0.588 0.265 0.592 0.702 0.327 0.119 0.829 0.603 0.121
## Kx=8  0.502 0.487 0.780 0.822 0.415 0.247 0.862 0.658 0.143
## Kx=9  0.463 0.390 0.636 0.783 0.351 0.226 0.832 0.559 0.142
## Kx=10 0.614 0.408 0.675 0.840 0.563 0.311 0.845 0.679 0.176
## 
## , , XClust_ncol_0, YClust_ncol_4
## 
##        Ky=2  Ky=3  Ky=4  Ky=5  Ky=6  Ky=7  Ky=8  Ky=9 Ky=10
## Kx=2  0.691 0.570 0.617 0.729 0.815 0.651 0.770 0.878 0.887
## Kx=3  1.000 0.951 0.937 1.000 0.871 0.909 1.000 0.835 0.945
## Kx=4  0.959 0.296 0.549 0.764 0.389 0.397 0.484 0.685 0.985
## Kx=5  0.369 0.221 0.474 0.535 0.535 0.411 0.452 0.494 0.941
## Kx=6  0.440 0.173 0.556 0.505 0.432 0.222 0.241 0.335 0.851
## Kx=7  0.588 0.254 0.579 0.545 0.383 0.168 0.236 0.329 0.571
## Kx=8  0.502 0.253 0.662 0.690 0.470 0.153 0.343 0.352 0.560
## Kx=9  0.463 0.191 0.611 0.637 0.390 0.134 0.311 0.301 0.544
## Kx=10 0.614 0.197 0.642 0.735 0.542 0.254 0.344 0.290 0.631
## 
## , , XClust_ncol_0, YClust_ncol_5
## 
##        Ky=2  Ky=3  Ky=4  Ky=5  Ky=6  Ky=7  Ky=8  Ky=9 Ky=10
## Kx=2  0.691 0.588 0.664 0.351 0.708 0.291 0.962 0.430 0.916
## Kx=3  1.000 0.943 0.503 0.788 0.823 0.410 0.931 0.857 0.175
## Kx=4  0.959 0.406 0.390 0.508 0.811 0.184 0.890 0.652 0.157
## Kx=5  0.369 0.285 0.197 0.487 0.581 0.314 0.899 0.567 0.101
## Kx=6  0.440 0.356 0.505 0.504 0.528 0.065 0.904 0.402 0.100
## Kx=7  0.588 0.478 0.358 0.638 0.538 0.085 0.786 0.361 0.139
## Kx=8  0.502 0.506 0.365 0.696 0.605 0.149 0.832 0.233 0.147
## Kx=9  0.463 0.427 0.300 0.645 0.594 0.121 0.764 0.187 0.094
## Kx=10 0.614 0.505 0.408 0.739 0.632 0.178 0.738 0.265 0.177
## 
## , , XClust_ncol_0, YClust_ncol_6
## 
##        Ky=2  Ky=3  Ky=4  Ky=5  Ky=6  Ky=7  Ky=8  Ky=9 Ky=10
## Kx=2  1.000 0.642 0.635 0.759 1.000 0.868 0.756 0.855 0.923
## Kx=3  0.562 0.890 0.862 0.983 1.000 0.601 0.746 0.694 0.932
## Kx=4  0.275 0.937 0.573 0.793 0.790 0.285 0.513 0.727 0.921
## Kx=5  0.332 0.451 0.493 0.420 0.730 0.362 0.313 0.560 0.912
## Kx=6  0.389 0.541 0.506 0.347 0.705 0.385 0.254 0.350 0.528
## Kx=7  0.382 0.410 0.394 0.433 0.572 0.268 0.146 0.298 0.452
## Kx=8  0.596 0.332 0.503 0.335 0.718 0.407 0.219 0.277 0.634
## Kx=9  0.592 0.259 0.395 0.296 0.604 0.341 0.211 0.250 0.623
## Kx=10 0.619 0.338 0.406 0.535 0.579 0.376 0.263 0.224 0.594
```

```
#getBVTPval(bvt_ay_eo_h$PVals)
```

```
set.seed(61916)


compareEncodings(encodeout_ageyeild_all$nrow_7_ncol_0,
                 dmgridout_healthy$nrow_7_ncol_5,
                 colorby = 'PMI',
                 simPMI = 'NullParam',
                 alpha = 0.05,
                 nsim = 2000,
                 gradient_color_low = 'steelblue',
                 gradient_color_high = 'orange',
                 filename = 'Viz/OverallTB/BivarTest_AgeYeild/EO_Healthy',
                 fontsize = 30,
                 imheight = 38,
                 imwidth = 30)
```

```
## file saved to Viz/OverallTB/BivarTest_AgeYeild/EO_Healthy.pdf
```

The higher yeilding older cows were over-represented at the very front of the queue.

#### All

```
set.seed(61916)

bvt_ay_eo_all <- bivarEncodeTest(encodeout_ageyeild_all,
                    dmgridout_all,
                    grid_x = 2:10, grid_y = 2:10, 
                    estimator = 'shrink',
                    rescaleMI = T,
                    verbose = T)
```

```
temp <- bvt_ay_eo_all
round(temp$PVals,3)
```

```
## , , XClust_ncol_0, YClust_ncol_1
## 
##        Ky=2  Ky=3  Ky=4  Ky=5  Ky=6  Ky=7  Ky=8  Ky=9 Ky=10
## Kx=2  0.409 0.576 0.439 0.530 0.145 0.295 0.396 0.349 0.332
## Kx=3  0.317 0.605 0.673 0.659 0.260 0.368 0.414 0.445 0.545
## Kx=4  0.054 0.078 0.152 0.224 0.095 0.150 0.200 0.232 0.232
## Kx=5  0.169 0.187 0.346 0.364 0.160 0.168 0.168 0.220 0.276
## Kx=6  0.279 0.489 0.632 0.685 0.459 0.560 0.521 0.611 0.709
## Kx=7  0.332 0.711 0.706 0.854 0.701 0.657 0.600 0.697 0.814
## Kx=8  0.379 0.758 0.754 0.882 0.568 0.734 0.757 0.785 0.639
## Kx=9  0.255 0.700 0.667 0.832 0.416 0.476 0.487 0.509 0.373
## Kx=10 0.501 0.762 0.786 0.860 0.633 0.678 0.653 0.595 0.485
## 
## , , XClust_ncol_0, YClust_ncol_2
## 
##        Ky=2  Ky=3  Ky=4  Ky=5  Ky=6  Ky=7  Ky=8  Ky=9 Ky=10
## Kx=2  0.734 0.777 0.948 0.612 0.115 0.339 0.208 0.396 0.473
## Kx=3  0.887 0.762 0.998 0.792 0.392 0.528 0.484 0.473 0.677
## Kx=4  0.398 0.205 0.270 0.290 0.011 0.027 0.246 0.159 0.213
## Kx=5  0.604 0.405 0.345 0.444 0.018 0.022 0.232 0.281 0.195
## Kx=6  0.767 0.718 0.646 0.785 0.126 0.172 0.680 0.737 0.643
## Kx=7  0.820 0.886 0.692 0.909 0.306 0.476 0.857 0.960 0.811
## Kx=8  0.884 0.899 0.807 0.916 0.214 0.656 0.784 0.926 0.683
## Kx=9  0.724 0.925 0.489 0.888 0.157 0.637 0.607 0.955 0.635
## Kx=10 0.874 0.910 0.599 0.927 0.288 0.608 0.748 0.940 0.947
## 
## , , XClust_ncol_0, YClust_ncol_3
## 
##        Ky=2  Ky=3  Ky=4  Ky=5  Ky=6  Ky=7  Ky=8  Ky=9 Ky=10
## Kx=2  0.872 1.000 0.905 1.000 0.359 0.453 0.102 0.278 0.183
## Kx=3  0.935 0.918 0.935 1.000 0.248 0.724 0.102 0.281 0.180
## Kx=4  0.452 0.471 0.265 0.642 0.070 0.177 0.063 0.226 0.161
## Kx=5  0.649 0.552 0.328 0.736 0.082 0.303 0.067 0.091 0.250
## Kx=6  0.793 0.767 0.577 0.955 0.249 0.781 0.306 0.468 0.674
## Kx=7  0.804 0.906 0.722 0.976 0.403 0.866 0.654 0.692 0.849
## Kx=8  0.873 0.928 0.881 0.984 0.534 0.775 0.671 0.718 0.682
## Kx=9  0.675 0.843 0.849 0.969 0.452 0.692 0.553 0.537 0.682
## Kx=10 0.859 0.605 0.750 0.962 0.472 0.937 0.512 0.421 0.729
## 
## , , XClust_ncol_0, YClust_ncol_4
## 
##        Ky=2  Ky=3  Ky=4  Ky=5  Ky=6  Ky=7  Ky=8  Ky=9 Ky=10
## Kx=2  0.552 0.468 0.384 0.738 0.345 0.807 0.230 0.271 0.039
## Kx=3  0.715 0.644 0.600 0.836 0.488 0.835 0.529 0.482 0.099
## Kx=4  0.077 0.237 0.205 0.326 0.227 0.436 0.095 0.172 0.030
## Kx=5  0.104 0.352 0.418 0.469 0.250 0.481 0.071 0.239 0.159
## Kx=6  0.194 0.628 0.694 0.829 0.564 0.891 0.368 0.627 0.519
## Kx=7  0.405 0.888 0.664 0.915 0.704 0.911 0.585 0.939 0.761
## Kx=8  0.341 0.854 0.785 0.918 0.743 0.945 0.642 0.905 0.630
## Kx=9  0.501 0.938 0.676 0.896 0.605 0.920 0.663 0.951 0.443
## Kx=10 0.205 0.970 0.867 0.887 0.846 0.914 0.801 0.984 0.619
## 
## , , XClust_ncol_0, YClust_ncol_5
## 
##        Ky=2  Ky=3  Ky=4  Ky=5  Ky=6  Ky=7  Ky=8  Ky=9 Ky=10
## Kx=2  0.718 0.992 0.963 0.751 0.411 0.712 0.288 0.074 0.029
## Kx=3  0.842 0.947 0.987 0.765 0.582 0.896 0.466 0.272 0.075
## Kx=4  0.201 0.153 0.170 0.172 0.145 0.255 0.168 0.109 0.020
## Kx=5  0.357 0.247 0.336 0.269 0.257 0.244 0.271 0.039 0.020
## Kx=6  0.516 0.545 0.566 0.670 0.587 0.601 0.669 0.230 0.174
## Kx=7  0.609 0.754 0.781 0.852 0.729 0.747 0.786 0.562 0.471
## Kx=8  0.687 0.815 0.892 0.948 0.746 0.576 0.851 0.662 0.697
## Kx=9  0.506 0.641 0.896 0.888 0.559 0.507 0.813 0.598 0.730
## Kx=10 0.693 0.631 0.911 0.905 0.438 0.787 0.894 0.623 0.716
## 
## , , XClust_ncol_0, YClust_ncol_6
## 
##        Ky=2  Ky=3  Ky=4  Ky=5  Ky=6  Ky=7  Ky=8  Ky=9 Ky=10
## Kx=2  0.397 0.928 0.762 0.697 0.090 0.150 0.355 0.022 0.203
## Kx=3  0.640 0.681 0.903 0.946 0.225 0.451 0.472 0.078 0.220
## Kx=4  0.076 0.221 0.126 0.352 0.042 0.200 0.045 0.044 0.052
## Kx=5  0.097 0.321 0.269 0.597 0.186 0.310 0.056 0.124 0.094
## Kx=6  0.167 0.530 0.511 0.934 0.552 0.769 0.389 0.499 0.365
## Kx=7  0.326 0.739 0.749 0.978 0.788 0.848 0.765 0.727 0.682
## Kx=8  0.453 0.869 0.777 0.990 0.742 0.930 0.786 0.812 0.640
## Kx=9  0.649 0.659 0.811 0.983 0.634 0.885 0.853 0.729 0.511
## Kx=10 0.198 0.737 0.782 0.953 0.674 0.909 0.890 0.662 0.509
```

```
#getBVTPval(bvt_ay_eo_all$PVals)
```

```
set.seed(61916)

#View(bvt_ay_eo_all$TestResults$XCluster5_YCluster6) #C2

compareEncodings(encodeout_ageyeild_all$nrow_4_ncol_0,
                 dmgridout_all$nrow_6_ncol_2,
                 colorby = 'PMI',
                 simPMI = 'NullParam',
                 nsim = 2000,
                 alpha = 0.05,
                 gradient_color_low = 'steelblue',
                 gradient_color_high = 'orange',
                 filename = 'Viz/OverallTB/BivarTest_AgeYeild/EO_All',
                 fontsize = 30,
                 imheight = 38,
                 imwidth = 30)
```

```
## file saved to Viz/OverallTB/BivarTest_AgeYeild/EO_All.pdf
```

The parity 2 cows are over-represented at the very end of the queue.

The oldest cows (parity 3+) were over represented both at the very front of the queue, and amongst cows who frequented the center of the queue but also frequently jumped nearer the front and the rear.

## Bivar Tests - OTB vs Heatlh Status

### Noise Penalized

```
set.seed(61916)

sickcode <- data.frame(Sick = cowattribdat$Sick_Prepasture)
rownames(sickcode) <- cowattribdat$CowID

bvt_sickpen_ew <- bivarEncodeTest(sickcode,
                    EncodingsOTB$Ensemble,
                    grid_y = 2:10, 
                    estimator = 'shrink',
                    rescaleMI = T,
                    verbose = T)
```

```
set.seed(61916)

sickcode <- data.frame(Sick = cowattribdat$Sick_Prepasture)
rownames(sickcode) <- cowattribdat$CowID

compareEncodings(sickcode,
                 EncodingsOTB$Ensemble$nrow_7_ncol_0,
                 colorby = 'PMI',
                 simPMI = 'NullParam',
                 nsim = 2000,
                 alpha = 0.05,
                 gradient_color_low = 'steelblue',
                 gradient_color_high = 'orange',
                 filename = 'Viz/OverallTB/BivarTest_Sick/OTB_Health_NP',
                 fontsize = 30,
                 imheight = 38,
                 imwidth = 30)
```

```
## file saved to Viz/OverallTB/BivarTest_Sick/OTB_Health_NP.pdf
```

Sick cows are over-represtented in the clusters with relatively low (but not extremely low) time spent eating, and are under-represented in the cluster with the most moderate time budgets

### Plasticity Weighted

```
set.seed(61916)

sickcode <- data.frame(Sick = cowattribdat$Sick_Prepasture)
rownames(sickcode) <- cowattribdat$CowID

bvt_sickpen_pw <- bivarEncodeTest(sickcode,
                    EncodingsOTB$Plasticity,
                    grid_y = 2:10, estimator = 'shrink',
                    rescaleMI = T,
                    verbose = T)
```

```
bvt_sickpen_pw$PVals
```

```
## , , XClust_ncol_0, YClust_ncol_0
## 
##       Ky=2 Ky=3  Ky=4  Ky=5  Ky=6  Ky=7  Ky=8  Ky=9 Ky=10
## Kx=2 0.056 0.01 0.018 0.036 0.051 0.018 0.028 0.025 0.047
```

```
set.seed(61916)

sickcode <- data.frame(Sick = cowattribdat$Sick_Prepasture)
rownames(sickcode) <- cowattribdat$CowID

compareEncodings(sickcode,
                 EncodingsOTB$Plasticity$nrow_9_ncol_0,
                 colorby = 'PMI',
                 simPMI = 'NullParam',
                 alpha = 0.05,
                 nsim = 2000,
                 gradient_color_low = 'steelblue',
                 gradient_color_high = 'orange',
                 filename = 'Viz/OverallTB/BivarTest_Sick/OTB_Health_PP',
                 fontsize = 30,
                 imheight = 38,
                 imwidth = 30)
```

```
## file saved to Viz/OverallTB/BivarTest_Sick/OTB_Health_PP.pdf
```

Sick cows are again over-represented in the relatively low (but not extremely low) eating time cluster, and are under-represented in the cluster characterized by high time spent ruminating with moderate time invested in the other axes.

## Bivar Tests - Entry Order vs Health Status

### Health Status Pen

```
set.seed(61916)

sickcode <- data.frame(Sick = cowattribdat$Sick_Prepasture)
rownames(sickcode) <- cowattribdat$CowID

bvt_sickpen_eo <- bivarEncodeTest(sickcode,
                    dmgridout_all,
                    grid_y = 2:10, estimator = 'shrink',
                    rescaleMI = T, verbose = T)
```

```
bvt_sickpen_eo$PVals
```

```
## , , XClust_ncol_0, YClust_ncol_1
## 
##       Ky=2  Ky=3 Ky=4  Ky=5  Ky=6 Ky=7  Ky=8  Ky=9 Ky=10
## Kx=2 0.699 0.565 0.36 0.417 0.301  0.5 0.632 0.661 0.613
## 
## , , XClust_ncol_0, YClust_ncol_2
## 
##       Ky=2  Ky=3  Ky=4  Ky=5  Ky=6  Ky=7 Ky=8  Ky=9 Ky=10
## Kx=2 0.466 0.485 0.559 0.416 0.404 0.643 0.55 0.285 0.608
## 
## , , XClust_ncol_0, YClust_ncol_3
## 
##       Ky=2 Ky=3  Ky=4 Ky=5  Ky=6  Ky=7  Ky=8  Ky=9 Ky=10
## Kx=2 0.586 0.56 0.284 0.41 0.408 0.408 0.292 0.209 0.762
## 
## , , XClust_ncol_0, YClust_ncol_4
## 
##       Ky=2 Ky=3 Ky=4  Ky=5  Ky=6  Ky=7  Ky=8  Ky=9 Ky=10
## Kx=2 0.183 0.11 0.05 0.367 0.306 0.571 0.633 0.445 0.362
## 
## , , XClust_ncol_0, YClust_ncol_5
## 
##       Ky=2  Ky=3  Ky=4  Ky=5 Ky=6 Ky=7  Ky=8  Ky=9 Ky=10
## Kx=2 0.802 0.248 0.274 0.569  0.4 0.42 0.432 0.403  0.57
## 
## , , XClust_ncol_0, YClust_ncol_6
## 
##       Ky=2  Ky=3  Ky=4  Ky=5  Ky=6  Ky=7  Ky=8  Ky=9 Ky=10
## Kx=2 0.734 0.638 0.197 0.439 0.288 0.219 0.696 0.825 0.594
```

A weakly significant (marginal) result.

```
set.seed(61916)

sickcode <- data.frame(Sick = cowattribdat$Sick_Prepasture)
rownames(sickcode) <- cowattribdat$CowID

compareEncodings(sickcode,
                 dmgridout_all$nrow_4_ncol_4,
                 colorby = 'PMI',
                 simPMI = 'NullParam',
                 alpha = 0.05,
                 nsim = 2000,
                 gradient_color_low = 'steelblue',
                 gradient_color_high = 'orange',
                 filename = 'Viz/OverallTB/BivarTest_Sick/EO_Health',
                 fontsize = 30,
                 imheight = 38,
                 imwidth = 30)
```

```
## file saved to Viz/OverallTB/BivarTest_Sick/EO_Health.pdf
```

The marginally significant result revealed that sick cows were over-represented among cows that were consistently at the very end and nearer the end of the queue.

### Health Status Pasture

```
set.seed(61916)

sickcode <- data.frame(Sick = cowattribdat$Sick_Pasture)
rownames(sickcode) <- cowattribdat$CowID

bvt_sickpast_eo <- bivarEncodeTest(sickcode,
                    dmgridout_all,
                    grid_y = 2:10, estimator = 'shrink',
                    rescaleMI = T, verbose = T)
```

```
bvt_sickpast_eo$PVals
```

```
## , , XClust_ncol_0, YClust_ncol_1
## 
##      Ky=2  Ky=3  Ky=4  Ky=5  Ky=6  Ky=7  Ky=8  Ky=9 Ky=10
## Kx=2 0.45 0.684 0.379 0.294 0.334 0.398 0.367 0.231 0.453
## 
## , , XClust_ncol_0, YClust_ncol_2
## 
##       Ky=2  Ky=3  Ky=4  Ky=5  Ky=6  Ky=7  Ky=8  Ky=9 Ky=10
## Kx=2 0.484 0.627 0.449 0.582 0.519 0.383 0.404 0.326 0.516
## 
## , , XClust_ncol_0, YClust_ncol_3
## 
##       Ky=2  Ky=3  Ky=4  Ky=5  Ky=6  Ky=7  Ky=8  Ky=9 Ky=10
## Kx=2 0.499 0.107 0.476 0.103 0.798 0.273 0.692 0.264 0.396
## 
## , , XClust_ncol_0, YClust_ncol_4
## 
##       Ky=2  Ky=3  Ky=4  Ky=5  Ky=6  Ky=7  Ky=8  Ky=9 Ky=10
## Kx=2 0.981 0.644 0.937 0.379 0.648 0.322 0.981 0.396 0.335
## 
## , , XClust_ncol_0, YClust_ncol_5
## 
##       Ky=2  Ky=3  Ky=4  Ky=5 Ky=6  Ky=7  Ky=8  Ky=9 Ky=10
## Kx=2 0.419 0.896 0.292 0.436 0.31 0.161 0.218 0.594 0.299
## 
## , , XClust_ncol_0, YClust_ncol_6
## 
##      Ky=2  Ky=3  Ky=4  Ky=5  Ky=6  Ky=7  Ky=8  Ky=9 Ky=10
## Kx=2 0.72 0.404 0.231 0.813 0.458 0.197 0.473 0.221 0.243
```

```
set.seed(61916)

sickcode <- data.frame(Sick = cowattribdat$Sick_Pasture)
rownames(sickcode) <- cowattribdat$CowID

compareEncodings(sickcode,
                 dmgridout_all$nrow_5_ncol_3,
                 colorby = 'PMI',
                 simPMI = 'NullParam',
                 alpha = 0.05,
                 nsim = 2000,
                 gradient_color_low = 'steelblue',
                 gradient_color_high = 'orange',
                 filename = 'Viz/OverallTB/BivarTest_Sick/EO_HealthPasture',
                 fontsize = 30,
                 imheight = 38,
                 imwidth = 30)
```

```
## file saved to Viz/OverallTB/BivarTest_Sick/EO_HealthPasture.pdf
```

No significant association.

## Bivar Tests - OTB vs Diagnosis

```
cowattribdat$Diagnosis <- cowattribdat$DIGNOSIS

cowattribdat$Diagnosis[
  cowattribdat$Diagnosis == 'met'|
  cowattribdat$Diagnosis == 'rp' |
  cowattribdat$Diagnosis == 'uta' |
  cowattribdat$Diagnosis == 'pyo'] <- 'Repro' 

cowattribdat$Diagnosis[
  cowattribdat$Diagnosis == 'mast' |
  cowattribdat$Diagnosis == 'edm'] <- 'Mammary'

cowattribdat$Diagnosis[
  cowattribdat$Diagnosis == 'acd'|
  cowattribdat$Diagnosis == 'ket' |
  cowattribdat$Diagnosis == 'lda' |
  cowattribdat$Diagnosis == 'con' ] <- 'Digestive' 

cowattribdat$Diagnosis[cowattribdat$Diagnosis == 'dig'|
  cowattribdat$Diagnosis == 'lame'] <- 'Hoof' 

cowattribdat$Diagnosis[cowattribdat$Diagnosis == 'inj'|
  cowattribdat$Diagnosis == 'resp' |
  cowattribdat$Diagnosis == 'tore'] <- NA
cowattribdat$Diagnosis[is.na(cowattribdat$Diagnosis)] <- 'Healthy'


#table(cowattribdat$Diagnosis)

cowattribdat$DiagnosisPen <- cowattribdat$Diagnosis
cowattribdat$DiagnosisPen[cowattribdat$Sick_Prepasture == "Healthy"] <- 'Healthy'

cowattribdat$DiagnosisPen <- factor(cowattribdat$DiagnosisPen,
                levels = c('Healthy', 'Hoof', 'Mammary', 'Repro', 'Digestive'), ordered = T) 

table(cowattribdat$DiagnosisPen)
```

```
## 
##   Healthy      Hoof   Mammary     Repro Digestive 
##       154         5        19        17         7
```

#### Noise Penalized

```
set.seed(6191)

sickcode <- data.frame(Sick = cowattribdat$DiagnosisPen)
rownames(sickcode) <- cowattribdat$CowID

bvt_diagpen_ew <- bivarEncodeTest(sickcode,
                    encodeout_otb_ew,
                    grid_y = 2:10, estimator = 'shrink',
                    rescaleMI = T, verbose = T)
```

```
bvt_diagpen_ew$PVals
```

```
## , , XClust_ncol_0, YClust_ncol_0
## 
##       Ky=2  Ky=3  Ky=4  Ky=5  Ky=6  Ky=7  Ky=8  Ky=9 Ky=10
## Kx=5 0.356 0.072 0.011 0.012 0.006 0.013 0.008 0.015 0.032
```

```
set.seed(61916)

sickcode <- data.frame(Sick = cowattribdat$DiagnosisPen)
rownames(sickcode) <- cowattribdat$CowID


compareEncodings(sickcode,
                 EncodingsOTB$Ensemble$nrow_7_ncol_0, 
                 colorby = 'PMI',
                 simPMI = 'NullParam',
                 alpha = 0.05,
                 nsim = 2000,
                 gradient_color_low = 'steelblue',
                 gradient_color_high = 'orange',
                 filename = 'Viz/OverallTB/BivarTest_Sick/OTB_Diag_NP',
                 fontsize = 30,
                 imheight = 38,
                 imwidth = 30)
```

```
## file saved to Viz/OverallTB/BivarTest_Sick/OTB_Diag_NP.pdf
```

Among cows with the most moderate time budgets, healthy cows were over-represented, and cows with digestive and reproductive illnesses were slightly under-represented.

Digestive/metabolic diseases were only found among cows with low time spent eating.

Mammary diseases were slightly over-represented among cows with extremely high time spent eating.

Cows with moderate time spent eating, low nonactivity, and relatively high time spent ruminating were slightly over-represented among reproductive diseases

No significant associations were recovered with time budget encodings for hoof diseases.

#### Plasticity Penalized

```
set.seed(6191)

sickcode <- data.frame(Sick = cowattribdat$DiagnosisPen)
rownames(sickcode) <- cowattribdat$CowID

bvt_diagpen_pw <- bivarEncodeTest(sickcode,
                    EncodingsOTB$Plasticity,
                    grid_y = 2:10,
                    estimator = 'shrink',
                    rescaleMI = T, verbose = T)
```

```
bvt_diagpen_pw$PVals
```

```
## , , XClust_ncol_0, YClust_ncol_0
## 
##       Ky=2  Ky=3  Ky=4  Ky=5  Ky=6  Ky=7  Ky=8  Ky=9 Ky=10
## Kx=5 0.046 0.007 0.012 0.022 0.085 0.047 0.011 0.017 0.043
```

```
set.seed(61916)

sickcode <- data.frame(Sick = cowattribdat$DiagnosisPen)
rownames(sickcode) <- cowattribdat$CowID

compareEncodings(sickcode,
                 EncodingsOTB$Plasticity$nrow_9_ncol_0,
                 colorby = 'PMI',
                 simPMI = 'NullParam',
                 alpha = 0.05,
                 gradient_color_low = 'steelblue',
                 gradient_color_high = 'orange',
                 filename = 'Viz/OverallTB/BivarTest_Sick/OTB_Diag_PP',
                 fontsize = 30,
                 imheight = 38,
                 imwidth = 30)
```

```
## file saved to Viz/OverallTB/BivarTest_Sick/OTB_Diag_PP.pdf
```

Cows with time budgets with extremely high amount of time spent eating were over-represented among cows with mammary infections.

Cows that spent moderate among of timeeating and a high amount of time nonactive were over-represented among reproductive diseases. While cows with the most moderate time bugets were under-represented among reproductive diseases.

## Bivar test - Entry Position vs Diagnosis

```
set.seed(6191)

sickcode <- data.frame(Sick = cowattribdat$DiagnosisPen)
rownames(sickcode) <- cowattribdat$CowID

bvt_diagpen_eo <- bivarEncodeTest(sickcode,
                    dmgridout_all,
                    grid_y = 2:10, 
                    estimator = 'shrink',
                    rescaleMI = T, verbose = T)
```

```
bvt_diagpen_eo$PVals
```

```
## , , XClust_ncol_0, YClust_ncol_1
## 
##       Ky=2  Ky=3  Ky=4  Ky=5 Ky=6  Ky=7  Ky=8  Ky=9 Ky=10
## Kx=5 0.559 0.699 0.759 0.675 0.75 0.864 0.811 0.712 0.804
## 
## , , XClust_ncol_0, YClust_ncol_2
## 
##       Ky=2  Ky=3  Ky=4  Ky=5  Ky=6  Ky=7  Ky=8 Ky=9 Ky=10
## Kx=5 0.333 0.768 0.658 0.809 0.627 0.942 0.947 0.46 0.936
## 
## , , XClust_ncol_0, YClust_ncol_3
## 
##       Ky=2  Ky=3  Ky=4  Ky=5  Ky=6  Ky=7  Ky=8  Ky=9 Ky=10
## Kx=5 0.369 0.573 0.814 0.547 0.339 0.929 0.901 0.619 0.522
## 
## , , XClust_ncol_0, YClust_ncol_4
## 
##       Ky=2  Ky=3  Ky=4  Ky=5  Ky=6  Ky=7  Ky=8  Ky=9 Ky=10
## Kx=5 0.221 0.033 0.089 0.574 0.359 0.824 0.606 0.871 0.868
## 
## , , XClust_ncol_0, YClust_ncol_5
## 
##       Ky=2  Ky=3  Ky=4  Ky=5  Ky=6  Ky=7  Ky=8  Ky=9 Ky=10
## Kx=5 0.617 0.297 0.419 0.644 0.662 0.594 0.702 0.796  0.53
## 
## , , XClust_ncol_0, YClust_ncol_6
## 
##       Ky=2  Ky=3 Ky=4  Ky=5  Ky=6  Ky=7  Ky=8  Ky=9 Ky=10
## Kx=5 0.726 0.683 0.55 0.674 0.424 0.348 0.944 0.635  0.65
```

```
set.seed(61916)

sickcode <- data.frame(Sick = cowattribdat$DiagnosisPen)
rownames(sickcode) <- cowattribdat$CowID

compareEncodings(sickcode,
                 dmgridout_all$nrow_3_ncol_4,
                 colorby = 'PMI',
                 simPMI = 'NullParam',
                 alpha = 0.05,
                 gradient_color_low = 'steelblue',
                 gradient_color_high = 'orange',
                 filename = 'Viz/OverallTB/BivarTest_Sick/EO_Diag',
                 fontsize = 30,
                 imheight = 38,
                 imwidth = 30)
```

```
## file saved to Viz/OverallTB/BivarTest_Sick/EO_Diag.pdf
```

Cows that entered at the back or nearer the back of the herd were under-represented among the healthy cows, and over-represented among cows with reproductive diseases.

## Bivar Test - OTB vs Entry Position

### Noise Penalized

#### Healthy Cows

```
set.seed(619166)

bvt_otb_ew <- bivarEncodeTest(EncodingsOTB$Ensemble,
                    dmgridout_healthy,
                    grid_x = 2:10, grid_y = 2:10,
                    estimator = 'shrink',
                    rescaleMI = T, verbose = T)
```

```
bvt_otb_ew$PVals
```

```
## , , XClust_ncol_0, YClust_ncol_1
## 
##        Ky=2  Ky=3  Ky=4  Ky=5  Ky=6  Ky=7  Ky=8  Ky=9 Ky=10
## Kx=2  0.435 0.803 0.738 0.877 0.993 0.977 0.858 0.640 0.644
## Kx=3  0.680 0.947 0.498 0.348 0.458 0.573 0.463 0.327 0.467
## Kx=4  0.513 0.842 0.581 0.084 0.107 0.137 0.057 0.029 0.078
## Kx=5  0.707 0.952 0.844 0.288 0.384 0.364 0.200 0.106 0.226
## Kx=6  0.544 0.768 0.674 0.138 0.295 0.334 0.218 0.141 0.149
## Kx=7  0.301 0.055 0.106 0.009 0.033 0.015 0.019 0.020 0.016
## Kx=8  0.377 0.085 0.149 0.011 0.032 0.018 0.030 0.026 0.020
## Kx=9  0.466 0.125 0.187 0.020 0.056 0.033 0.034 0.034 0.033
## Kx=10 0.531 0.157 0.195 0.035 0.046 0.035 0.024 0.014 0.016
## 
## , , XClust_ncol_0, YClust_ncol_2
## 
##        Ky=2  Ky=3  Ky=4  Ky=5  Ky=6  Ky=7  Ky=8  Ky=9 Ky=10
## Kx=2  0.582 0.777 0.831 0.949 0.959 0.990 0.760 0.950 0.913
## Kx=3  0.848 0.549 0.582 0.386 0.304 0.369 0.462 0.895 0.769
## Kx=4  0.656 0.616 0.633 0.065 0.054 0.103 0.025 0.702 0.133
## Kx=5  0.730 0.824 0.814 0.261 0.227 0.274 0.097 0.878 0.273
## Kx=6  0.668 0.580 0.834 0.222 0.203 0.171 0.079 0.772 0.264
## Kx=7  0.249 0.118 0.196 0.017 0.013 0.014 0.003 0.097 0.028
## Kx=8  0.264 0.191 0.214 0.012 0.016 0.014 0.003 0.119 0.015
## Kx=9  0.333 0.280 0.309 0.028 0.021 0.017 0.002 0.167 0.031
## Kx=10 0.388 0.294 0.199 0.020 0.016 0.011 0.002 0.138 0.008
## 
## , , XClust_ncol_0, YClust_ncol_3
## 
##        Ky=2  Ky=3  Ky=4  Ky=5  Ky=6  Ky=7  Ky=8  Ky=9 Ky=10
## Kx=2  0.858 0.890 0.897 0.993 0.973 0.980 0.865 0.900 0.969
## Kx=3  0.871 0.457 0.525 0.662 0.533 0.926 0.671 0.515 0.994
## Kx=4  0.762 0.458 0.615 0.187 0.166 0.181 0.070 0.071 0.243
## Kx=5  0.846 0.713 0.781 0.480 0.395 0.469 0.229 0.182 0.522
## Kx=6  0.895 0.618 0.595 0.280 0.247 0.311 0.165 0.200 0.319
## Kx=7  0.017 0.076 0.176 0.017 0.013 0.082 0.009 0.026 0.005
## Kx=8  0.027 0.102 0.161 0.014 0.009 0.065 0.010 0.046 0.002
## Kx=9  0.040 0.141 0.221 0.020 0.013 0.098 0.015 0.062 0.006
## Kx=10 0.046 0.109 0.194 0.022 0.009 0.052 0.012 0.019 0.006
## 
## , , XClust_ncol_0, YClust_ncol_4
## 
##        Ky=2  Ky=3  Ky=4  Ky=5  Ky=6  Ky=7  Ky=8  Ky=9 Ky=10
## Kx=2  0.858 0.728 0.862 0.996 0.958 0.945 0.398 0.968 0.838
## Kx=3  0.871 0.465 0.683 0.484 0.272 0.627 0.583 0.736 0.695
## Kx=4  0.762 0.550 0.743 0.119 0.054 0.014 0.054 0.393 0.358
## Kx=5  0.846 0.785 0.928 0.400 0.115 0.037 0.226 0.469 0.337
## Kx=6  0.895 0.844 0.907 0.265 0.092 0.042 0.256 0.407 0.271
## Kx=7  0.017 0.249 0.175 0.017 0.009 0.000 0.114 0.063 0.040
## Kx=8  0.027 0.338 0.215 0.019 0.007 0.002 0.126 0.068 0.050
## Kx=9  0.040 0.435 0.302 0.037 0.011 0.008 0.147 0.123 0.053
## Kx=10 0.046 0.431 0.217 0.033 0.014 0.001 0.112 0.096 0.018
## 
## , , XClust_ncol_0, YClust_ncol_5
## 
##        Ky=2  Ky=3  Ky=4  Ky=5  Ky=6  Ky=7  Ky=8  Ky=9 Ky=10
## Kx=2  0.858 0.863 0.792 0.997 0.901 0.948 0.909 0.986 1.000
## Kx=3  0.871 0.665 0.431 0.643 0.565 0.415 0.410 0.766 0.828
## Kx=4  0.762 0.633 0.400 0.160 0.078 0.144 0.064 0.100 0.496
## Kx=5  0.846 0.838 0.661 0.458 0.270 0.230 0.194 0.225 0.683
## Kx=6  0.895 0.642 0.442 0.292 0.212 0.208 0.158 0.046 0.703
## Kx=7  0.017 0.100 0.200 0.017 0.024 0.003 0.010 0.002 0.144
## Kx=8  0.027 0.123 0.284 0.013 0.049 0.001 0.013 0.003 0.148
## Kx=9  0.040 0.177 0.357 0.017 0.079 0.007 0.017 0.007 0.111
## Kx=10 0.046 0.196 0.426 0.018 0.044 0.008 0.004 0.004 0.060
## 
## , , XClust_ncol_0, YClust_ncol_6
## 
##        Ky=2  Ky=3  Ky=4  Ky=5  Ky=6  Ky=7  Ky=8  Ky=9 Ky=10
## Kx=2  0.751 0.997 0.950 0.985 0.918 0.690 0.985 0.961 0.921
## Kx=3  0.926 0.999 0.352 0.611 0.368 0.602 0.703 0.732 0.381
## Kx=4  0.709 0.998 0.424 0.425 0.113 0.174 0.132 0.034 0.038
## Kx=5  0.852 0.996 0.710 0.652 0.336 0.410 0.256 0.075 0.168
## Kx=6  0.849 0.969 0.572 0.383 0.209 0.298 0.240 0.061 0.130
## Kx=7  0.177 0.184 0.160 0.047 0.037 0.012 0.031 0.001 0.011
## Kx=8  0.211 0.211 0.225 0.054 0.041 0.013 0.031 0.001 0.015
## Kx=9  0.266 0.262 0.307 0.091 0.046 0.015 0.059 0.001 0.011
## Kx=10 0.306 0.311 0.161 0.074 0.040 0.016 0.063 0.001 0.001
```

```
#getBVTPval(bvt_otb_ew$PVals)
#which(min(bvt_otb_ew$PVals) == bvt_otb_ew$PVals, arr.ind = T)
```

```
set.seed(61916)

#View(bvt_otb_ew$TestResults$XCluster8_YCluster5) #C3

compareEncodings(EncodingsOTB$Ensemble$nrow_7_ncol_0,
                 dmgridout_healthy$nrow_7_ncol_4,
                 alpha = 0.05,
                 nsim = 2000,
                 colorby = 'PMI',
                 simPMI = 'NullParam',
                 gradient_color_low = 'steelblue',
                 gradient_color_high = 'orange',
                 filename = 'Viz/OverallTB/BivarTest_EntryOrder/OTB_Healthy_NP',
                 fontsize = 30,
                 imheight = 38,
                 imwidth = 30)
```

```
## file saved to Viz/OverallTB/BivarTest_EntryOrder/OTB_Healthy_NP.pdf
```

Cows that consistently enter either in the center or nearer the back of the queue are under-represented among the moderate time budgets, and over-represented in the time budget cluster characterized by relatively low time spent eating and moderate time pent nonative or ruminating (the same one with elevated rates of health complications).

Among healthy cows no time budget clusters were over-represented in the time budget cluster with extremely low time spent eating with high rates of nonactivity and rumination, but most of the cows remaining in this cluster were found nearer the end of the herd.

Cows that entered either in the center or nearer the front of the queue while while housed over night in the pen, and shifted their queue position nearer the rear of the herd when over-nighting on pasture, were also slightly over-represented in the time budget cluster characterized low time spent eating, low-to-moderate time spent nonactive, moderate-to-high time spent ruminating, and higher rates of activity.

Cows that entered just behind the leaders were under-represented in the time budget cluster characterized by high time spent ruminating and low time spent eating or nonactive.

#### All Cows

And now all the cows.

```
set.seed(6191)

bvt_otb_ew_all <- bivarEncodeTest(EncodingsOTB$Ensemble,
                    dmgridout_all,
                    grid_x = 2:10, grid_y = 2:10,
                    estimator = 'shrink',
                    rescaleMI = T, verbose = T)
```

```
bvt_otb_ew_all$PVals
```

```
## , , XClust_ncol_0, YClust_ncol_1
## 
##        Ky=2  Ky=3  Ky=4  Ky=5  Ky=6  Ky=7  Ky=8  Ky=9 Ky=10
## Kx=2  0.241 0.531 0.525 0.701 0.852 0.942 0.787 0.817 0.783
## Kx=3  0.428 0.812 0.761 0.843 0.935 0.978 0.958 0.982 0.988
## Kx=4  0.512 0.874 0.877 0.494 0.704 0.645 0.620 0.665 0.560
## Kx=5  0.666 0.893 0.934 0.652 0.823 0.846 0.774 0.753 0.745
## Kx=6  0.683 0.906 0.926 0.717 0.738 0.872 0.862 0.859 0.816
## Kx=7  0.566 0.270 0.517 0.282 0.300 0.584 0.688 0.713 0.752
## Kx=8  0.664 0.319 0.594 0.330 0.400 0.691 0.757 0.792 0.784
## Kx=9  0.750 0.447 0.714 0.429 0.502 0.685 0.803 0.844 0.766
## Kx=10 0.821 0.569 0.815 0.613 0.612 0.764 0.859 0.901 0.871
## 
## , , XClust_ncol_0, YClust_ncol_2
## 
##        Ky=2  Ky=3  Ky=4  Ky=5  Ky=6  Ky=7  Ky=8  Ky=9 Ky=10
## Kx=2  0.251 0.742 0.761 0.867 0.786 0.952 0.977 0.821 0.962
## Kx=3  0.455 0.732 0.816 0.933 0.978 0.772 0.794 0.936 0.849
## Kx=4  0.556 0.735 0.830 0.609 0.873 0.212 0.182 0.334 0.329
## Kx=5  0.619 0.874 0.927 0.721 0.975 0.447 0.379 0.586 0.268
## Kx=6  0.749 0.830 0.878 0.755 0.756 0.278 0.323 0.323 0.354
## Kx=7  0.271 0.197 0.181 0.221 0.339 0.021 0.055 0.004 0.196
## Kx=8  0.347 0.218 0.185 0.198 0.275 0.020 0.092 0.009 0.242
## Kx=9  0.391 0.320 0.254 0.270 0.289 0.041 0.091 0.010 0.327
## Kx=10 0.483 0.325 0.271 0.430 0.387 0.032 0.096 0.011 0.286
## 
## , , XClust_ncol_0, YClust_ncol_3
## 
##        Ky=2  Ky=3  Ky=4  Ky=5  Ky=6  Ky=7  Ky=8  Ky=9 Ky=10
## Kx=2  0.223 0.566 0.193 0.845 0.932 0.728 0.943 0.937 0.841
## Kx=3  0.384 0.787 0.506 0.935 0.954 0.717 0.996 0.968 0.982
## Kx=4  0.423 0.855 0.580 0.860 0.577 0.437 0.985 0.973 0.772
## Kx=5  0.607 0.953 0.777 0.974 0.891 0.525 0.994 0.997 0.887
## Kx=6  0.728 0.826 0.577 0.988 0.727 0.326 0.895 0.981 0.848
## Kx=7  0.300 0.380 0.176 0.705 0.140 0.097 0.434 0.821 0.480
## Kx=8  0.377 0.453 0.257 0.729 0.148 0.109 0.439 0.840 0.540
## Kx=9  0.431 0.498 0.354 0.817 0.203 0.186 0.475 0.864 0.510
## Kx=10 0.510 0.609 0.292 0.865 0.366 0.327 0.562 0.930 0.471
## 
## , , XClust_ncol_0, YClust_ncol_4
## 
##        Ky=2  Ky=3  Ky=4  Ky=5  Ky=6  Ky=7  Ky=8  Ky=9 Ky=10
## Kx=2  0.438 0.659 0.724 0.743 0.551 0.900 0.920 0.964 0.850
## Kx=3  0.535 0.608 0.791 0.859 0.843 0.944 0.951 0.571 0.939
## Kx=4  0.537 0.748 0.729 0.512 0.409 0.806 0.238 0.182 0.694
## Kx=5  0.686 0.845 0.853 0.750 0.616 0.980 0.606 0.569 0.753
## Kx=6  0.625 0.890 0.547 0.595 0.409 0.898 0.436 0.589 0.769
## Kx=7  0.008 0.649 0.306 0.045 0.060 0.264 0.128 0.190 0.383
## Kx=8  0.011 0.728 0.377 0.046 0.102 0.327 0.135 0.211 0.417
## Kx=9  0.025 0.799 0.497 0.072 0.163 0.358 0.141 0.334 0.476
## Kx=10 0.038 0.799 0.636 0.144 0.196 0.283 0.201 0.213 0.407
## 
## , , XClust_ncol_0, YClust_ncol_5
## 
##        Ky=2  Ky=3  Ky=4  Ky=5  Ky=6  Ky=7  Ky=8  Ky=9 Ky=10
## Kx=2  0.281 0.917 0.224 0.578 0.840 0.992 0.945 0.981 0.936
## Kx=3  0.401 0.850 0.521 0.857 0.939 0.976 0.844 0.982 0.922
## Kx=4  0.583 0.837 0.057 0.184 0.833 0.134 0.099 0.946 0.583
## Kx=5  0.765 0.851 0.155 0.192 0.970 0.312 0.241 0.951 0.766
## Kx=6  0.869 0.447 0.100 0.093 0.928 0.253 0.114 0.730 0.569
## Kx=7  0.353 0.314 0.018 0.006 0.357 0.104 0.006 0.164 0.043
## Kx=8  0.445 0.397 0.028 0.006 0.450 0.152 0.010 0.180 0.030
## Kx=9  0.500 0.516 0.047 0.011 0.538 0.174 0.020 0.254 0.031
## Kx=10 0.595 0.513 0.045 0.009 0.364 0.159 0.032 0.409 0.110
## 
## , , XClust_ncol_0, YClust_ncol_6
## 
##        Ky=2  Ky=3  Ky=4  Ky=5  Ky=6  Ky=7  Ky=8  Ky=9 Ky=10
## Kx=2  0.604 0.512 0.878 0.762 0.797 0.933 0.760 0.906 0.971
## Kx=3  0.822 0.711 0.978 0.957 0.926 0.746 0.675 0.990 0.950
## Kx=4  0.812 0.325 0.571 0.931 0.264 0.390 0.098 0.779 0.812
## Kx=5  0.897 0.458 0.863 0.996 0.621 0.762 0.278 0.755 0.972
## Kx=6  0.913 0.623 0.386 0.917 0.381 0.702 0.267 0.639 0.903
## Kx=7  0.039 0.223 0.087 0.711 0.070 0.237 0.097 0.209 0.632
## Kx=8  0.062 0.263 0.118 0.769 0.088 0.108 0.146 0.326 0.755
## Kx=9  0.110 0.262 0.164 0.821 0.123 0.131 0.145 0.466 0.728
## Kx=10 0.153 0.388 0.265 0.752 0.104 0.107 0.151 0.500 0.395
```

```
#getBVTPval(bvt_otb_ew_all$PVals)
```

```
set.seed(61916)

#View(bvt_otb_ew_all$TestResults$XCluster7_YCluster5) #C2

compareEncodings(EncodingsOTB$Ensemble$nrow_7_ncol_0,
                 dmgridout_all$nrow_5_ncol_5,
                 colorby = 'PMI',
                 simPMI = 'NullParam',
                 alpha = 0.05,
                 nsim = 2000,
                 gradient_color_low = 'steelblue',
                 gradient_color_high = 'orange',
                 filename = 'Viz/OverallTB/BivarTest_EntryOrder/OTB_All_NP',
                 fontsize = 30,
                 imheight = 38,
                 imwidth = 30)
```

```
## file saved to Viz/OverallTB/BivarTest_EntryOrder/OTB_All_NP.pdf
```

Cows that consistently entered at the very end of the queue were over-represented in the time budget cluster characterized by moderate time spent eating, low time nonactive, and high time spent ruminating. These same cows were also significantly under-represented in the time budget cluster characterized by extremely low time spent eating and high time spent ruminating and nonactive.

Interestingly, cows that consistently entered just ahead of these caboose cows were over-represented in the time budget cluster characterized by extremely low time spent eating and high time spent ruminating and nonactive, which the caboose cows were under-represented in. These cows consistently entering nearer the end of the queue were also under-represented among the more moderate time budgets (which also had lower rates of illness).

Cows that consistently entered nearer the front of the queue, but not amongst the leaders, were also under-represented in the time budget cluster characterized by moderate time spent eating, low time nonactive, and high time spent ruminating, which the caboose cows were over-represented in.

### Plasticity Penalized

#### Healthy Cows

First the healthy cows

```
set.seed(61916)

bvt_otb_pw <- bivarEncodeTest(EncodingsOTB$Plasticity,
                    dmgridout_healthy,
                    grid_x = 2:10, grid_y = 2:10,
                    estimator = 'shrink',
                    rescaleMI = T, verbose = T)
```

```
bvt_otb_pw$PVals
```

```
## , , XClust_ncol_0, YClust_ncol_1
## 
##        Ky=2  Ky=3  Ky=4  Ky=5  Ky=6  Ky=7  Ky=8  Ky=9 Ky=10
## Kx=2  0.513 0.578 0.503 0.316 0.288 0.303 0.116 0.080 0.140
## Kx=3  0.522 0.885 0.624 0.486 0.568 0.634 0.219 0.134 0.188
## Kx=4  0.779 0.980 0.848 0.706 0.812 0.534 0.280 0.190 0.312
## Kx=5  0.723 0.805 0.786 0.659 0.385 0.186 0.095 0.088 0.180
## Kx=6  0.801 0.578 0.647 0.822 0.284 0.204 0.102 0.110 0.189
## Kx=7  0.779 0.500 0.597 0.508 0.100 0.080 0.040 0.038 0.070
## Kx=8  0.671 0.413 0.502 0.176 0.035 0.034 0.022 0.025 0.016
## Kx=9  0.631 0.332 0.470 0.193 0.078 0.024 0.020 0.022 0.010
## Kx=10 0.665 0.505 0.532 0.307 0.218 0.089 0.093 0.111 0.095
## 
## , , XClust_ncol_0, YClust_ncol_2
## 
##        Ky=2  Ky=3  Ky=4  Ky=5  Ky=6  Ky=7  Ky=8  Ky=9 Ky=10
## Kx=2  0.474 0.504 0.448 0.220 0.215 0.274 0.024 0.810 0.097
## Kx=3  0.558 0.747 0.702 0.476 0.471 0.580 0.086 0.957 0.341
## Kx=4  0.623 0.911 0.673 0.724 0.541 0.623 0.112 0.917 0.395
## Kx=5  0.322 0.891 0.379 0.237 0.144 0.455 0.033 0.394 0.325
## Kx=6  0.431 0.834 0.123 0.066 0.151 0.431 0.055 0.483 0.482
## Kx=7  0.475 0.683 0.103 0.015 0.041 0.151 0.016 0.372 0.229
## Kx=8  0.316 0.646 0.094 0.007 0.015 0.030 0.007 0.256 0.240
## Kx=9  0.427 0.524 0.064 0.005 0.015 0.015 0.004 0.463 0.132
## Kx=10 0.558 0.667 0.117 0.061 0.046 0.054 0.023 0.649 0.321
## 
## , , XClust_ncol_0, YClust_ncol_3
## 
##        Ky=2  Ky=3  Ky=4  Ky=5  Ky=6  Ky=7  Ky=8  Ky=9 Ky=10
## Kx=2  0.407 0.504 0.545 0.229 0.469 0.117 0.086 0.127 0.246
## Kx=3  0.750 0.671 0.755 0.432 0.735 0.285 0.205 0.250 0.575
## Kx=4  0.872 0.864 0.660 0.633 0.674 0.392 0.260 0.342 0.621
## Kx=5  0.476 0.431 0.596 0.433 0.638 0.296 0.062 0.068 0.035
## Kx=6  0.391 0.589 0.488 0.565 0.716 0.317 0.048 0.144 0.091
## Kx=7  0.338 0.513 0.451 0.386 0.476 0.224 0.008 0.047 0.067
## Kx=8  0.415 0.400 0.441 0.066 0.168 0.109 0.007 0.024 0.054
## Kx=9  0.107 0.362 0.571 0.088 0.085 0.110 0.002 0.031 0.028
## Kx=10 0.172 0.470 0.680 0.201 0.170 0.244 0.033 0.123 0.202
## 
## , , XClust_ncol_0, YClust_ncol_4
## 
##        Ky=2  Ky=3  Ky=4  Ky=5  Ky=6  Ky=7  Ky=8  Ky=9 Ky=10
## Kx=2  0.407 0.421 0.490 0.656 0.185 0.055 0.089 0.251 0.443
## Kx=3  0.750 0.656 0.783 0.919 0.470 0.213 0.096 0.601 0.838
## Kx=4  0.872 0.869 0.907 0.975 0.272 0.133 0.242 0.523 0.394
## Kx=5  0.476 0.781 0.574 0.713 0.134 0.048 0.118 0.137 0.158
## Kx=6  0.391 0.793 0.409 0.876 0.155 0.114 0.190 0.219 0.411
## Kx=7  0.338 0.636 0.320 0.539 0.058 0.025 0.134 0.153 0.252
## Kx=8  0.415 0.645 0.200 0.177 0.018 0.025 0.045 0.155 0.284
## Kx=9  0.107 0.579 0.238 0.288 0.010 0.014 0.075 0.075 0.185
## Kx=10 0.172 0.733 0.370 0.387 0.038 0.051 0.227 0.195 0.610
## 
## , , XClust_ncol_0, YClust_ncol_5
## 
##        Ky=2  Ky=3  Ky=4  Ky=5  Ky=6  Ky=7  Ky=8  Ky=9 Ky=10
## Kx=2  0.407 0.457 0.456 0.450 0.164 0.286 0.035 0.069 0.383
## Kx=3  0.750 0.755 0.575 0.783 0.520 0.697 0.099 0.267 0.886
## Kx=4  0.872 0.924 0.861 0.903 0.671 0.270 0.121 0.198 0.928
## Kx=5  0.476 0.779 0.545 0.498 0.121 0.064 0.052 0.092 0.132
## Kx=6  0.391 0.703 0.539 0.530 0.055 0.032 0.075 0.283 0.239
## Kx=7  0.338 0.551 0.448 0.223 0.012 0.006 0.027 0.101 0.067
## Kx=8  0.415 0.450 0.339 0.056 0.044 0.012 0.008 0.029 0.203
## Kx=9  0.107 0.392 0.455 0.070 0.046 0.003 0.006 0.012 0.293
## Kx=10 0.172 0.548 0.529 0.195 0.263 0.044 0.036 0.109 0.429
## 
## , , XClust_ncol_0, YClust_ncol_6
## 
##        Ky=2  Ky=3  Ky=4  Ky=5  Ky=6  Ky=7  Ky=8  Ky=9 Ky=10
## Kx=2  0.369 0.938 0.336 0.574 0.345 0.461 0.382 0.010 0.040
## Kx=3  0.574 1.000 0.638 0.892 0.586 0.708 0.762 0.095 0.139
## Kx=4  0.710 0.980 0.744 0.942 0.700 0.597 0.859 0.090 0.211
## Kx=5  0.127 0.439 0.326 0.787 0.389 0.129 0.438 0.042 0.163
## Kx=6  0.185 0.377 0.326 0.812 0.390 0.160 0.616 0.118 0.410
## Kx=7  0.226 0.400 0.233 0.495 0.149 0.073 0.332 0.027 0.214
## Kx=8  0.115 0.296 0.264 0.245 0.035 0.021 0.140 0.035 0.091
## Kx=9  0.235 0.045 0.451 0.194 0.039 0.009 0.090 0.014 0.037
## Kx=10 0.297 0.093 0.685 0.290 0.073 0.038 0.262 0.090 0.118
```

```
#getBVTPval(bvt_otb_pw$PVals)
```

```
set.seed(61916)

#View(bvt_otb_pw$TestResults$XCluster8_YCluster5) #C2

compareEncodings(EncodingsOTB$Plasticity$nrow_8_ncol_0,
                 dmgridout_healthy$nrow_5_ncol_2,
                 colorby = 'PMI',
                 simPMI = 'NullParam',
                 alpha = 0.05,
                 nsim = 2000,
                 gradient_color_low = 'steelblue',
                 gradient_color_high = 'orange',
                 filename = 'Viz/OverallTB/BivarTest_EntryOrder/OTB_Healthy_PP',
                 fontsize = 30,
                 imheight = 38,
                 imwidth = 30)
```

```
## file saved to Viz/OverallTB/BivarTest_EntryOrder/OTB_Healthy_PP.pdf
```

Cows found consistently at the very back of the queue (the tail-end charlies) were over-represented in the time budget characterized by moderate-to-high time sepnt eating, low-to-moderate time nonactive, and moderate-to-high time spent ruminating.

Interestingly, cows consistently found nearer the end of the queue (but not the tail end charlies) were under-represented in this moderate time budget cluster that was dominat among the healthy caboose cows. Cows nearer the rear were alo over-represented the time budget cluster with relatively low time spent eating and moderate nonactivity (the same cluster that had elevated rates of illness).

Cows at the center of the queue were under-represented in the time budget cluster with high time spent nonactive and moderate time spent eating.

#### All Cows

And now all the cows.

```
set.seed(61916)

bvt_otb_pw_all <- bivarEncodeTest(EncodingsOTB$Plasticity,
                    dmgridout_all,
                    grid_x = 2:10, grid_y = 2:10,
                    estimator = 'shrink',
                    rescaleMI = T, verbose = T)
```

```
bvt_otb_pw_all$PVals
```

```
## , , XClust_ncol_0, YClust_ncol_1
## 
##        Ky=2  Ky=3  Ky=4  Ky=5  Ky=6  Ky=7  Ky=8  Ky=9 Ky=10
## Kx=2  0.520 0.681 0.913 0.564 0.646 0.586 0.628 0.518 0.377
## Kx=3  0.388 0.775 0.819 0.584 0.775 0.781 0.722 0.704 0.516
## Kx=4  0.603 0.820 0.909 0.629 0.772 0.885 0.789 0.720 0.620
## Kx=5  0.613 0.442 0.682 0.463 0.658 0.730 0.799 0.747 0.738
## Kx=6  0.698 0.448 0.627 0.681 0.787 0.672 0.731 0.767 0.751
## Kx=7  0.682 0.474 0.629 0.649 0.758 0.585 0.663 0.718 0.733
## Kx=8  0.710 0.330 0.464 0.310 0.401 0.325 0.337 0.471 0.441
## Kx=9  0.415 0.149 0.231 0.168 0.177 0.192 0.208 0.216 0.226
## Kx=10 0.247 0.099 0.147 0.136 0.099 0.169 0.213 0.243 0.206
## 
## , , XClust_ncol_0, YClust_ncol_2
## 
##        Ky=2  Ky=3  Ky=4  Ky=5  Ky=6  Ky=7  Ky=8  Ky=9 Ky=10
## Kx=2  0.554 0.594 0.772 0.578 0.643 0.174 0.196 0.024 0.187
## Kx=3  0.377 0.791 0.862 0.658 0.816 0.448 0.502 0.149 0.518
## Kx=4  0.615 0.875 0.864 0.662 0.924 0.552 0.674 0.351 0.429
## Kx=5  0.156 0.424 0.445 0.529 0.614 0.314 0.432 0.064 0.486
## Kx=6  0.220 0.365 0.238 0.741 0.574 0.595 0.501 0.113 0.522
## Kx=7  0.270 0.307 0.205 0.698 0.591 0.393 0.319 0.148 0.385
## Kx=8  0.263 0.172 0.043 0.395 0.476 0.164 0.133 0.189 0.137
## Kx=9  0.252 0.051 0.013 0.236 0.276 0.069 0.047 0.014 0.057
## Kx=10 0.264 0.021 0.010 0.247 0.226 0.102 0.026 0.047 0.029
## 
## , , XClust_ncol_0, YClust_ncol_3
## 
##        Ky=2  Ky=3  Ky=4  Ky=5  Ky=6  Ky=7  Ky=8  Ky=9 Ky=10
## Kx=2  0.465 0.757 0.855 0.742 0.533 0.197 0.820 0.860 0.332
## Kx=3  0.299 0.842 0.454 0.929 0.762 0.466 0.937 0.995 0.581
## Kx=4  0.530 0.931 0.630 0.989 0.894 0.453 0.987 0.998 0.811
## Kx=5  0.159 0.340 0.516 0.390 0.520 0.425 0.841 0.946 0.675
## Kx=6  0.212 0.426 0.582 0.581 0.730 0.658 0.910 0.988 0.650
## Kx=7  0.246 0.482 0.545 0.521 0.619 0.456 0.933 0.979 0.575
## Kx=8  0.189 0.526 0.438 0.434 0.359 0.335 0.702 0.984 0.531
## Kx=9  0.162 0.406 0.356 0.327 0.141 0.231 0.669 0.975 0.097
## Kx=10 0.152 0.463 0.261 0.553 0.171 0.215 0.594 0.983 0.208
## 
## , , XClust_ncol_0, YClust_ncol_4
## 
##        Ky=2  Ky=3  Ky=4  Ky=5  Ky=6  Ky=7  Ky=8  Ky=9 Ky=10
## Kx=2  0.353 0.488 0.752 0.558 0.543 0.648 0.383 0.349 0.512
## Kx=3  0.586 0.689 0.786 0.617 0.554 0.894 0.594 0.769 0.742
## Kx=4  0.703 0.822 0.914 0.771 0.574 0.962 0.751 0.934 0.643
## Kx=5  0.228 0.724 0.820 0.342 0.286 0.763 0.467 0.656 0.550
## Kx=6  0.255 0.822 0.822 0.480 0.455 0.856 0.559 0.690 0.649
## Kx=7  0.196 0.791 0.722 0.393 0.464 0.777 0.340 0.305 0.648
## Kx=8  0.153 0.571 0.678 0.044 0.156 0.690 0.207 0.222 0.324
## Kx=9  0.002 0.423 0.623 0.005 0.072 0.350 0.073 0.078 0.194
## Kx=10 0.006 0.284 0.514 0.011 0.039 0.424 0.101 0.112 0.208
## 
## , , XClust_ncol_0, YClust_ncol_5
## 
##        Ky=2  Ky=3  Ky=4  Ky=5  Ky=6  Ky=7  Ky=8  Ky=9 Ky=10
## Kx=2  0.928 0.348 0.045 0.184 0.791 0.160 0.063 0.339 0.372
## Kx=3  0.484 0.704 0.068 0.164 0.902 0.386 0.293 0.842 0.793
## Kx=4  0.627 0.892 0.238 0.207 0.954 0.535 0.372 0.736 0.904
## Kx=5  0.154 0.666 0.138 0.192 0.573 0.360 0.141 0.584 0.459
## Kx=6  0.192 0.680 0.242 0.419 0.638 0.312 0.198 0.688 0.447
## Kx=7  0.240 0.612 0.179 0.378 0.613 0.179 0.106 0.676 0.320
## Kx=8  0.192 0.401 0.042 0.150 0.297 0.057 0.034 0.525 0.385
## Kx=9  0.194 0.308 0.012 0.092 0.180 0.012 0.002 0.444 0.191
## Kx=10 0.187 0.221 0.048 0.099 0.219 0.037 0.009 0.429 0.259
## 
## , , XClust_ncol_0, YClust_ncol_6
## 
##        Ky=2  Ky=3  Ky=4  Ky=5  Ky=6  Ky=7  Ky=8  Ky=9 Ky=10
## Kx=2  0.344 0.349 0.261 0.862 0.211 0.199 0.107 0.414 0.637
## Kx=3  0.689 0.462 0.460 0.852 0.373 0.382 0.153 0.786 0.932
## Kx=4  0.812 0.577 0.786 0.972 0.693 0.748 0.394 0.679 0.988
## Kx=5  0.225 0.160 0.412 0.773 0.478 0.602 0.316 0.492 0.911
## Kx=6  0.344 0.260 0.554 0.787 0.467 0.701 0.304 0.612 0.924
## Kx=7  0.369 0.239 0.553 0.790 0.386 0.428 0.248 0.538 0.865
## Kx=8  0.359 0.312 0.172 0.497 0.109 0.272 0.171 0.161 0.714
## Kx=9  0.108 0.173 0.055 0.686 0.046 0.177 0.129 0.120 0.487
## Kx=10 0.180 0.134 0.094 0.599 0.039 0.223 0.133 0.124 0.542
```

```
#getBVTPval(bvt_otb_pw_all$PVals)
```

```
set.seed(6191)

#View(bvt_otb_pw_all$TestResults$XCluster9_YCluster5) #C4

compareEncodings(EncodingsOTB$Plasticity$nrow_9_ncol_0, 
                 dmgridout_all$nrow_5_ncol_4,
                 colorby = 'PMI',
                 simPMI = 'NullParam',
                 alpha = 0.05,
                 nsim = 2000,
                 gradient_color_low = 'steelblue',
                 gradient_color_high = 'orange',
                 filename = 'Viz/OverallTB/BivarTest_EntryOrder/OTB_All_PP',
                 fontsize = 30,
                 imheight = 38,
                 imwidth = 30)
```

```
## file saved to Viz/OverallTB/BivarTest_EntryOrder/OTB_All_PP.pdf
```

Cows that entered near the end of the queue were significantly over-represented in the time budget characterized by extremely low time spent eating ang high time nonactive and ruminating. Though not statistically significant, no cows taht consistently entered at the very end of the queue were found in this time budget cluster.

Cows that entered near the end of the queue were significantly over-represented in the time budget cluster characterized by low-to-moderate time spent eating, low time spent eating, slightly elevated activity rates, and high rates of rumination. Cows at the very end of the queue were also over-represented in this time budget cluster. Almost no other cows were found in this time budget cluster, with cows consistently entering just behind the leaders under-represented in this time budget cluster.

Cows that entered near the end of the queue were significantly under-represented in perhaps the most moderate time budget cluster charactertized with moderate-to-high time spent eating, low-to-moderate time spent nonactive, and moderate time spent ruminating. Though not statistically significant, it is notable that this same cluster was comprised nearly 1/3 of the cluster cows that consistently enetered at the very rear of the queue.

Though not significant, it is also noteable that a large number of cows that entered just in front of our caboose cows were also found in the cluster characterized by low time spent eating and moderate nonactivity, and also the cluster with moderate time spend eating and quite high time spent nonactive.

Finally cows are the center of the queue were under-represented in the time budget cluster characterized by moderate time spent eating and elevated time spent nonactive.

# Save output

```
save(EncodingsOTB, file = 'EncodingsOTB.RData')
save.image("OverallTB.RData")
```
