# Supplementary figures and images for: Livestock Informatics Toolkit: A Case Study in Visually Characterizing Complex Behavioral Patterns across Multiple Sensor Platforms, Using Novel Unsupervised Machine Learning and Information Theoretic Approaches (part 2 of 2)
Source: Sensors (Basel). 2021 Dec 21;22(1):1. doi: 10.3390/s22010001 (PMC8747447; doi:10.3390/s22010001)

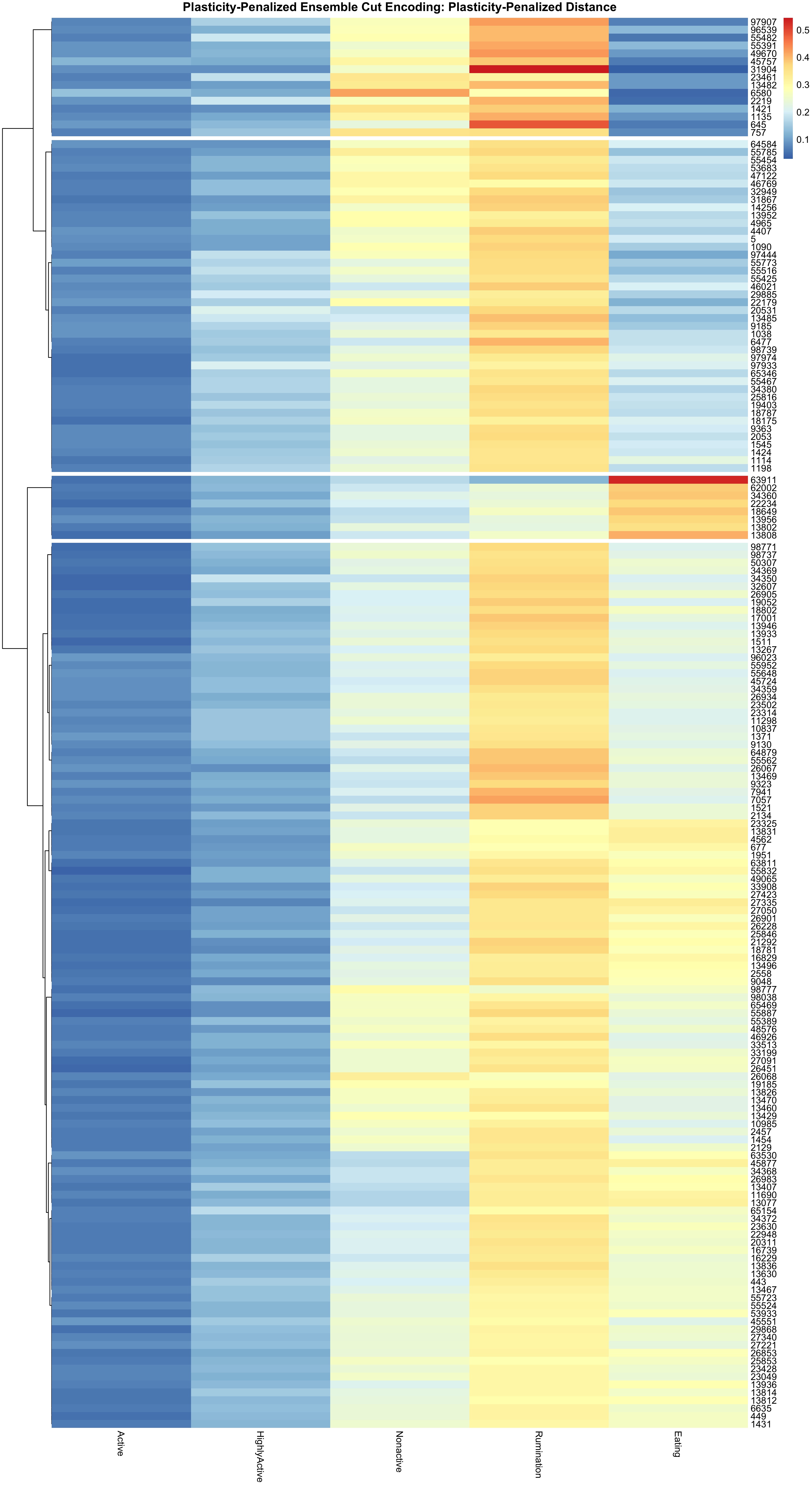

Supplement: Supplementary file 1 [file sensors-22-00001-s001.zip › sensors-1463895-supplementary/OverallTB/EnsembleCut/PPEncode/PWEncode_R4_C0.jpeg]

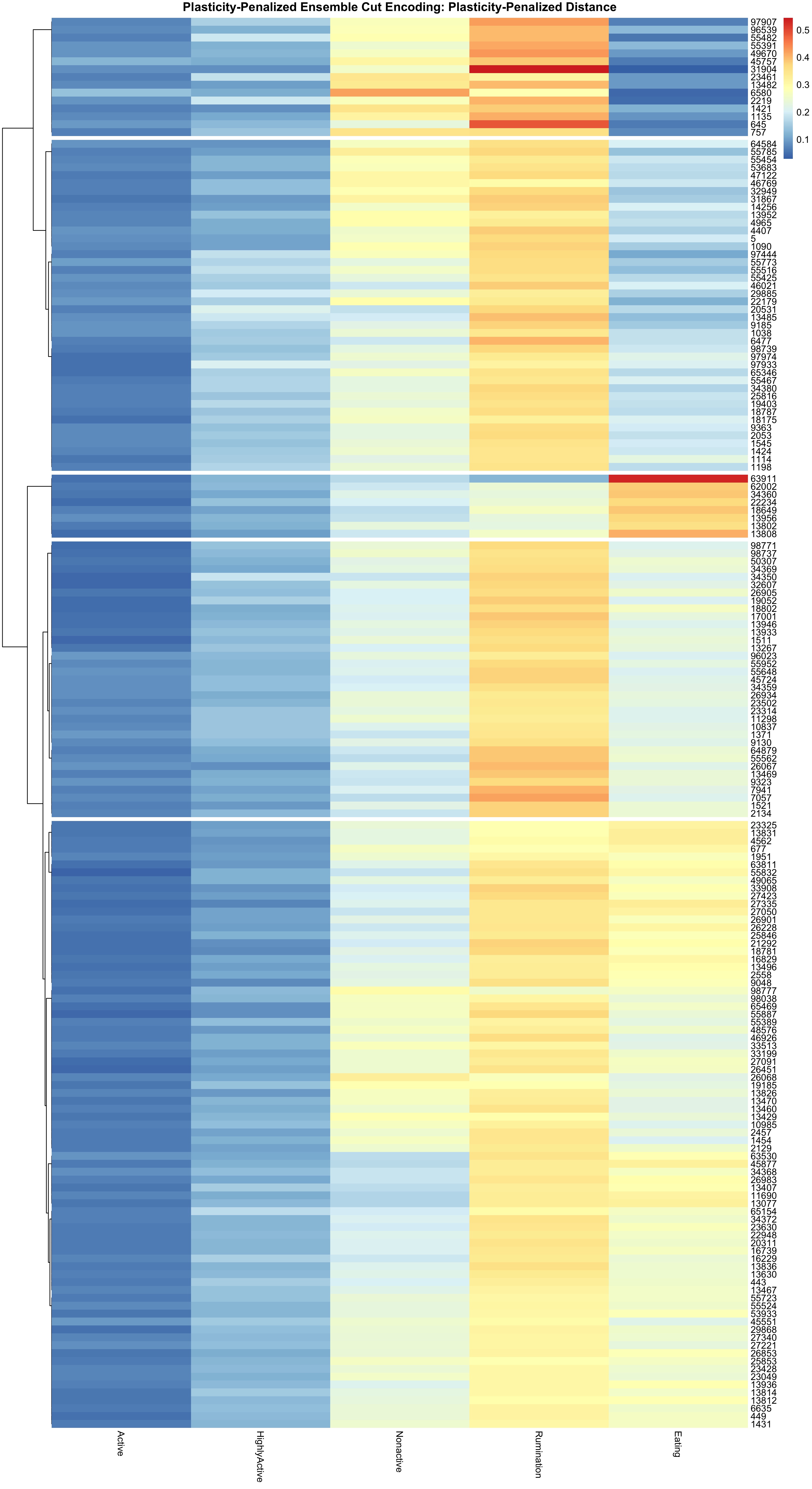

Supplement: Supplementary file 1 [file sensors-22-00001-s001.zip › sensors-1463895-supplementary/OverallTB/EnsembleCut/PPEncode/PWEncode_R5_C0.jpeg]

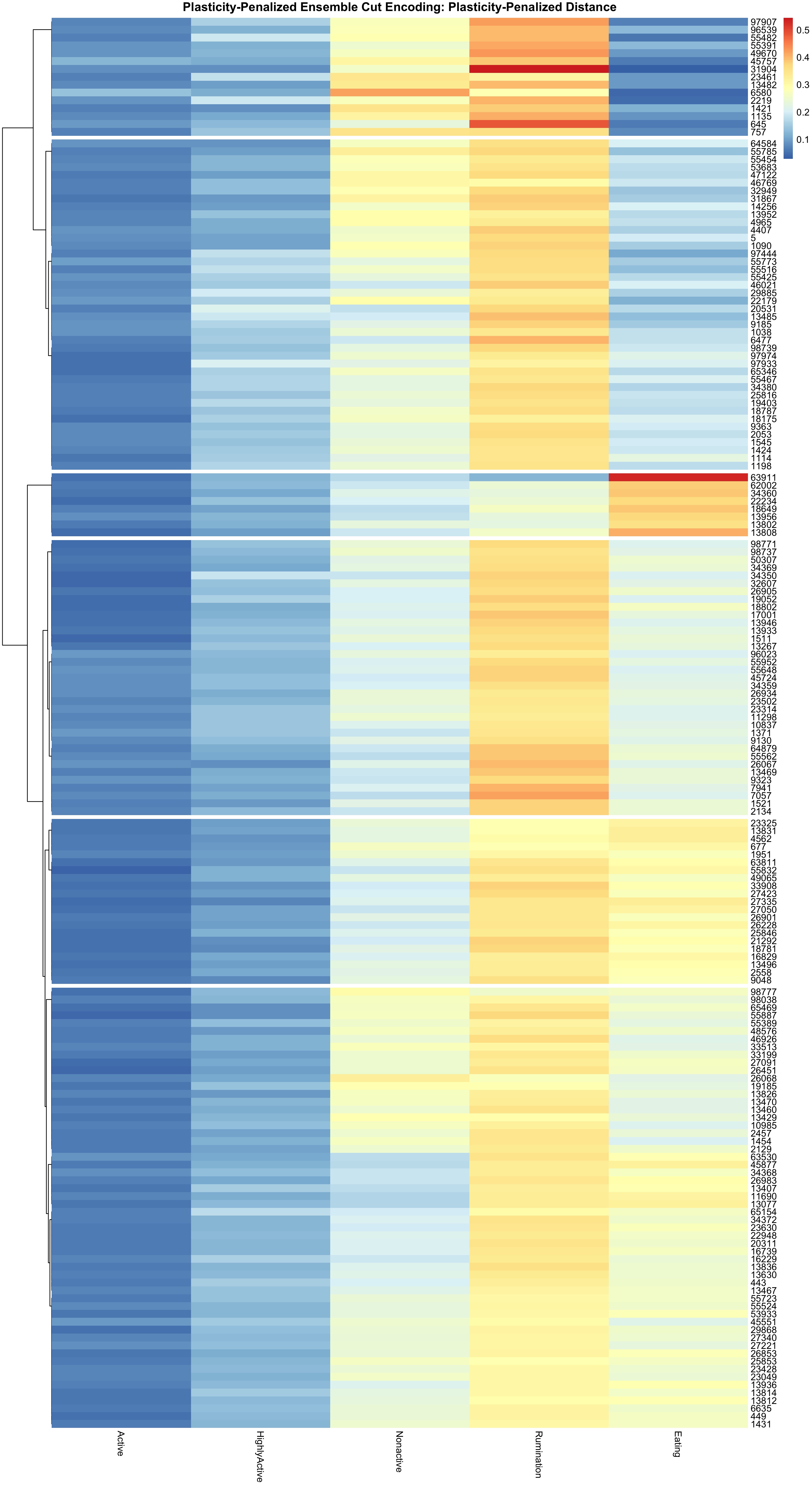

Supplement: Supplementary file 1 [file sensors-22-00001-s001.zip › sensors-1463895-supplementary/OverallTB/EnsembleCut/PPEncode/PWEncode_R6_C0.jpeg]

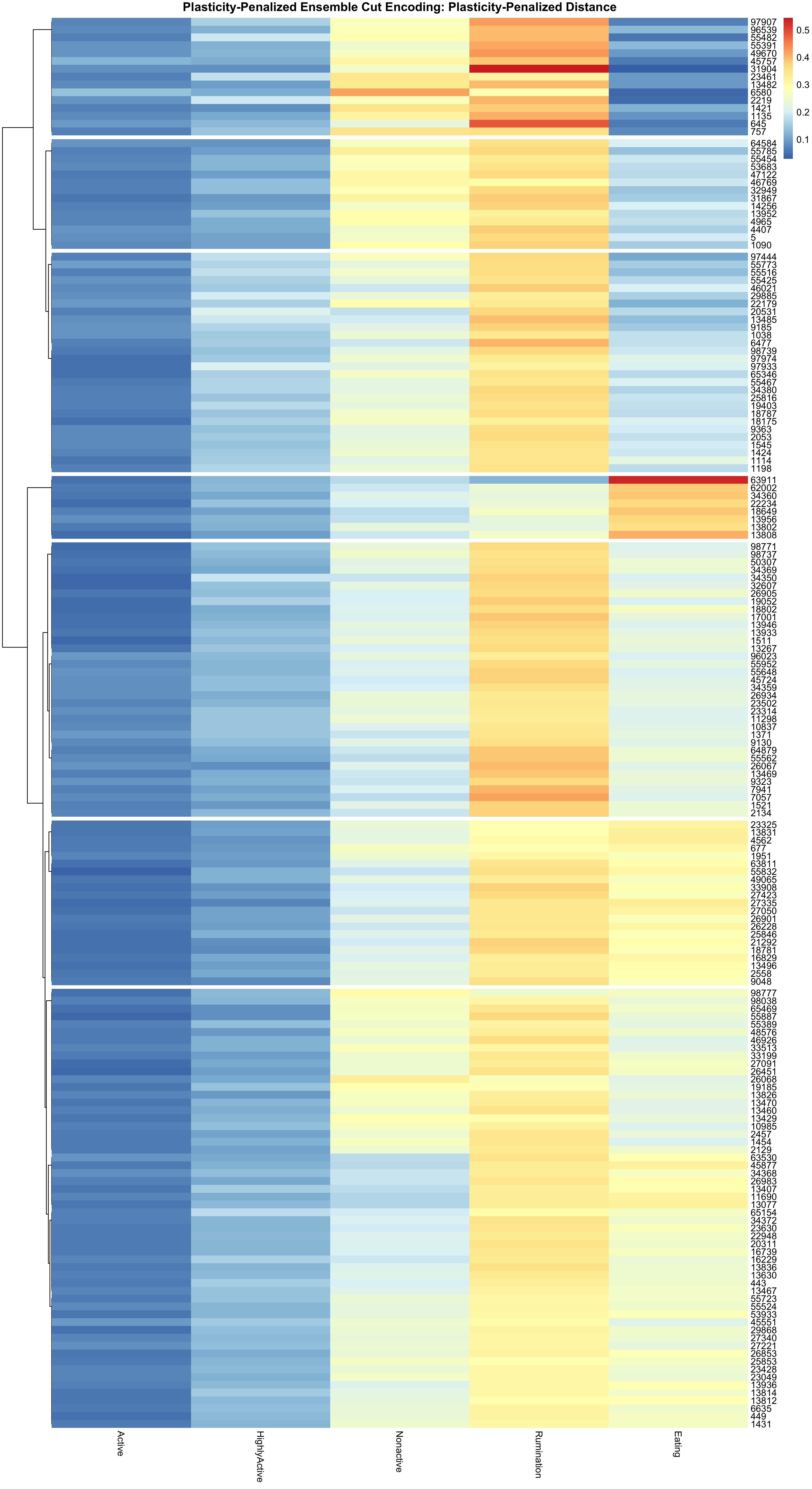

Supplement: Supplementary file 1 [file sensors-22-00001-s001.zip › sensors-1463895-supplementary/OverallTB/EnsembleCut/PPEncode/PWEncode_R7_C0.jpeg]

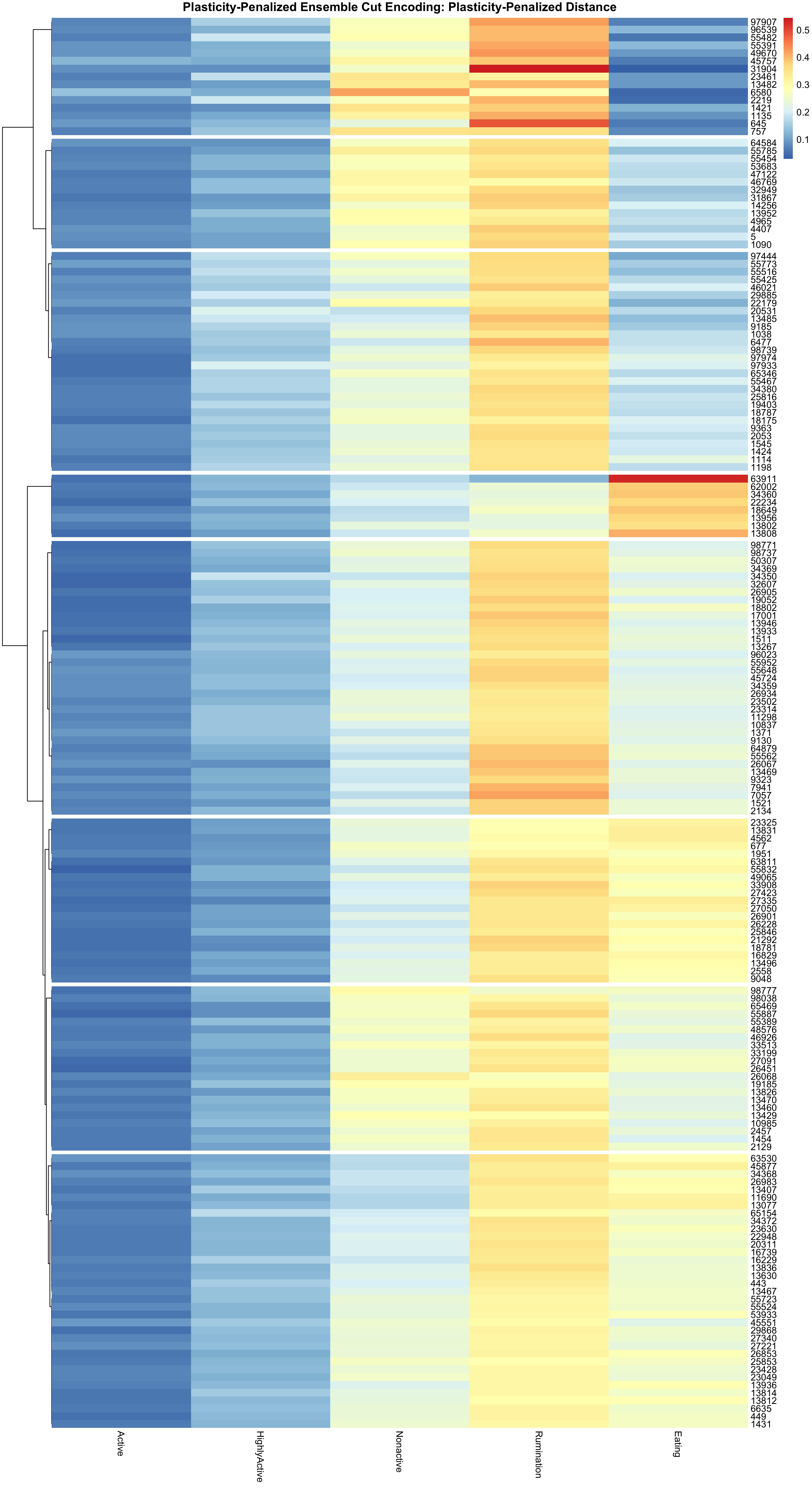

Supplement: Supplementary file 1 [file sensors-22-00001-s001.zip › sensors-1463895-supplementary/OverallTB/EnsembleCut/PPEncode/PWEncode_R8_C0.jpeg]

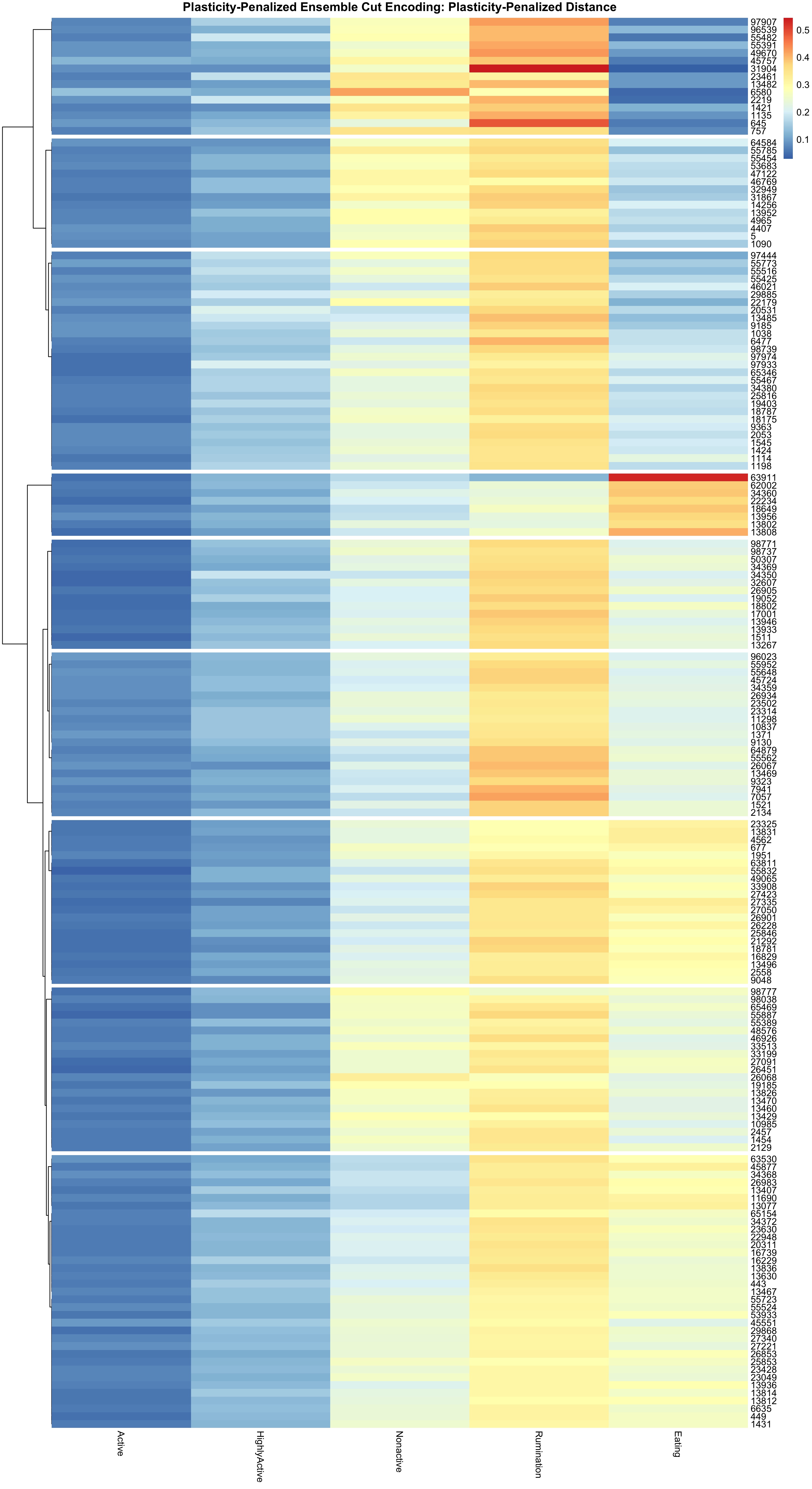

Supplement: Supplementary file 1 [file sensors-22-00001-s001.zip › sensors-1463895-supplementary/OverallTB/EnsembleCut/PPEncode/PWEncode_R9_C0.jpeg]

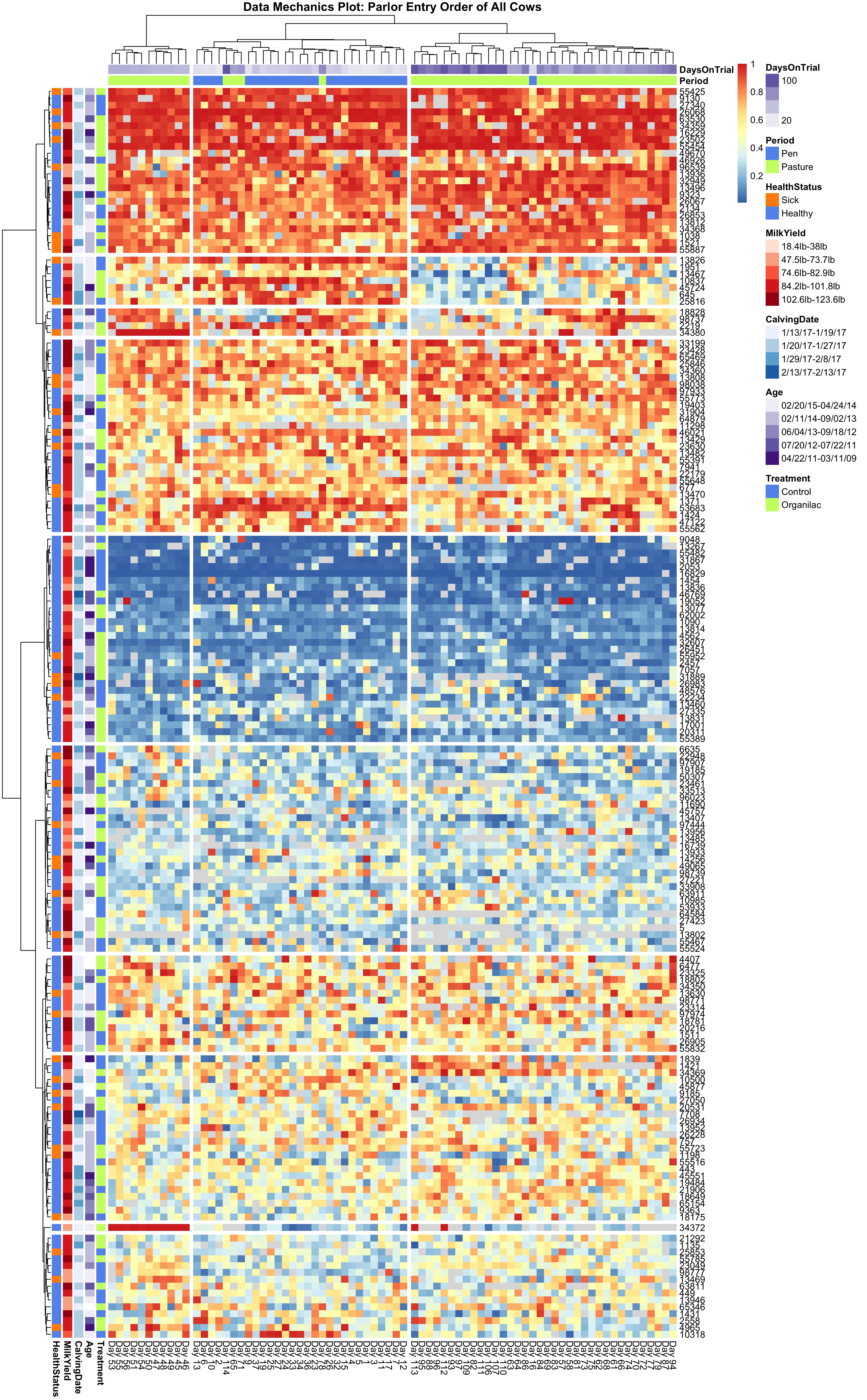

Supplement: Supplementary file 1 [file sensors-22-00001-s001.zip › sensors-1463895-supplementary/OverallTB/MilkOrder/DMPlot_EntryOrder_all .jpeg]

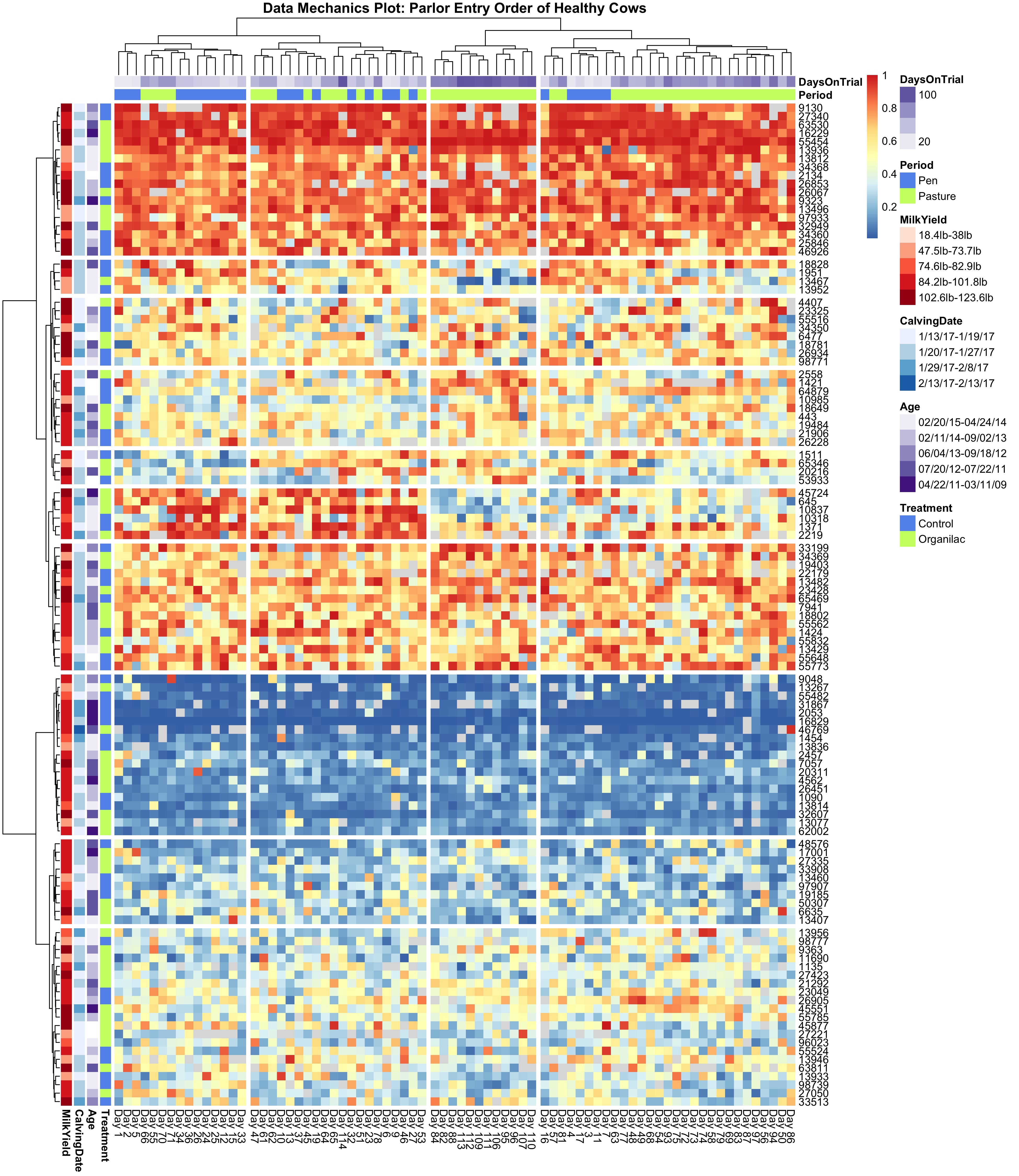

Supplement: Supplementary file 1 [file sensors-22-00001-s001.zip › sensors-1463895-supplementary/OverallTB/MilkOrder/DMPlot_EntryOrder_healthy .jpeg]

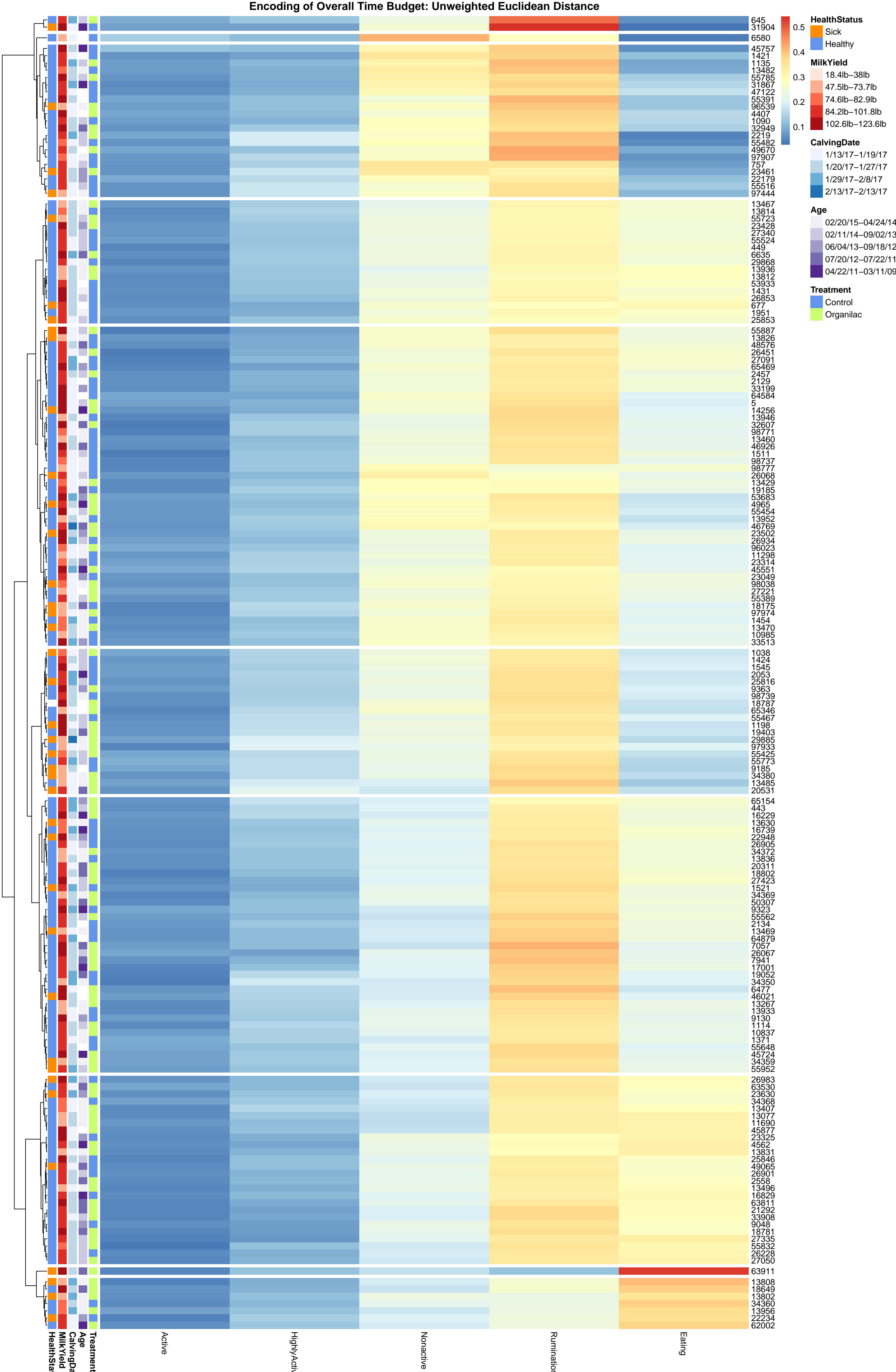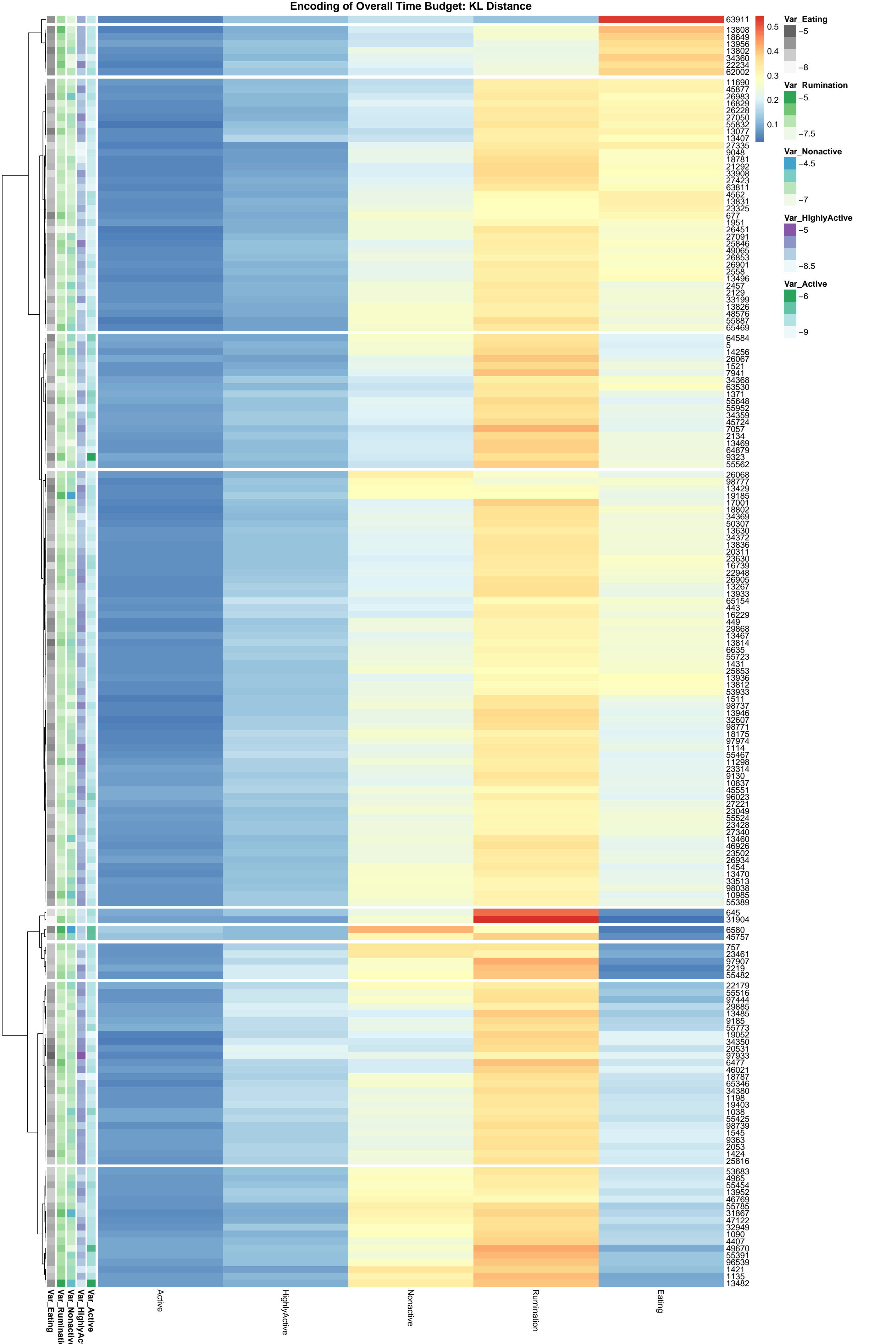

Supplement: Supplementary file 1 [file sensors-22-00001-s001.zip › sensors-1463895-supplementary/OverallTB/OTBEncodings/EncodingSummary.pdf]

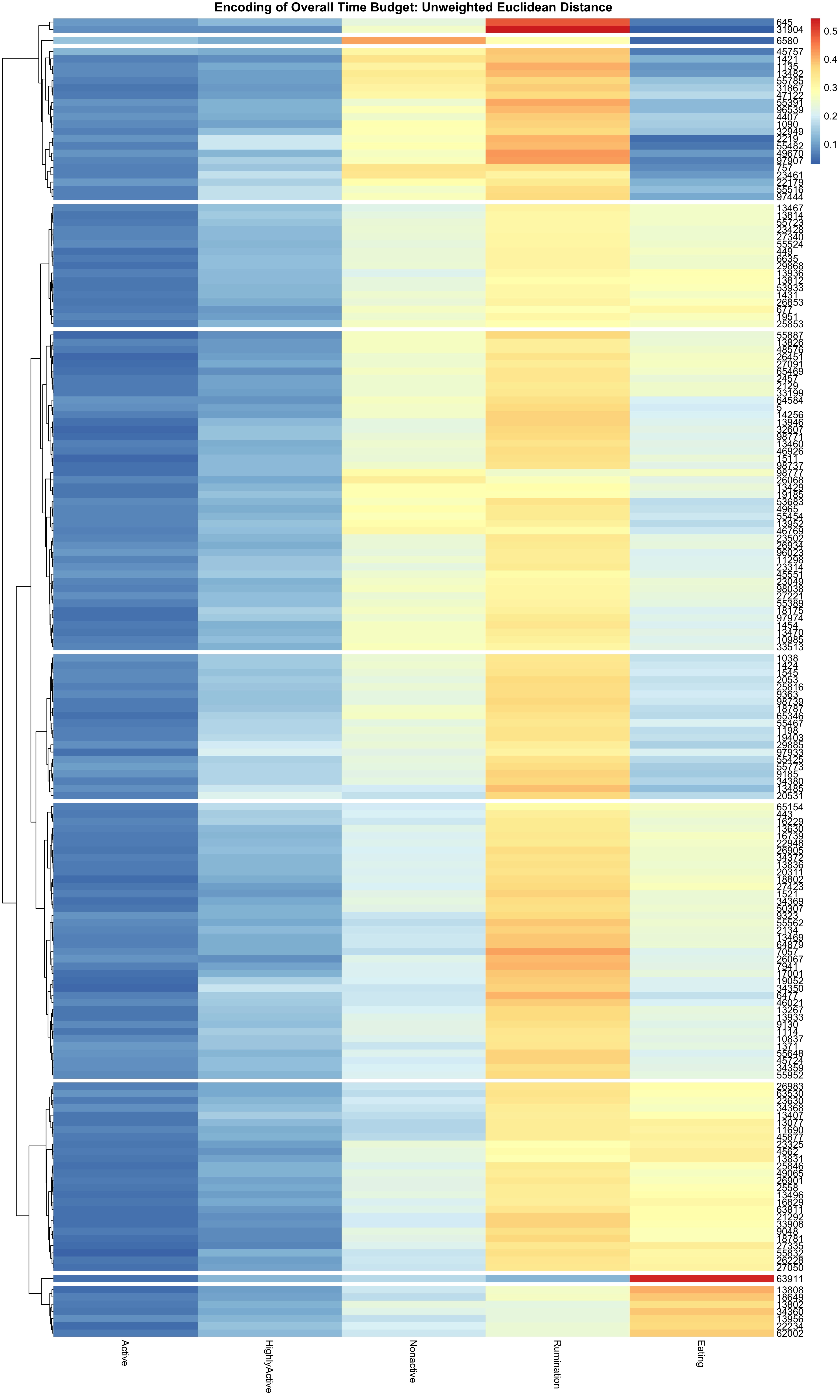

Supplement: Supplementary file 1 [file sensors-22-00001-s001.zip › sensors-1463895-supplementary/OverallTB/OTBEncodings/Euclidean/OverallTB_Eucidean_R10_C0.jpeg]

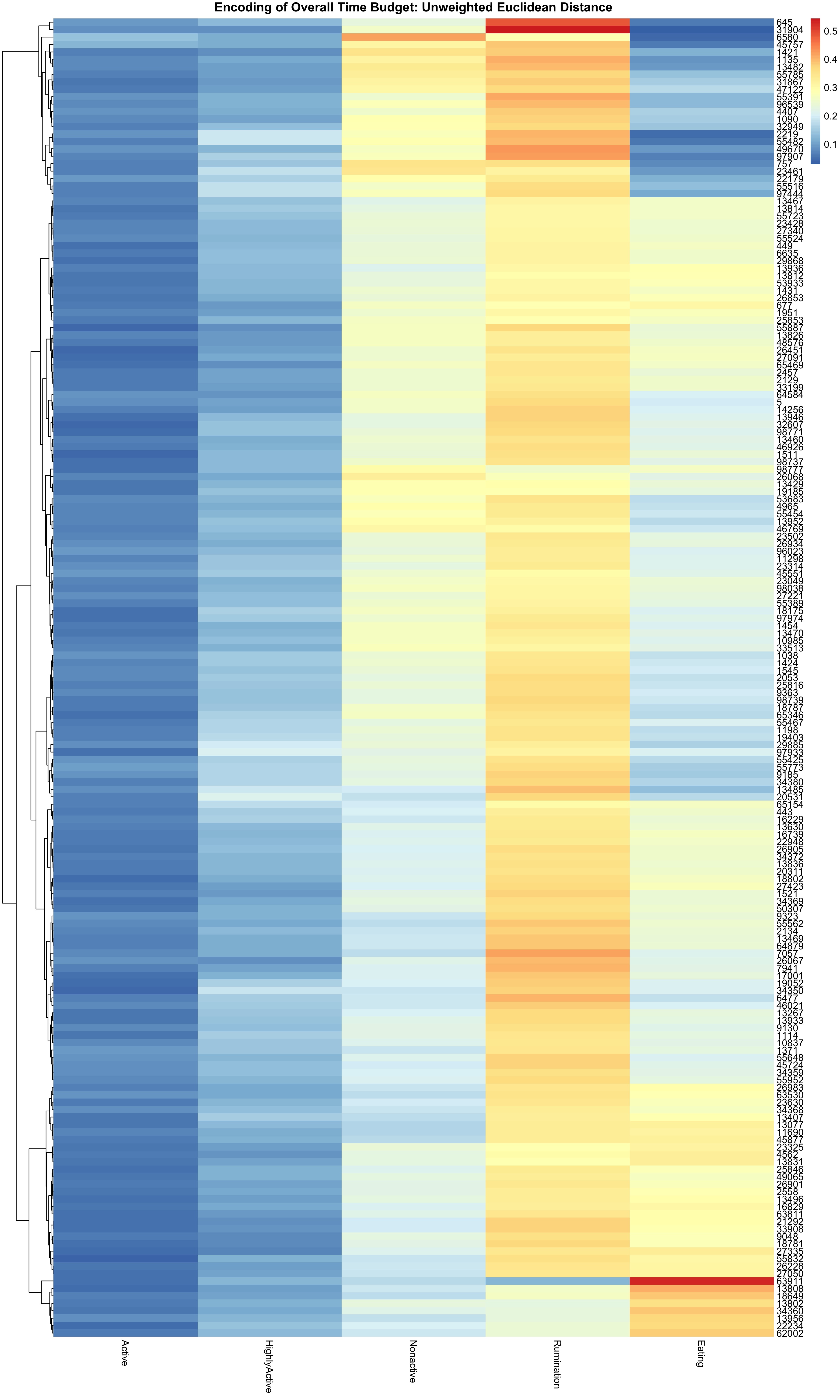

Supplement: Supplementary file 1 [file sensors-22-00001-s001.zip › sensors-1463895-supplementary/OverallTB/OTBEncodings/Euclidean/OverallTB_Eucidean_R1_C0.jpeg]

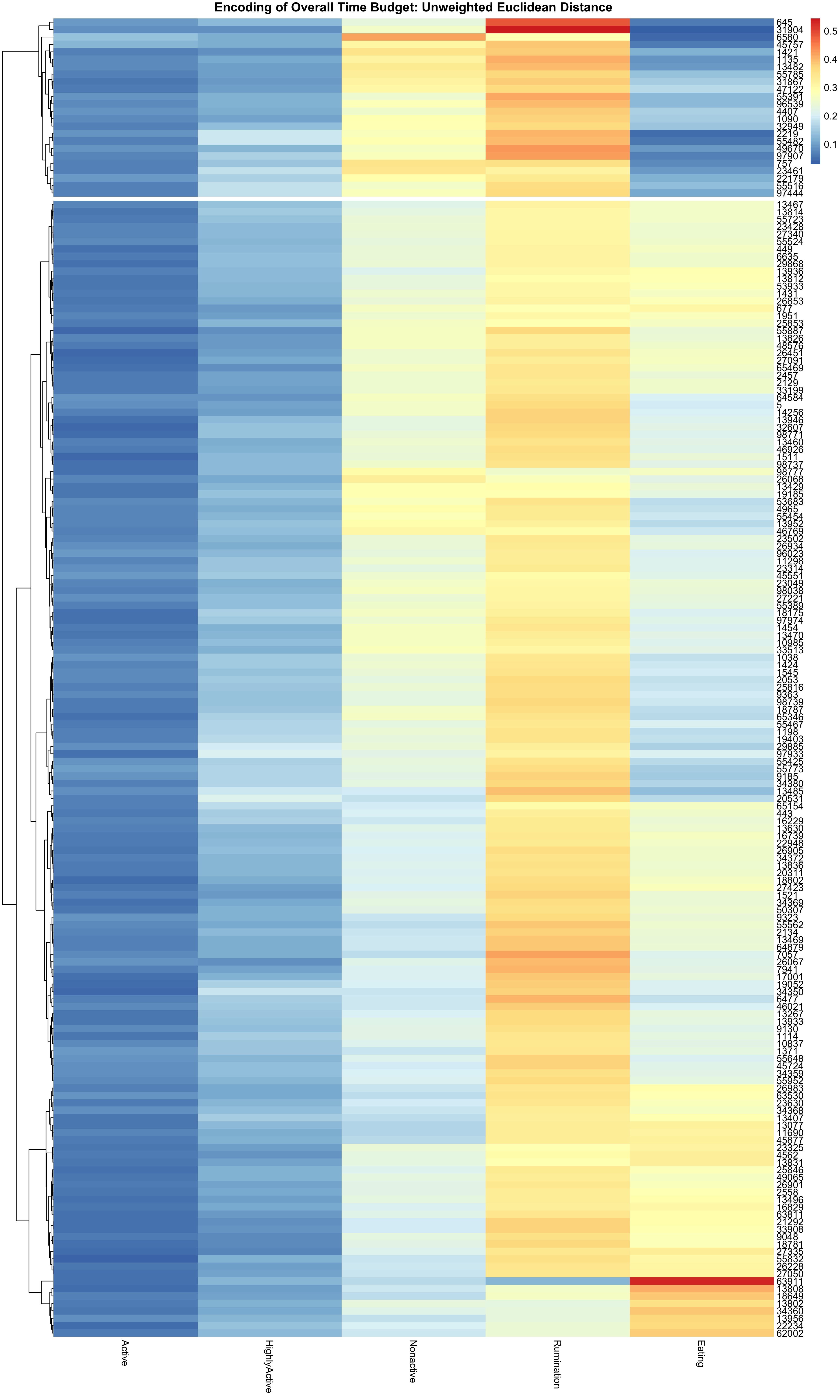

Supplement: Supplementary file 1 [file sensors-22-00001-s001.zip › sensors-1463895-supplementary/OverallTB/OTBEncodings/Euclidean/OverallTB_Eucidean_R2_C0.jpeg]

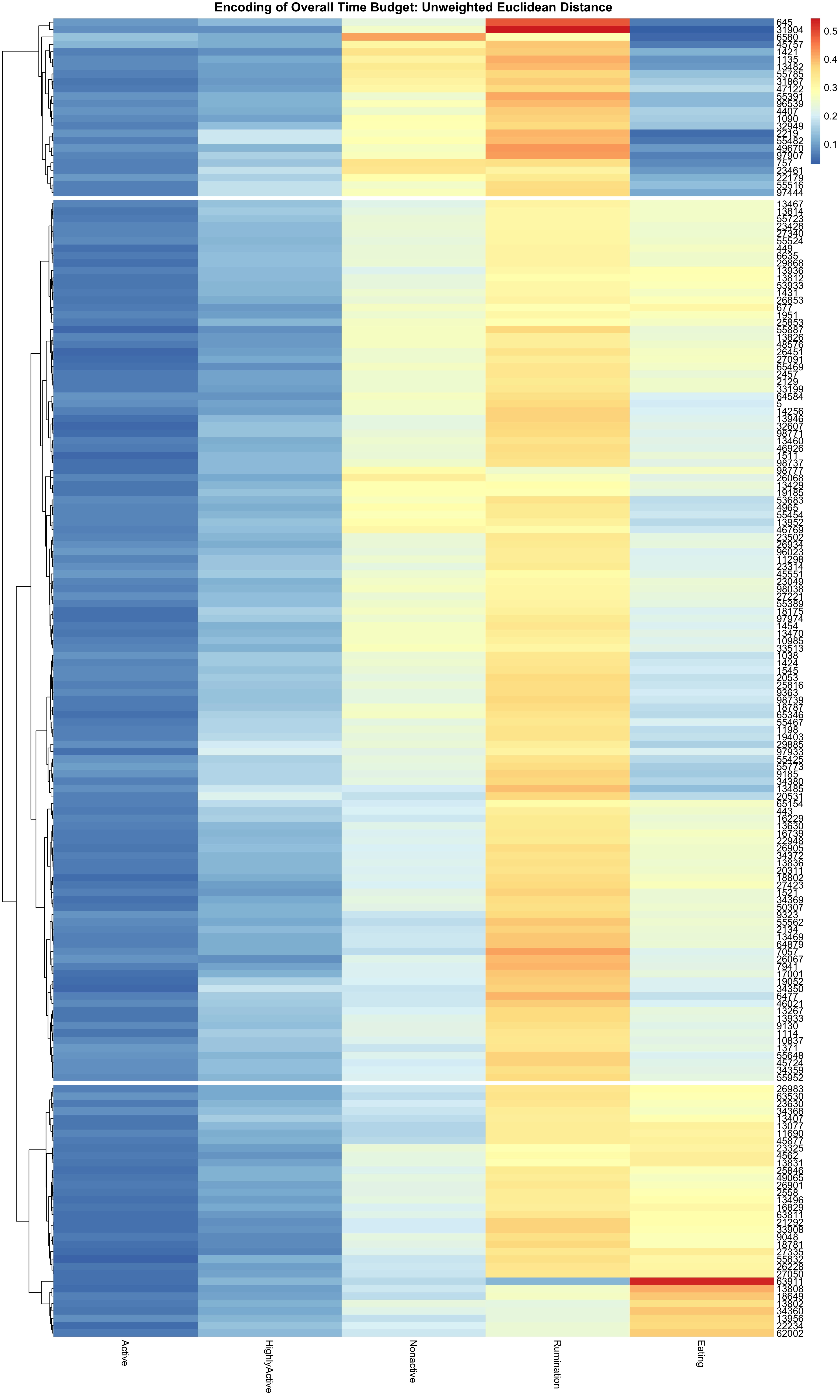

Supplement: Supplementary file 1 [file sensors-22-00001-s001.zip › sensors-1463895-supplementary/OverallTB/OTBEncodings/Euclidean/OverallTB_Eucidean_R3_C0.jpeg]

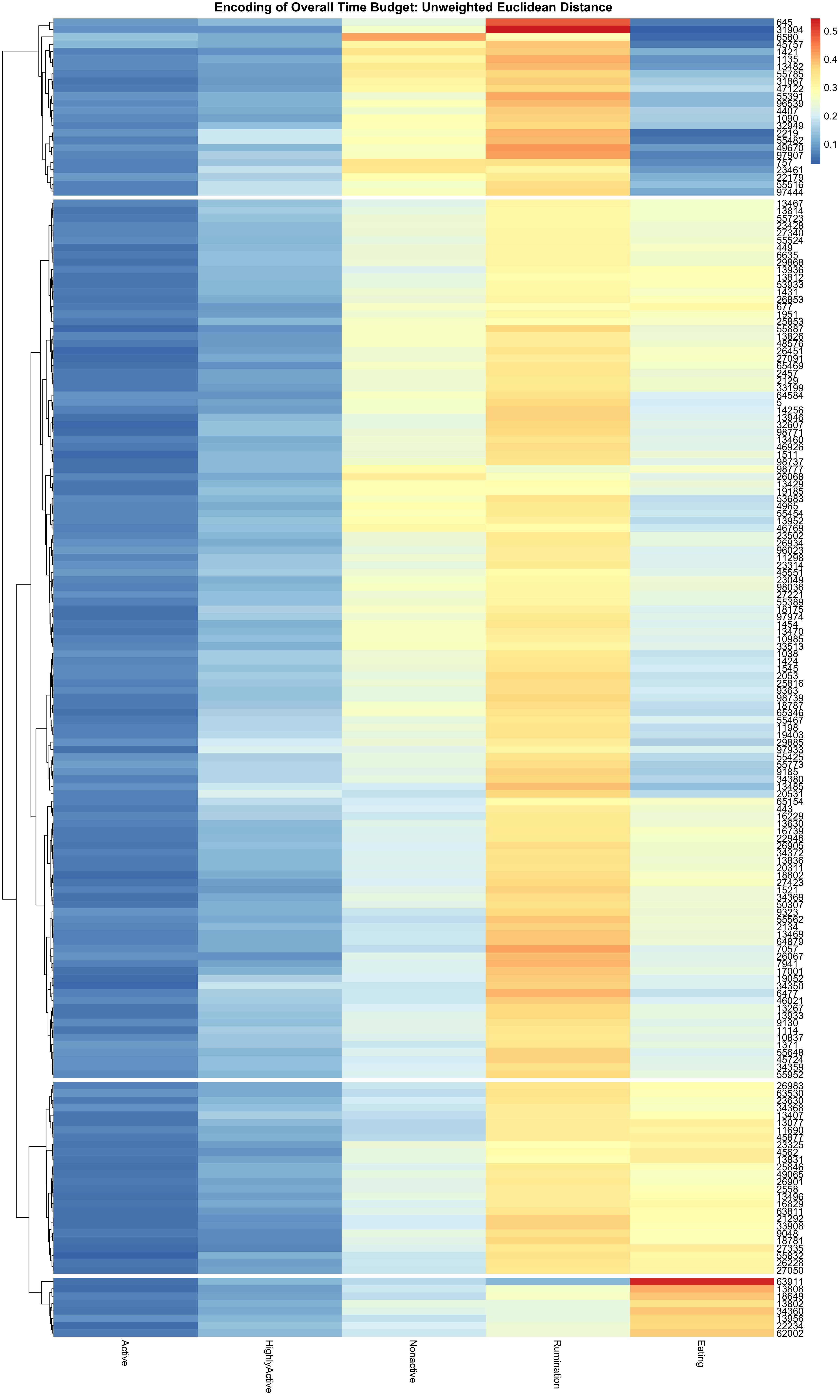

Supplement: Supplementary file 1 [file sensors-22-00001-s001.zip › sensors-1463895-supplementary/OverallTB/OTBEncodings/Euclidean/OverallTB_Eucidean_R4_C0.jpeg]

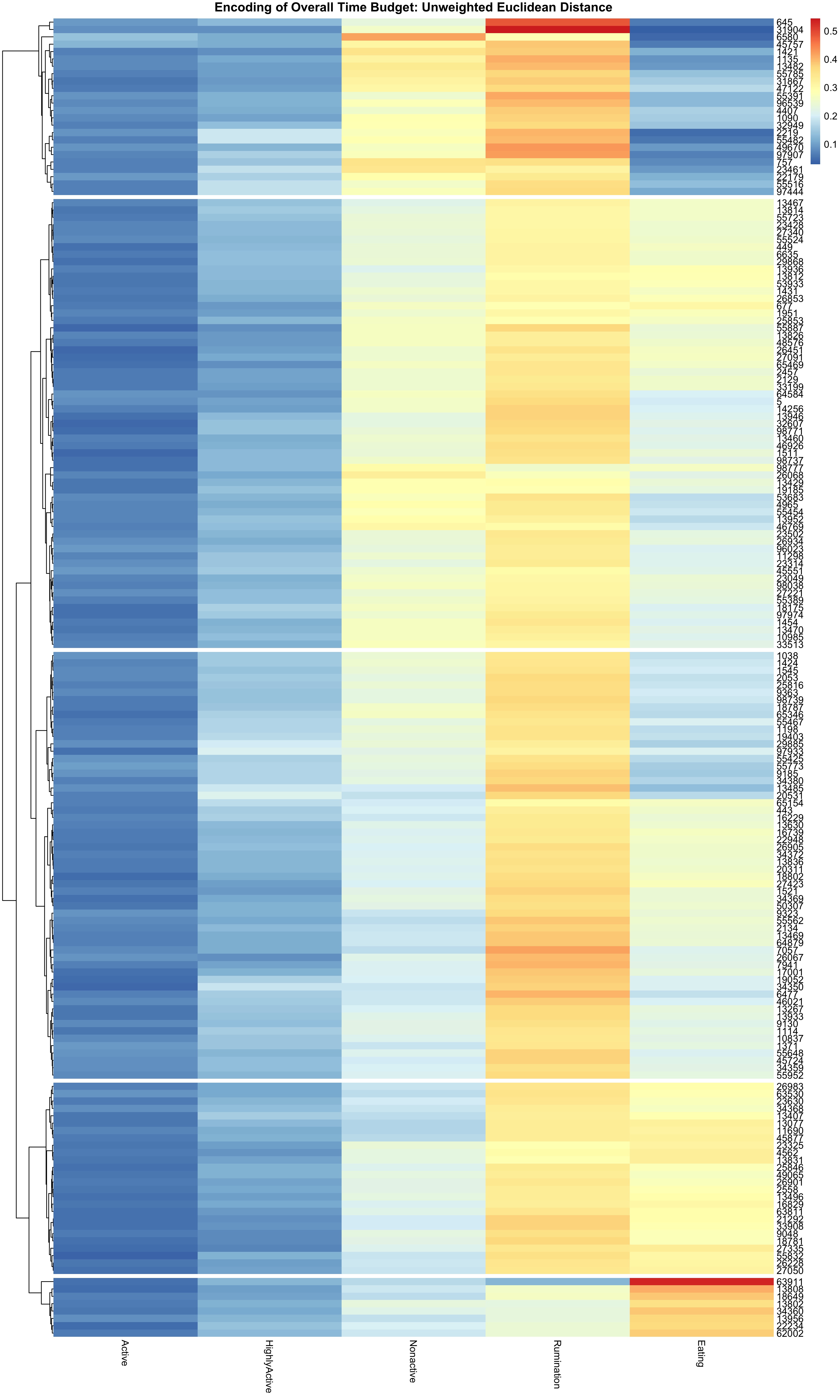

Supplement: Supplementary file 1 [file sensors-22-00001-s001.zip › sensors-1463895-supplementary/OverallTB/OTBEncodings/Euclidean/OverallTB_Eucidean_R5_C0.jpeg]

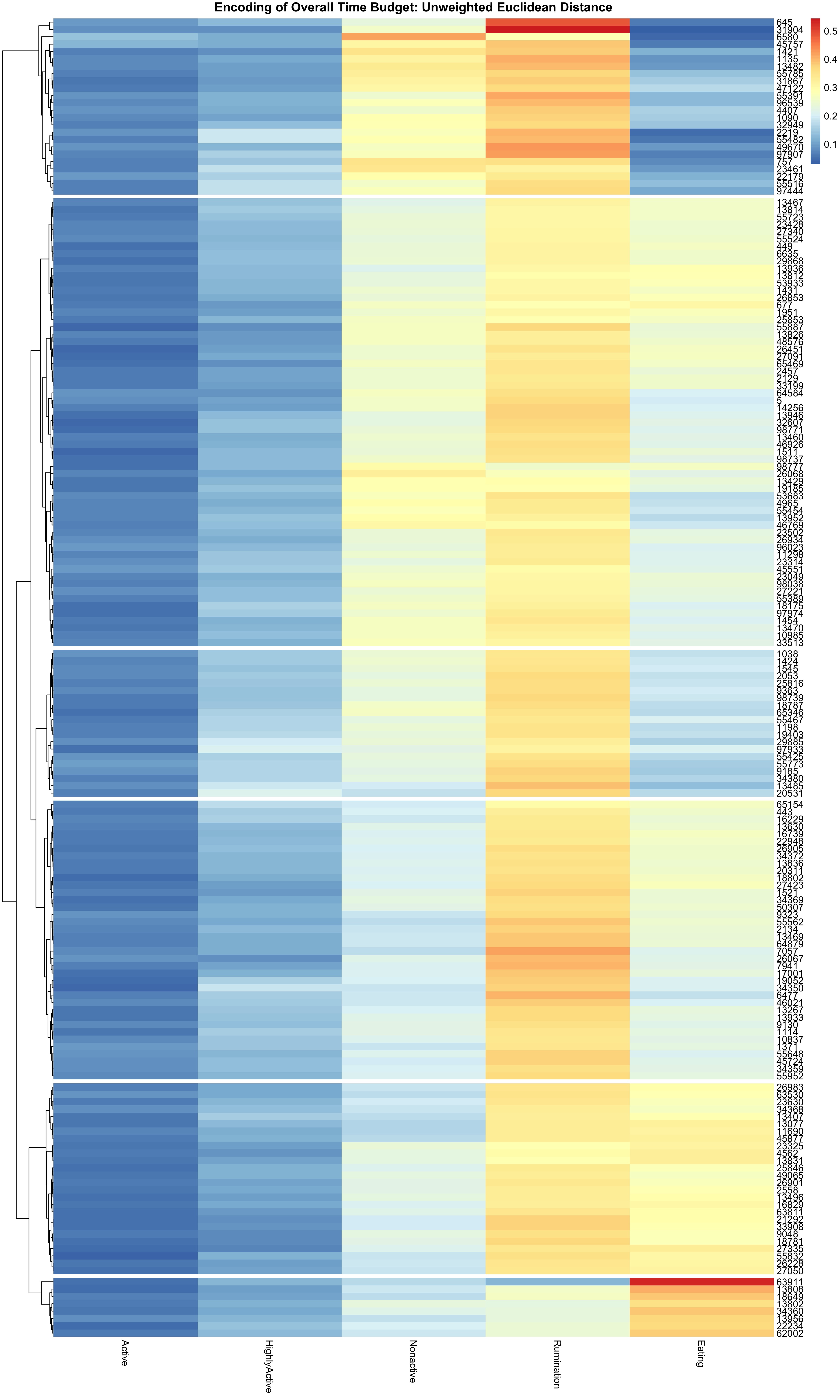

Supplement: Supplementary file 1 [file sensors-22-00001-s001.zip › sensors-1463895-supplementary/OverallTB/OTBEncodings/Euclidean/OverallTB_Eucidean_R6_C0.jpeg]

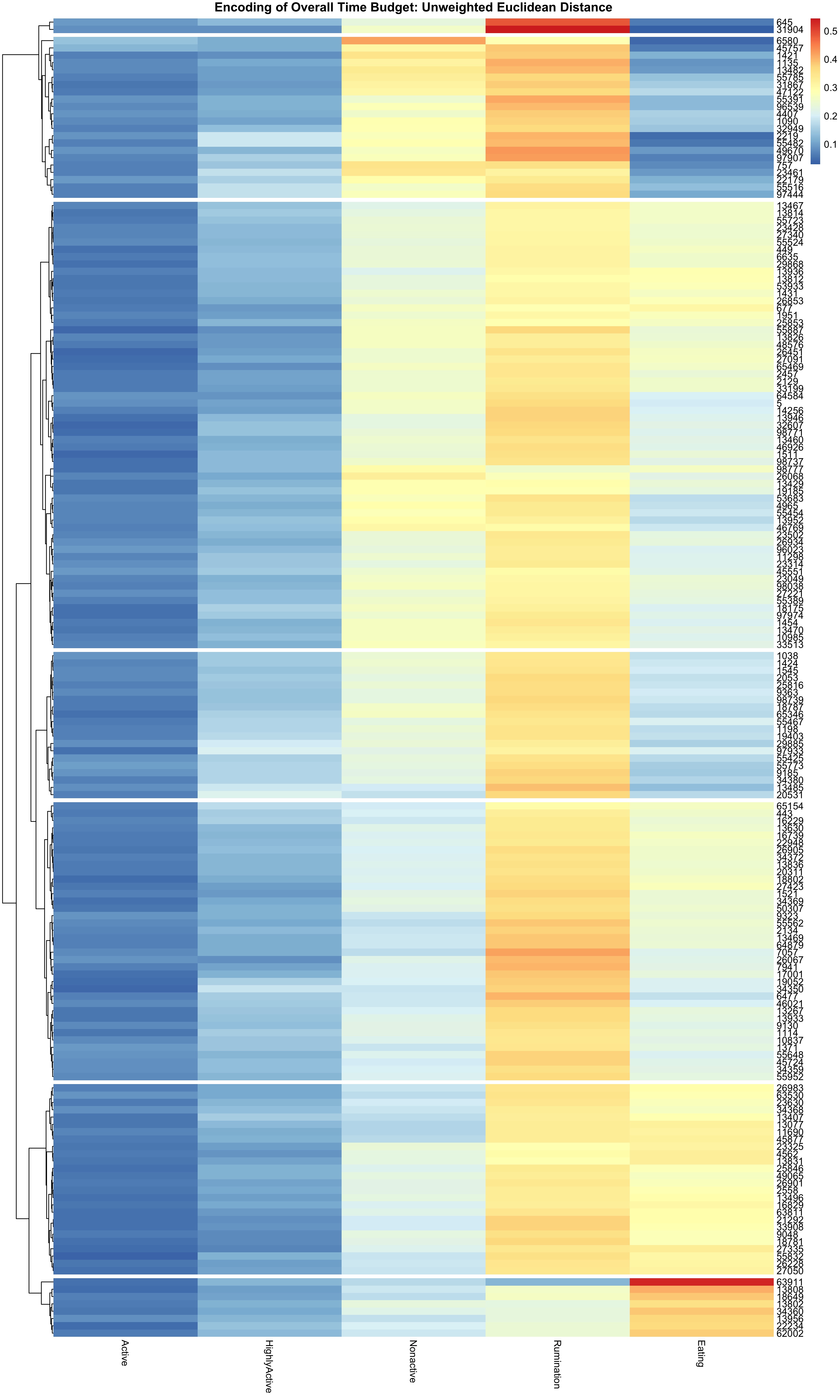

Supplement: Supplementary file 1 [file sensors-22-00001-s001.zip › sensors-1463895-supplementary/OverallTB/OTBEncodings/Euclidean/OverallTB_Eucidean_R7_C0.jpeg]

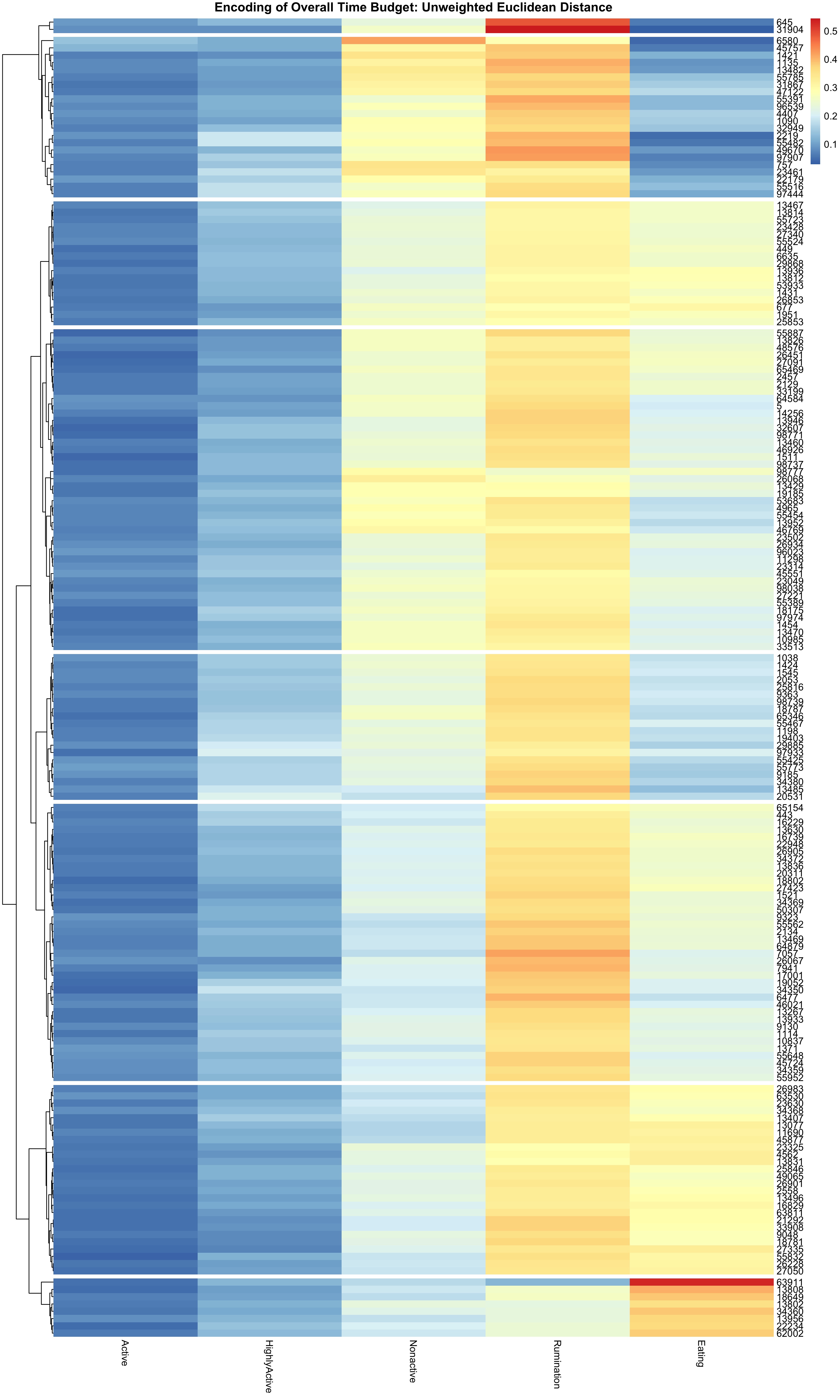

Supplement: Supplementary file 1 [file sensors-22-00001-s001.zip › sensors-1463895-supplementary/OverallTB/OTBEncodings/Euclidean/OverallTB_Eucidean_R8_C0.jpeg]

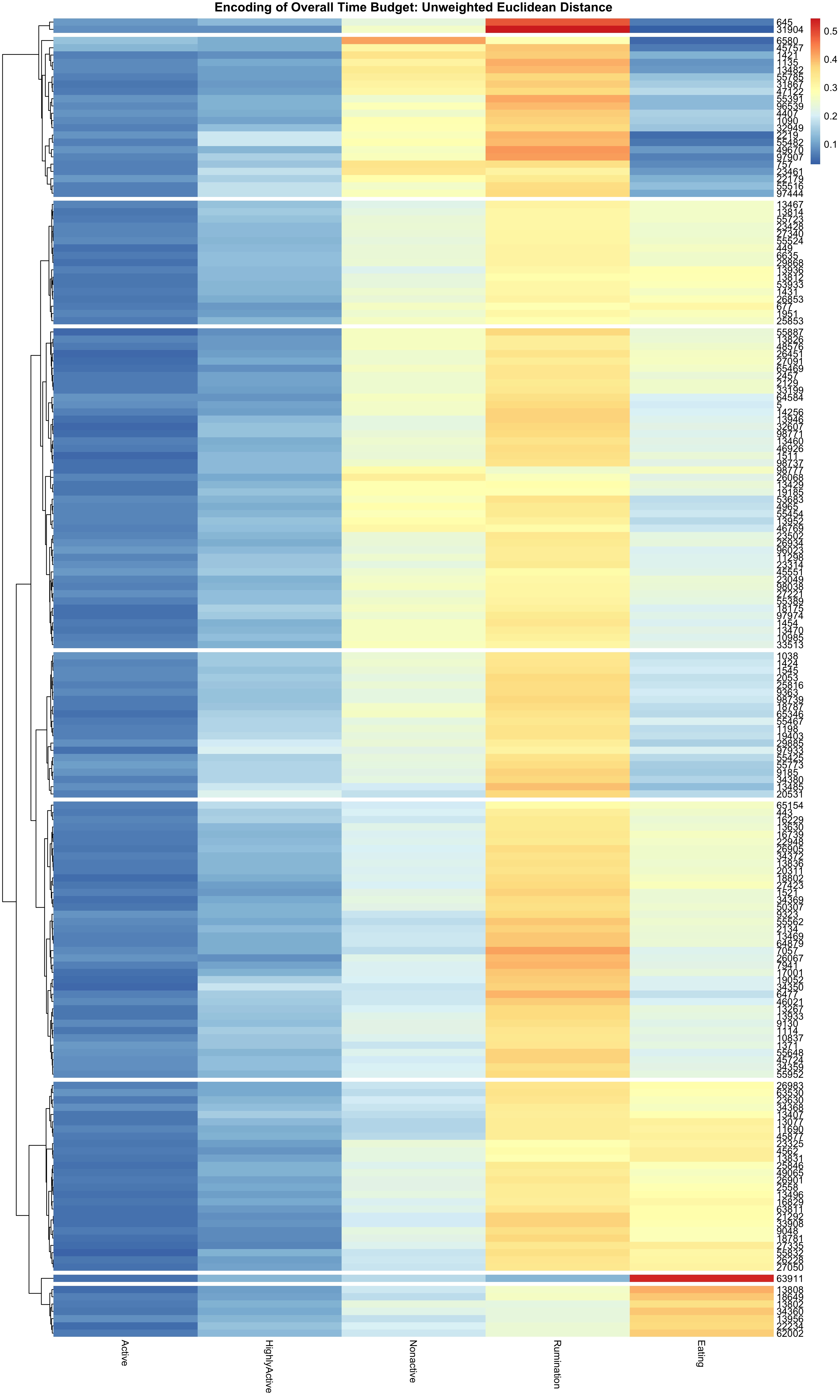

Supplement: Supplementary file 1 [file sensors-22-00001-s001.zip › sensors-1463895-supplementary/OverallTB/OTBEncodings/Euclidean/OverallTB_Eucidean_R9_C0.jpeg]

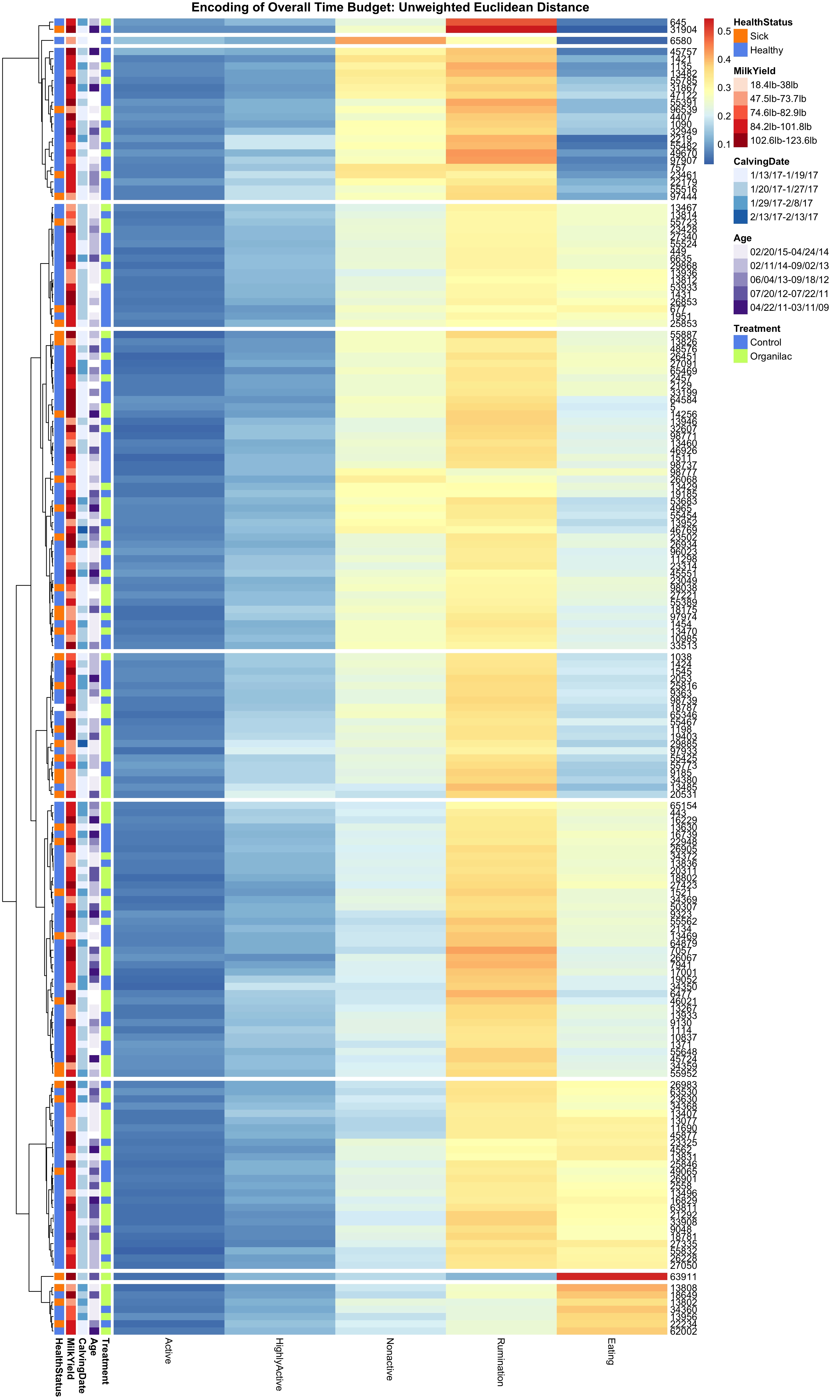

Supplement: Supplementary file 1 [file sensors-22-00001-s001.zip › sensors-1463895-supplementary/OverallTB/OTBEncodings/Euclidean/_OverallTB_Eucidean_AuxVar_R10_C0.jpeg]

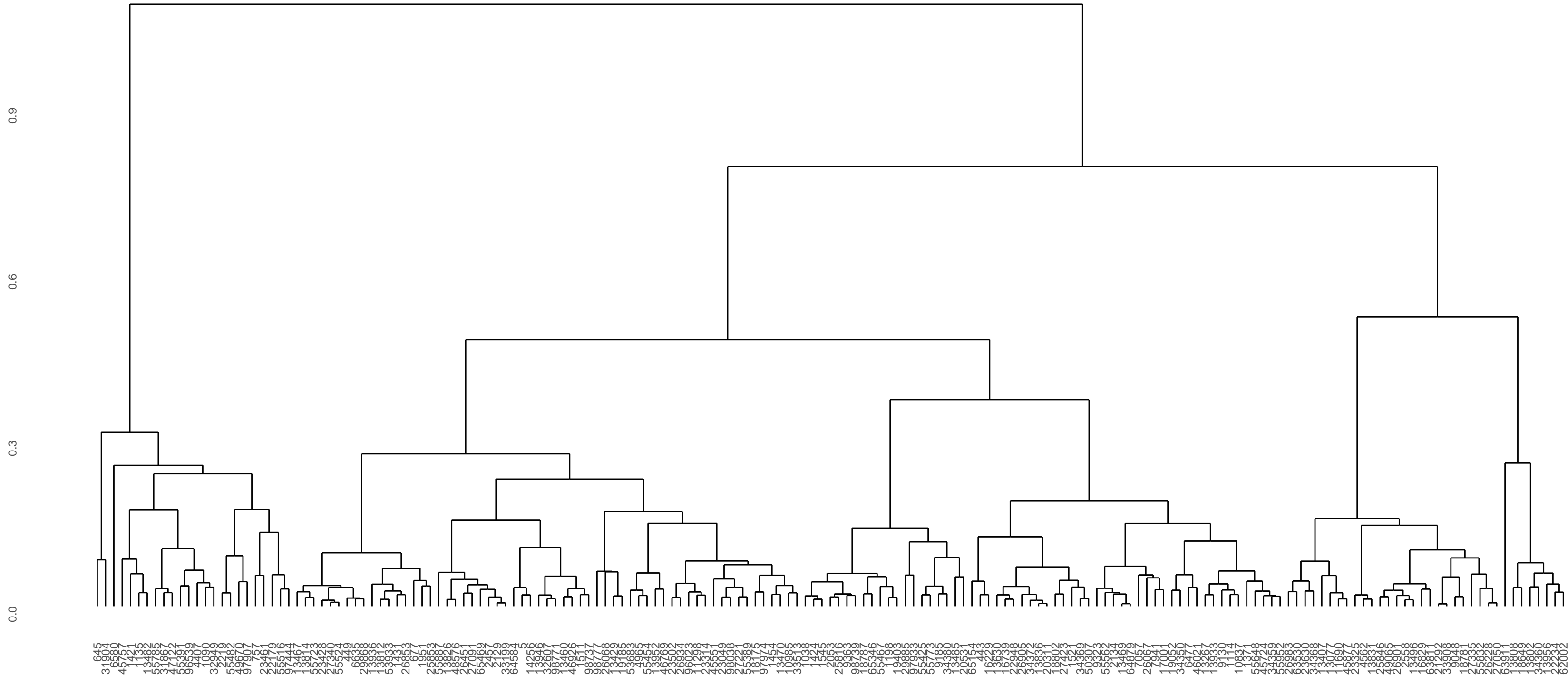

Supplement: Supplementary file 1 [file sensors-22-00001-s001.zip › sensors-1463895-supplementary/OverallTB/OTBEncodings/Euclidean/_OverallTB_Eucidean_TBVar_Dendrogram_.pdf]

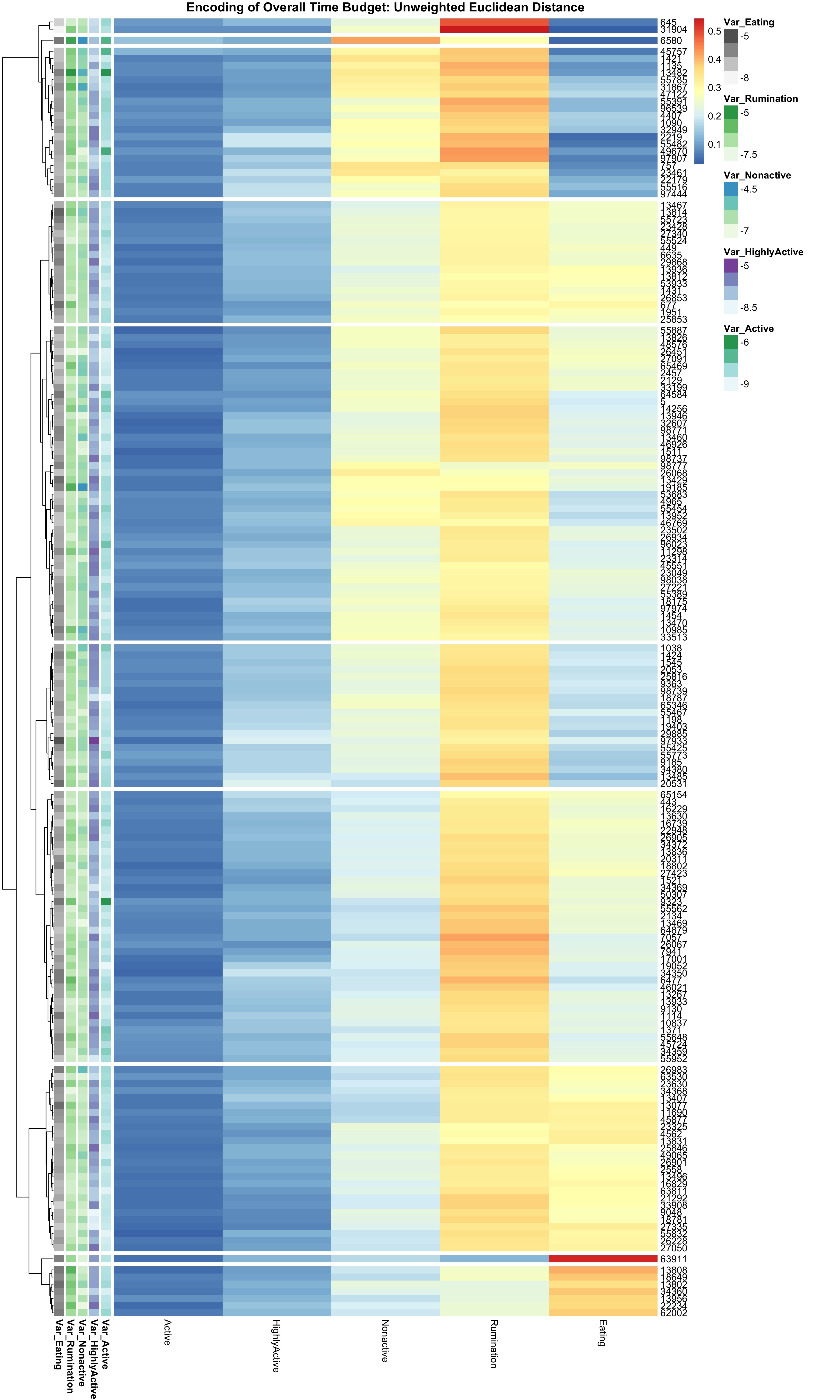

Supplement: Supplementary file 1 [file sensors-22-00001-s001.zip › sensors-1463895-supplementary/OverallTB/OTBEncodings/Euclidean/_OverallTB_Eucidean_TBVar_R10_C0.jpeg]

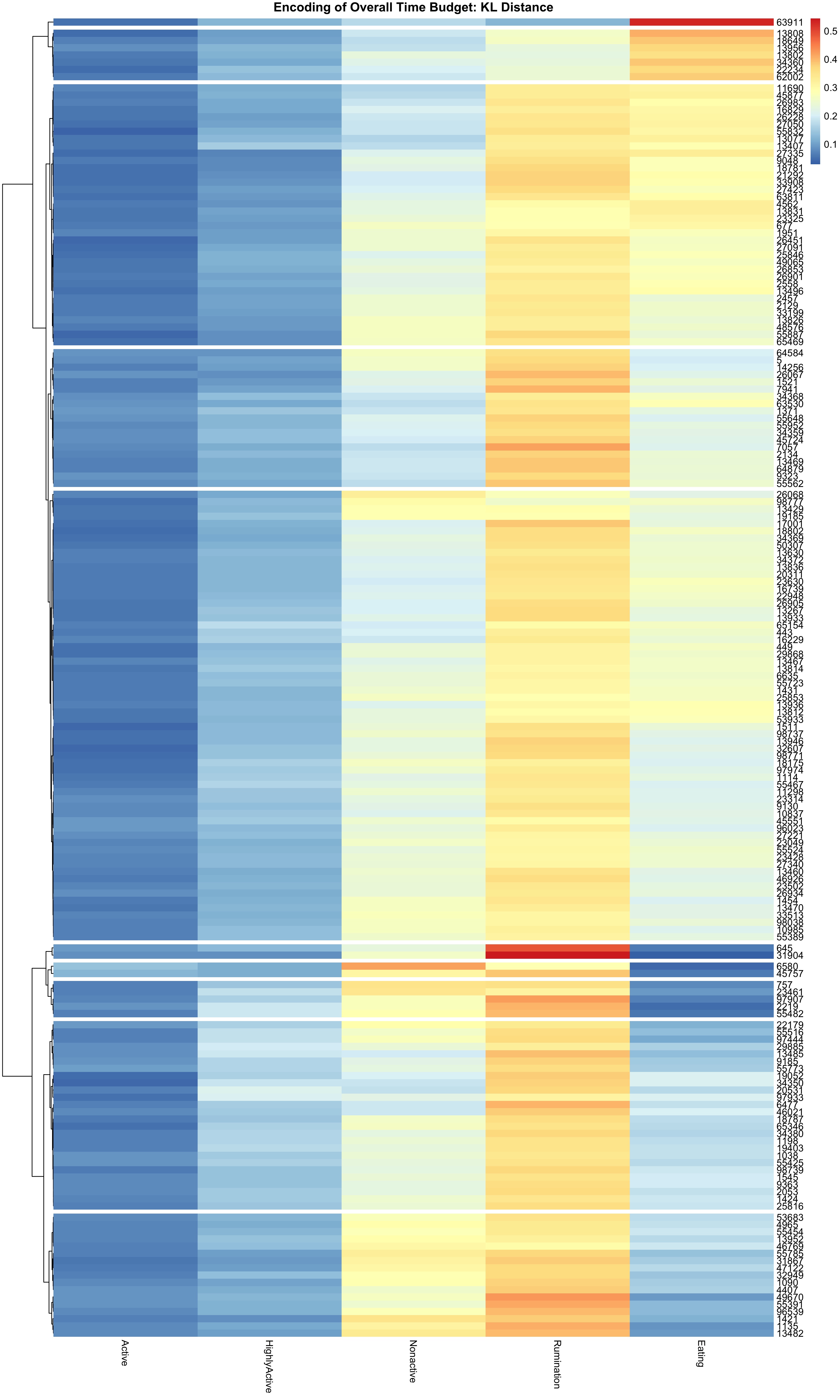

Supplement: Supplementary file 1 [file sensors-22-00001-s001.zip › sensors-1463895-supplementary/OverallTB/OTBEncodings/KLDivergence/OverallTB_KLD_R10_C0.jpeg]

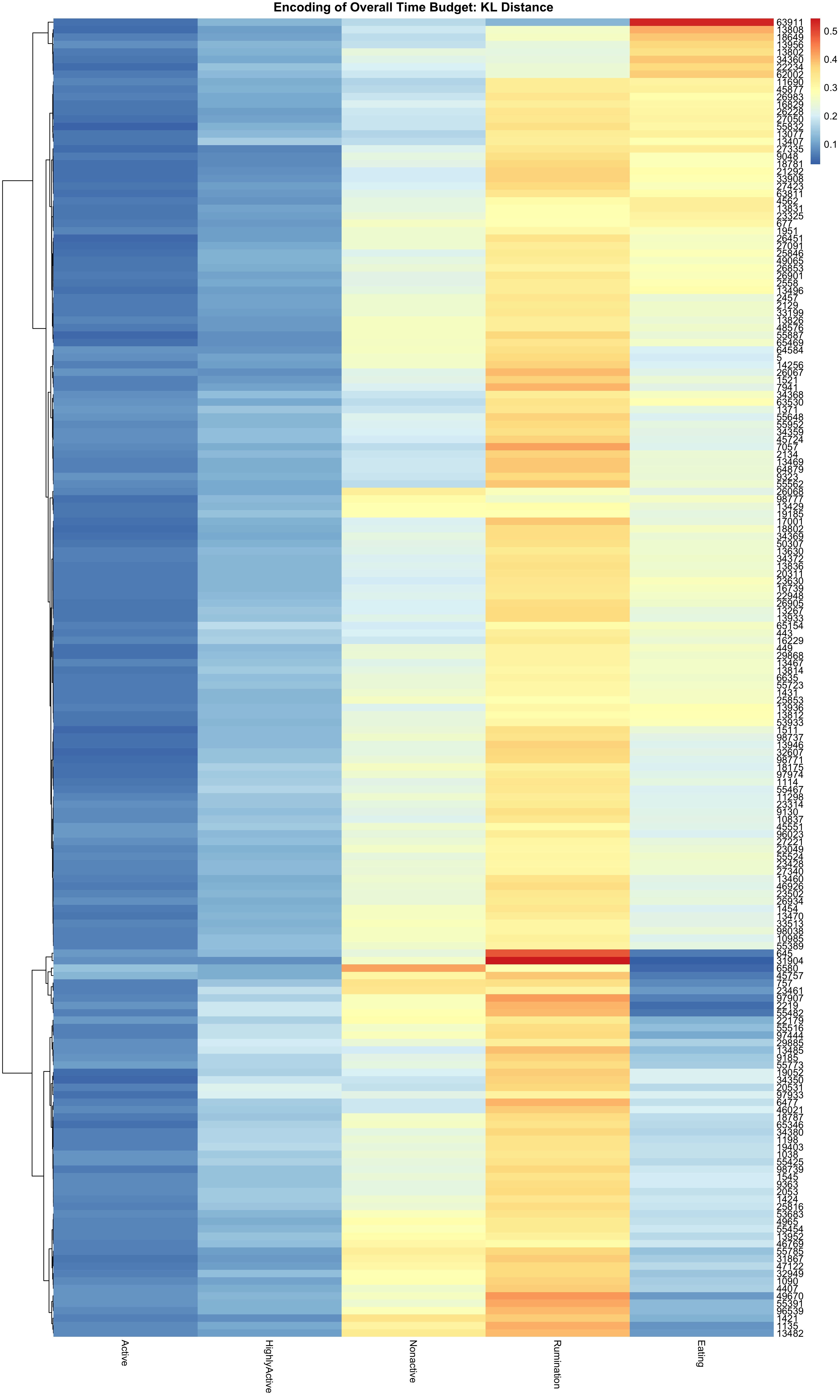

Supplement: Supplementary file 1 [file sensors-22-00001-s001.zip › sensors-1463895-supplementary/OverallTB/OTBEncodings/KLDivergence/OverallTB_KLD_R1_C0.jpeg]

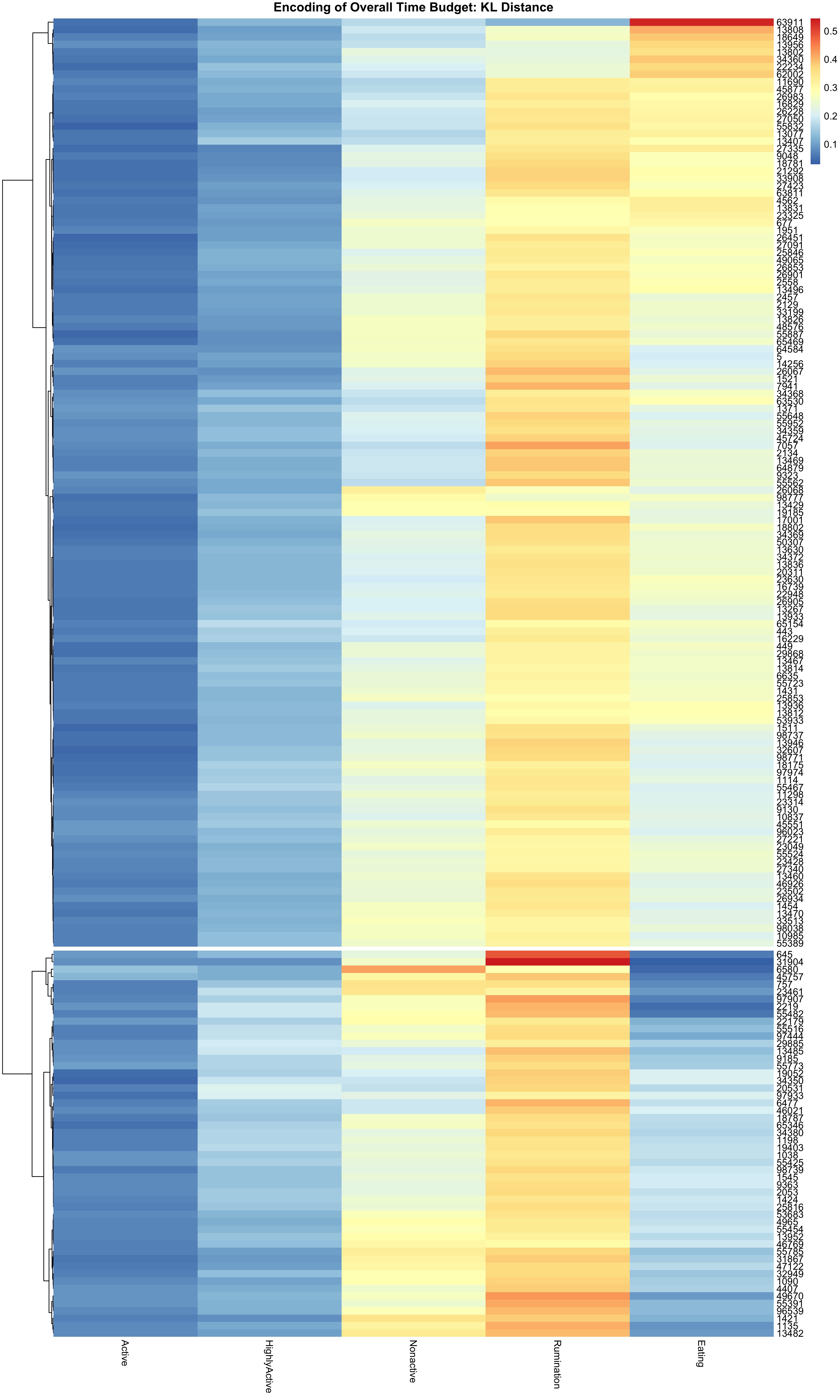

Supplement: Supplementary file 1 [file sensors-22-00001-s001.zip › sensors-1463895-supplementary/OverallTB/OTBEncodings/KLDivergence/OverallTB_KLD_R2_C0.jpeg]

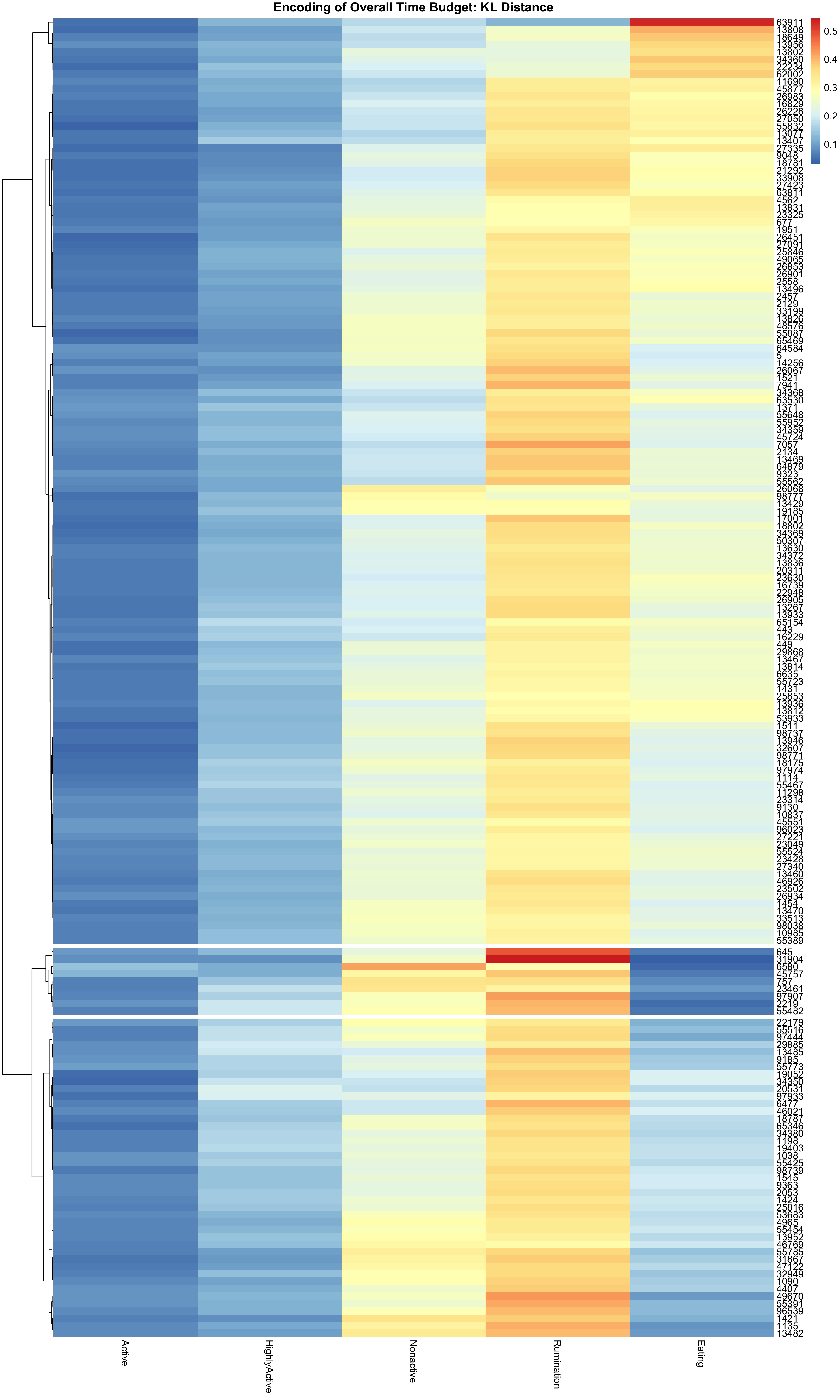

Supplement: Supplementary file 1 [file sensors-22-00001-s001.zip › sensors-1463895-supplementary/OverallTB/OTBEncodings/KLDivergence/OverallTB_KLD_R3_C0.jpeg]

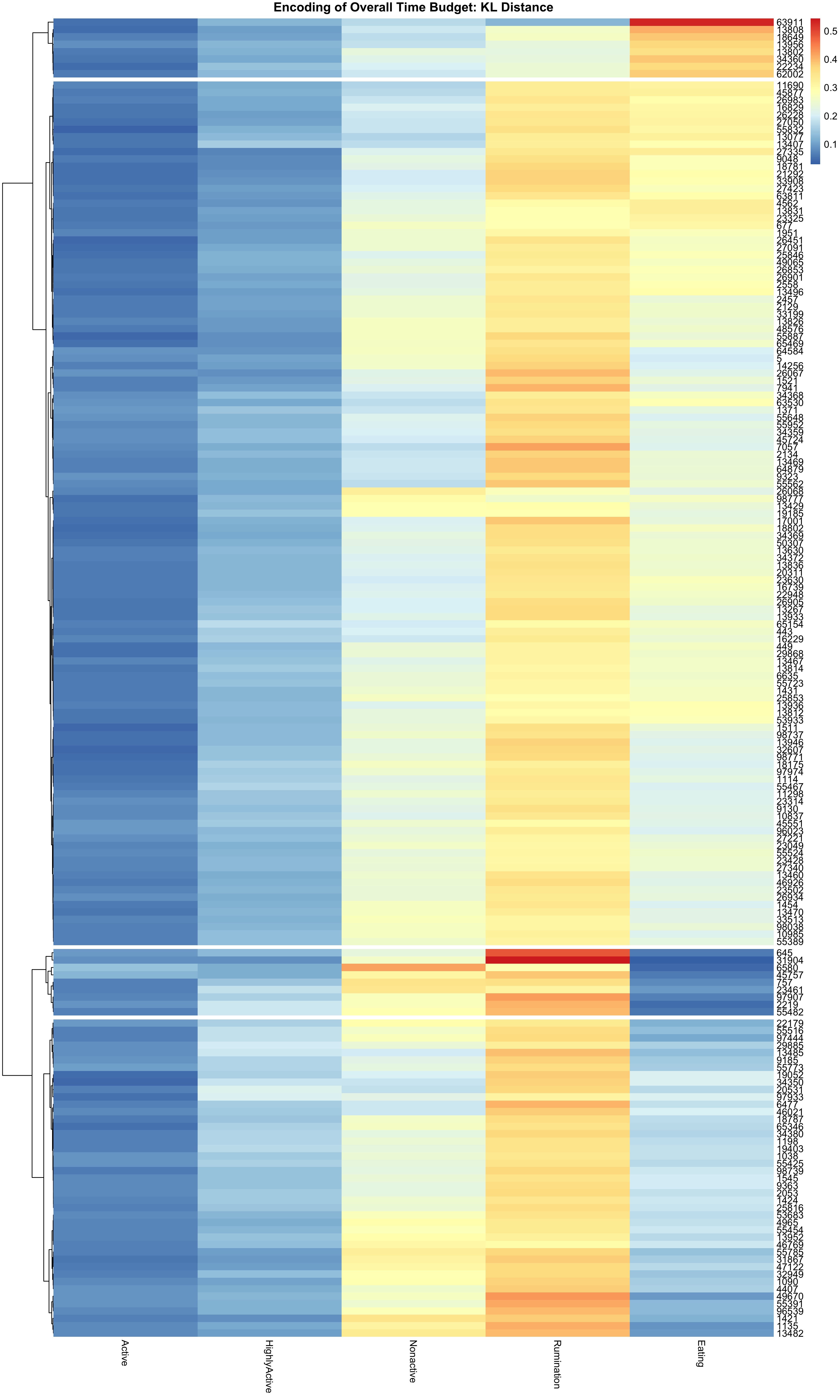

Supplement: Supplementary file 1 [file sensors-22-00001-s001.zip › sensors-1463895-supplementary/OverallTB/OTBEncodings/KLDivergence/OverallTB_KLD_R4_C0.jpeg]

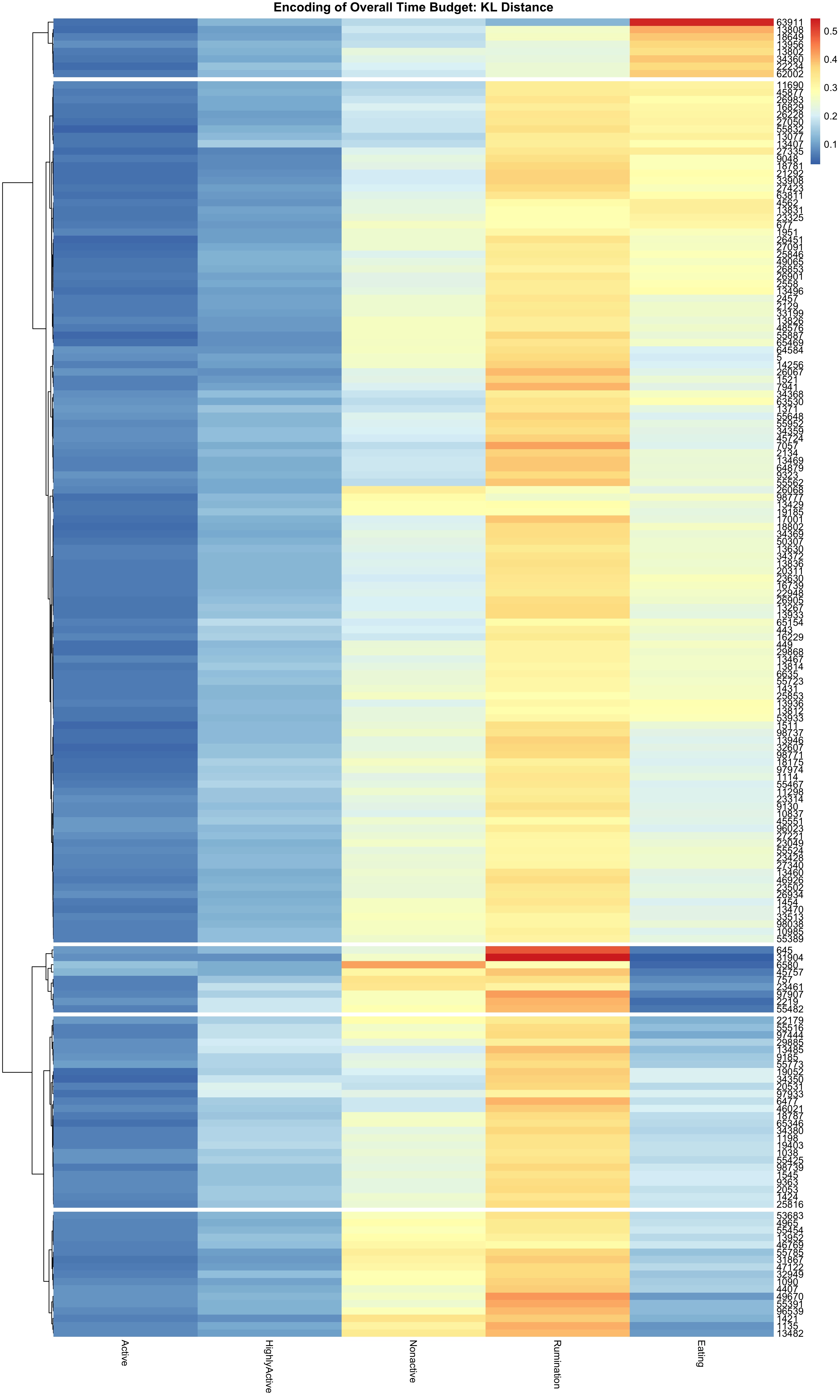

Supplement: Supplementary file 1 [file sensors-22-00001-s001.zip › sensors-1463895-supplementary/OverallTB/OTBEncodings/KLDivergence/OverallTB_KLD_R5_C0.jpeg]

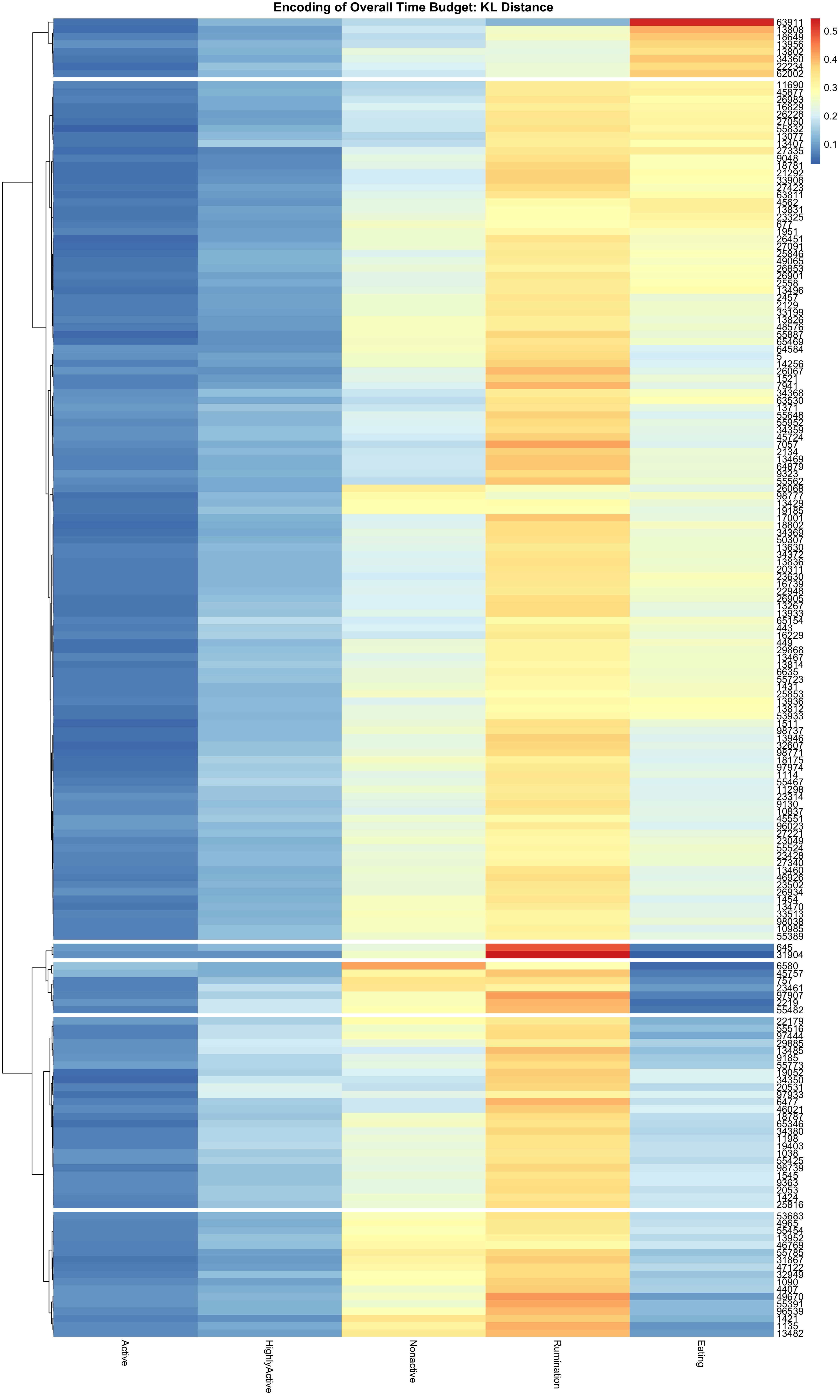

Supplement: Supplementary file 1 [file sensors-22-00001-s001.zip › sensors-1463895-supplementary/OverallTB/OTBEncodings/KLDivergence/OverallTB_KLD_R6_C0.jpeg]

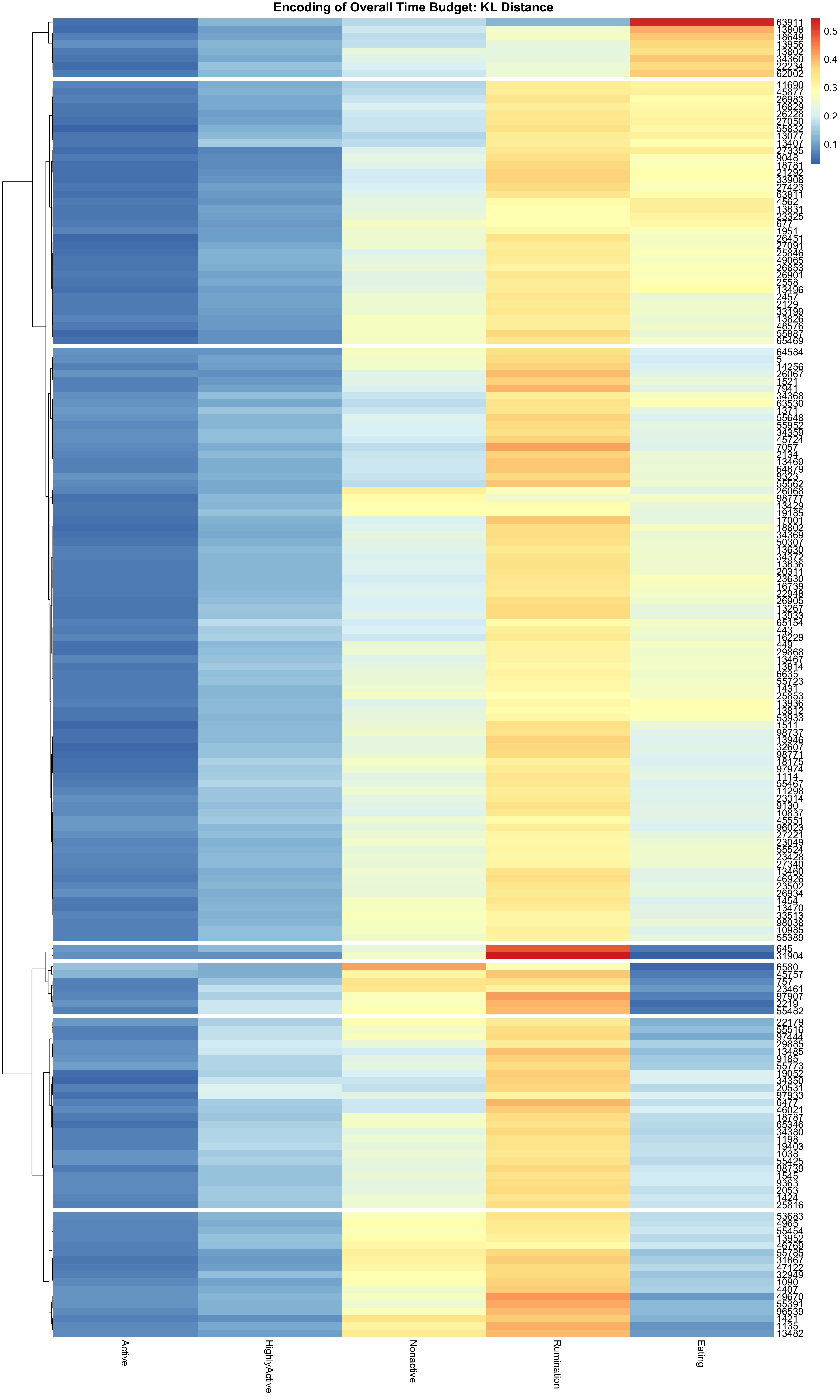

Supplement: Supplementary file 1 [file sensors-22-00001-s001.zip › sensors-1463895-supplementary/OverallTB/OTBEncodings/KLDivergence/OverallTB_KLD_R7_C0.jpeg]

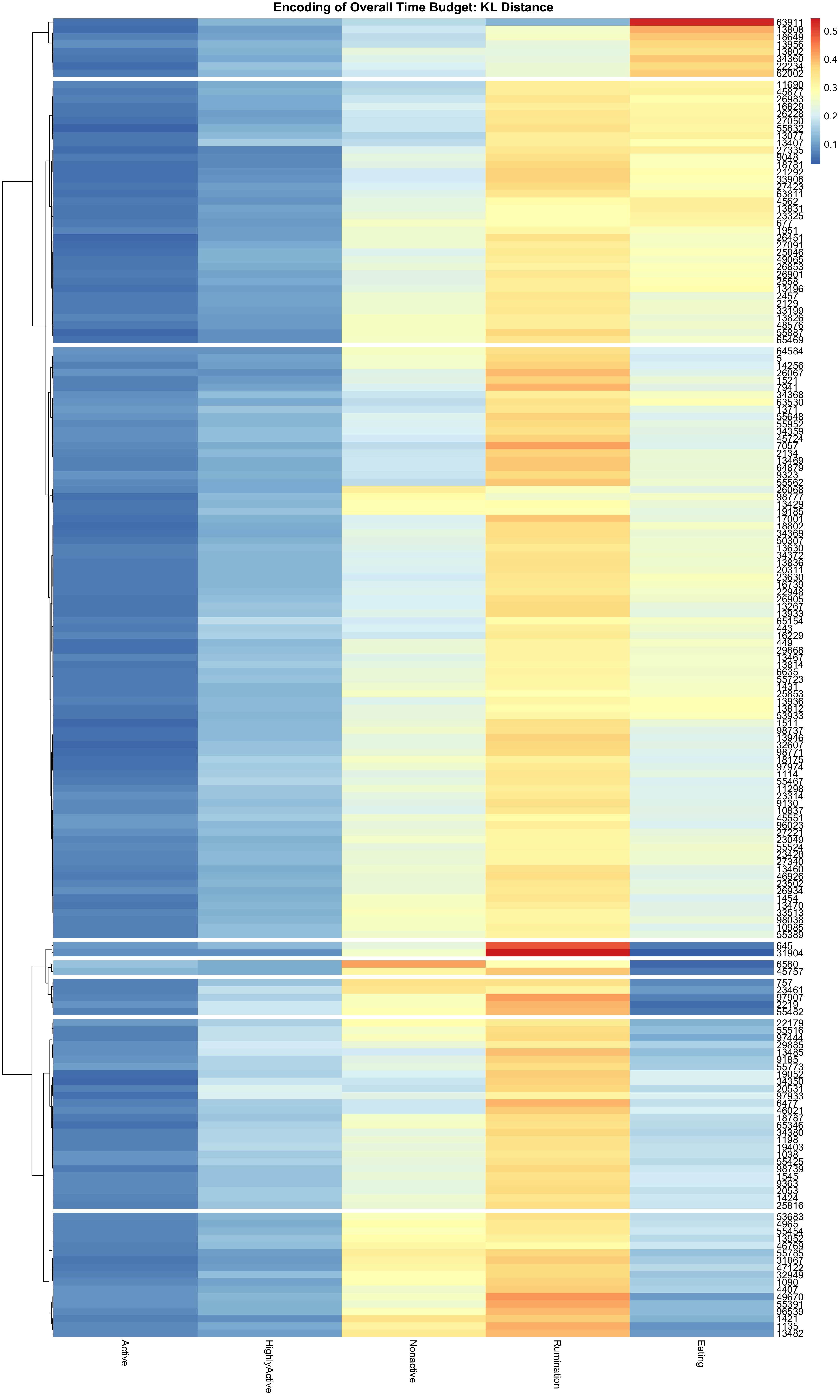

Supplement: Supplementary file 1 [file sensors-22-00001-s001.zip › sensors-1463895-supplementary/OverallTB/OTBEncodings/KLDivergence/OverallTB_KLD_R8_C0.jpeg]

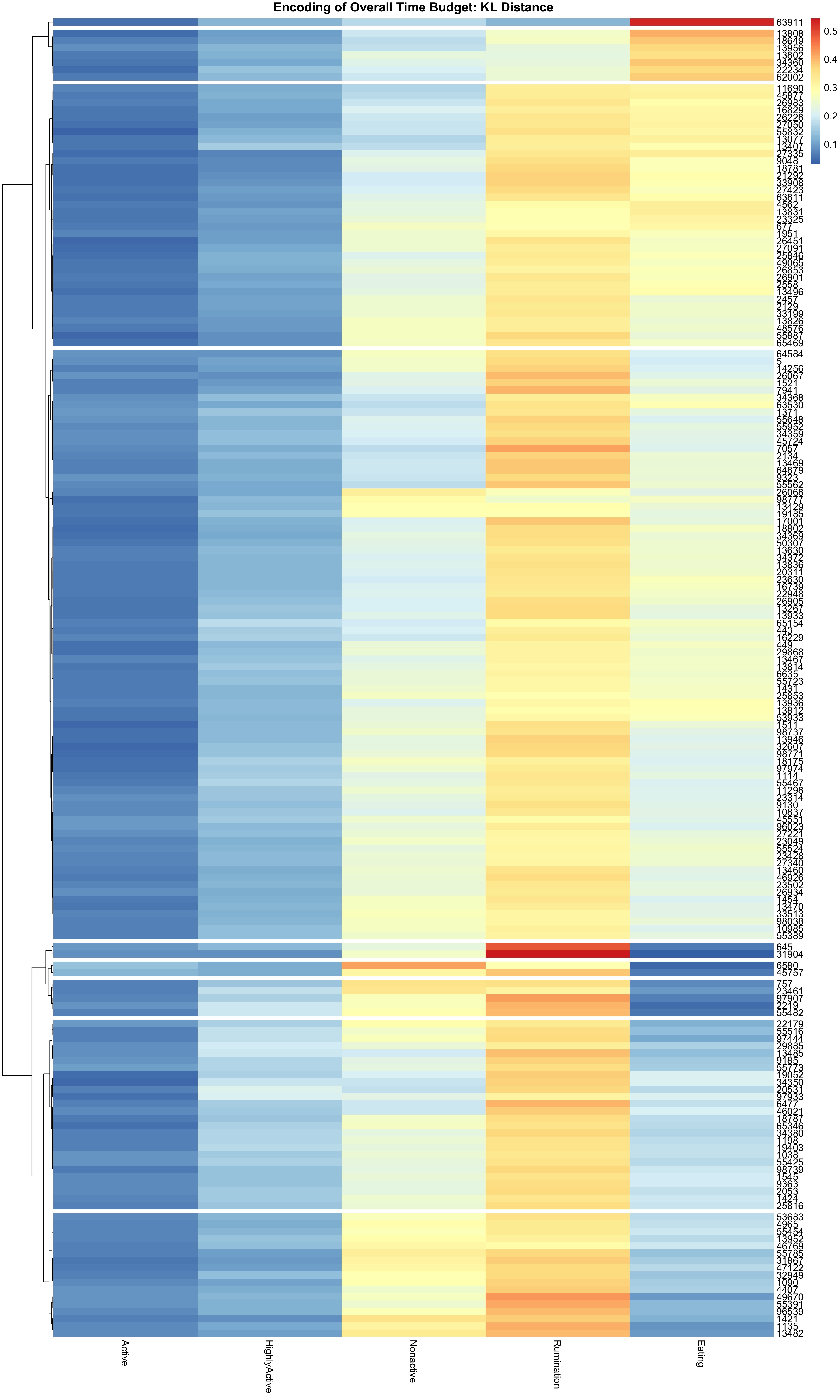

Supplement: Supplementary file 1 [file sensors-22-00001-s001.zip › sensors-1463895-supplementary/OverallTB/OTBEncodings/KLDivergence/OverallTB_KLD_R9_C0.jpeg]

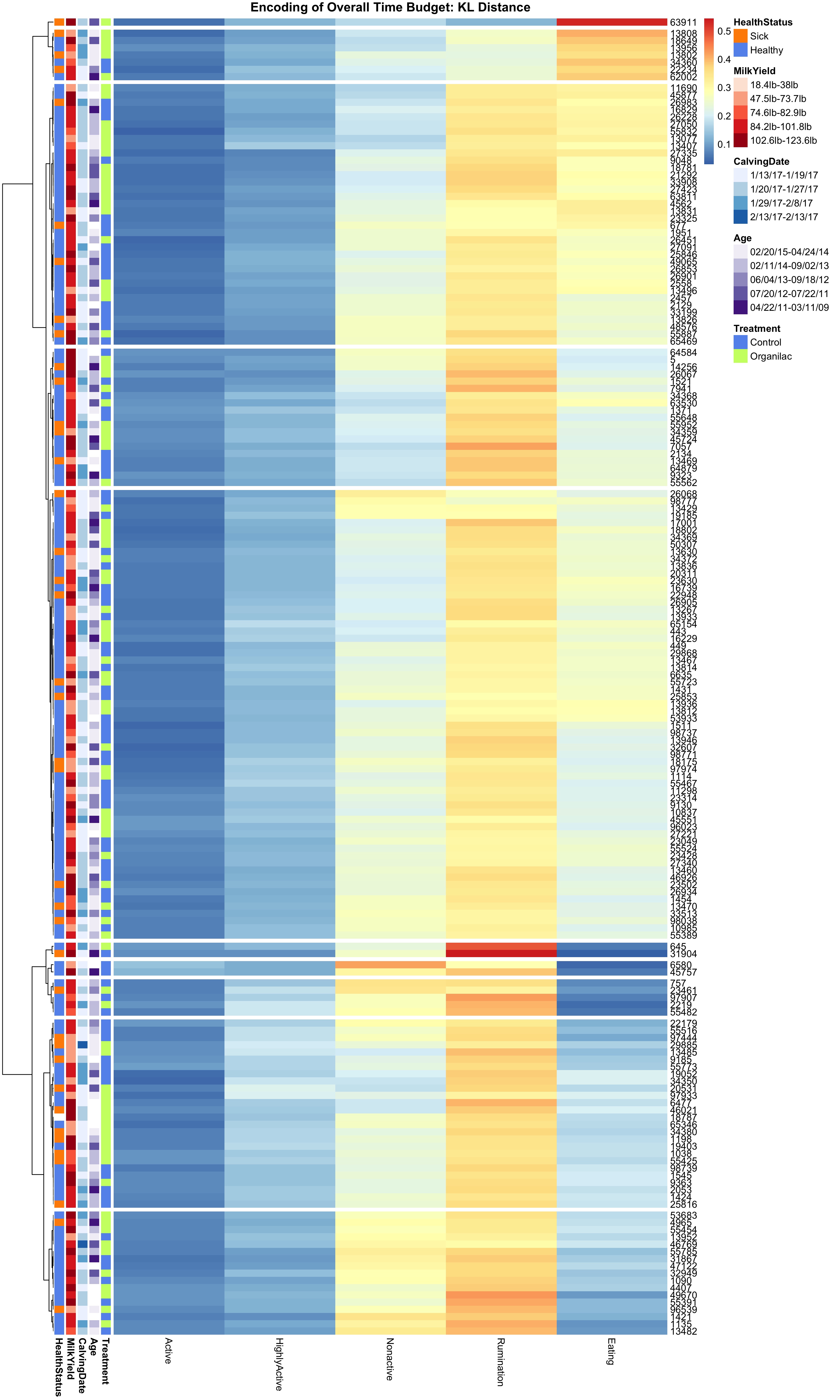

Supplement: Supplementary file 1 [file sensors-22-00001-s001.zip › sensors-1463895-supplementary/OverallTB/OTBEncodings/KLDivergence/_OverallTB_KLD_AuxVar_R10_C0.jpeg]

3

2

1

0

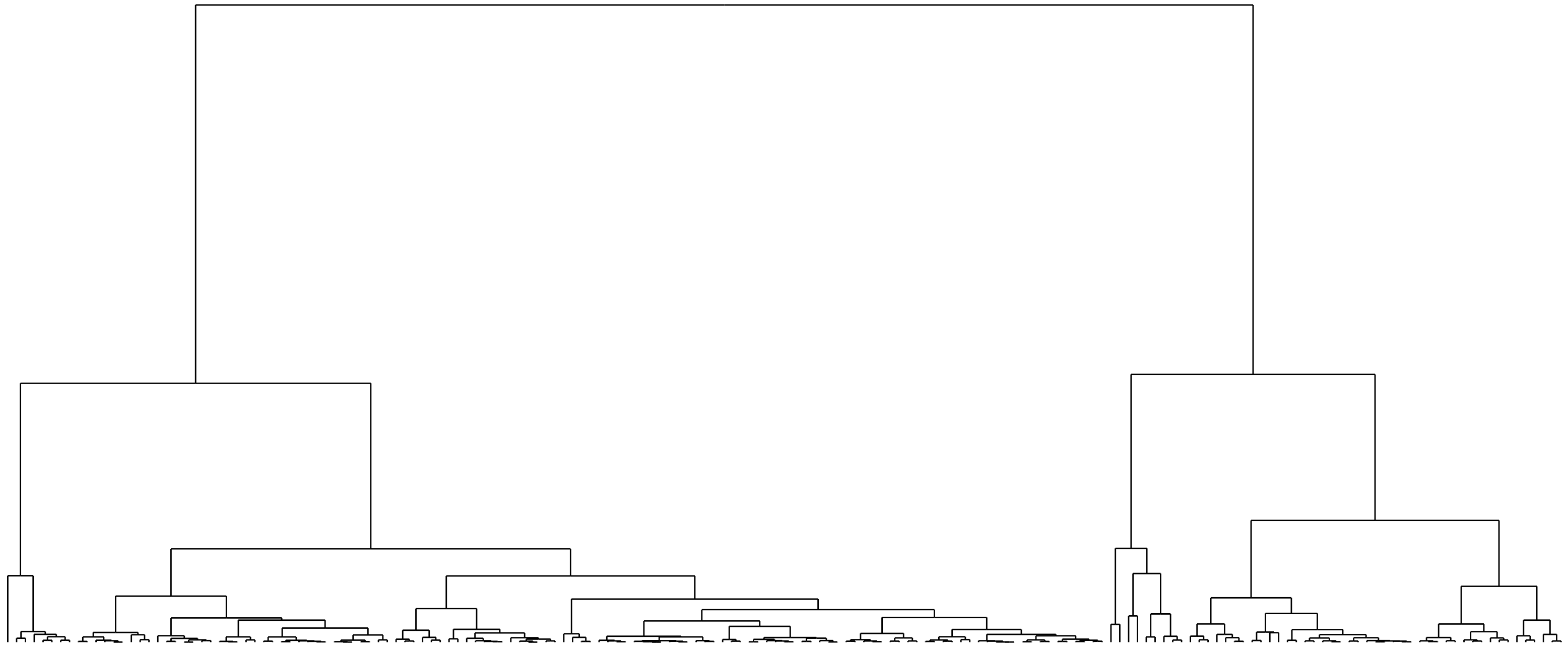

1 2 3 4 5 6 7 8 9 10 11 12 13 14 15 16 17 18 19 20 21 22 23 24 25 26 27 28 29 30 31 32 33 34 35 36 37 38 39 40 41 42 43 44 45 46 47 48 49 50 51 52 53 54 55 56 57 58 59 60 61 62 63 64 65 66 67 68 69 70 71 72 73 74 75 76 77 78 79 80 81 82 83 84 85 86 87 88 89 90 91 92 93 94 95 96 97 98 99 100

Supplement: Supplementary file 1 [file sensors-22-00001-s001.zip › sensors-1463895-supplementary/OverallTB/OTBEncodings/KLDivergence/_OverallTB_KLD_TBVar_Dendrogram_.pdf]

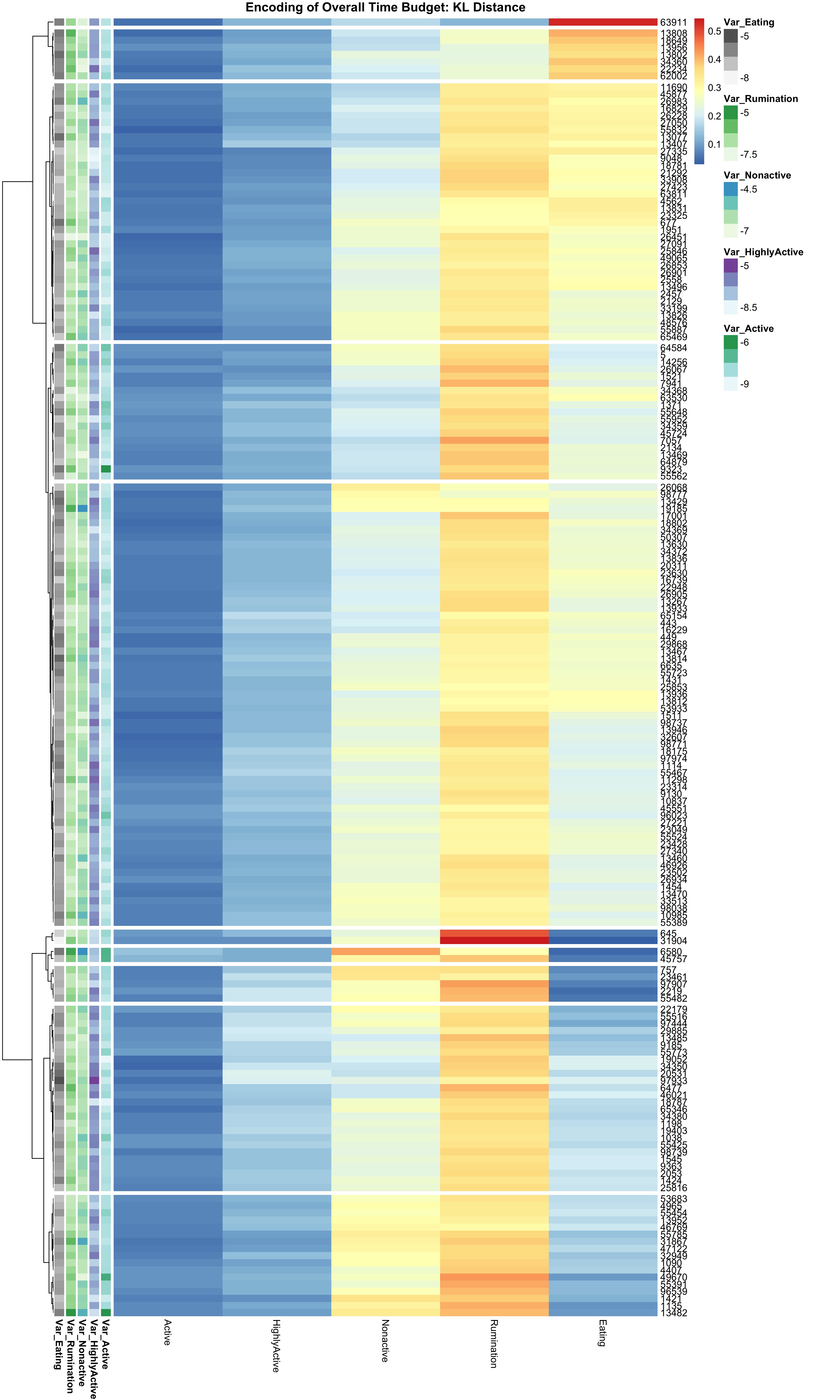

Supplement: Supplementary file 1 [file sensors-22-00001-s001.zip › sensors-1463895-supplementary/OverallTB/OTBEncodings/KLDivergence/_OverallTB_KLD_TBVar_R10_C0.jpeg]

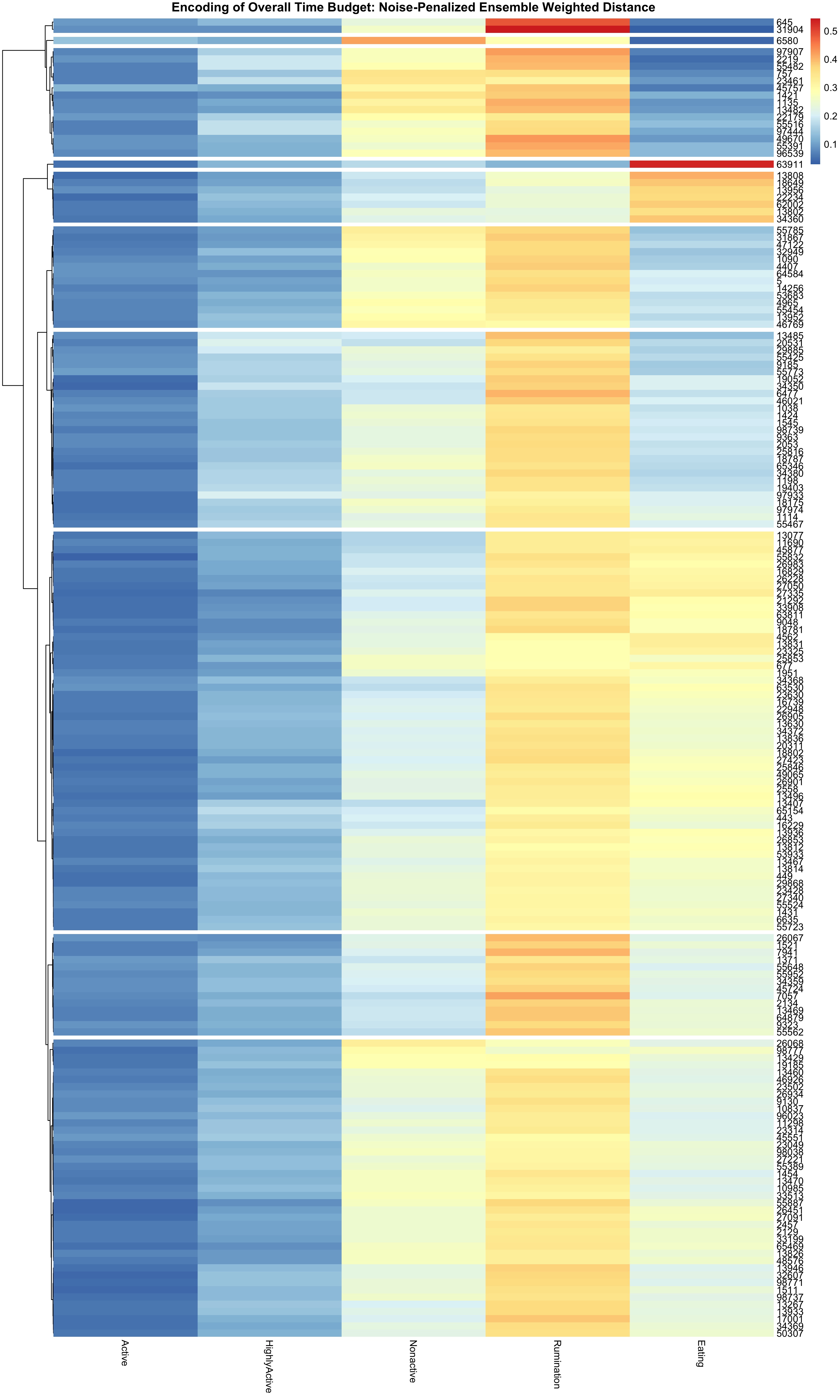

Supplement: Supplementary file 1 [file sensors-22-00001-s001.zip › sensors-1463895-supplementary/OverallTB/OTBEncodings/NoisePenalized/OverallTB_EW_R10_C0.jpeg]

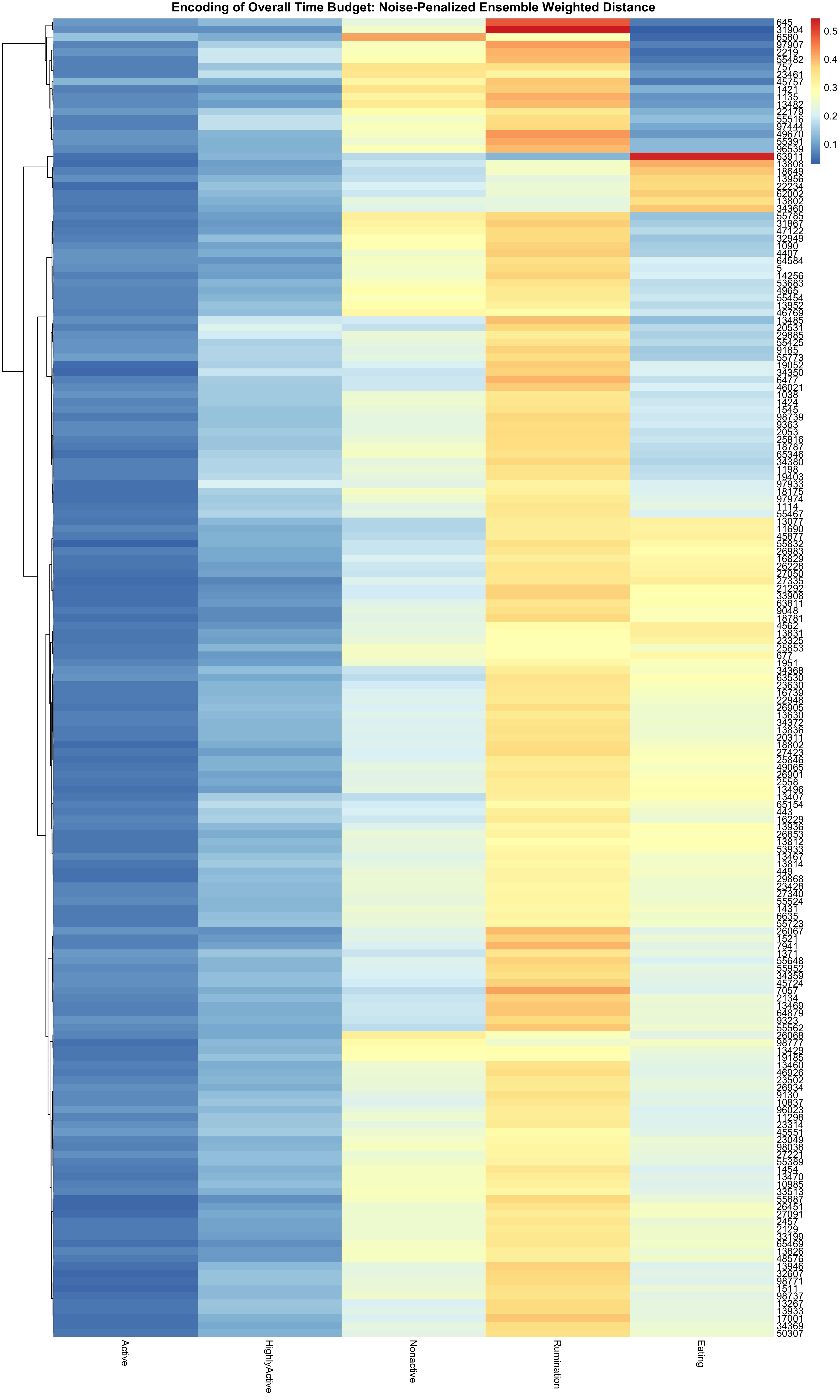

Supplement: Supplementary file 1 [file sensors-22-00001-s001.zip › sensors-1463895-supplementary/OverallTB/OTBEncodings/NoisePenalized/OverallTB_EW_R1_C0.jpeg]

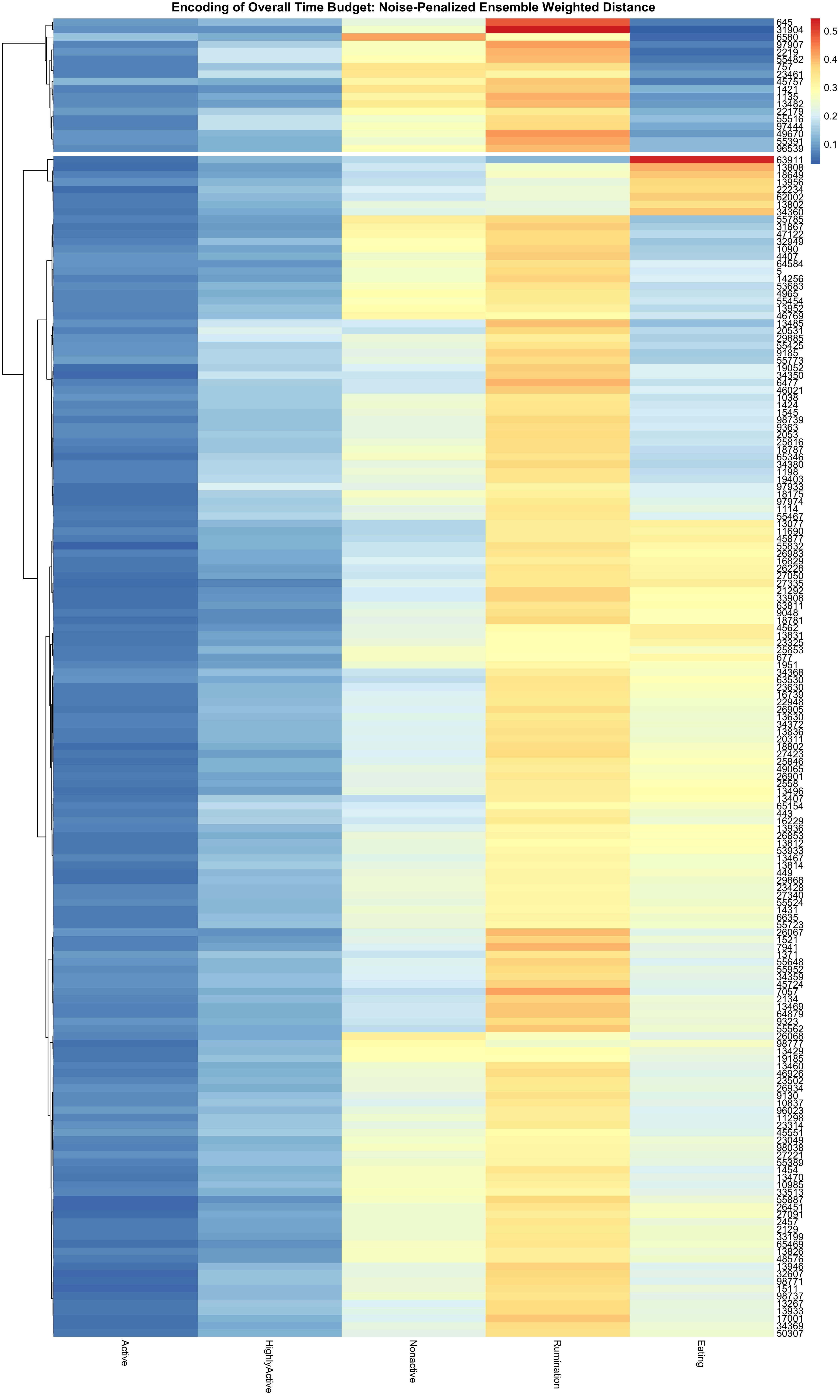

Supplement: Supplementary file 1 [file sensors-22-00001-s001.zip › sensors-1463895-supplementary/OverallTB/OTBEncodings/NoisePenalized/OverallTB_EW_R2_C0.jpeg]

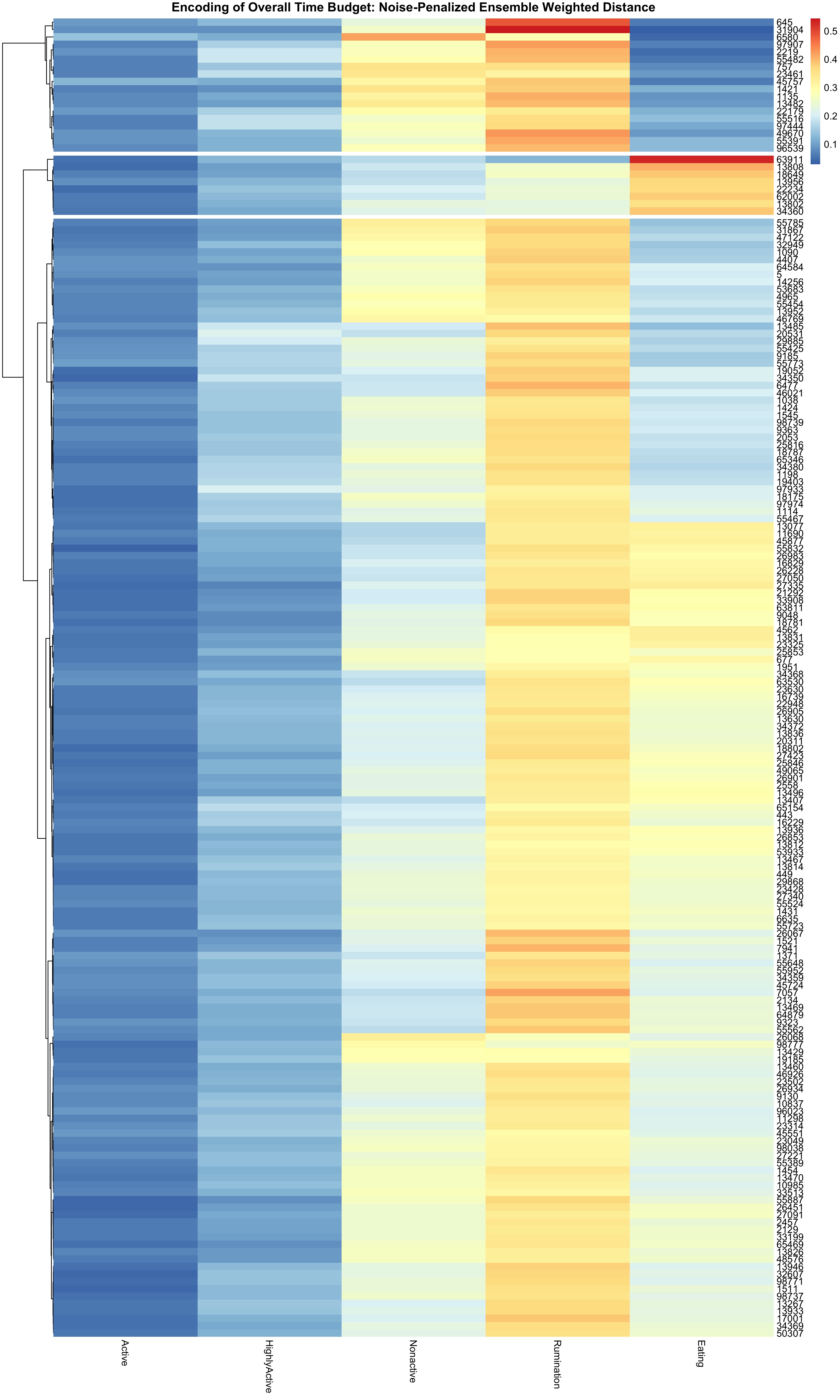

Supplement: Supplementary file 1 [file sensors-22-00001-s001.zip › sensors-1463895-supplementary/OverallTB/OTBEncodings/NoisePenalized/OverallTB_EW_R3_C0.jpeg]

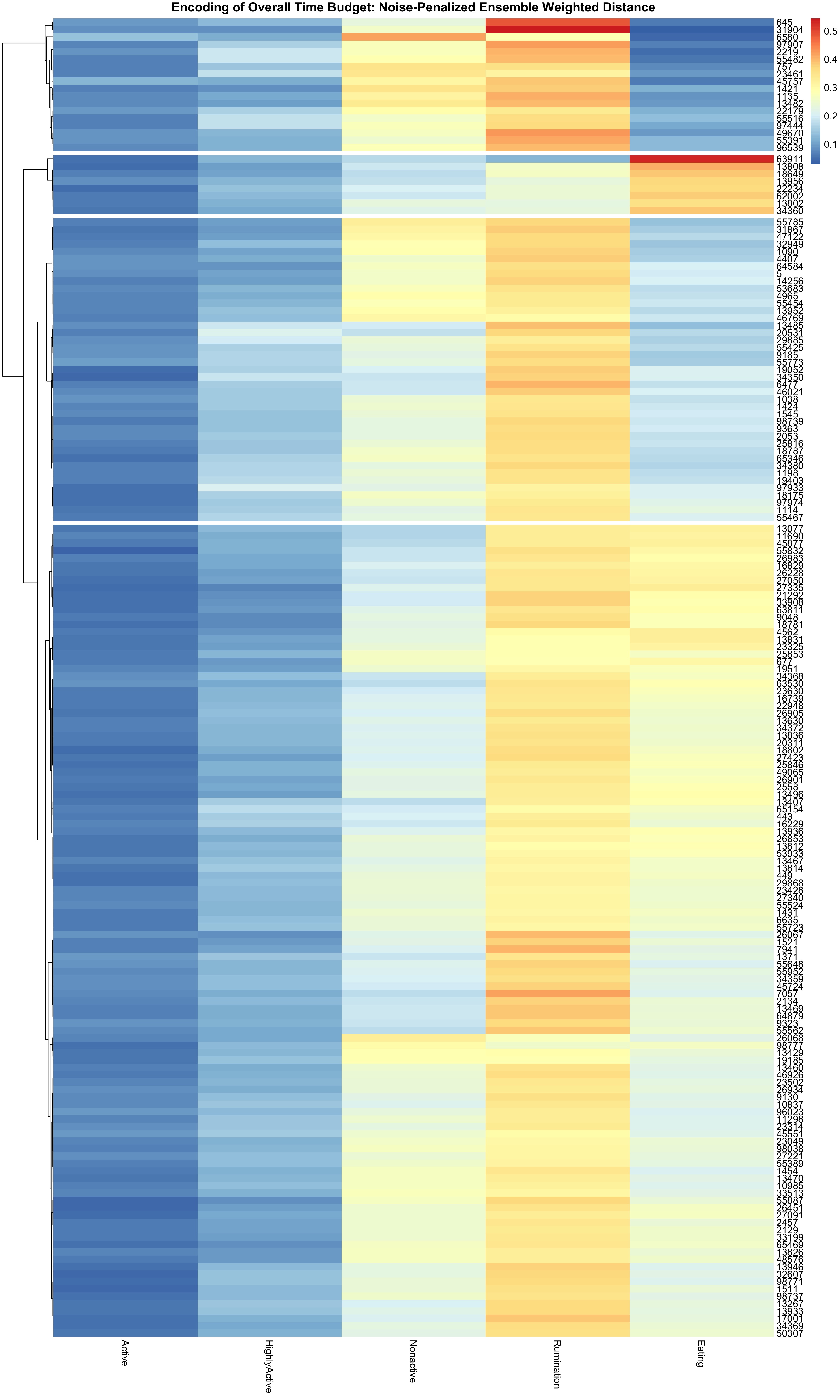

Supplement: Supplementary file 1 [file sensors-22-00001-s001.zip › sensors-1463895-supplementary/OverallTB/OTBEncodings/NoisePenalized/OverallTB_EW_R4_C0.jpeg]

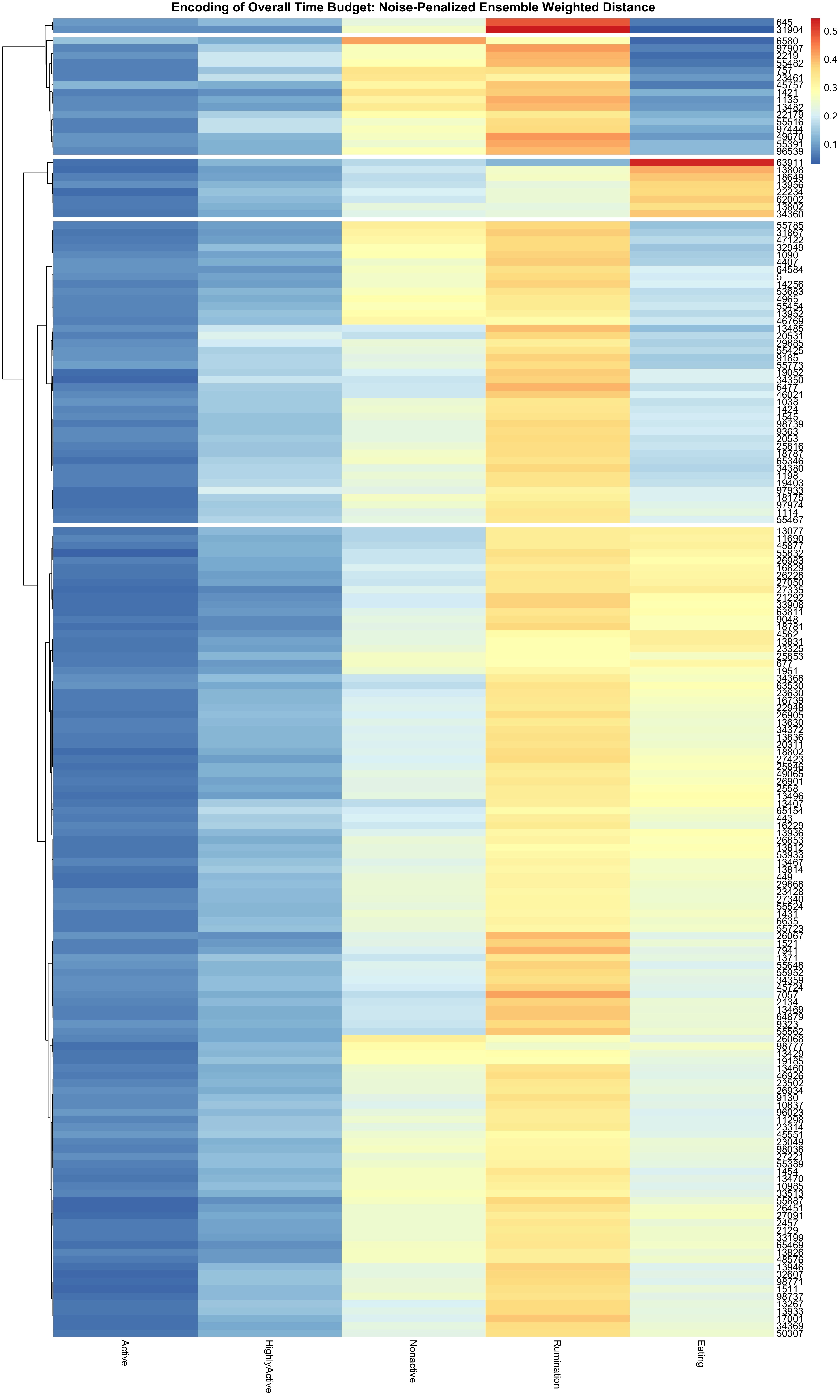

Supplement: Supplementary file 1 [file sensors-22-00001-s001.zip › sensors-1463895-supplementary/OverallTB/OTBEncodings/NoisePenalized/OverallTB_EW_R5_C0.jpeg]

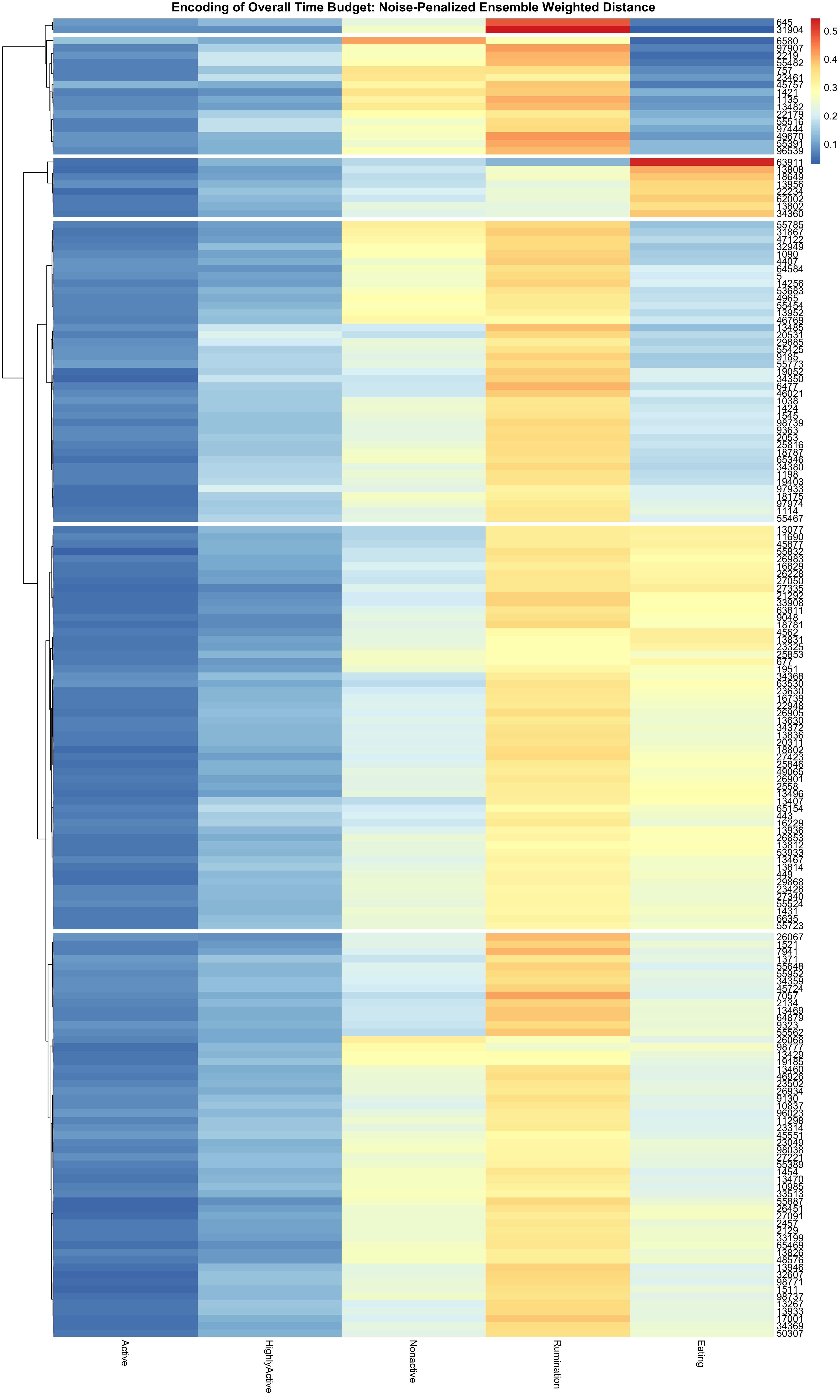

Supplement: Supplementary file 1 [file sensors-22-00001-s001.zip › sensors-1463895-supplementary/OverallTB/OTBEncodings/NoisePenalized/OverallTB_EW_R6_C0.jpeg]

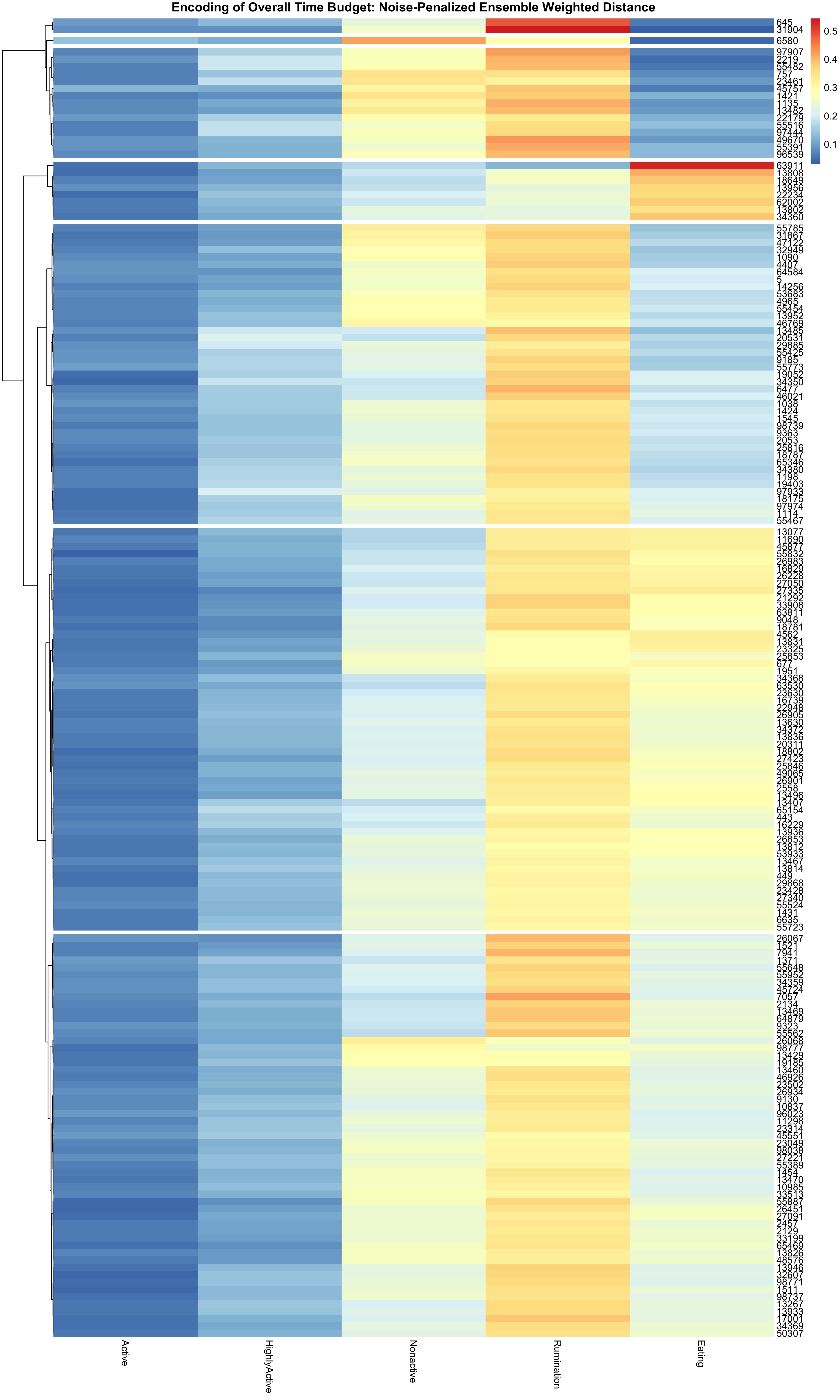

Supplement: Supplementary file 1 [file sensors-22-00001-s001.zip › sensors-1463895-supplementary/OverallTB/OTBEncodings/NoisePenalized/OverallTB_EW_R7_C0.jpeg]

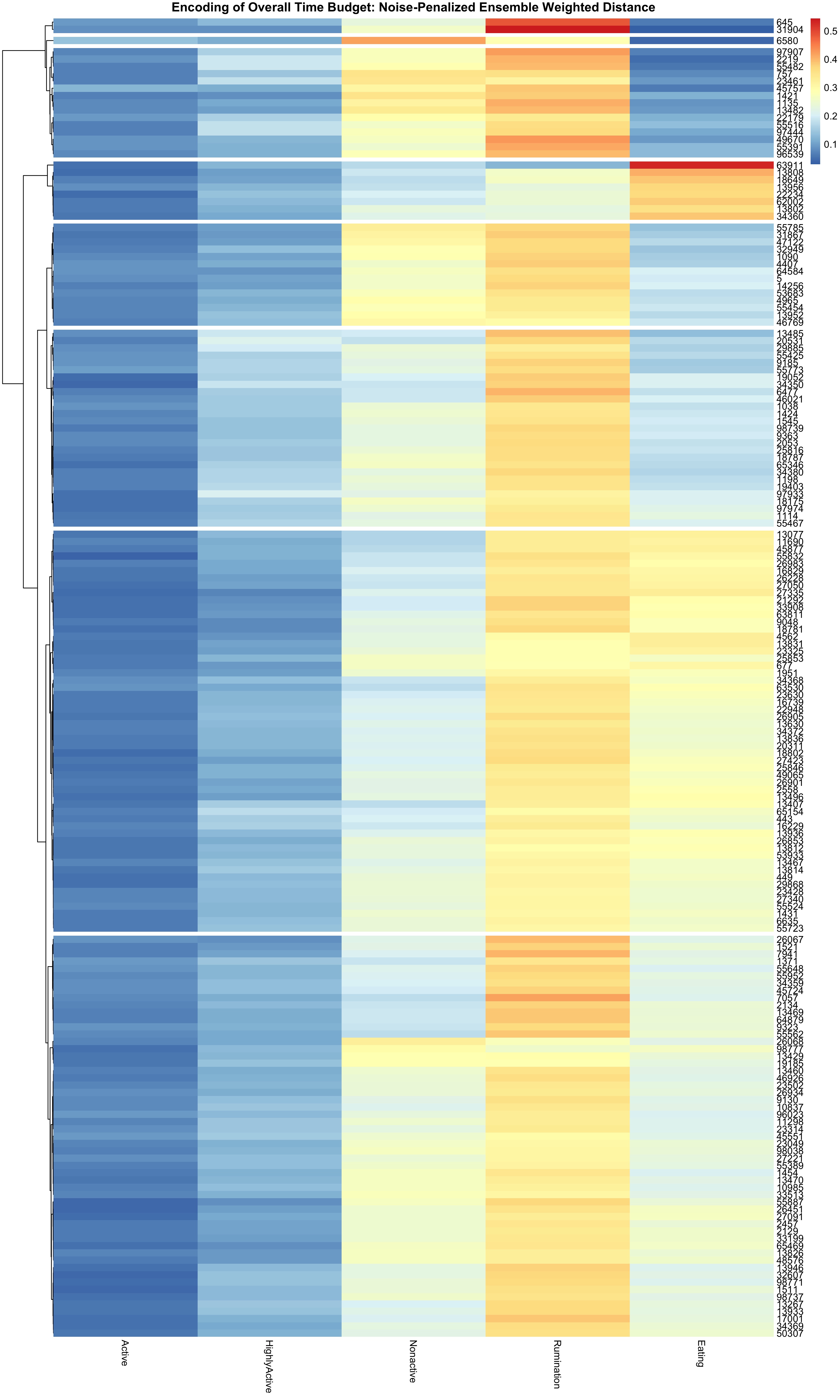

Supplement: Supplementary file 1 [file sensors-22-00001-s001.zip › sensors-1463895-supplementary/OverallTB/OTBEncodings/NoisePenalized/OverallTB_EW_R8_C0.jpeg]

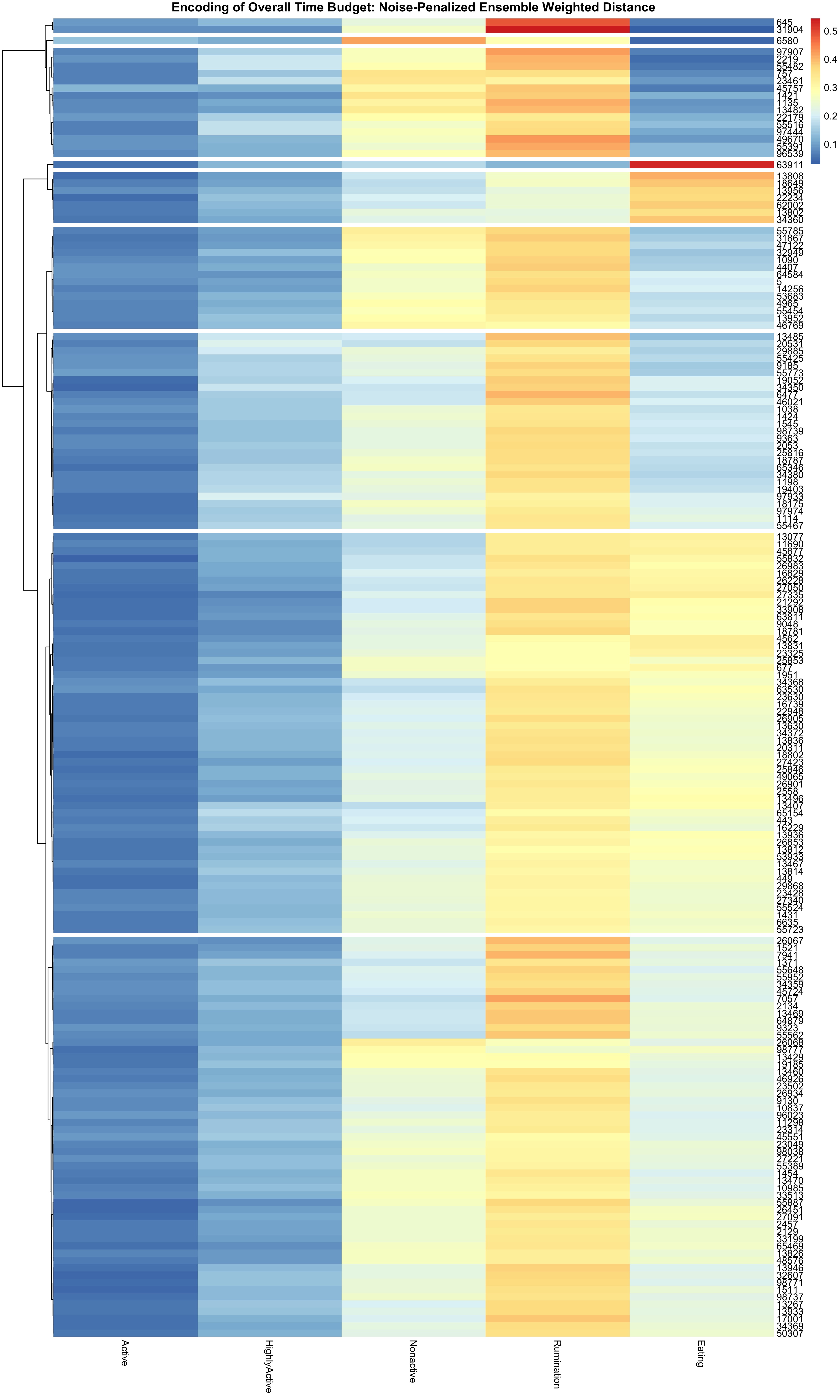

Supplement: Supplementary file 1 [file sensors-22-00001-s001.zip › sensors-1463895-supplementary/OverallTB/OTBEncodings/NoisePenalized/OverallTB_EW_R9_C0.jpeg]

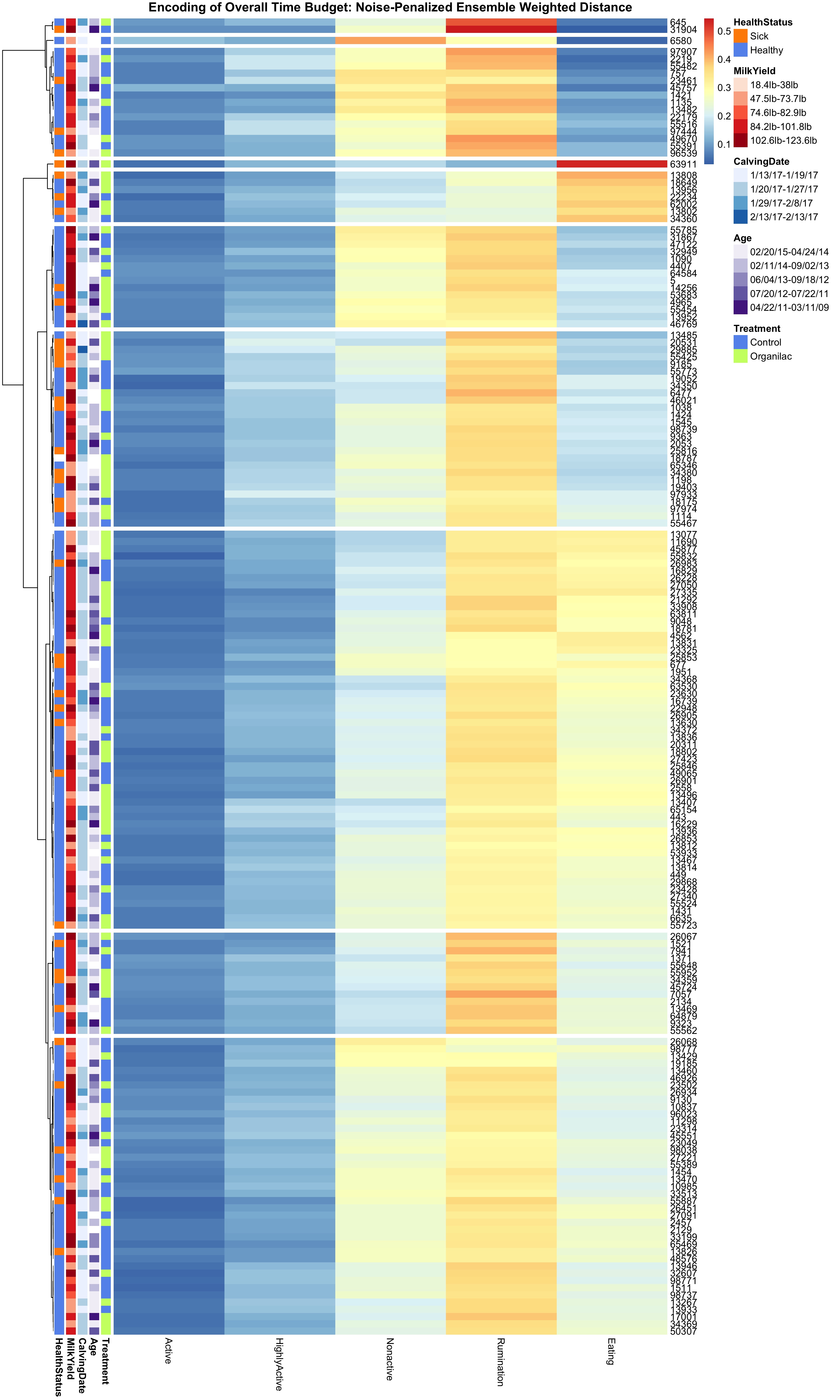

Supplement: Supplementary file 1 [file sensors-22-00001-s001.zip › sensors-1463895-supplementary/OverallTB/OTBEncodings/NoisePenalized/_OverallTB_EW_AuxVar_R10_C0.jpeg]

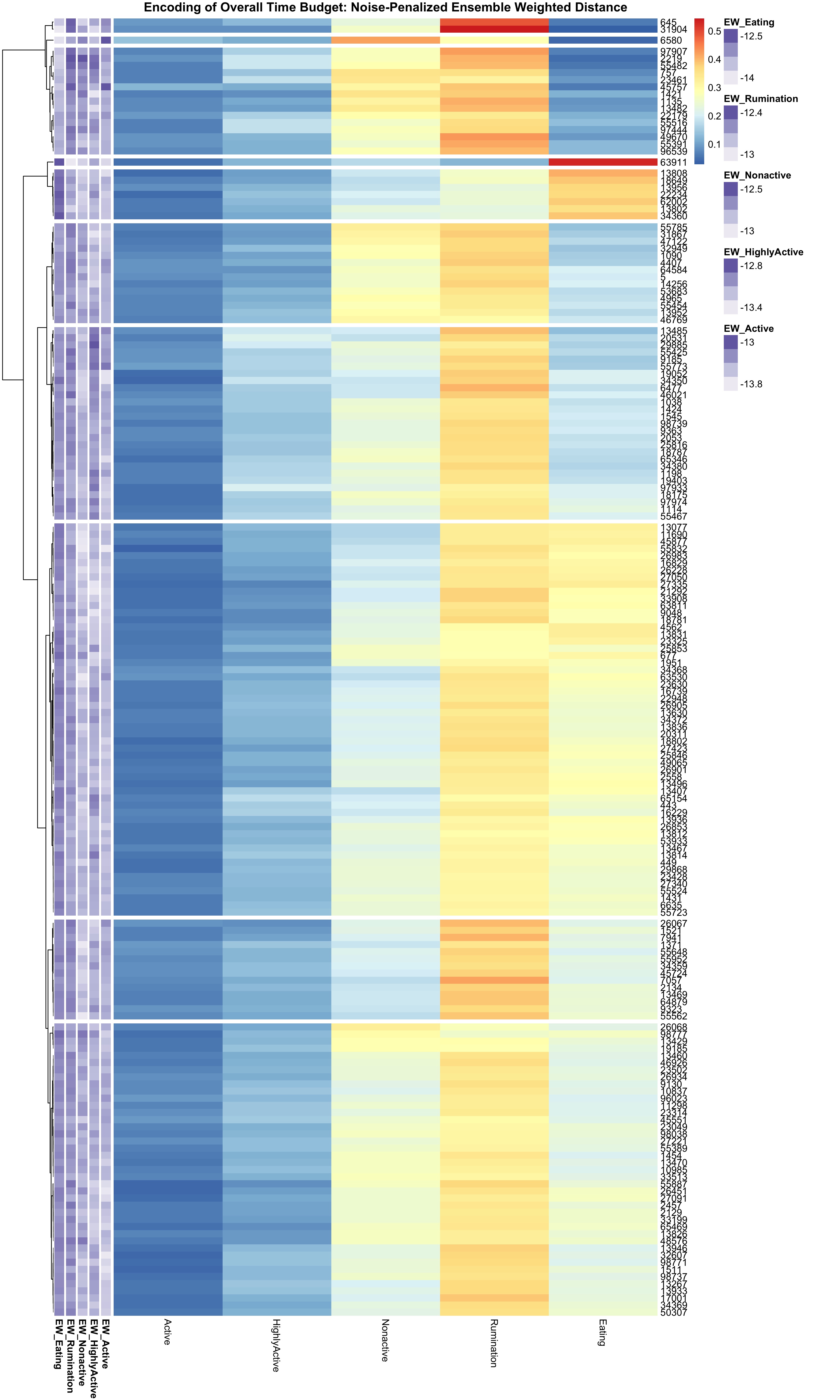

Supplement: Supplementary file 1 [file sensors-22-00001-s001.zip › sensors-1463895-supplementary/OverallTB/OTBEncodings/NoisePenalized/_OverallTB_EW_EWVAR_R10_C0.jpeg]

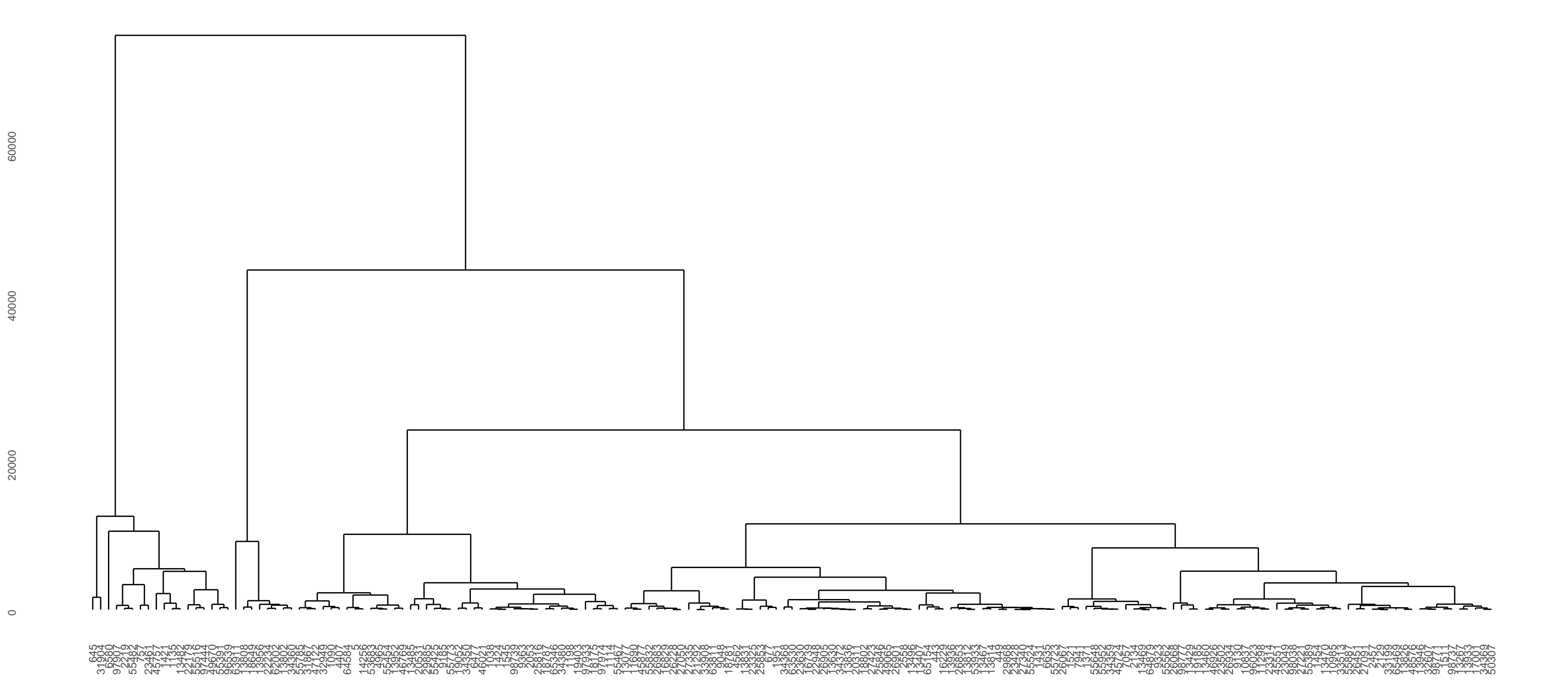

Supplement: Supplementary file 1 [file sensors-22-00001-s001.zip › sensors-1463895-supplementary/OverallTB/OTBEncodings/NoisePenalized/_OverallTB_EW_TBVAR_Dendrogram_.pdf]

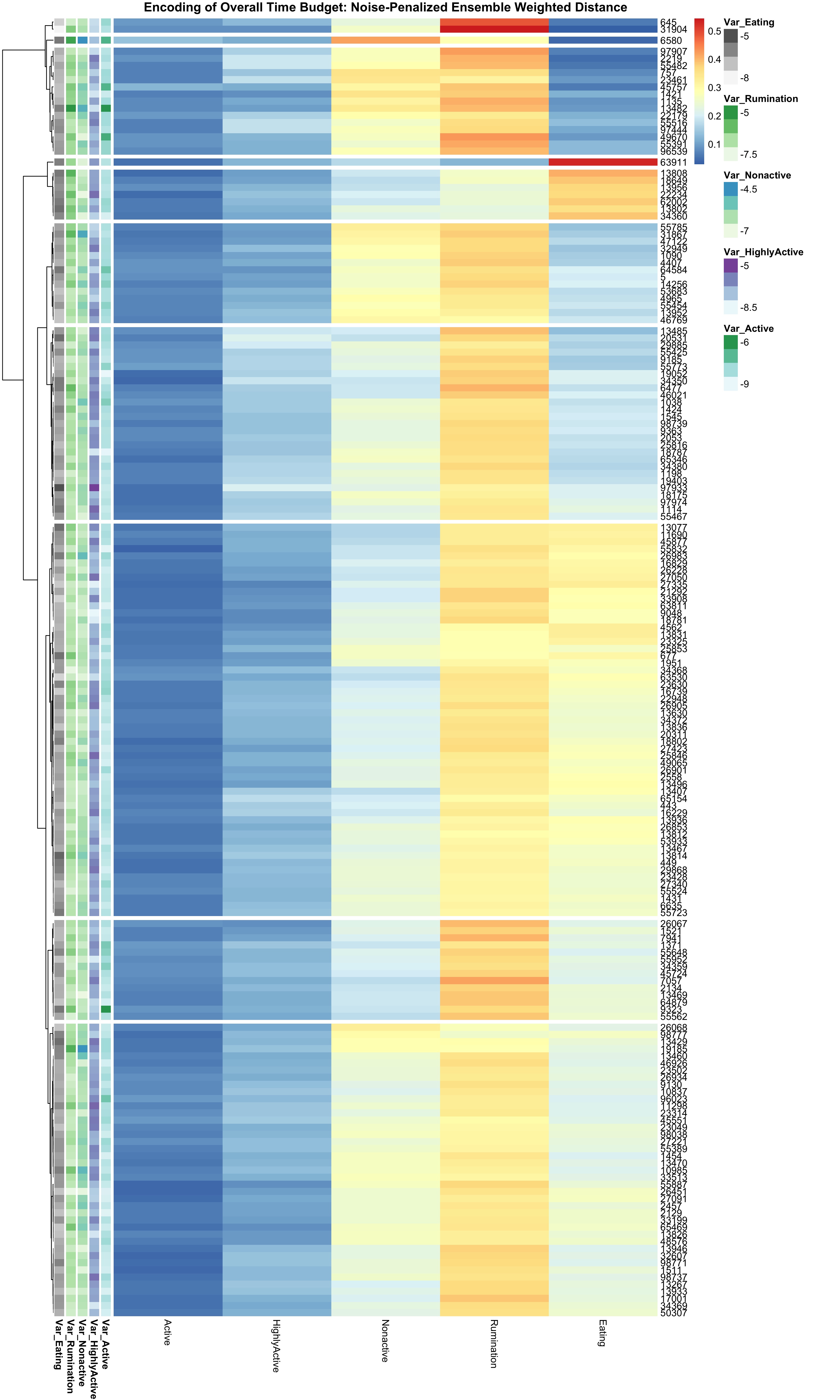

Supplement: Supplementary file 1 [file sensors-22-00001-s001.zip › sensors-1463895-supplementary/OverallTB/OTBEncodings/NoisePenalized/_OverallTB_EW_TBVAR_R10_C0.jpeg]

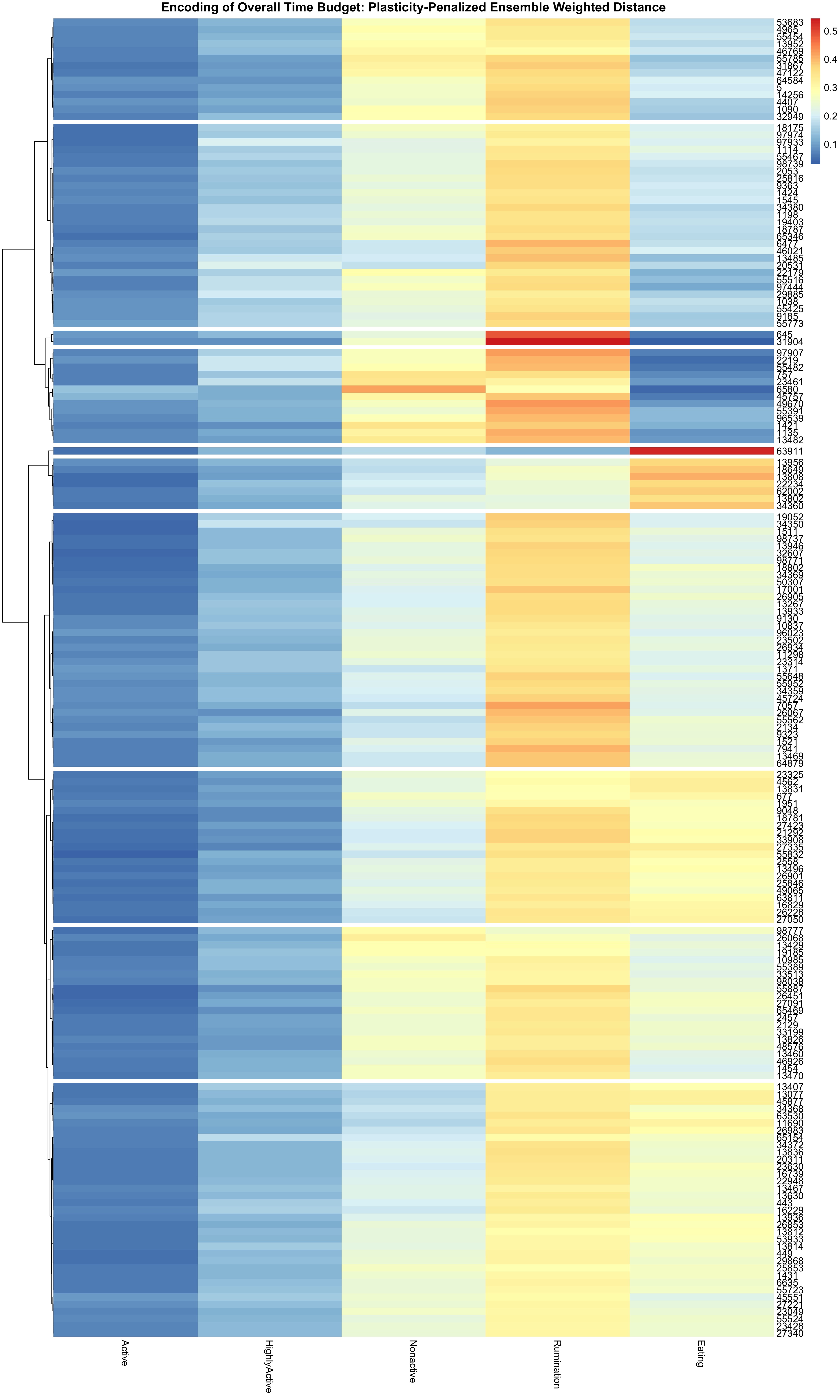

Supplement: Supplementary file 1 [file sensors-22-00001-s001.zip › sensors-1463895-supplementary/OverallTB/OTBEncodings/PlasticityPenalized/OverallTB_PW_R10_C0.jpeg]

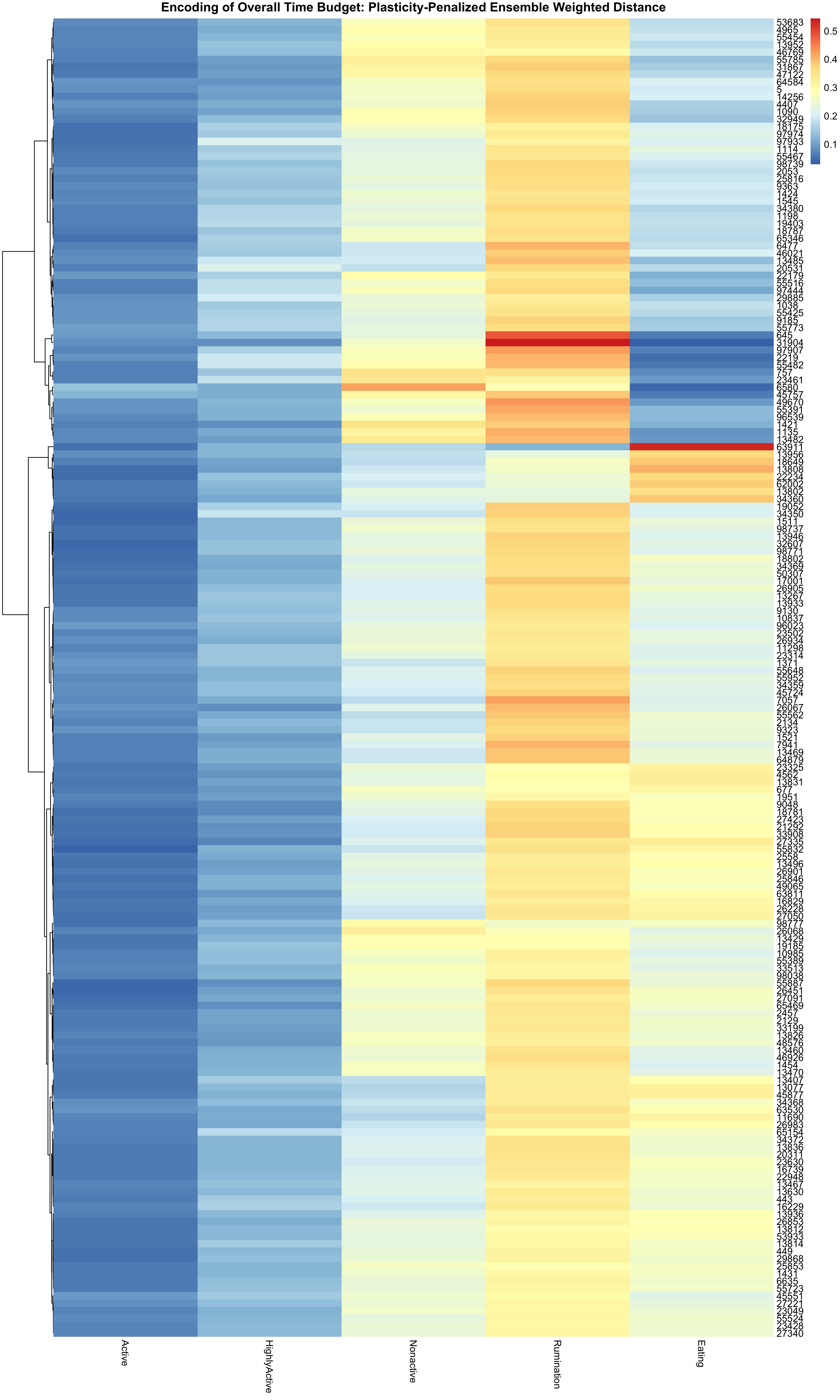

Supplement: Supplementary file 1 [file sensors-22-00001-s001.zip › sensors-1463895-supplementary/OverallTB/OTBEncodings/PlasticityPenalized/OverallTB_PW_R1_C0.jpeg]

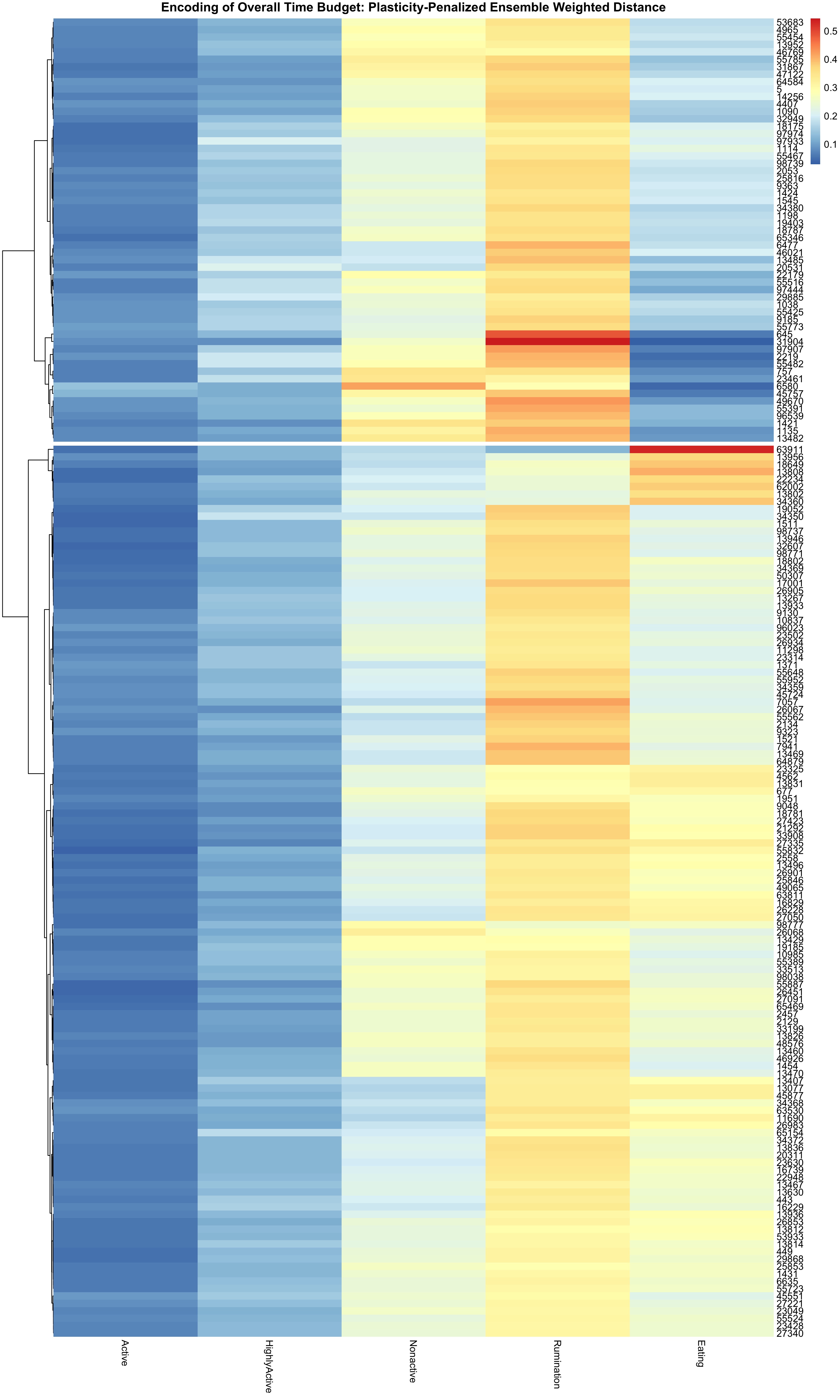

Supplement: Supplementary file 1 [file sensors-22-00001-s001.zip › sensors-1463895-supplementary/OverallTB/OTBEncodings/PlasticityPenalized/OverallTB_PW_R2_C0.jpeg]

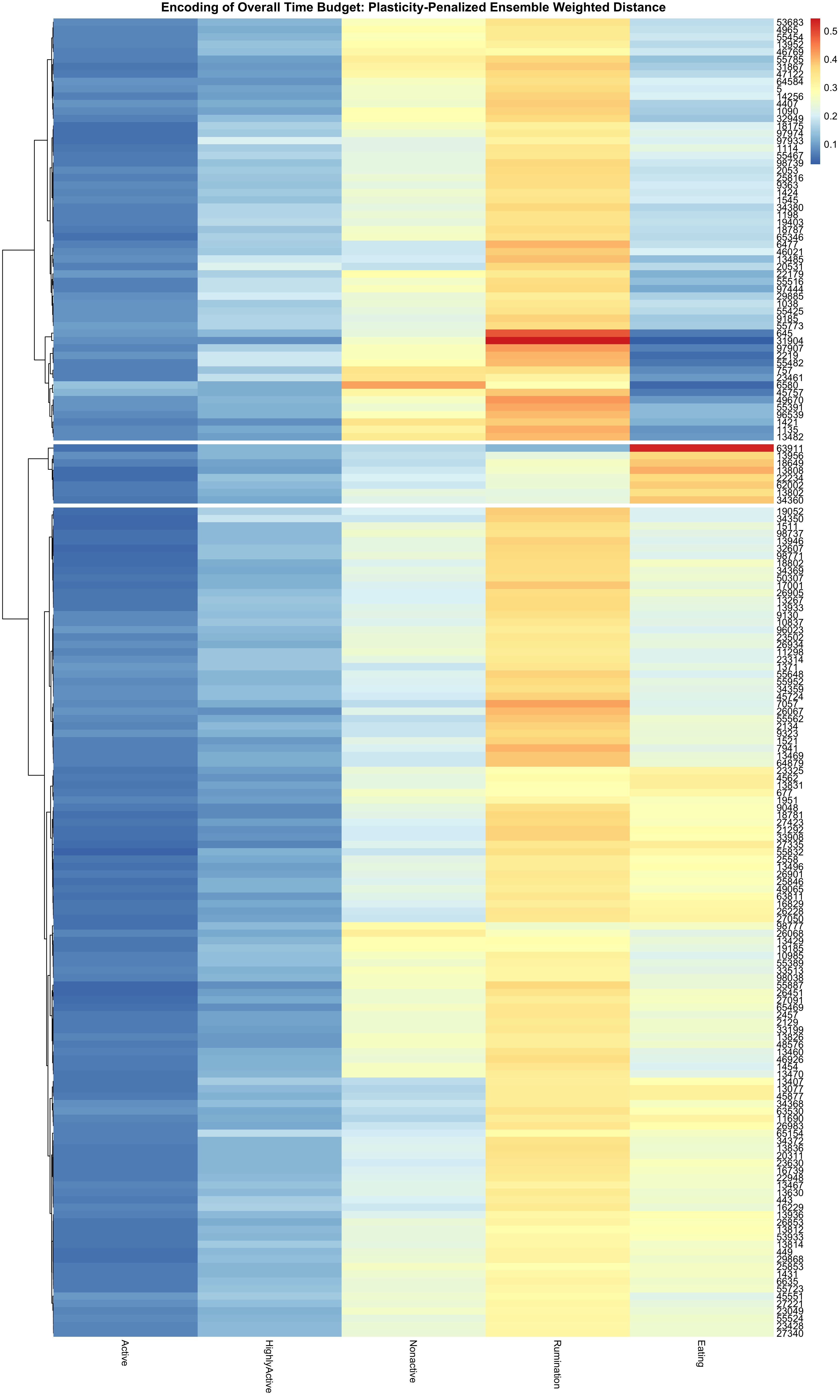

Supplement: Supplementary file 1 [file sensors-22-00001-s001.zip › sensors-1463895-supplementary/OverallTB/OTBEncodings/PlasticityPenalized/OverallTB_PW_R3_C0.jpeg]

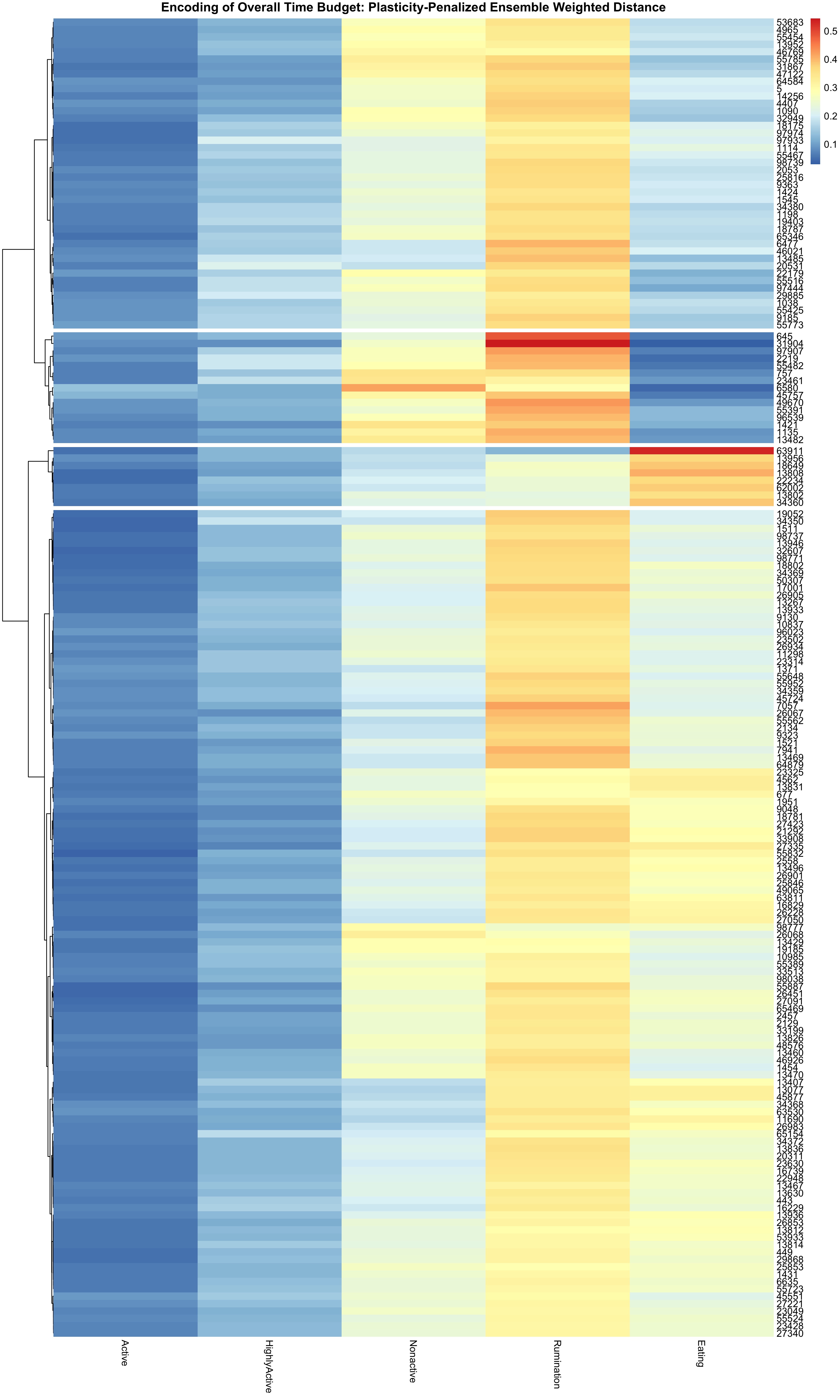

Supplement: Supplementary file 1 [file sensors-22-00001-s001.zip › sensors-1463895-supplementary/OverallTB/OTBEncodings/PlasticityPenalized/OverallTB_PW_R4_C0.jpeg]

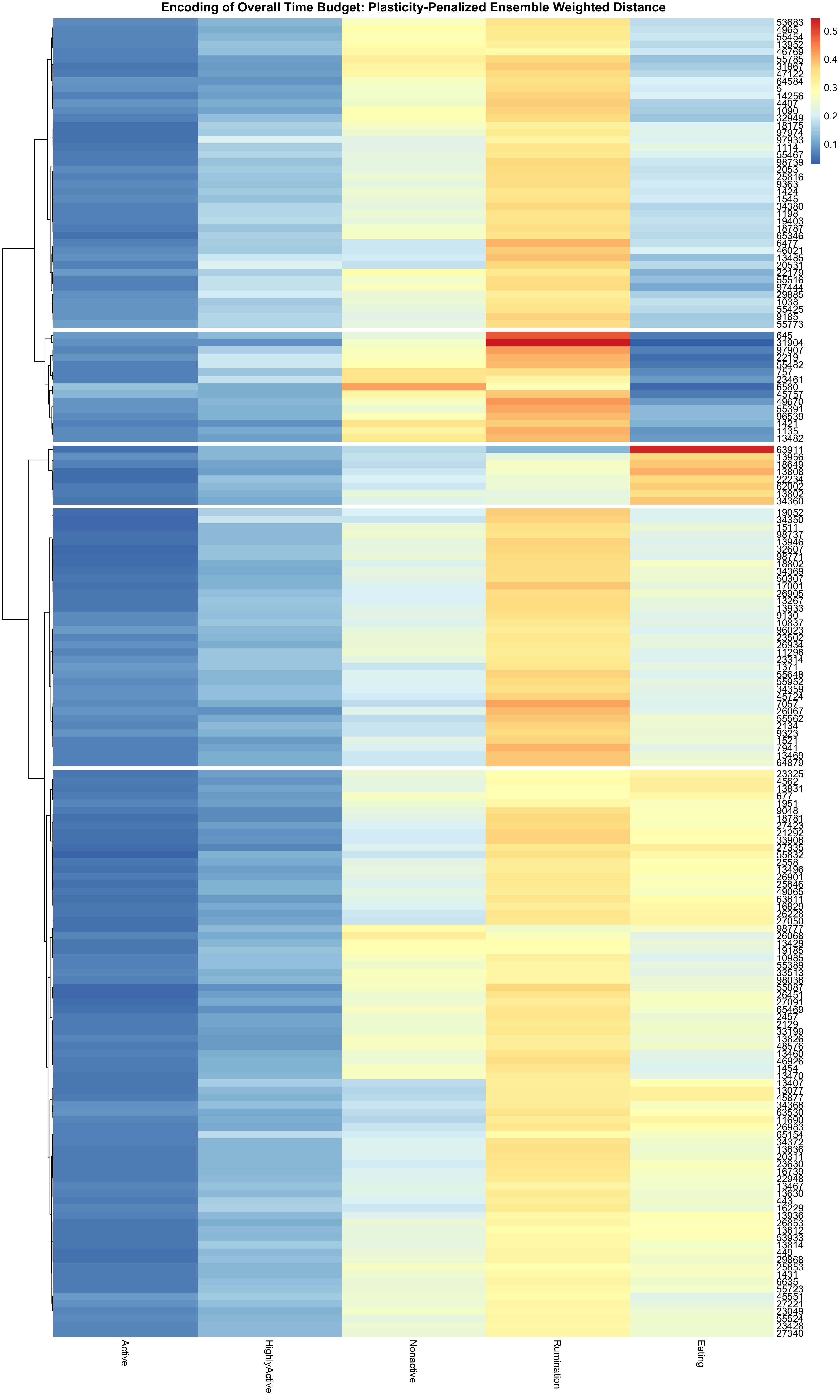

Supplement: Supplementary file 1 [file sensors-22-00001-s001.zip › sensors-1463895-supplementary/OverallTB/OTBEncodings/PlasticityPenalized/OverallTB_PW_R5_C0.jpeg]

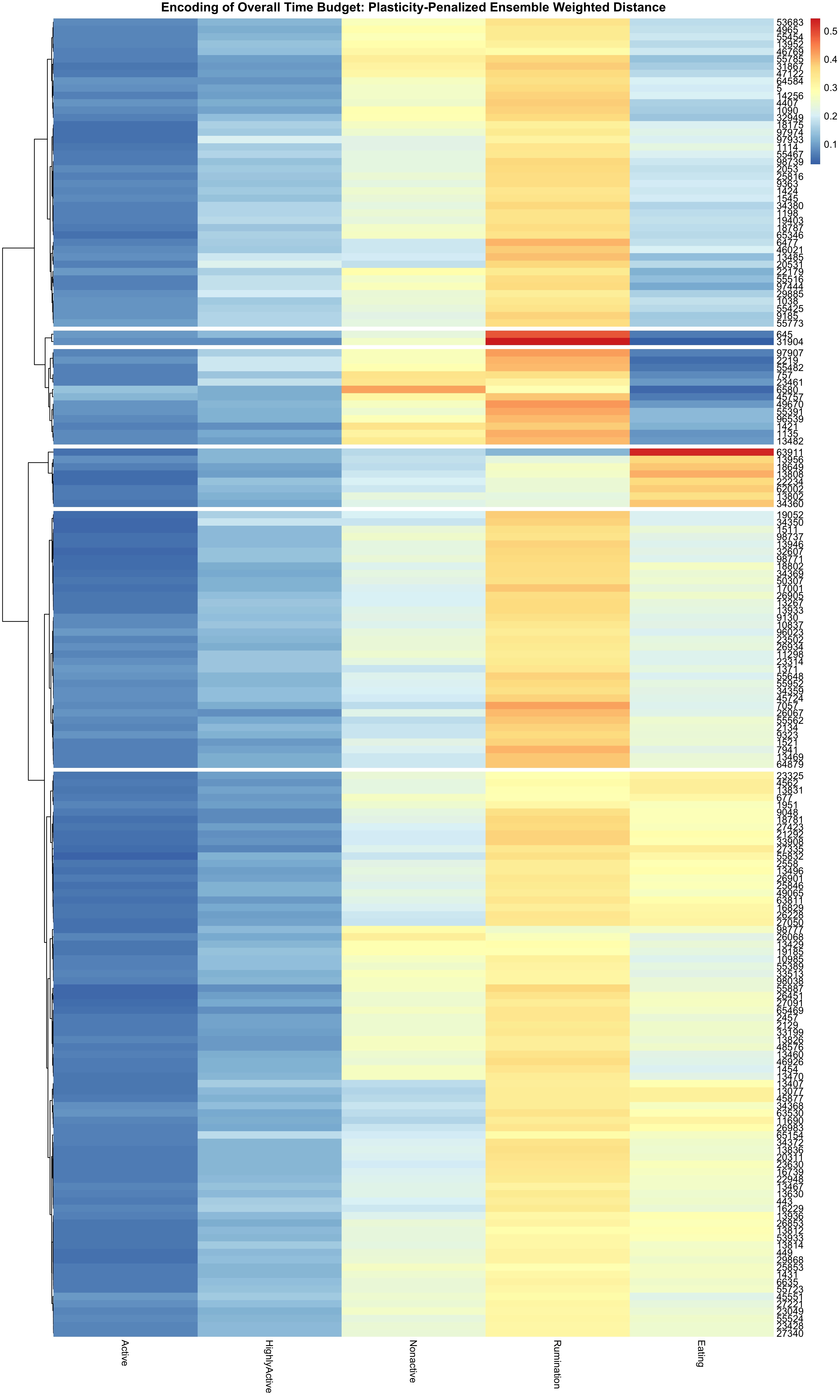

Supplement: Supplementary file 1 [file sensors-22-00001-s001.zip › sensors-1463895-supplementary/OverallTB/OTBEncodings/PlasticityPenalized/OverallTB_PW_R6_C0.jpeg]

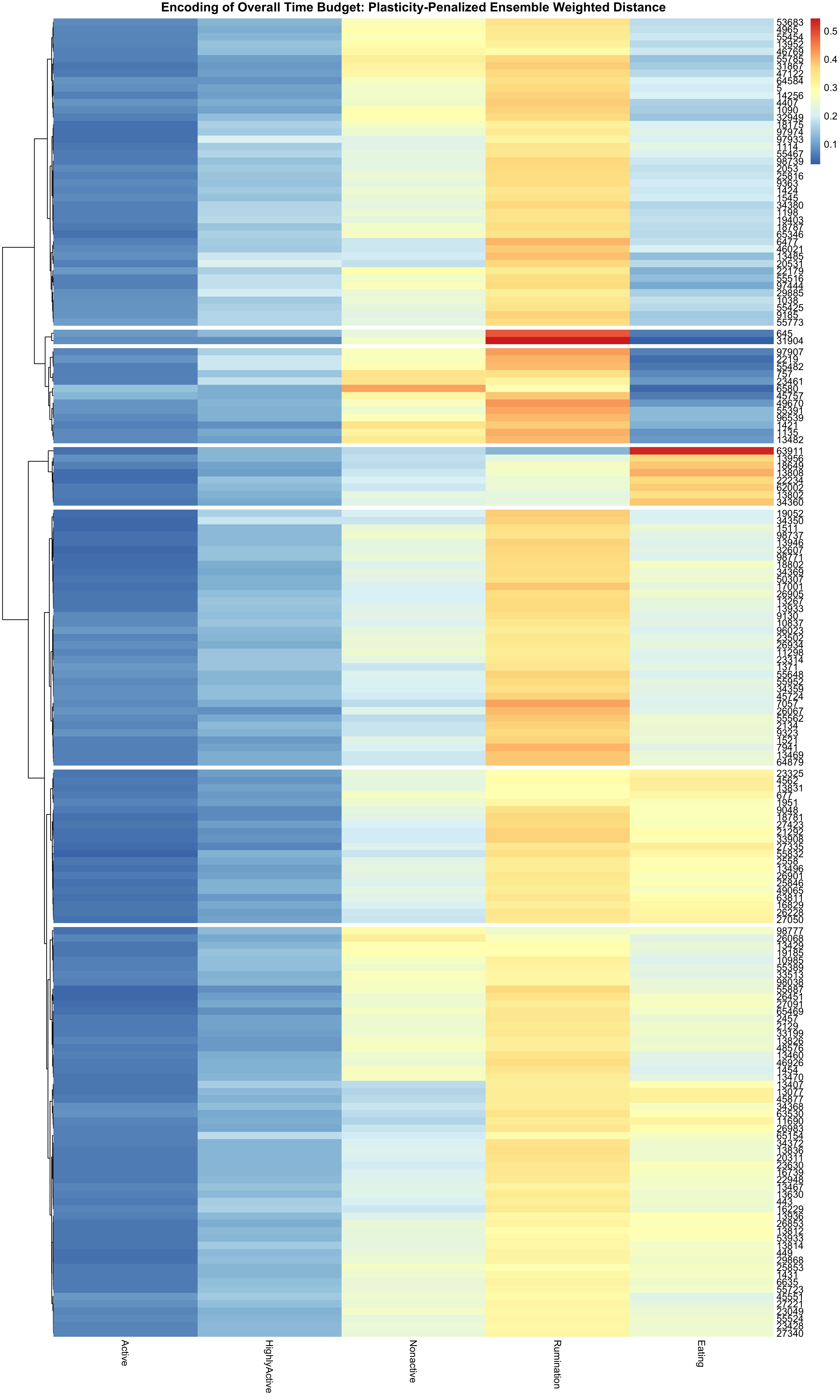

Supplement: Supplementary file 1 [file sensors-22-00001-s001.zip › sensors-1463895-supplementary/OverallTB/OTBEncodings/PlasticityPenalized/OverallTB_PW_R7_C0.jpeg]

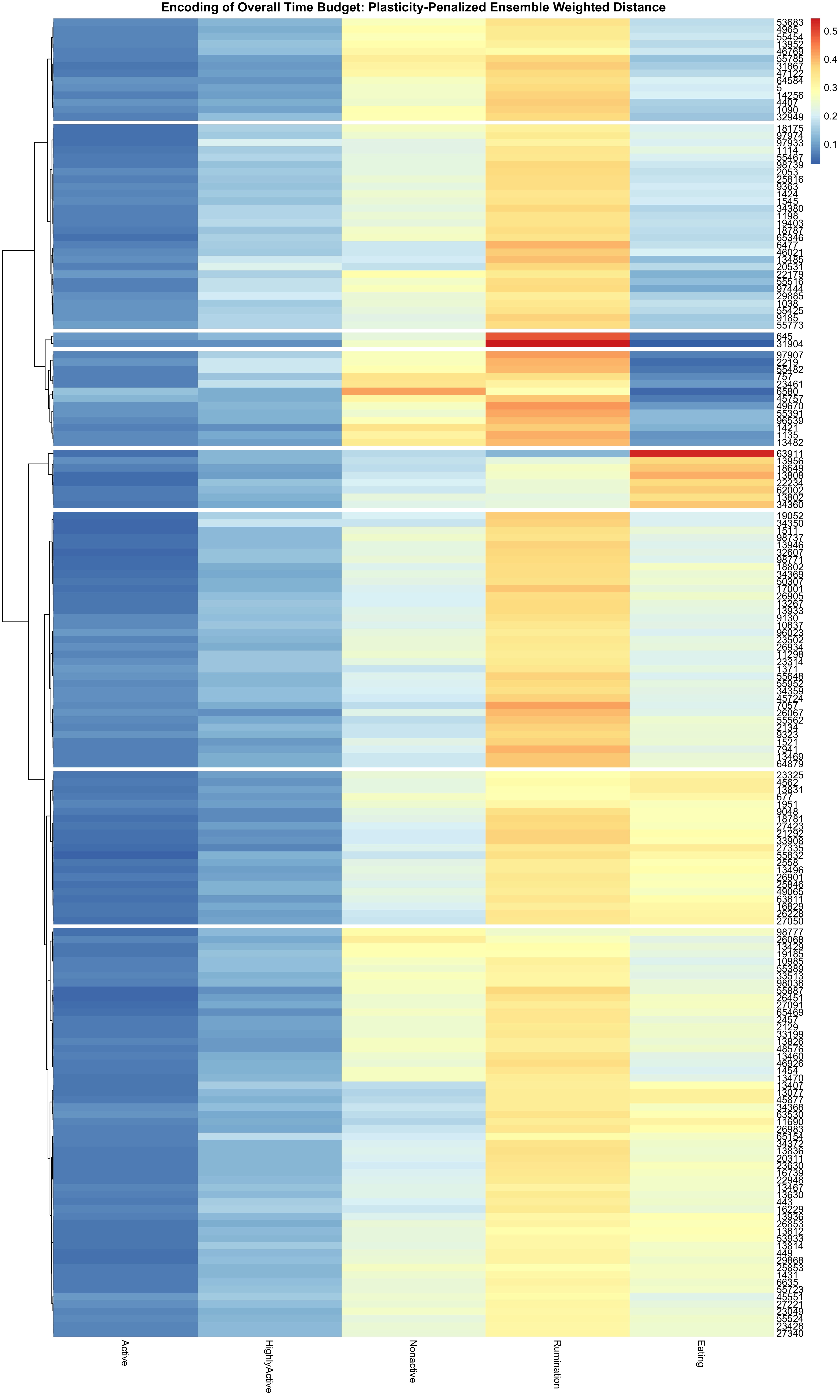

Supplement: Supplementary file 1 [file sensors-22-00001-s001.zip › sensors-1463895-supplementary/OverallTB/OTBEncodings/PlasticityPenalized/OverallTB_PW_R8_C0.jpeg]

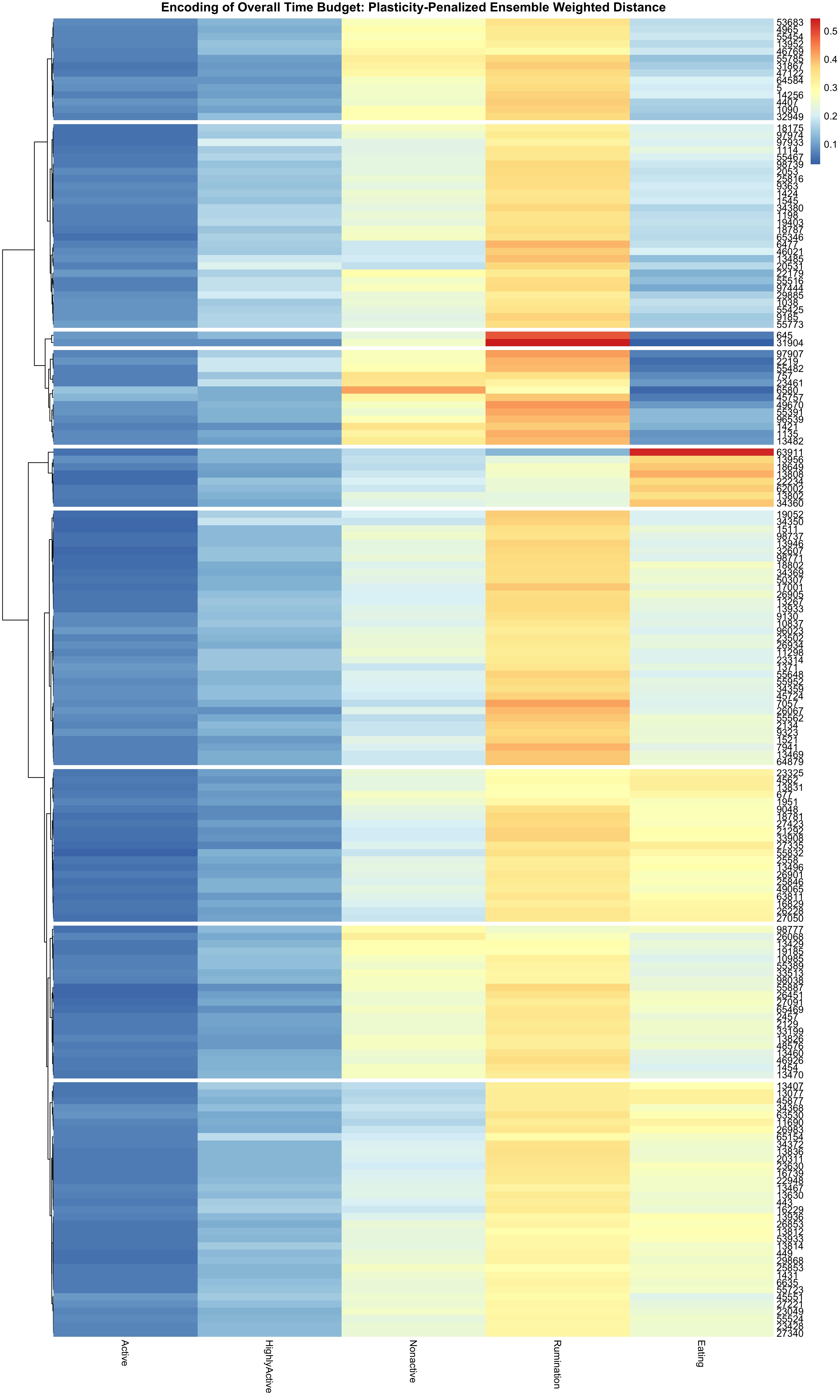

Supplement: Supplementary file 1 [file sensors-22-00001-s001.zip › sensors-1463895-supplementary/OverallTB/OTBEncodings/PlasticityPenalized/OverallTB_PW_R9_C0.jpeg]

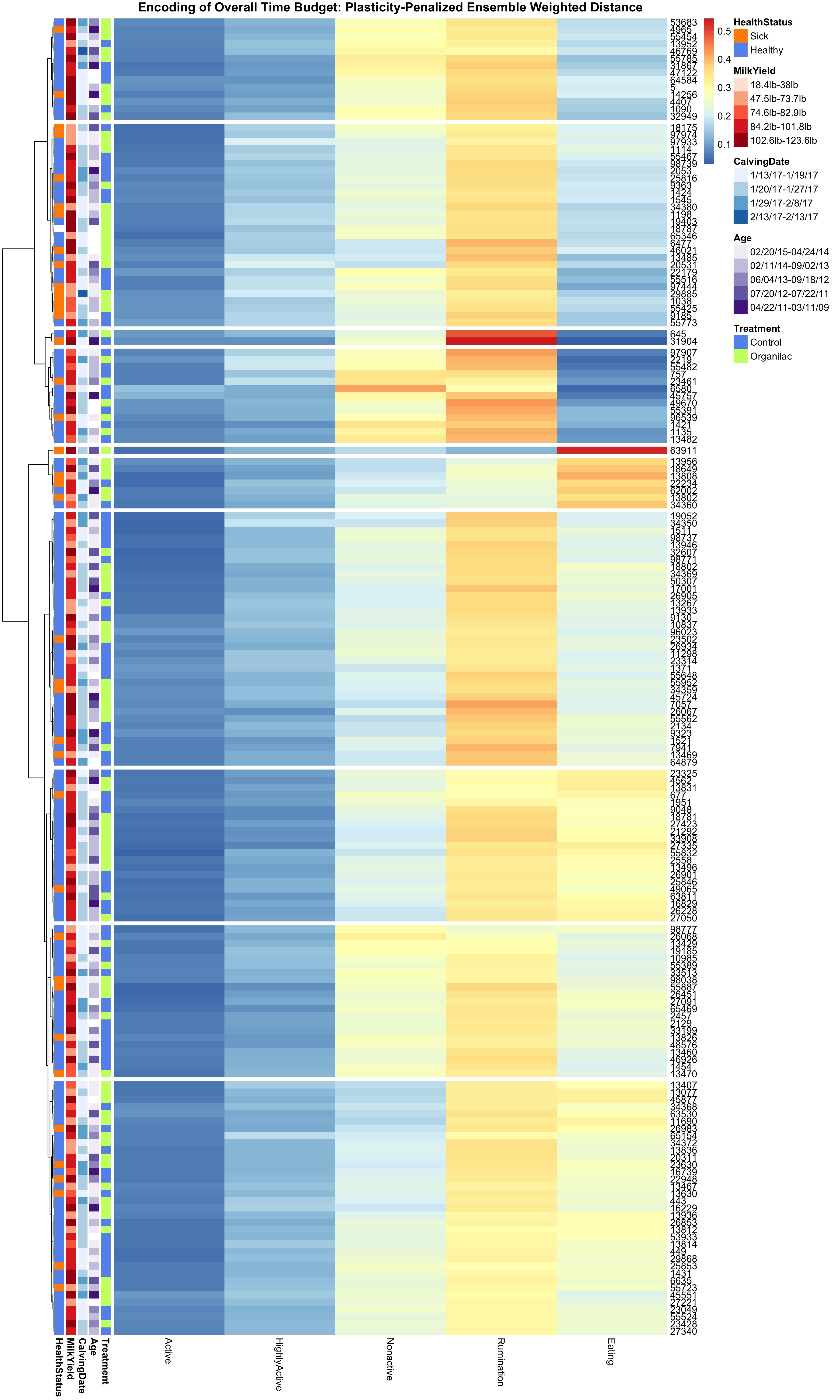

Supplement: Supplementary file 1 [file sensors-22-00001-s001.zip › sensors-1463895-supplementary/OverallTB/OTBEncodings/PlasticityPenalized/_OverallTB_PW_AuxVar_R10_C0.jpeg]

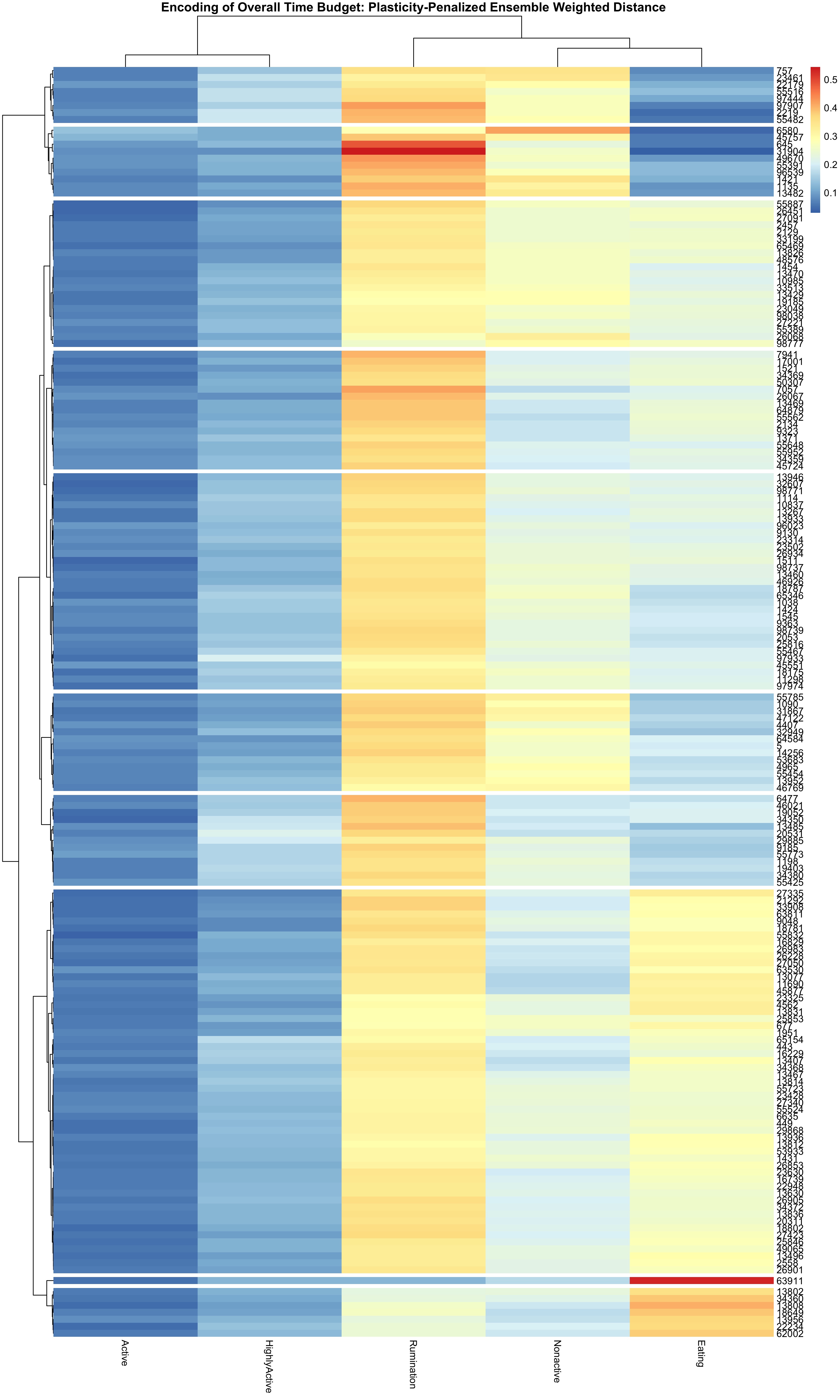

Supplement: Supplementary file 1 [file sensors-22-00001-s001.zip › sensors-1463895-supplementary/OverallTB/OTBEncodings/PlasticityPenalized/_OverallTB_PW_Clean_R10_C0.jpeg]

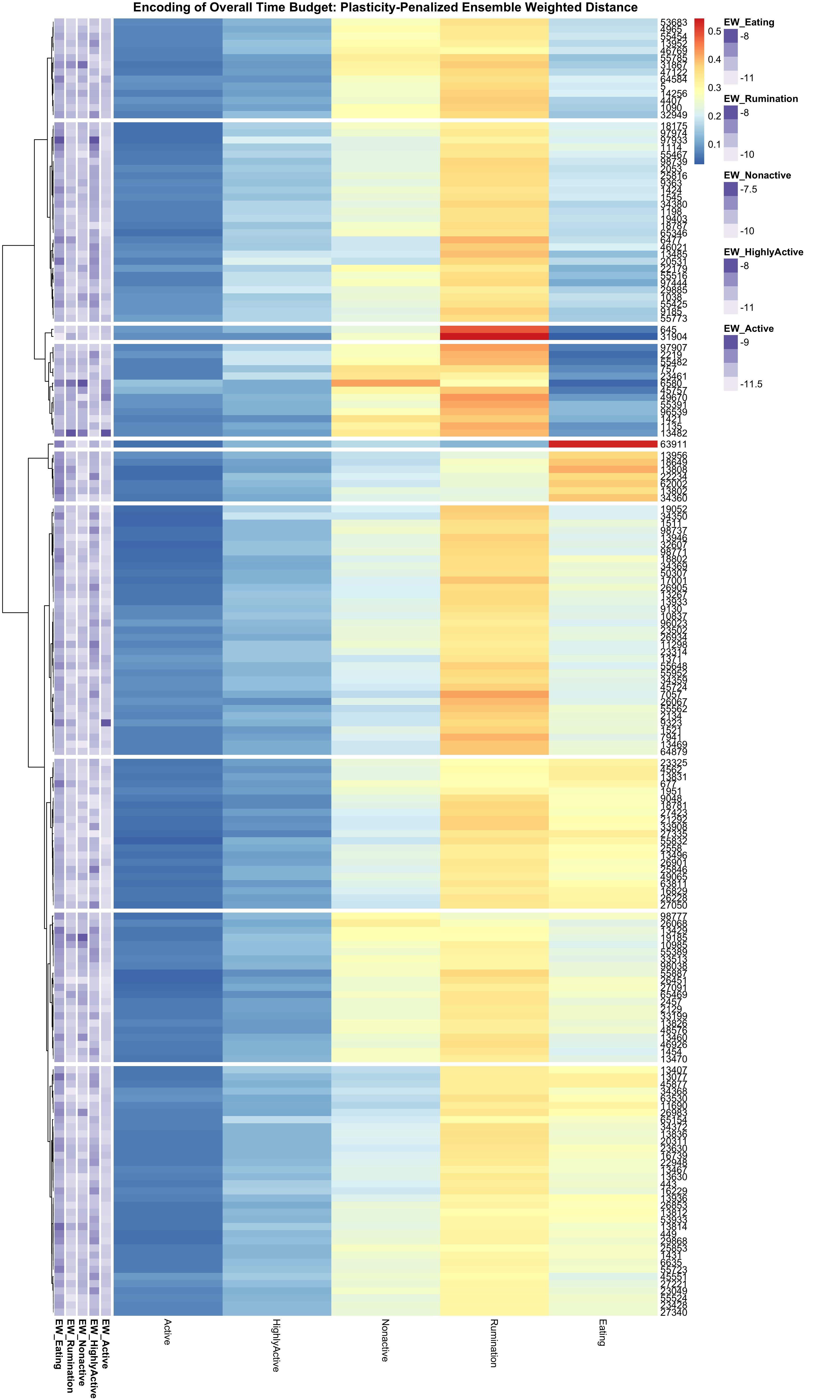

Supplement: Supplementary file 1 [file sensors-22-00001-s001.zip › sensors-1463895-supplementary/OverallTB/OTBEncodings/PlasticityPenalized/_OverallTB_PW_EWVAR_R10_C0.jpeg]

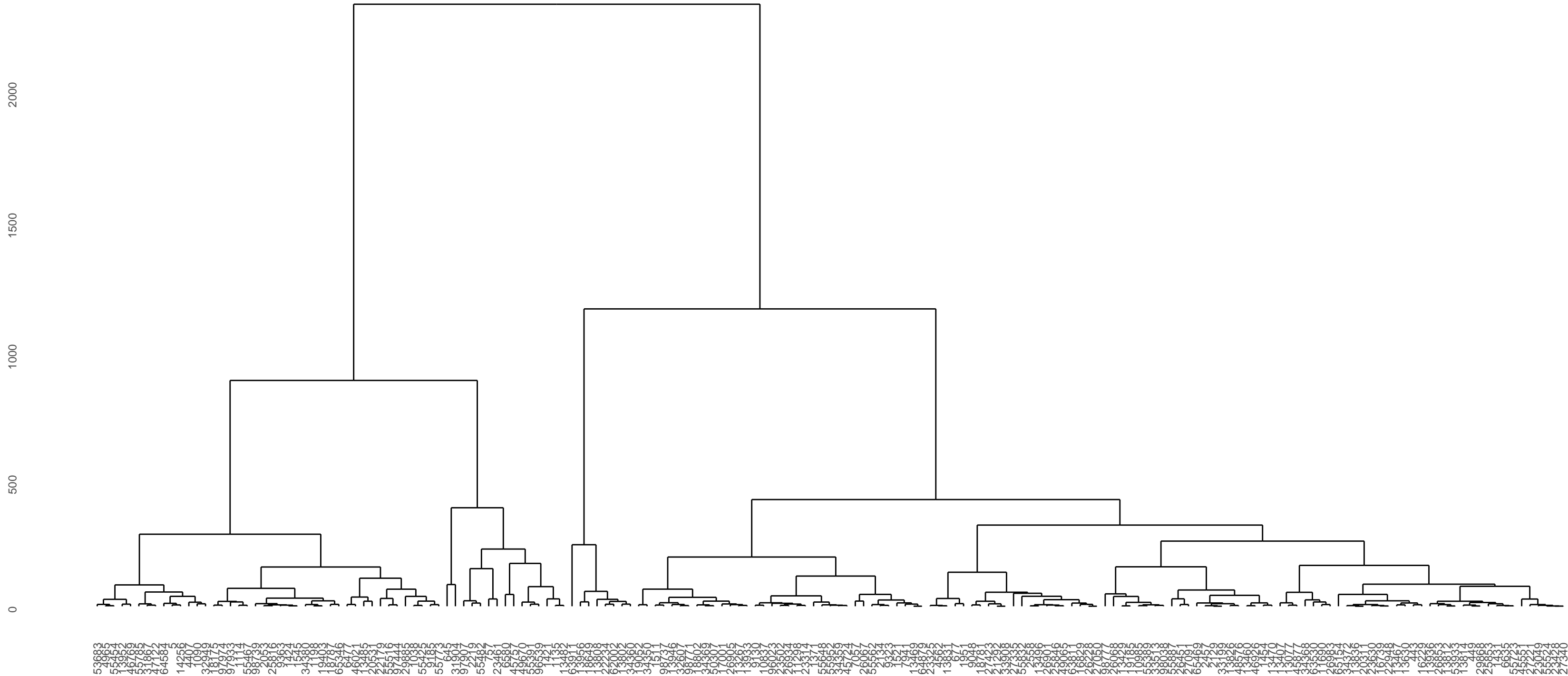

Supplement: Supplementary file 1 [file sensors-22-00001-s001.zip › sensors-1463895-supplementary/OverallTB/OTBEncodings/PlasticityPenalized/_OverallTB_PW_TBVAR_Dendrogram_.pdf]

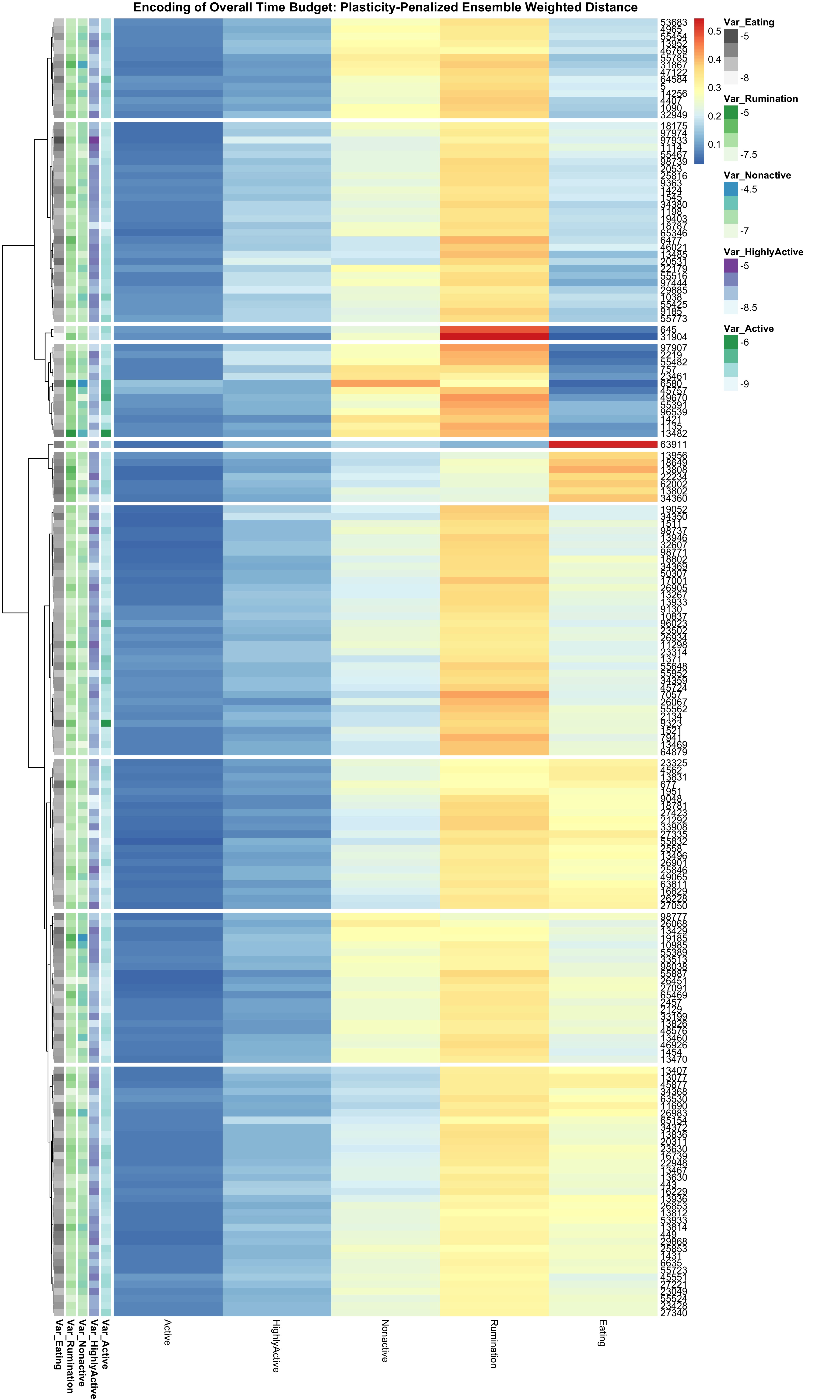

Supplement: Supplementary file 1 [file sensors-22-00001-s001.zip › sensors-1463895-supplementary/OverallTB/OTBEncodings/PlasticityPenalized/_OverallTB_PW_TBVAR_R10_C0.jpeg]
