# Supplementary figures and images for: Livestock Informatics Toolkit: A Case Study in Visually Characterizing Complex Behavioral Patterns across Multiple Sensor Platforms, Using Novel Unsupervised Machine Learning and Information Theoretic Approaches (part 1 of 2)
Source: Sensors (Basel). 2021 Dec 21;22(1):1. doi: 10.3390/s22010001 (PMC8747447; doi:10.3390/s22010001)

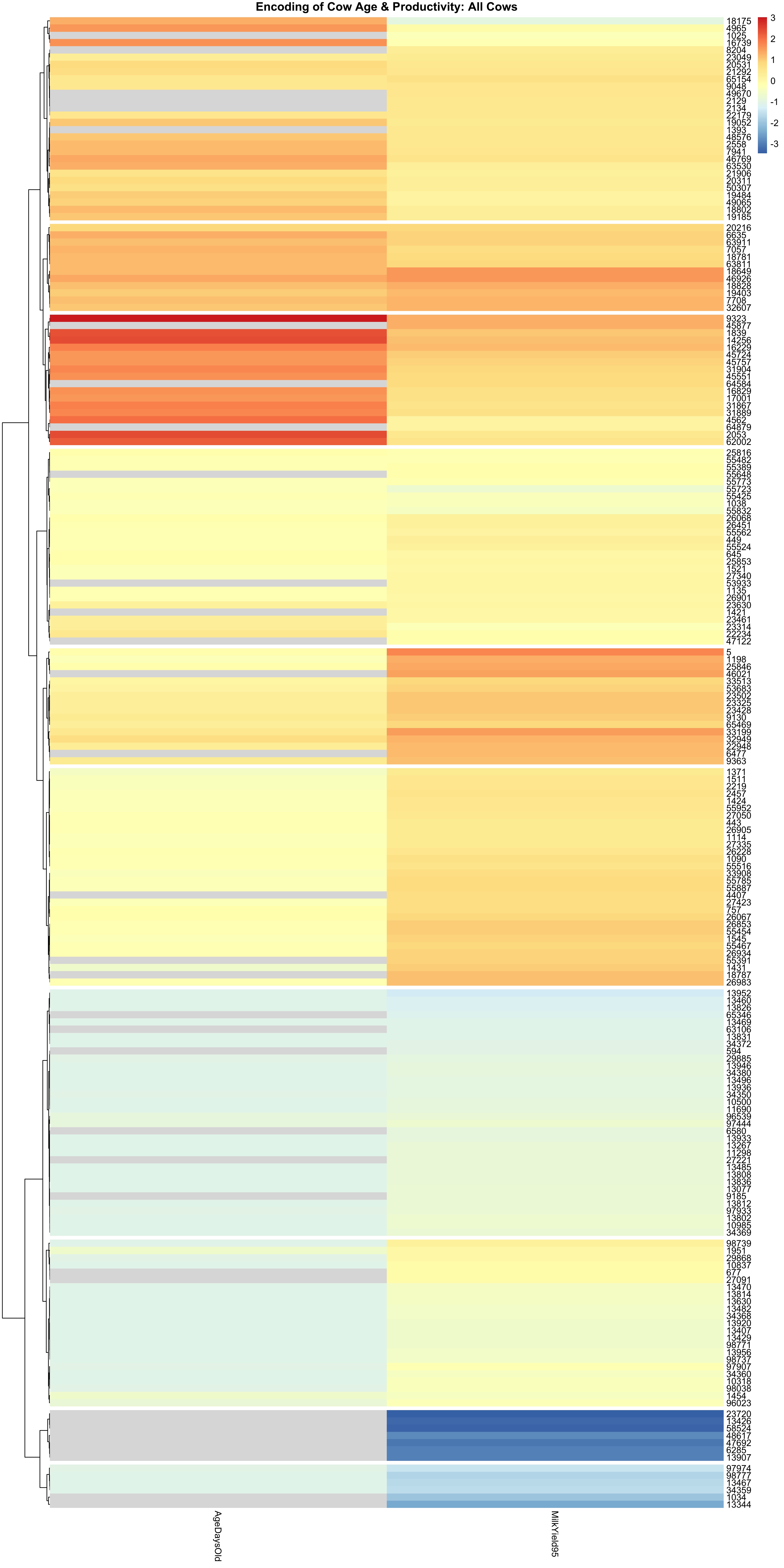

Supplement: Supplementary file 1 [file sensors-22-00001-s001.zip › sensors-1463895-supplementary/OverallTB/BivarTest_AgeYeild/AgeYeildEncoding/All/AgeYeild_R10_C0.jpeg]

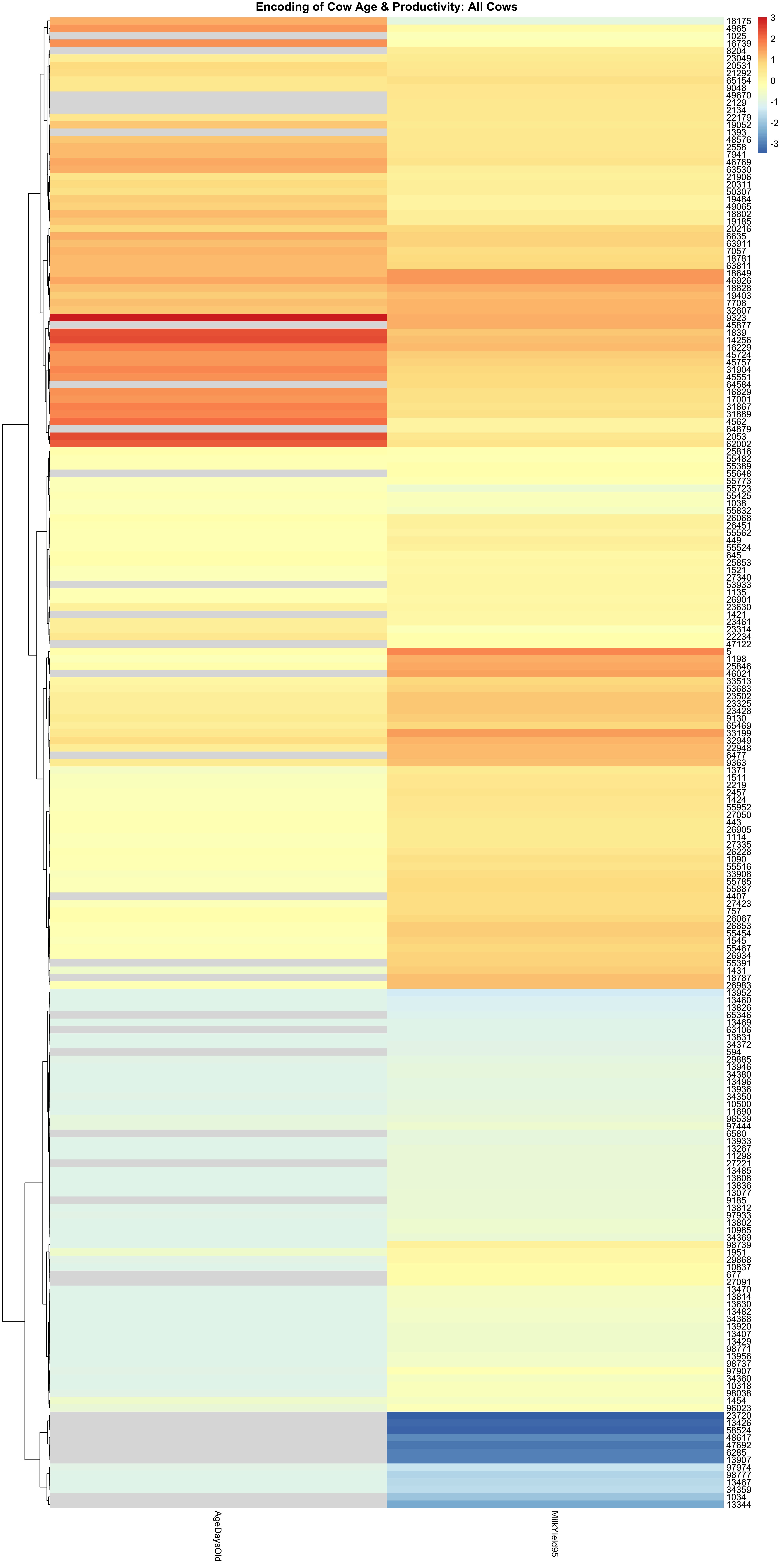

Supplement: Supplementary file 1 [file sensors-22-00001-s001.zip › sensors-1463895-supplementary/OverallTB/BivarTest_AgeYeild/AgeYeildEncoding/All/AgeYeild_R1_C0.jpeg]

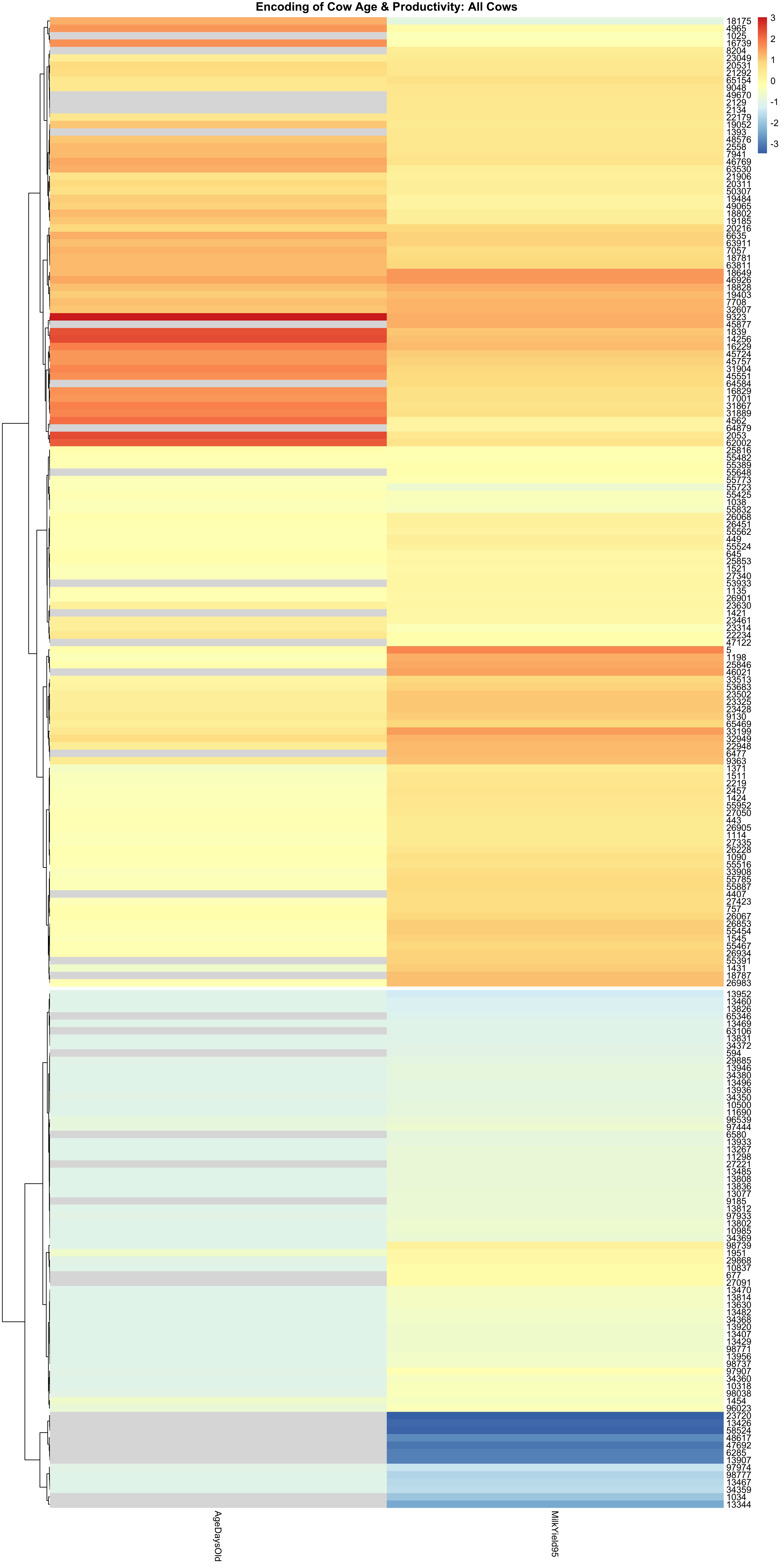

Supplement: Supplementary file 1 [file sensors-22-00001-s001.zip › sensors-1463895-supplementary/OverallTB/BivarTest_AgeYeild/AgeYeildEncoding/All/AgeYeild_R2_C0.jpeg]

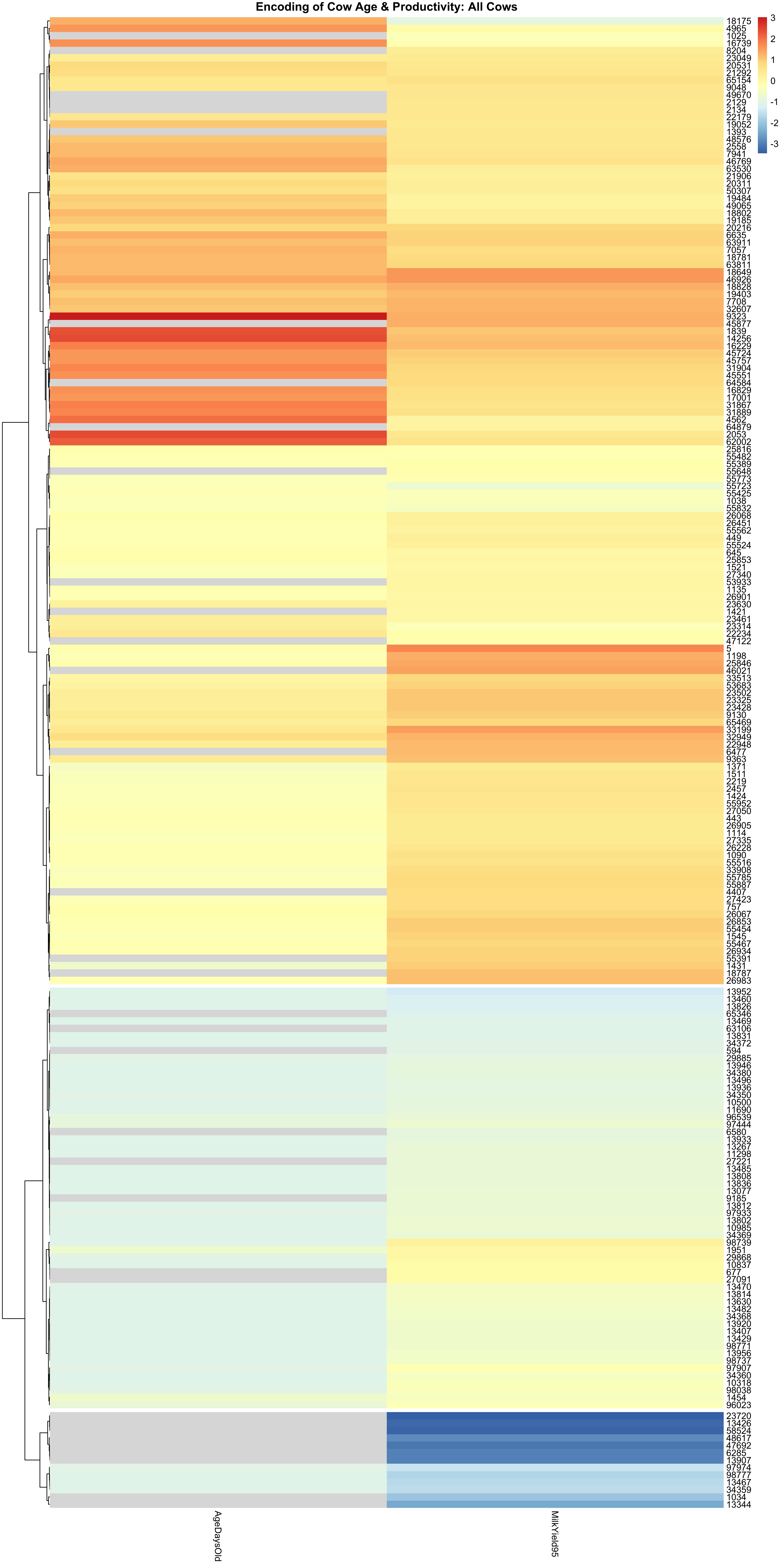

Supplement: Supplementary file 1 [file sensors-22-00001-s001.zip › sensors-1463895-supplementary/OverallTB/BivarTest_AgeYeild/AgeYeildEncoding/All/AgeYeild_R3_C0.jpeg]

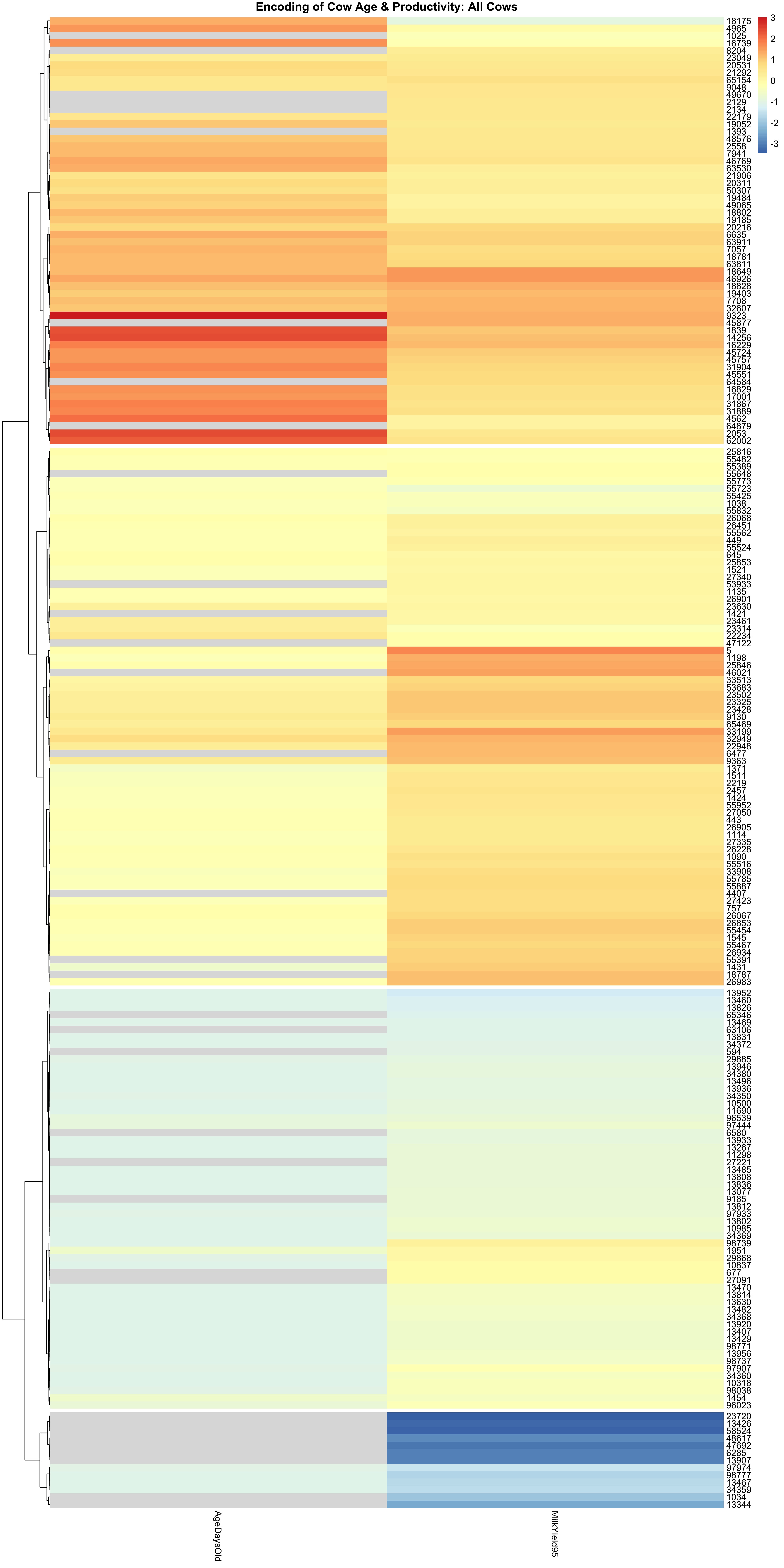

Supplement: Supplementary file 1 [file sensors-22-00001-s001.zip › sensors-1463895-supplementary/OverallTB/BivarTest_AgeYeild/AgeYeildEncoding/All/AgeYeild_R4_C0.jpeg]

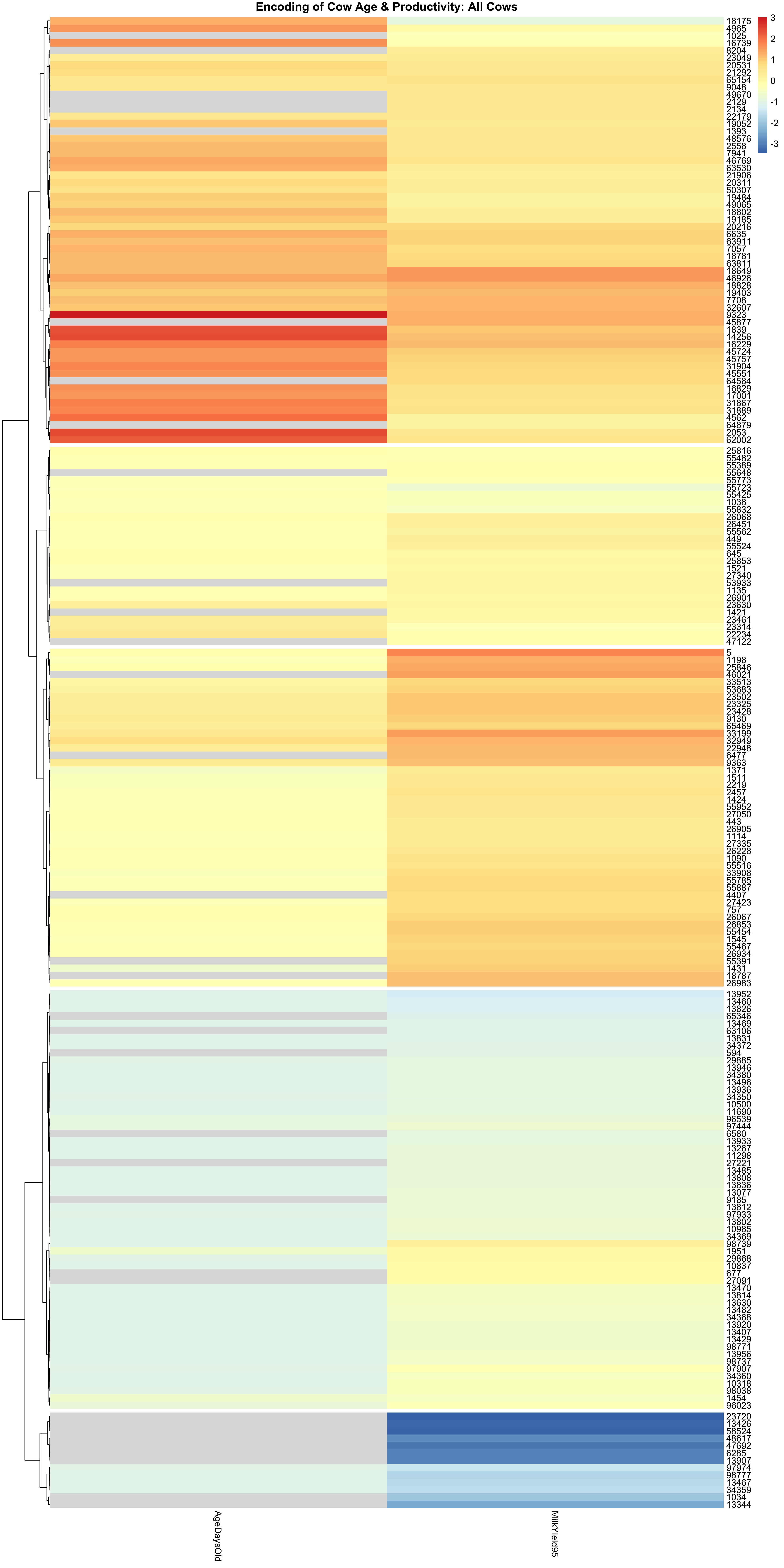

Supplement: Supplementary file 1 [file sensors-22-00001-s001.zip › sensors-1463895-supplementary/OverallTB/BivarTest_AgeYeild/AgeYeildEncoding/All/AgeYeild_R5_C0.jpeg]

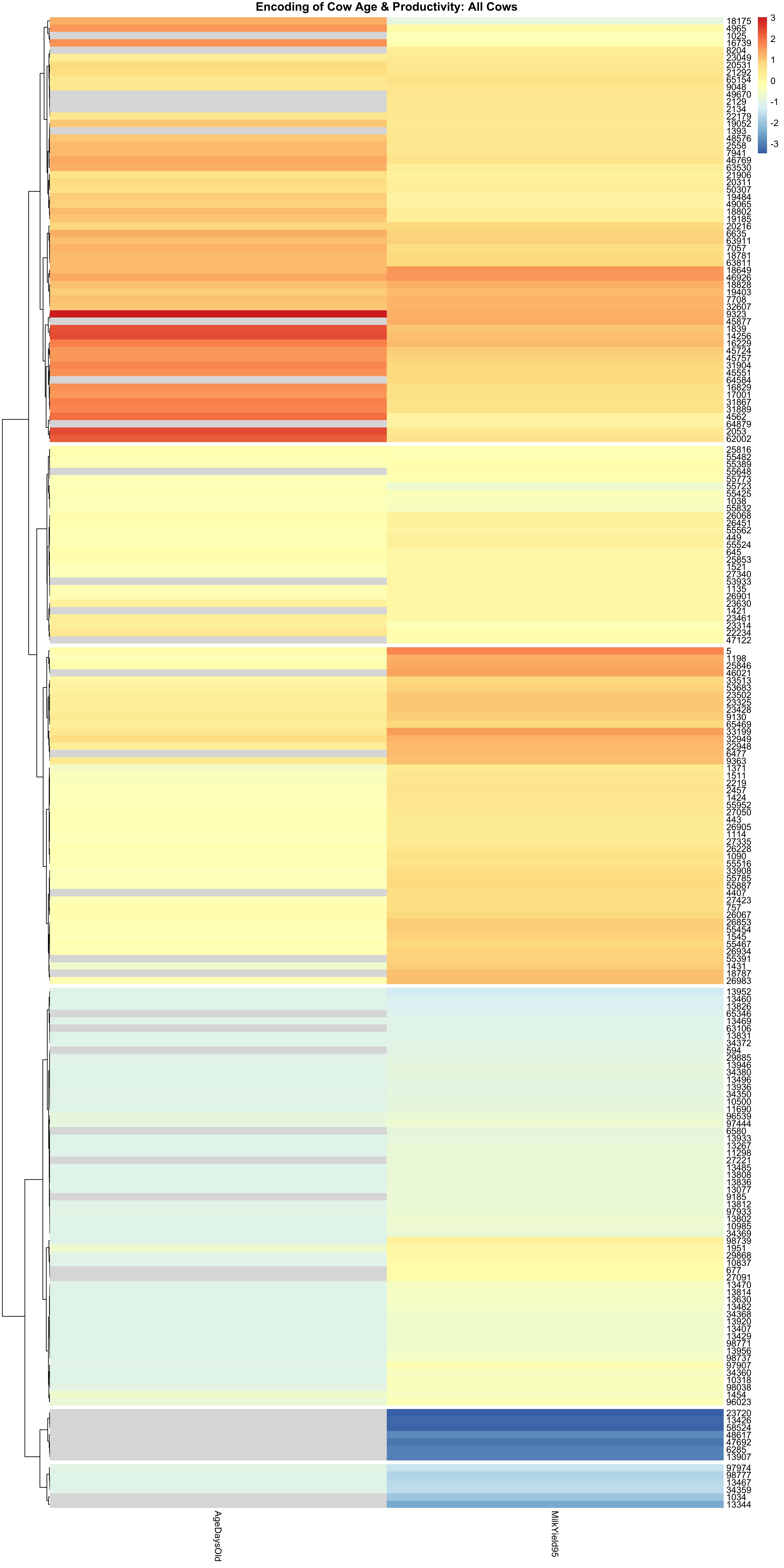

Supplement: Supplementary file 1 [file sensors-22-00001-s001.zip › sensors-1463895-supplementary/OverallTB/BivarTest_AgeYeild/AgeYeildEncoding/All/AgeYeild_R6_C0.jpeg]

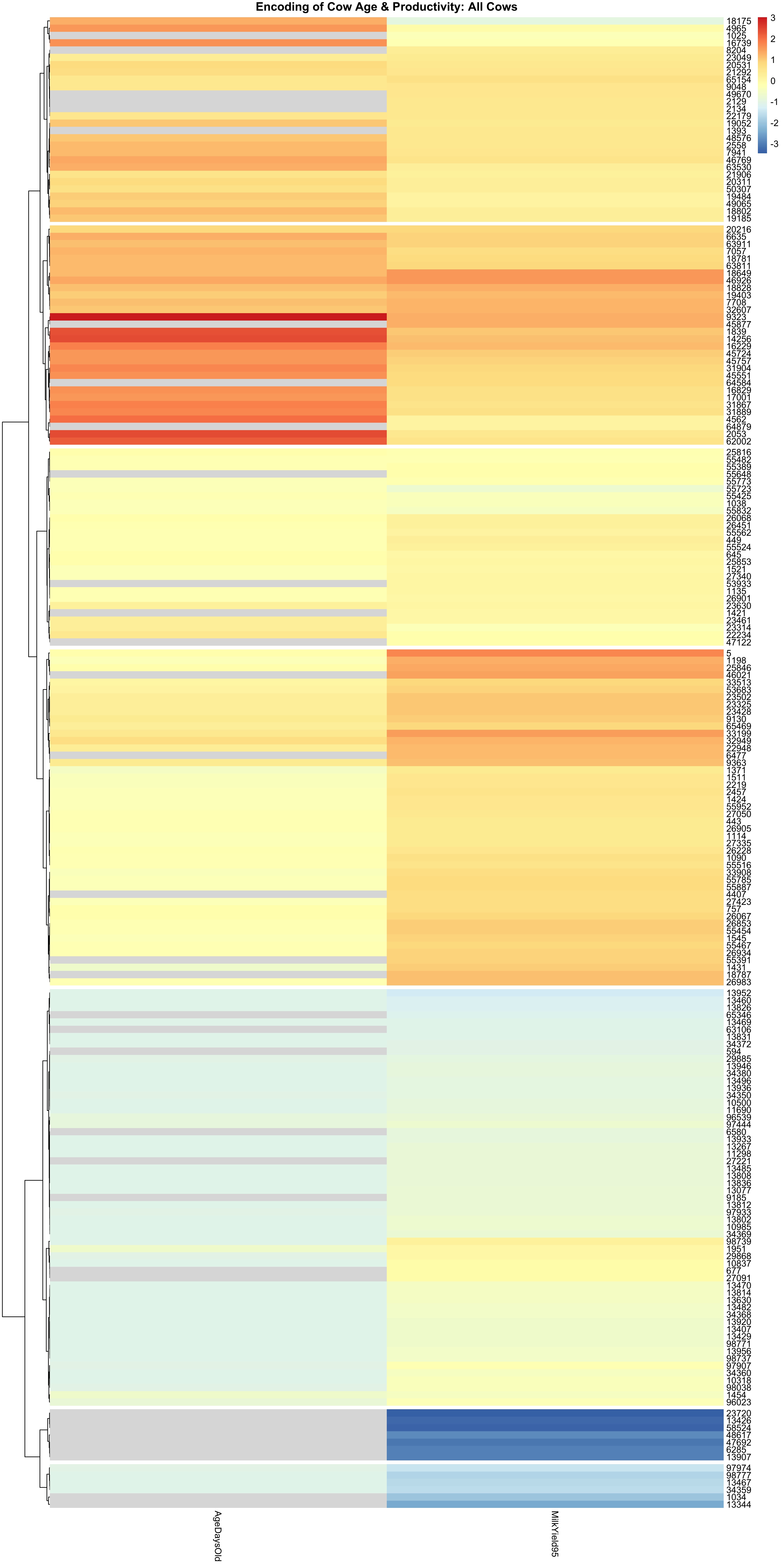

Supplement: Supplementary file 1 [file sensors-22-00001-s001.zip › sensors-1463895-supplementary/OverallTB/BivarTest_AgeYeild/AgeYeildEncoding/All/AgeYeild_R7_C0.jpeg]

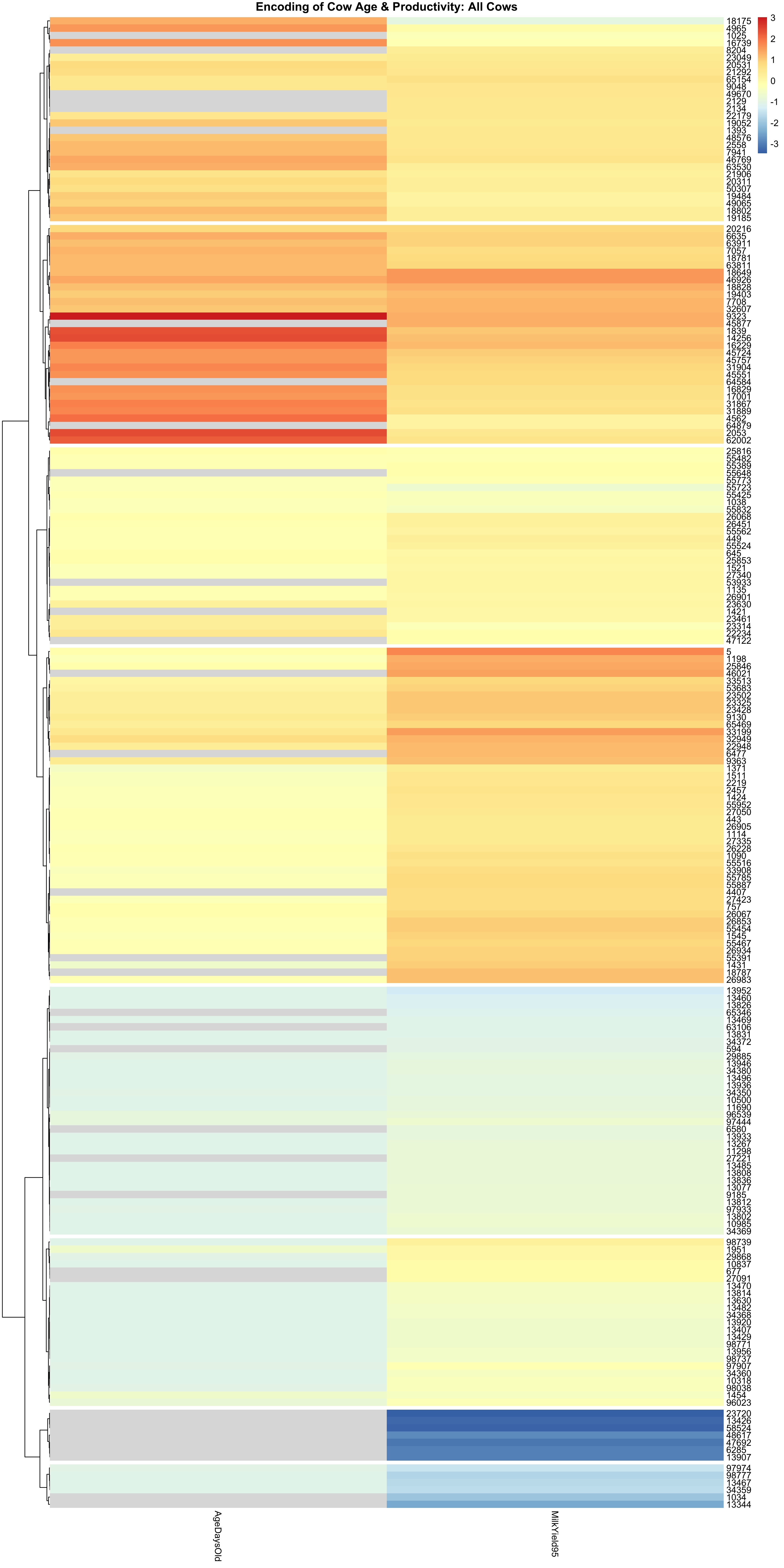

Supplement: Supplementary file 1 [file sensors-22-00001-s001.zip › sensors-1463895-supplementary/OverallTB/BivarTest_AgeYeild/AgeYeildEncoding/All/AgeYeild_R8_C0.jpeg]

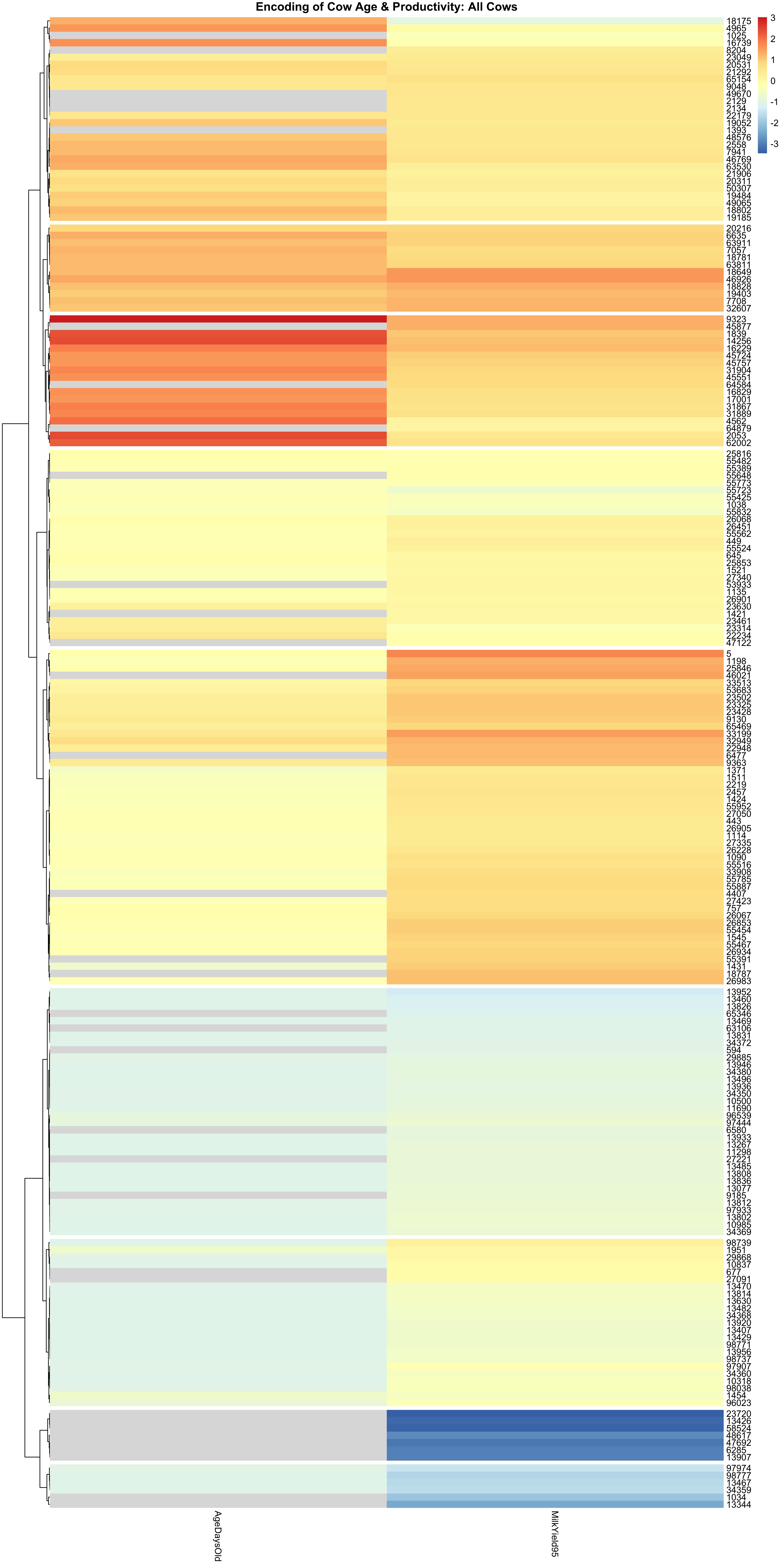

Supplement: Supplementary file 1 [file sensors-22-00001-s001.zip › sensors-1463895-supplementary/OverallTB/BivarTest_AgeYeild/AgeYeildEncoding/All/AgeYeild_R9_C0.jpeg]

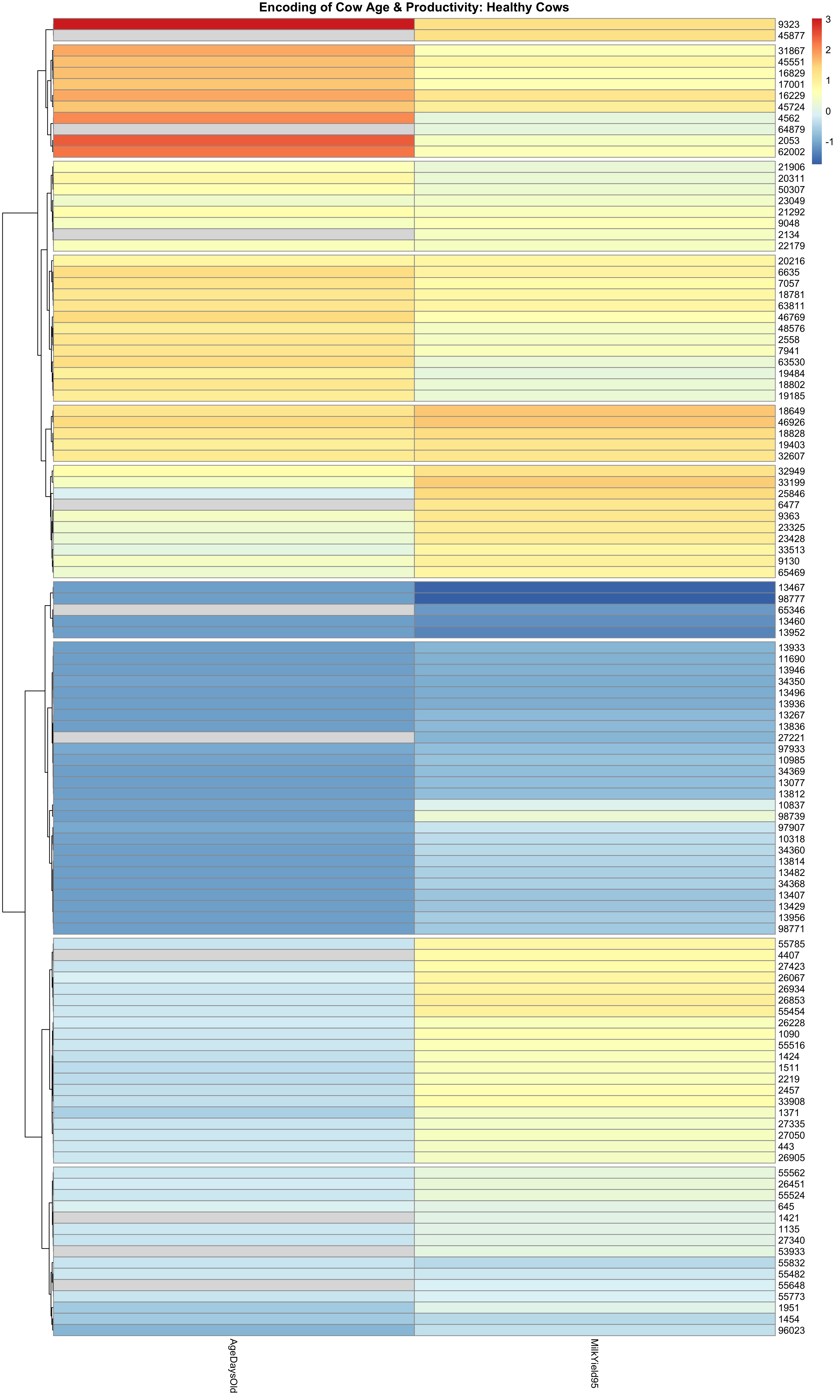

Supplement: Supplementary file 1 [file sensors-22-00001-s001.zip › sensors-1463895-supplementary/OverallTB/BivarTest_AgeYeild/AgeYeildEncoding/Healthy/AgeYeild_R10_C0.jpeg]

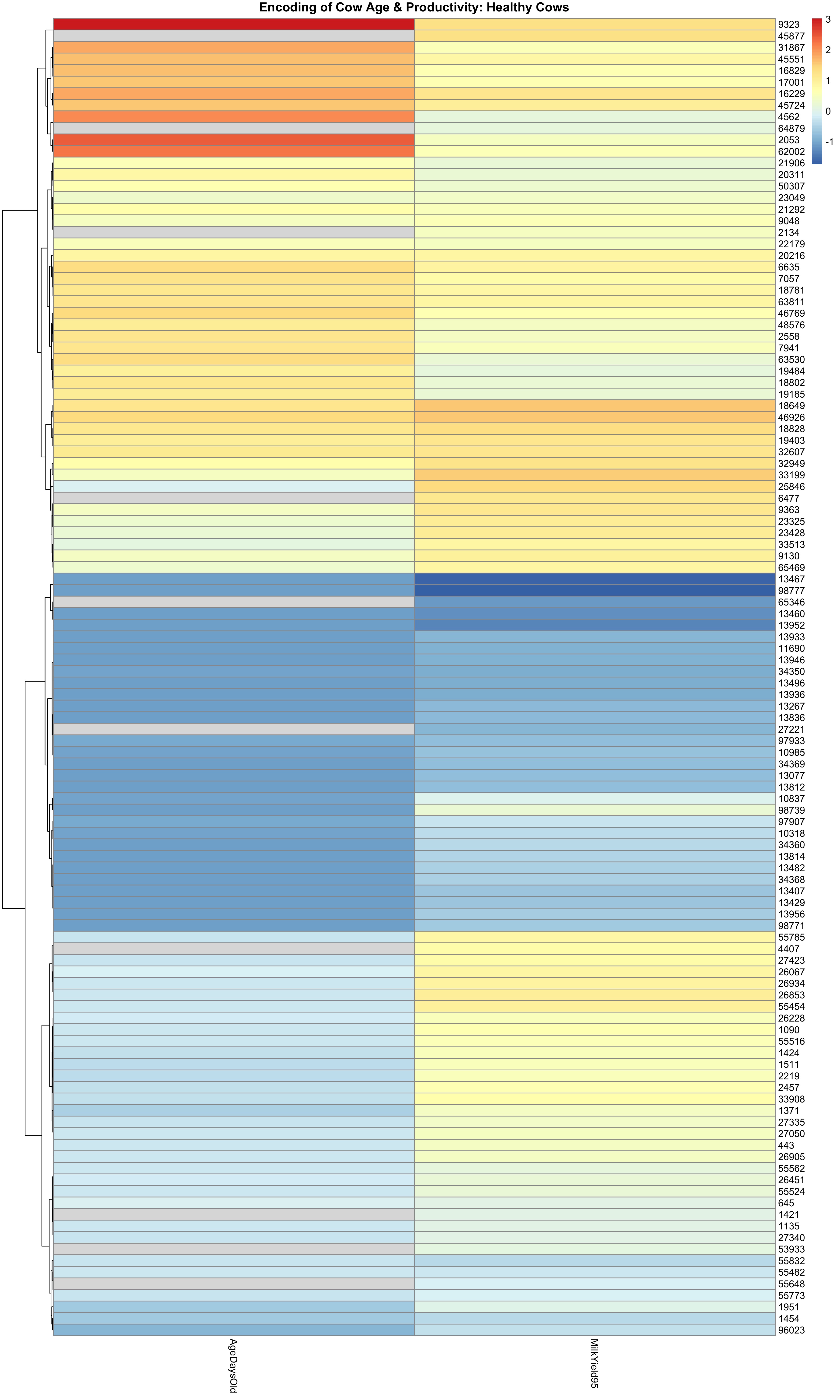

Supplement: Supplementary file 1 [file sensors-22-00001-s001.zip › sensors-1463895-supplementary/OverallTB/BivarTest_AgeYeild/AgeYeildEncoding/Healthy/AgeYeild_R1_C0.jpeg]

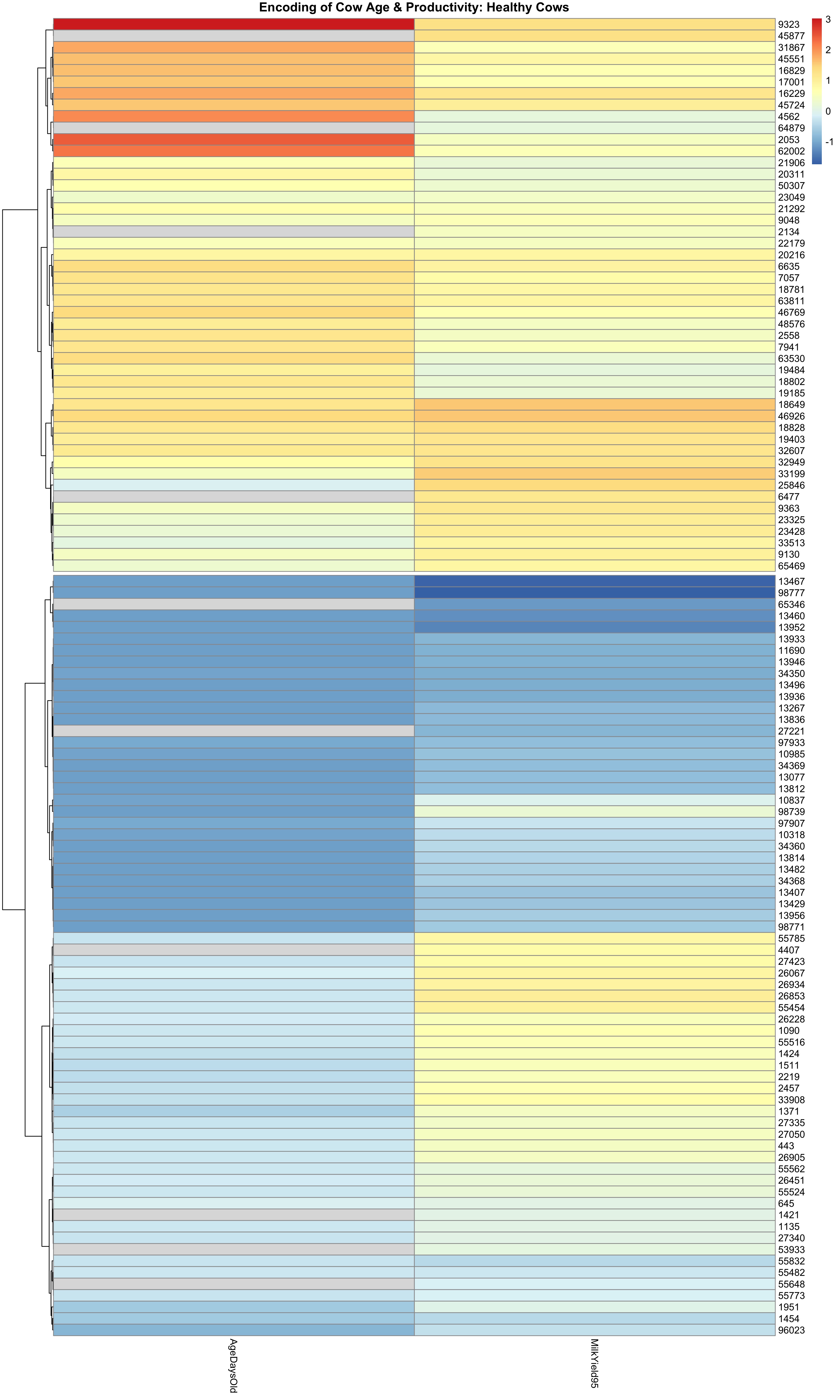

Supplement: Supplementary file 1 [file sensors-22-00001-s001.zip › sensors-1463895-supplementary/OverallTB/BivarTest_AgeYeild/AgeYeildEncoding/Healthy/AgeYeild_R2_C0.jpeg]

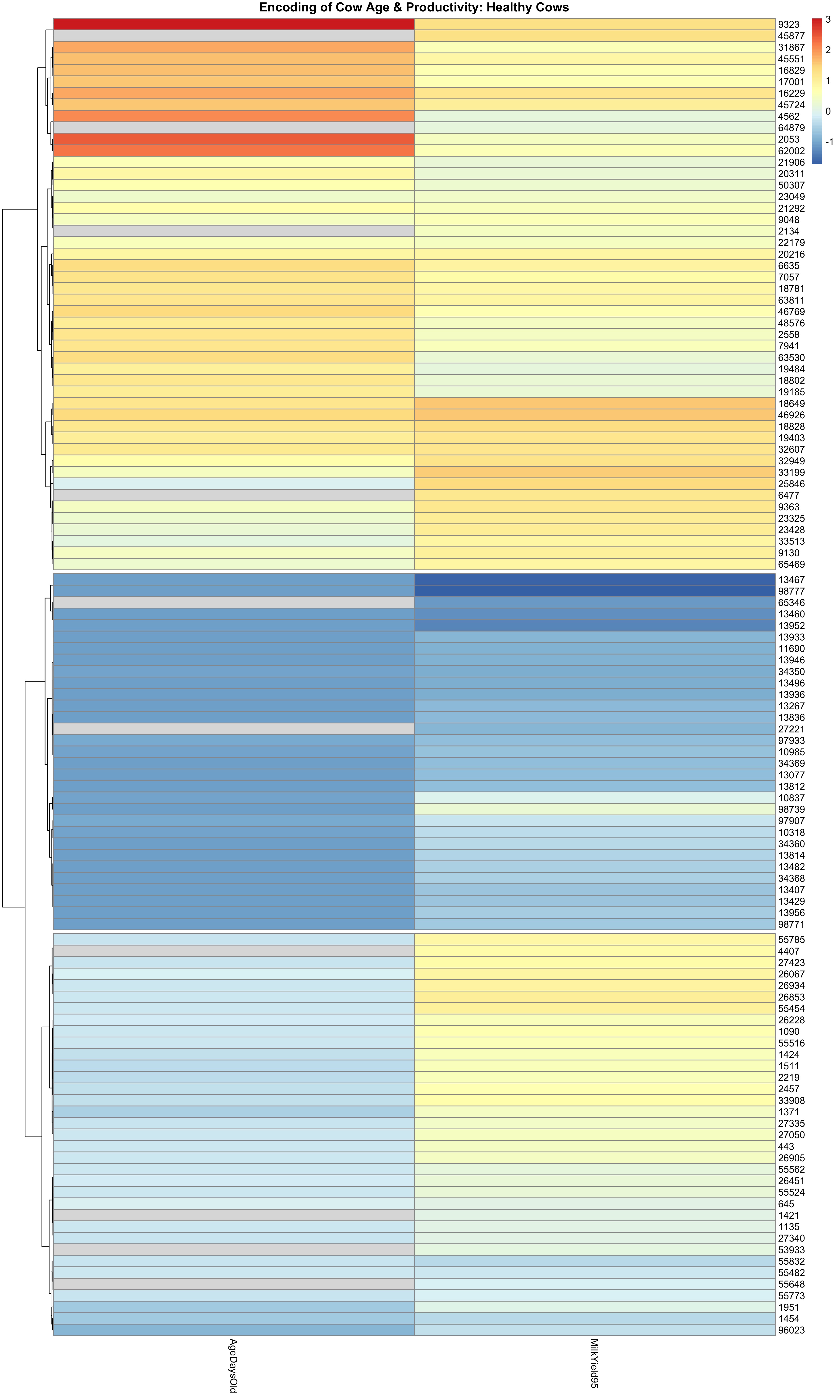

Supplement: Supplementary file 1 [file sensors-22-00001-s001.zip › sensors-1463895-supplementary/OverallTB/BivarTest_AgeYeild/AgeYeildEncoding/Healthy/AgeYeild_R3_C0.jpeg]

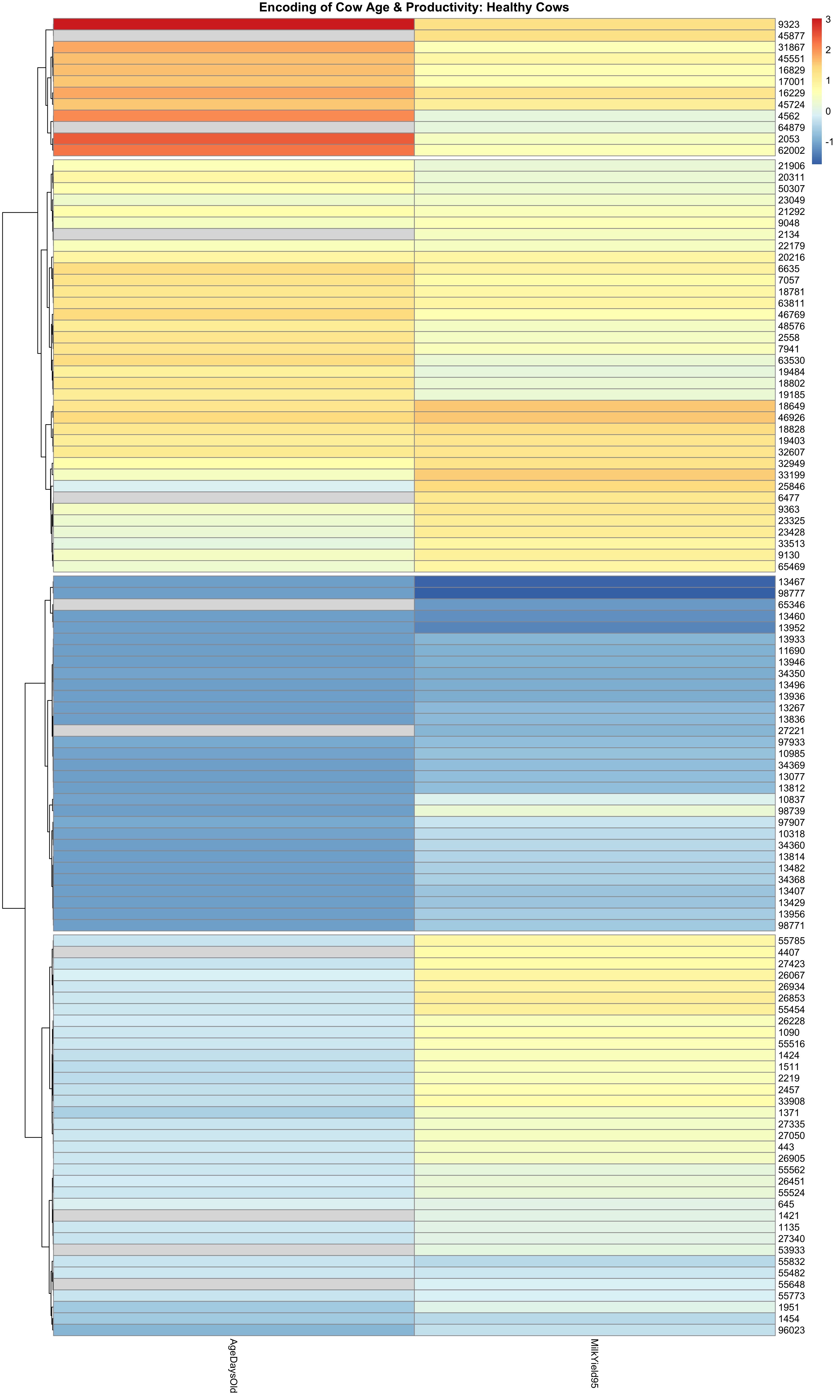

Supplement: Supplementary file 1 [file sensors-22-00001-s001.zip › sensors-1463895-supplementary/OverallTB/BivarTest_AgeYeild/AgeYeildEncoding/Healthy/AgeYeild_R4_C0.jpeg]

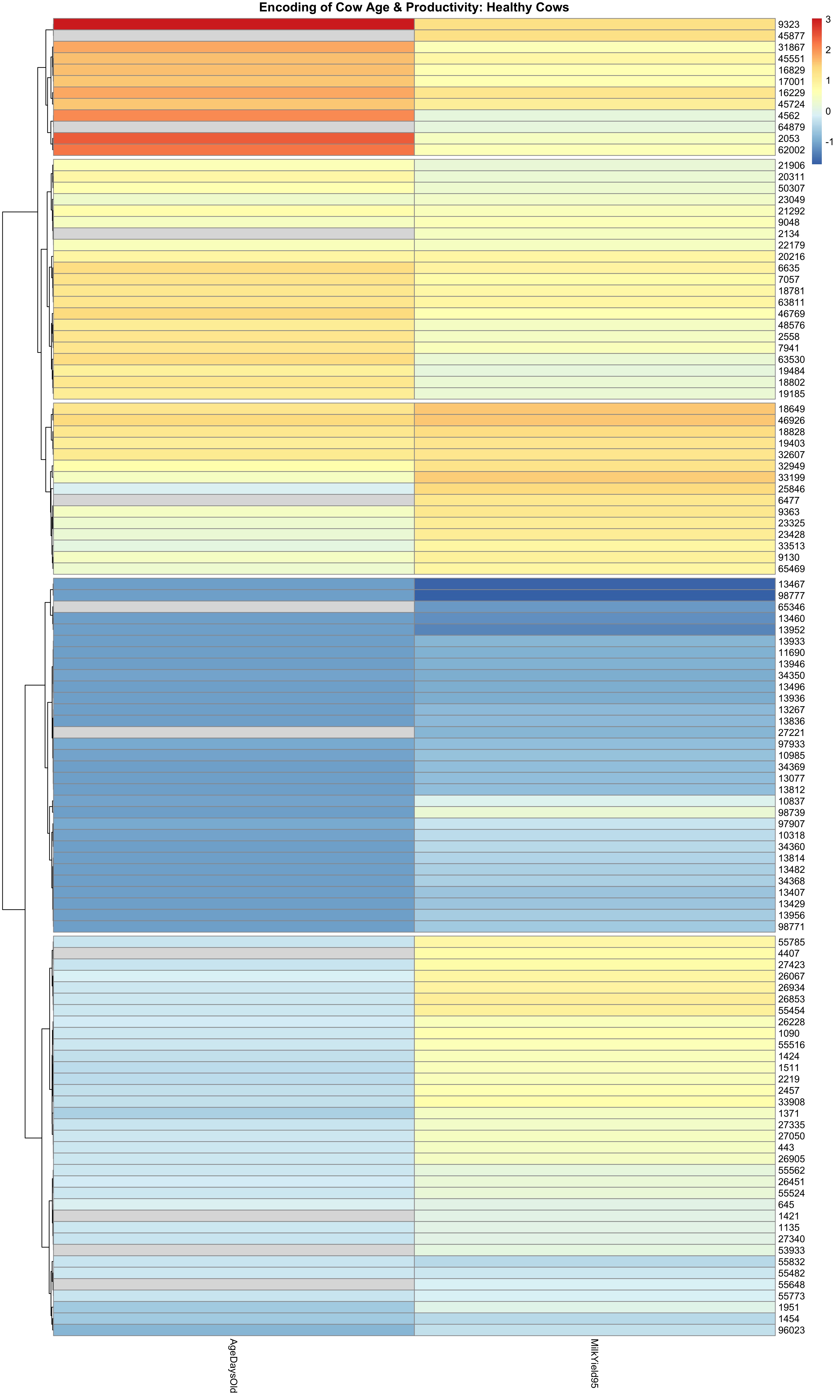

Supplement: Supplementary file 1 [file sensors-22-00001-s001.zip › sensors-1463895-supplementary/OverallTB/BivarTest_AgeYeild/AgeYeildEncoding/Healthy/AgeYeild_R5_C0.jpeg]

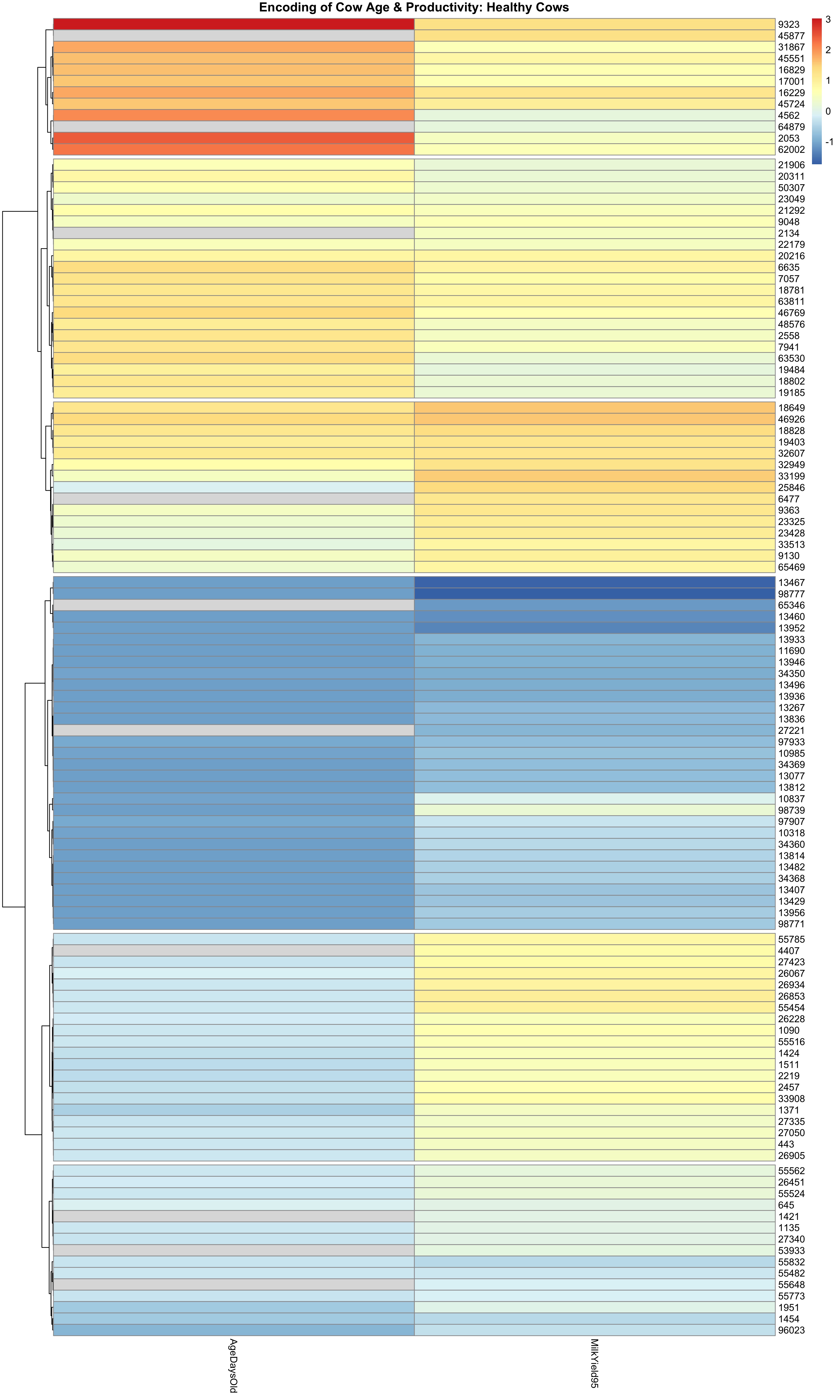

Supplement: Supplementary file 1 [file sensors-22-00001-s001.zip › sensors-1463895-supplementary/OverallTB/BivarTest_AgeYeild/AgeYeildEncoding/Healthy/AgeYeild_R6_C0.jpeg]

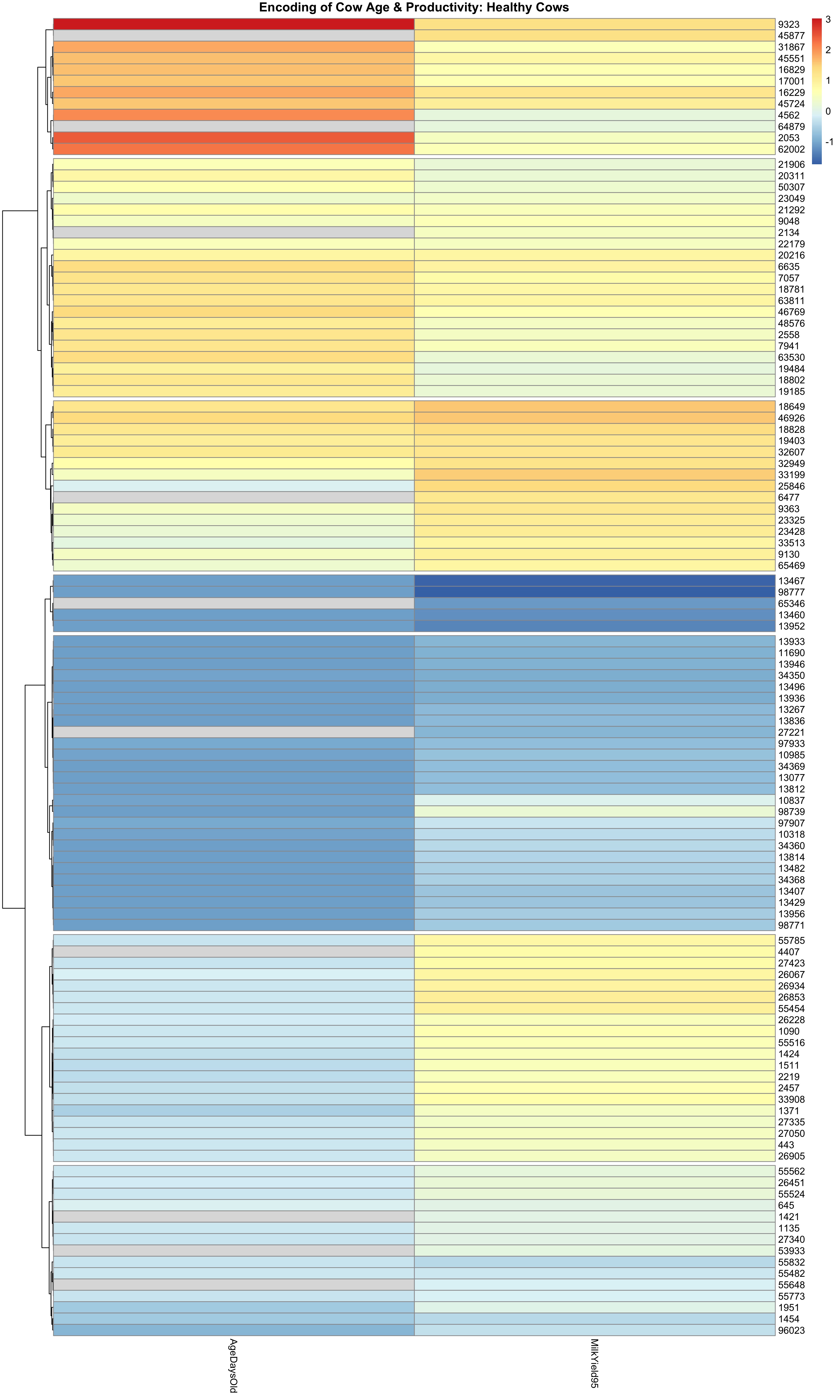

Supplement: Supplementary file 1 [file sensors-22-00001-s001.zip › sensors-1463895-supplementary/OverallTB/BivarTest_AgeYeild/AgeYeildEncoding/Healthy/AgeYeild_R7_C0.jpeg]

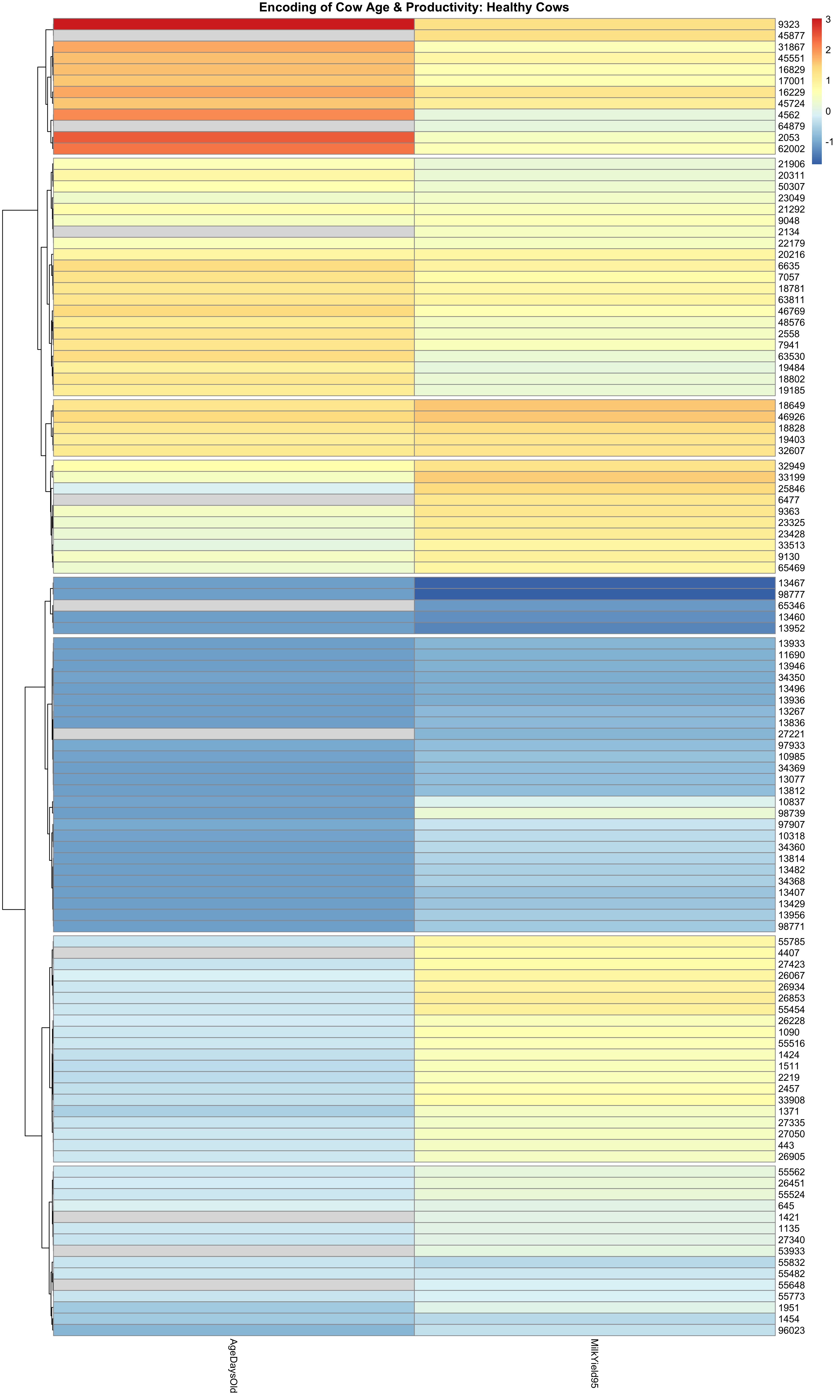

Supplement: Supplementary file 1 [file sensors-22-00001-s001.zip › sensors-1463895-supplementary/OverallTB/BivarTest_AgeYeild/AgeYeildEncoding/Healthy/AgeYeild_R8_C0.jpeg]

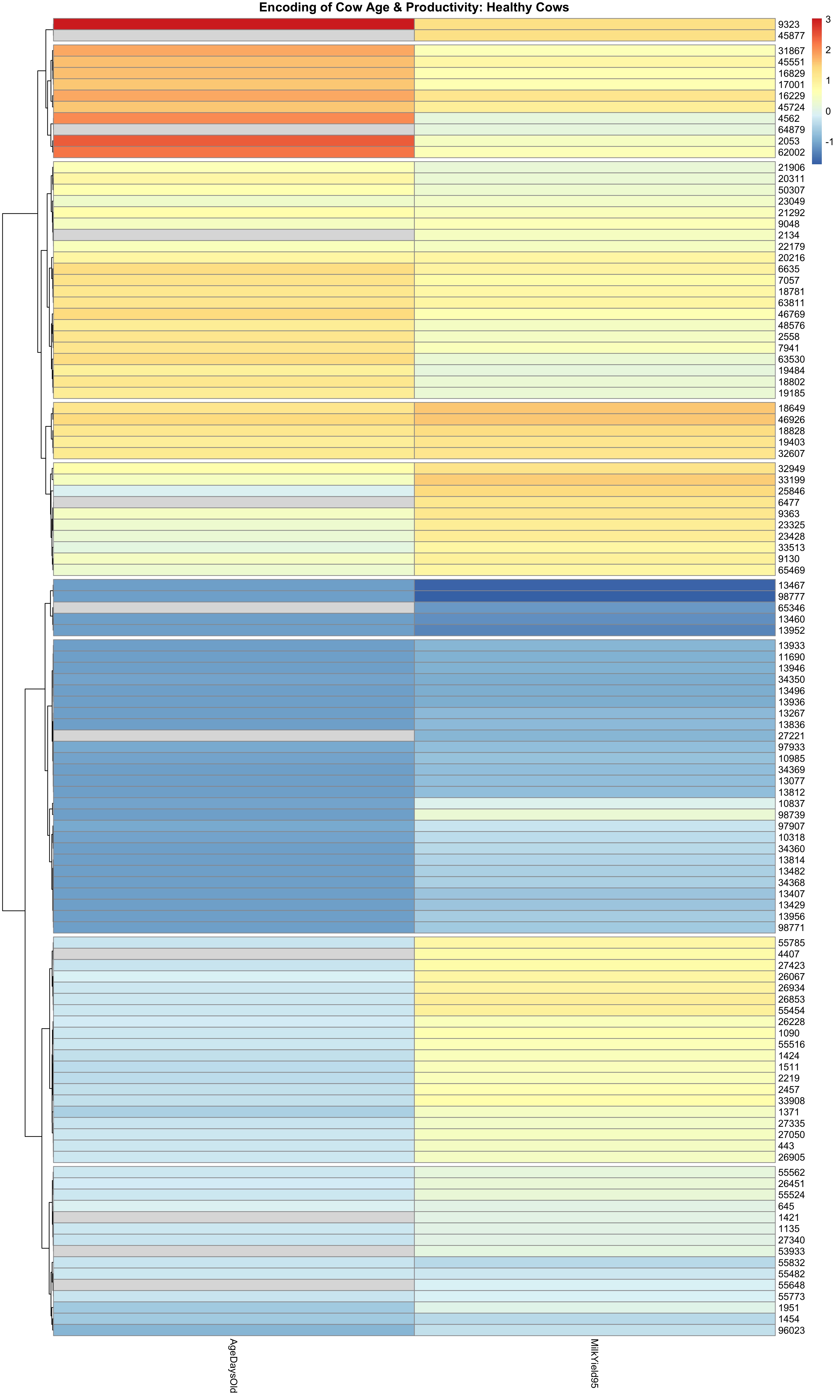

Supplement: Supplementary file 1 [file sensors-22-00001-s001.zip › sensors-1463895-supplementary/OverallTB/BivarTest_AgeYeild/AgeYeildEncoding/Healthy/AgeYeild_R9_C0.jpeg]

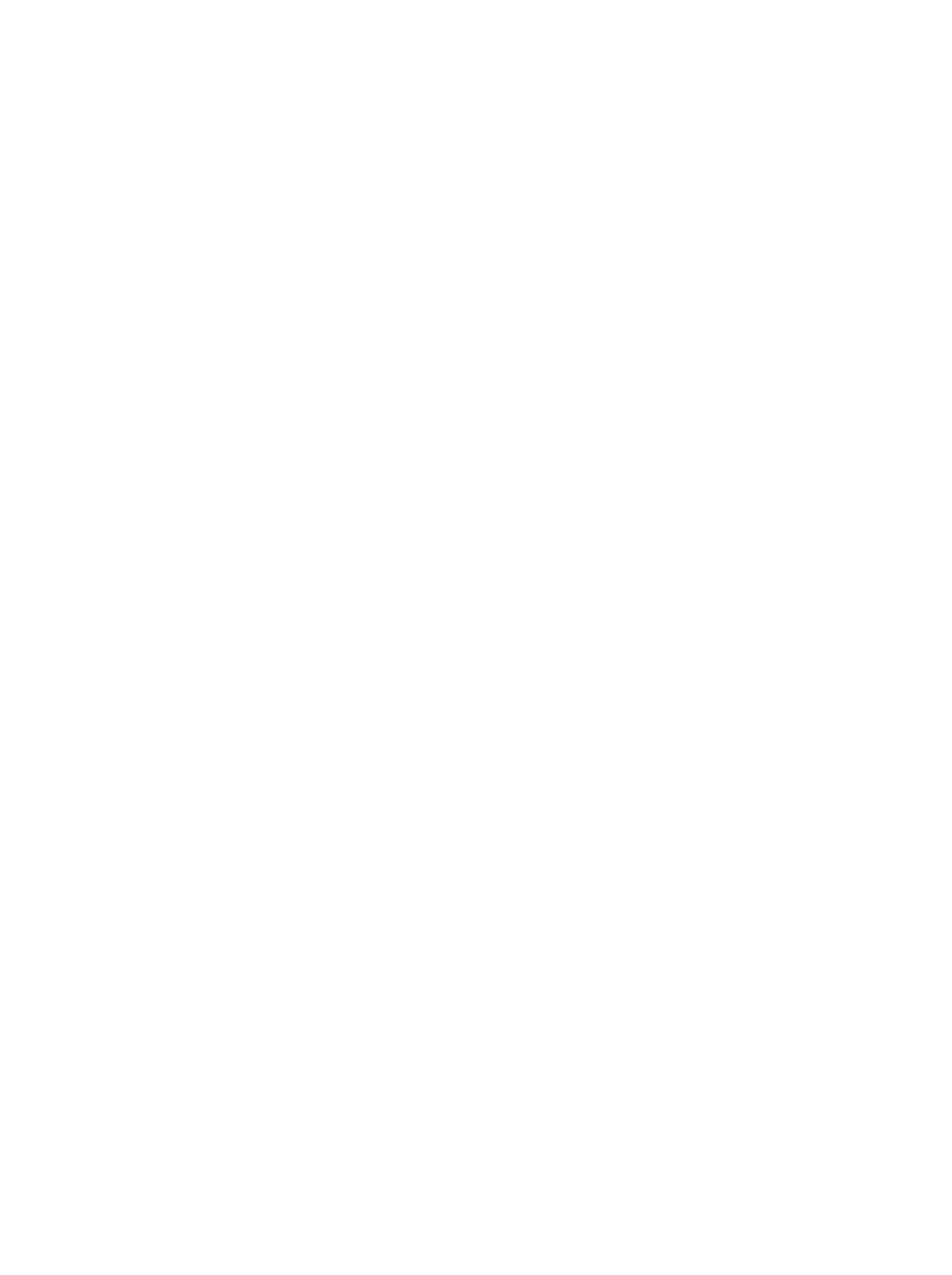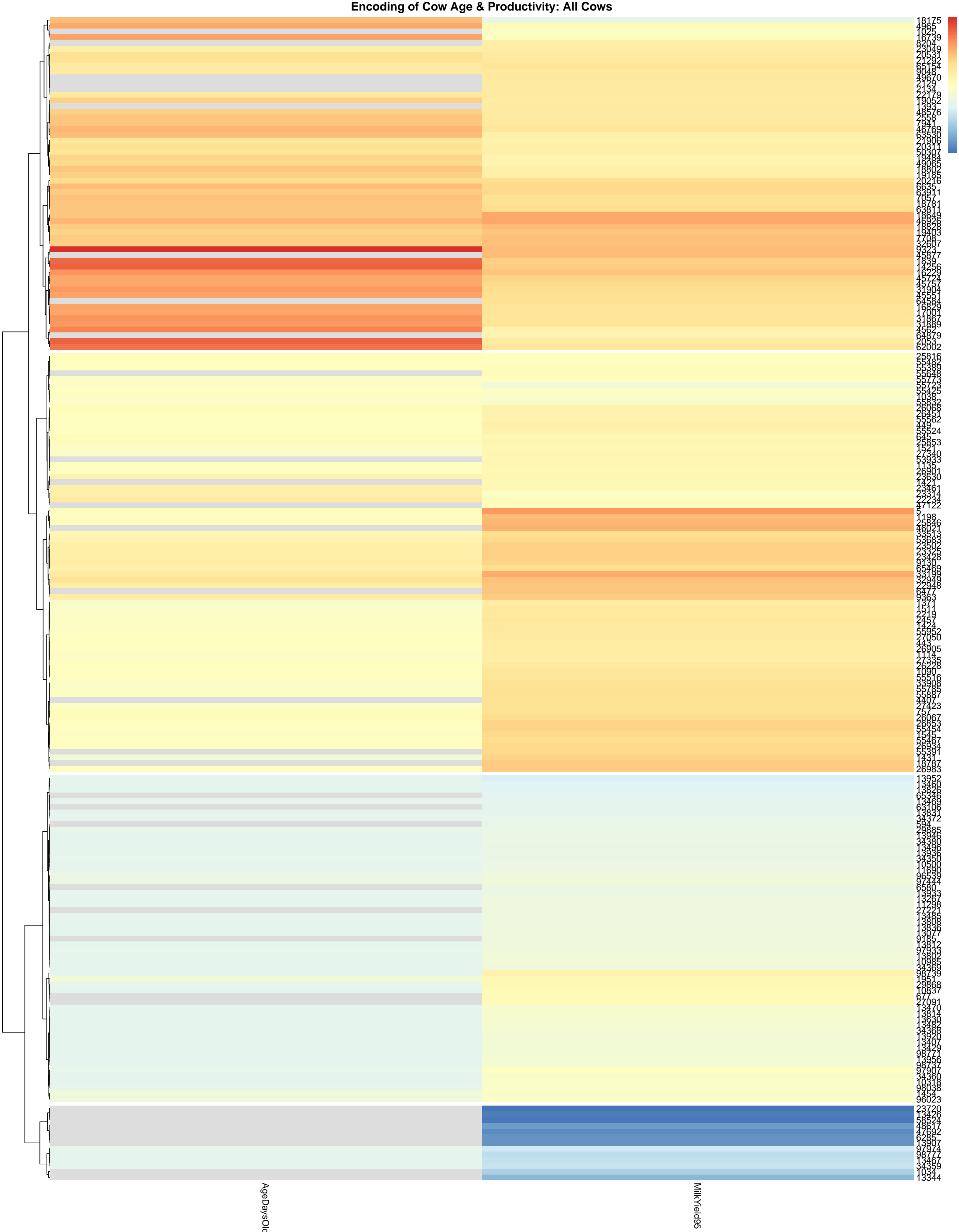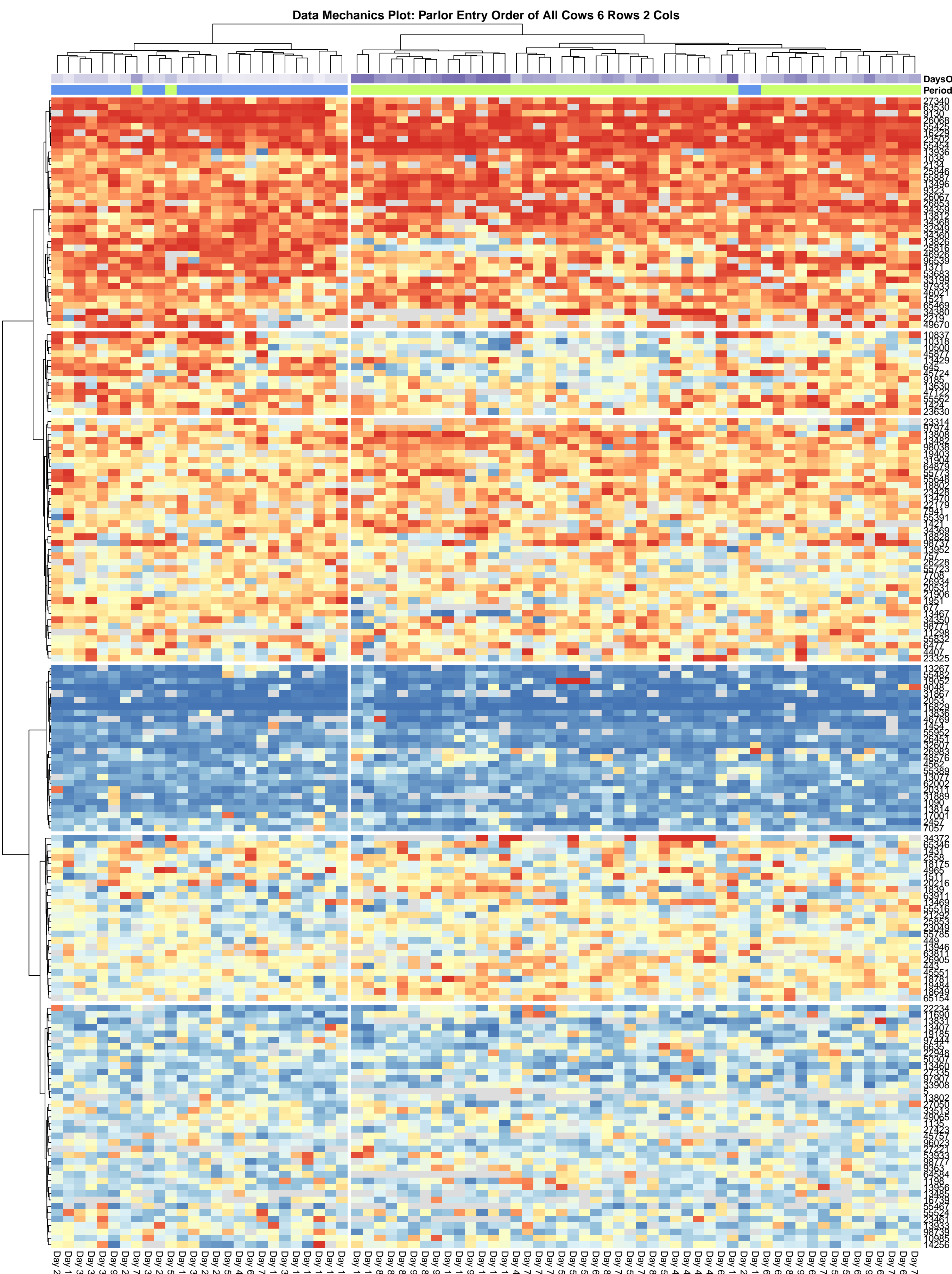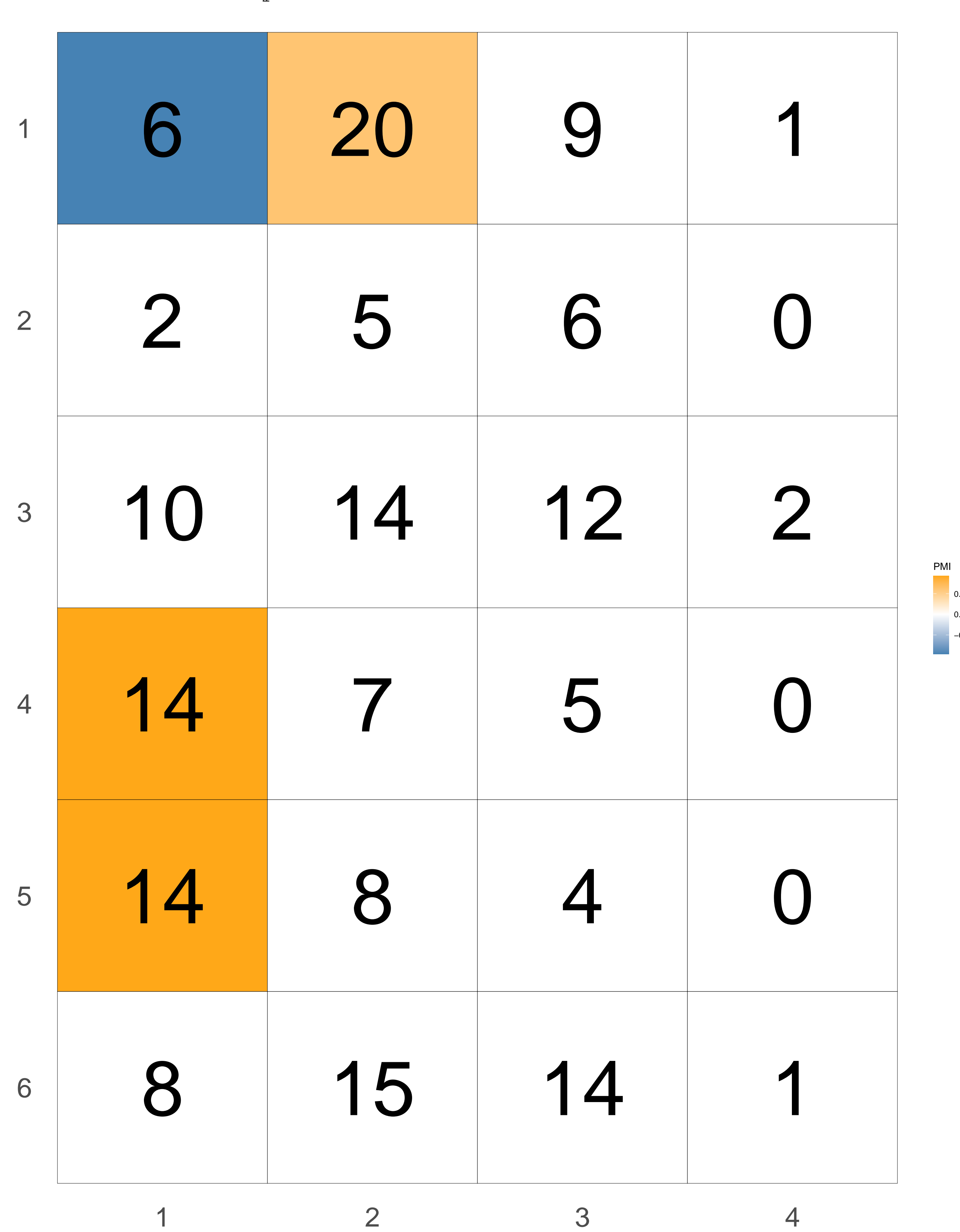

Supplement: Supplementary file 1 [file sensors-22-00001-s001.zip › sensors-1463895-supplementary/OverallTB/BivarTest_AgeYeild/EO_All.pdf]

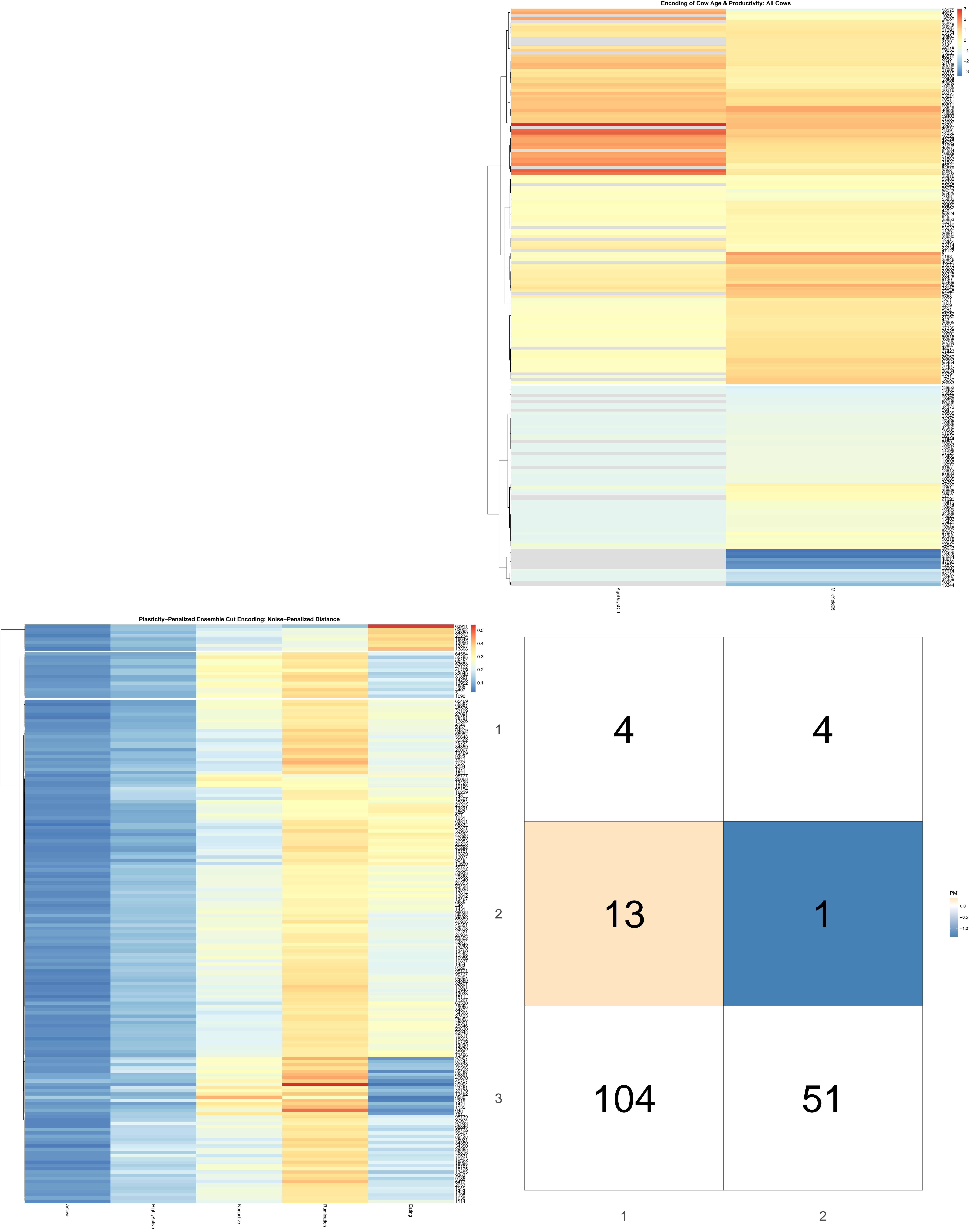

Supplement: Supplementary file 1 [file sensors-22-00001-s001.zip › sensors-1463895-supplementary/OverallTB/BivarTest_AgeYeild/OTB_All_NP.pdf]

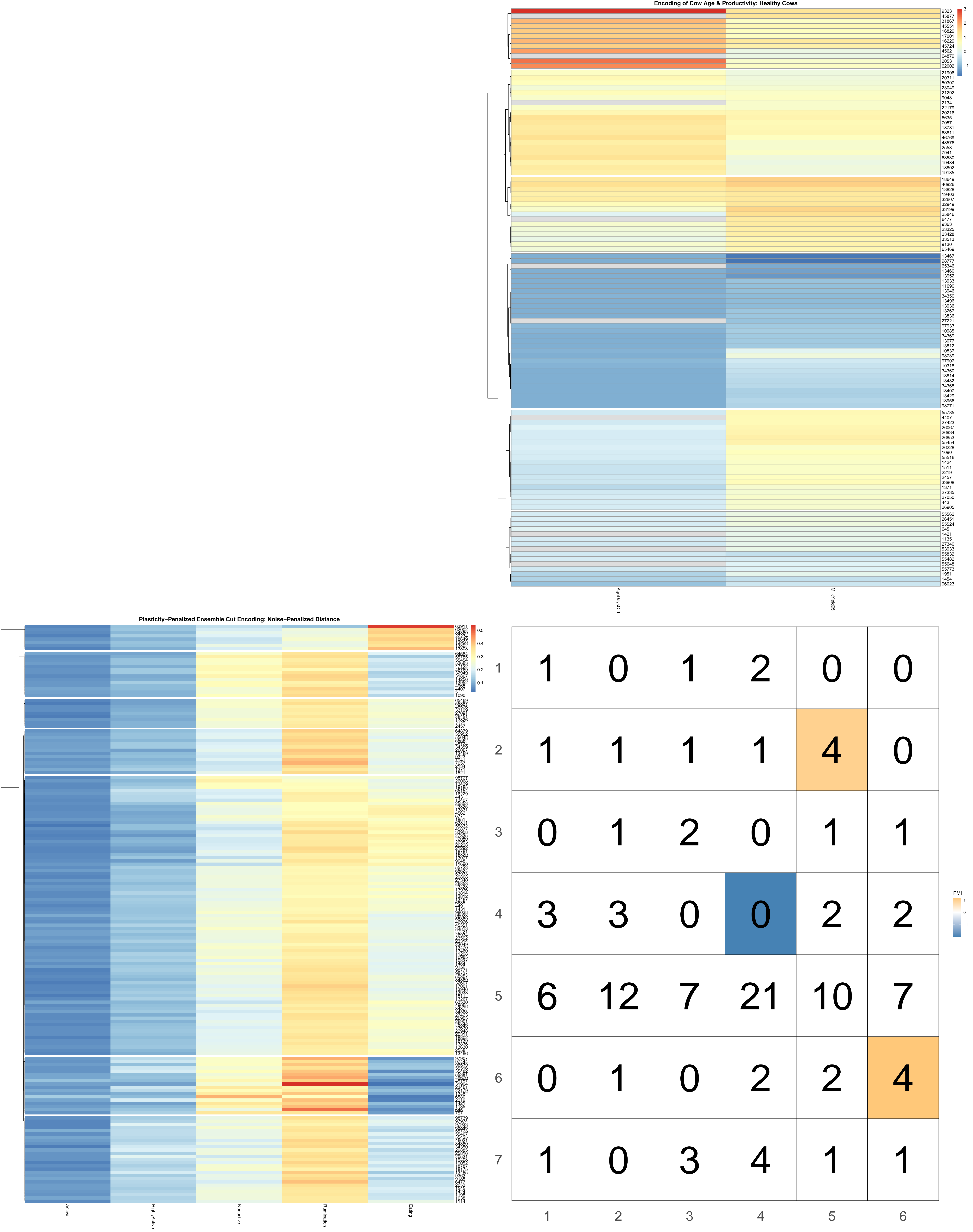

Supplement: Supplementary file 1 [file sensors-22-00001-s001.zip › sensors-1463895-supplementary/OverallTB/BivarTest_AgeYeild/OTB_Healthy_NP.pdf]

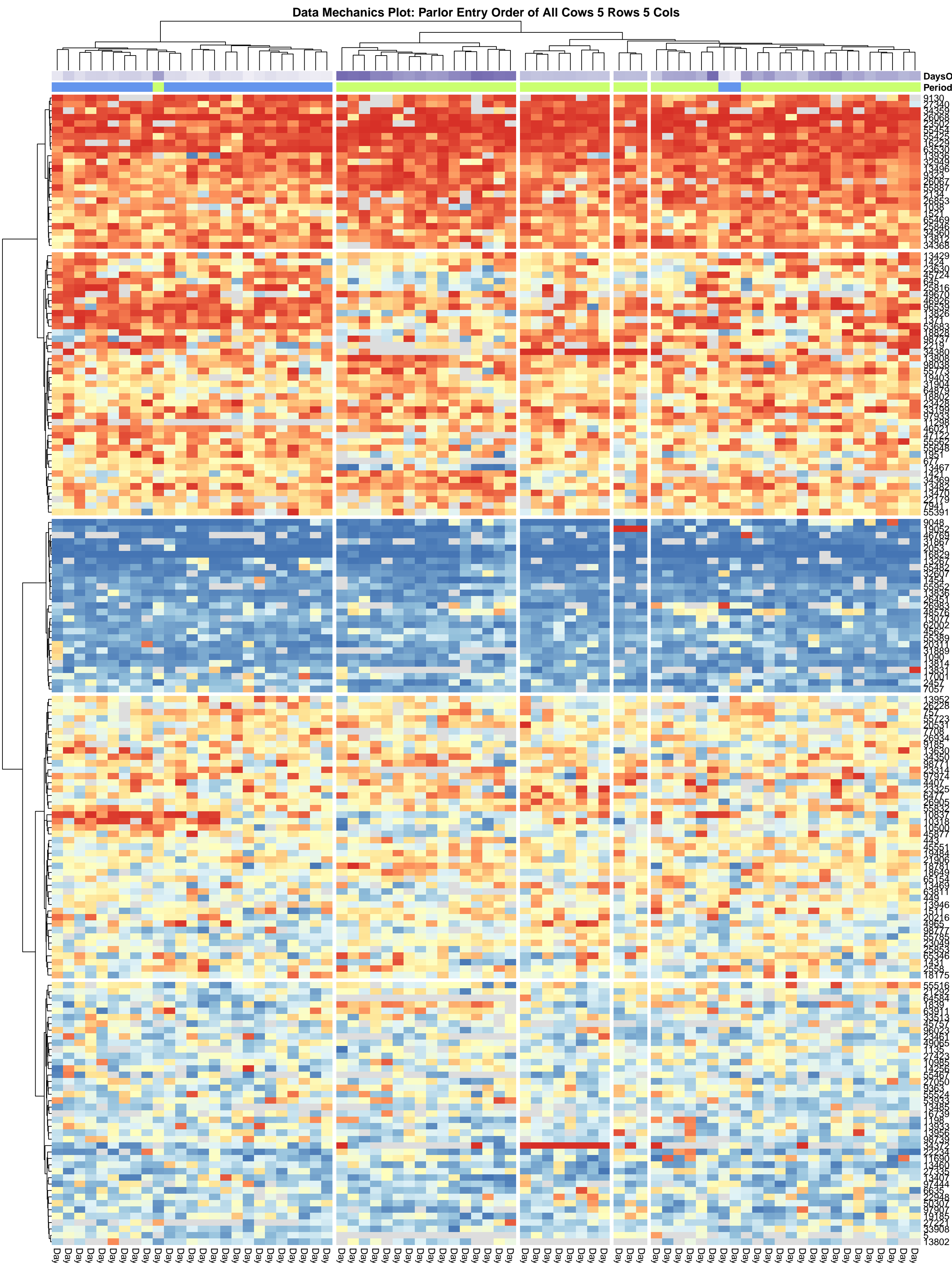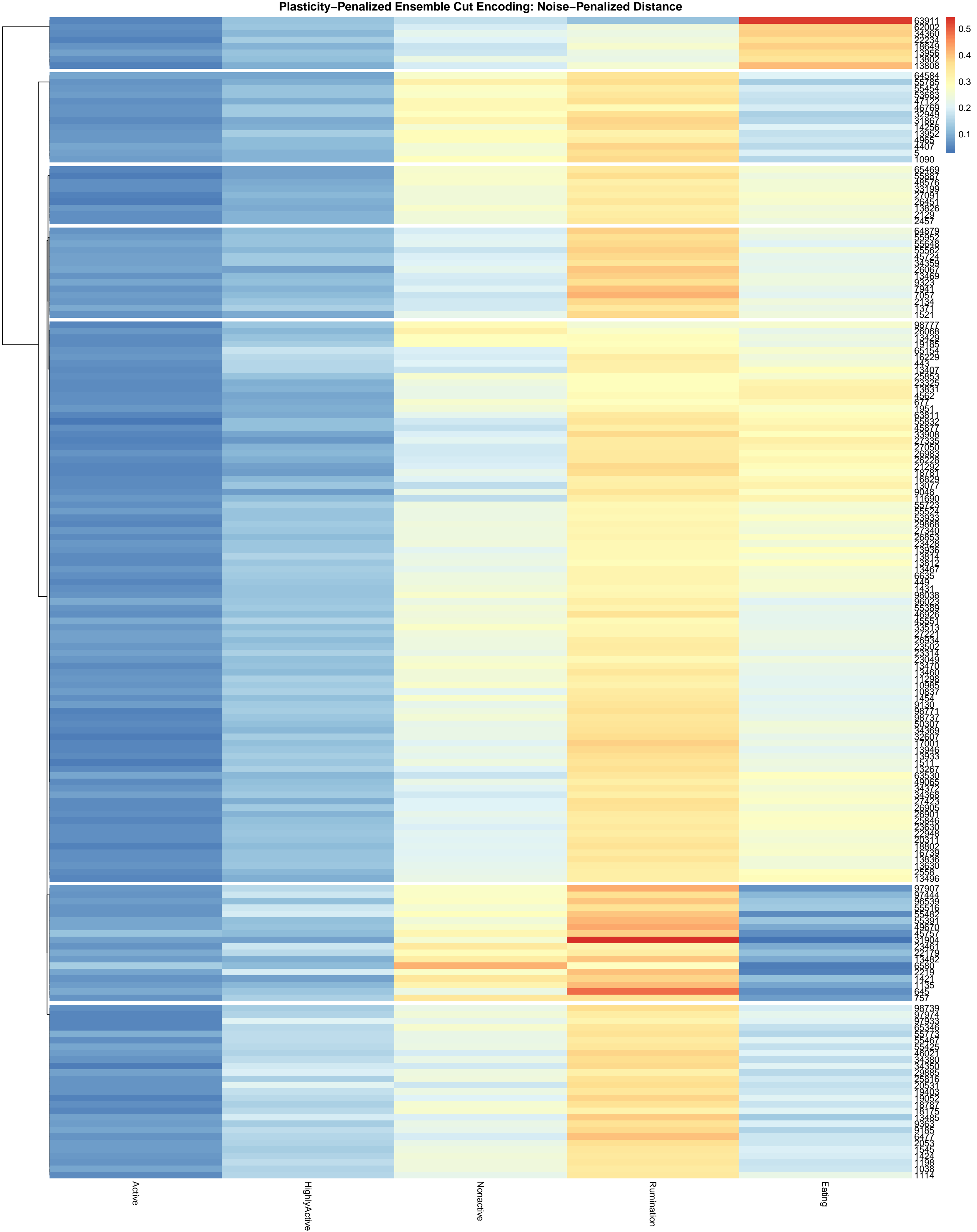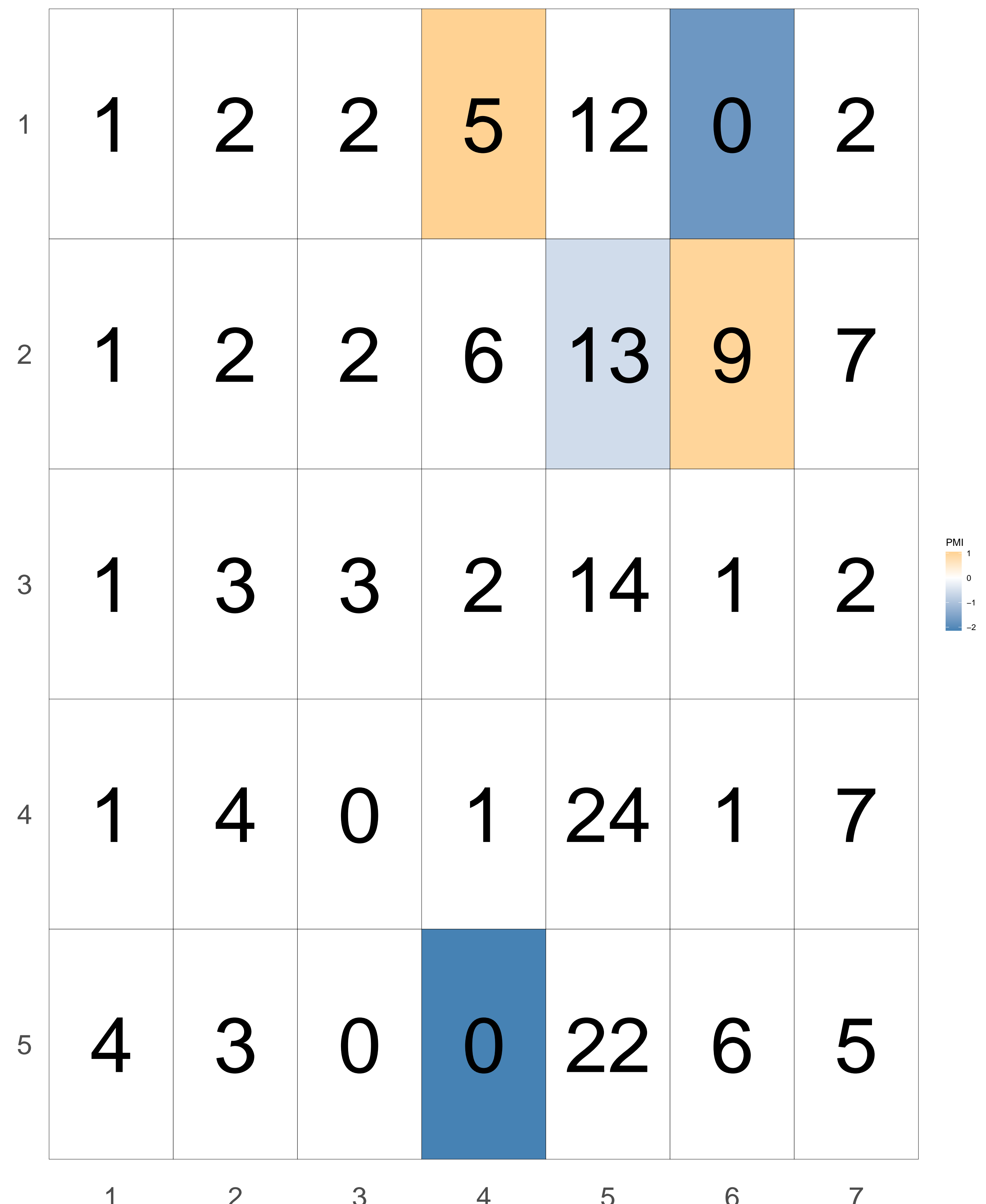

Supplement: Supplementary file 1 [file sensors-22-00001-s001.zip › sensors-1463895-supplementary/OverallTB/BivarTest_EntryOrder/OTB_All_NP.pdf]

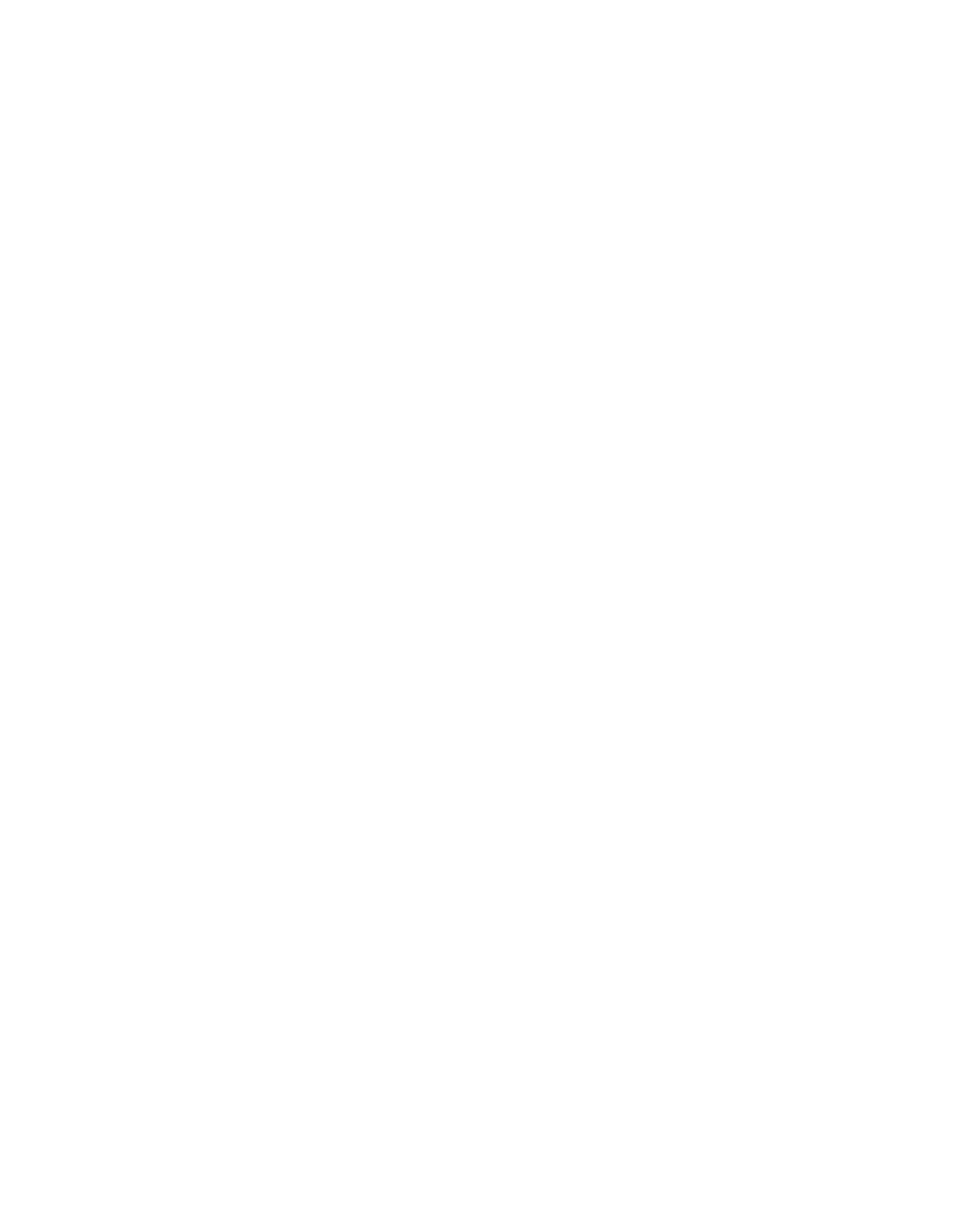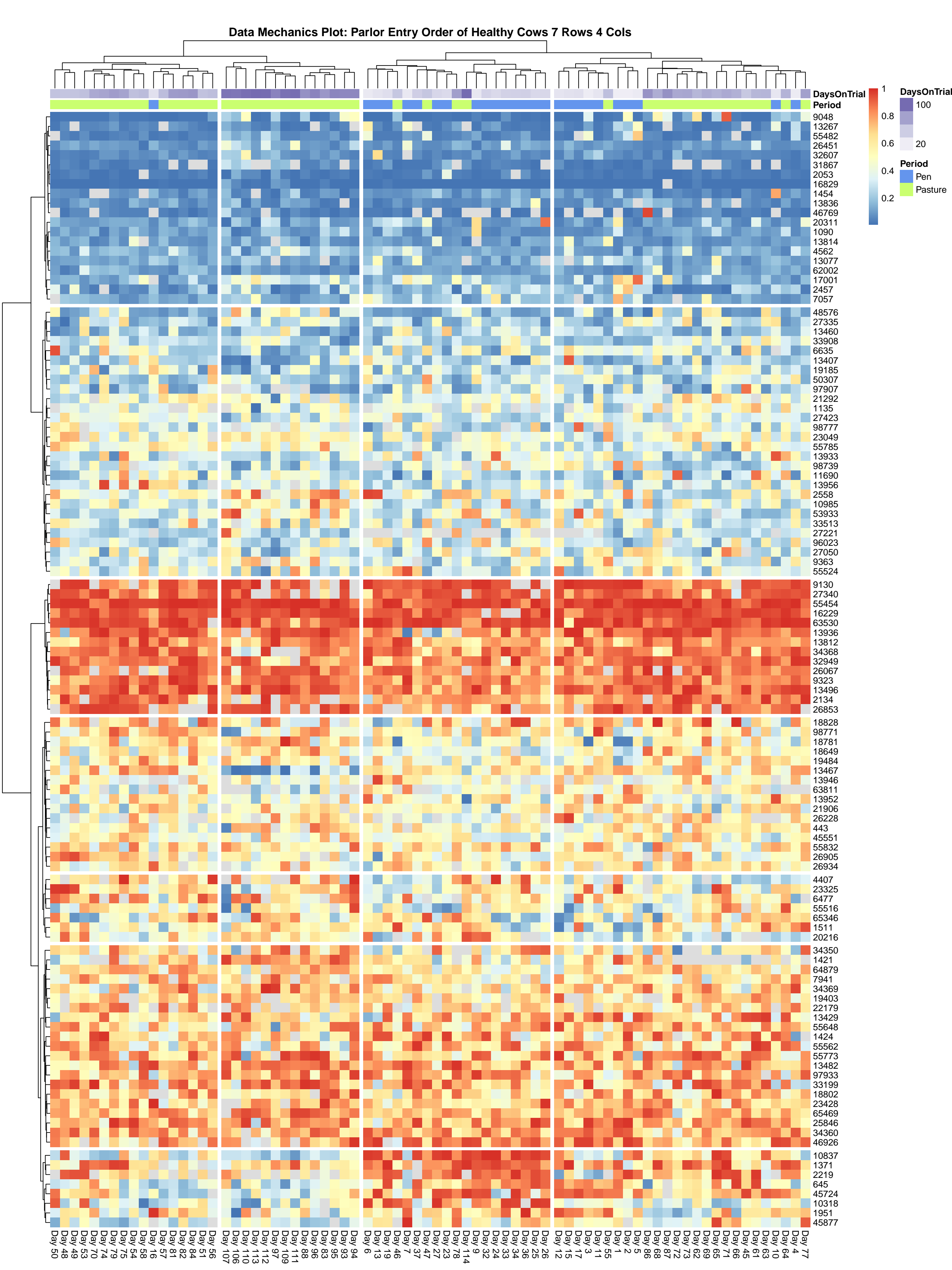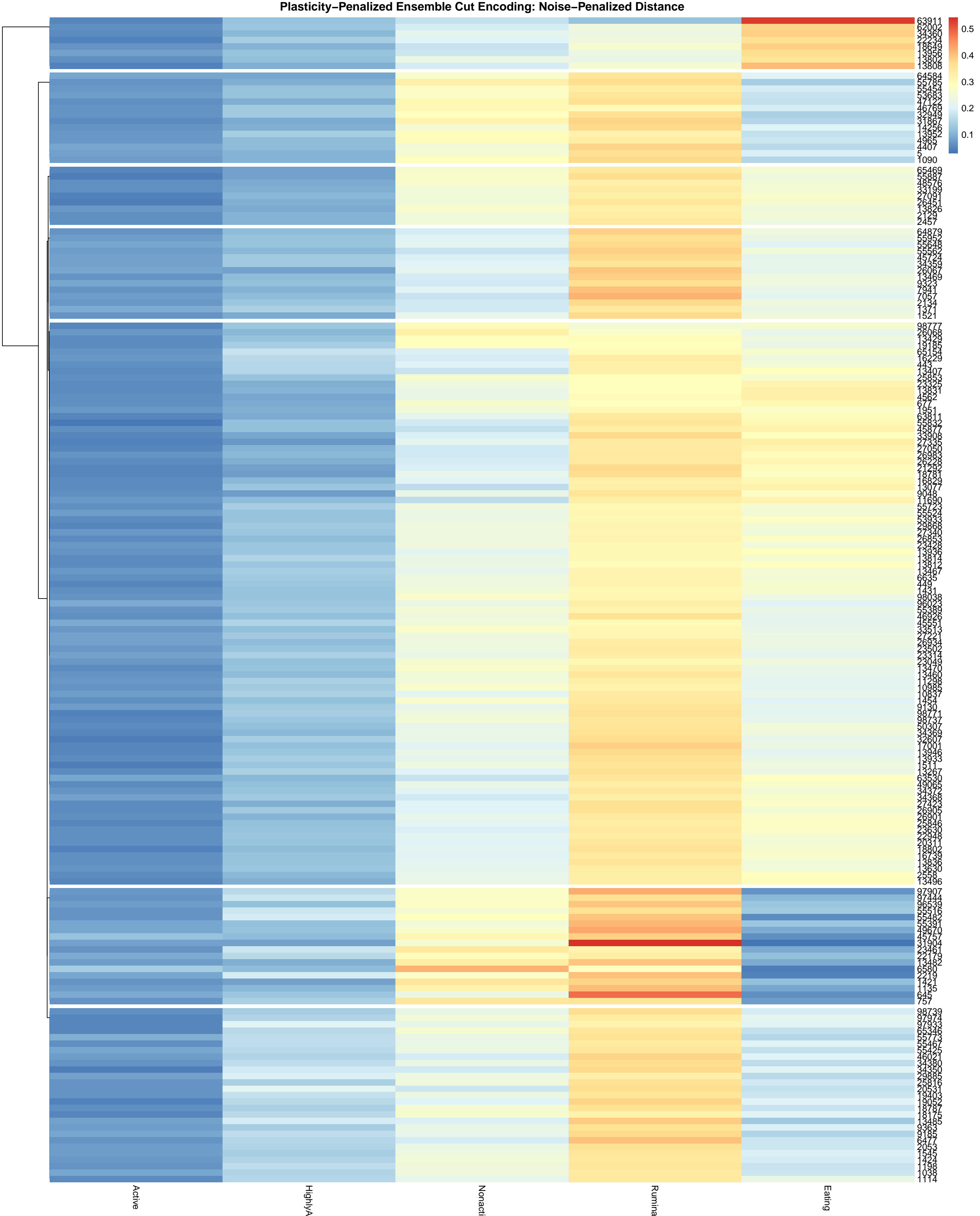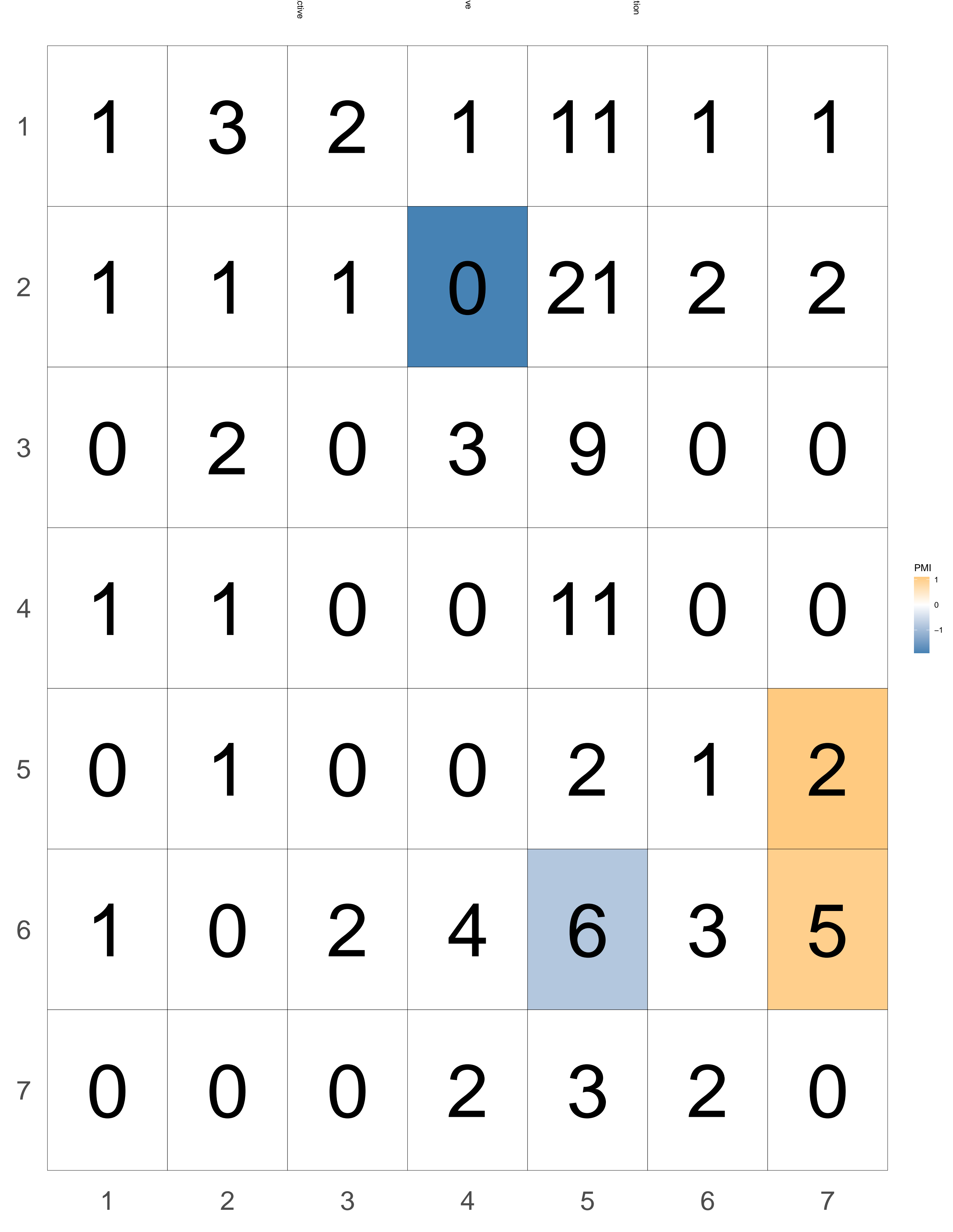

Supplement: Supplementary file 1 [file sensors-22-00001-s001.zip › sensors-1463895-supplementary/OverallTB/BivarTest_EntryOrder/OTB_Healthy_NP.pdf]

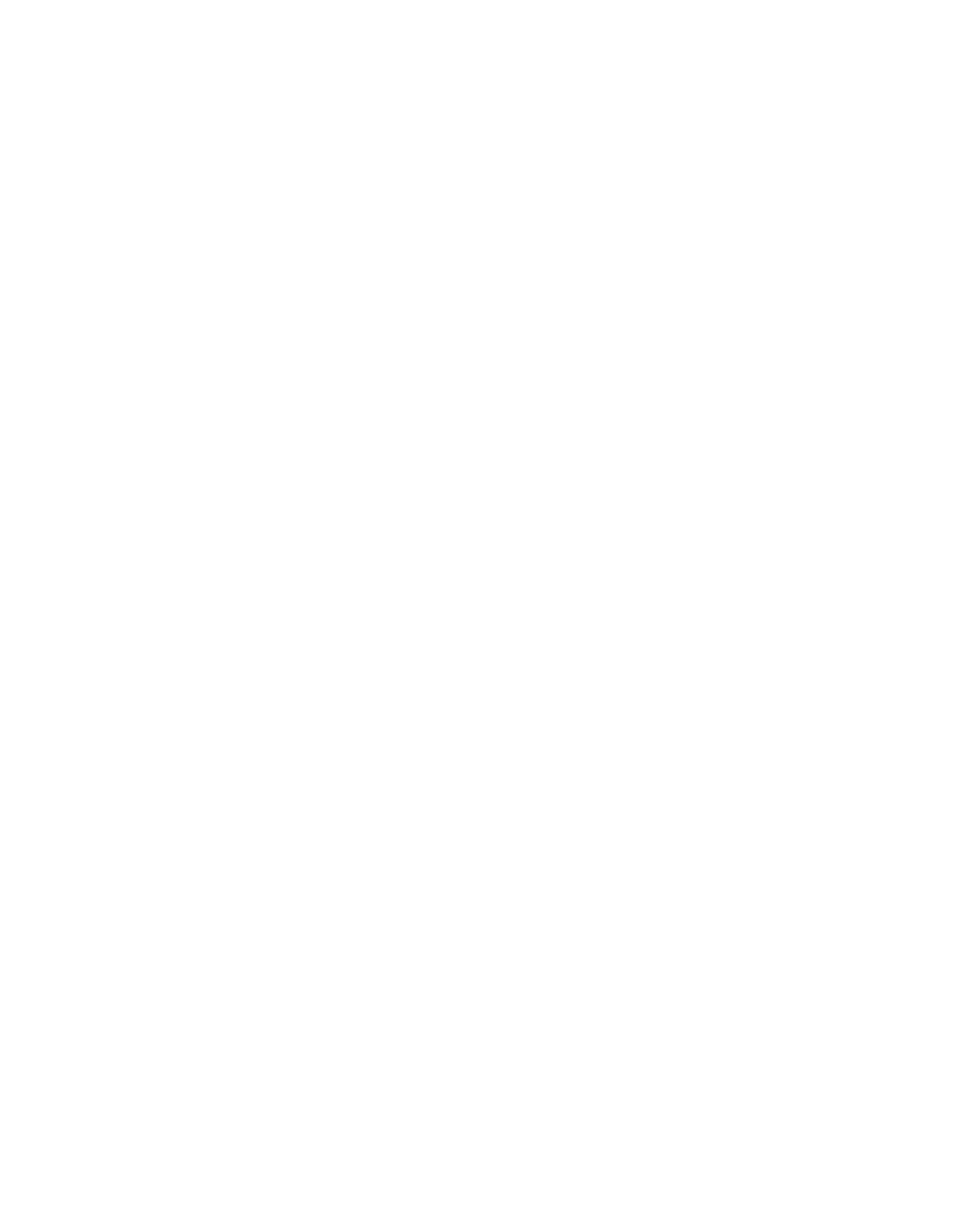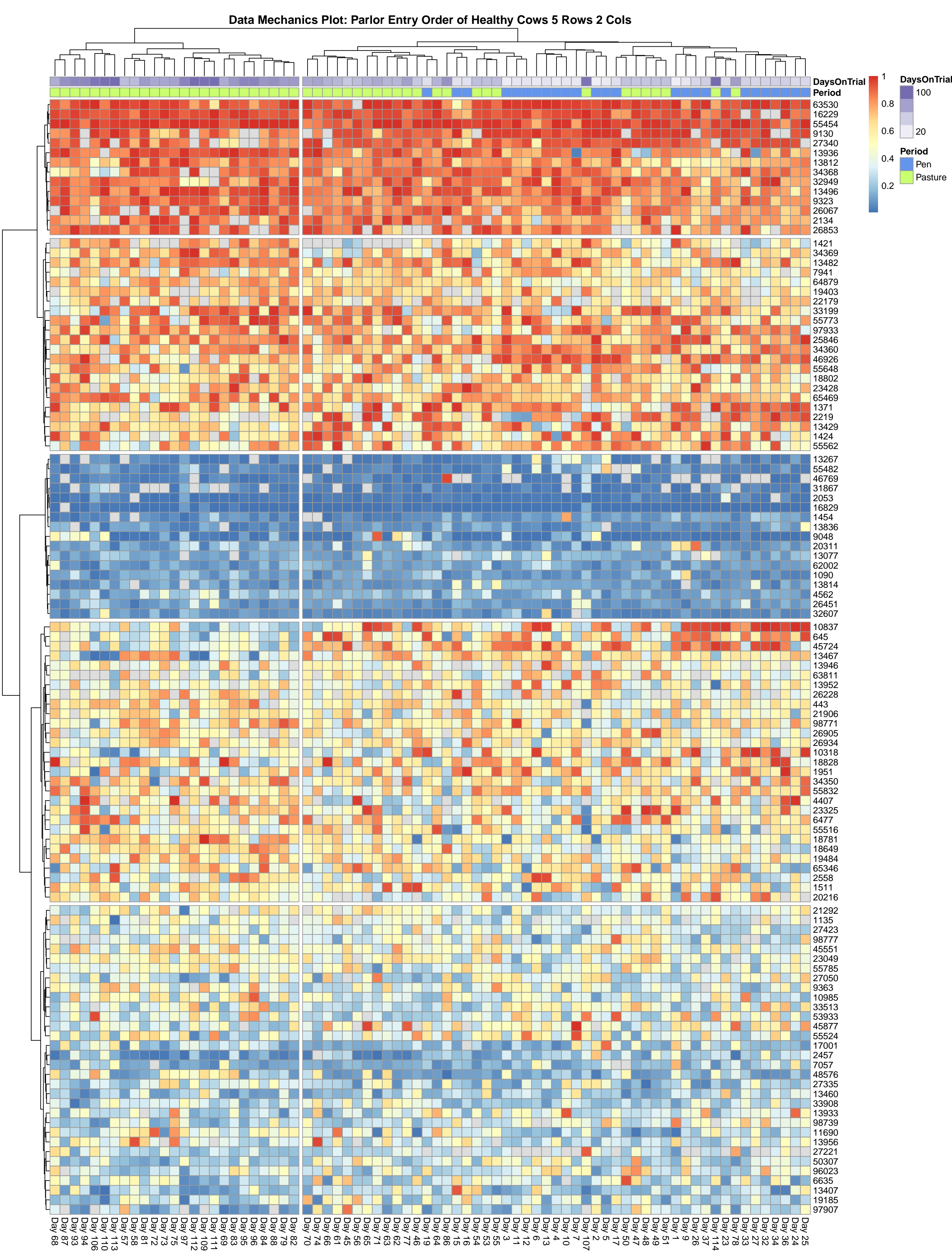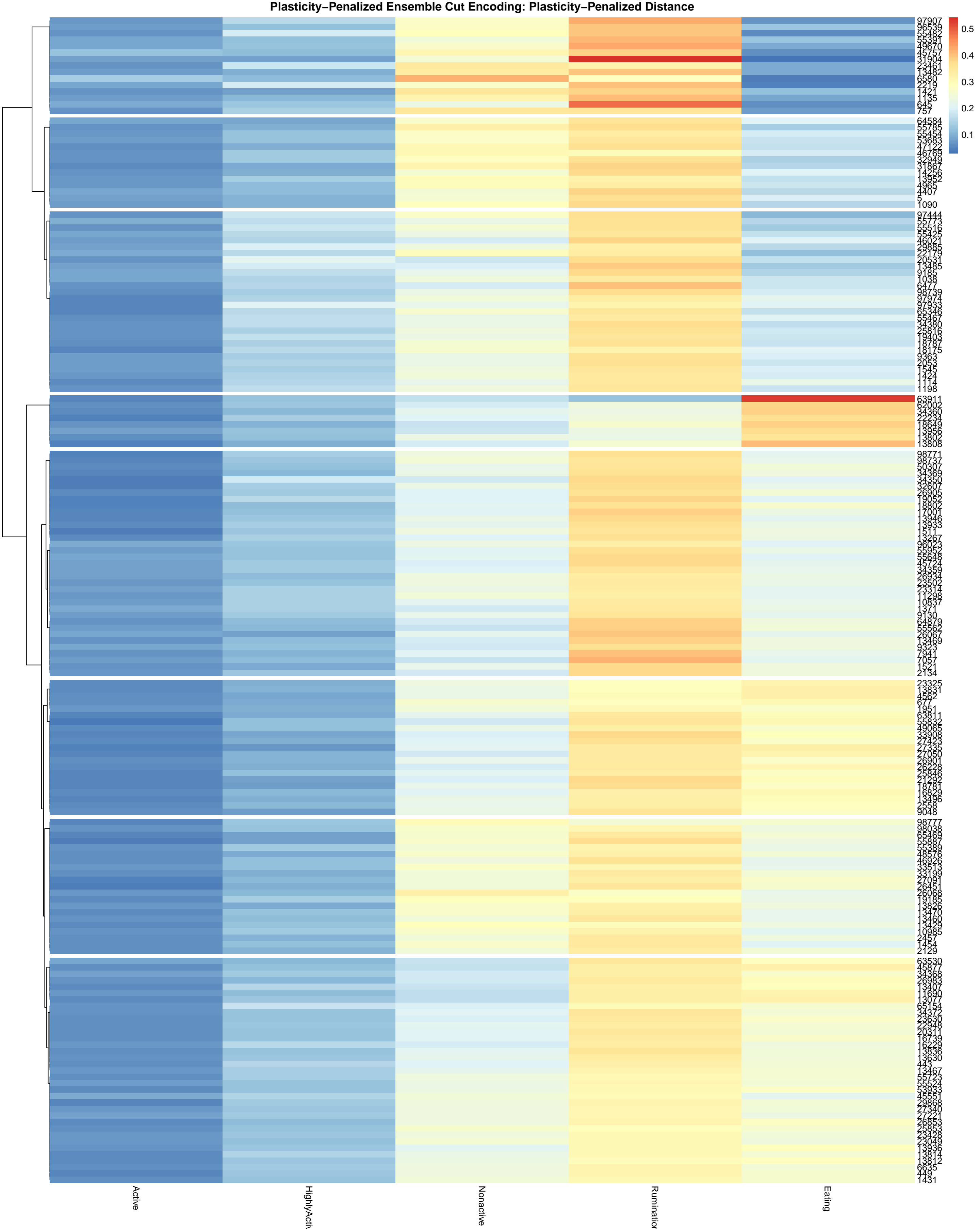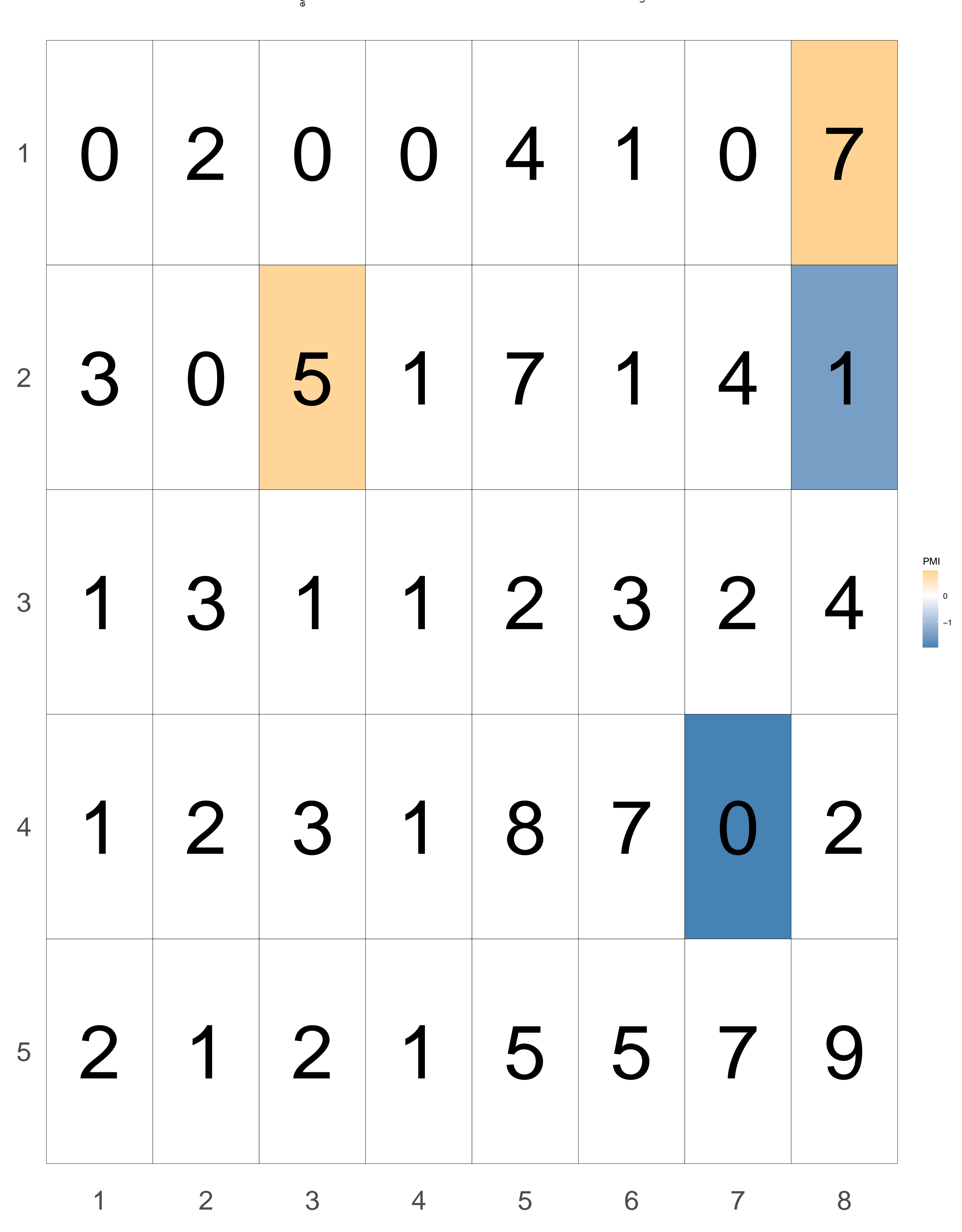

Supplement: Supplementary file 1 [file sensors-22-00001-s001.zip › sensors-1463895-supplementary/OverallTB/BivarTest_EntryOrder/OTB_Healthy_PP.pdf]

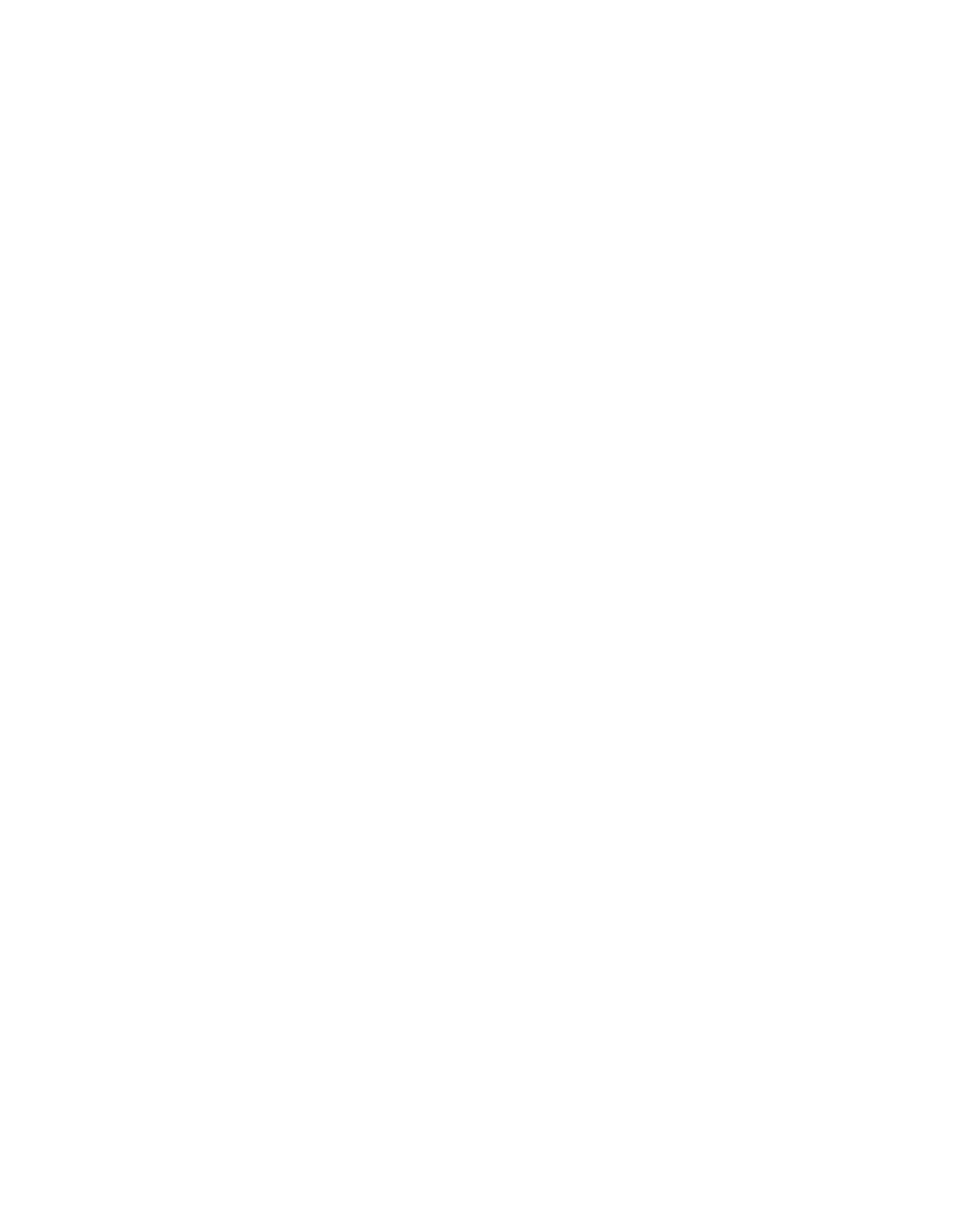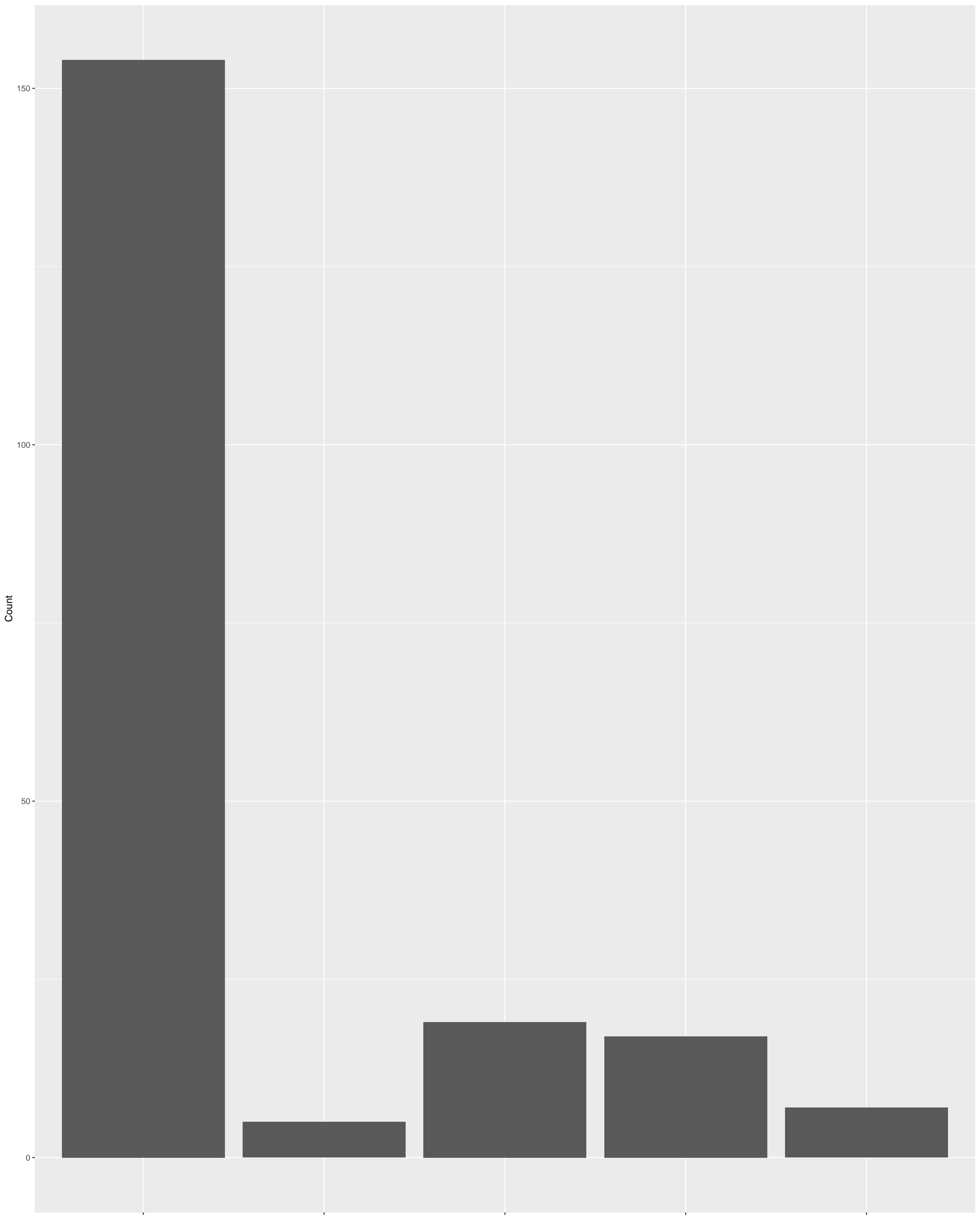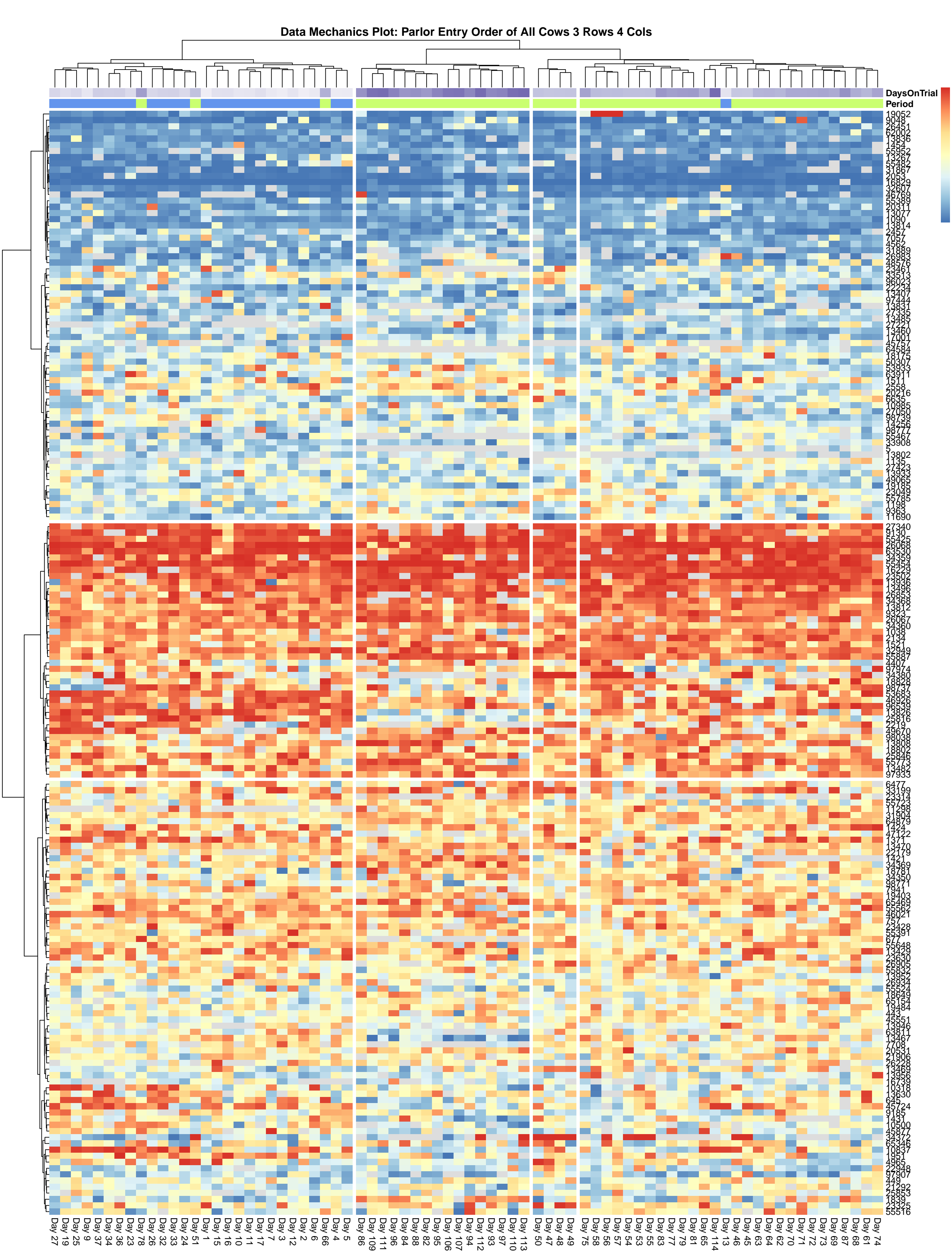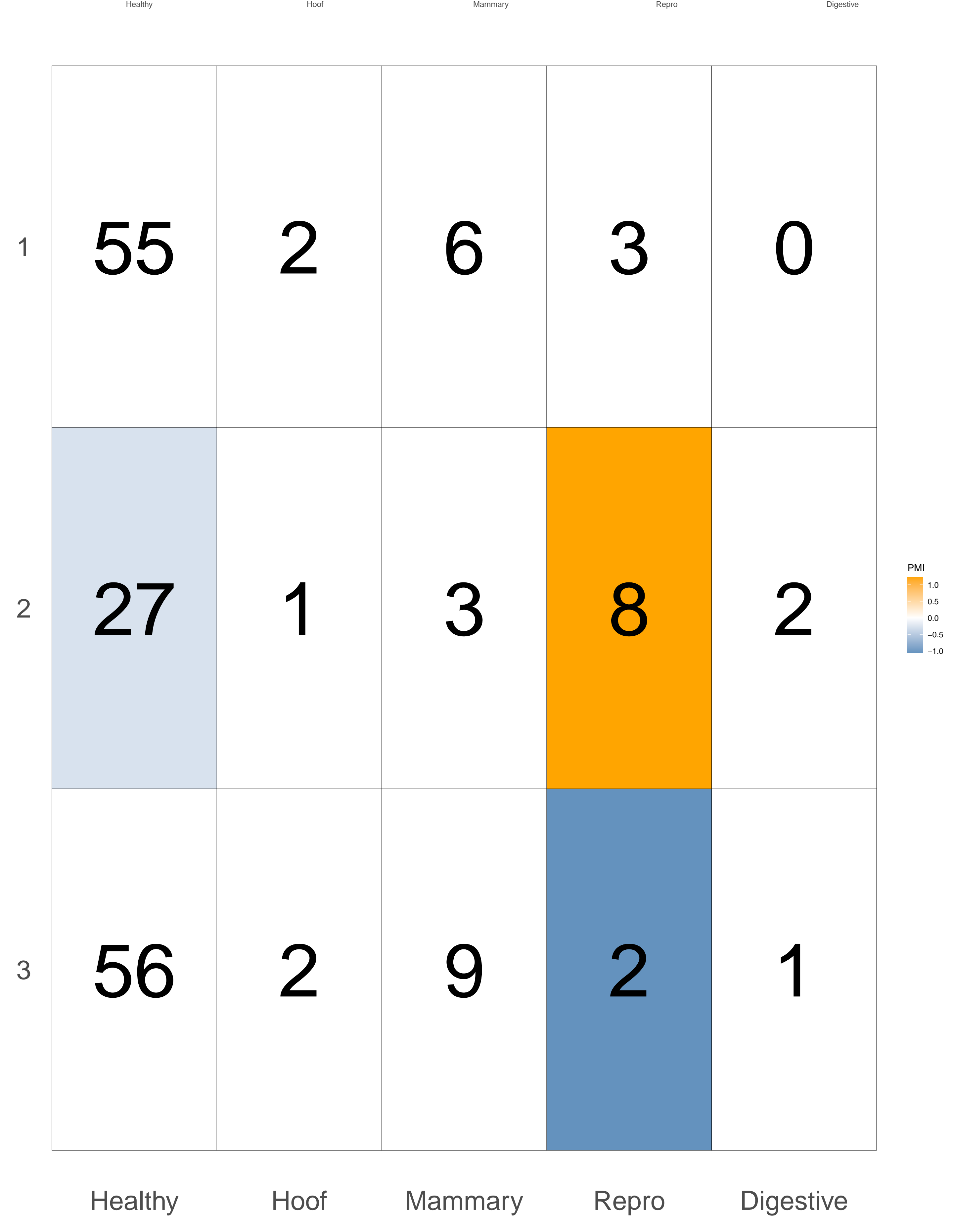

|   | Healthy | Hoof | Mammary | Repro | Digestive |
|---|---------|------|---------|-------|-----------|
| 1 | 55      | 2    | 6       | 3     | 0         |
| 2 | 27      | 1    | 3       | 8     | 2         |
| 3 | 56      | 2    | 9       | 2     | 1         |

Supplement: Supplementary file 1 [file sensors-22-00001-s001.zip › sensors-1463895-supplementary/OverallTB/BivarTest_Sick/EO_Diag.pdf]

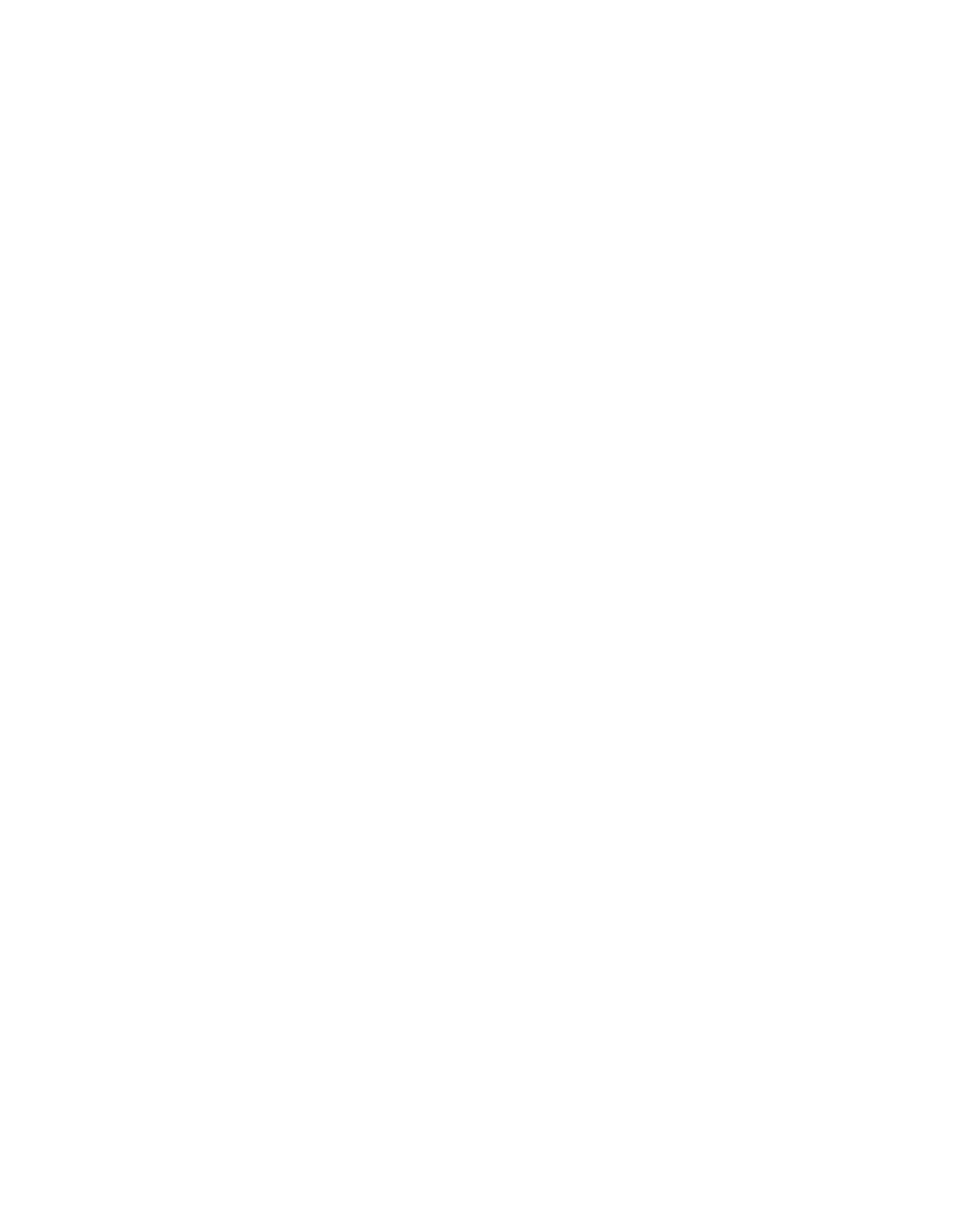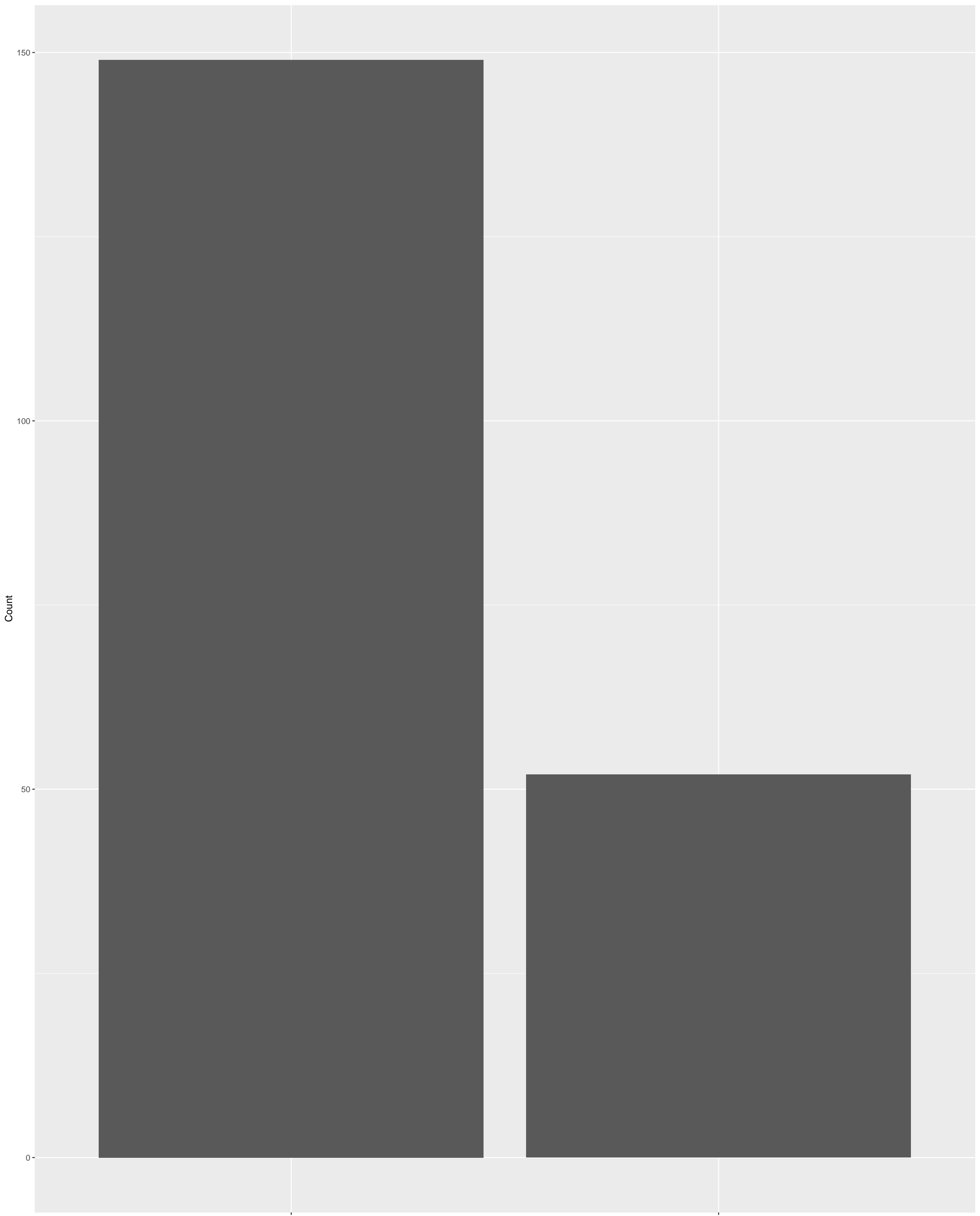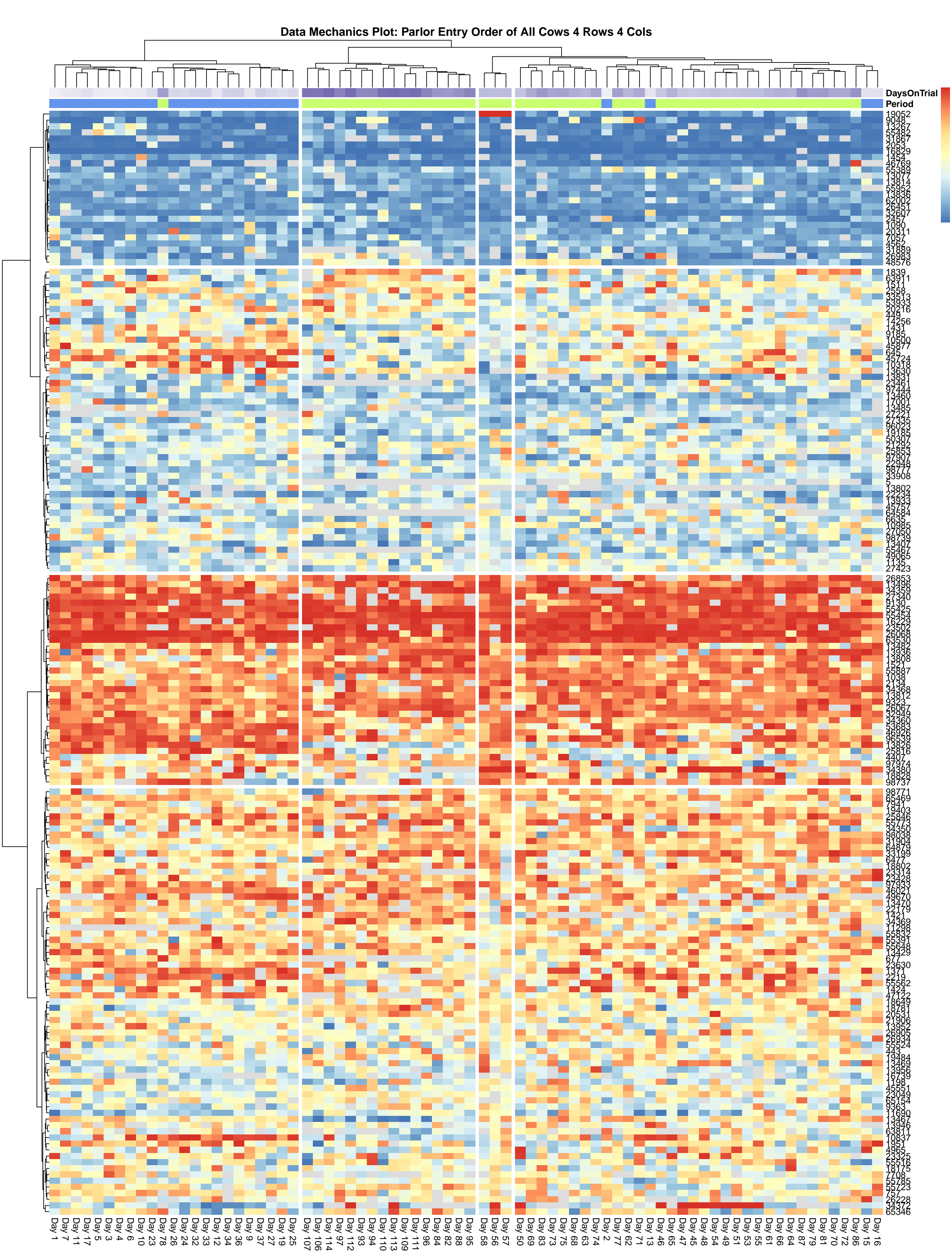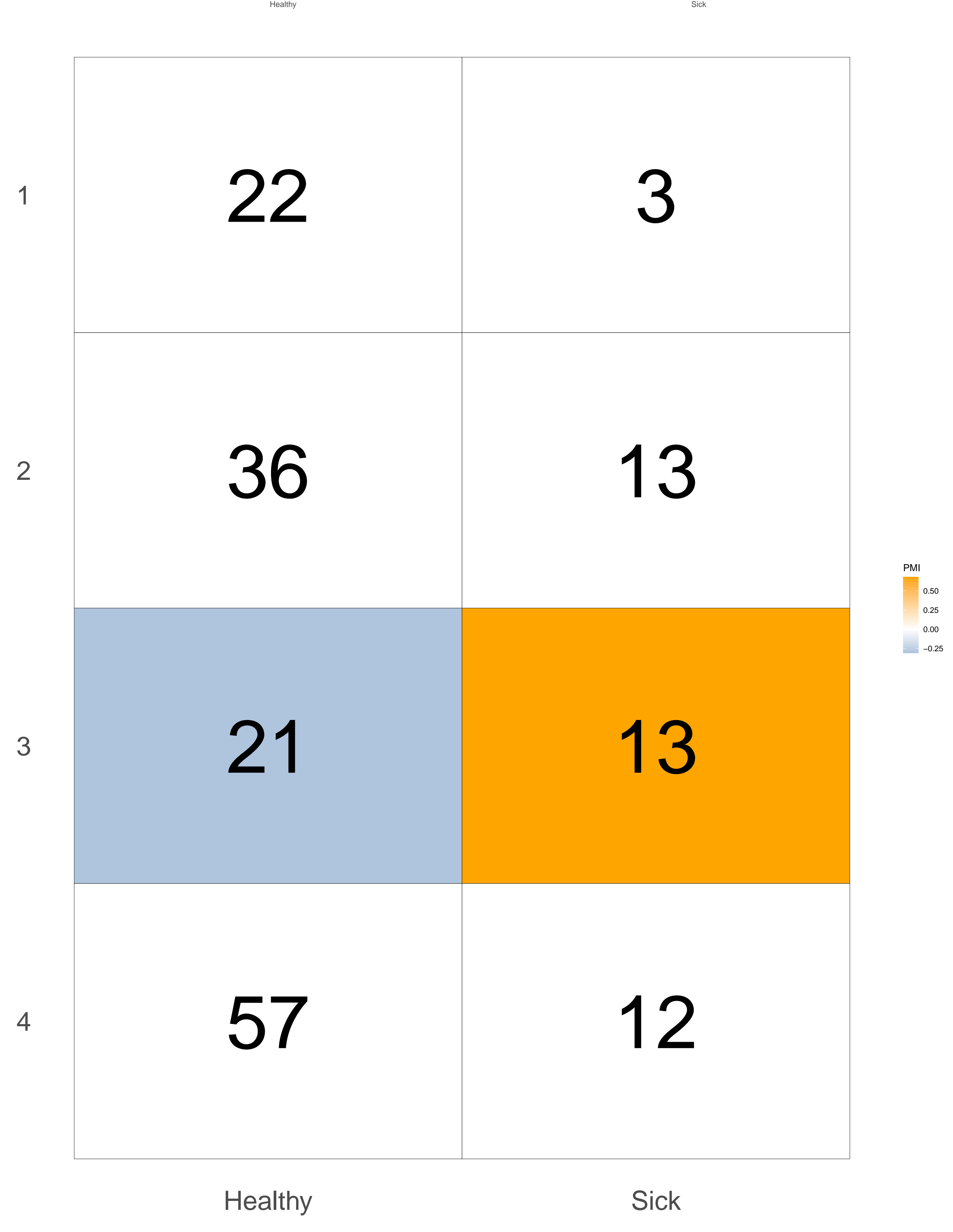

Supplement: Supplementary file 1 [file sensors-22-00001-s001.zip › sensors-1463895-supplementary/OverallTB/BivarTest_Sick/EO_Health.pdf]

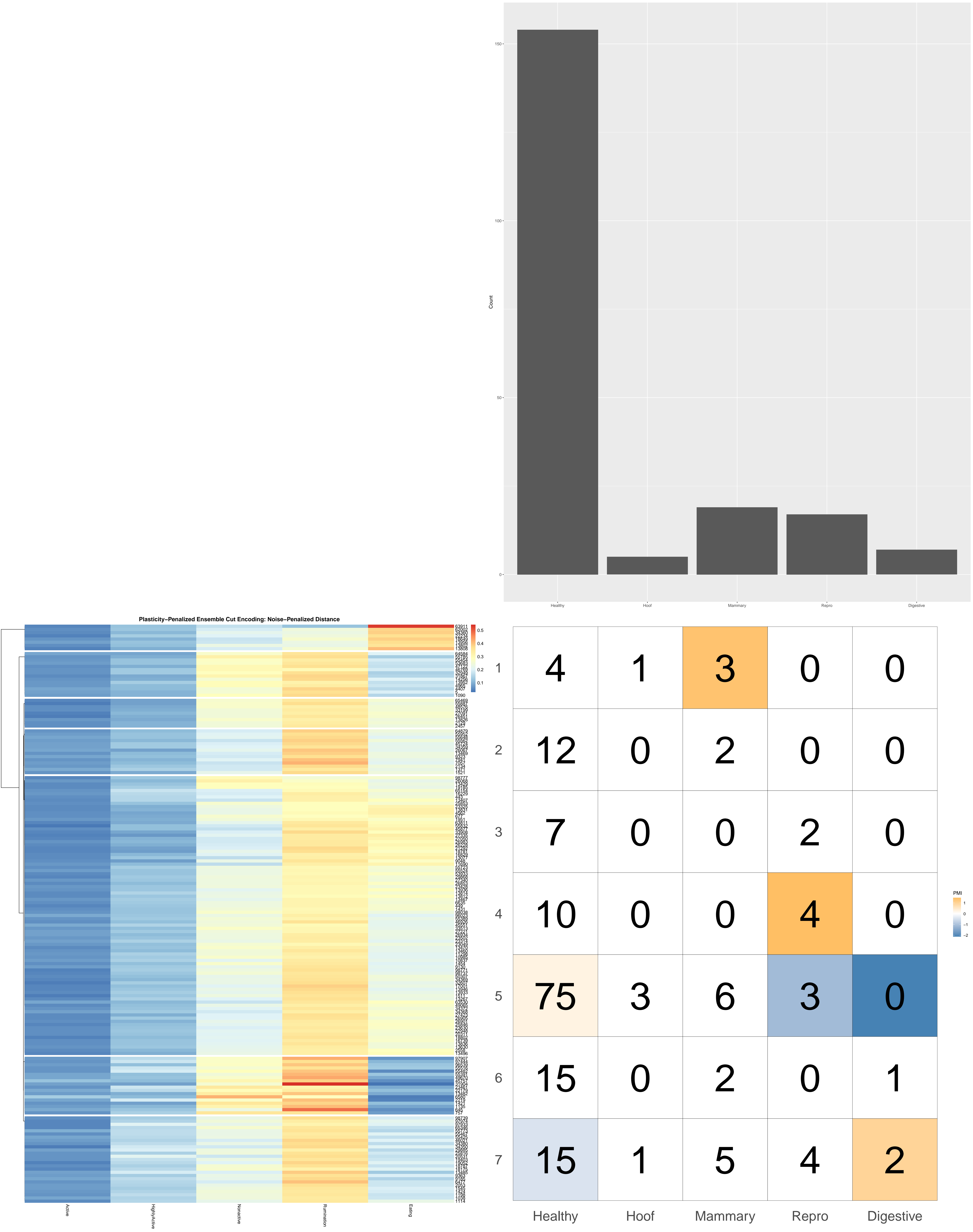

Supplement: Supplementary file 1 [file sensors-22-00001-s001.zip › sensors-1463895-supplementary/OverallTB/BivarTest_Sick/OTB_Diag_NP.pdf]

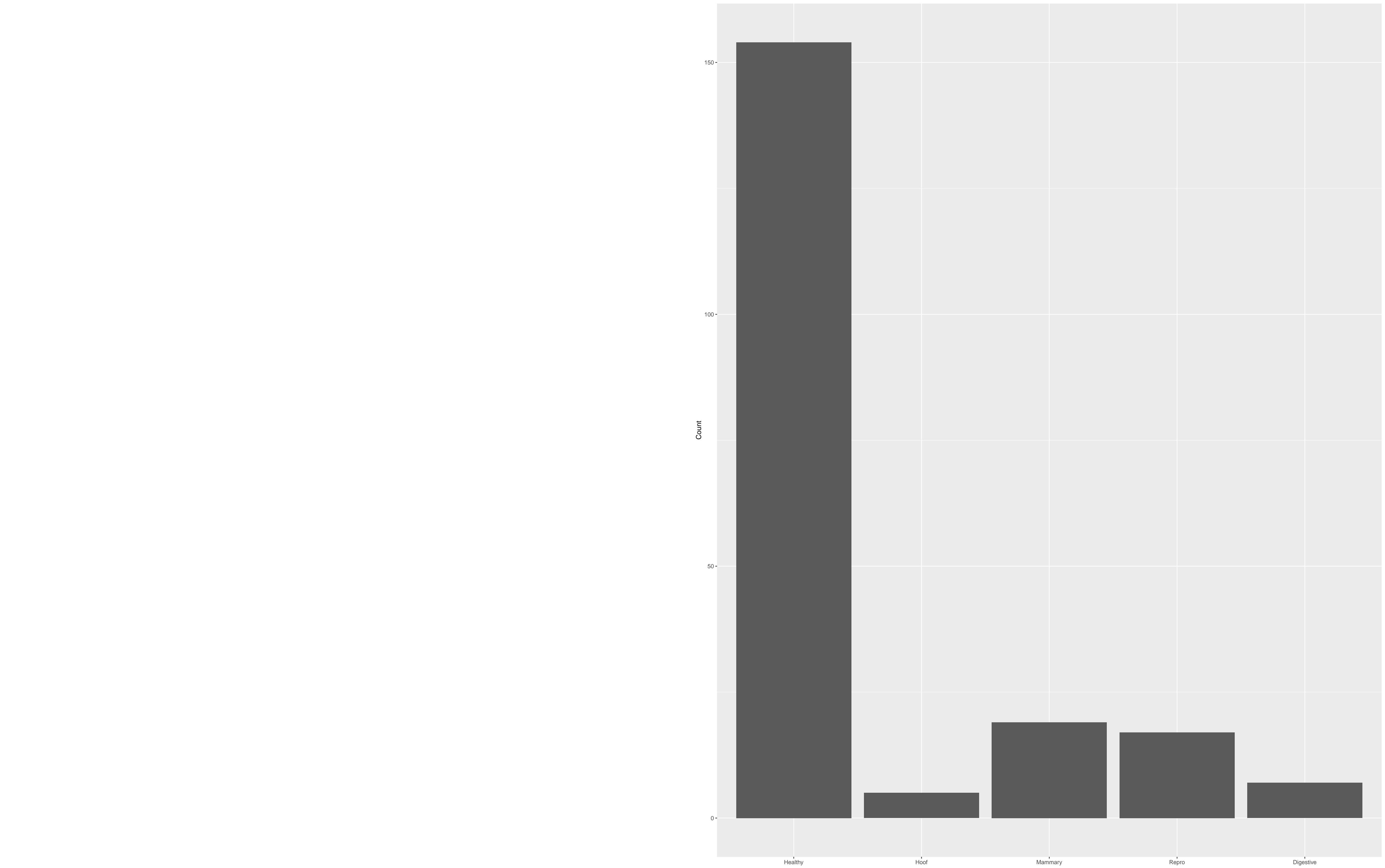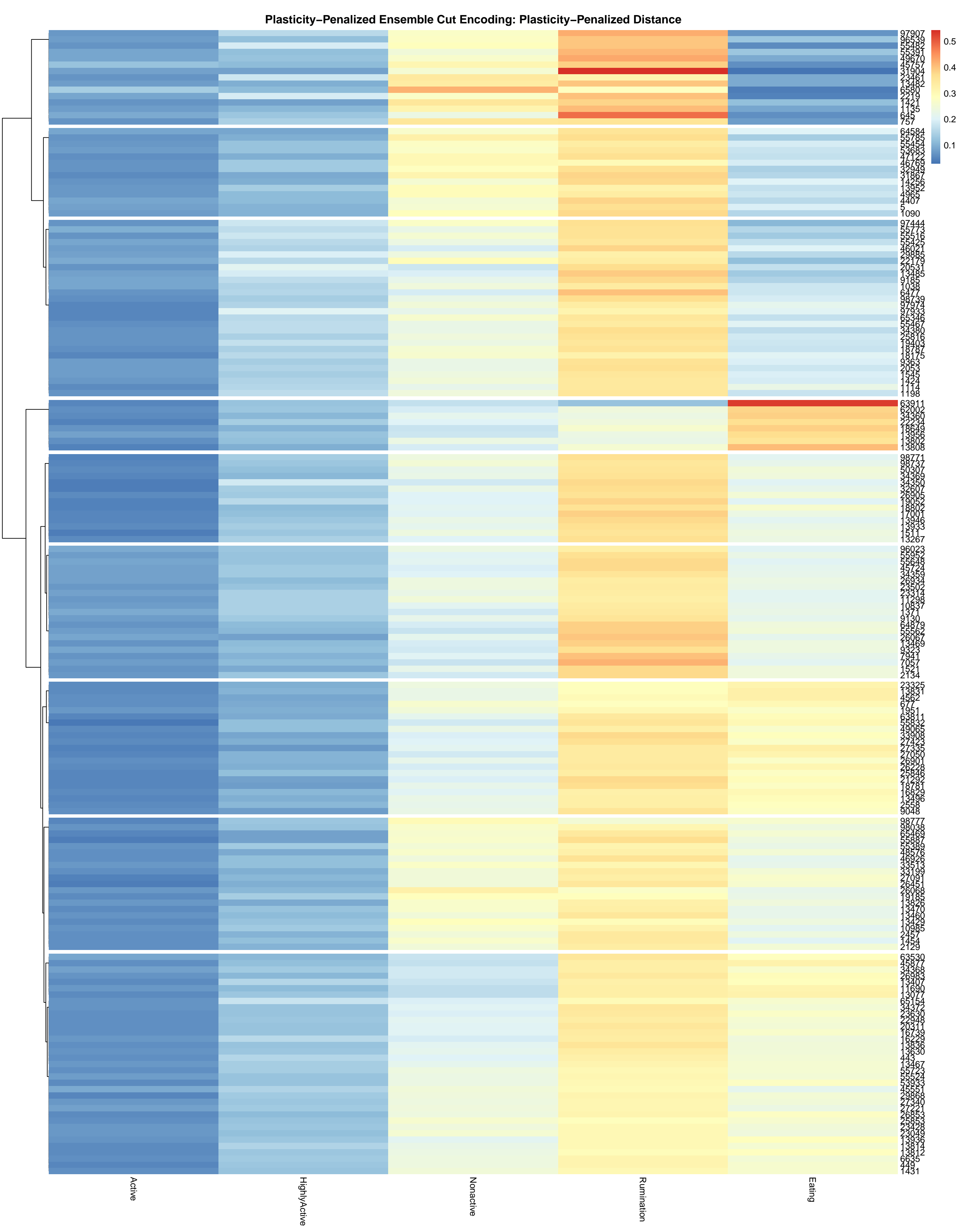

|   | Healthy | Hoof | Mammary | Repro | Digestive |
|---|---------|------|---------|-------|-----------|
| 1 | 12      | 0    | 2       | 0     | 1         |
| 2 | 12      | 0    | 2       | 0     | 0         |
| 3 | 16      | 1    | 5       | 4     | 2         |
| 4 | 4       | 1    | 3       | 0     | 0         |
| 5 | 14      | 0    | 0       | 0     | 0         |
| 6 | 16      | 1    | 0       | 4     | 0         |
| 7 | 19      | 0    | 1       | 1     | 0         |
| 8 | 16      | 0    | 1       | 4     | 0         |
| 9 | 29      | 2    | 4       | 0     | 0         |

Supplement: Supplementary file 1 [file sensors-22-00001-s001.zip › sensors-1463895-supplementary/OverallTB/BivarTest_Sick/OTB_Diag_PP.pdf]

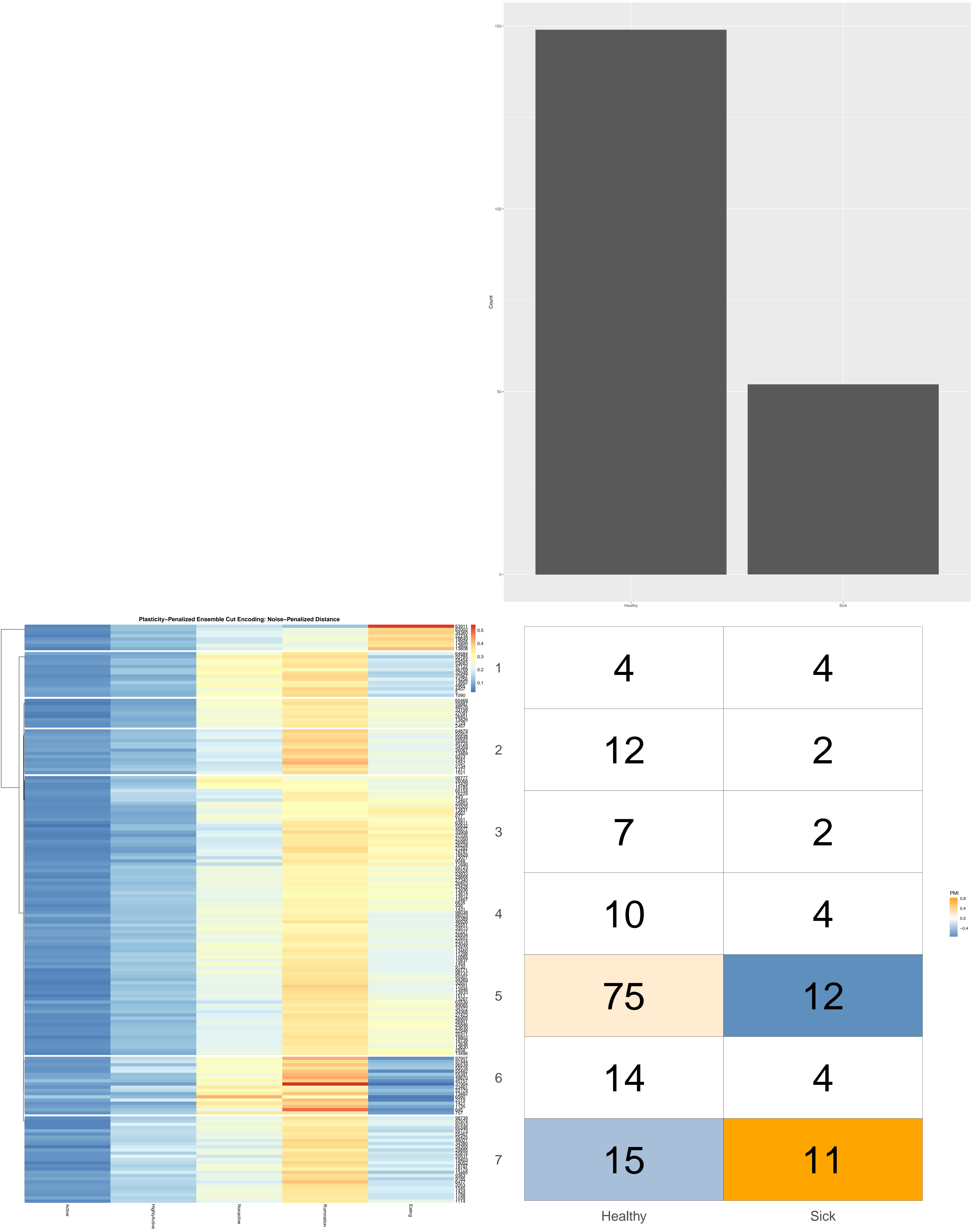

Supplement: Supplementary file 1 [file sensors-22-00001-s001.zip › sensors-1463895-supplementary/OverallTB/BivarTest_Sick/OTB_Health_NP.pdf]

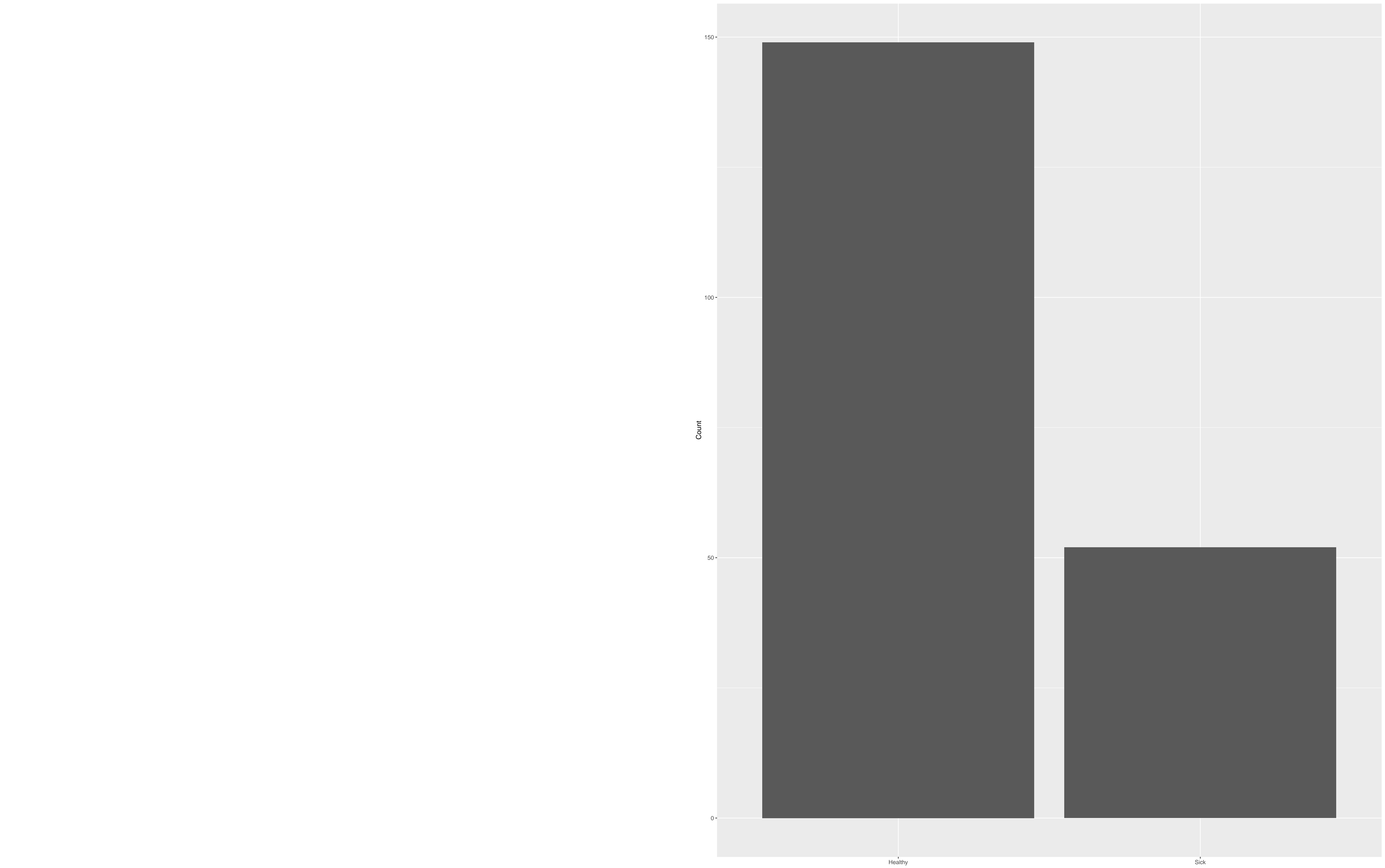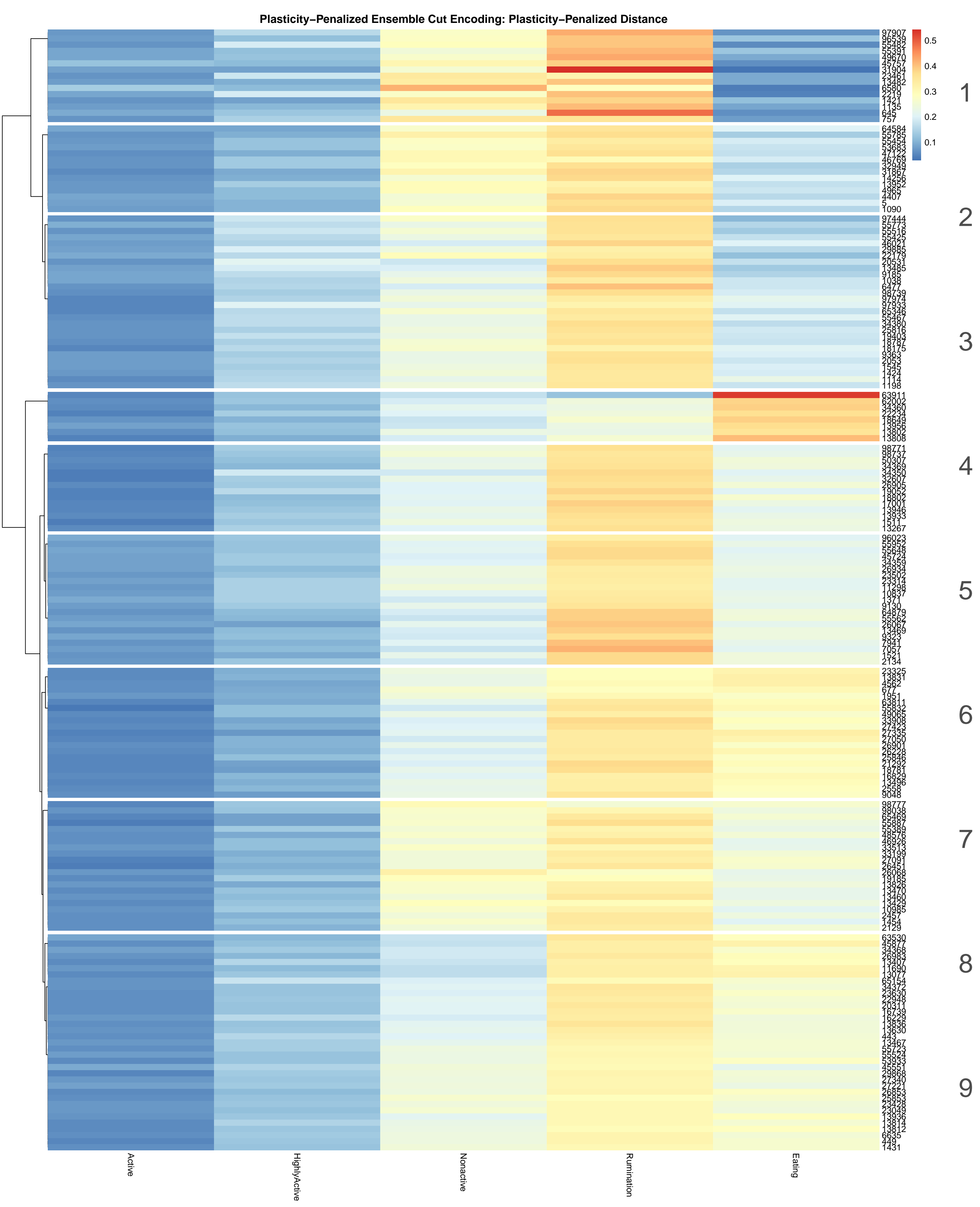

|         |      |
|---------|------|
| 12      | 3    |
| 12      | 2    |
| 15      | 12   |
| 4       | 4    |
| 14      | 0    |
| 16      | 5    |
| 19      | 2    |
| 16      | 5    |
| 29      | 6    |
| Healthy | Sick |

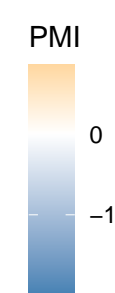

Supplement: Supplementary file 1 [file sensors-22-00001-s001.zip › sensors-1463895-supplementary/OverallTB/BivarTest_Sick/OTB_Health_PP.pdf]

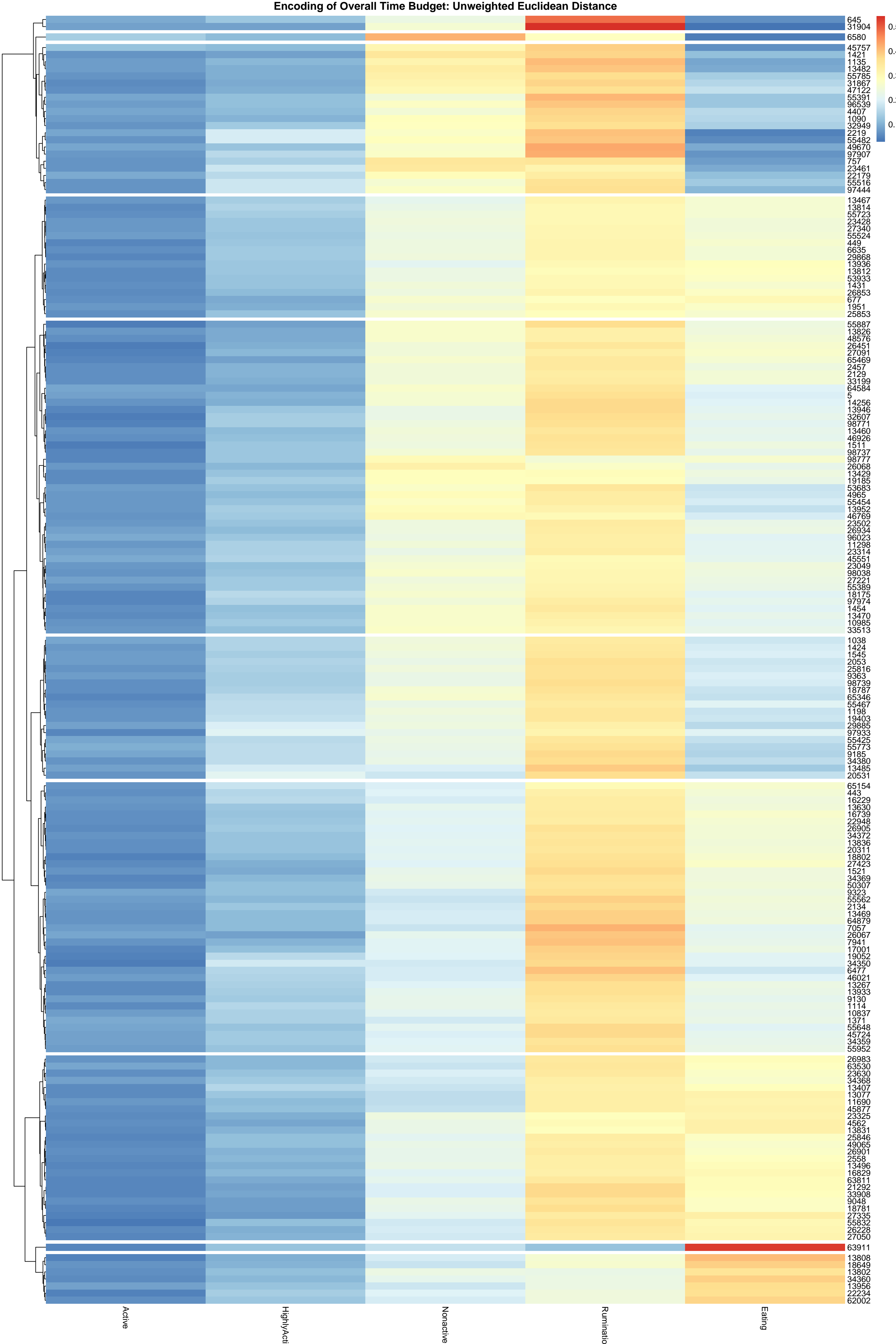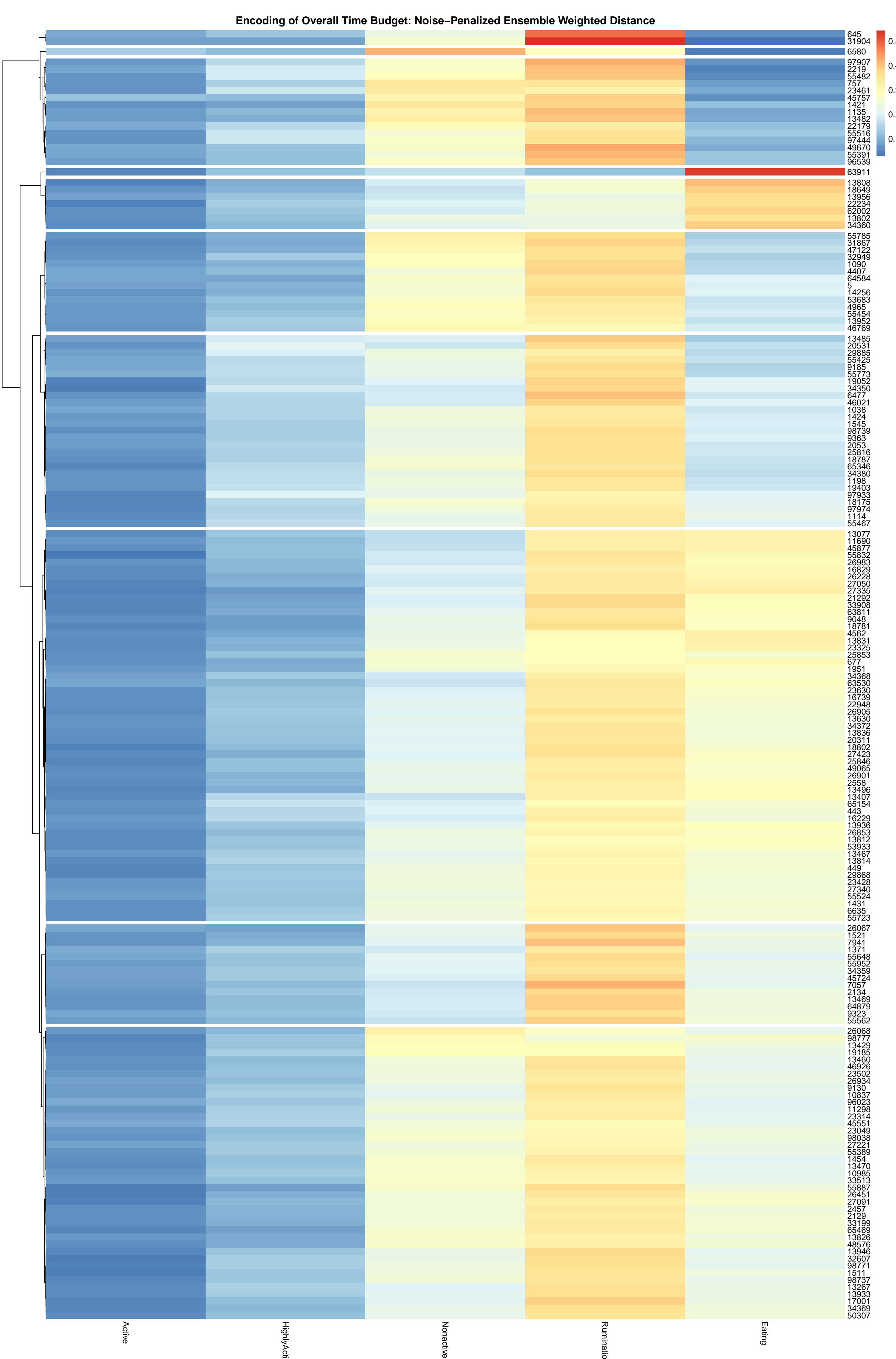

|    | 1 | 2 | 3  | 4  | 5  | 6  | 7  | 8  | 9 | 10 |
|----|---|---|----|----|----|----|----|----|---|----|
| 1  | 2 | 0 | 0  | 0  | 0  | 0  | 0  | 0  | 0 | 0  |
| 2  | 0 | 1 | 0  | 0  | 0  | 0  | 0  | 0  | 0 | 0  |
| 3  | 0 | 0 | 15 | 0  | 0  | 0  | 0  | 0  | 0 | 0  |
| 4  | 0 | 0 | 0  | 0  | 0  | 0  | 0  | 0  | 1 | 0  |
| 5  | 0 | 0 | 0  | 0  | 0  | 0  | 0  | 0  | 0 | 7  |
| 6  | 0 | 0 | 6  | 0  | 8  | 0  | 0  | 0  | 0 | 0  |
| 7  | 0 | 0 | 0  | 0  | 2  | 20 | 5  | 0  | 0 | 0  |
| 8  | 0 | 0 | 0  | 17 | 0  | 0  | 12 | 26 | 0 | 0  |
| 9  | 0 | 0 | 0  | 0  | 0  | 0  | 14 | 0  | 0 | 0  |
| 10 | 0 | 0 | 0  | 0  | 34 | 0  | 7  | 0  | 0 | 0  |

Supplement: Supplementary file 1 [file sensors-22-00001-s001.zip › sensors-1463895-supplementary/OverallTB/ContrastPlots/Euclid_vs_EW_10x10.pdf]

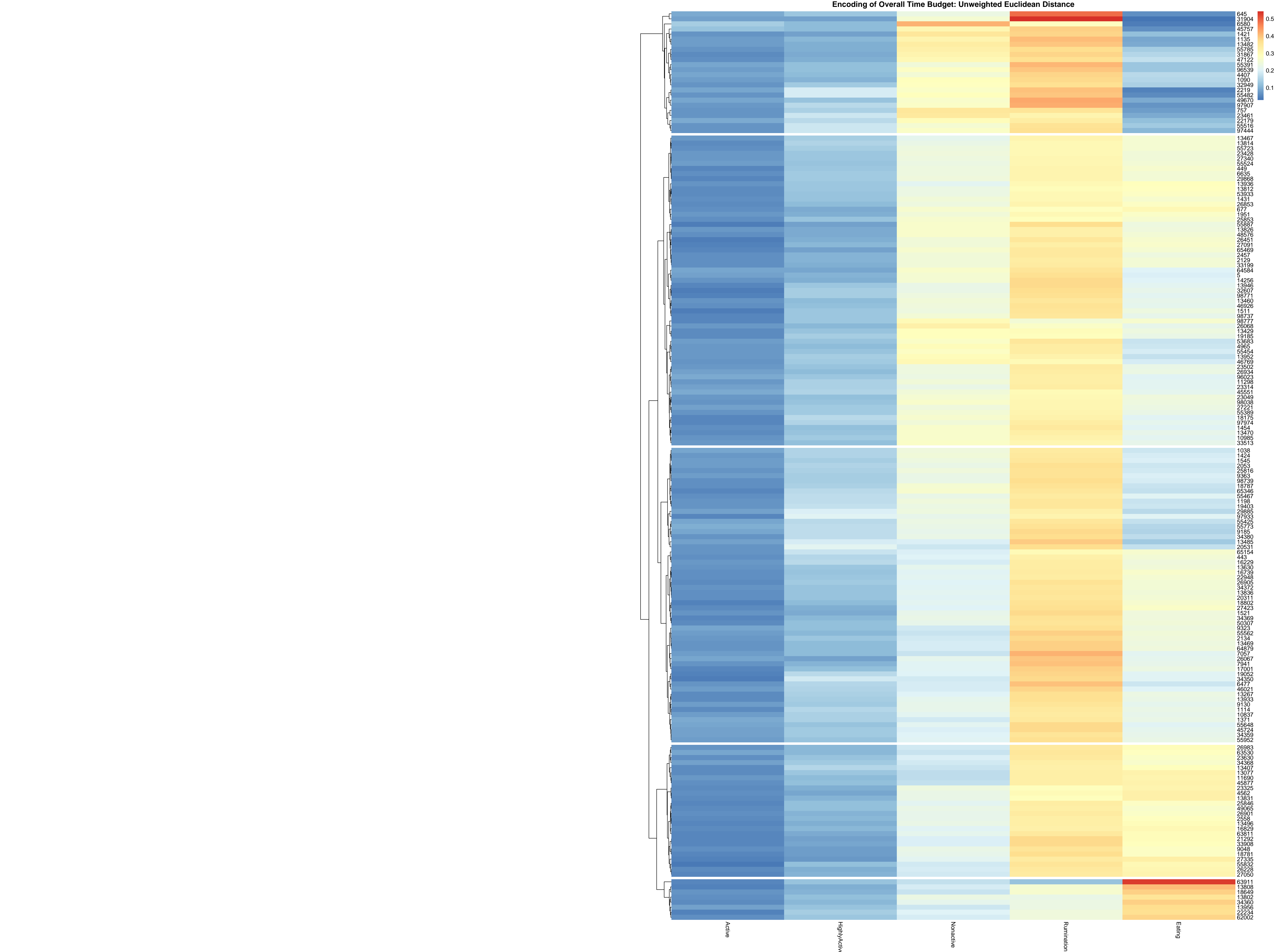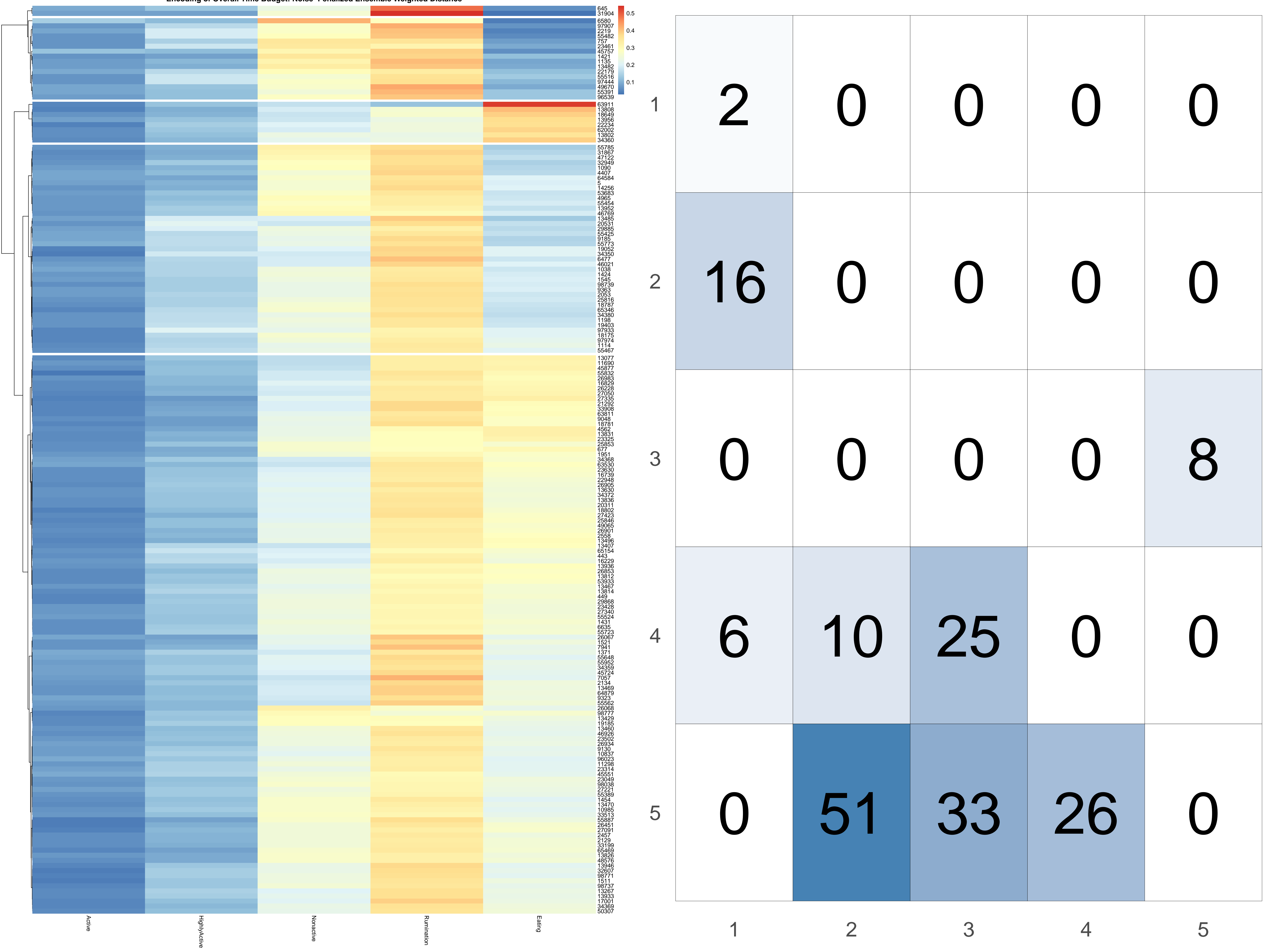

|   |    |    |    |    |   |
|---|----|----|----|----|---|
| 1 | 2  | 0  | 0  | 0  | 0 |
| 2 | 16 | 0  | 0  | 0  | 0 |
| 3 | 0  | 0  | 0  | 0  | 8 |
| 4 | 6  | 10 | 25 | 0  | 0 |
| 5 | 0  | 51 | 33 | 26 | 0 |
|   | 1  | 2  | 3  | 4  | 5 |

Supplement: Supplementary file 1 [file sensors-22-00001-s001.zip › sensors-1463895-supplementary/OverallTB/ContrastPlots/Euclid_vs_EW_5x5.pdf]

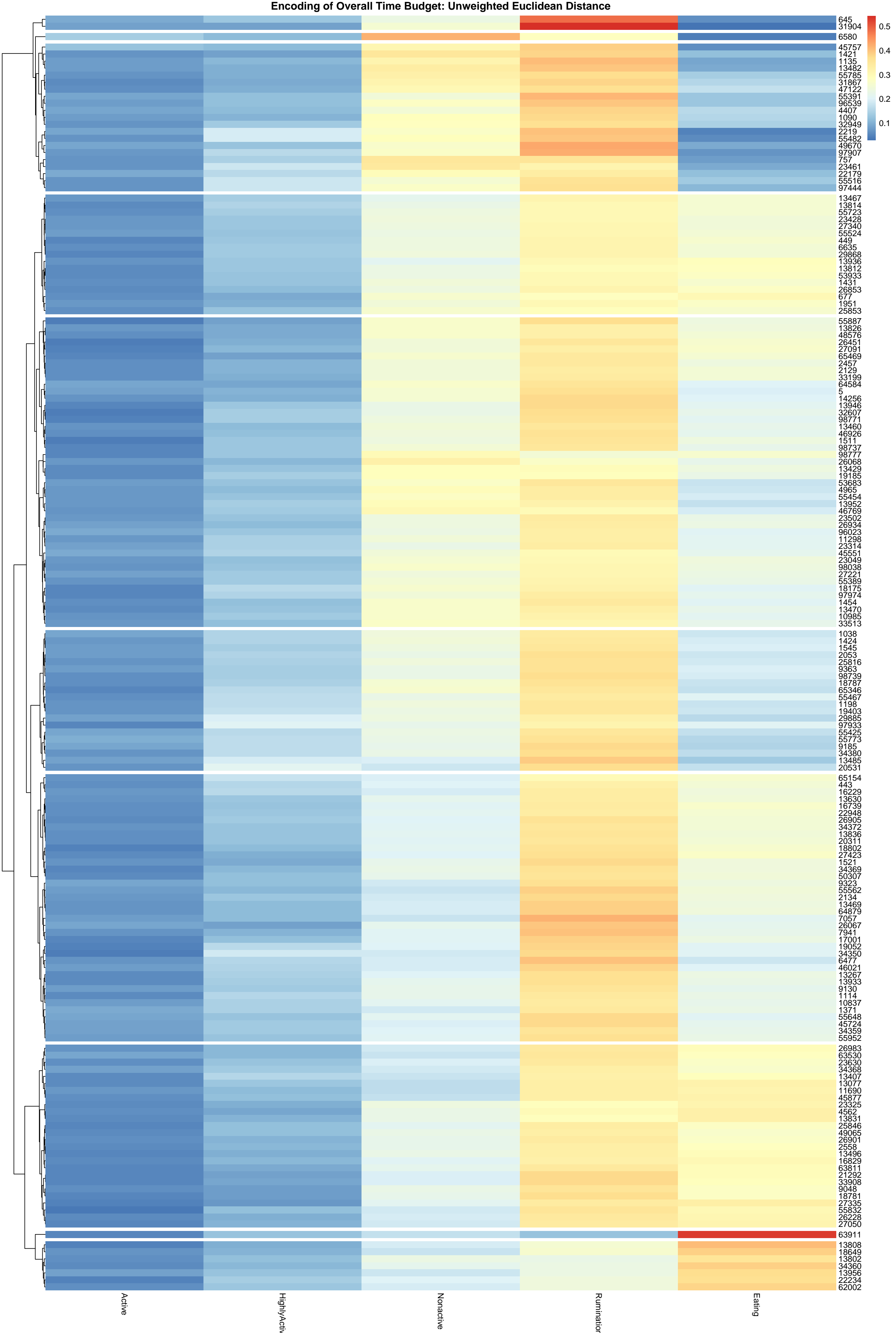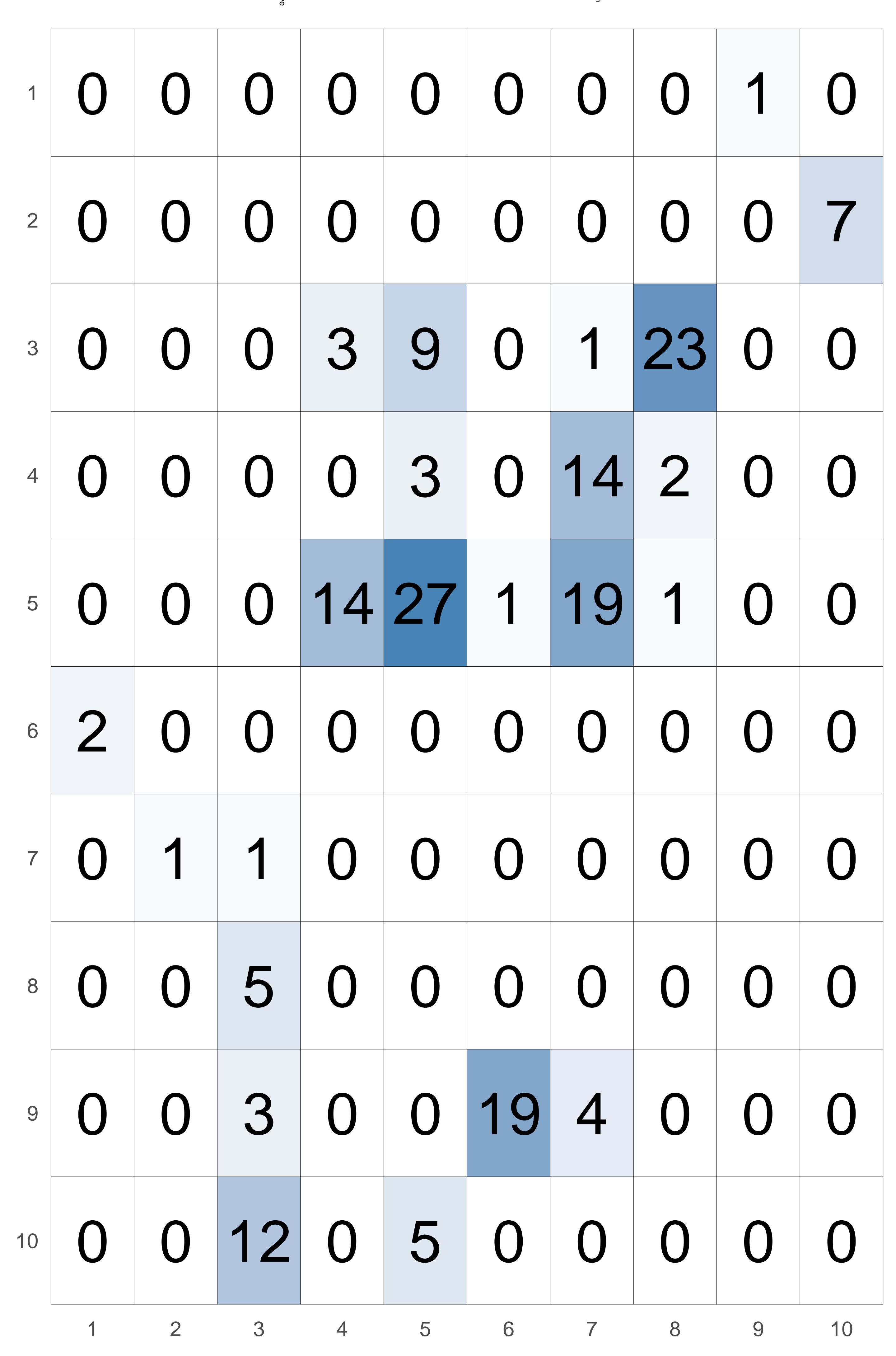

Supplement: Supplementary file 1 [file sensors-22-00001-s001.zip › sensors-1463895-supplementary/OverallTB/ContrastPlots/Euclid_vs_KLD_10x10.pdf]

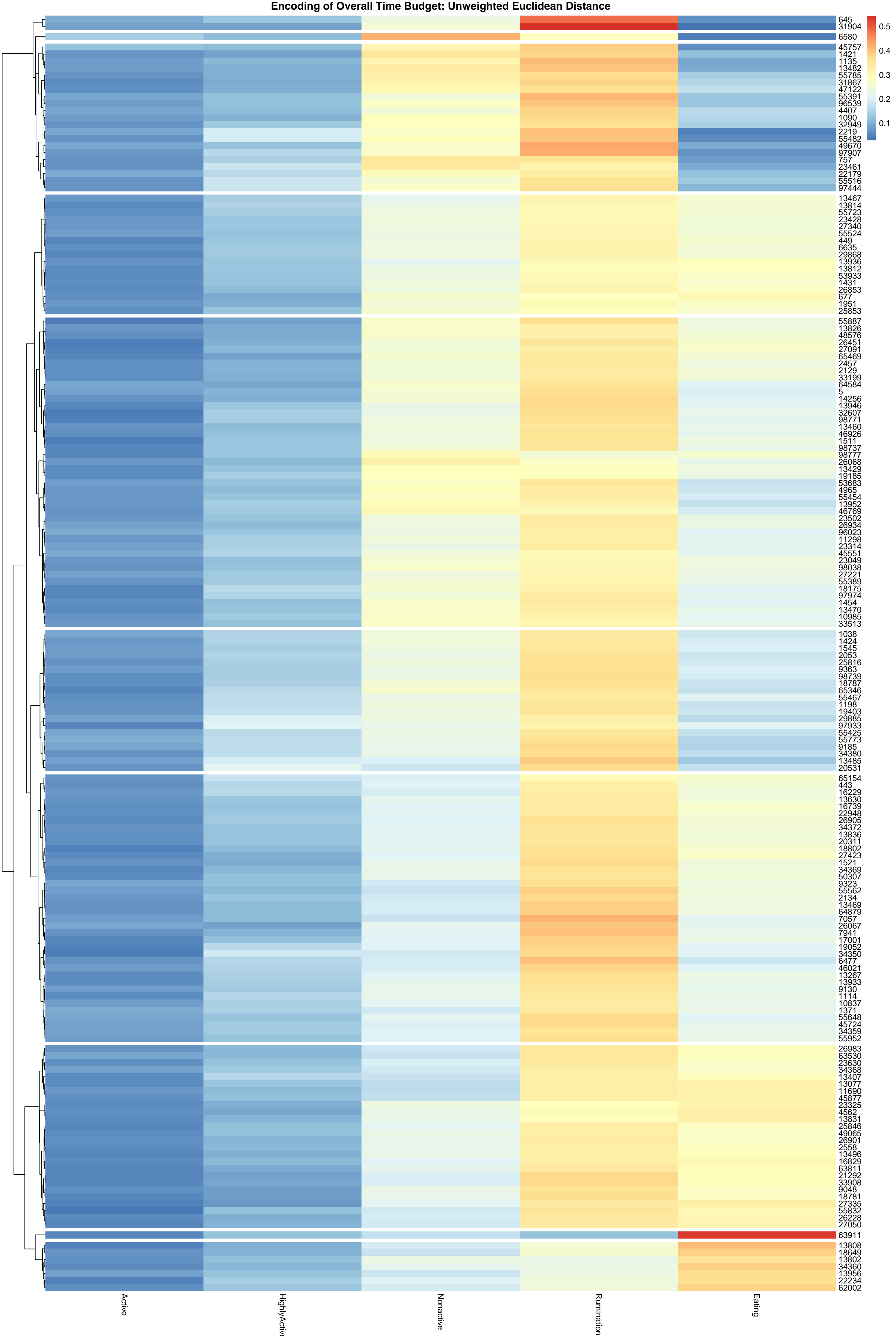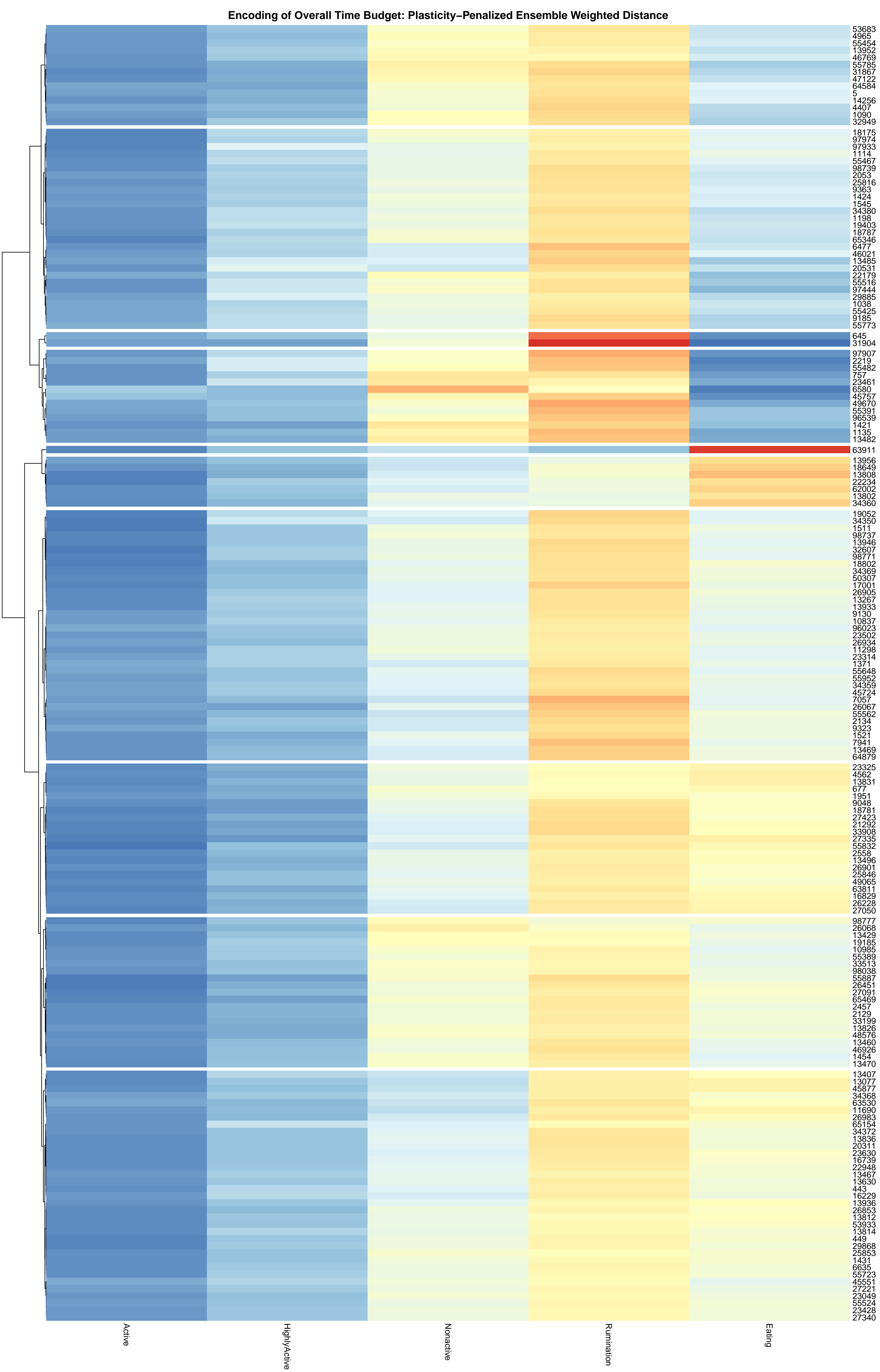

|    |   |   |    |    |    |    |    |    |   |   |
|----|---|---|----|----|----|----|----|----|---|---|
| 1  | 0 | 0 | 6  | 0  | 8  | 0  | 0  | 0  | 0 | 0 |
| 2  | 0 | 0 | 3  | 0  | 2  | 20 | 3  | 0  | 0 | 0 |
| 3  | 2 | 0 | 0  | 0  | 0  | 0  | 0  | 0  | 0 | 0 |
| 4  | 0 | 1 | 12 | 0  | 0  | 0  | 0  | 0  | 0 | 0 |
| 5  | 0 | 0 | 0  | 0  | 0  | 0  | 0  | 0  | 1 | 0 |
| 6  | 0 | 0 | 0  | 0  | 0  | 0  | 0  | 0  | 0 | 7 |
| 7  | 0 | 0 | 0  | 0  | 10 | 0  | 25 | 0  | 0 | 0 |
| 8  | 0 | 0 | 0  | 2  | 0  | 0  | 1  | 18 | 0 | 0 |
| 9  | 0 | 0 | 0  | 0  | 21 | 0  | 0  | 0  | 0 | 0 |
| 10 | 0 | 0 | 0  | 15 | 3  | 0  | 9  | 8  | 0 | 0 |

Supplement: Supplementary file 1 [file sensors-22-00001-s001.zip › sensors-1463895-supplementary/OverallTB/ContrastPlots/Euclid_vs_PW_10x10.pdf]

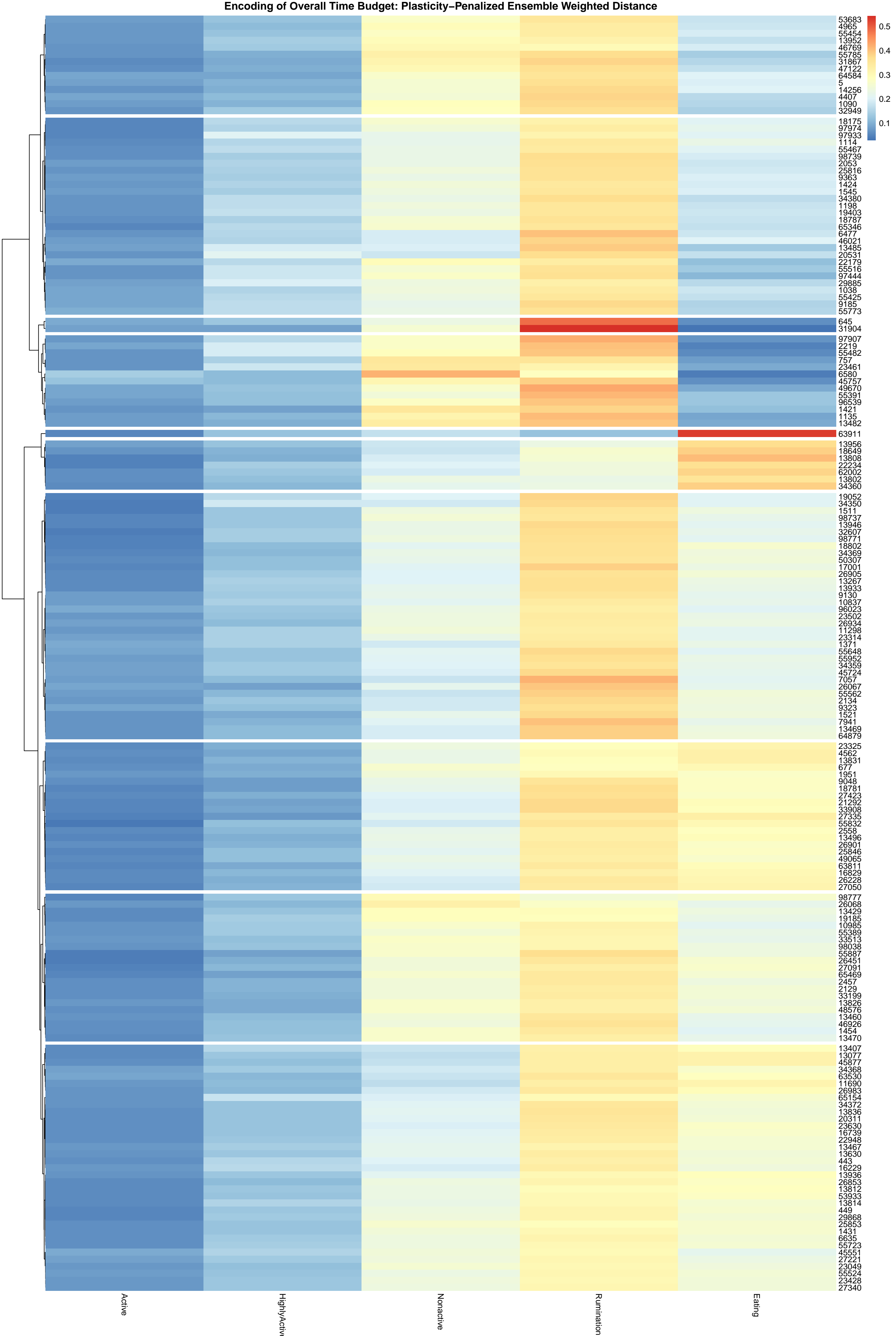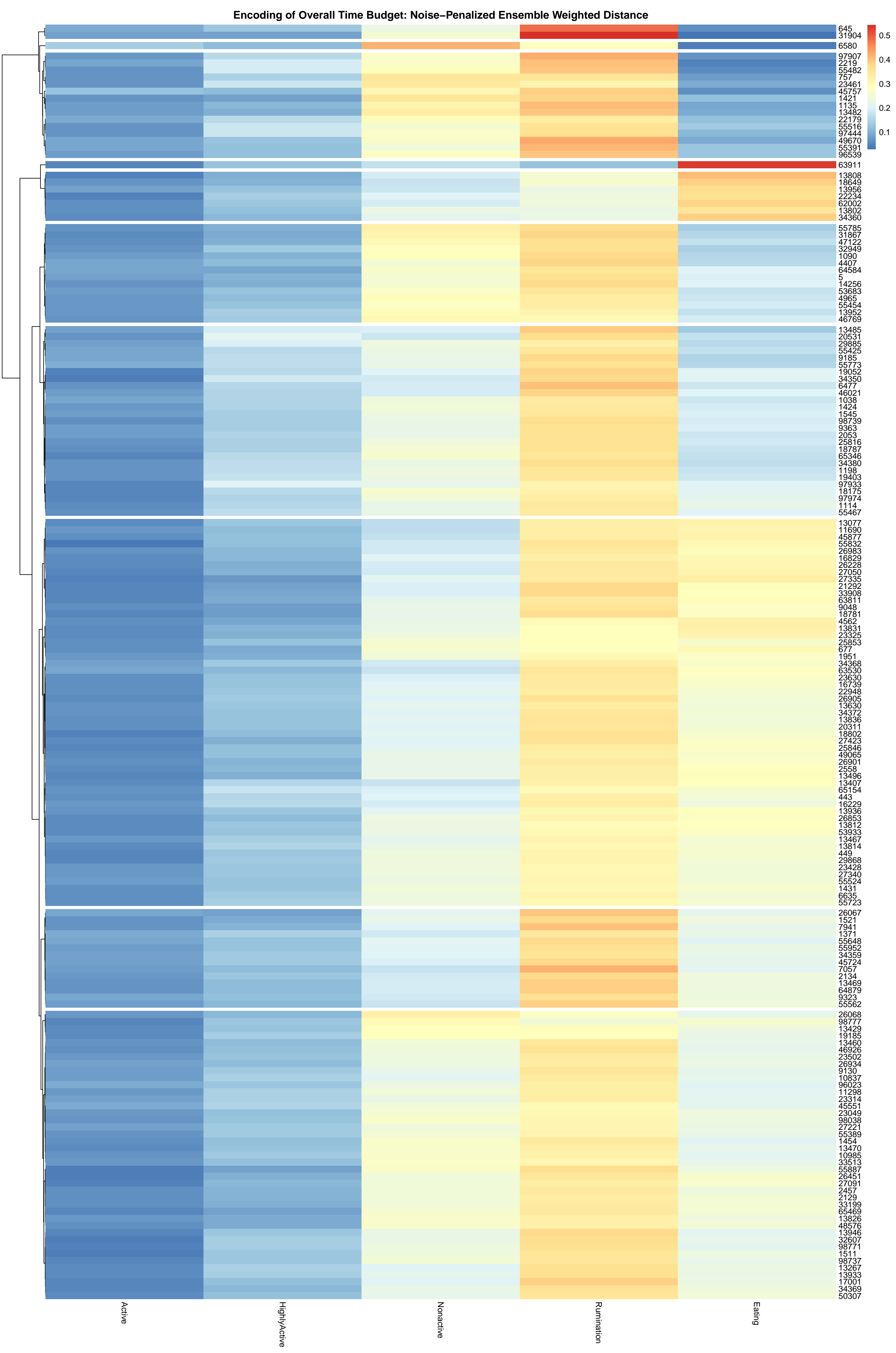

|    | 1  | 2  | 3 | 4  | 5 | 6 | 7  | 8  | 9  | 10 |
|----|----|----|---|----|---|---|----|----|----|----|
| 1  | 0  | 0  | 2 | 0  | 0 | 0 | 0  | 0  | 0  | 0  |
| 2  | 0  | 0  | 0 | 1  | 0 | 0 | 0  | 0  | 0  | 0  |
| 3  | 0  | 3  | 0 | 12 | 0 | 0 | 0  | 0  | 0  | 0  |
| 4  | 0  | 0  | 0 | 0  | 1 | 0 | 0  | 0  | 0  | 0  |
| 5  | 0  | 0  | 0 | 0  | 0 | 7 | 0  | 0  | 0  | 0  |
| 6  | 14 | 0  | 0 | 0  | 0 | 0 | 0  | 0  | 0  | 0  |
| 7  | 0  | 25 | 0 | 0  | 0 | 0 | 2  | 0  | 0  | 0  |
| 8  | 0  | 0  | 0 | 0  | 0 | 0 | 2  | 21 | 0  | 32 |
| 9  | 0  | 0  | 0 | 0  | 0 | 0 | 14 | 0  | 0  | 0  |
| 10 | 0  | 0  | 0 | 0  | 0 | 0 | 17 | 0  | 21 | 3  |

Supplement: Supplementary file 1 [file sensors-22-00001-s001.zip › sensors-1463895-supplementary/OverallTB/ContrastPlots/EW_vs_PW_10x10.pdf]

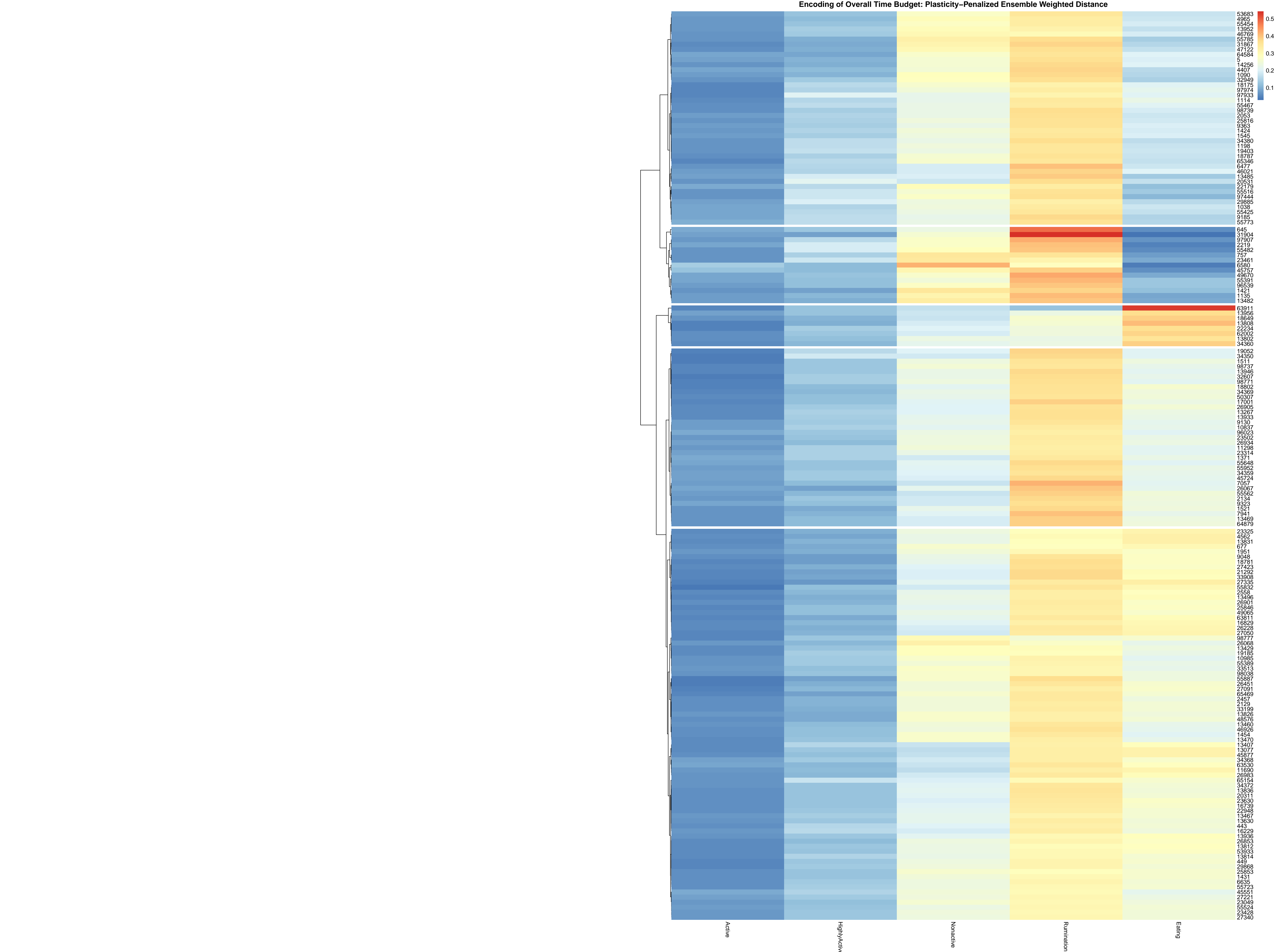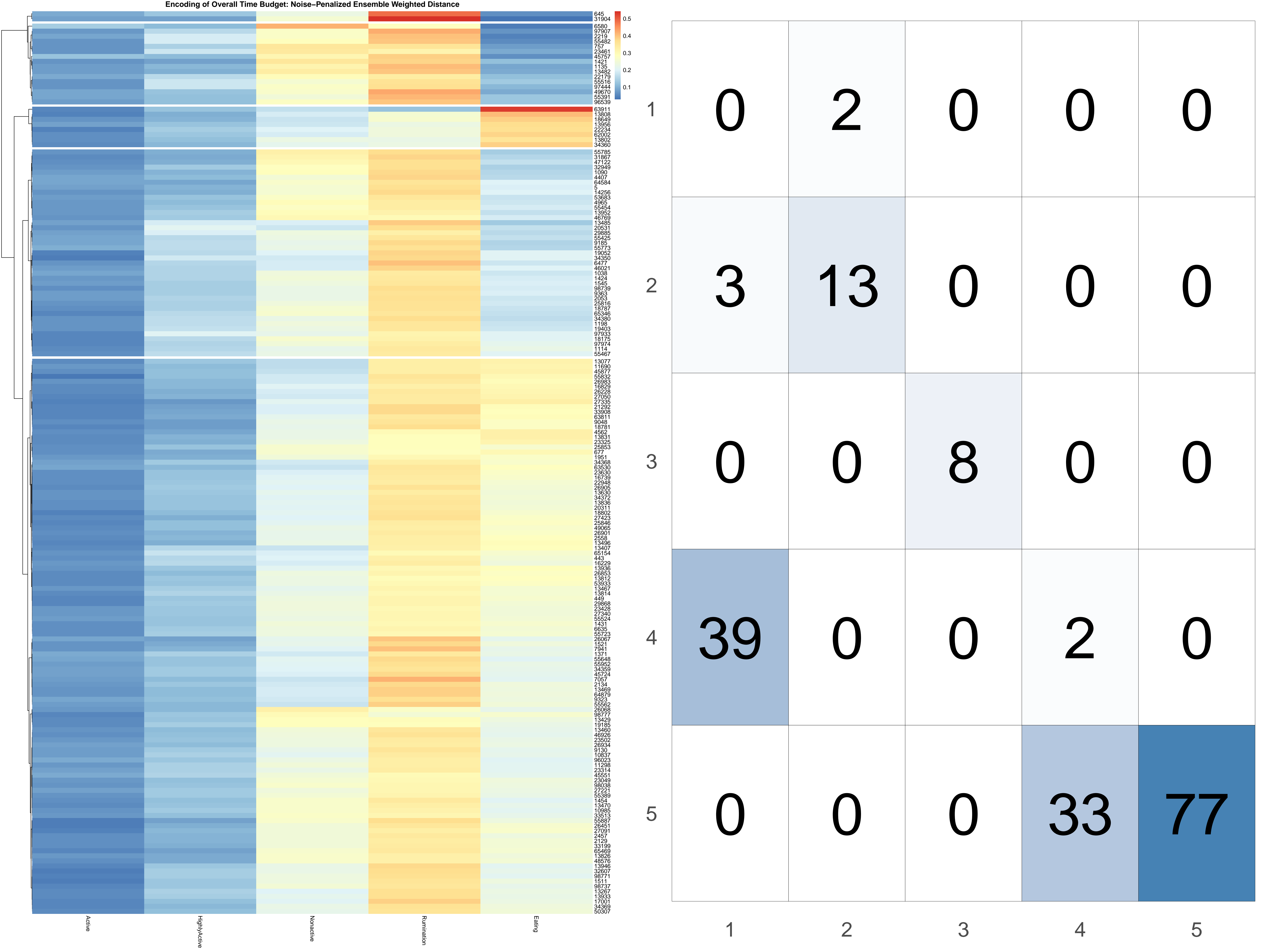

Supplement: Supplementary file 1 [file sensors-22-00001-s001.zip › sensors-1463895-supplementary/OverallTB/ContrastPlots/EW_vs_PW_5x5.pdf]

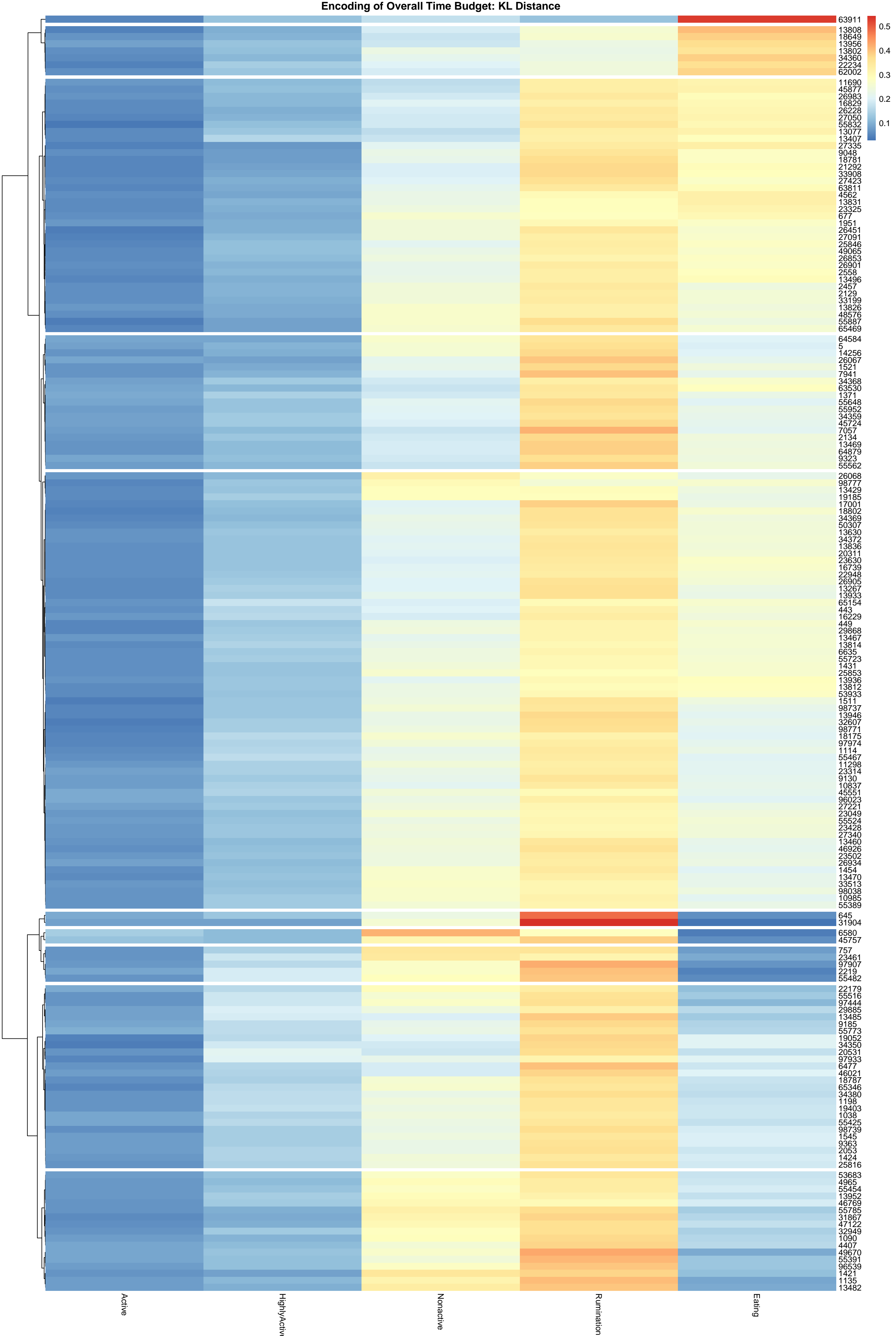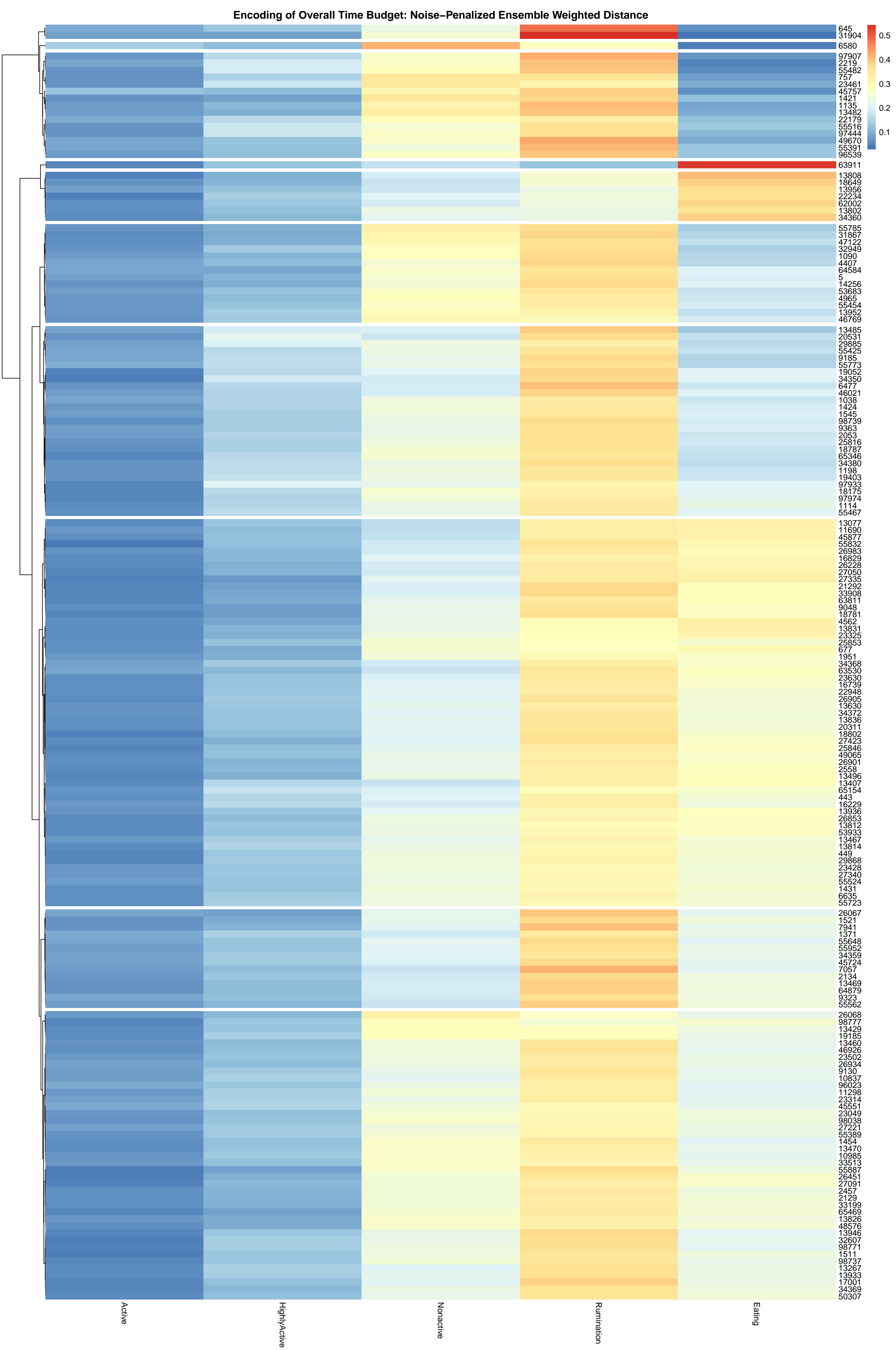

|    | 1 | 2 | 3  | 4  | 5  | 6 | 7 | 8 | 9  | 10 |
|----|---|---|----|----|----|---|---|---|----|----|
| 1  | 0 | 0 | 0  | 0  | 0  | 2 | 0 | 0 | 0  | 0  |
| 2  | 0 | 0 | 0  | 0  | 0  | 0 | 1 | 0 | 0  | 0  |
| 3  | 0 | 0 | 0  | 0  | 0  | 0 | 1 | 5 | 3  | 6  |
| 4  | 1 | 0 | 0  | 0  | 0  | 0 | 0 | 0 | 0  | 0  |
| 5  | 0 | 7 | 0  | 0  | 0  | 0 | 0 | 0 | 0  | 0  |
| 6  | 0 | 0 | 0  | 3  | 0  | 0 | 0 | 0 | 0  | 11 |
| 7  | 0 | 0 | 0  | 0  | 4  | 0 | 0 | 0 | 23 | 0  |
| 8  | 0 | 0 | 27 | 2  | 26 | 0 | 0 | 0 | 0  | 0  |
| 9  | 0 | 0 | 0  | 14 | 0  | 0 | 0 | 0 | 0  | 0  |
| 10 | 0 | 0 | 9  | 0  | 32 | 0 | 0 | 0 | 0  | 0  |

Supplement: Supplementary file 1 [file sensors-22-00001-s001.zip › sensors-1463895-supplementary/OverallTB/ContrastPlots/KLD_vs_EW_10x10.pdf]

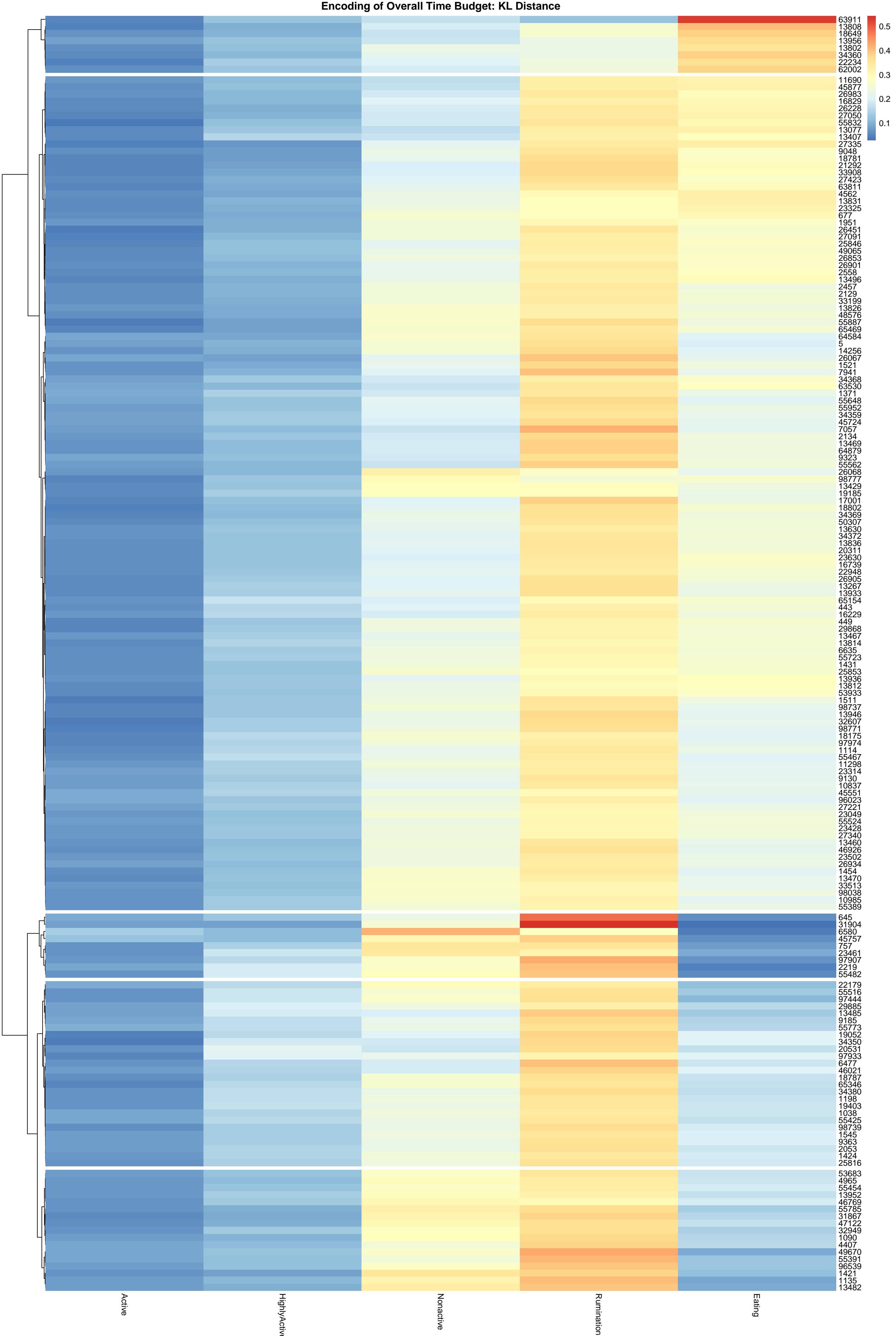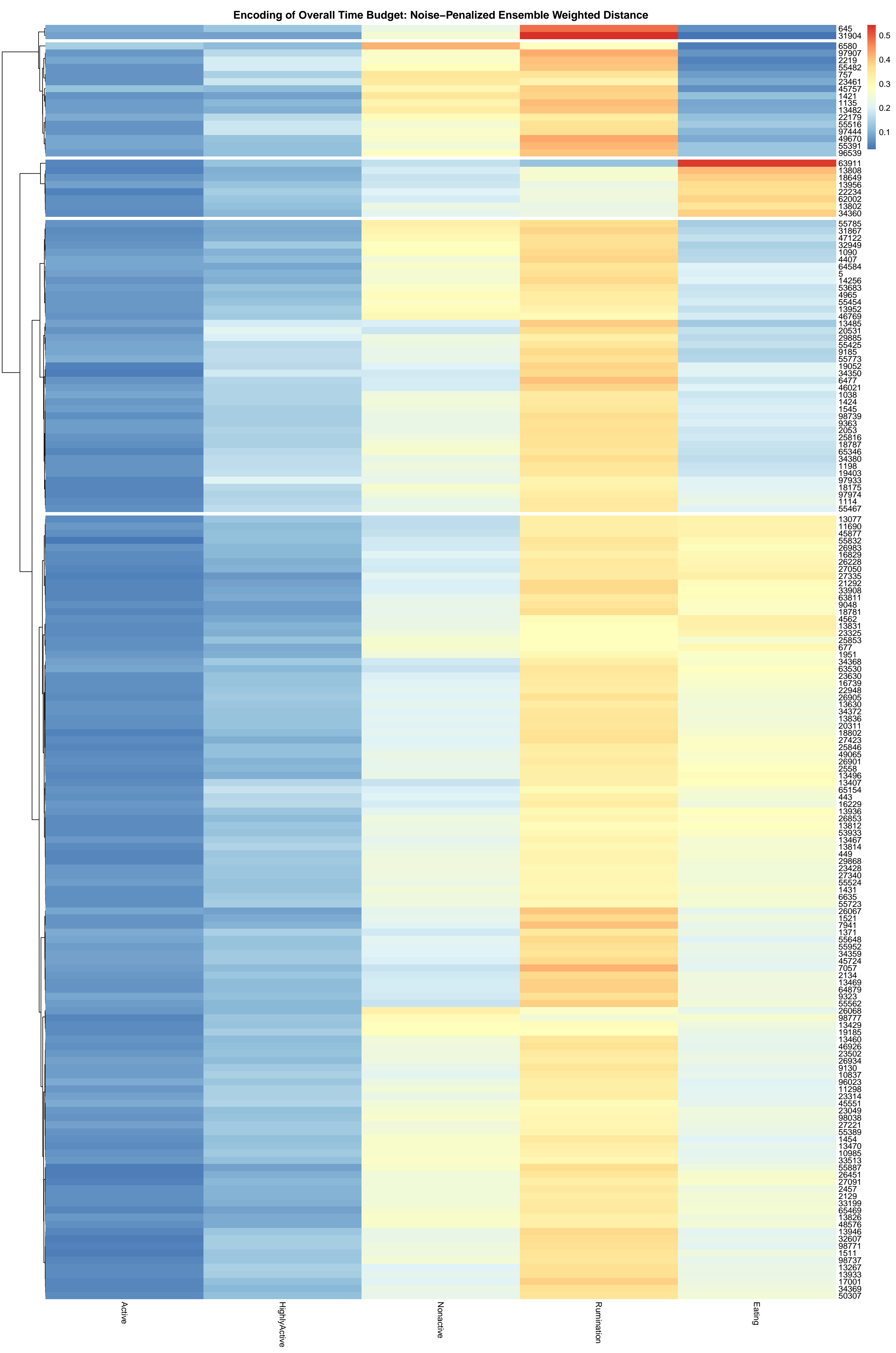

|   |   |     |   |    |    |
|---|---|-----|---|----|----|
| 1 | 0 | 0   | 2 | 0  | 0  |
| 2 | 0 | 0   | 7 | 3  | 6  |
| 3 | 8 | 0   | 0 | 0  | 0  |
| 4 | 0 | 7   | 0 | 23 | 11 |
| 5 | 0 | 110 | 0 | 0  | 0  |
|   | 1 | 2   | 3 | 4  | 5  |

Supplement: Supplementary file 1 [file sensors-22-00001-s001.zip › sensors-1463895-supplementary/OverallTB/ContrastPlots/KLD_vs_EW_5x5.pdf]

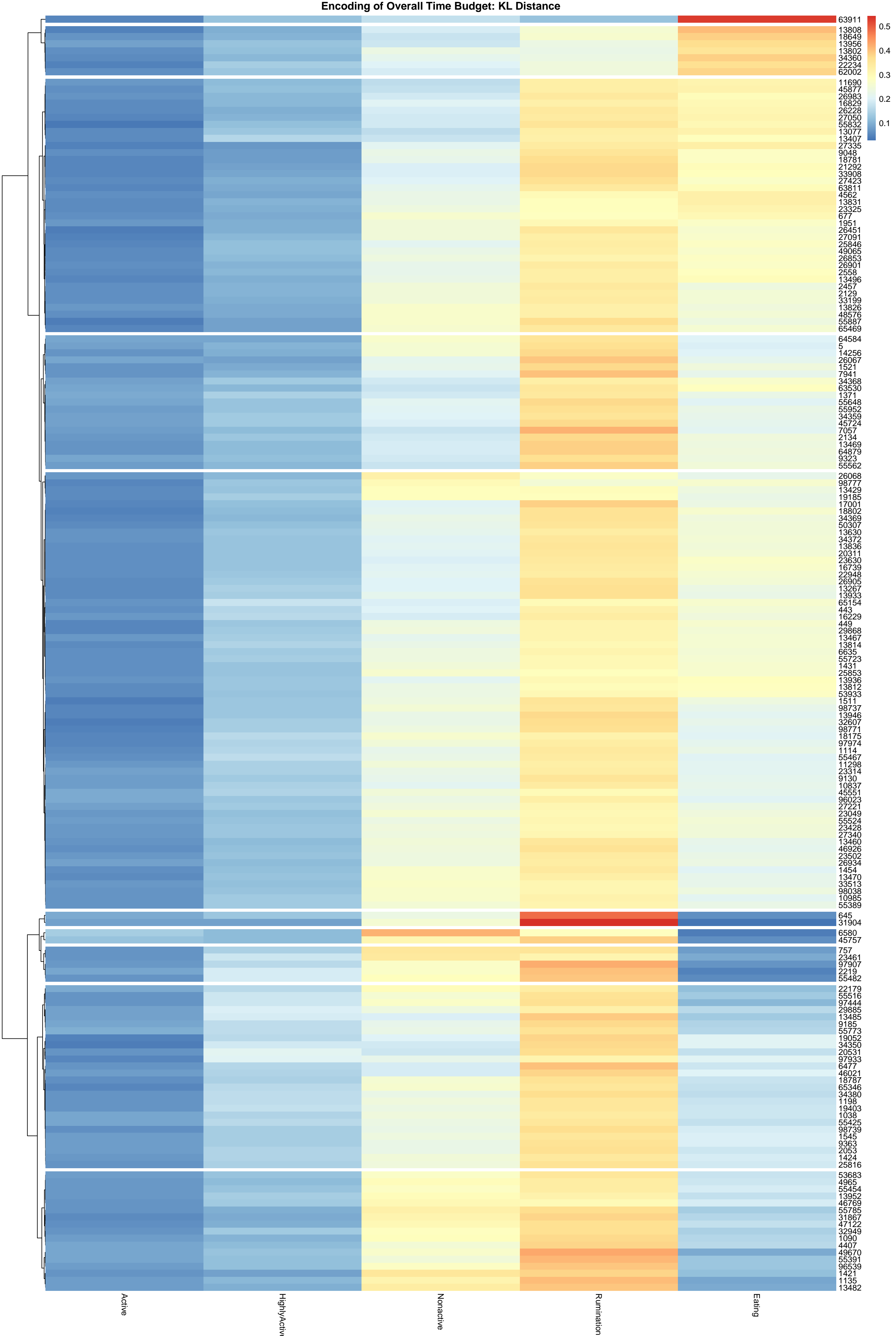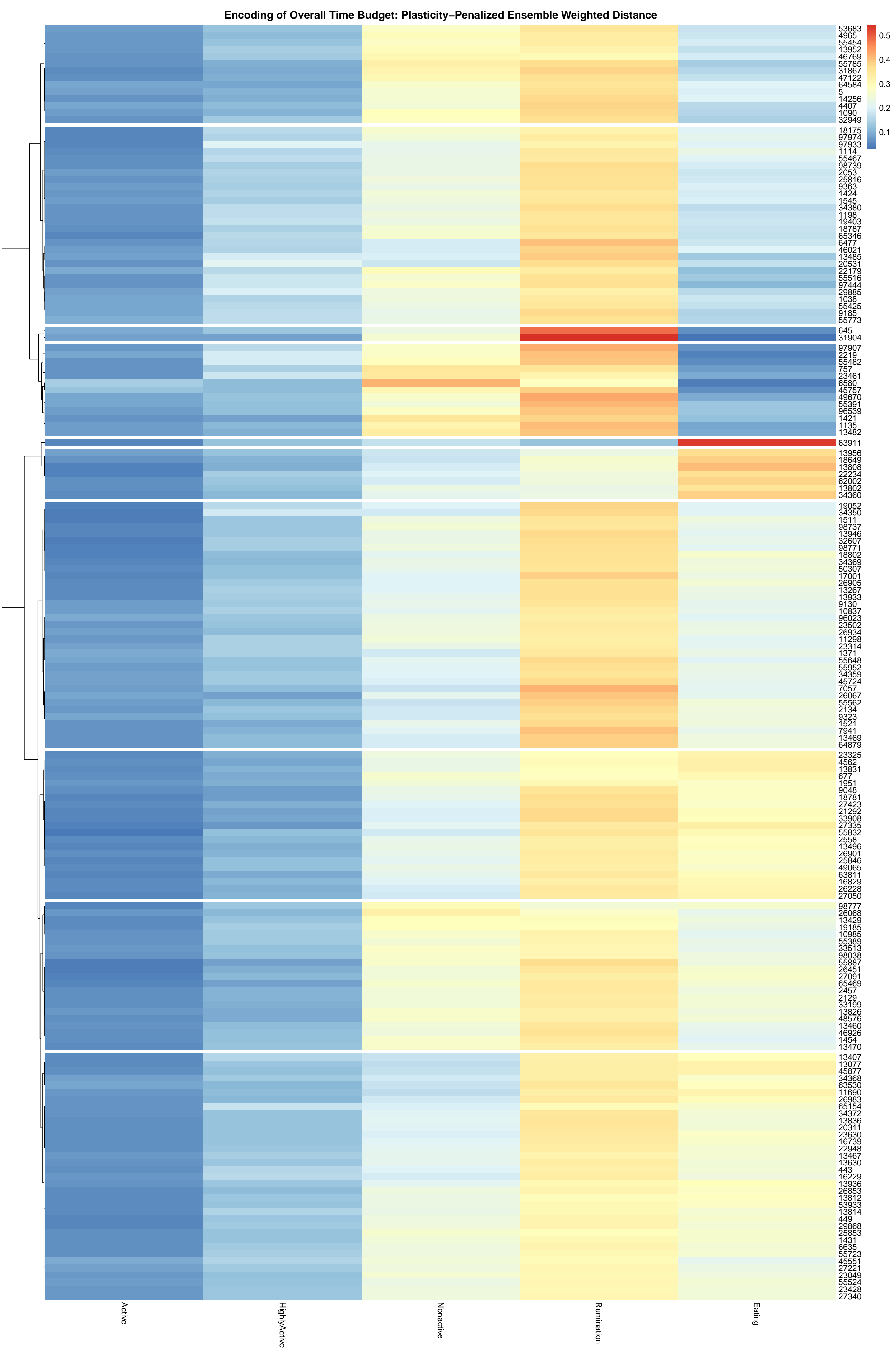

|    |   |   |    |    |    |   |   |   |    |    |
|----|---|---|----|----|----|---|---|---|----|----|
| 1  | 0 | 0 | 0  | 3  | 0  | 0 | 0 | 0 | 0  | 11 |
| 2  | 0 | 0 | 0  | 0  | 4  | 0 | 0 | 0 | 24 | 0  |
| 3  | 0 | 0 | 0  | 0  | 0  | 2 | 0 | 0 | 0  | 0  |
| 4  | 0 | 0 | 0  | 0  | 0  | 0 | 2 | 5 | 0  | 6  |
| 5  | 1 | 0 | 0  | 0  | 0  | 0 | 0 | 0 | 0  | 0  |
| 6  | 0 | 7 | 0  | 0  | 0  | 0 | 0 | 0 | 0  | 0  |
| 7  | 0 | 0 | 0  | 14 | 19 | 0 | 0 | 0 | 2  | 0  |
| 8  | 0 | 0 | 21 | 0  | 0  | 0 | 0 | 0 | 0  | 0  |
| 9  | 0 | 0 | 9  | 0  | 12 | 0 | 0 | 0 | 0  | 0  |
| 10 | 0 | 0 | 6  | 2  | 27 | 0 | 0 | 0 | 0  | 0  |
|    | 1 | 2 | 3  | 4  | 5  | 6 | 7 | 8 | 9  | 10 |

Supplement: Supplementary file 1 [file sensors-22-00001-s001.zip › sensors-1463895-supplementary/OverallTB/ContrastPlots/KLD_vs_PW_10x10.pdf]

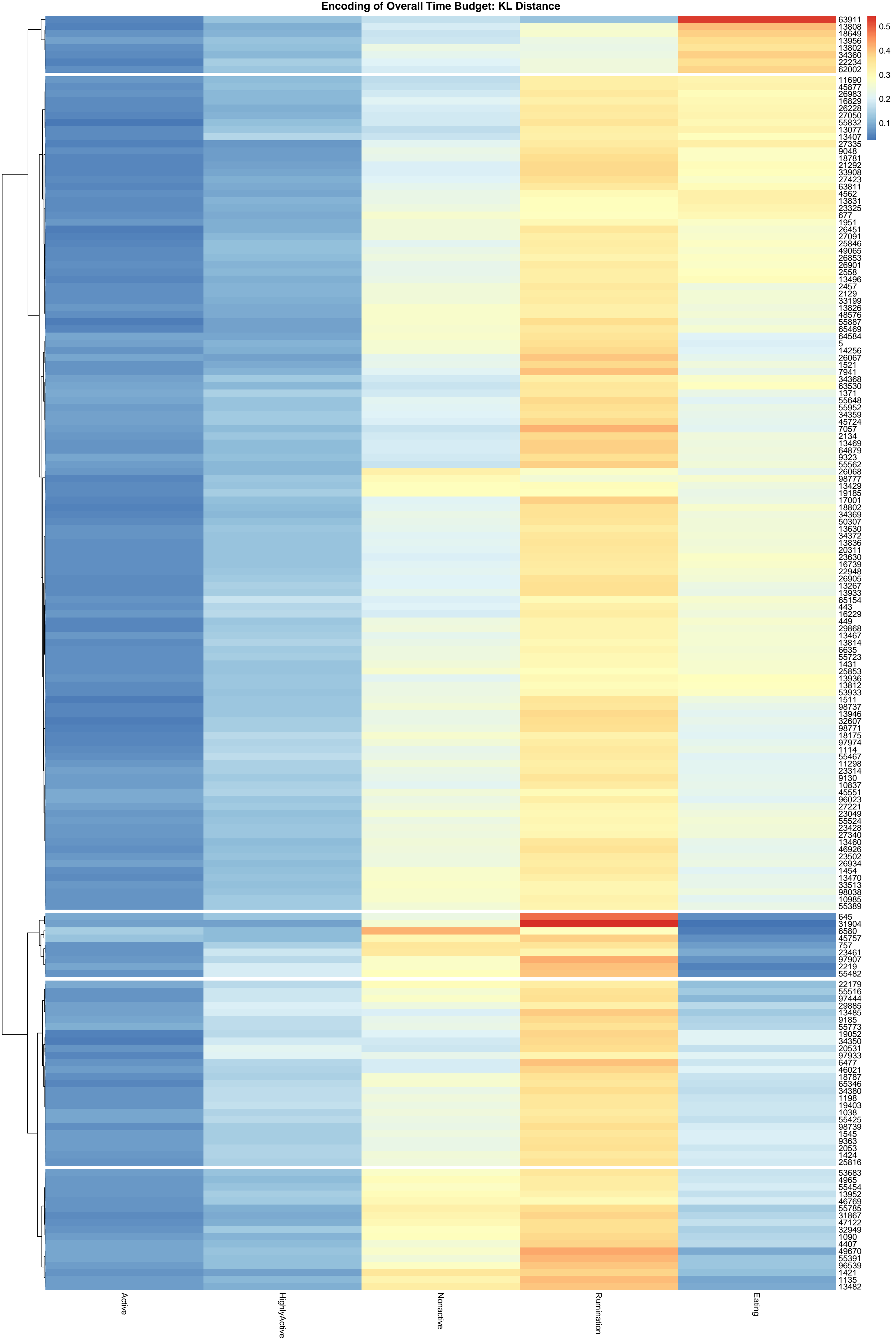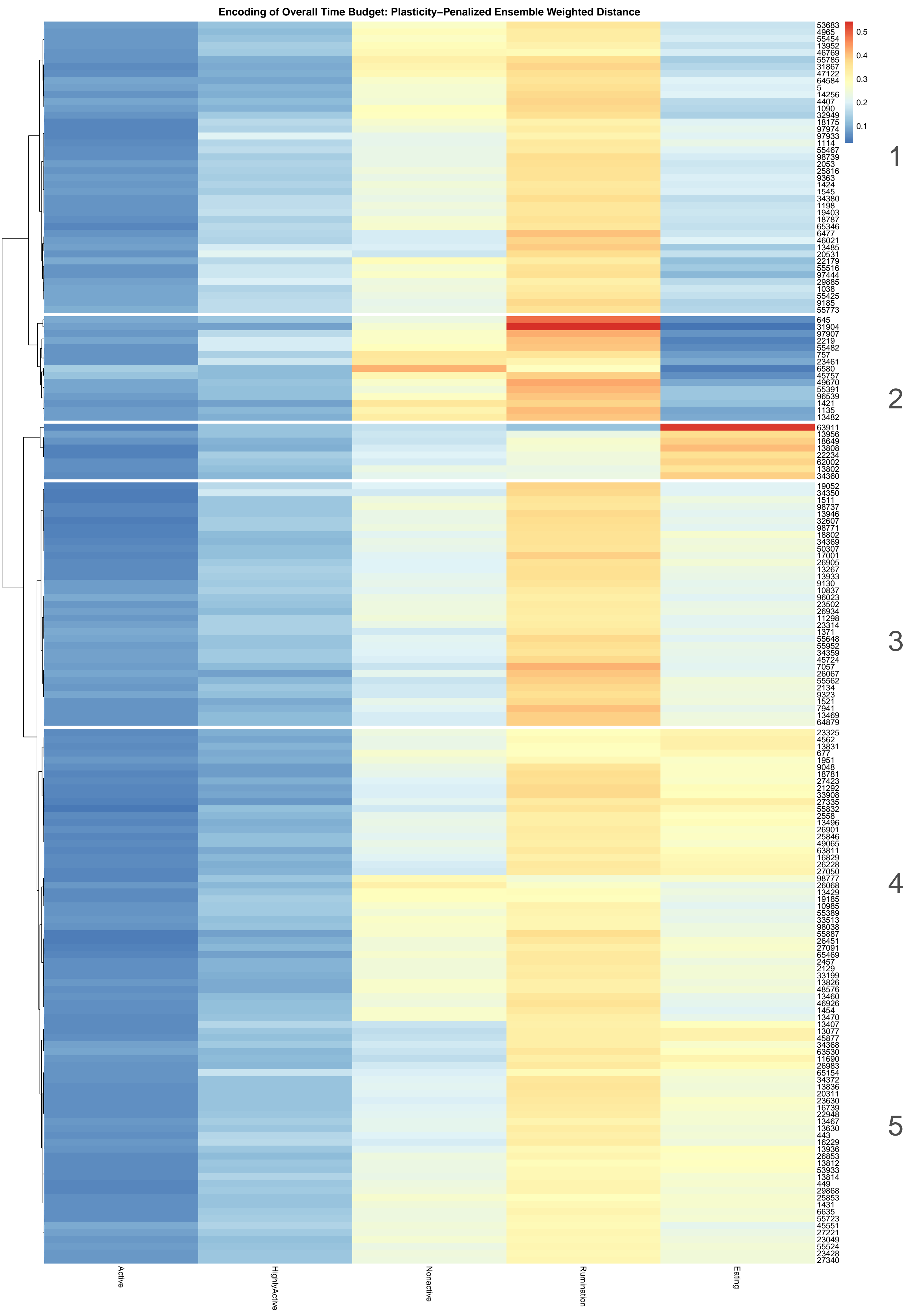

|   |   |    |   |    |    |
|---|---|----|---|----|----|
| 1 | 0 | 7  | 0 | 24 | 11 |
| 2 | 0 | 0  | 9 | 0  | 6  |
| 3 | 8 | 0  | 0 | 0  | 0  |
| 4 | 0 | 33 | 0 | 2  | 0  |
| 5 | 0 | 77 | 0 | 0  | 0  |
|   | 1 | 2  | 3 | 4  | 5  |

Supplement: Supplementary file 1 [file sensors-22-00001-s001.zip › sensors-1463895-supplementary/OverallTB/ContrastPlots/KLD_vs_PW_5x5.pdf]

# Result of Bifucation Simulation: N 177

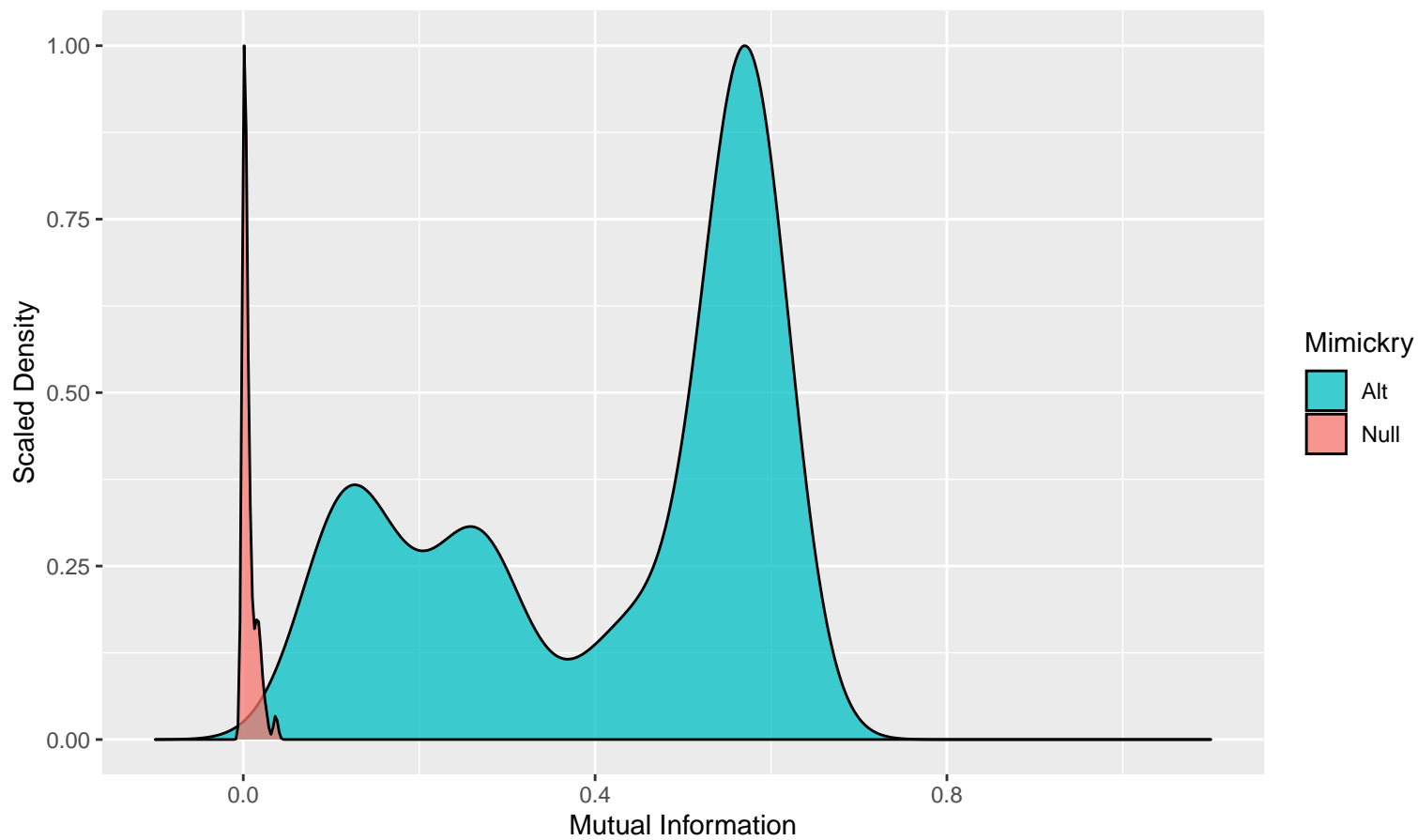

Supplement: Supplementary file 1 [file sensors-22-00001-s001.zip › sensors-1463895-supplementary/OverallTB/EnsembleCut/BifucationViz.pdf]

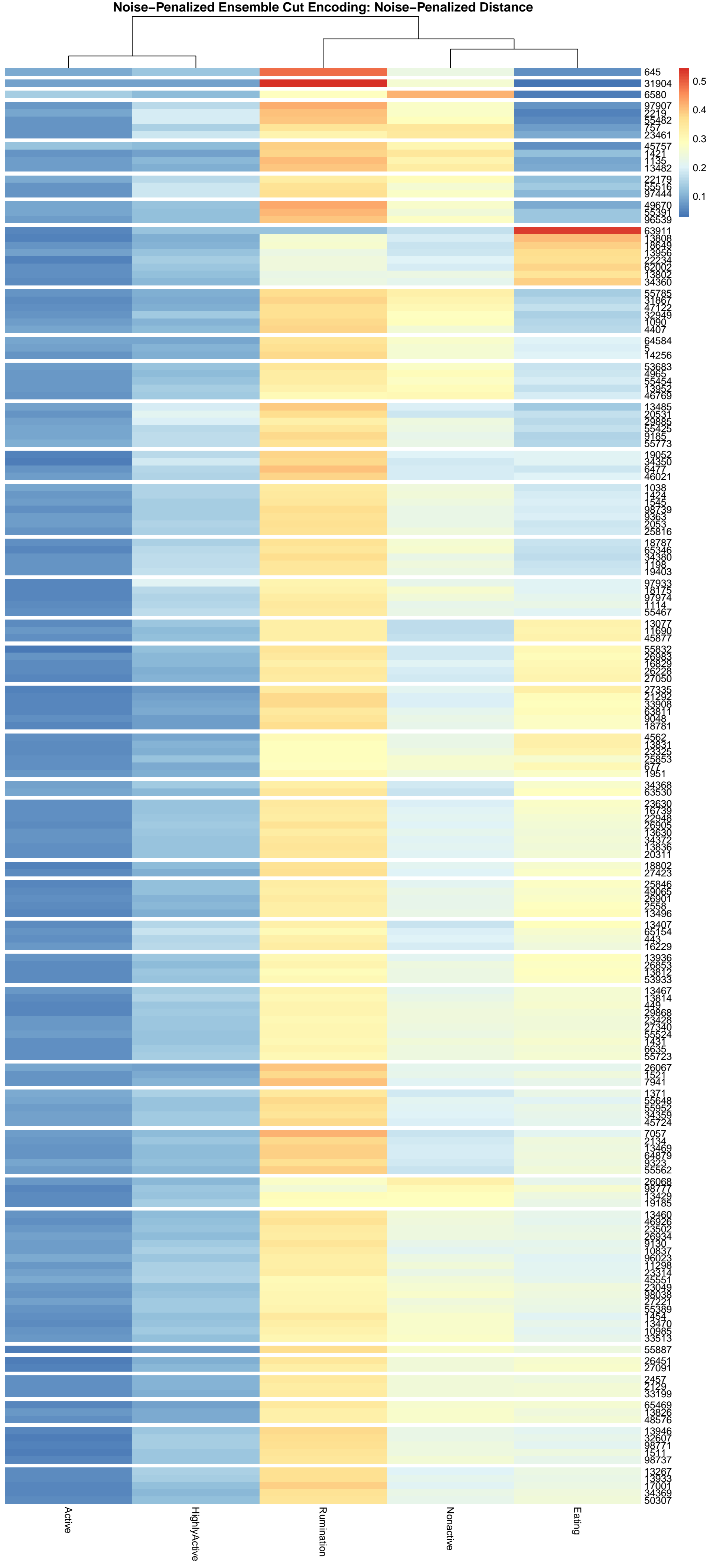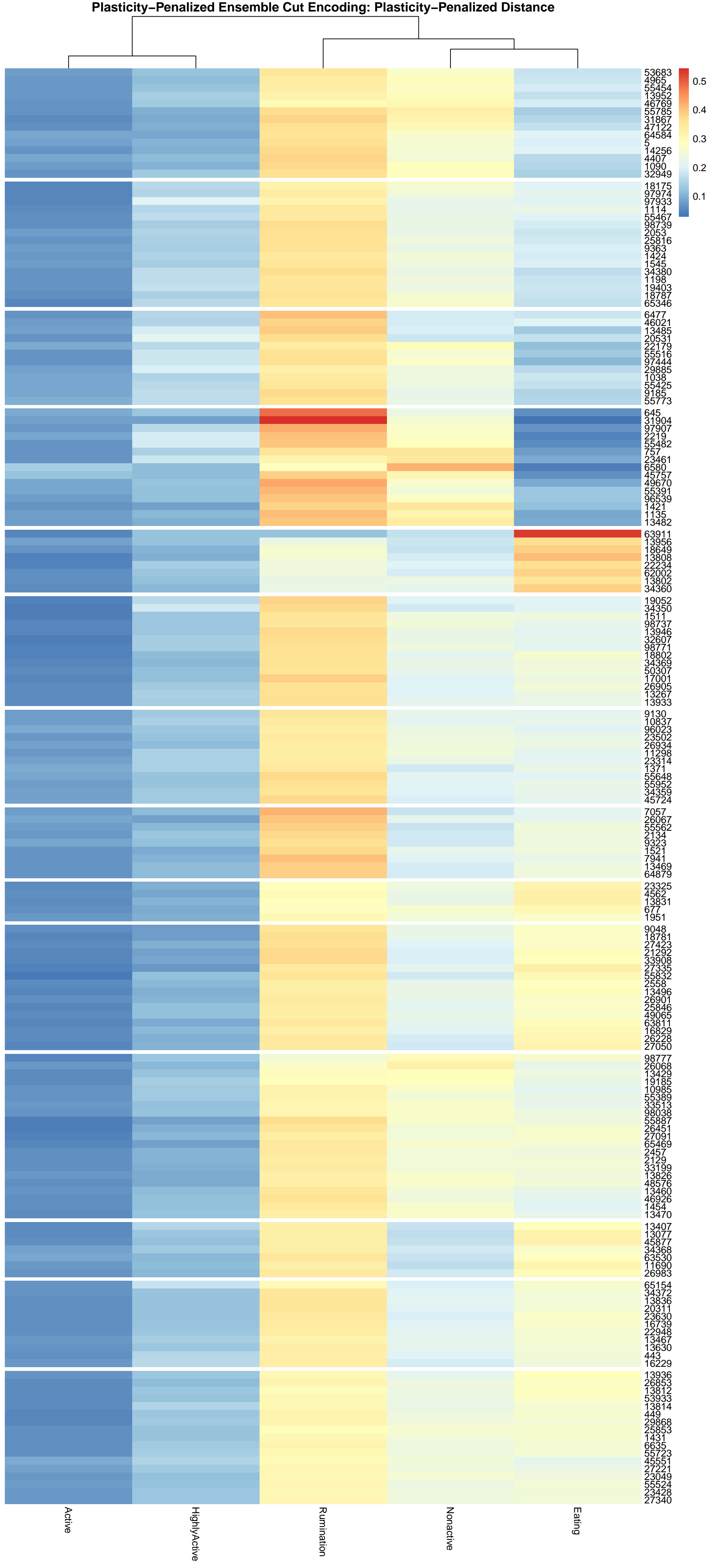

Supplement: Supplementary file 1 [file sensors-22-00001-s001.zip › sensors-1463895-supplementary/OverallTB/EnsembleCut/EnsembleEncodings.pdf]

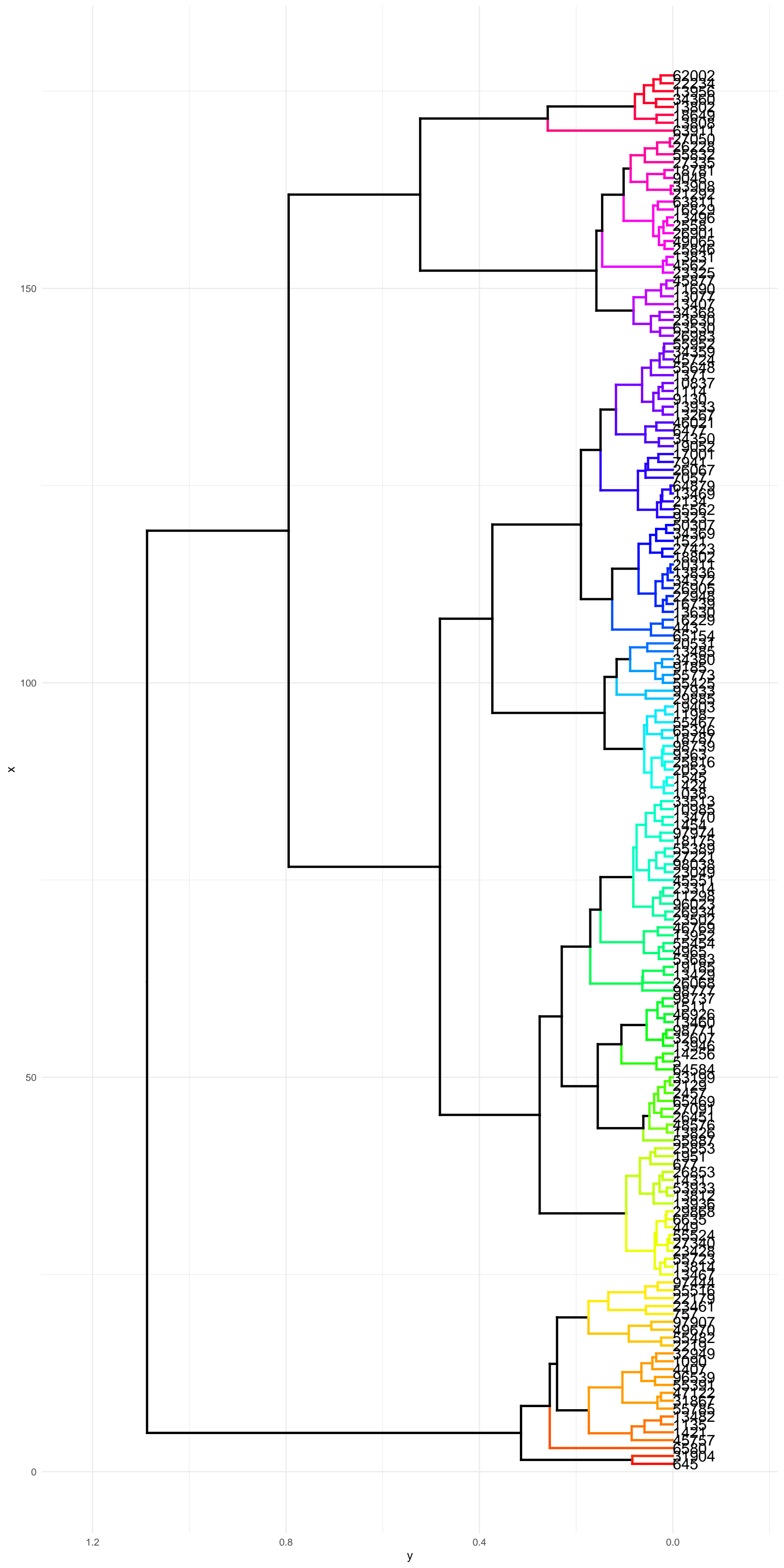

Supplement: Supplementary file 1 [file sensors-22-00001-s001.zip › sensors-1463895-supplementary/OverallTB/EnsembleCut/Euclidean_NP_Dendrogram.pdf]

Noise-Penalized Ensemble Cut Encoding: Euclidean Distance

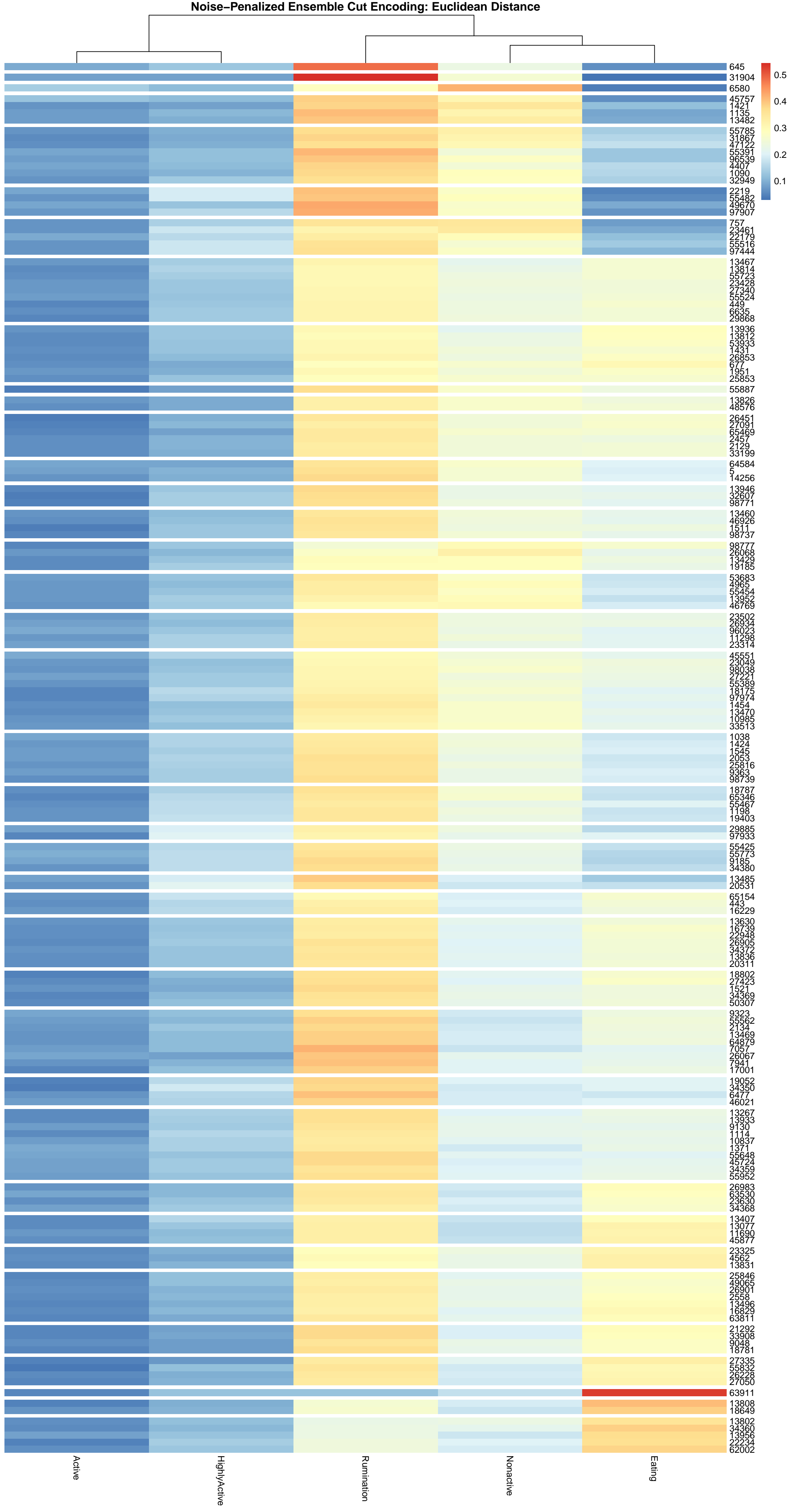

Supplement: Supplementary file 1 [file sensors-22-00001-s001.zip › sensors-1463895-supplementary/OverallTB/EnsembleCut/Euclidean_NP_Heatmap.pdf]

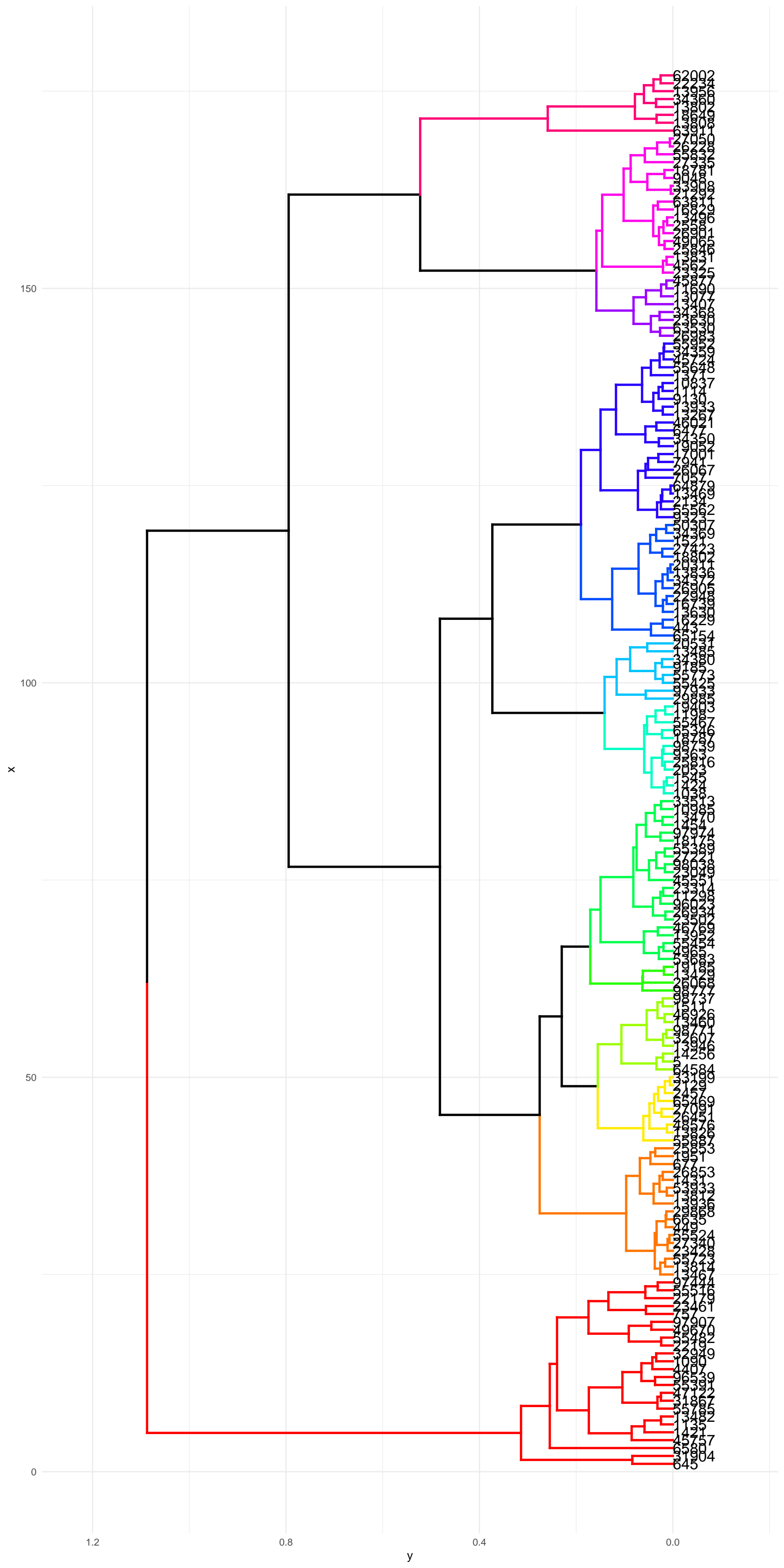

Supplement: Supplementary file 1 [file sensors-22-00001-s001.zip › sensors-1463895-supplementary/OverallTB/EnsembleCut/Euclidean_PP_Dendrogram.pdf]

Plasticity-Penalized Ensemble Cut Encoding: Euclidean Distance

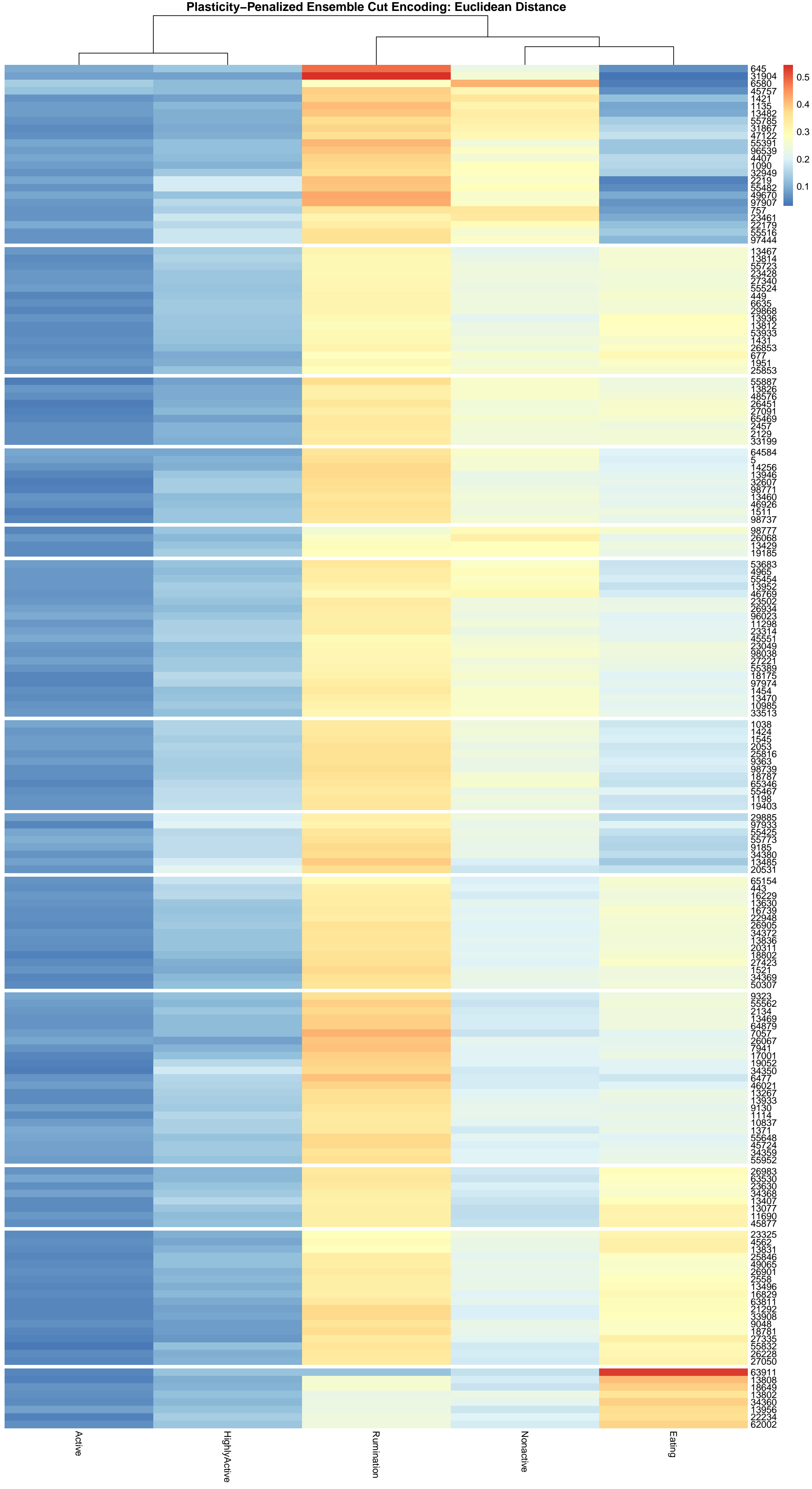

Supplement: Supplementary file 1 [file sensors-22-00001-s001.zip › sensors-1463895-supplementary/OverallTB/EnsembleCut/Euclidean_PP_Heatmap.pdf]

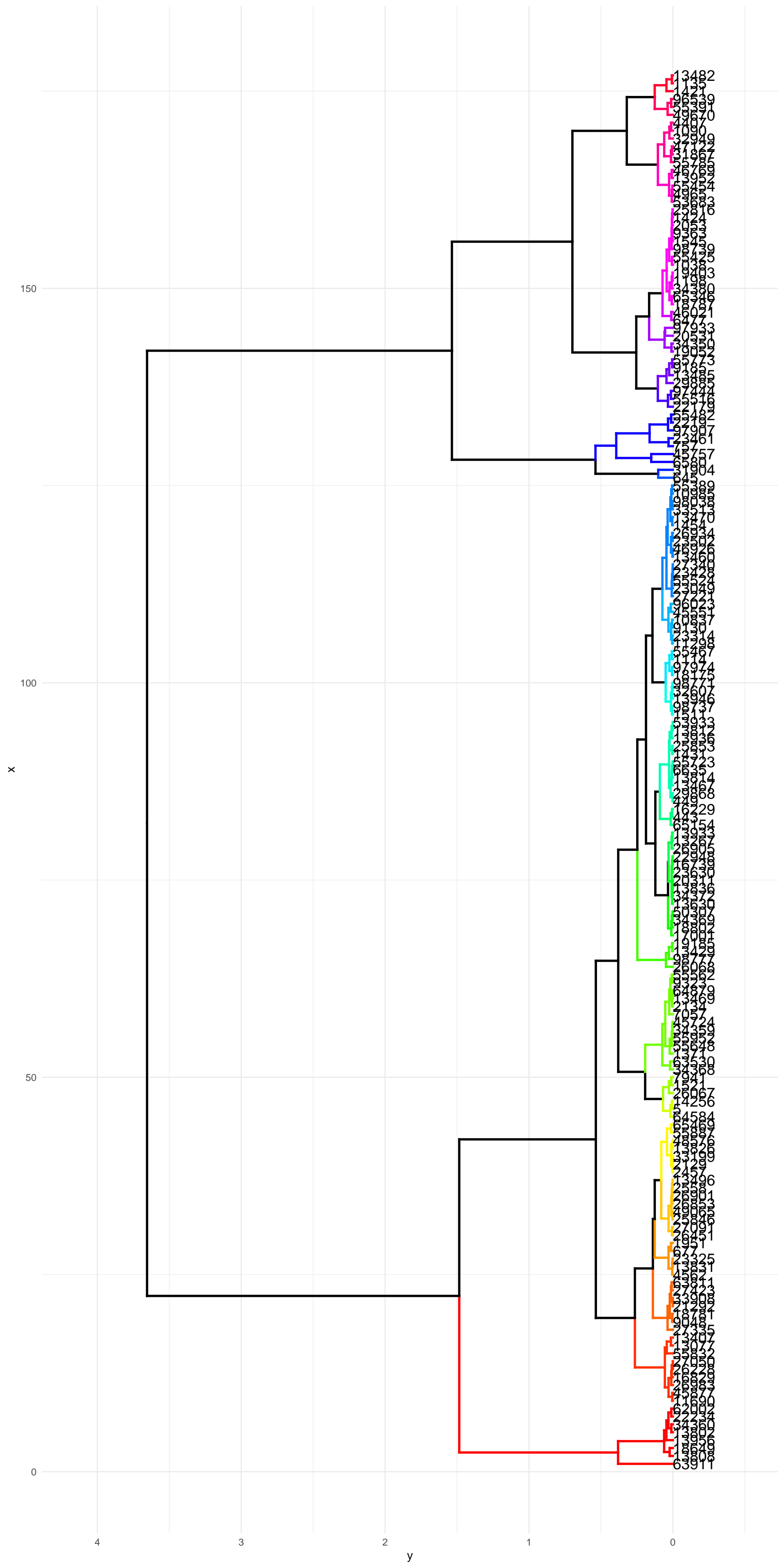

Supplement: Supplementary file 1 [file sensors-22-00001-s001.zip › sensors-1463895-supplementary/OverallTB/EnsembleCut/KLD_NP_Dendrogram.pdf]

Noise-Penalized Ensemble Cut Encoding: KL Distance

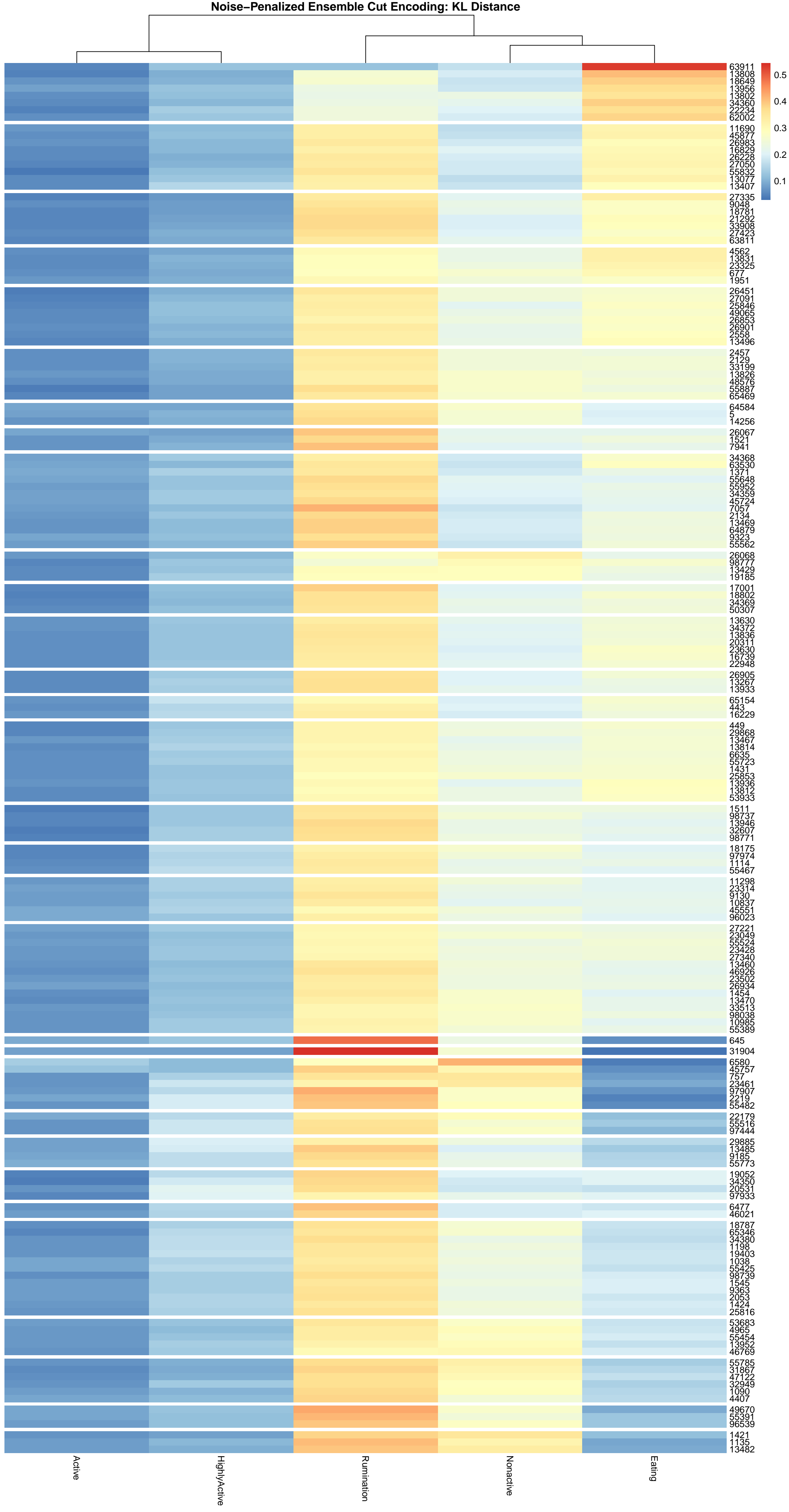

Supplement: Supplementary file 1 [file sensors-22-00001-s001.zip › sensors-1463895-supplementary/OverallTB/EnsembleCut/KLD_NP_Heatmap.pdf]

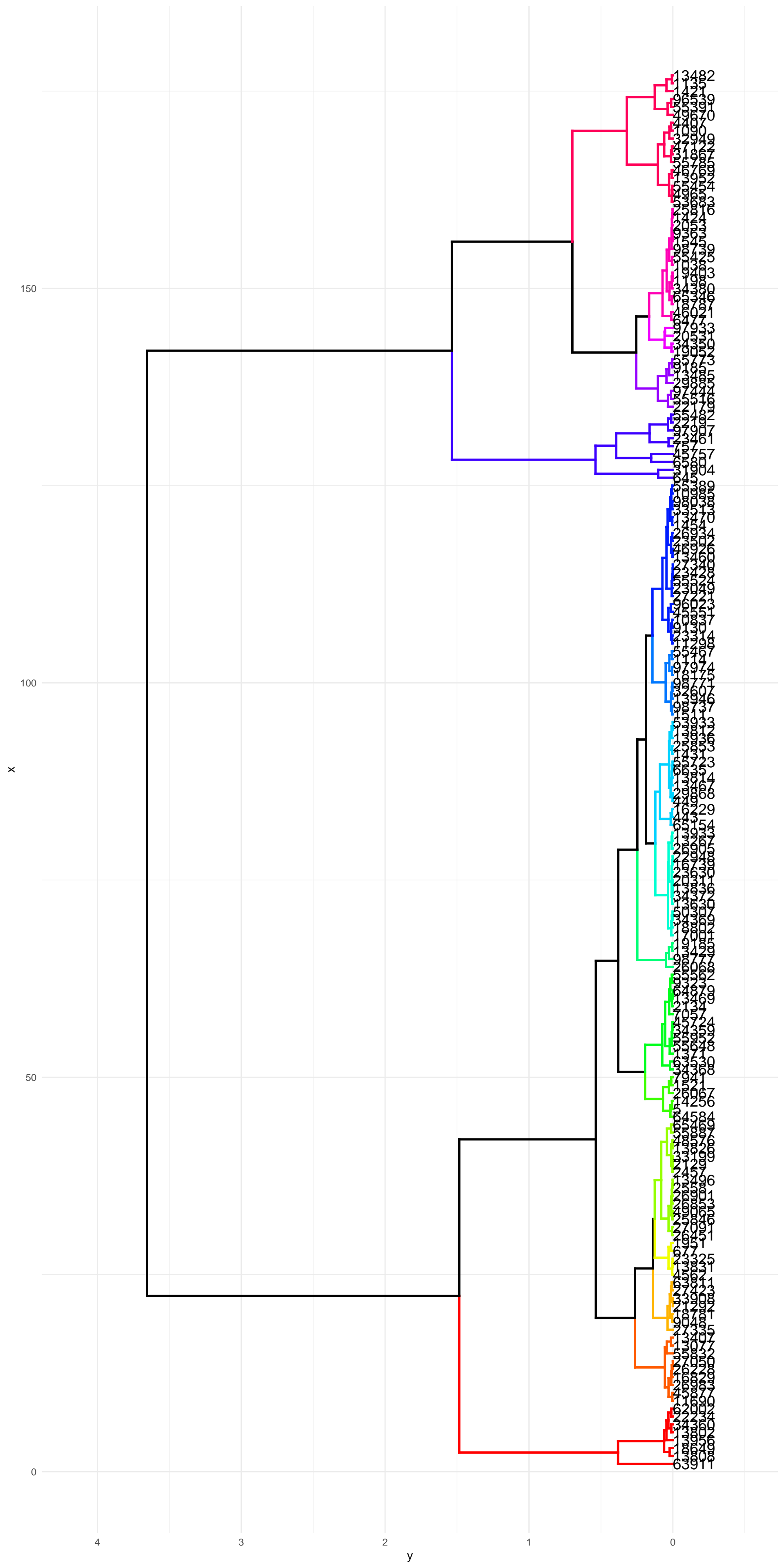

Supplement: Supplementary file 1 [file sensors-22-00001-s001.zip › sensors-1463895-supplementary/OverallTB/EnsembleCut/KLD_Sub_Dendrogram.pdf]

Plasticity-Penalized Ensemble Cut Encoding: KL Distance

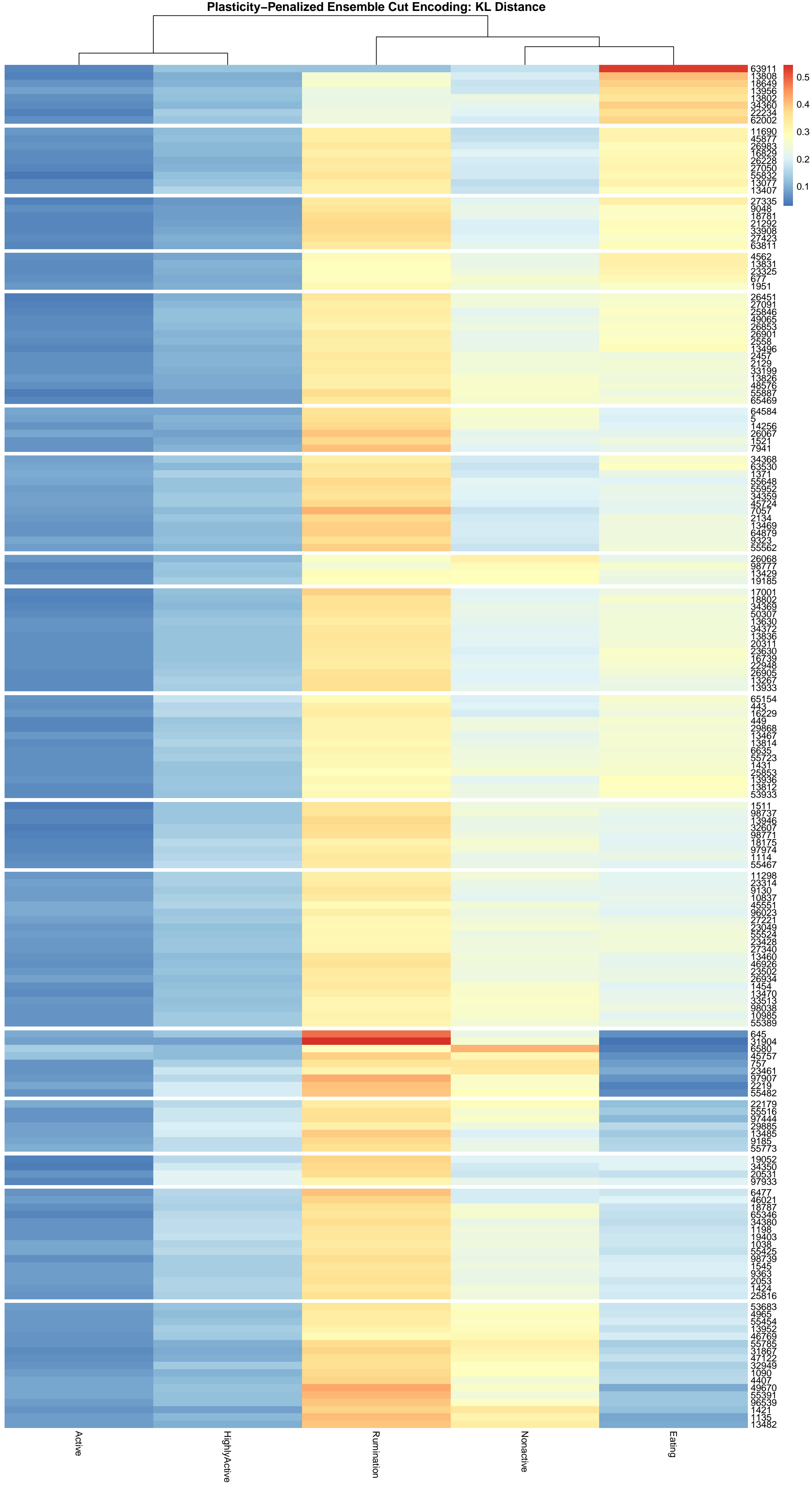

Supplement: Supplementary file 1 [file sensors-22-00001-s001.zip › sensors-1463895-supplementary/OverallTB/EnsembleCut/KLD_Sub_Heatmap.pdf]

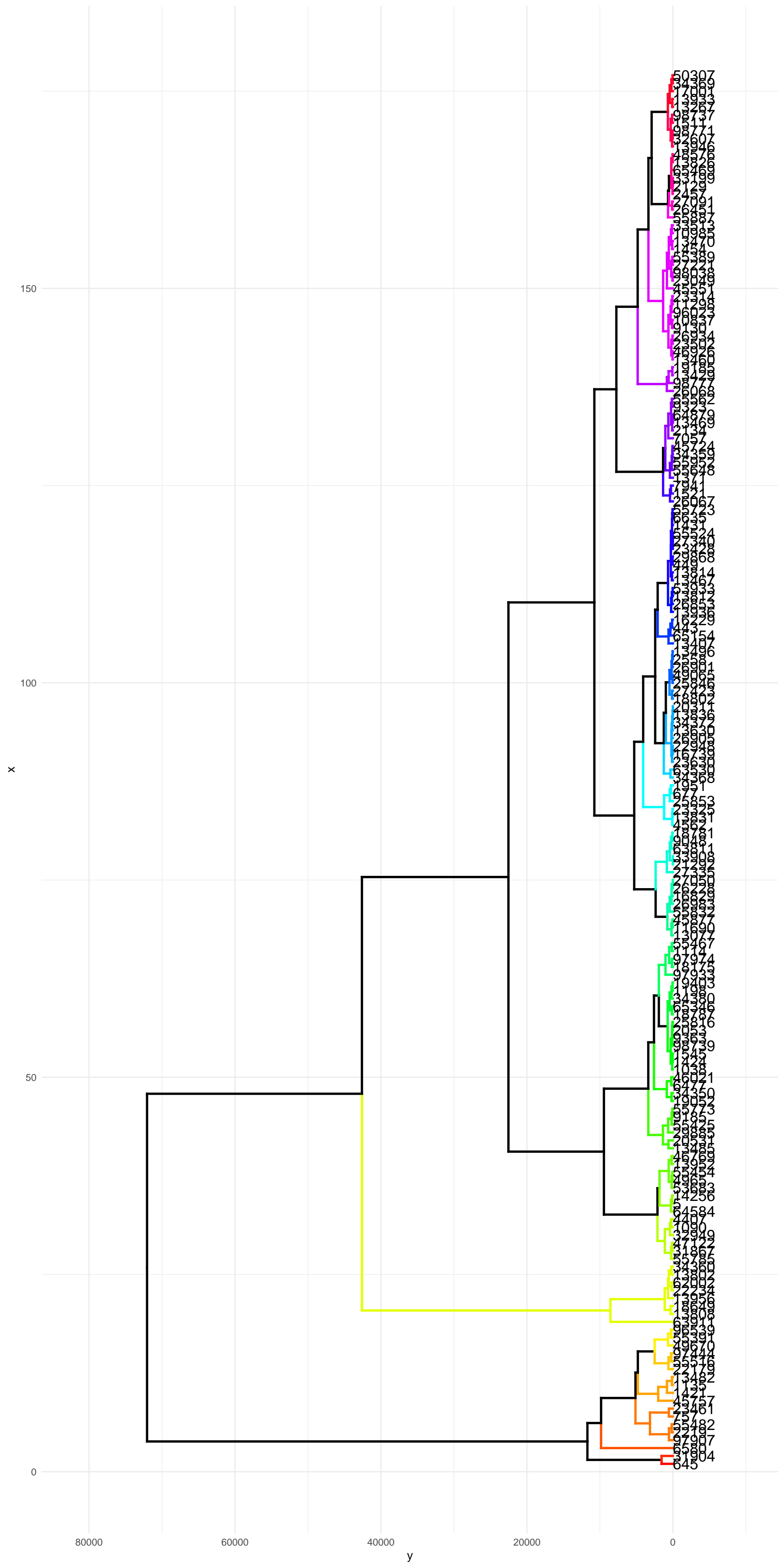

Supplement: Supplementary file 1 [file sensors-22-00001-s001.zip › sensors-1463895-supplementary/OverallTB/EnsembleCut/NoisePen_NP_Dendrogram.pdf]

Noise-Penalized Ensemble Cut Encoding: Noise-Penalized Distance

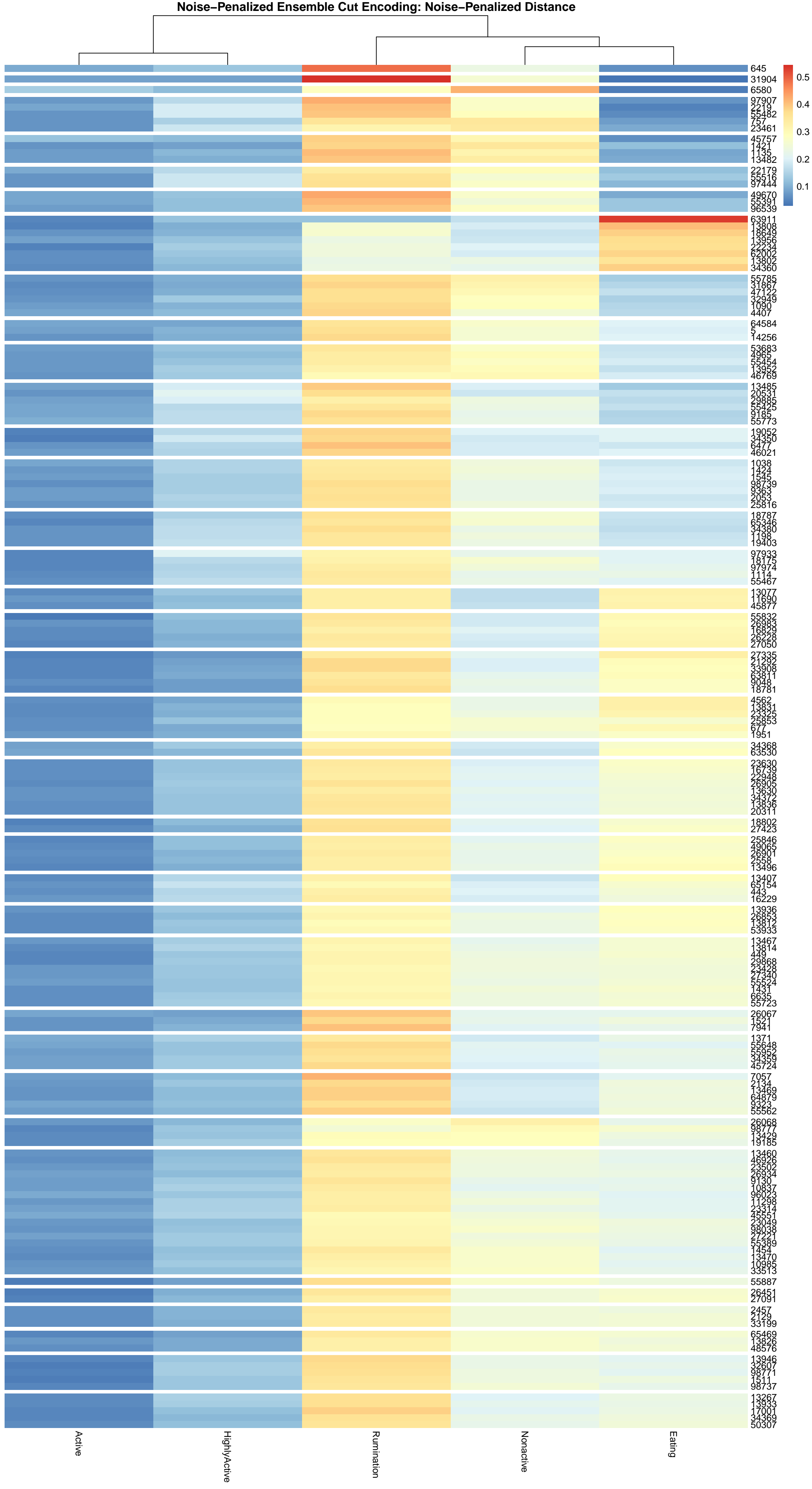

Supplement: Supplementary file 1 [file sensors-22-00001-s001.zip › sensors-1463895-supplementary/OverallTB/EnsembleCut/NoisePen_NP_Heatmap.pdf]

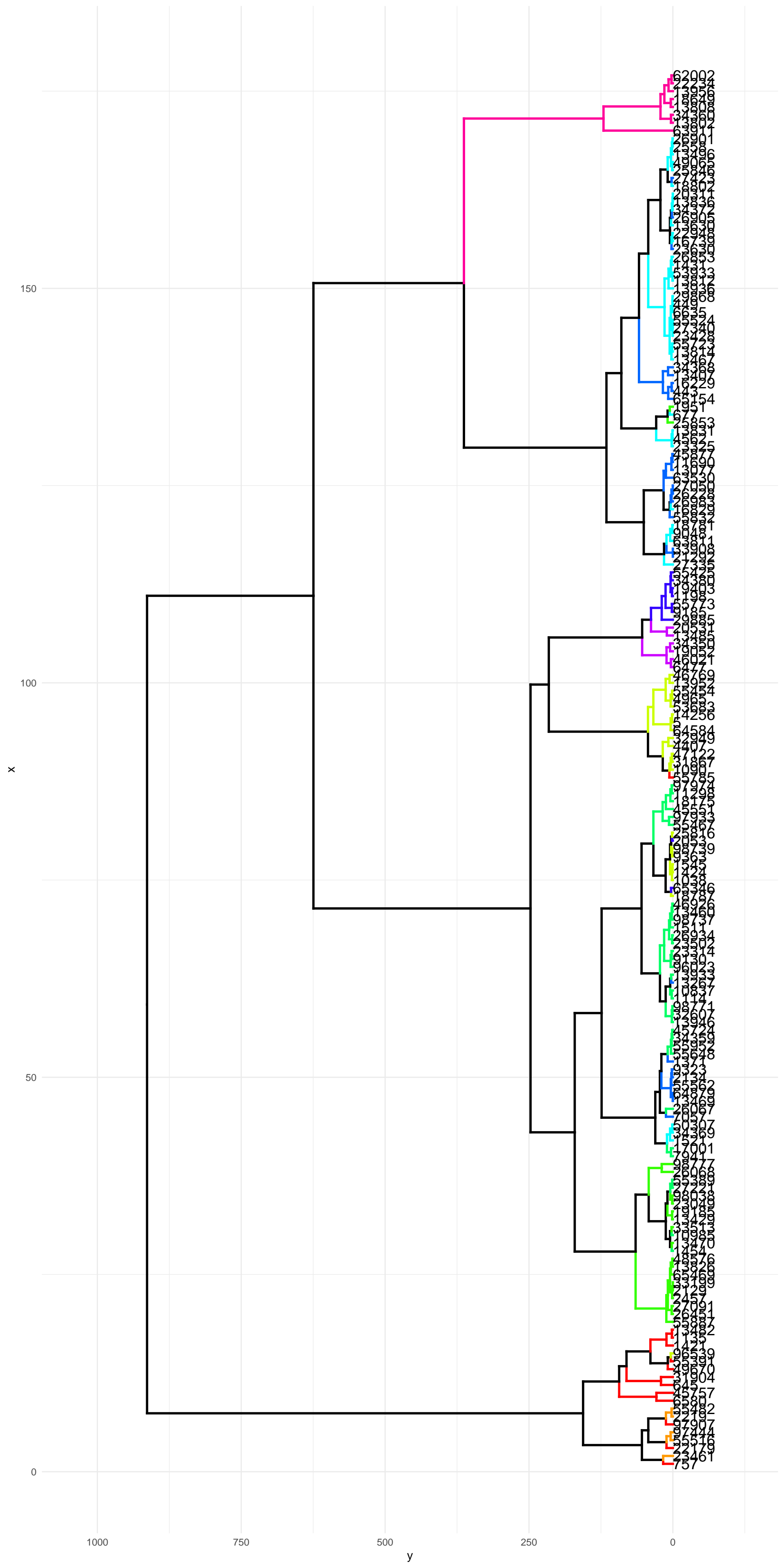

Supplement: Supplementary file 1 [file sensors-22-00001-s001.zip › sensors-1463895-supplementary/OverallTB/EnsembleCut/NoisePen_PP_Dendrogram.pdf]

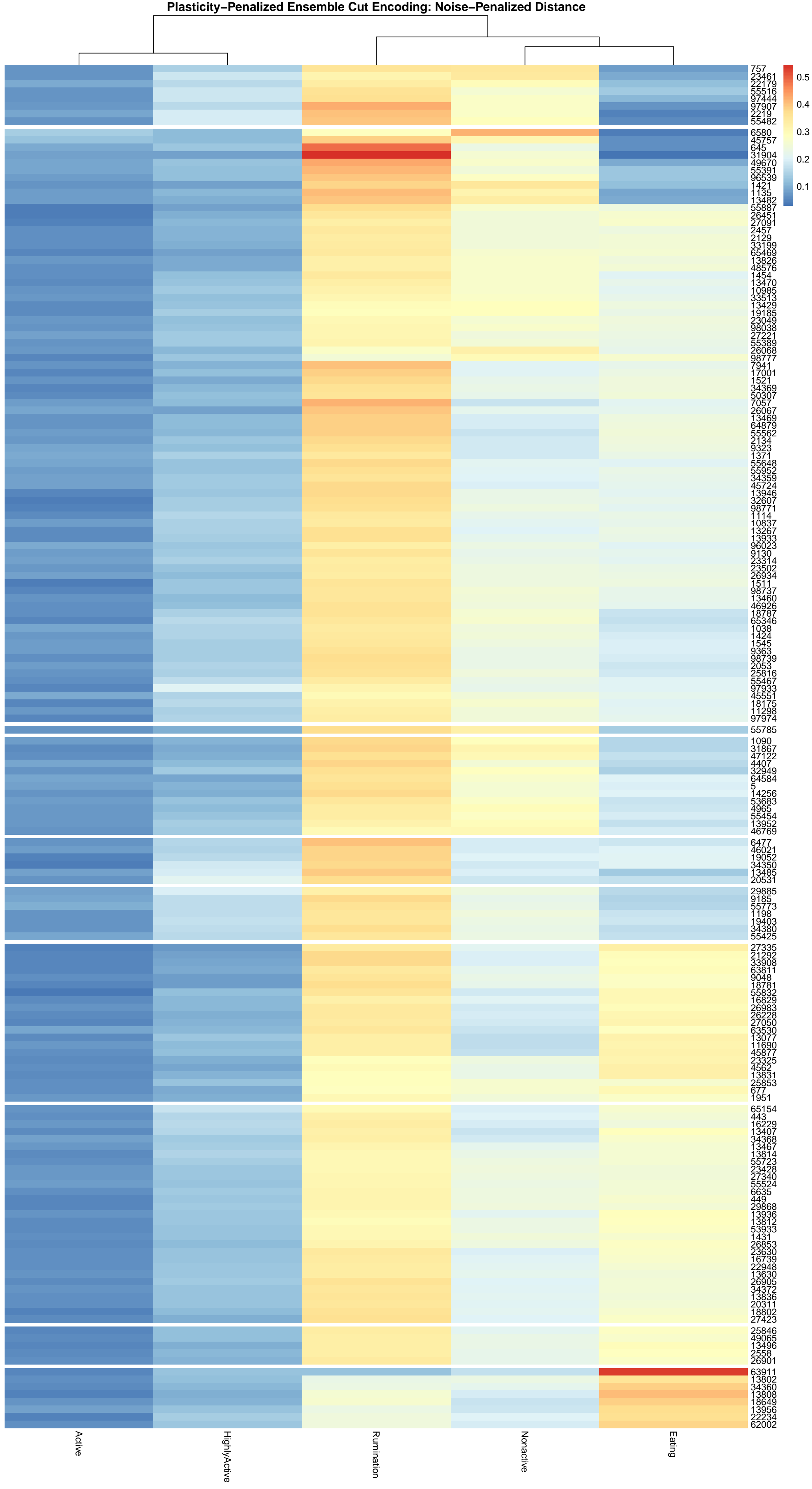

Supplement: Supplementary file 1 [file sensors-22-00001-s001.zip › sensors-1463895-supplementary/OverallTB/EnsembleCut/NoisePen_PP_Heatmap.pdf]

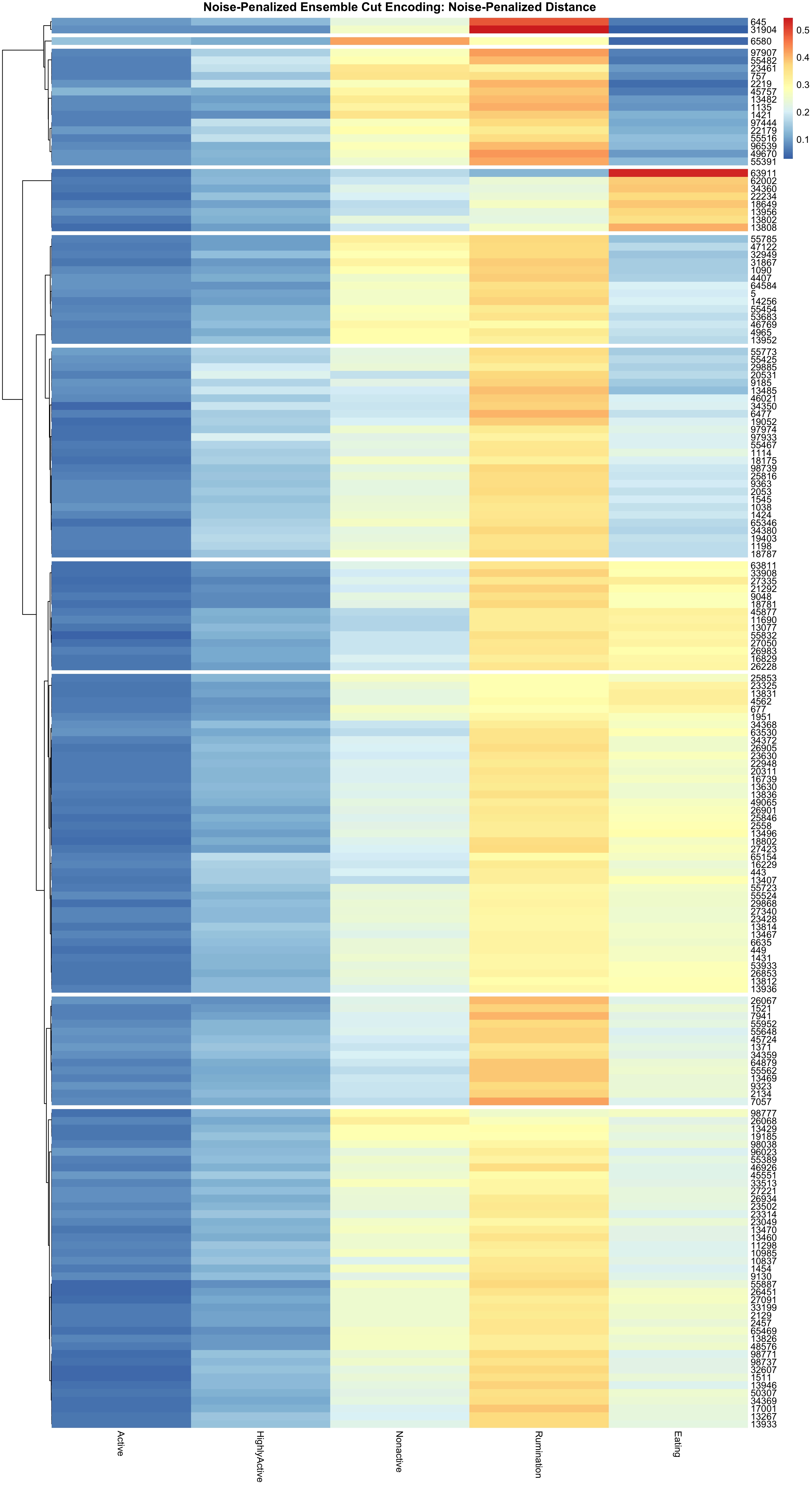

Supplement: Supplementary file 1 [file sensors-22-00001-s001.zip › sensors-1463895-supplementary/OverallTB/EnsembleCut/NPEncode/NPCut/NPEncode_R10_C0.jpeg]

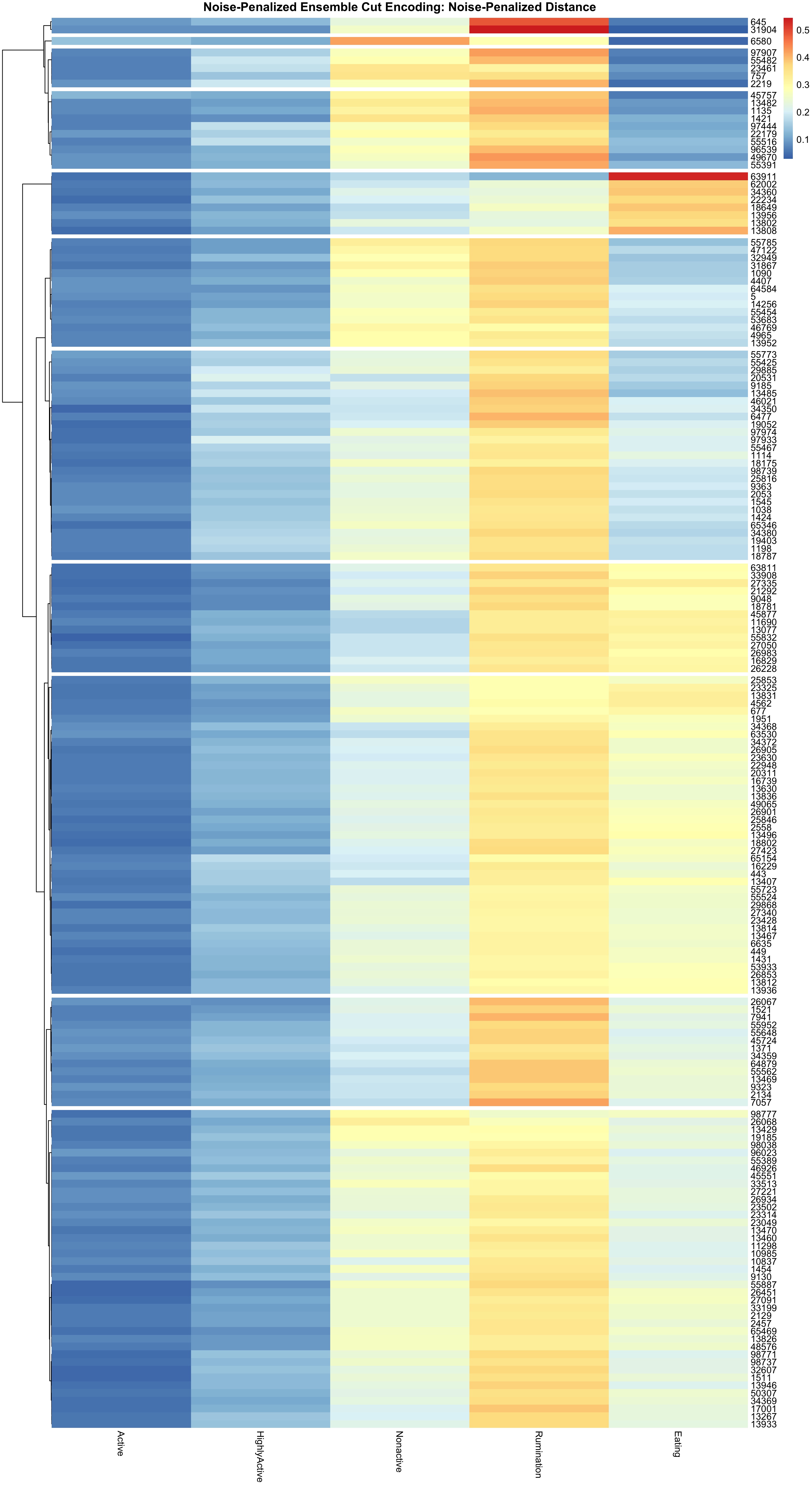

Supplement: Supplementary file 1 [file sensors-22-00001-s001.zip › sensors-1463895-supplementary/OverallTB/EnsembleCut/NPEncode/NPCut/NPEncode_R11_C0.jpeg]

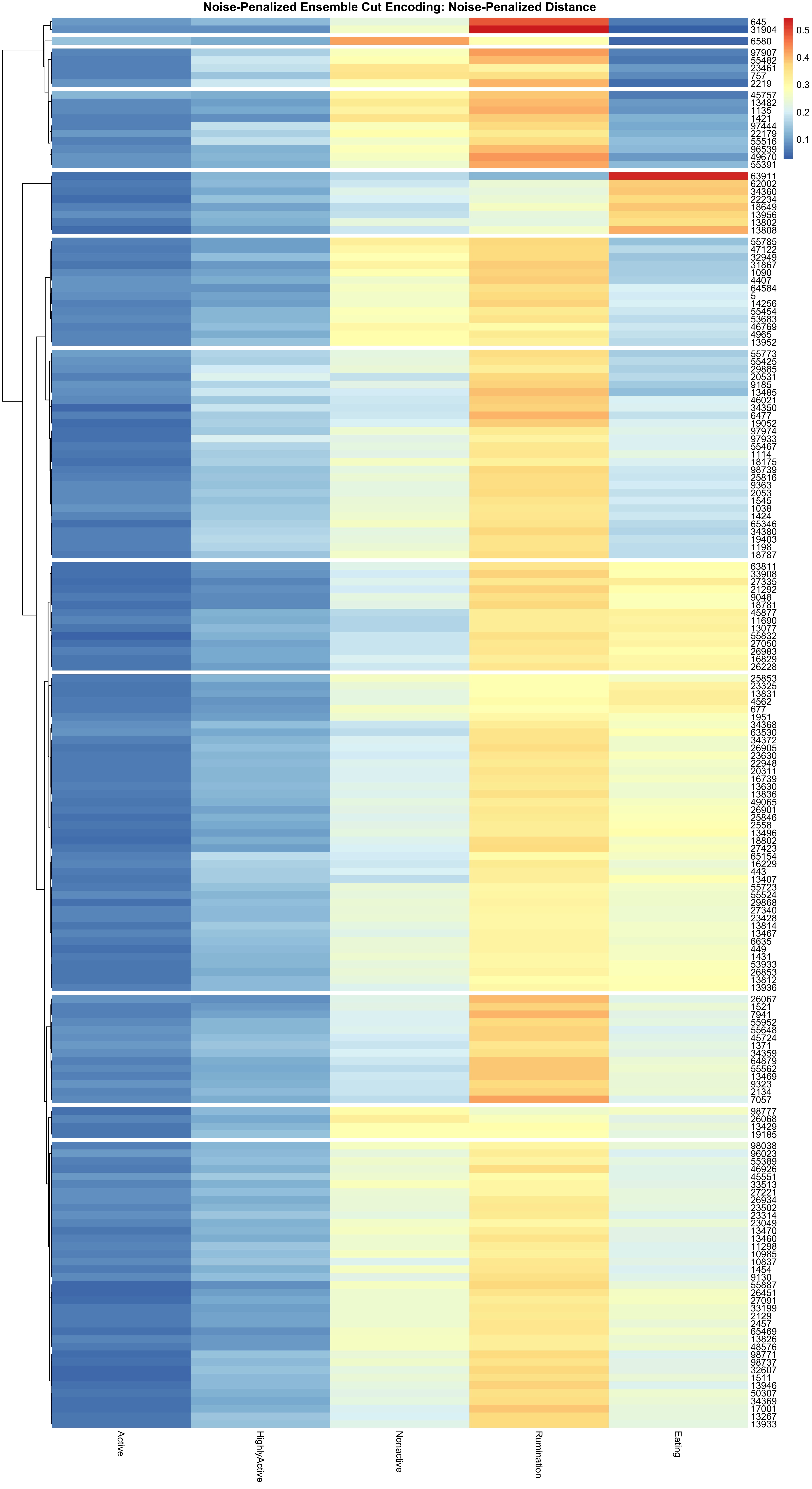

Supplement: Supplementary file 1 [file sensors-22-00001-s001.zip › sensors-1463895-supplementary/OverallTB/EnsembleCut/NPEncode/NPCut/NPEncode_R12_C0.jpeg]

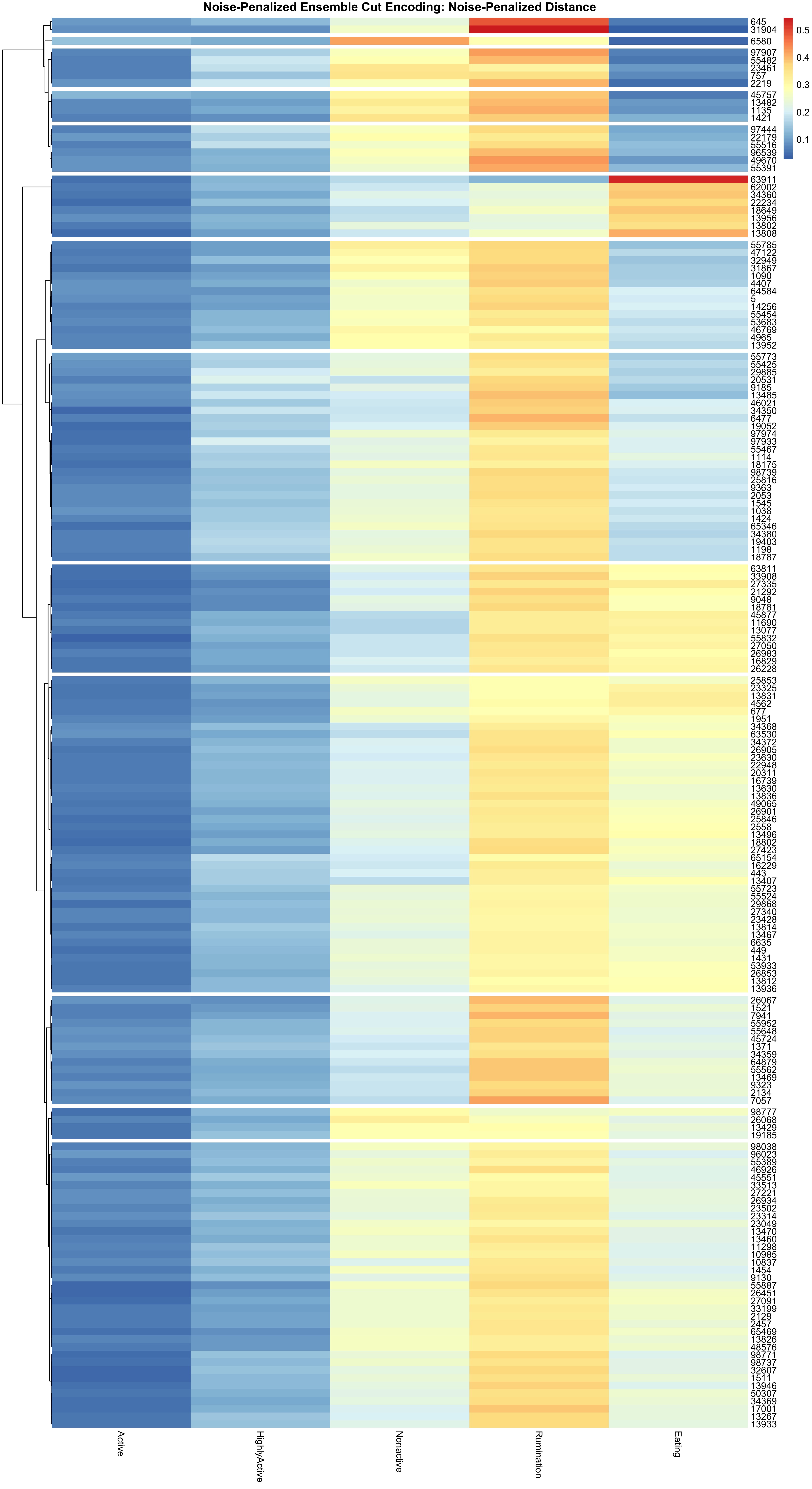

Supplement: Supplementary file 1 [file sensors-22-00001-s001.zip › sensors-1463895-supplementary/OverallTB/EnsembleCut/NPEncode/NPCut/NPEncode_R13_C0.jpeg]

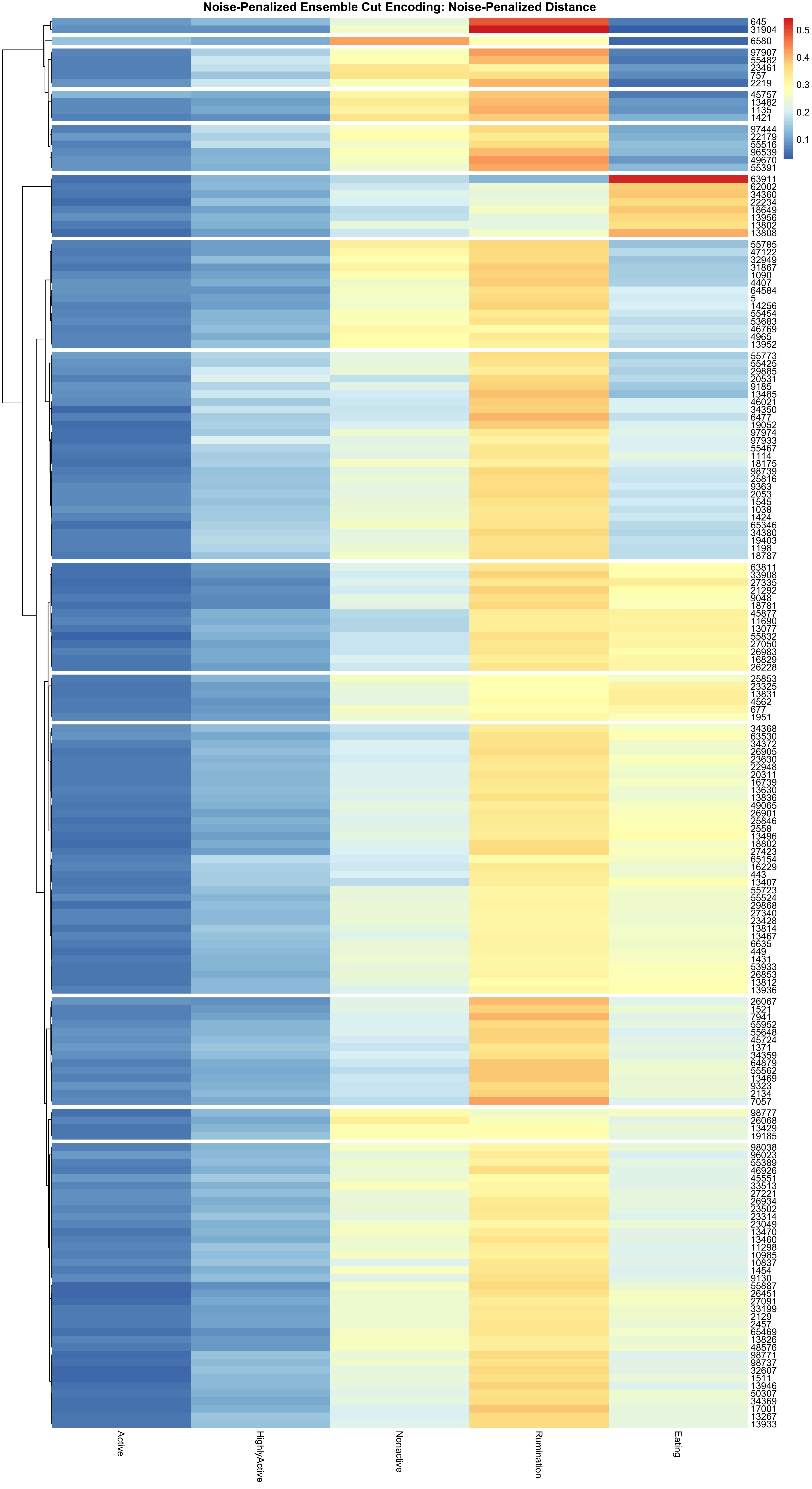

Supplement: Supplementary file 1 [file sensors-22-00001-s001.zip › sensors-1463895-supplementary/OverallTB/EnsembleCut/NPEncode/NPCut/NPEncode_R14_C0.jpeg]

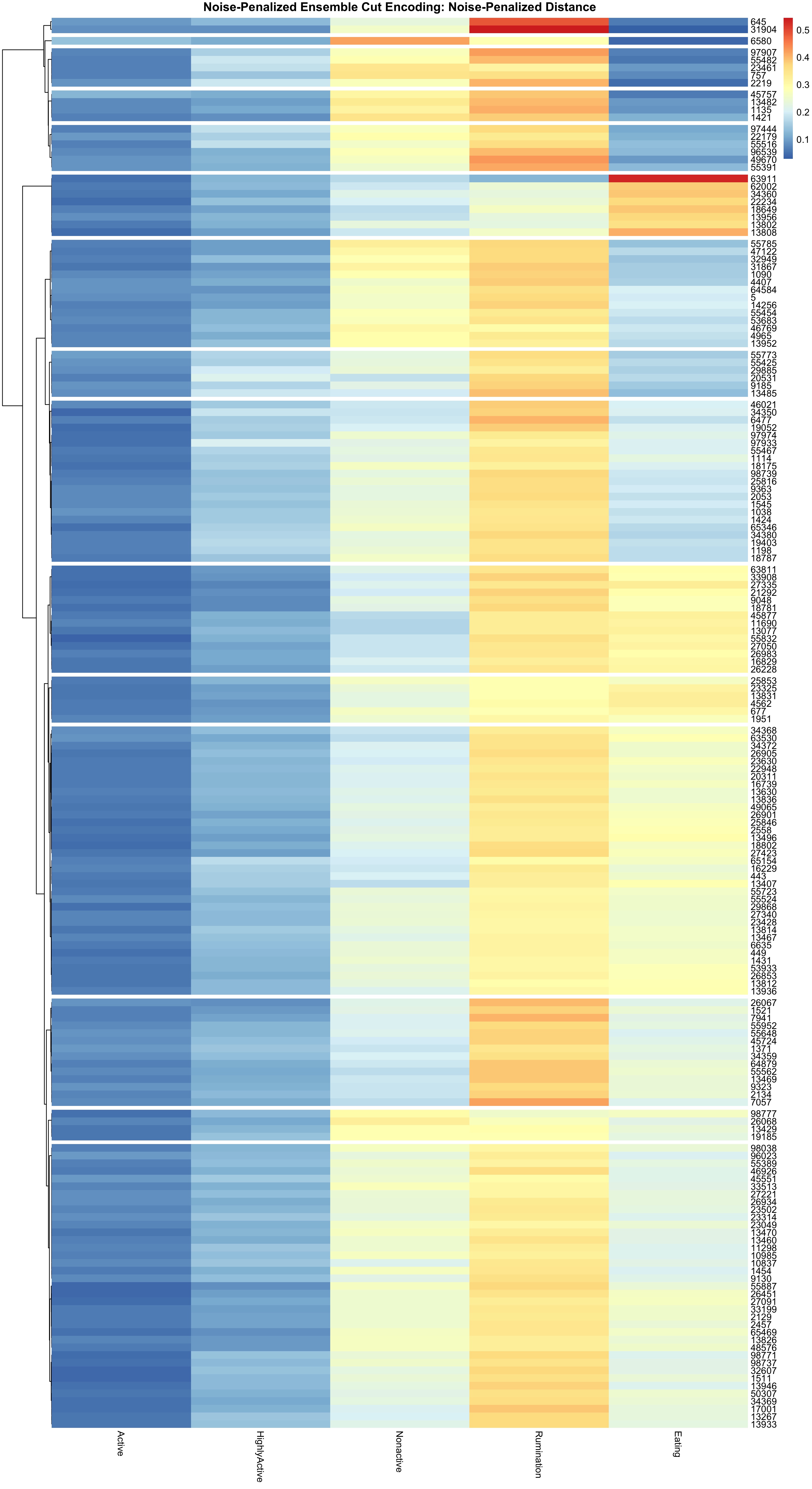

Supplement: Supplementary file 1 [file sensors-22-00001-s001.zip › sensors-1463895-supplementary/OverallTB/EnsembleCut/NPEncode/NPCut/NPEncode_R15_C0.jpeg]

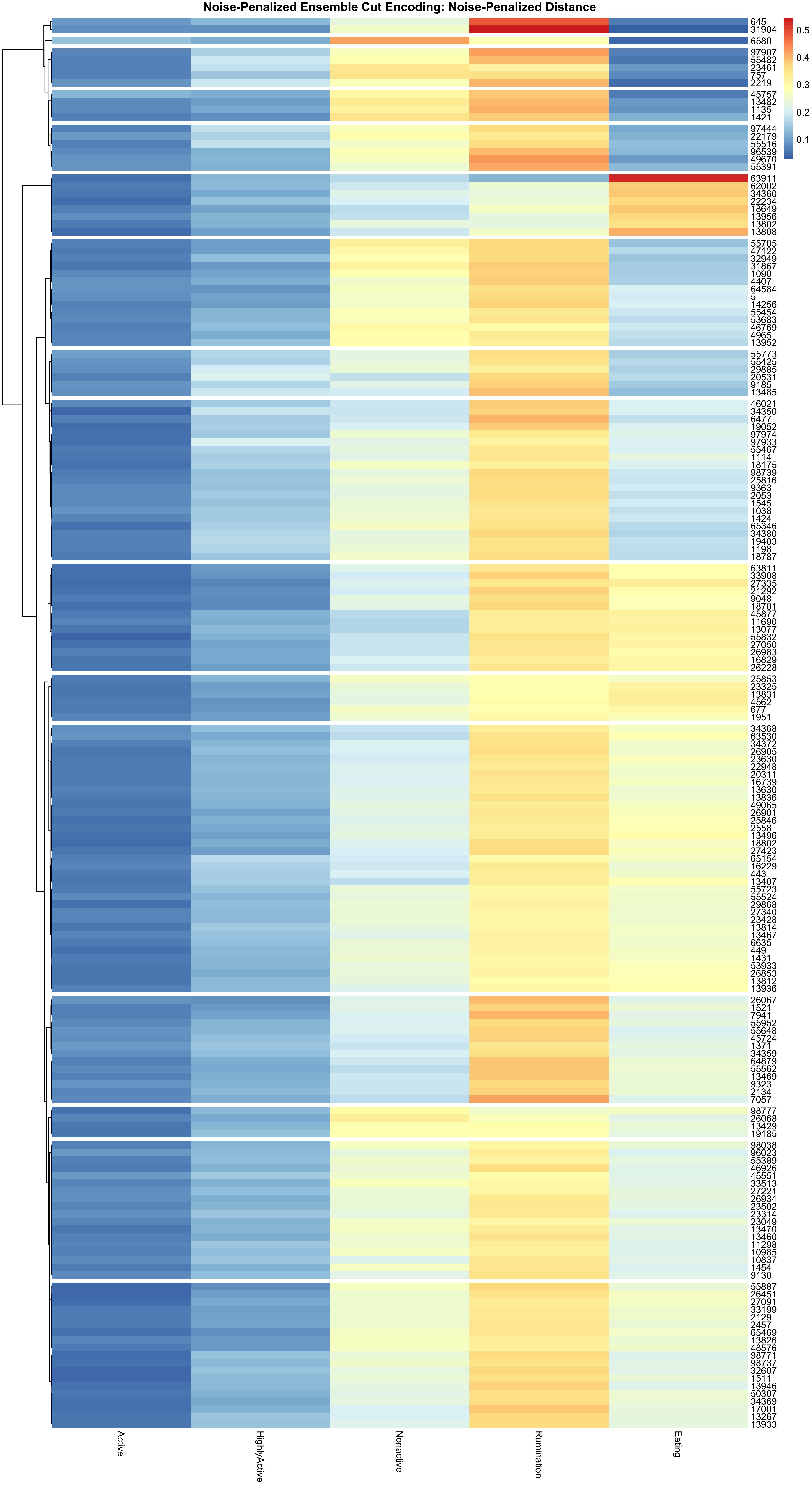

Supplement: Supplementary file 1 [file sensors-22-00001-s001.zip › sensors-1463895-supplementary/OverallTB/EnsembleCut/NPEncode/NPCut/NPEncode_R16_C0.jpeg]

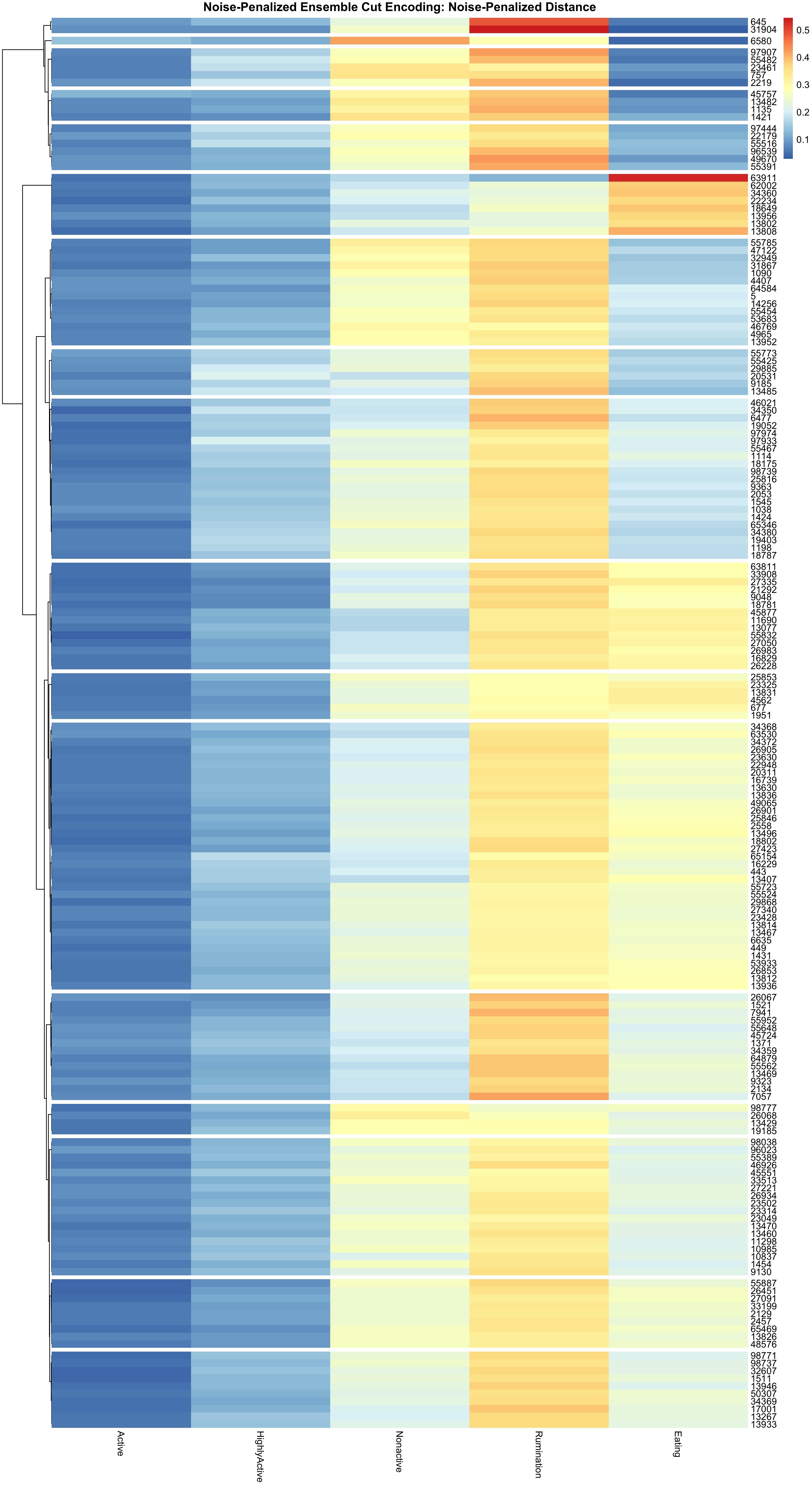

Supplement: Supplementary file 1 [file sensors-22-00001-s001.zip › sensors-1463895-supplementary/OverallTB/EnsembleCut/NPEncode/NPCut/NPEncode_R17_C0.jpeg]

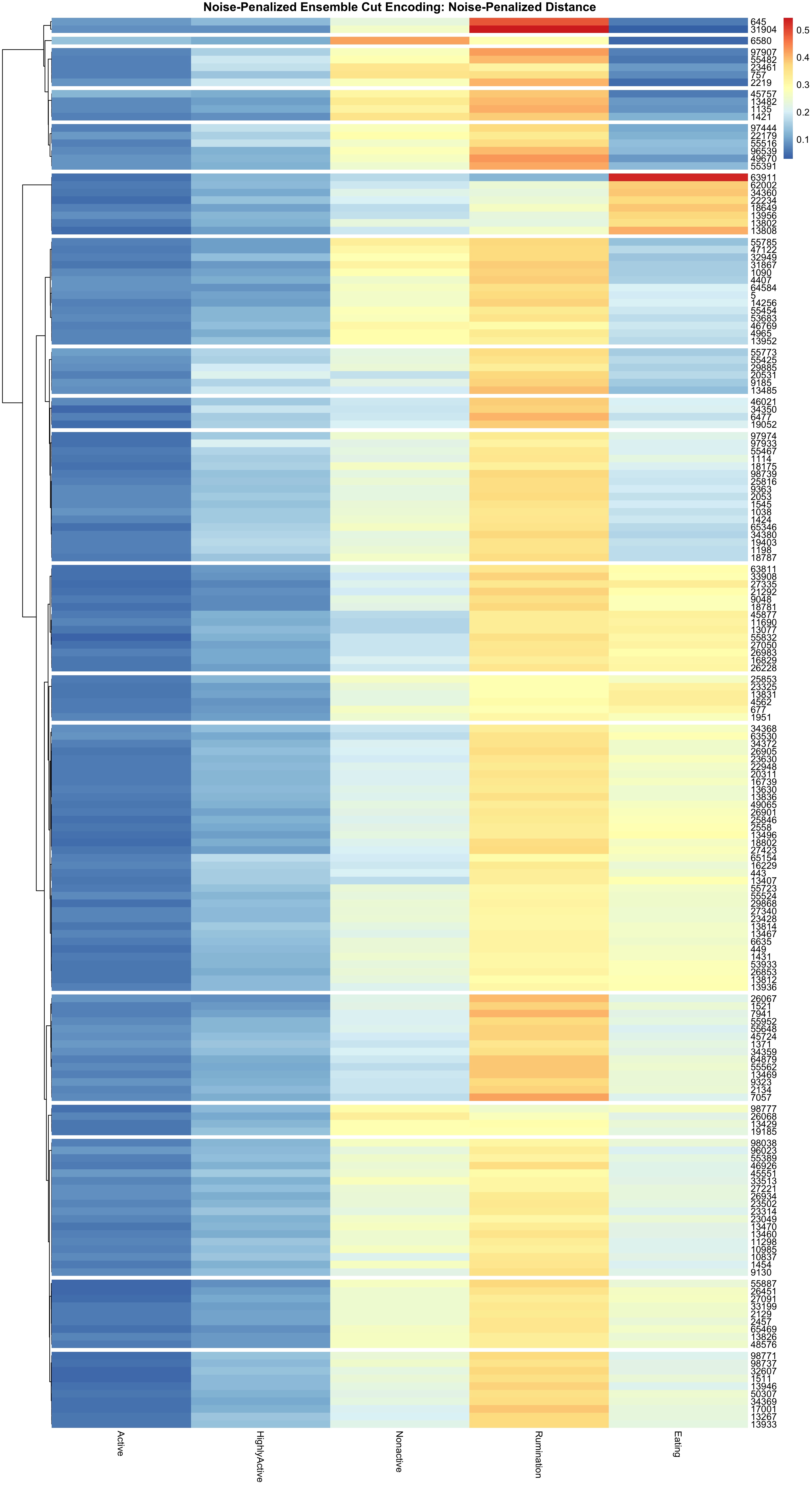

Supplement: Supplementary file 1 [file sensors-22-00001-s001.zip › sensors-1463895-supplementary/OverallTB/EnsembleCut/NPEncode/NPCut/NPEncode_R18_C0.jpeg]

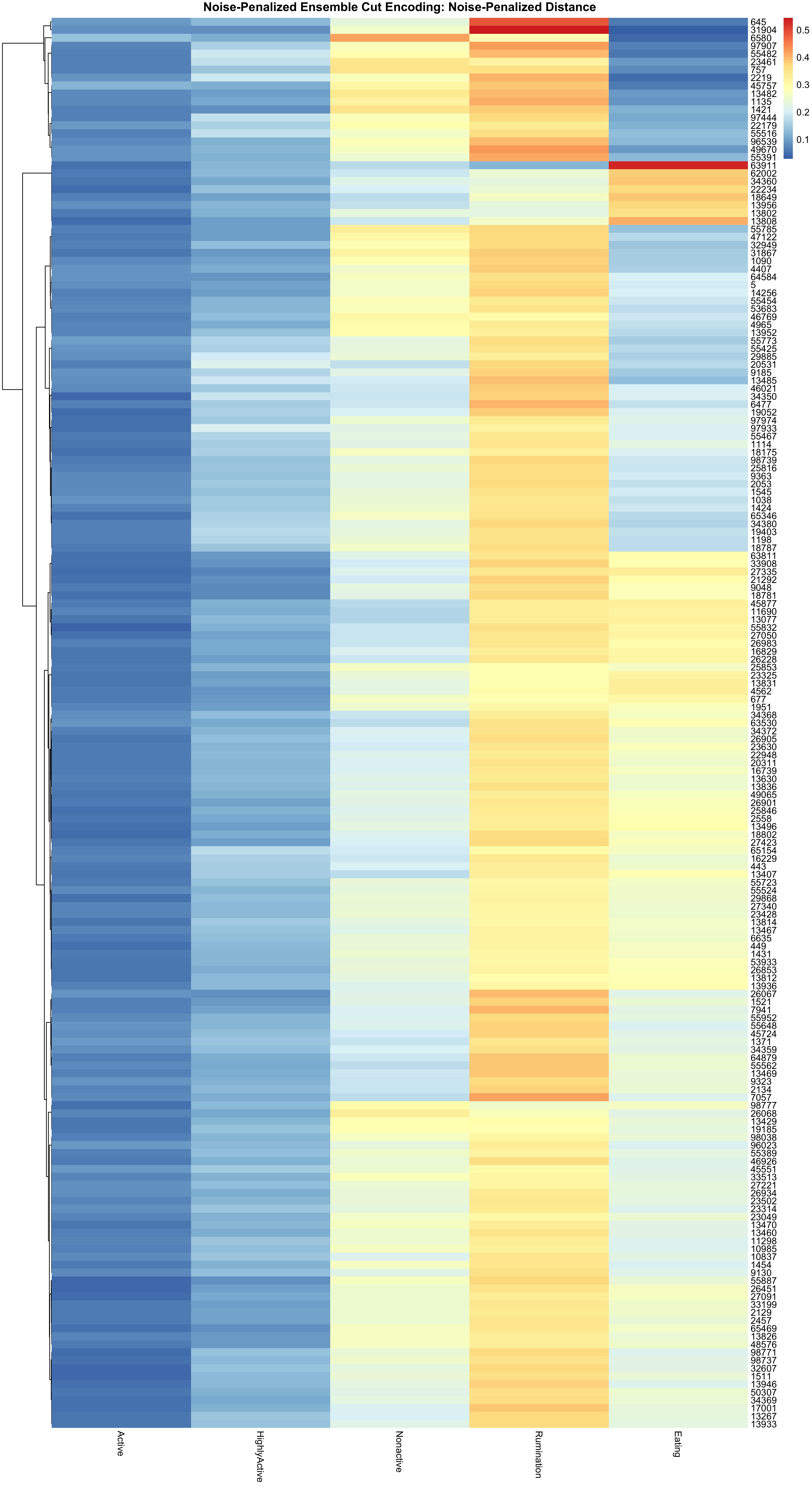

Supplement: Supplementary file 1 [file sensors-22-00001-s001.zip › sensors-1463895-supplementary/OverallTB/EnsembleCut/NPEncode/NPCut/NPEncode_R1_C0.jpeg]

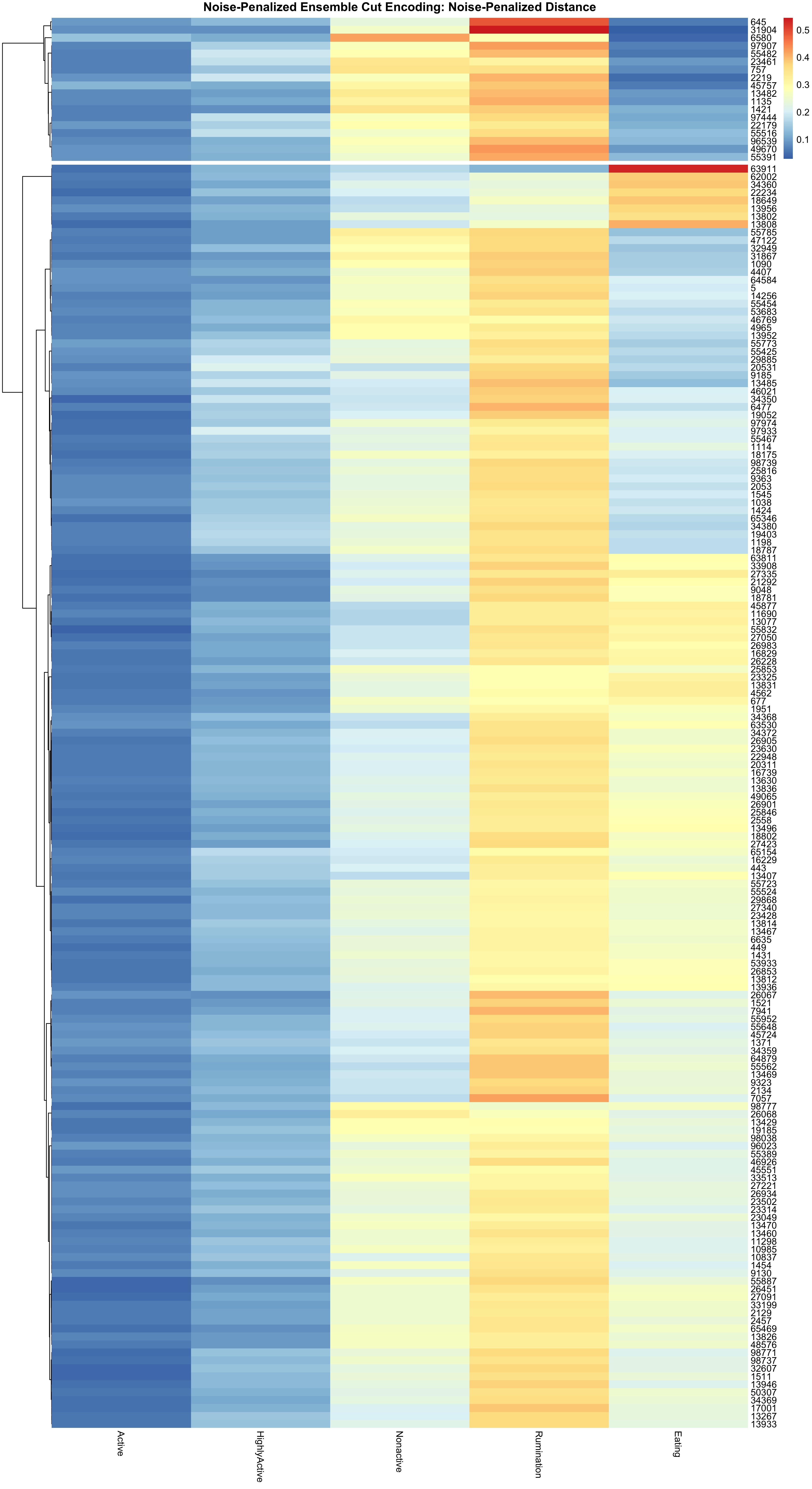

Supplement: Supplementary file 1 [file sensors-22-00001-s001.zip › sensors-1463895-supplementary/OverallTB/EnsembleCut/NPEncode/NPCut/NPEncode_R2_C0.jpeg]

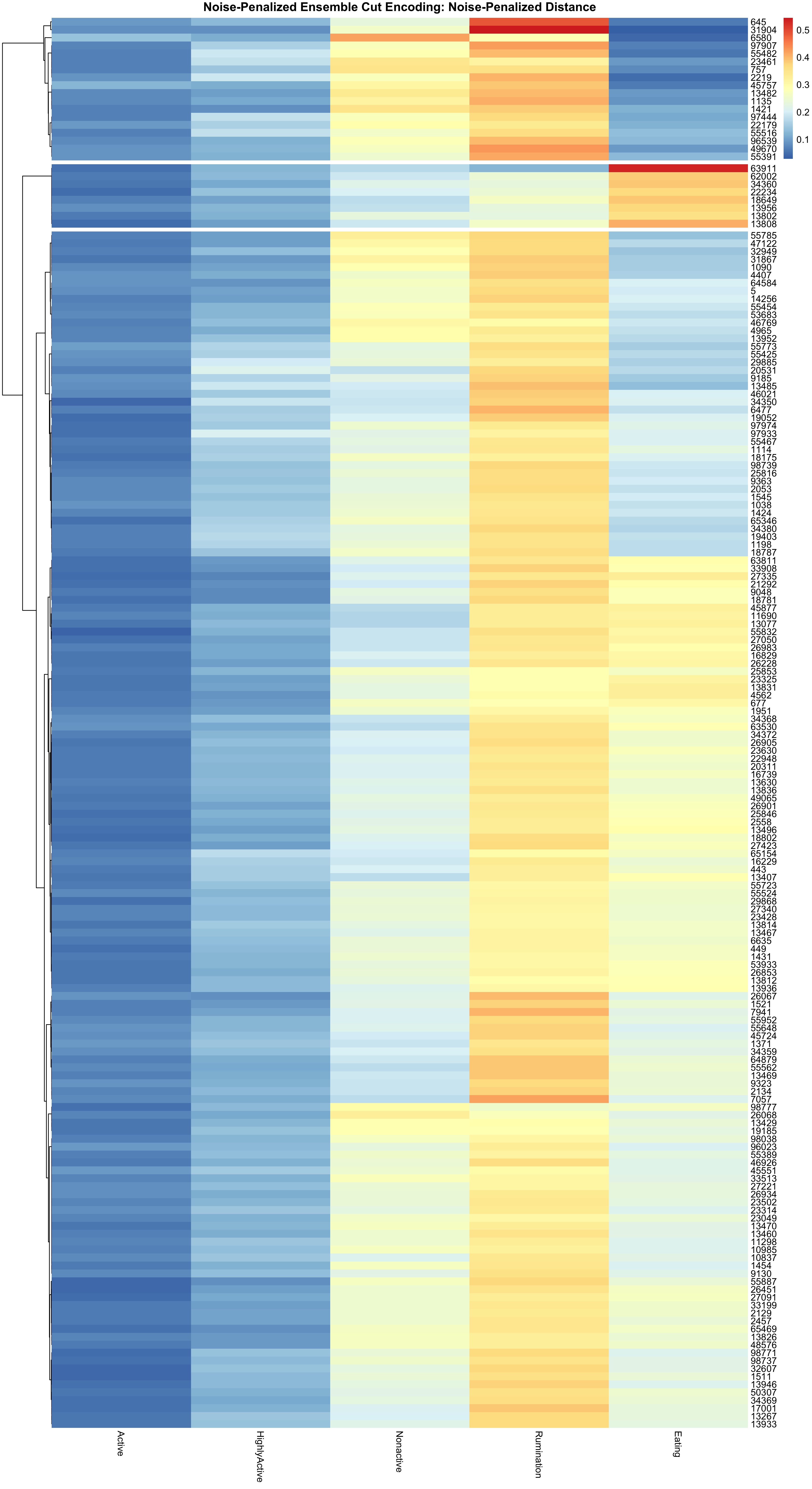

Supplement: Supplementary file 1 [file sensors-22-00001-s001.zip › sensors-1463895-supplementary/OverallTB/EnsembleCut/NPEncode/NPCut/NPEncode_R3_C0.jpeg]

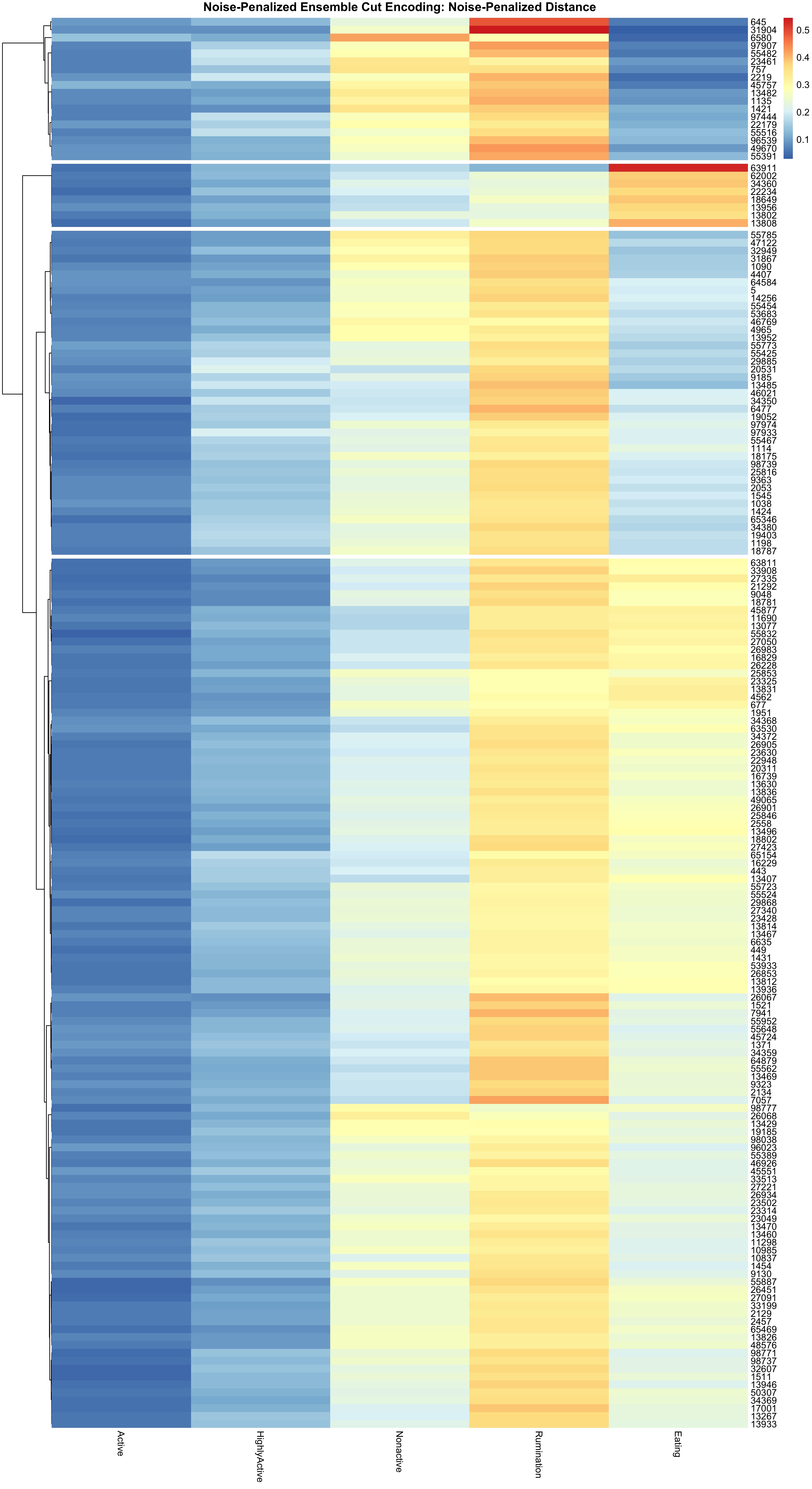

Supplement: Supplementary file 1 [file sensors-22-00001-s001.zip › sensors-1463895-supplementary/OverallTB/EnsembleCut/NPEncode/NPCut/NPEncode_R4_C0.jpeg]

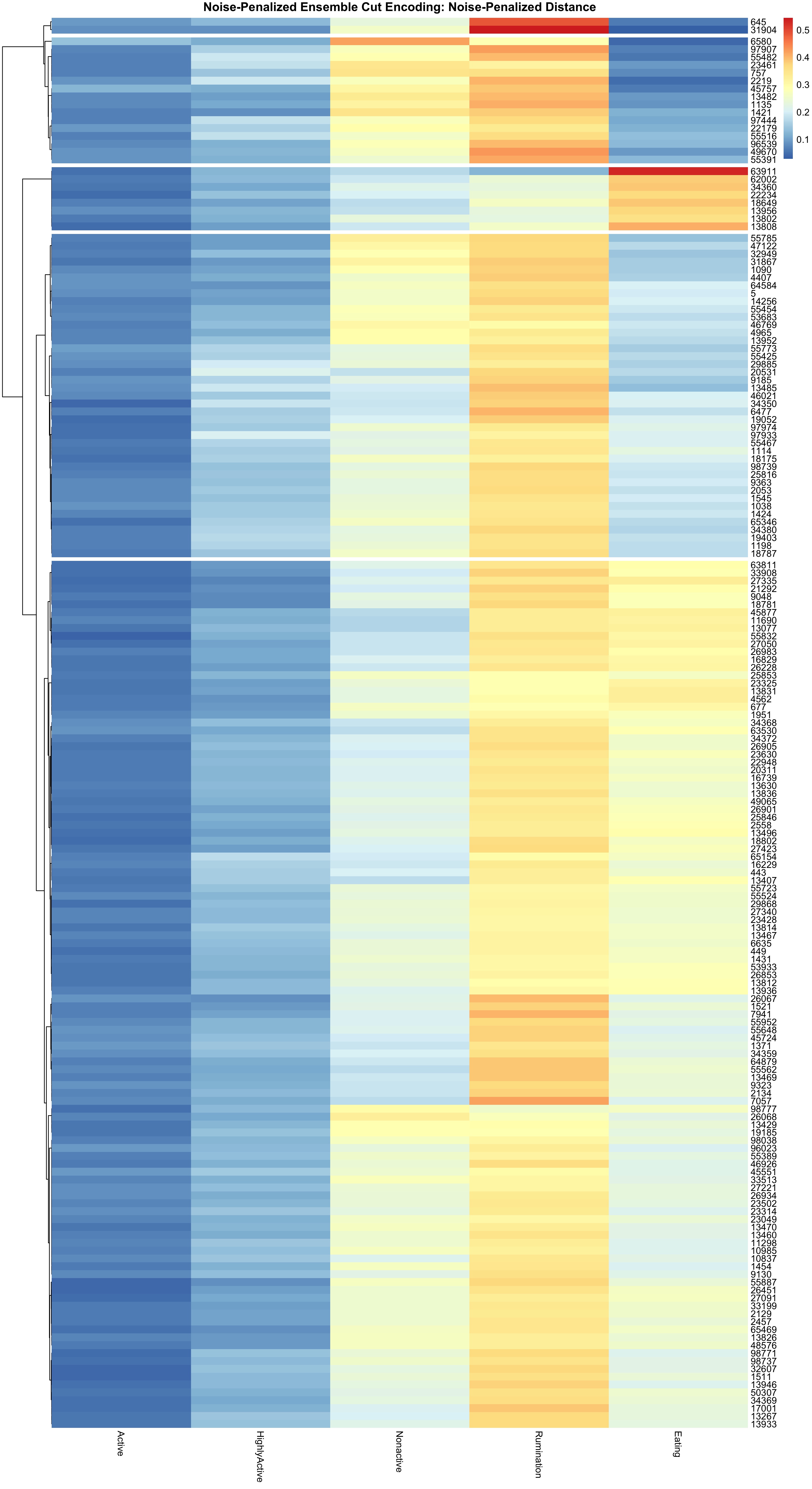

Supplement: Supplementary file 1 [file sensors-22-00001-s001.zip › sensors-1463895-supplementary/OverallTB/EnsembleCut/NPEncode/NPCut/NPEncode_R5_C0.jpeg]

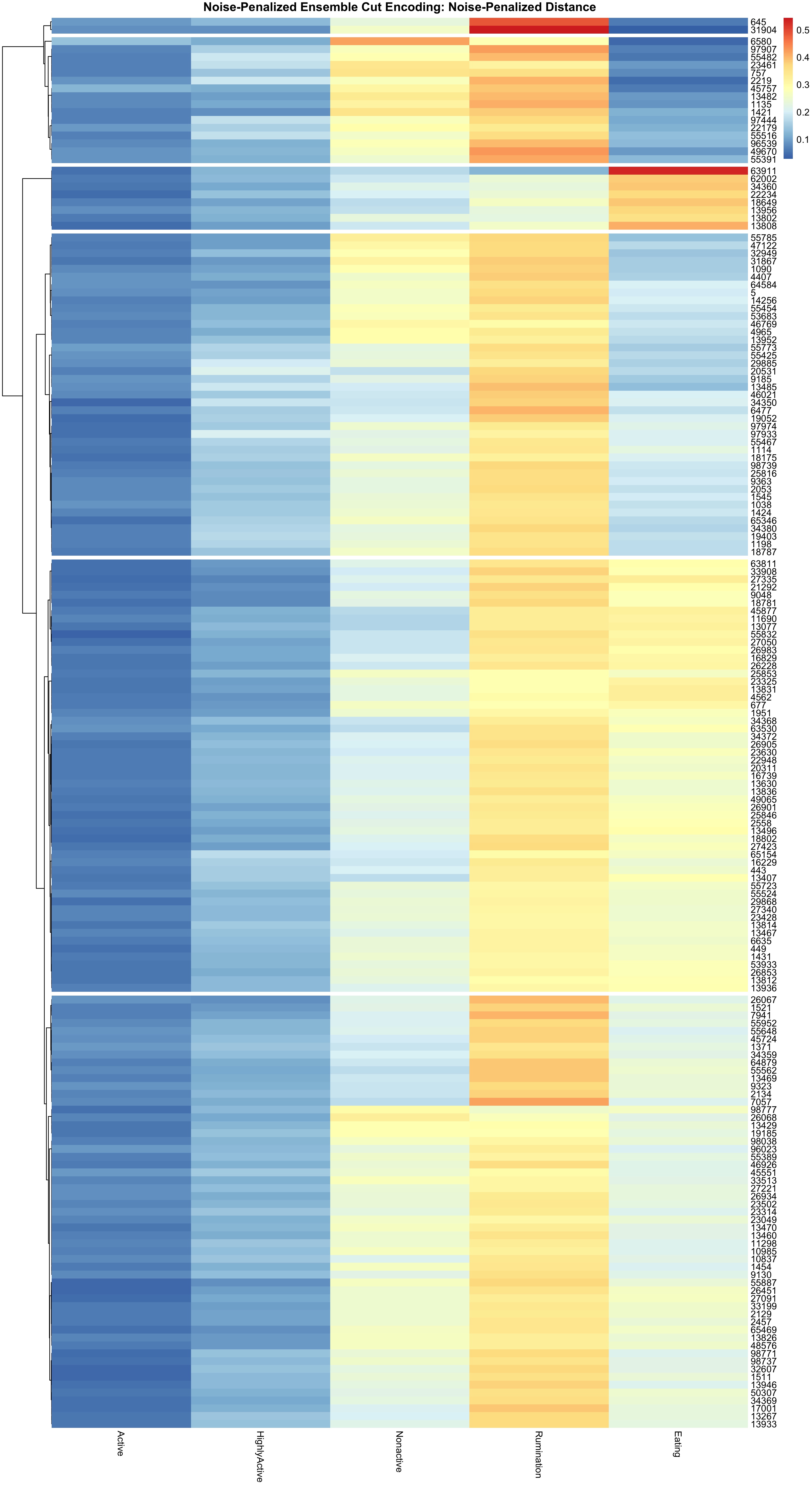

Supplement: Supplementary file 1 [file sensors-22-00001-s001.zip › sensors-1463895-supplementary/OverallTB/EnsembleCut/NPEncode/NPCut/NPEncode_R6_C0.jpeg]

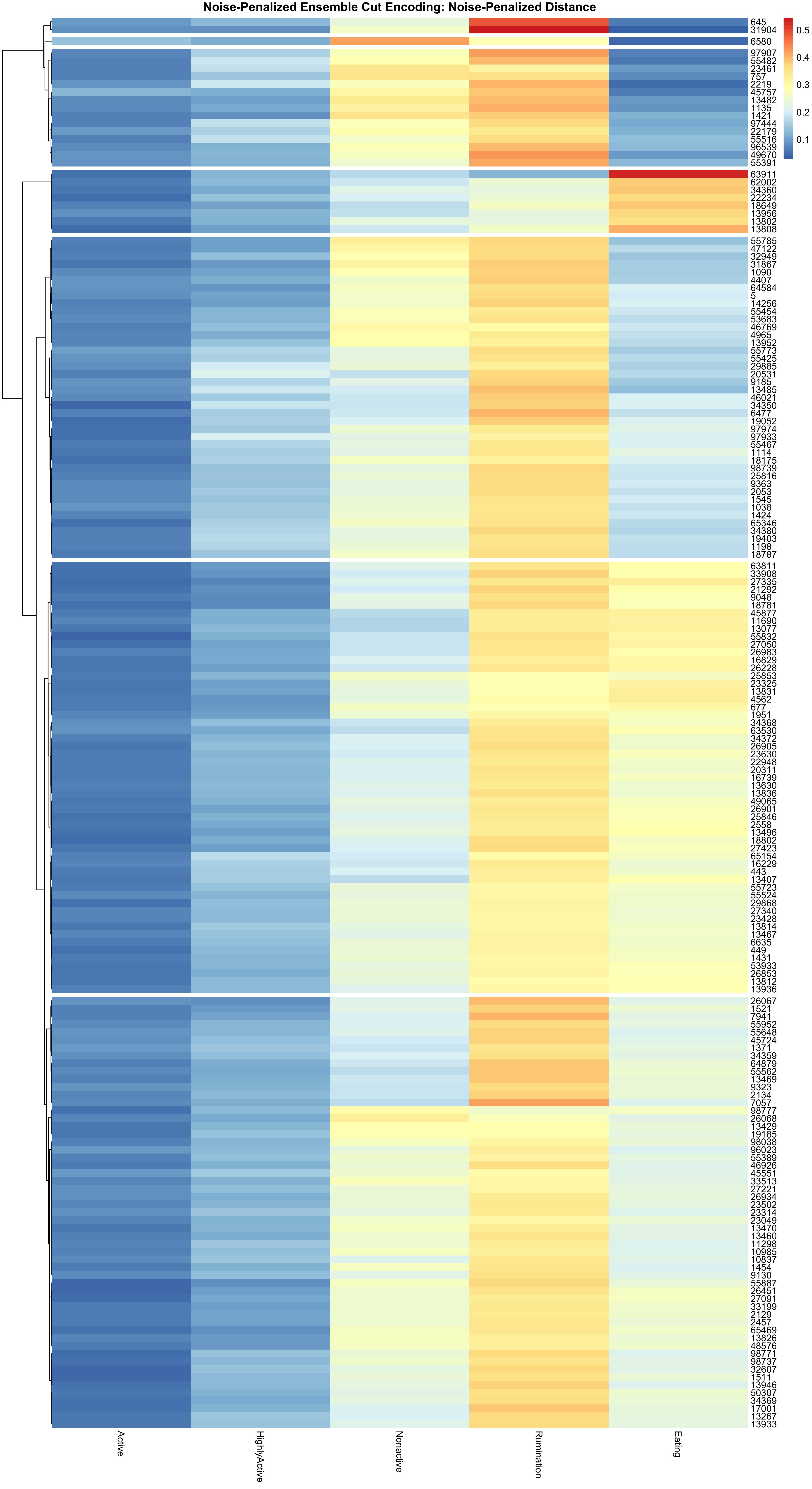

Supplement: Supplementary file 1 [file sensors-22-00001-s001.zip › sensors-1463895-supplementary/OverallTB/EnsembleCut/NPEncode/NPCut/NPEncode_R7_C0.jpeg]

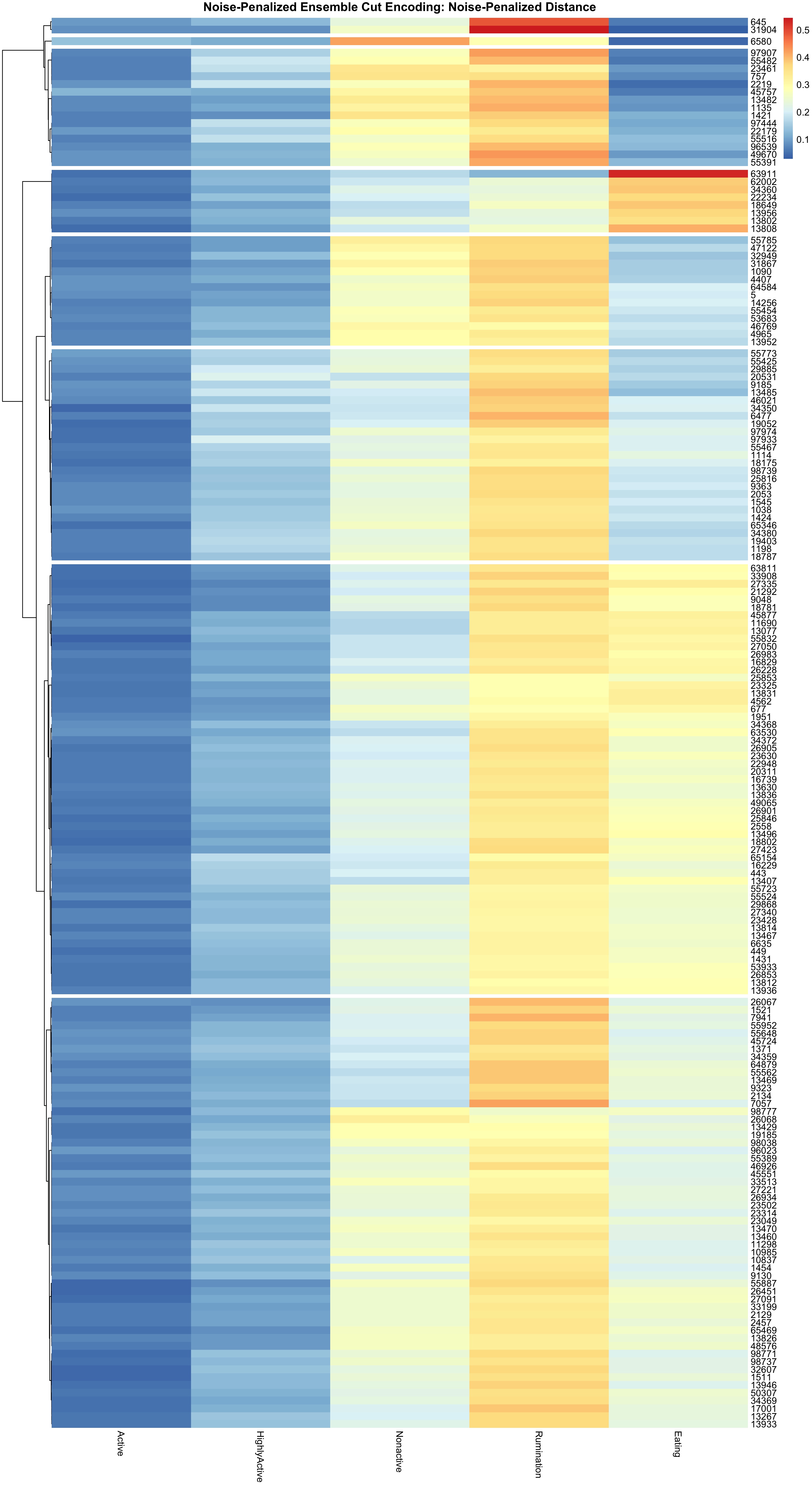

Supplement: Supplementary file 1 [file sensors-22-00001-s001.zip › sensors-1463895-supplementary/OverallTB/EnsembleCut/NPEncode/NPCut/NPEncode_R8_C0.jpeg]

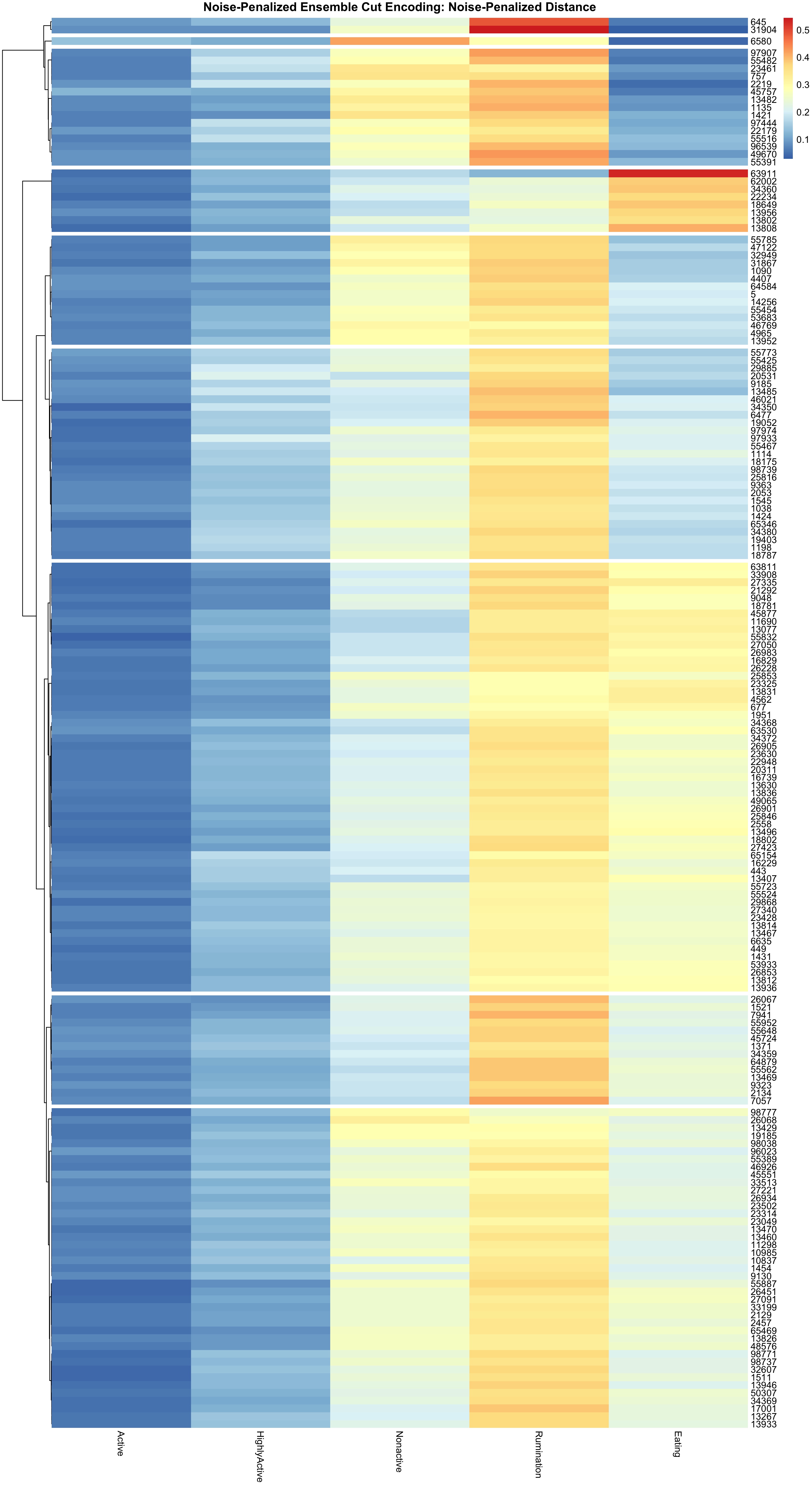

Supplement: Supplementary file 1 [file sensors-22-00001-s001.zip › sensors-1463895-supplementary/OverallTB/EnsembleCut/NPEncode/NPCut/NPEncode_R9_C0.jpeg]

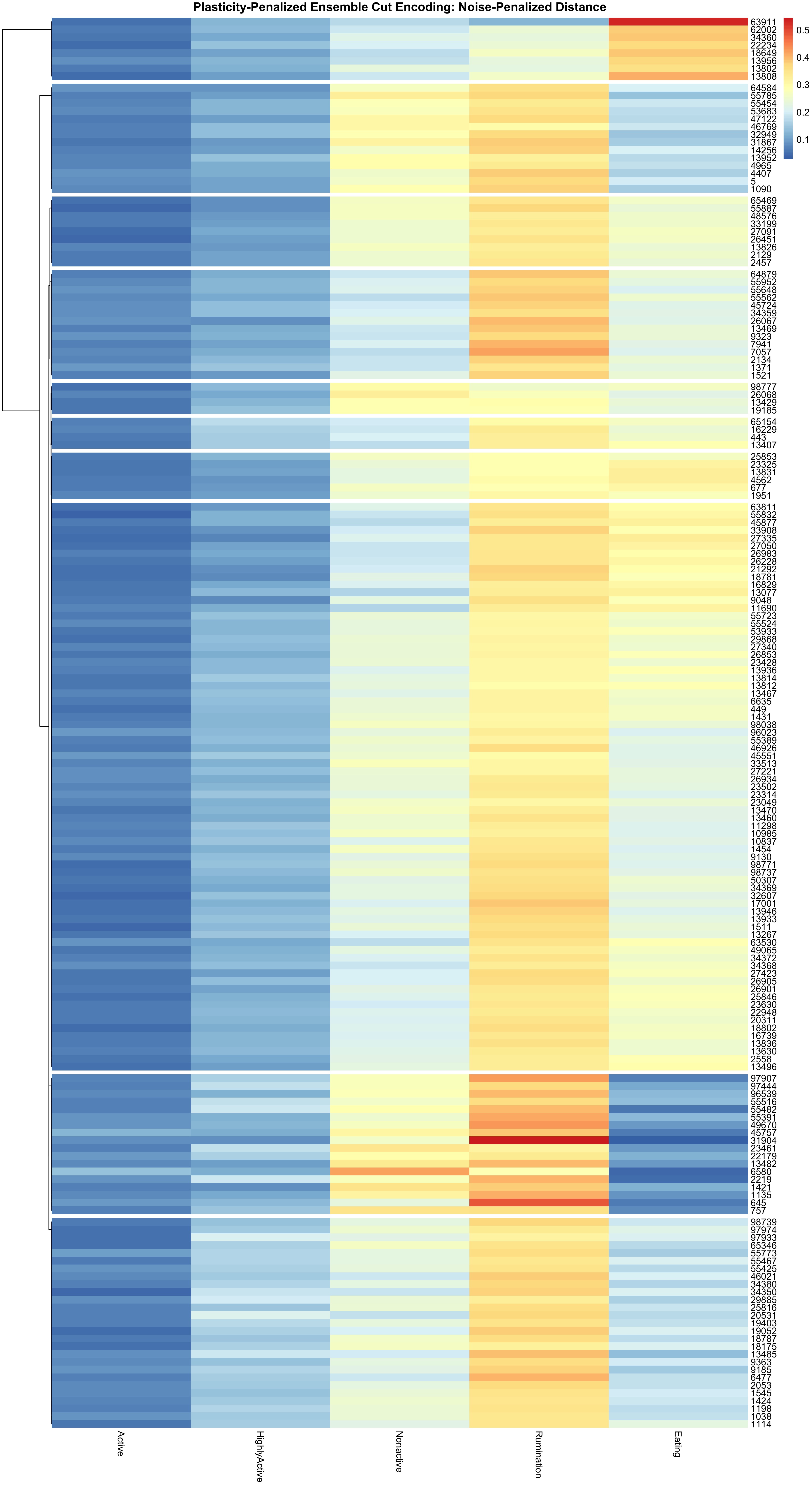

Supplement: Supplementary file 1 [file sensors-22-00001-s001.zip › sensors-1463895-supplementary/OverallTB/EnsembleCut/NPEncode/PPCut/NPEncode_R10_C0.jpeg]

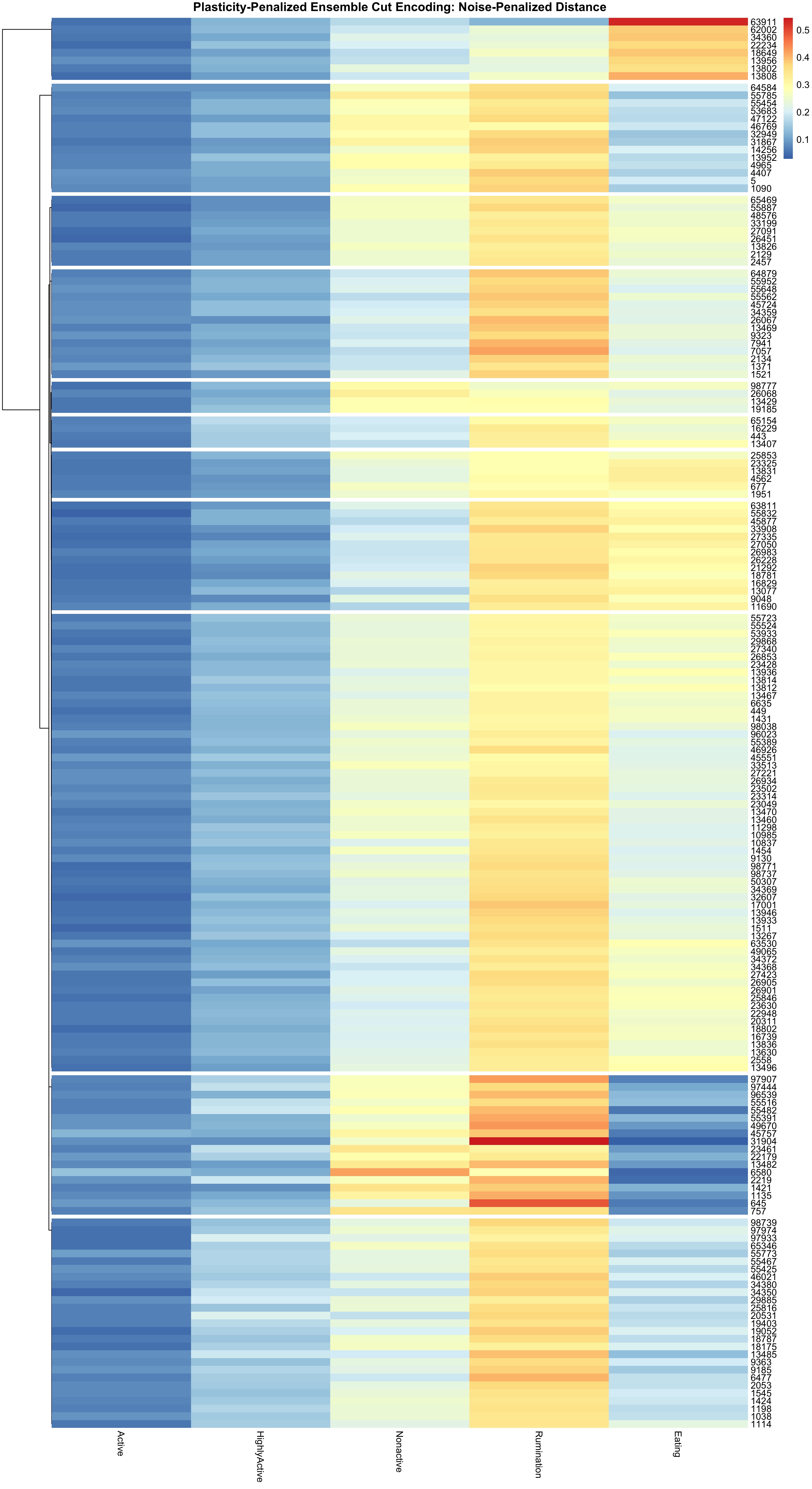

Supplement: Supplementary file 1 [file sensors-22-00001-s001.zip › sensors-1463895-supplementary/OverallTB/EnsembleCut/NPEncode/PPCut/NPEncode_R11_C0.jpeg]

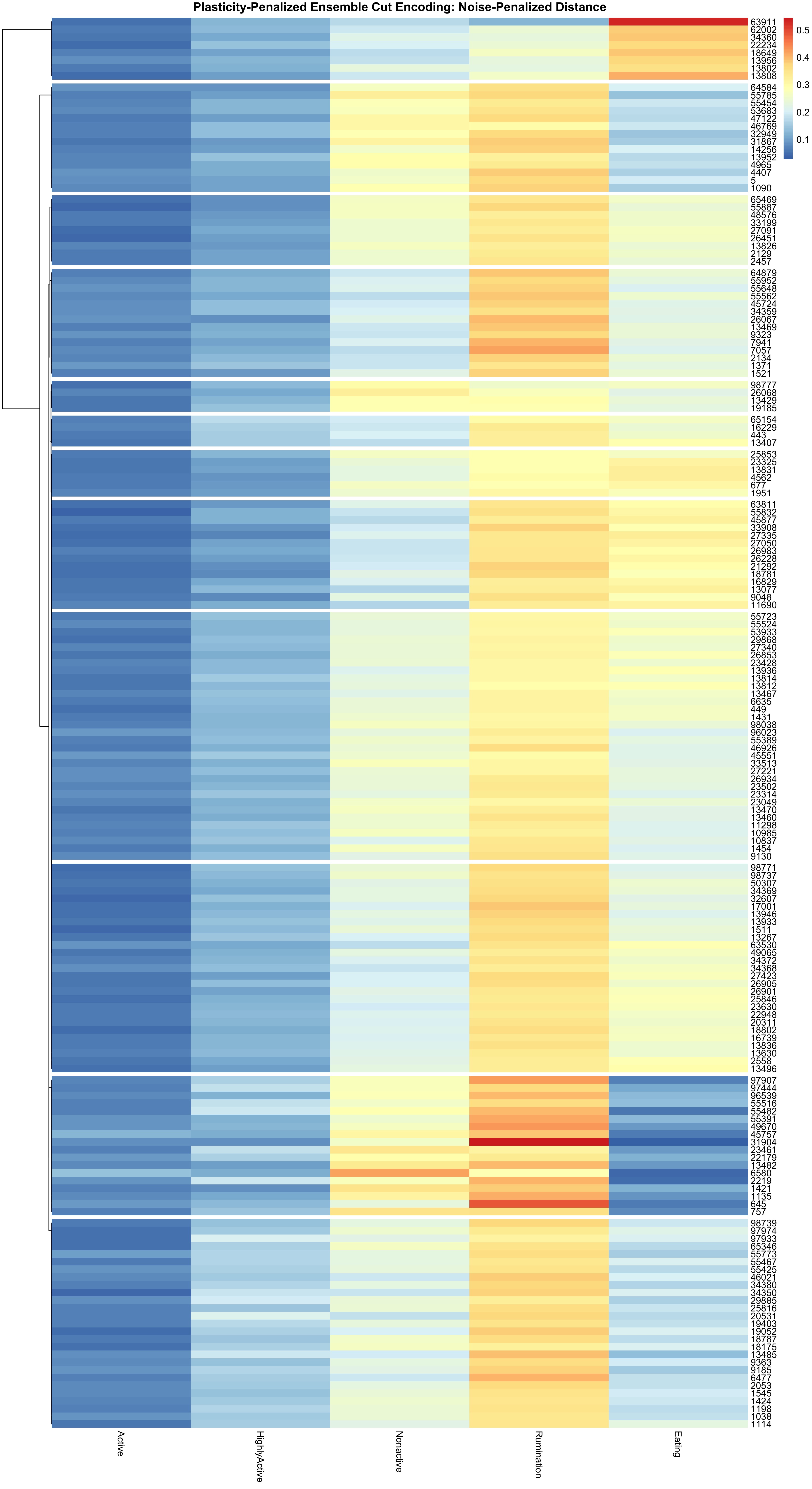

Supplement: Supplementary file 1 [file sensors-22-00001-s001.zip › sensors-1463895-supplementary/OverallTB/EnsembleCut/NPEncode/PPCut/NPEncode_R12_C0.jpeg]

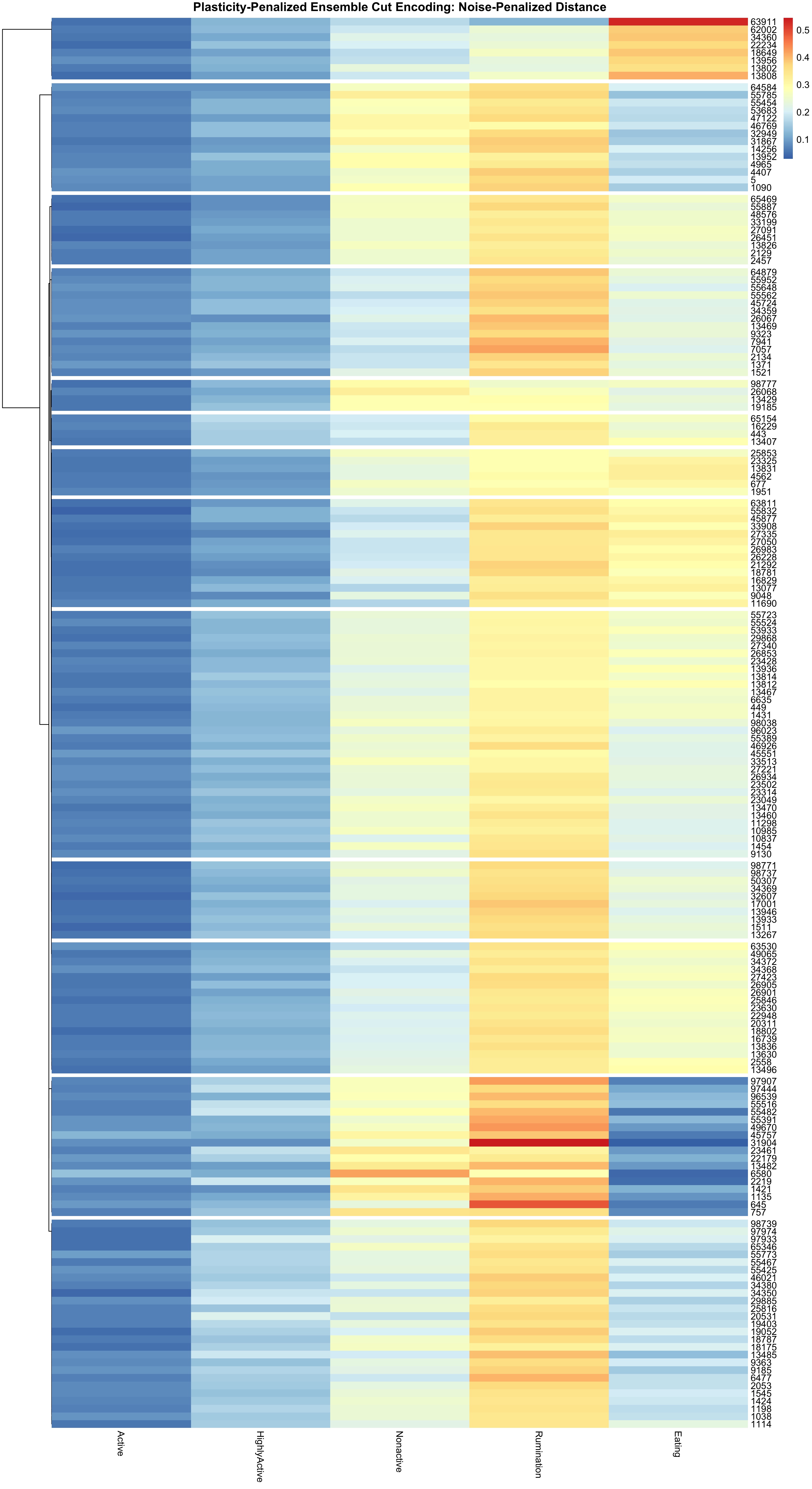

Supplement: Supplementary file 1 [file sensors-22-00001-s001.zip › sensors-1463895-supplementary/OverallTB/EnsembleCut/NPEncode/PPCut/NPEncode_R13_C0.jpeg]

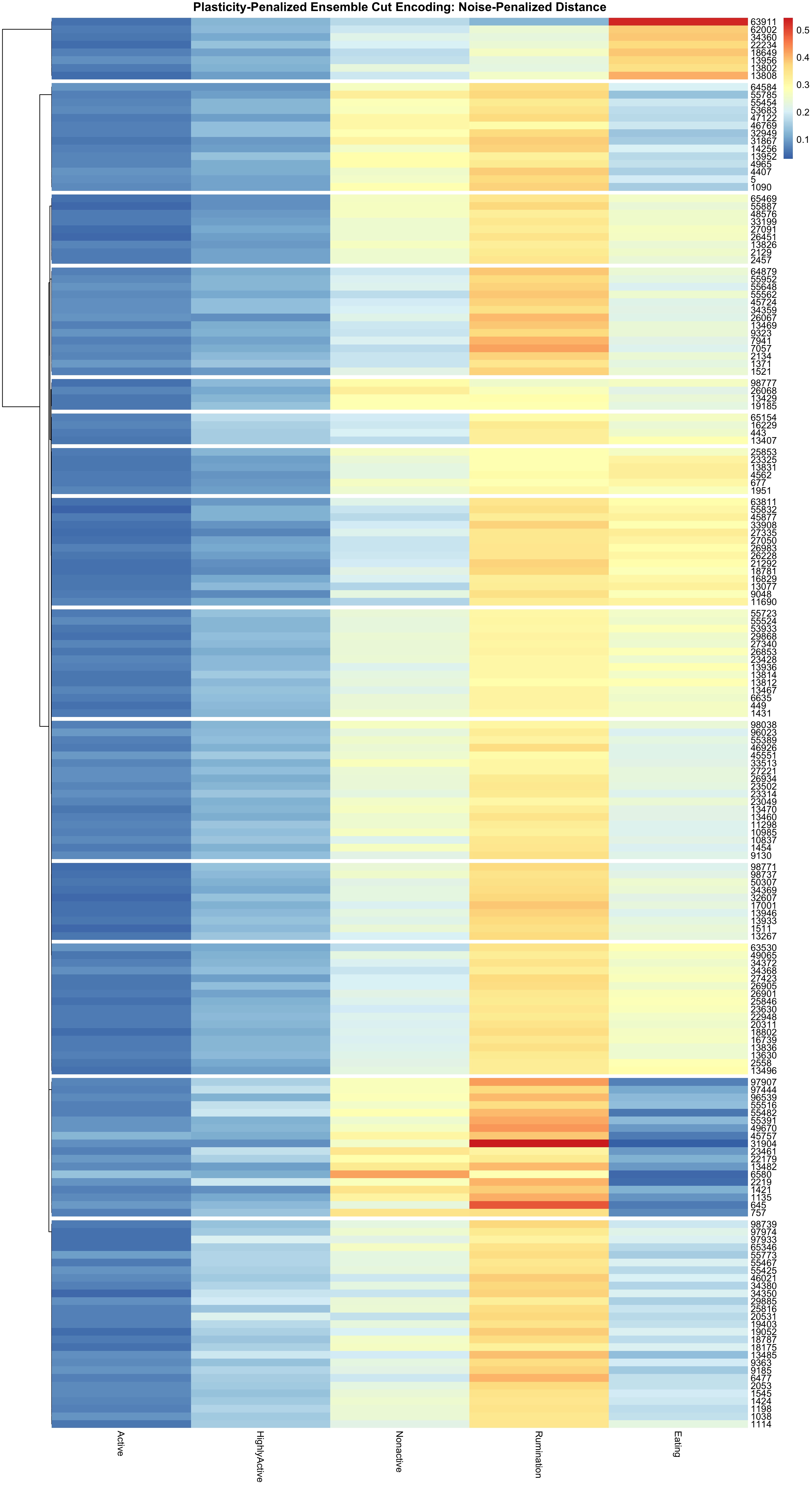

Supplement: Supplementary file 1 [file sensors-22-00001-s001.zip › sensors-1463895-supplementary/OverallTB/EnsembleCut/NPEncode/PPCut/NPEncode_R14_C0.jpeg]

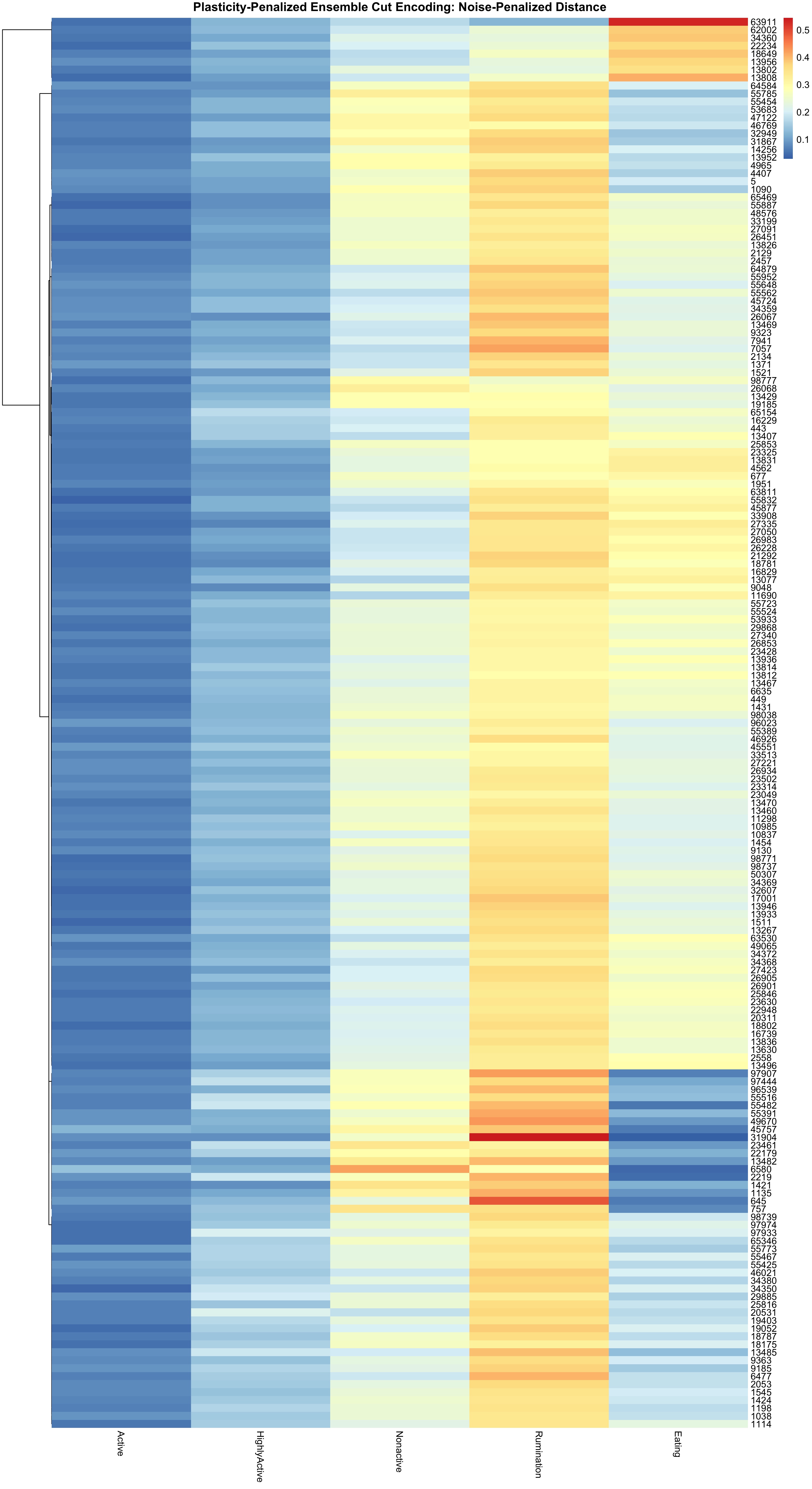

Supplement: Supplementary file 1 [file sensors-22-00001-s001.zip › sensors-1463895-supplementary/OverallTB/EnsembleCut/NPEncode/PPCut/NPEncode_R1_C0.jpeg]

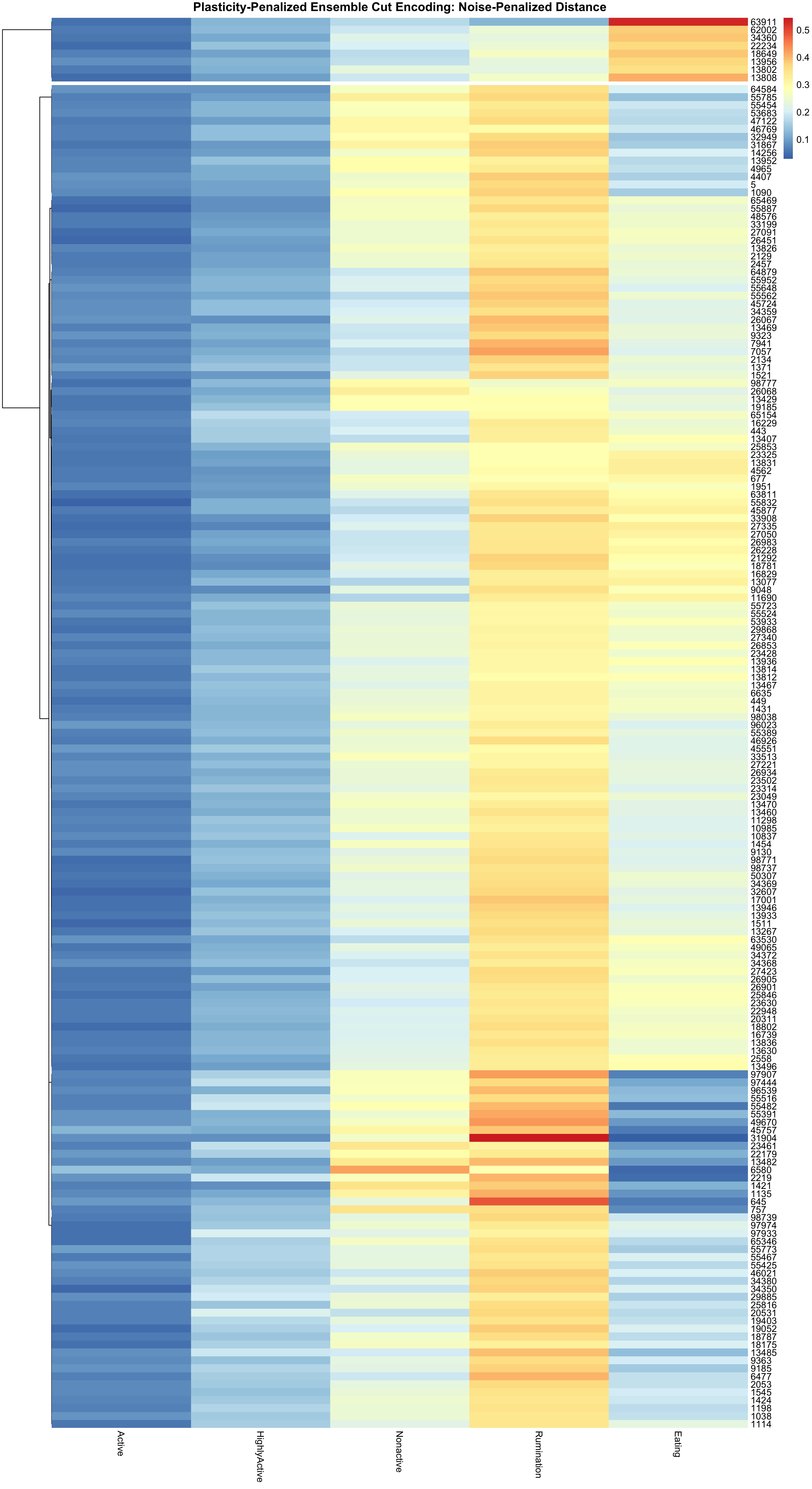

Supplement: Supplementary file 1 [file sensors-22-00001-s001.zip › sensors-1463895-supplementary/OverallTB/EnsembleCut/NPEncode/PPCut/NPEncode_R2_C0.jpeg]

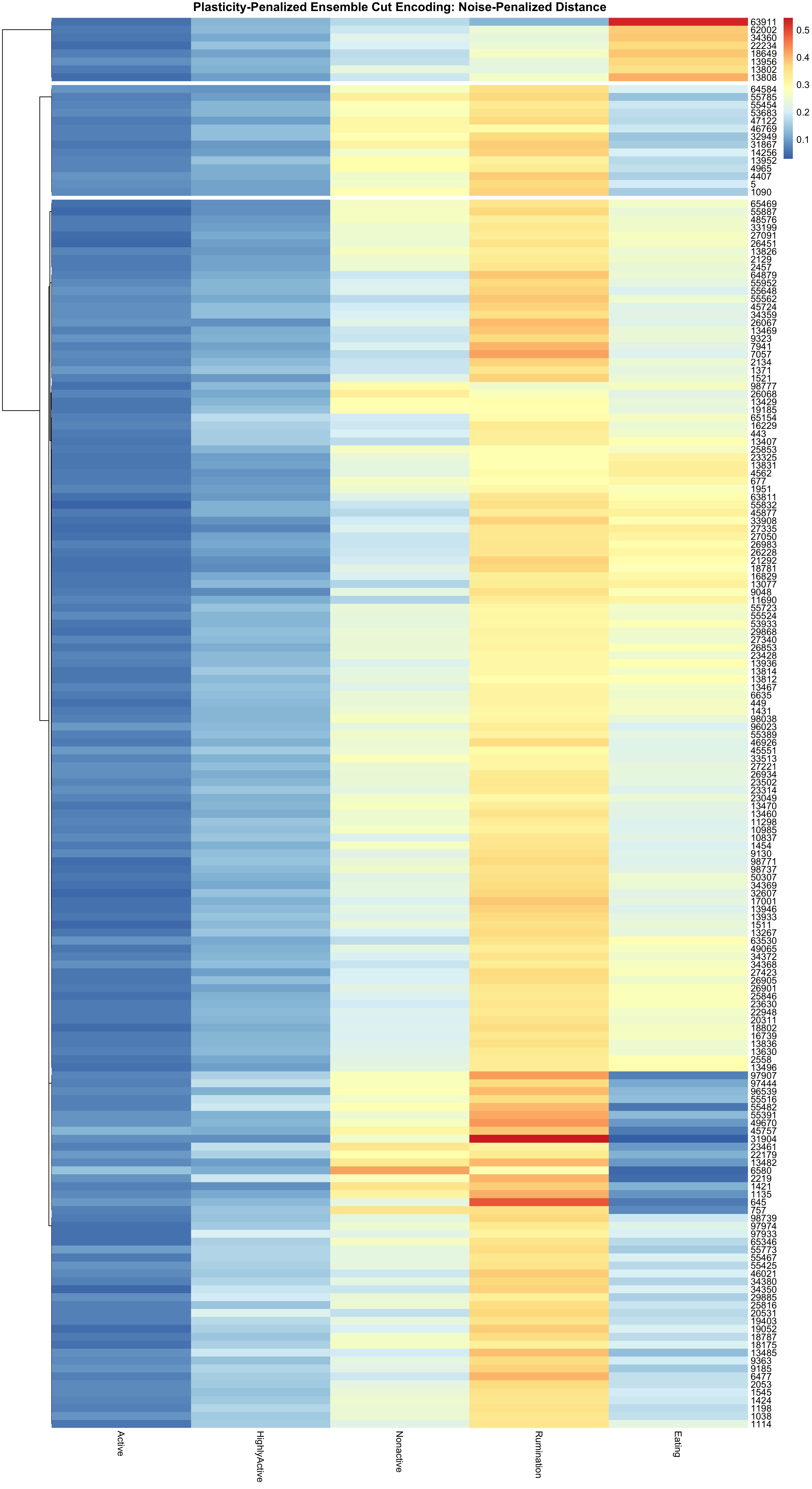

Supplement: Supplementary file 1 [file sensors-22-00001-s001.zip › sensors-1463895-supplementary/OverallTB/EnsembleCut/NPEncode/PPCut/NPEncode_R3_C0.jpeg]

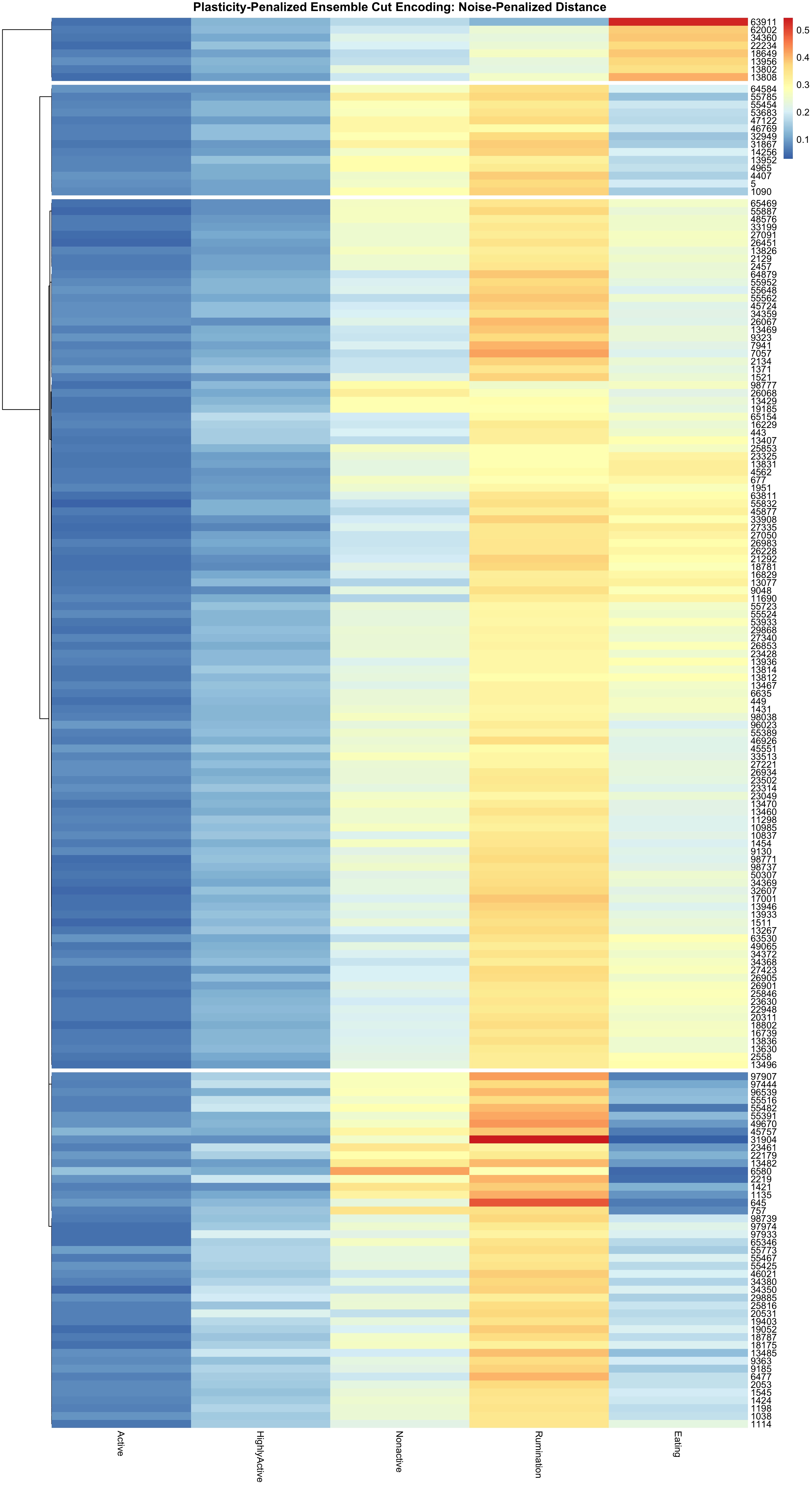

Supplement: Supplementary file 1 [file sensors-22-00001-s001.zip › sensors-1463895-supplementary/OverallTB/EnsembleCut/NPEncode/PPCut/NPEncode_R4_C0.jpeg]

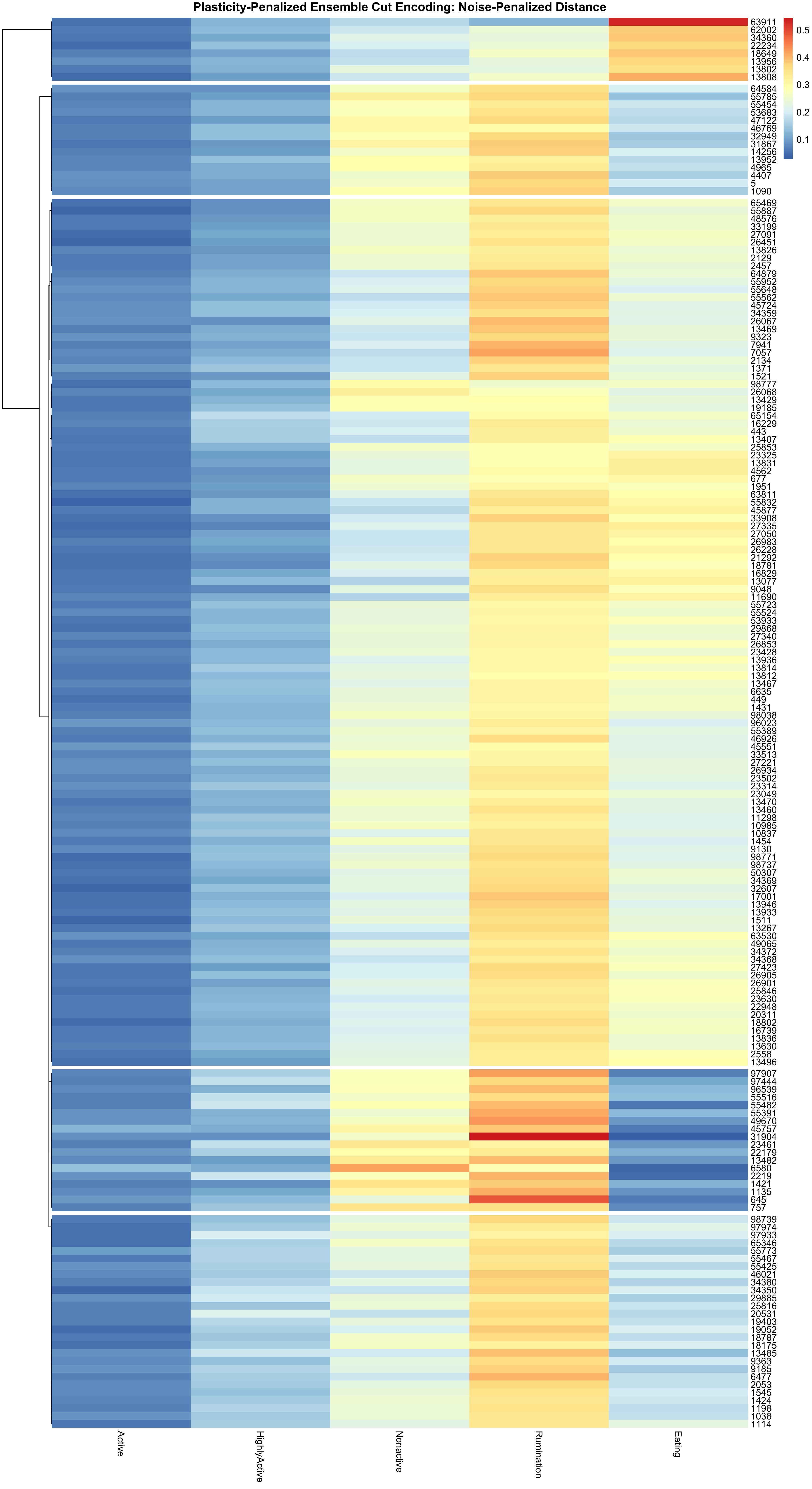

Supplement: Supplementary file 1 [file sensors-22-00001-s001.zip › sensors-1463895-supplementary/OverallTB/EnsembleCut/NPEncode/PPCut/NPEncode_R5_C0.jpeg]

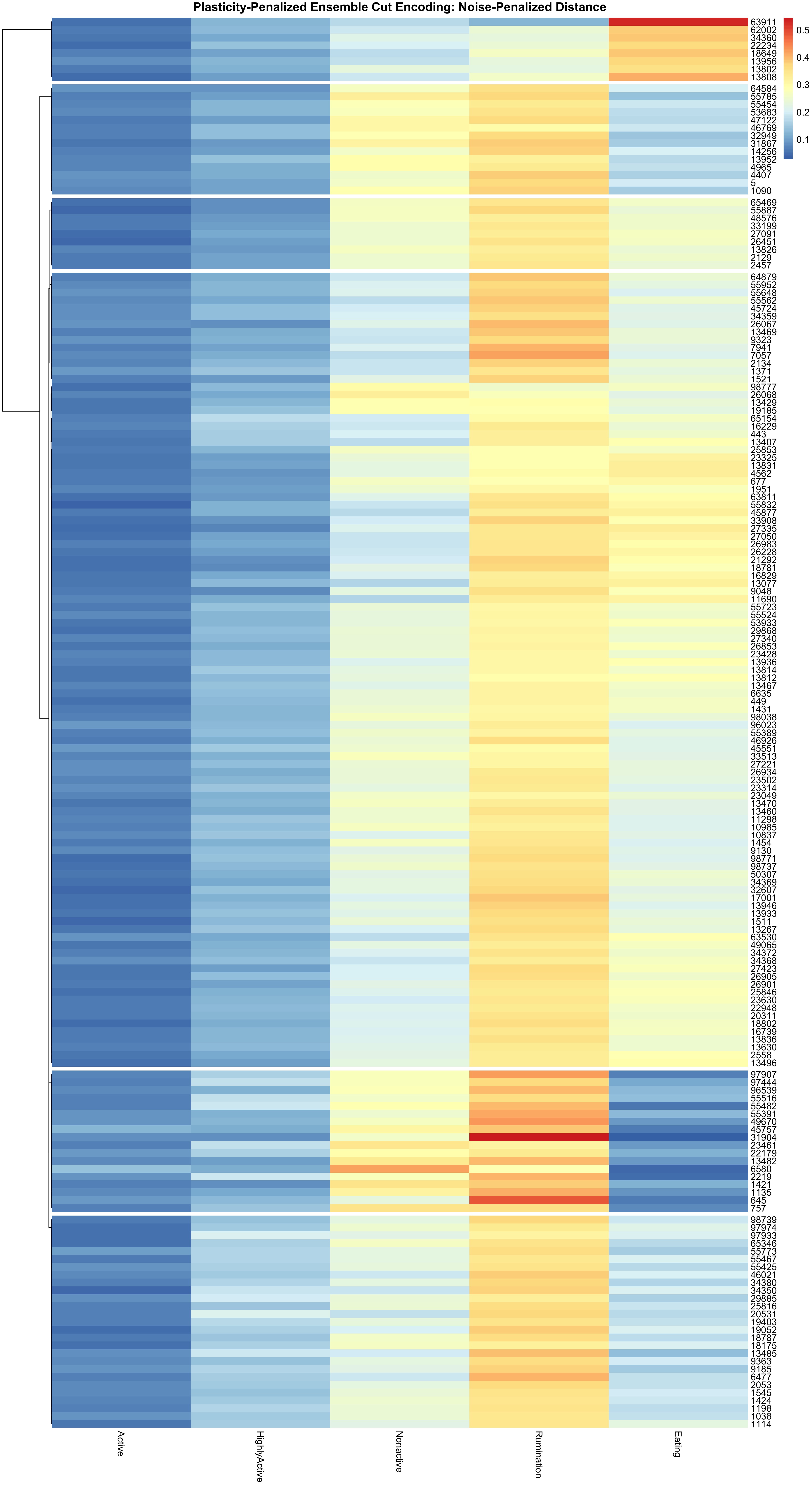

Supplement: Supplementary file 1 [file sensors-22-00001-s001.zip › sensors-1463895-supplementary/OverallTB/EnsembleCut/NPEncode/PPCut/NPEncode_R6_C0.jpeg]

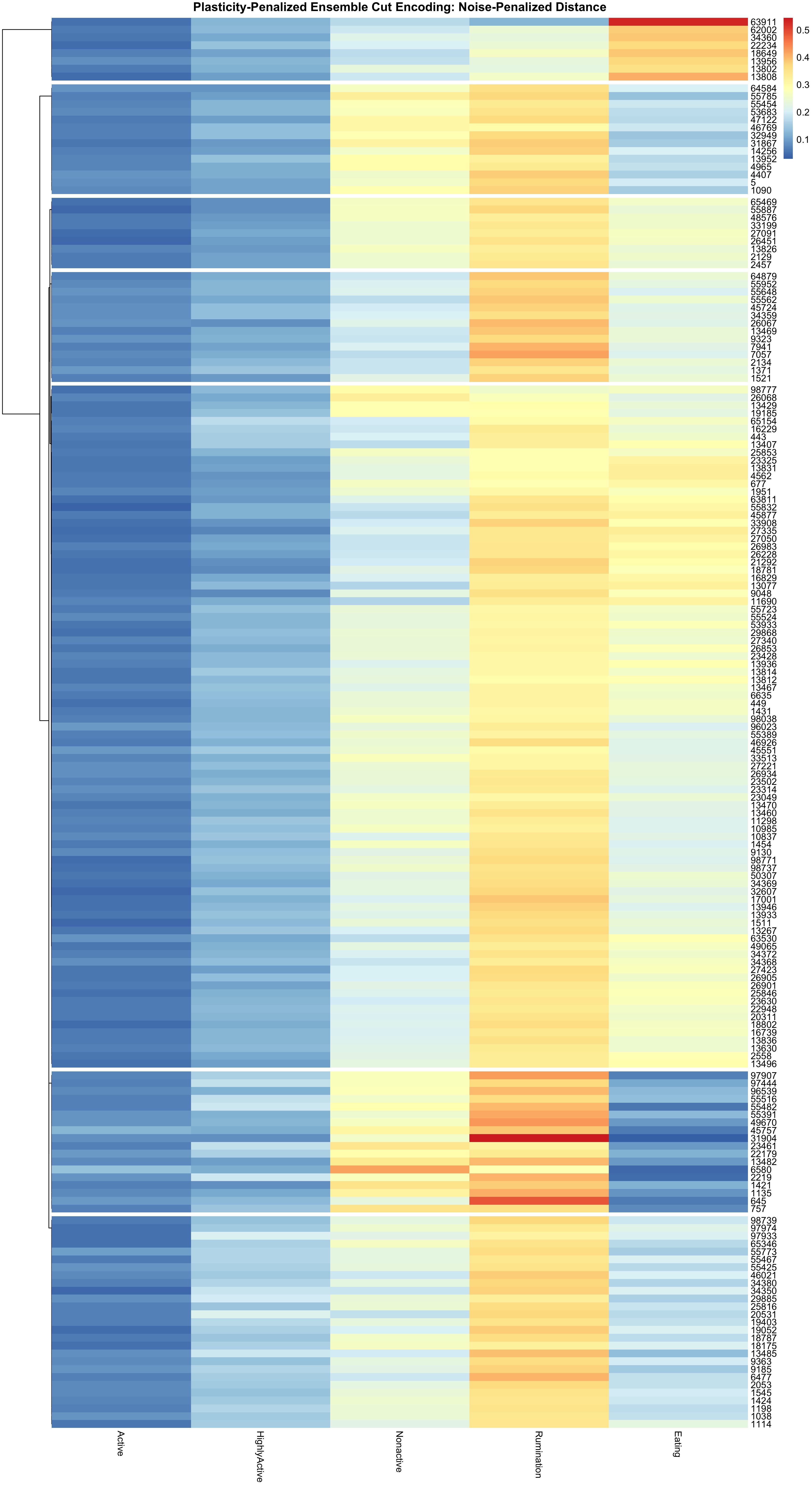

Supplement: Supplementary file 1 [file sensors-22-00001-s001.zip › sensors-1463895-supplementary/OverallTB/EnsembleCut/NPEncode/PPCut/NPEncode_R7_C0.jpeg]

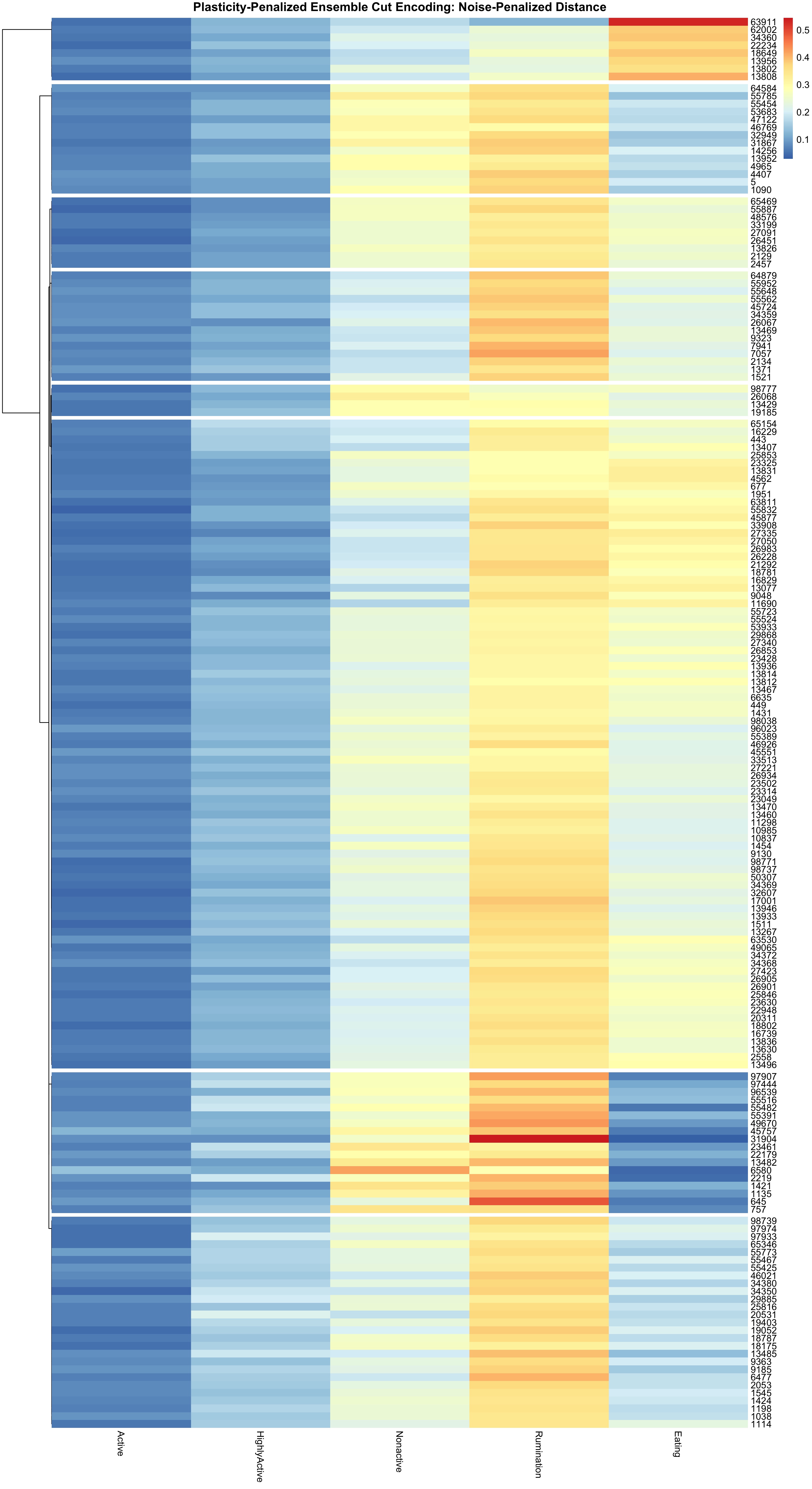

Supplement: Supplementary file 1 [file sensors-22-00001-s001.zip › sensors-1463895-supplementary/OverallTB/EnsembleCut/NPEncode/PPCut/NPEncode_R8_C0.jpeg]

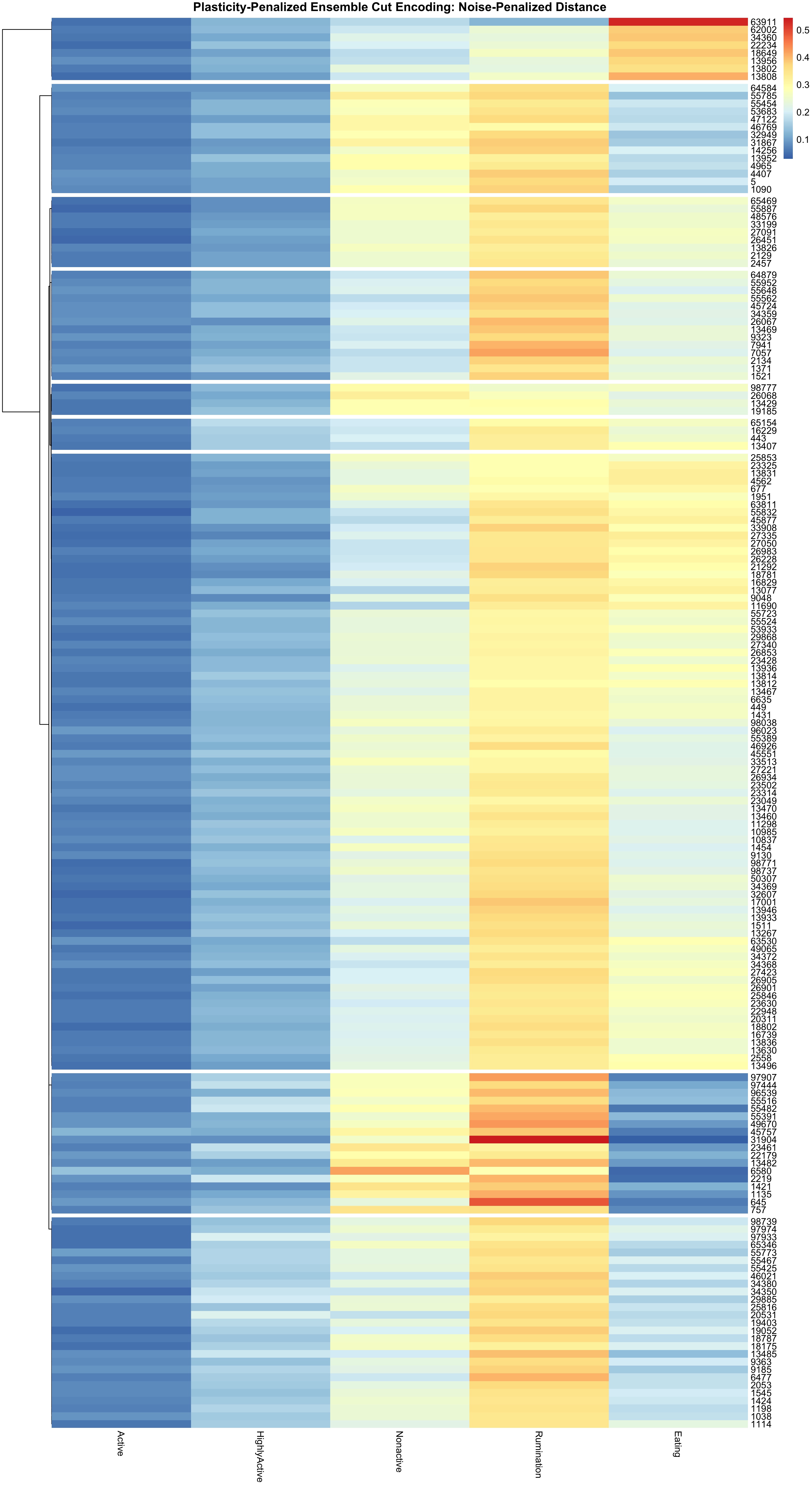

Supplement: Supplementary file 1 [file sensors-22-00001-s001.zip › sensors-1463895-supplementary/OverallTB/EnsembleCut/NPEncode/PPCut/NPEncode_R9_C0.jpeg]

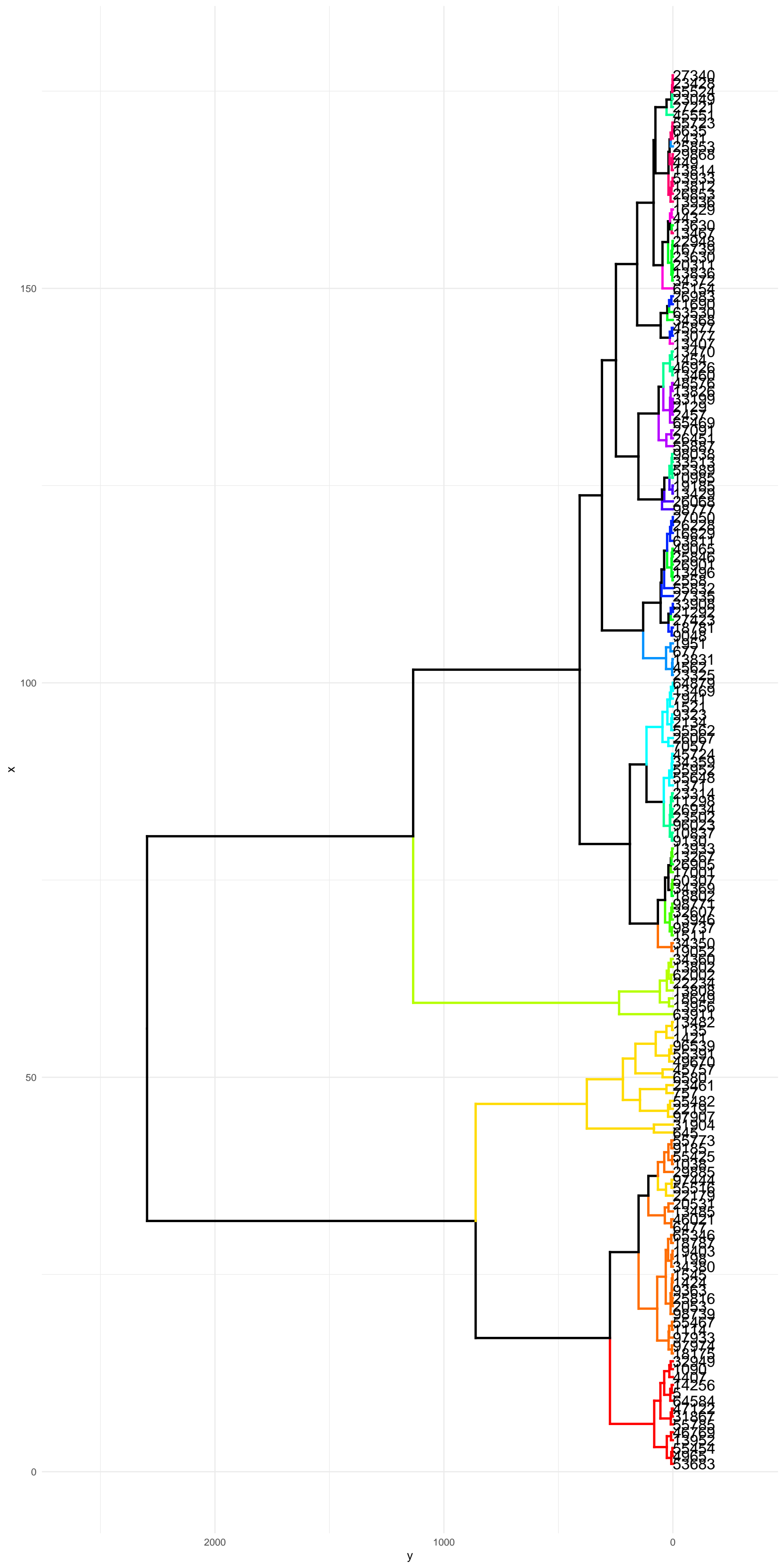

Supplement: Supplementary file 1 [file sensors-22-00001-s001.zip › sensors-1463895-supplementary/OverallTB/EnsembleCut/NPEncode/PPCut_Dendrogram.pdf]

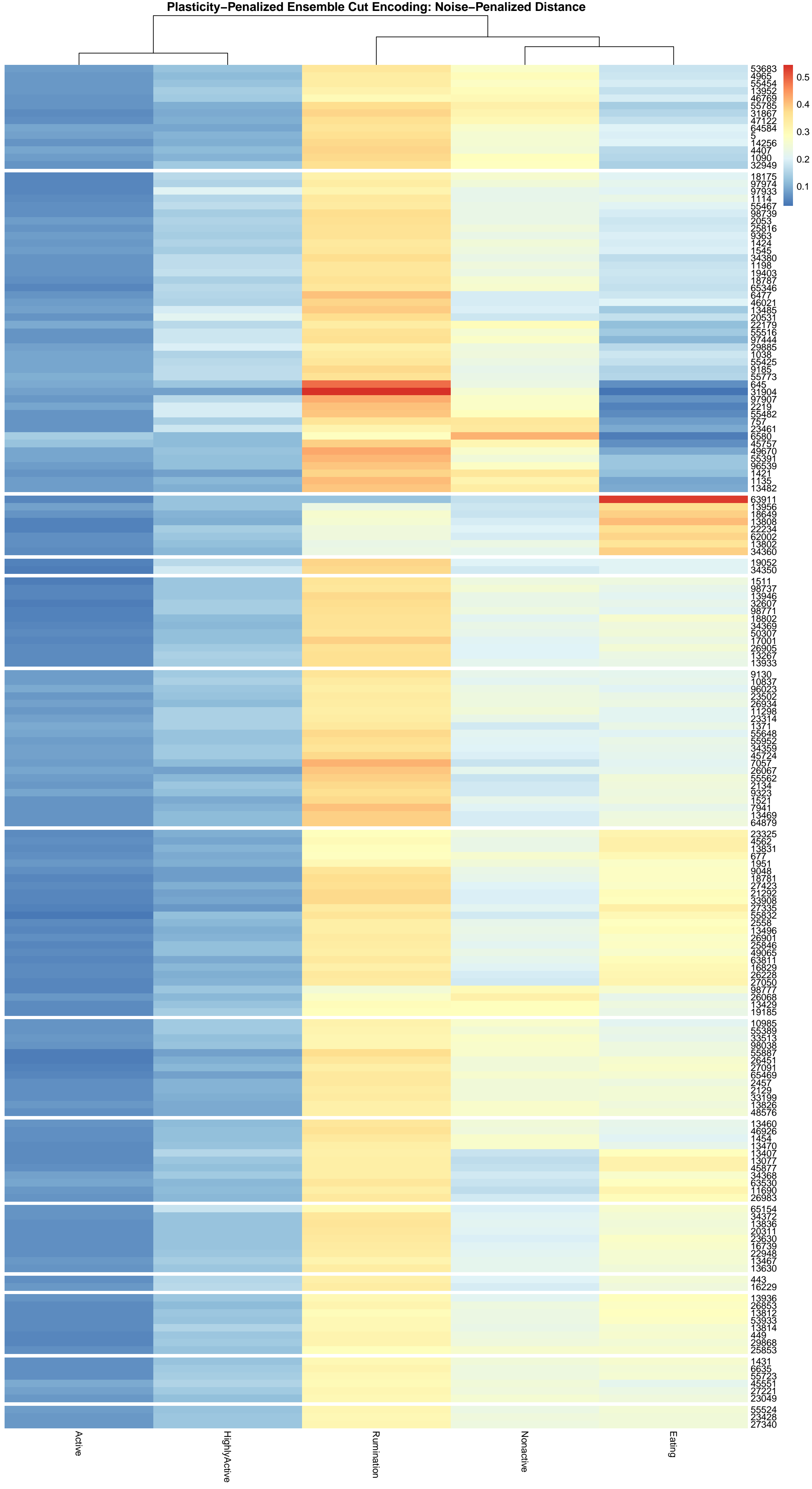

Supplement: Supplementary file 1 [file sensors-22-00001-s001.zip › sensors-1463895-supplementary/OverallTB/EnsembleCut/NPEncode/PPCut_Heatmap.pdf]

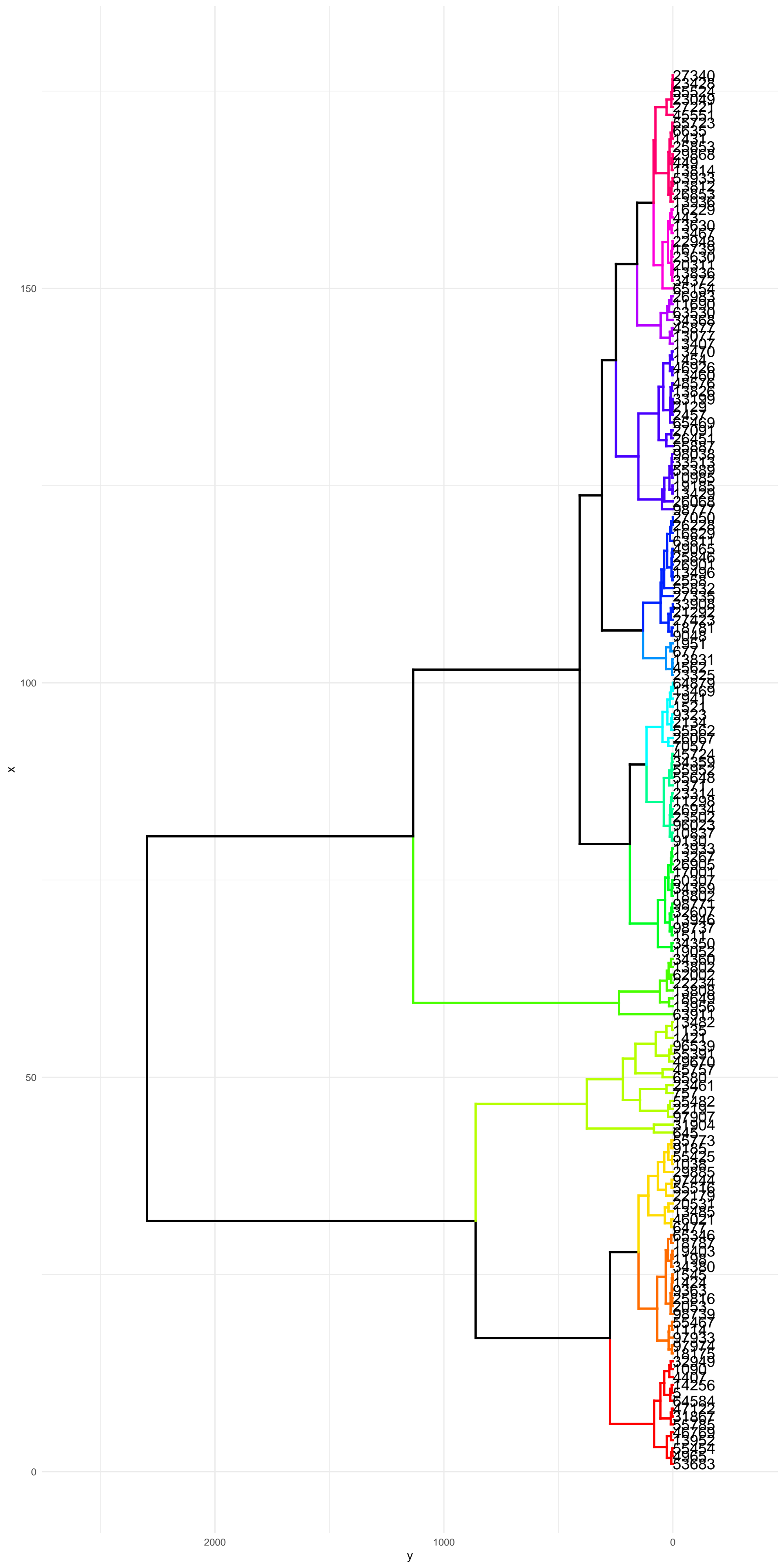

Supplement: Supplementary file 1 [file sensors-22-00001-s001.zip › sensors-1463895-supplementary/OverallTB/EnsembleCut/PlastPen_PP_Dendrogram.pdf]

Plasticity-Penalized Ensemble Cut Encoding: Plasticity-Penalized Distance

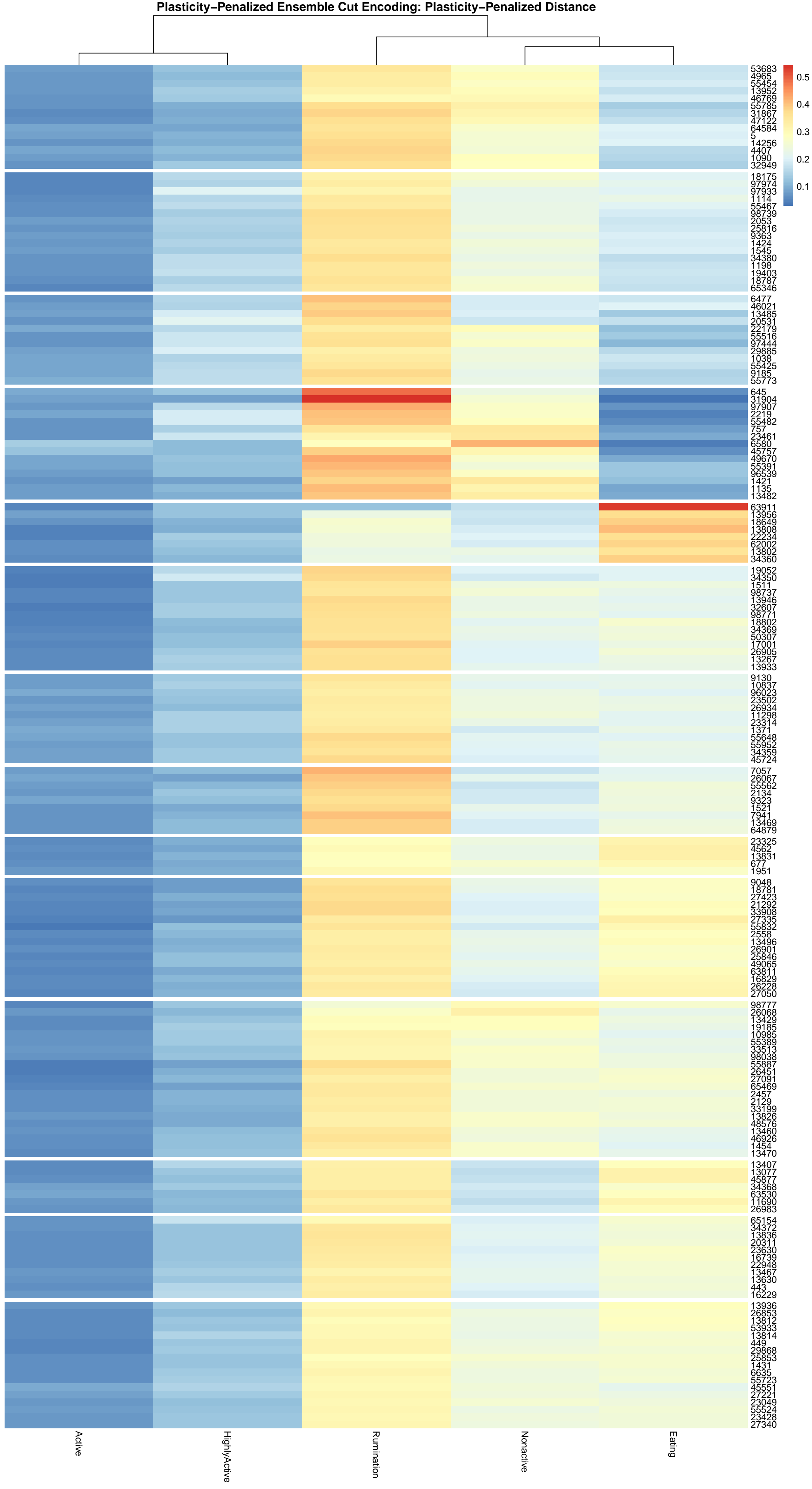

Supplement: Supplementary file 1 [file sensors-22-00001-s001.zip › sensors-1463895-supplementary/OverallTB/EnsembleCut/PlastPen_PP_Heatmap.pdf]

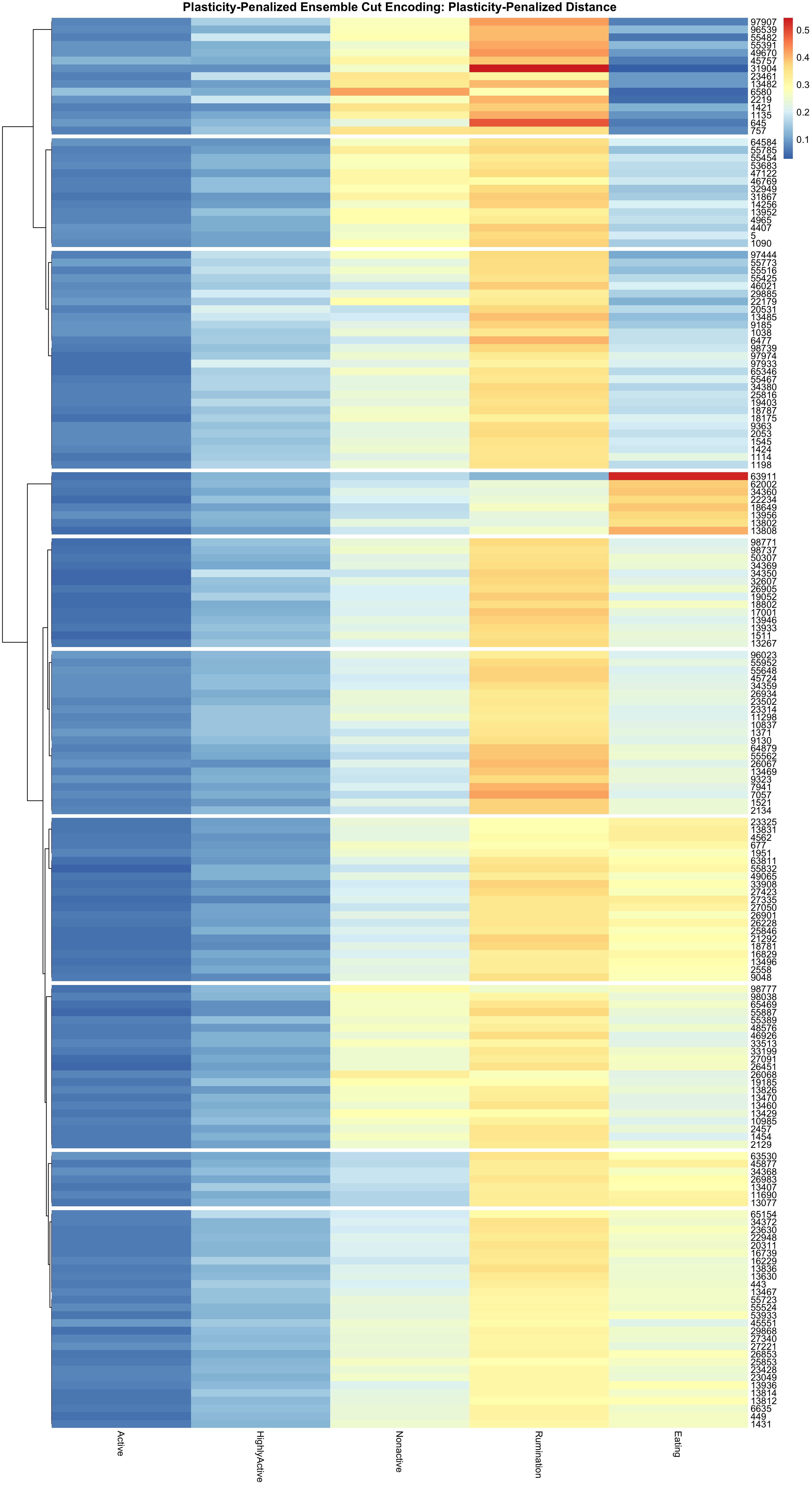

Supplement: Supplementary file 1 [file sensors-22-00001-s001.zip › sensors-1463895-supplementary/OverallTB/EnsembleCut/PPEncode/PWEncode_R10_C0.jpeg]

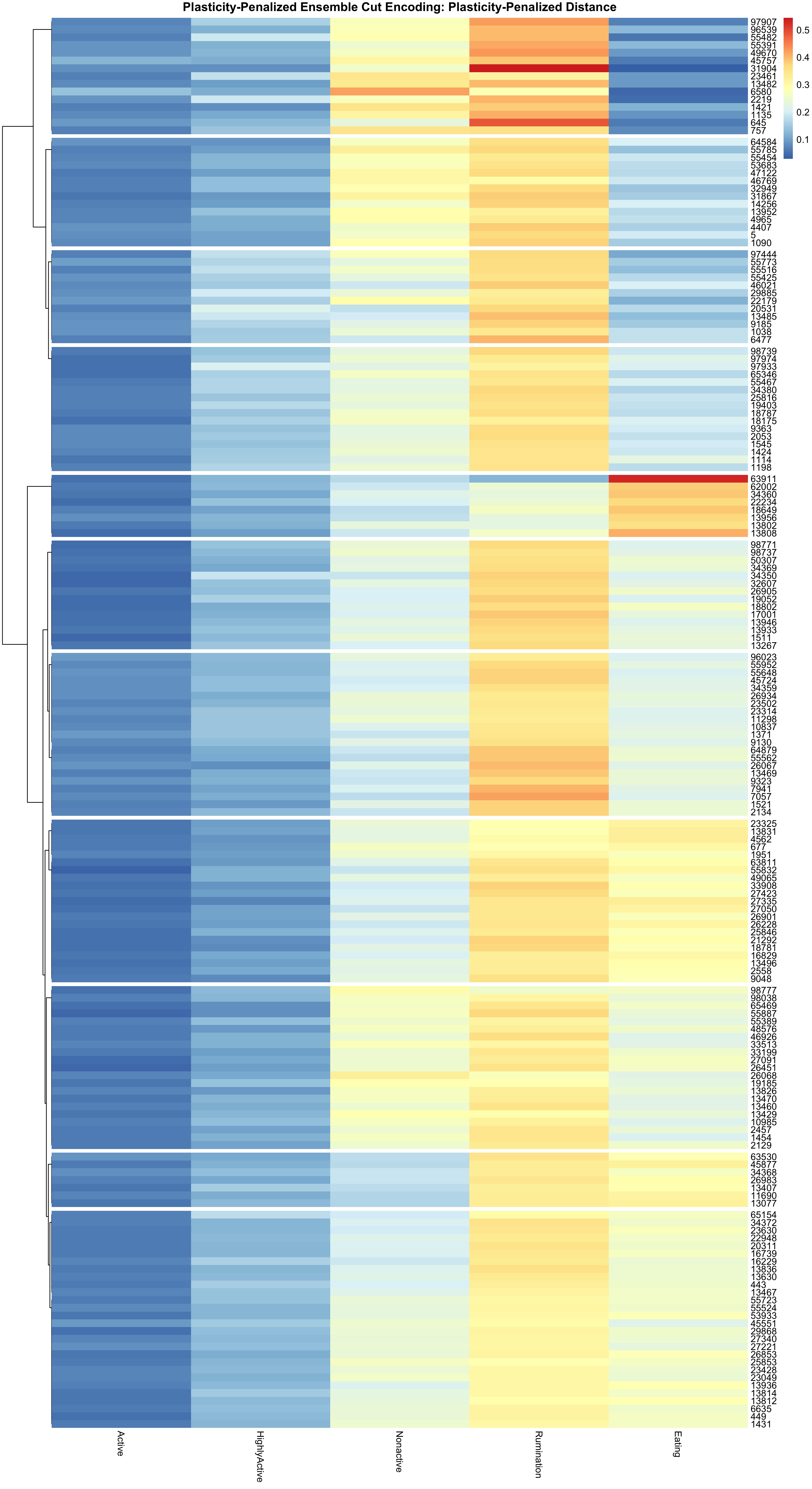

Supplement: Supplementary file 1 [file sensors-22-00001-s001.zip › sensors-1463895-supplementary/OverallTB/EnsembleCut/PPEncode/PWEncode_R11_C0.jpeg]

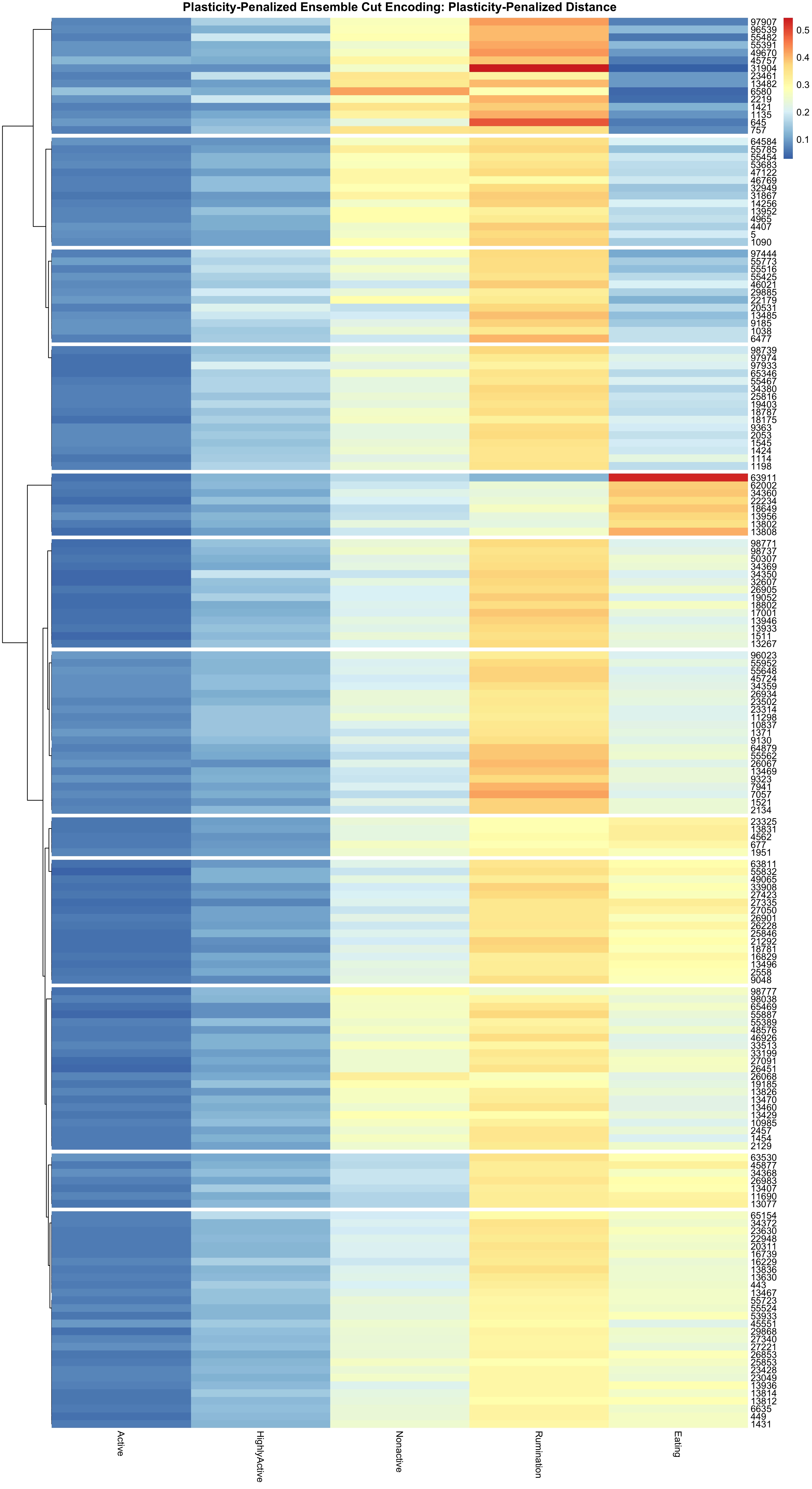

Supplement: Supplementary file 1 [file sensors-22-00001-s001.zip › sensors-1463895-supplementary/OverallTB/EnsembleCut/PPEncode/PWEncode_R12_C0.jpeg]

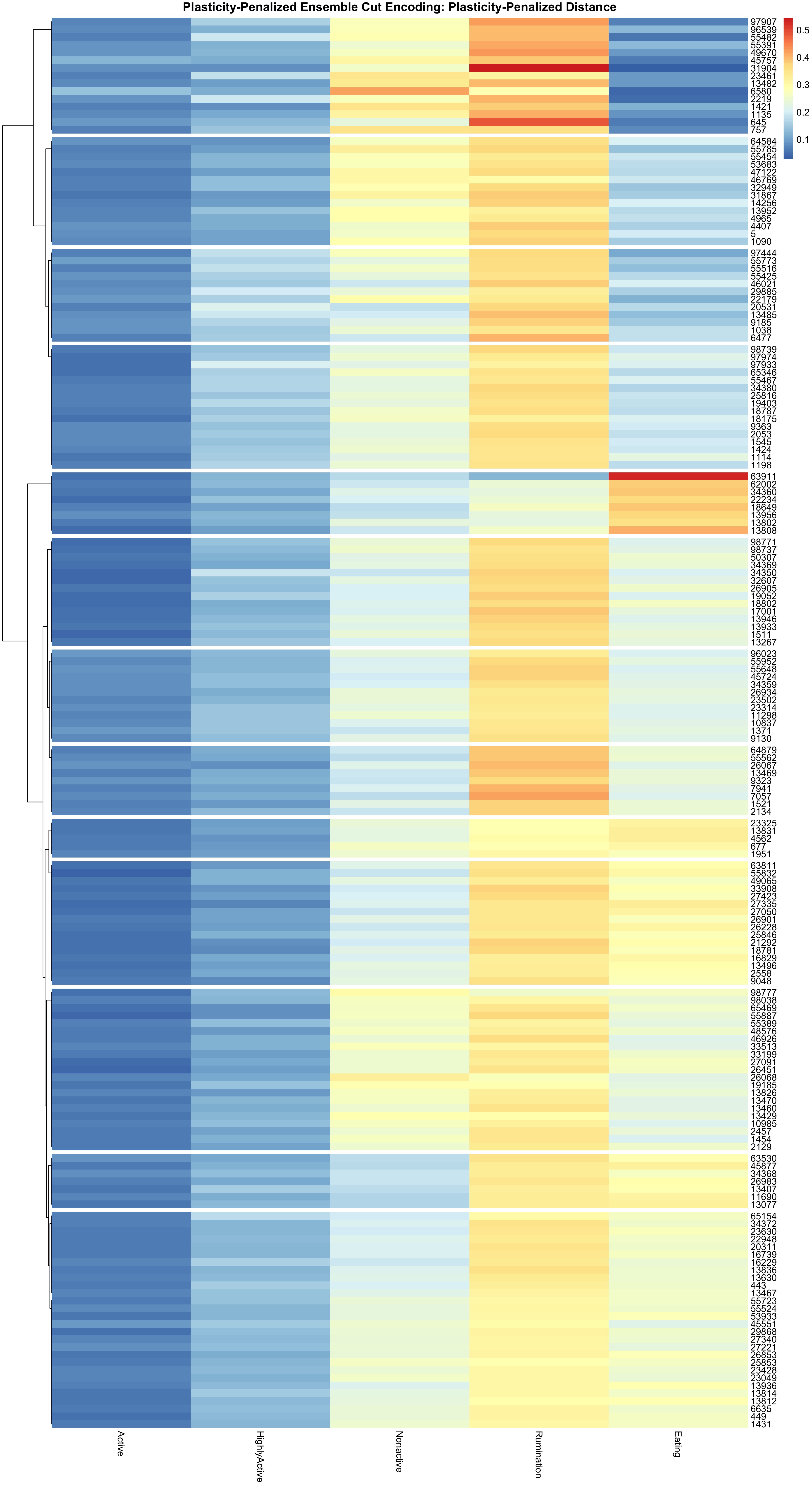

Supplement: Supplementary file 1 [file sensors-22-00001-s001.zip › sensors-1463895-supplementary/OverallTB/EnsembleCut/PPEncode/PWEncode_R13_C0.jpeg]

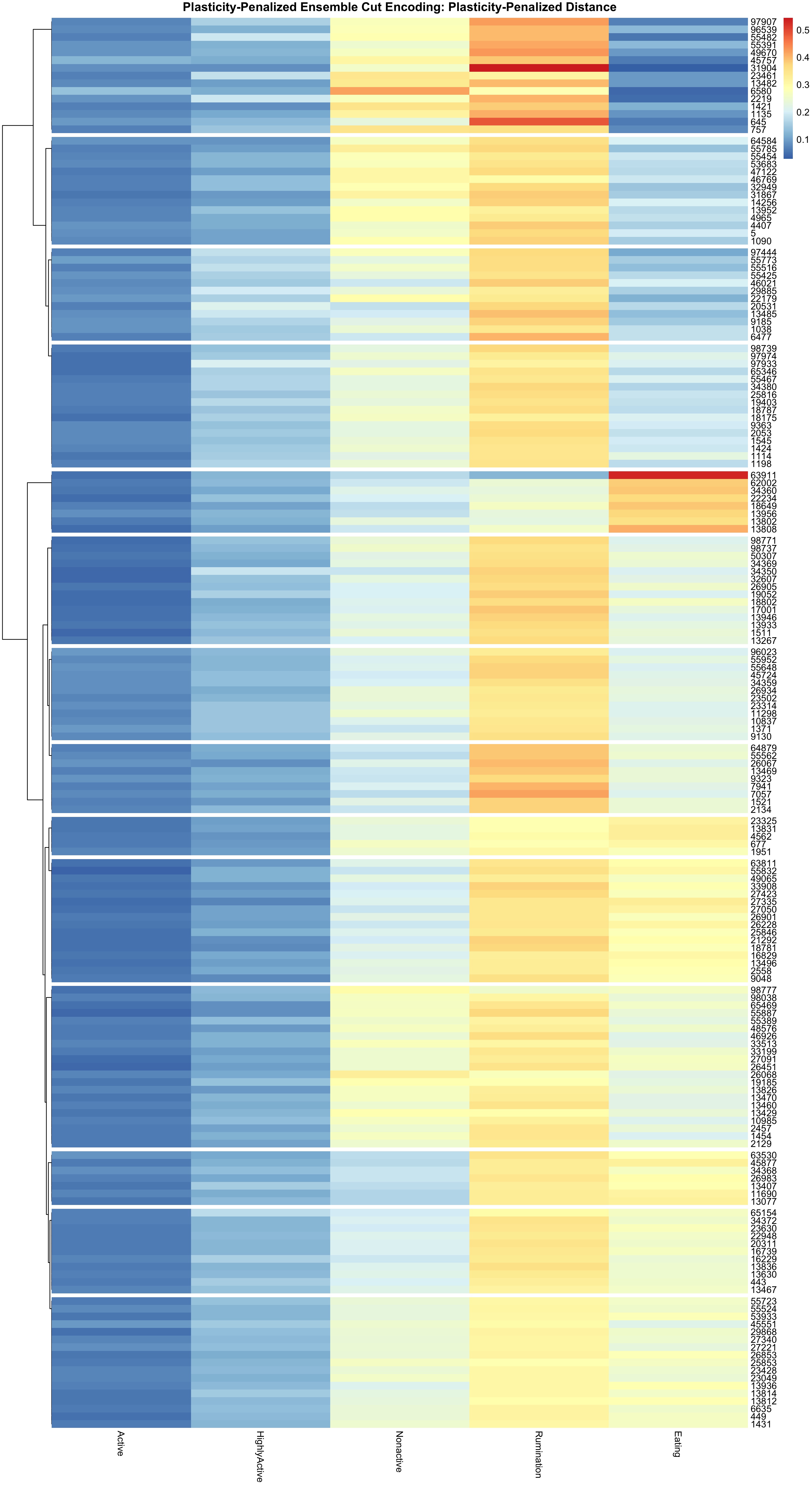

Supplement: Supplementary file 1 [file sensors-22-00001-s001.zip › sensors-1463895-supplementary/OverallTB/EnsembleCut/PPEncode/PWEncode_R14_C0.jpeg]

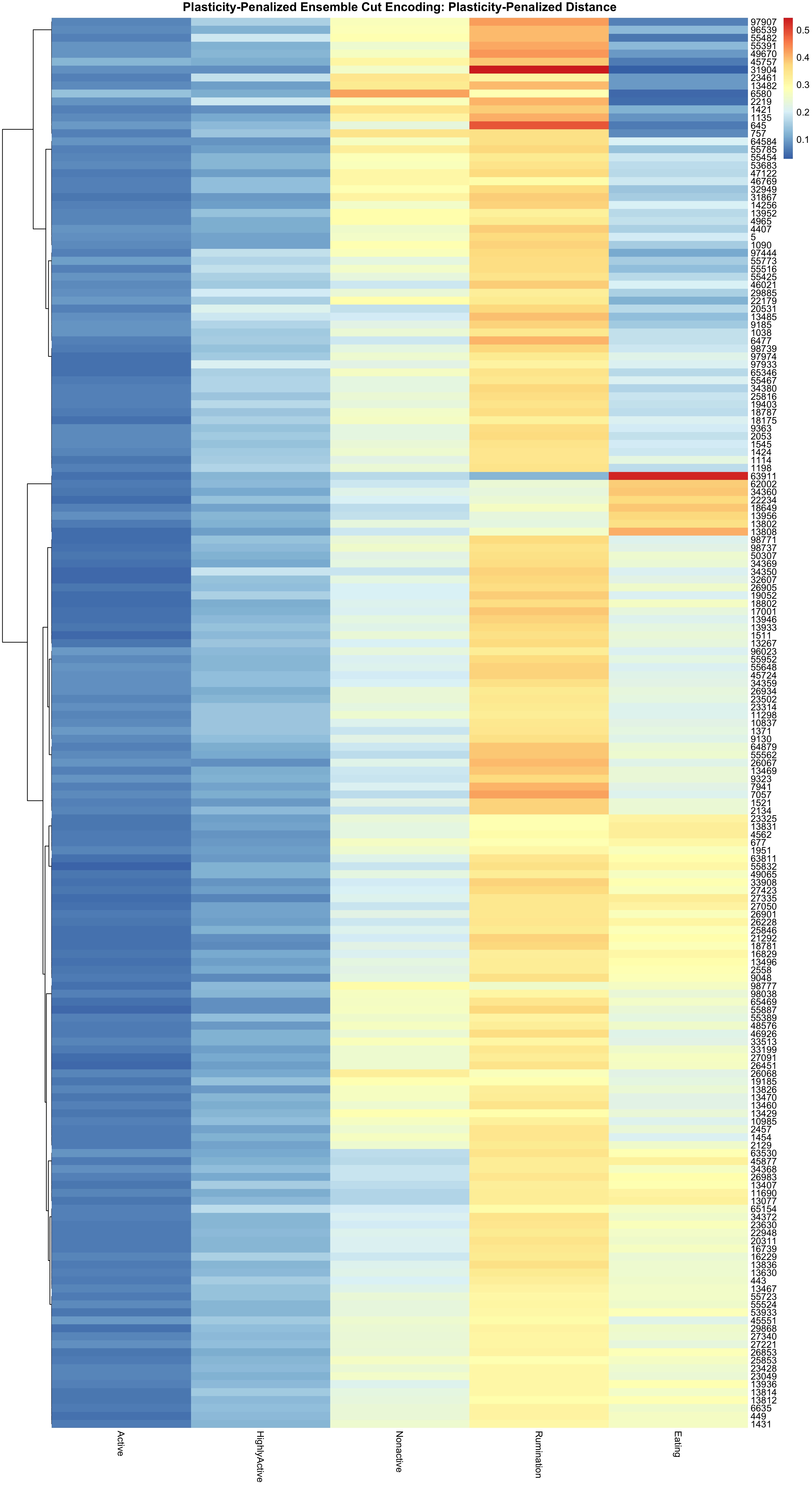

Supplement: Supplementary file 1 [file sensors-22-00001-s001.zip › sensors-1463895-supplementary/OverallTB/EnsembleCut/PPEncode/PWEncode_R1_C0.jpeg]

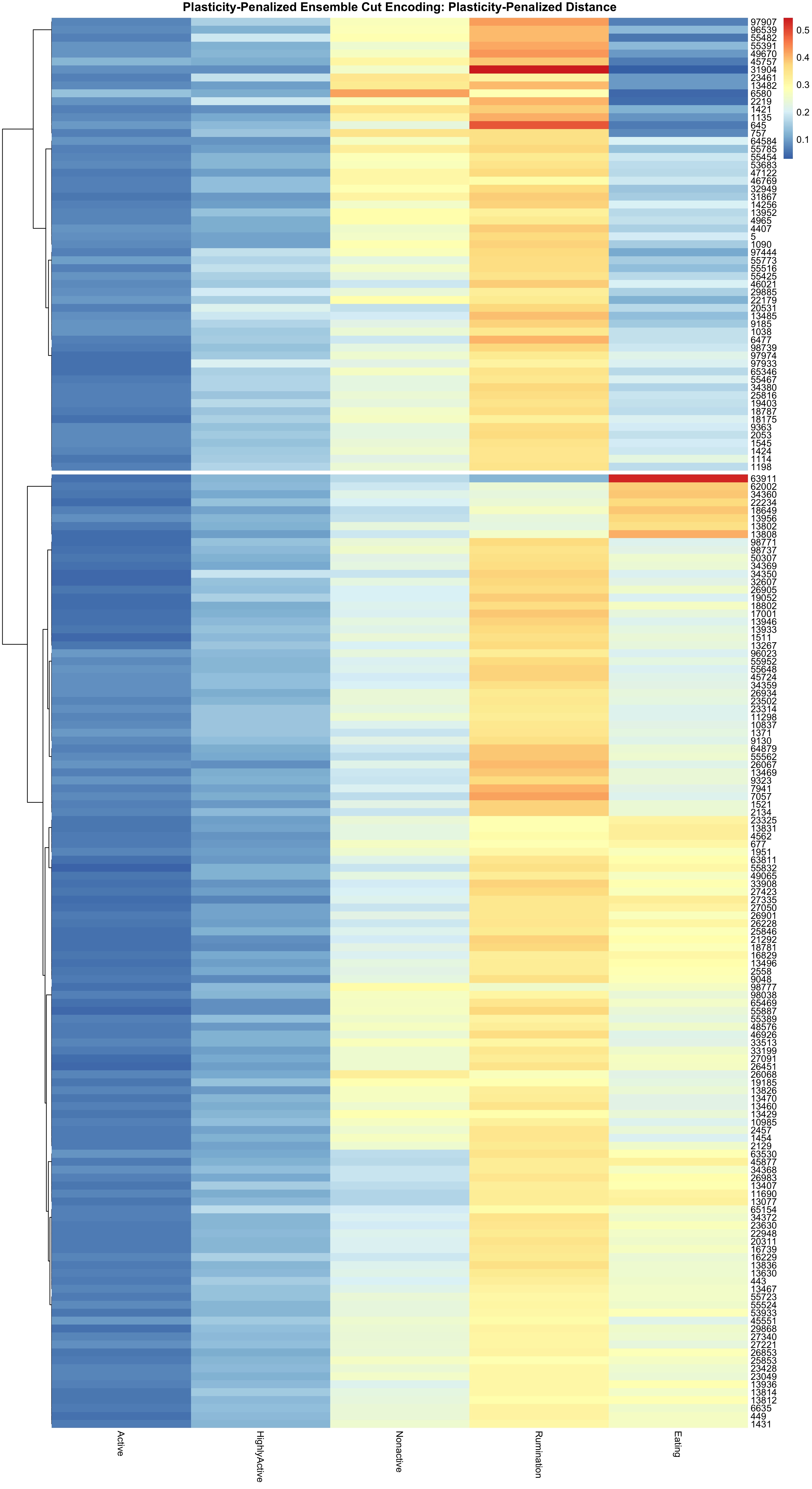

Supplement: Supplementary file 1 [file sensors-22-00001-s001.zip › sensors-1463895-supplementary/OverallTB/EnsembleCut/PPEncode/PWEncode_R2_C0.jpeg]

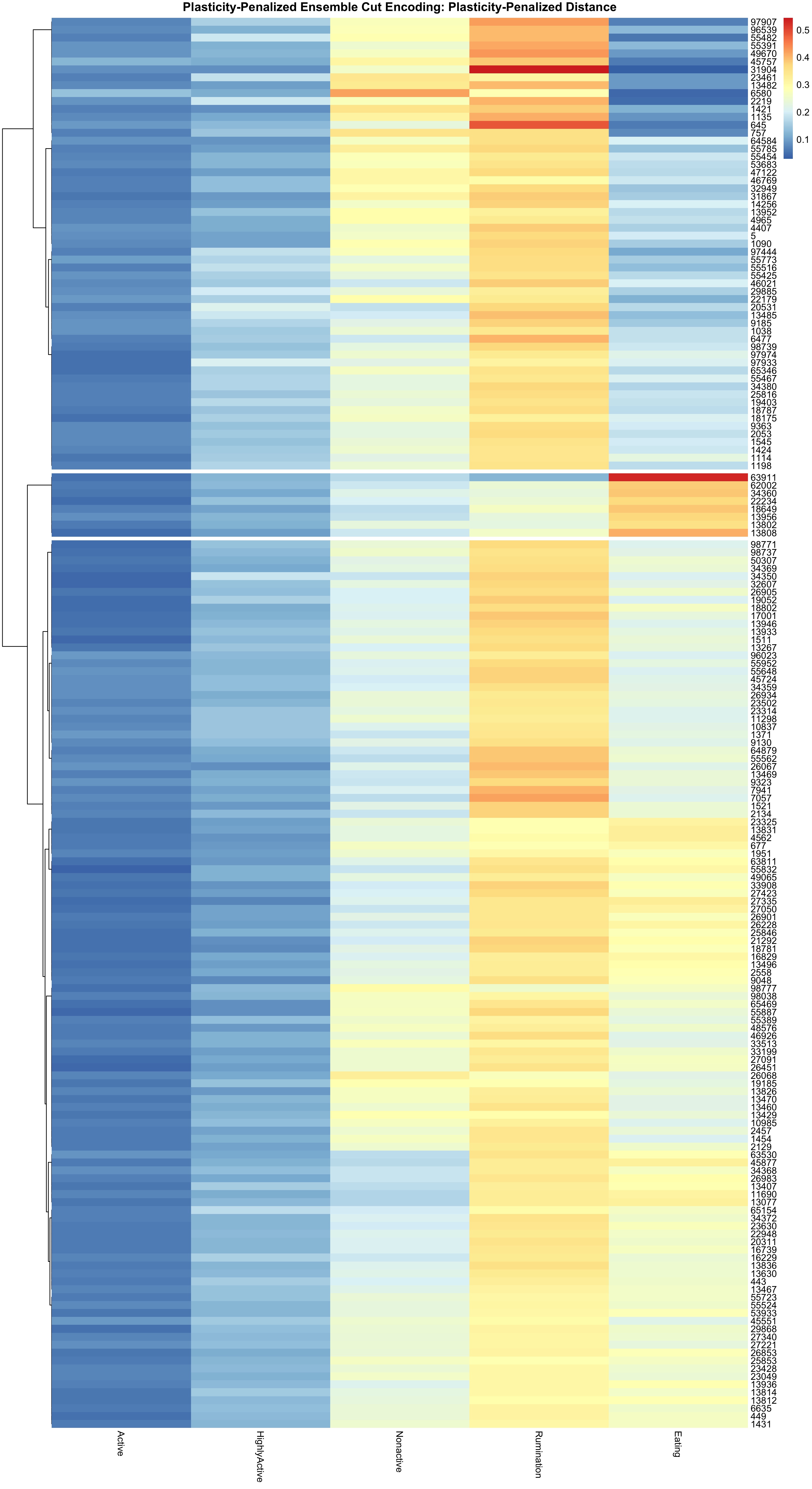

Supplement: Supplementary file 1 [file sensors-22-00001-s001.zip › sensors-1463895-supplementary/OverallTB/EnsembleCut/PPEncode/PWEncode_R3_C0.jpeg]
