# Supplementary material for: Livestock Informatics Toolkit: A Case Study in Visually Characterizing Complex Behavioral Patterns across Multiple Sensor Platforms, Using Novel Unsupervised Machine Learning and Information Theoretic Approaches
Source: Sensors (Basel). 2021 Dec 21;22(1):1. doi: 10.3390/s22010001 (PMC8747447; doi:10.3390/s22010001)
Supplement: Supplementary file 1 [file EO_Healthy.pdf › sensors-1463895-supplementary/OverallTB/BivarTest_AgeYeild/EO_Healthy.pdf]

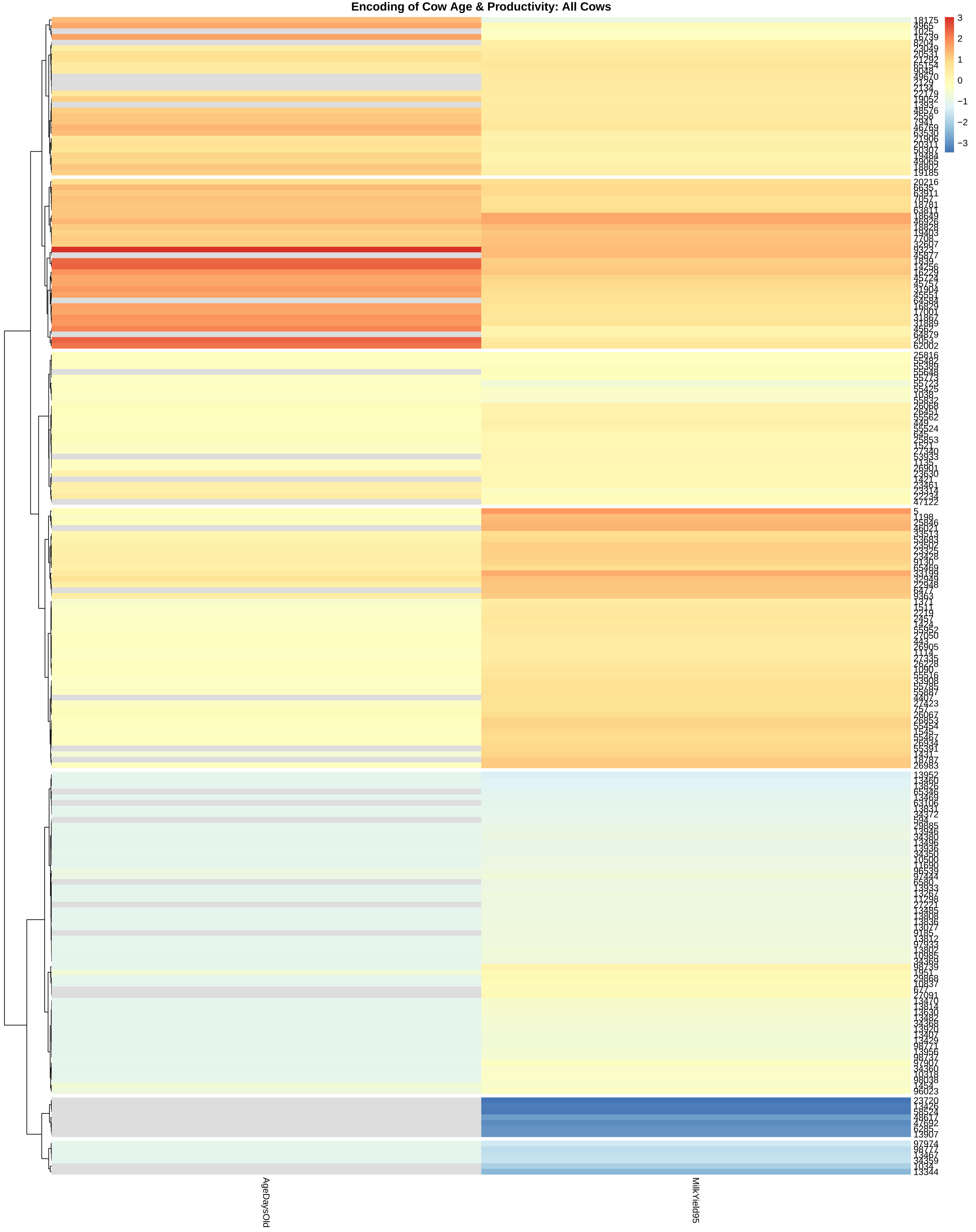
Encoding of Cow Age & Productivity: All Cows
AgeDaysOld
MilkYield95

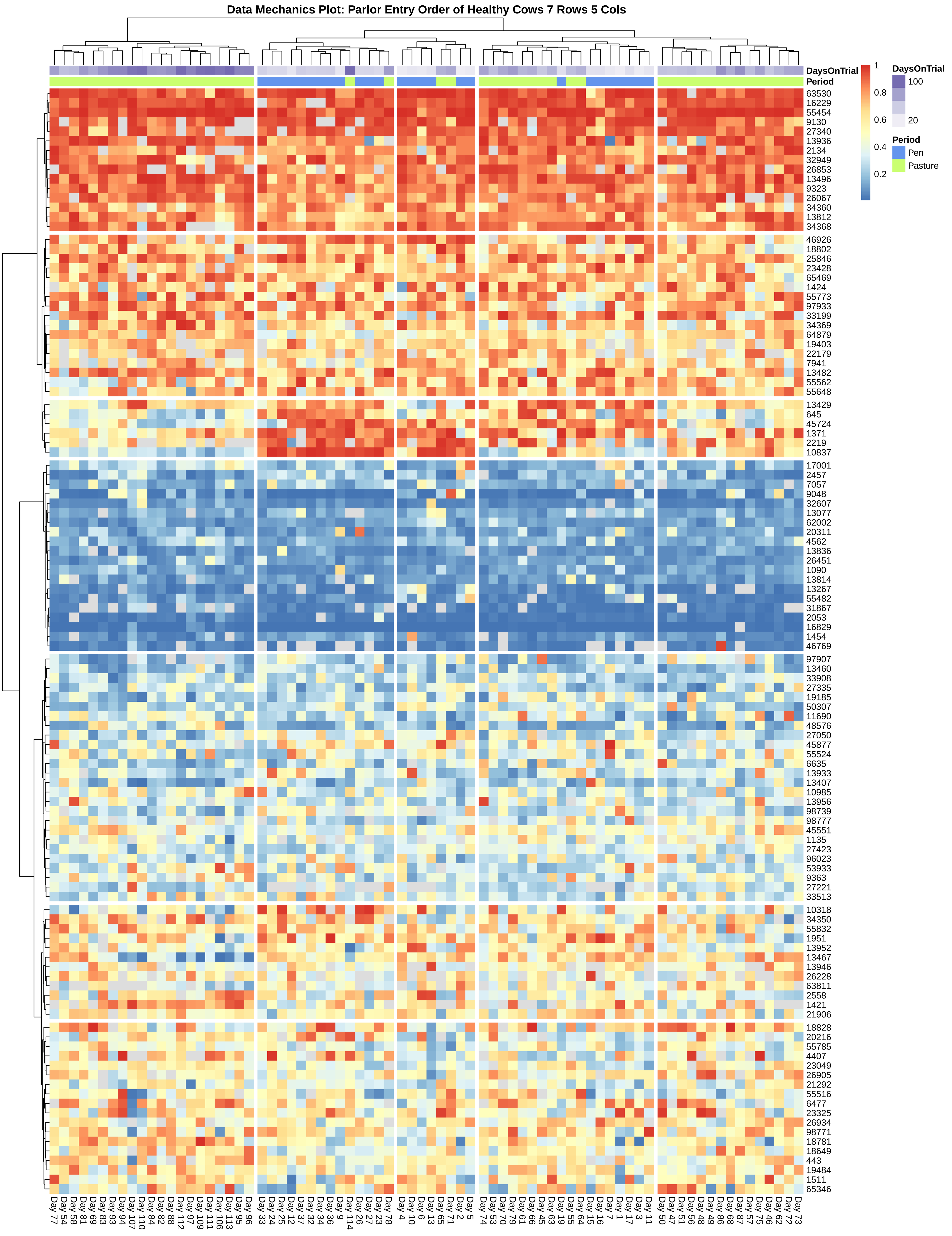
Data Mechanics Plot: Parlor Entry Order of Healthy Cows 7 Rows 5 Cols
DaysOnTrial
Period
Pen
Pasture

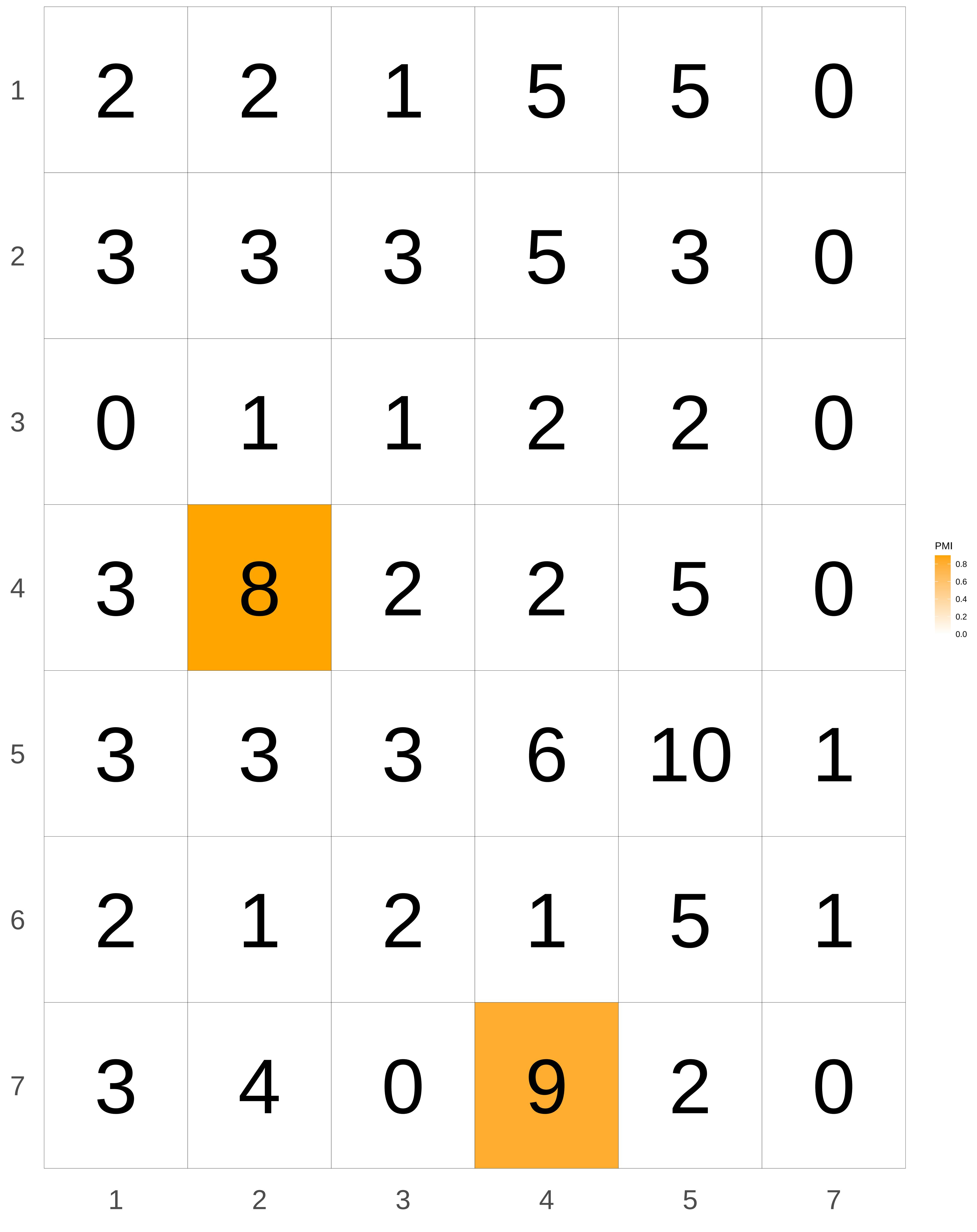
1 2 2 1 5 5 0
2 3 3 3 5 3 0
3 0 1 1 2 2 0
4 3 8 2 2 5 0
5 3 3 3 6 10 1
6 2 1 2 1 5 1
7 3 4 0 9 2 0
1 2 3 4 5 7
PMI
