# Supplementary material for: Livestock Informatics Toolkit: A Case Study in Visually Characterizing Complex Behavioral Patterns across Multiple Sensor Platforms, Using Novel Unsupervised Machine Learning and Information Theoretic Approaches
Source: Sensors (Basel). 2021 Dec 21;22(1):1. doi: 10.3390/s22010001 (PMC8747447; doi:10.3390/s22010001)
Supplement: Supplementary file 1 [file OTB_All_PP.pdf › sensors-1463895-supplementary/OverallTB/BivarTest_EntryOrder/OTB_All_PP.pdf]

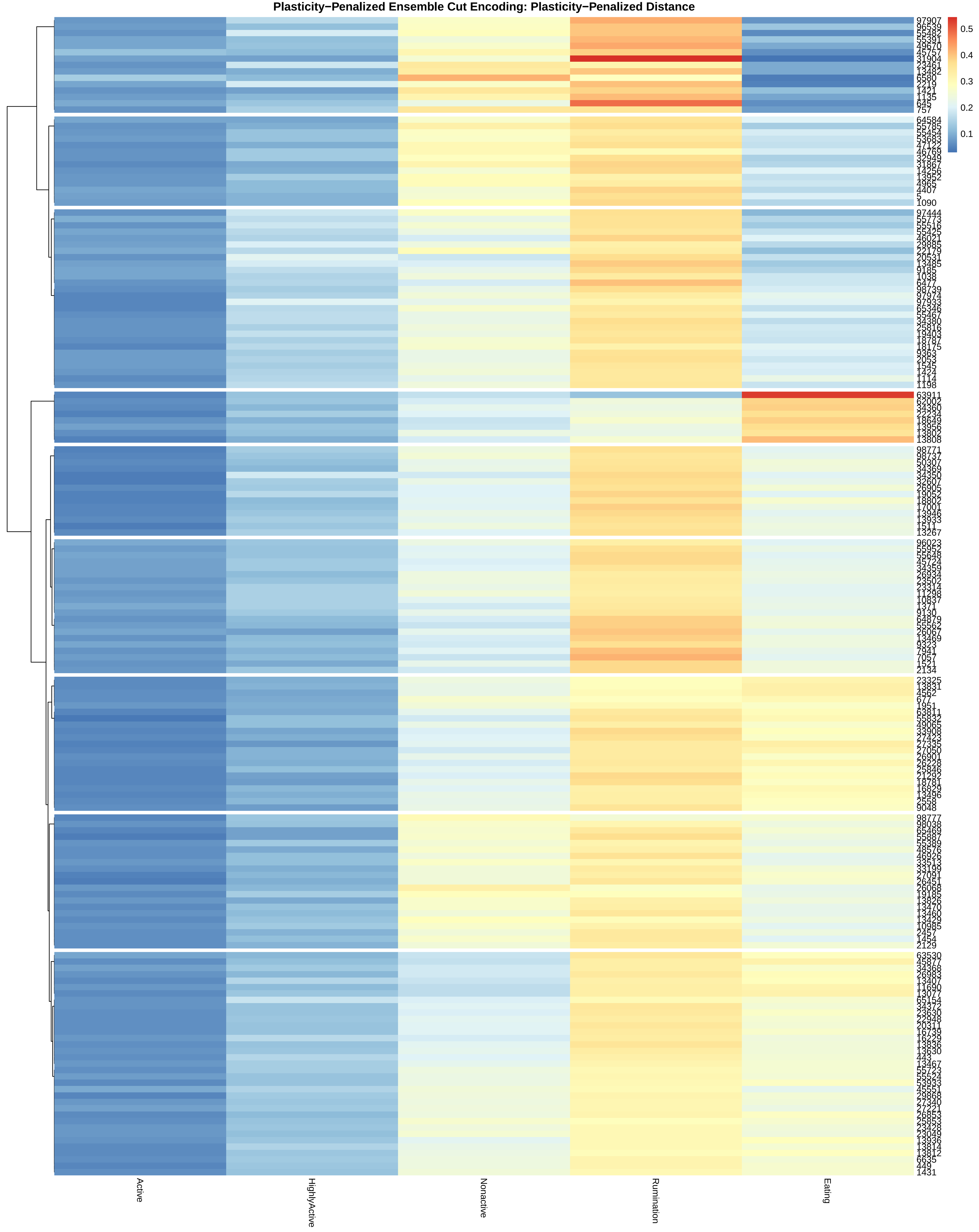
Plasticity−Penalized Ensemble Cut Encoding: Plasticity−Penalized Distance
Active
HighlyActive
NonActive
Rumination
Eating

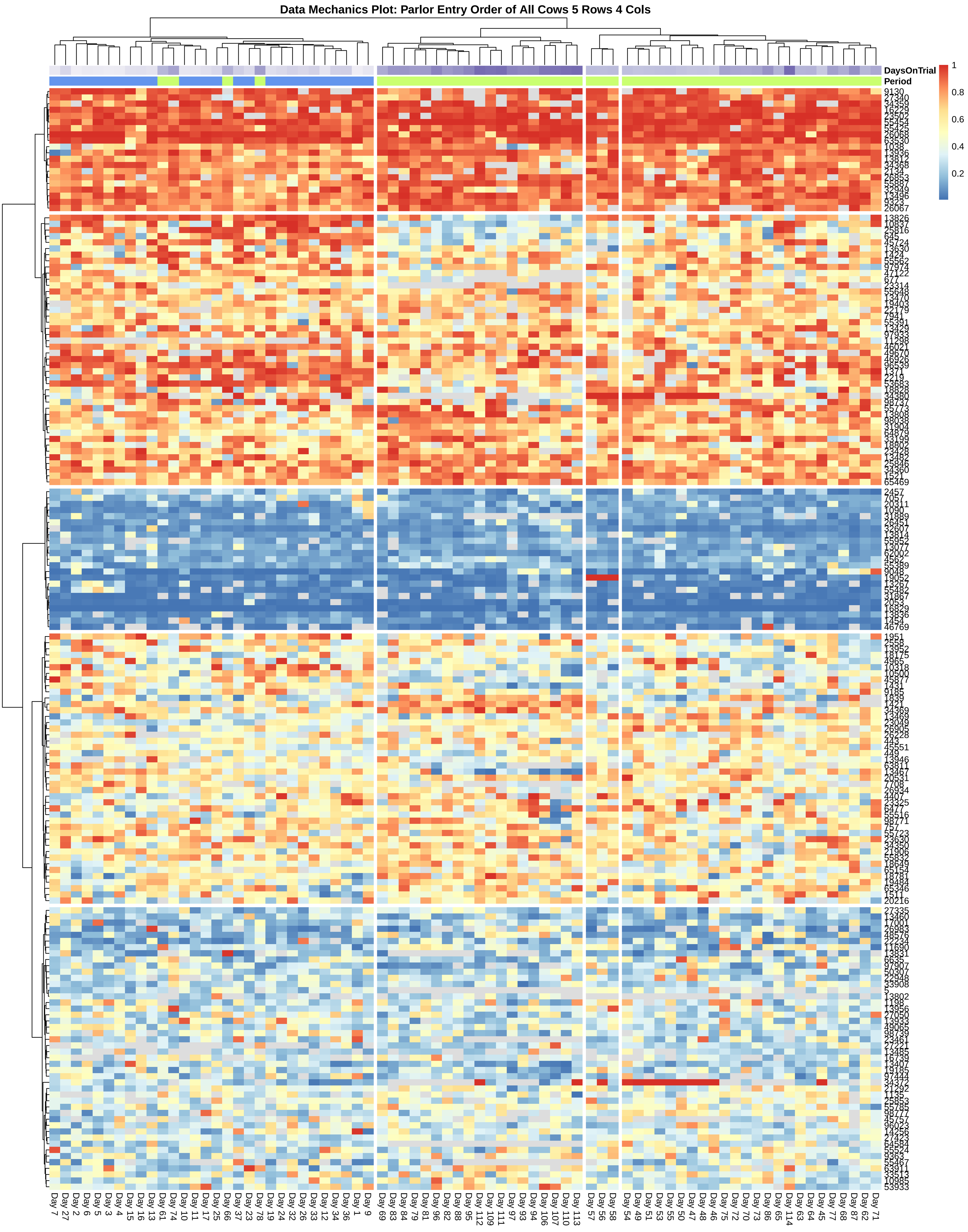
Data Mechanics Plot: Parlor Entry Order of All Cows 5 Rows 4 Cols
DaysOnTrial
Period

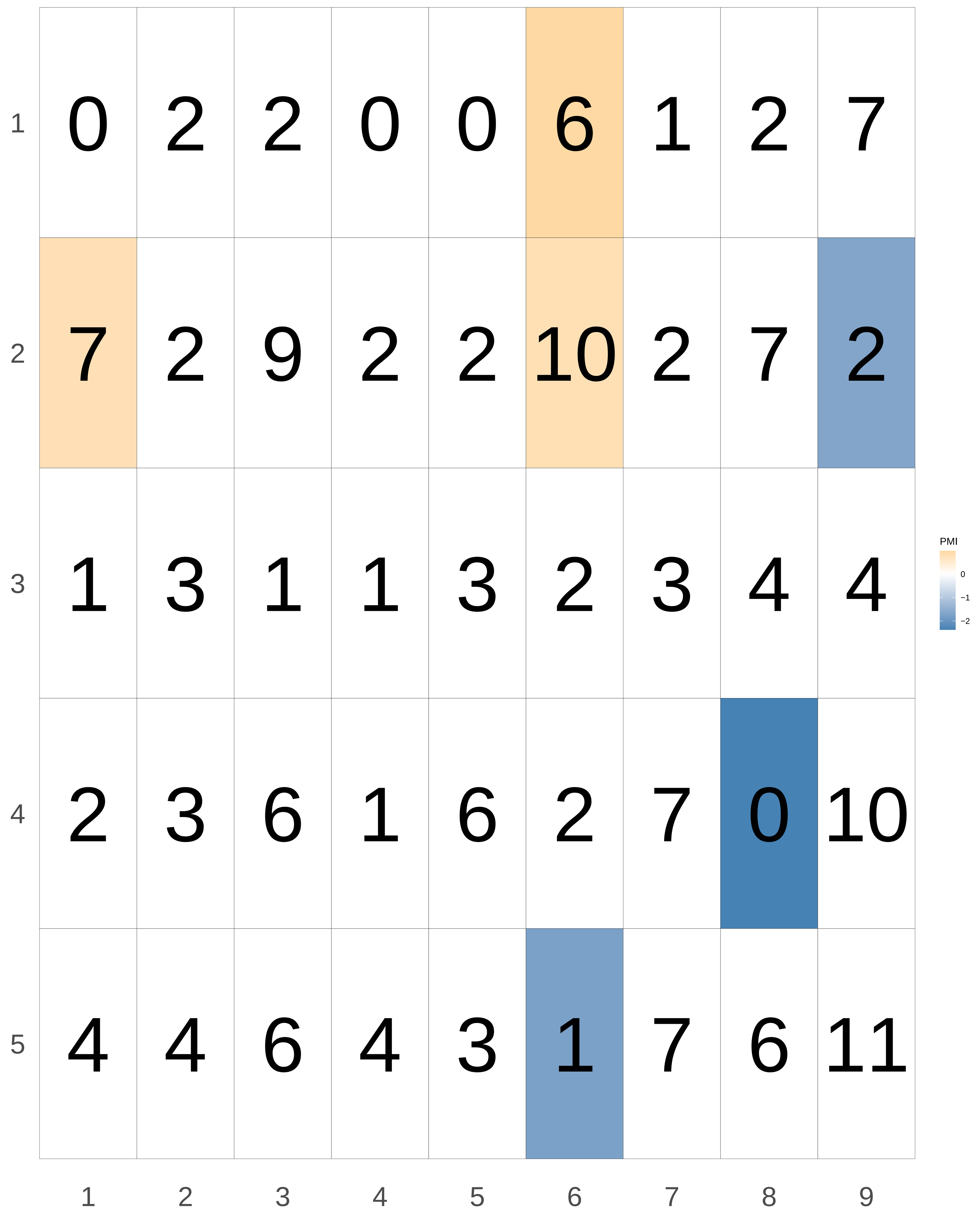
PMI
0 2 2 0 0 6 1 2 7
7 2 9 2 2 10 2 7 2
1 3 1 1 3 2 3 4 4
2 3 6 1 6 2 7 0 10
4 4 6 4 3 1 7 6 11
