# Supplementary material for: Livestock Informatics Toolkit: A Case Study in Visually Characterizing Complex Behavioral Patterns across Multiple Sensor Platforms, Using Novel Unsupervised Machine Learning and Information Theoretic Approaches
Source: Sensors (Basel). 2021 Dec 21;22(1):1. doi: 10.3390/s22010001 (PMC8747447; doi:10.3390/s22010001)
Supplement: Supplementary file 1 [file EO_HealthPasture.pdf › sensors-1463895-supplementary/OverallTB/BivarTest_Sick/EO_HealthPasture.pdf]

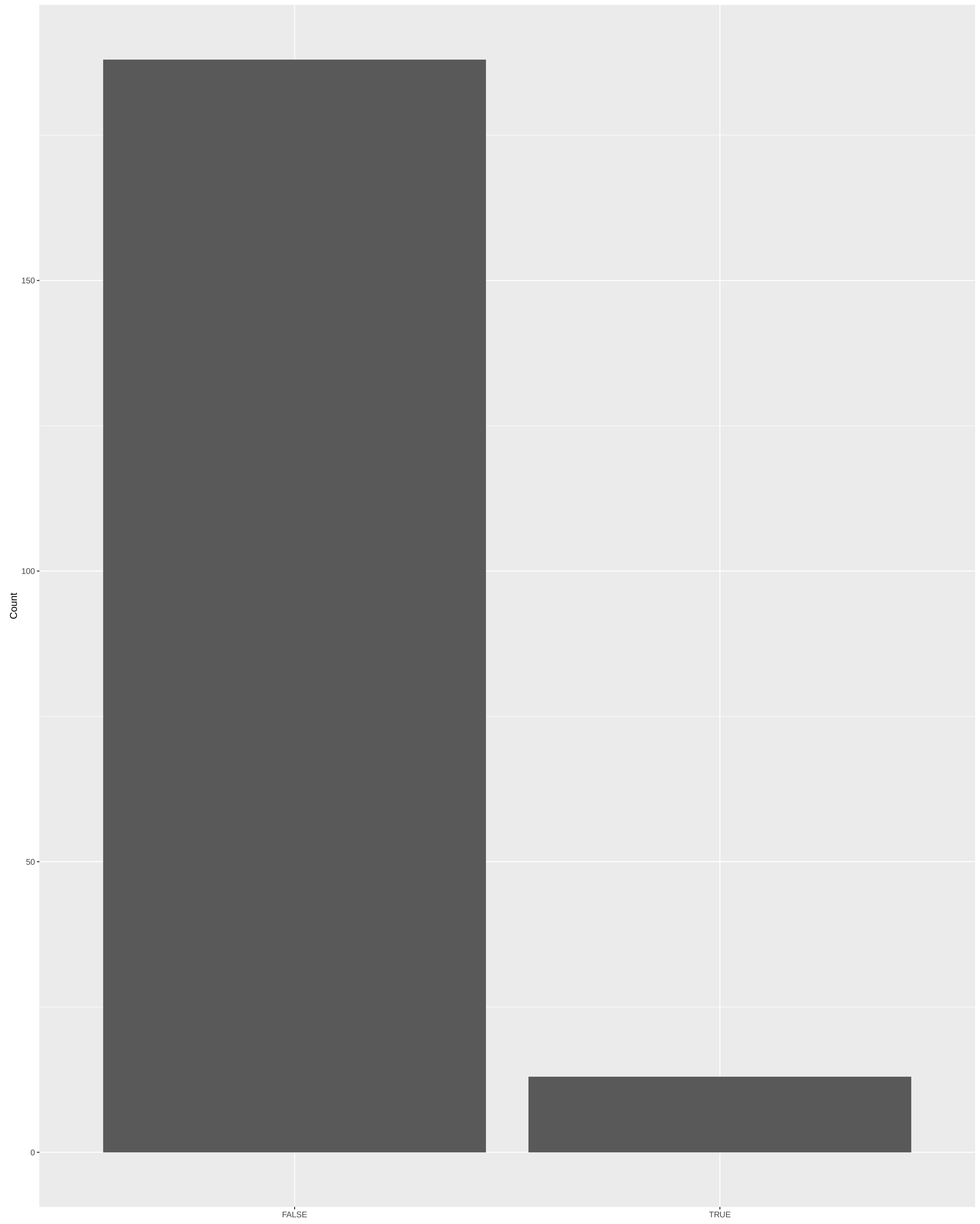
Count
150
100
50
0
FALSE
TRUE

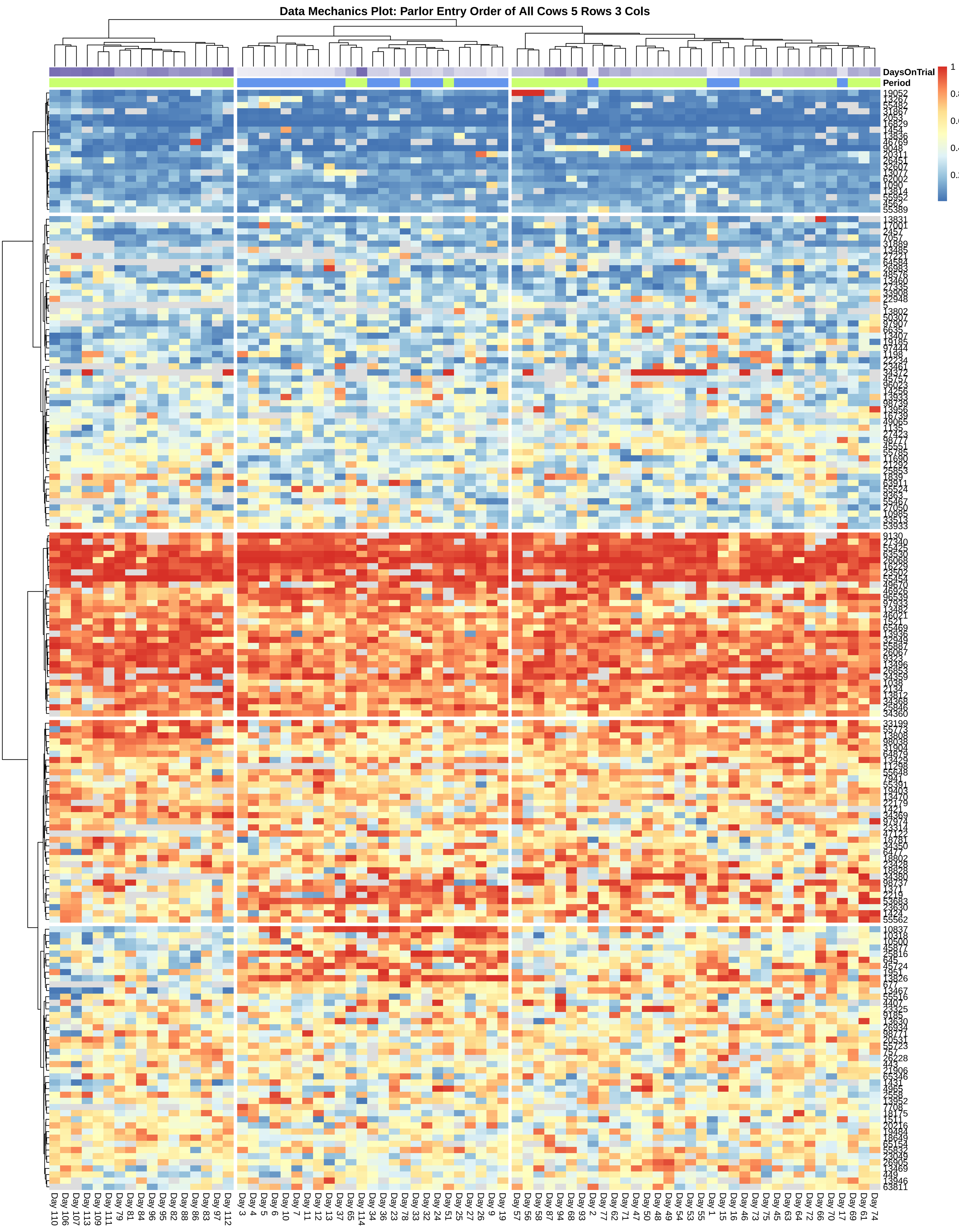
Data Mechanics Plot: Parlor Entry Order of All Cows 5 Rows 3 Cols
DaysOnTrial
Period
1
0.8
0.6
0.4
0.2

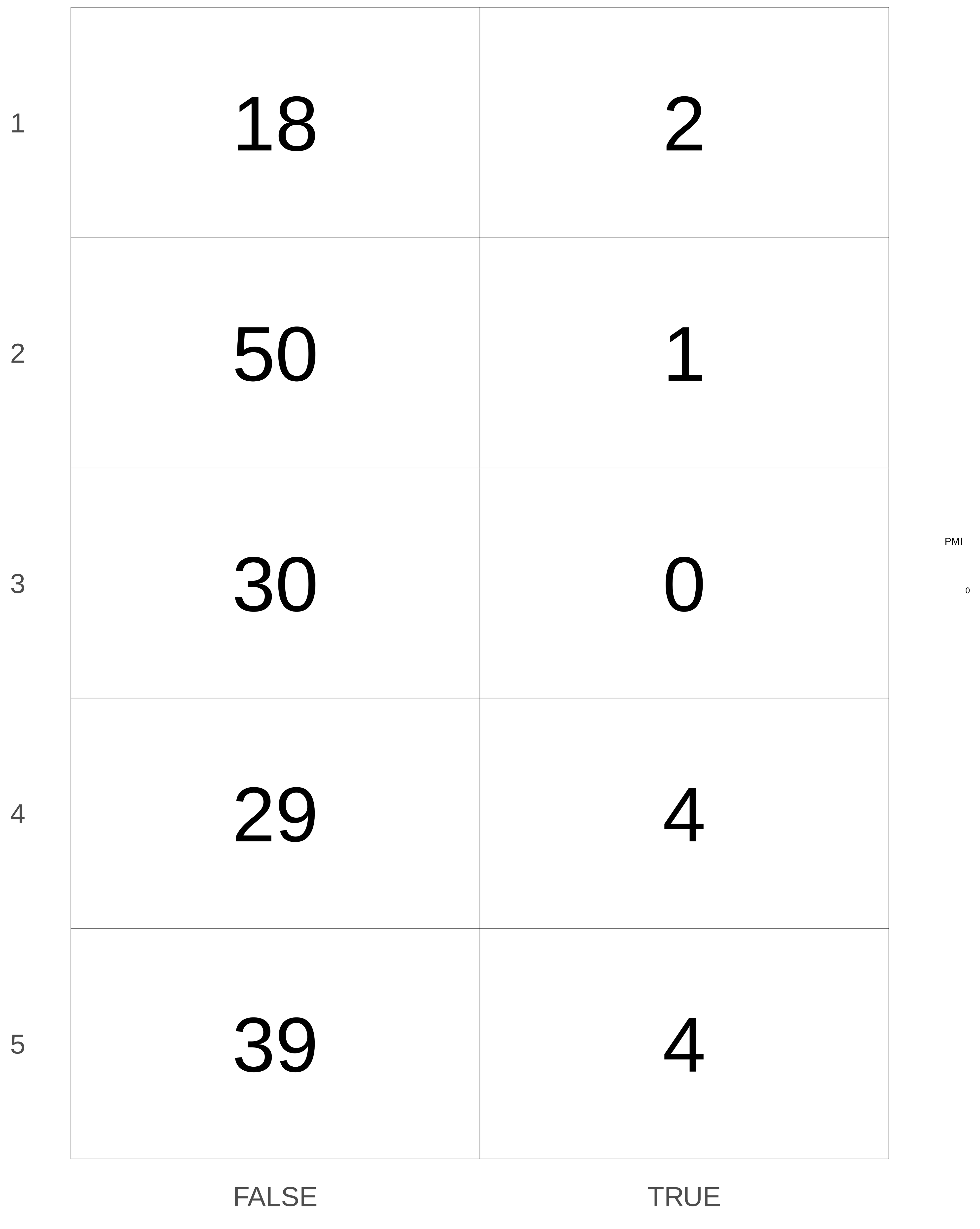
1
18
2
2
50
1
3
30
0
4
29
4
5
39
4
FALSE
TRUE
PMI
0
