# Supplementary material for: Livestock Informatics Toolkit: A Case Study in Visually Characterizing Complex Behavioral Patterns across Multiple Sensor Platforms, Using Novel Unsupervised Machine Learning and Information Theoretic Approaches
Source: Sensors (Basel). 2021 Dec 21;22(1):1. doi: 10.3390/s22010001 (PMC8747447; doi:10.3390/s22010001)
Supplement: Supplementary file 1 [file Euclid_vs_PW_5x5.pdf › sensors-1463895-supplementary/OverallTB/ContrastPlots/Euclid_vs_PW_5x5.pdf]

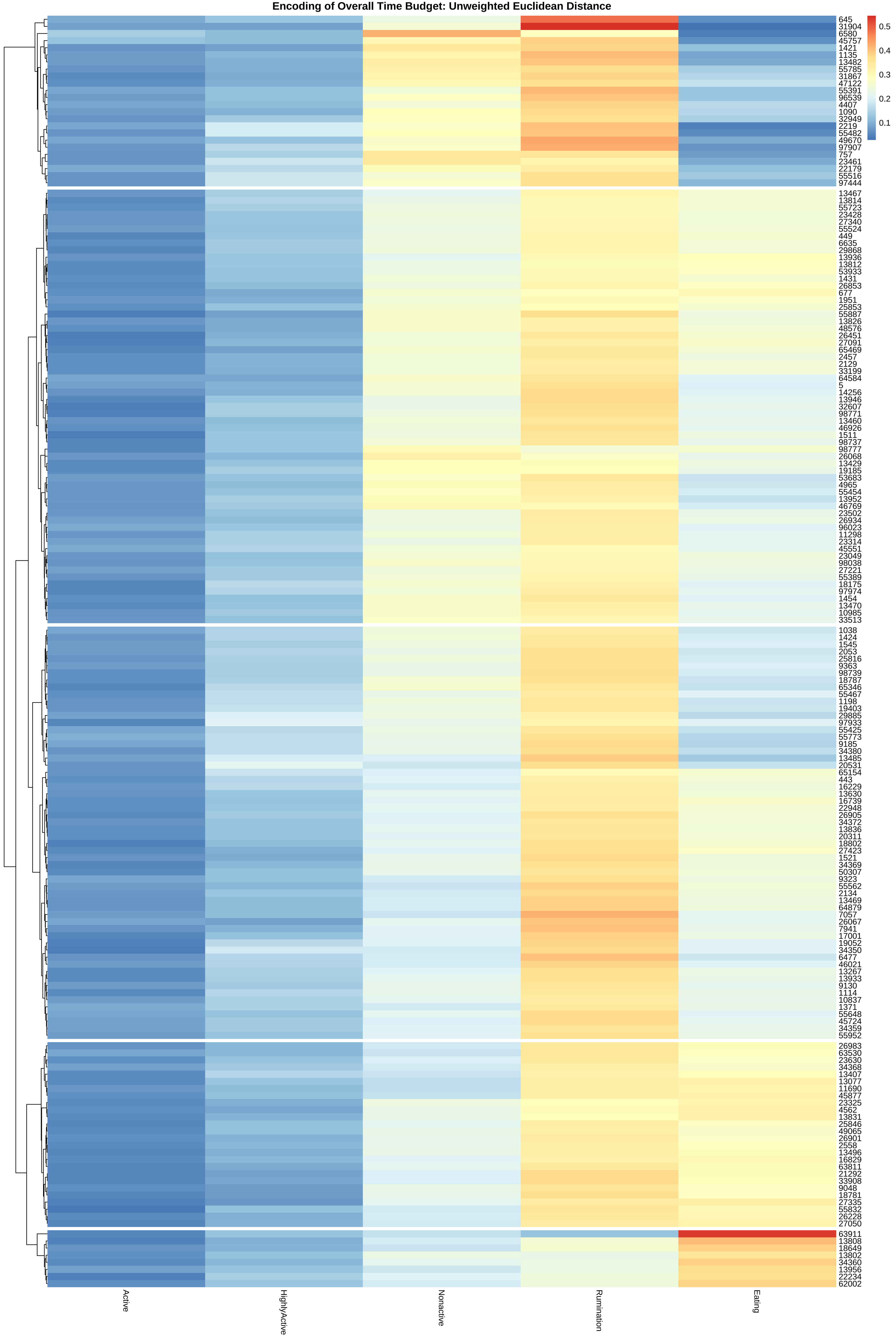
Encoding of Overall Time Budget: Unweighted Euclidean Distance
0.5
0.4
0.3
0.2
0.1
Active
HighlyActive
Nonactive
Rumination
Eating

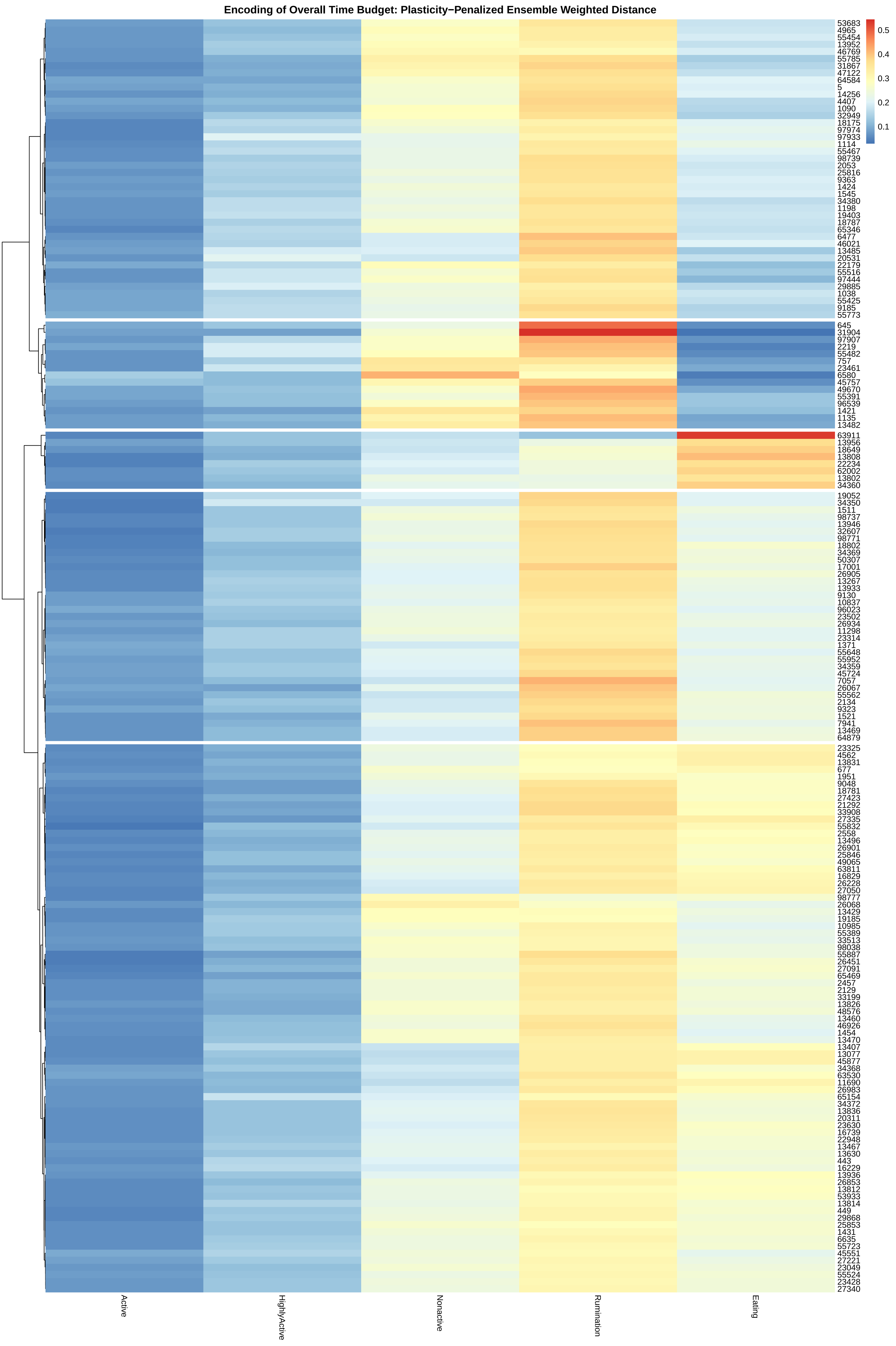
Encoding of Overall Time Budget: Plasticity−Penalized Ensemble Weighted Distance
0.5
0.4
0.3
0.2
0.1
Active
HighlyActive
Nonactive
Rumination
Eating

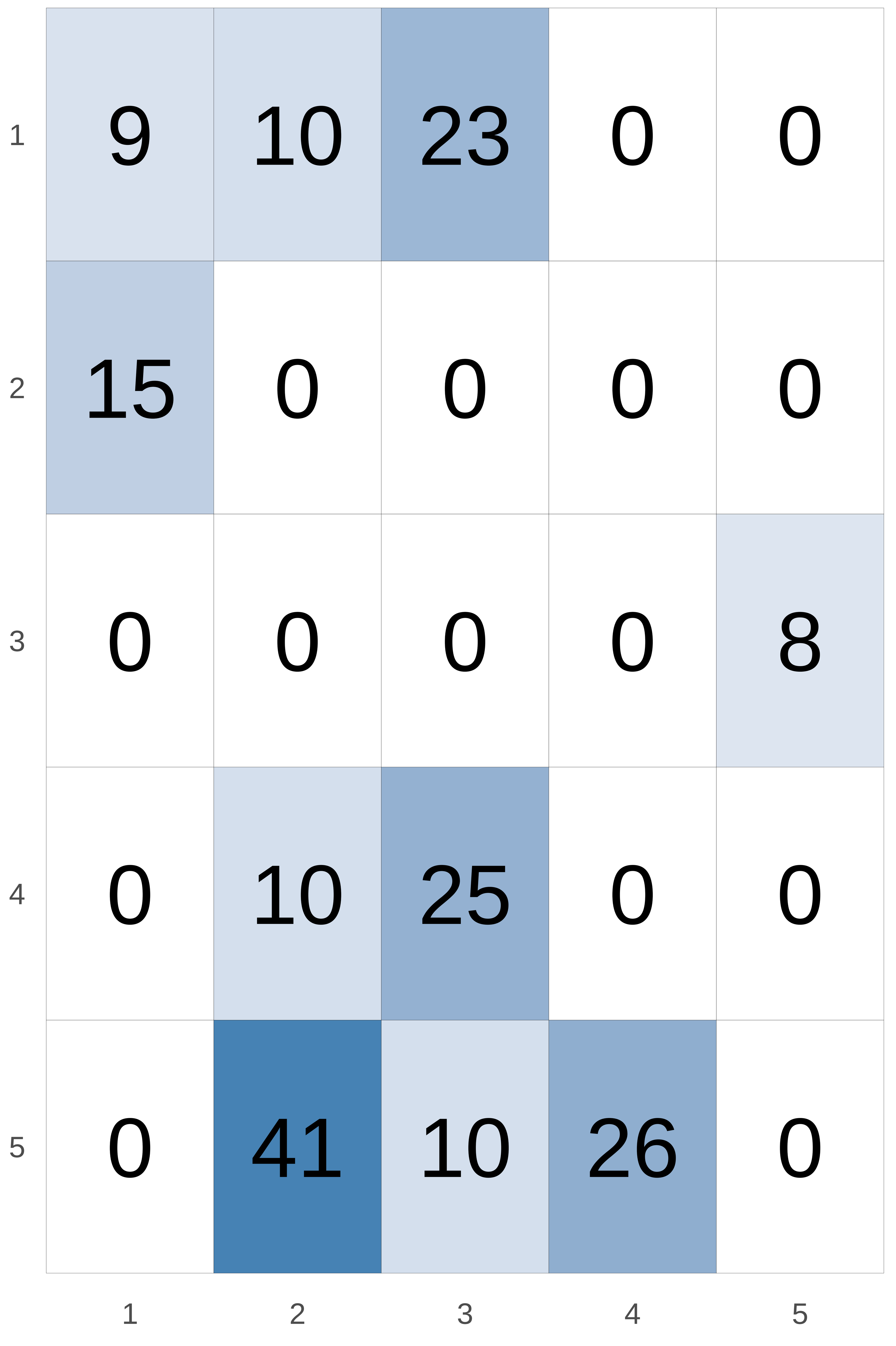
1
2
3
4
5
9 10 23 0 0
15 0 0 0 0
0 0 0 0 8
0 10 25 0 0
0 41 10 26 0
1
2
3
4
5
