# Supplementary material for: Livestock Informatics Toolkit: A Case Study in Visually Characterizing Complex Behavioral Patterns across Multiple Sensor Platforms, Using Novel Unsupervised Machine Learning and Information Theoretic Approaches
Source: Sensors (Basel). 2021 Dec 21;22(1):1. doi: 10.3390/s22010001 (PMC8747447; doi:10.3390/s22010001)
Supplement: Supplementary file 1 [file sensors-22-00001-s001.zip › sensors-1463895-supplementary/OverallTB/LIT_0.0.0.9001.tar.gz!/LIT/inst/doc/DMPlots.html]

Visualizing Heterogenous Behavioral Patterns in Repeated Measures Data Using Data Mechanics Plots


# Visualizing Heterogenous Behavioral Patterns in Repeated Measures Data Using Data Mechanics Plots

#### *Catie McVey*

#### *2021-10-20*

```
library(LIT)

library(dplyr)
library(ggplot2)
library(RColorBrewer)
```

# Introduction

In most data analyses, the biological system being scrutinized is assumed to be divisible into two distinct parts:

***observed value = signal + noise***

The signal component is any and all systematic differences between observations made on individuals within a population that can be attributed to intrinsic or extrinsic factors. After accounting for all these deterministic drivers of observational values, what remains is only noise, or purely stochastic inexplicable fluctuations in values.

In the context of observable behaviors that an animal displays, signal might relate to external factors affecting behavior such as weather, distribution of resources or barriers in the environment, social composition of the herd, etc. Signal may also capture intrinsic drivers of behavior such as personality, affective state, health status, etc. But even when all possible internal and external factors are taken into consideration, we still would not say that we can predict with complete certainty what behavior will be observed - like a coin flip, there is still a purely random (stochastic) component to how information will trickle its way through the neurons in an animal’s brain to generate a response. Measurement error also contributes to the noise component of any observation.

In model-based statistical analyses, we attempt to represent the deterministic component of a system by providing the model all factors that we anticipate my systematically influence observable behaviors. External factors affecting behaviors are often directly measured and incorporated into the model as a fixed effect. Internal factors often cannot be directly measured and may be accomodated in the model as a latent feature by taking repeated measures and fitting a random effect. Some external factors that may not be directly measurable may also be approximated using random effects. For example a farm effect may be used to collectively represent systematic differences between farms without measuring all environmental and managerial factors that may differ between them. All residual variation in observed values beyond the values that are predicted by the model representing the deterministic component of a system are then assumed to be attributed to noise.

Such models work well in experimental settings where a wide range of external factors can be held constant and where repeated measures are often measured over a tight temporal window so that latent intrinsic factors remain constant (stationary). But such assumptions do not always hold. When external factors that influence observed values cannot be held constant across trials and are not accomodated in the model via a fixed or random effect, systematic differences (heterogenity) will remain in residual values. This not only violates the IID sampling assumption on which many statistical evaluations (p-values, confidence intervals, credible intervales, etc) are contingent, but also risks overlooking important features of the system being studied. In data sets generated using sensor technologies, which often collect repeated measurements over extended periods of time, not only is there more opportunity for external vactors to vary, but internal factors, for example health status or affective state, may change over these extended windows of observations. If such latent variables cannot be assumed to be stationary, then a simple random intercept or random slope term may not suffice to capture such a dynamic component of the underlying signal.

*So how does one identify when such critical modeling assumptions have been violated?*

In unsupervised machine learning, model-free approaches are employed that attempt to systematically extract from the quagmire of a data set any robust systematic patterns, regardless of their source or cause, until only noise is left. Thus, such models represent a valuable tool to survey and explore all determinstic aspects of a system absent any prior assumptions or hypotheses. This might reveal the presence of extrinsic factors that may have been overlooked. Where repeated measures are taken over extended periods of time, such tools may also provide a means to assess the stationarity of behavioral patterns over time.

In this vignette we will demonstrate how data mechanics visualizations can be incorperated into statistical analyses to achieve both goals.

# Data Mechanics Explained

Data mechanics is conceptually quite similar to factor analysis, but neither requires that factors be linearly associated or that observations be independet. Instead, flexible hierarchical clustering techniques are used that leverage similarities between animals to reveal more complex nonlinear relationships between features.

Usually, an observation consists of all measures taken on an animal, and displayed on the row axis. On the column axis are multiple measurments collected on each animal. These might be repeated measurement of the same response variable measured at different time points (McVey et al 2020), or a collection of various (multivariate) measurements taken on the same animal that are thought to capture different aspects of some latent feature(s) of the animal (McCowan et al 2016).

Hierarchical clustering trees are used to represent systematic similarities and differences between animals and amongst features. Clusters identified on one axis are then used to re-weight similarity values calculated on the opposite axis, allowing transfer of information between axes. When thisre-weighting is repeated over several iterations, the trees on either axis will converge towards clusters wherein row-by-column blocks become more homogenous while differences between blocks are enhanced. When the data is subsequently visualized via a heatmaps constructed using these trees, systematic heterogenity within the data due to underlying deterministic drivers are easily seen as color differences between blocks. As the number of row and column clusters are varied, different elements of the underlying signal structure may come into resolution. In this way, just as one might scan a through a sample under a light microscope to better understand the resident microorganisms resident, data mechanics allow researchs to systematically search through large complex data sets to explore their deterministic structures.

For further technical details on the implementation of data mechanics algorithms, see Hsieh et al 2018.

# DM Plots with Experimental Models

First we will explore how data mechanics plots can be integrated into the analysis of a behavioral dataset of moderate size generated in a controlled experimental setting. To do so, we will expand upon an example from Section 6.4.3 from *Experimental Design for Laboratory Biologists: Maximising Information and Improving Reproducibility* by S.E. Lazic (2017), which is an excellent reference that we highly recommend for all readers striving towards robust analyses of complex biological systems.

We will be using the *locomotor* dataset availiable in the *labstats* package. This is data that was generated from a controlled experimental trial with rats. Rats were first injected with either a treatment or a control compound, and then placed into an open field testing arena. The total activity observed for each rat is recorded in 15 minute intervals over a 90 minute observation window. The goal of the statistical analyses is to determine if there is a significant effect of the drug treatment on locomotion patterns.

```
library(labstats)
data(locomotor)

summary(locomotor)
#>       drug         animal        time           dist        
#>  Control:138   Min.   : 1   Min.   :15.0   Min.   : 0.0000  
#>  Drug   :144   1st Qu.:12   1st Qu.:30.0   1st Qu.: 0.1983  
#>                Median :24   Median :52.5   Median : 0.5385  
#>                Mean   :24   Mean   :52.5   Mean   : 1.3541  
#>                3rd Qu.:36   3rd Qu.:75.0   3rd Qu.: 1.5330  
#>                Max.   :47   Max.   :90.0   Max.   :12.6150

locomotor[1:10,]
#>       drug animal time  dist
#> 1  Control      1   15 0.476
#> 2  Control      2   15 0.512
#> 3  Control      3   15 0.215
#> 4  Control      4   15 0.578
#> 5  Control      5   15 0.525
#> 6  Control      6   15 0.215
#> 7  Control      7   15 0.146
#> 8  Control      8   15 0.313
#> 9  Control      9   15 0.684
#> 10 Control     10   15 0.293
```

We’ll begin by plotting the data to visually compare the two treatment groups.

```
library(lattice)

xyplot(dist ~ factor(time)|drug, data=locomotor, group=animal,
       type=c("g","l"), between=list(x=1), 
       col="black", lty=1, 
       xlab="Time (min)", ylab="Locomotor Activity",
       strip=strip.custom(bg="lightgrey"), 
       scales=list(alternating=FALSE) )
```

As shown in Lazic (2017), when the lattice package is used to disagregate and visualize this data set, a clear difference between the treatment and control groups is immediately apparent. Activity rates amongst control animals remain low throughout the dureation of the trial, while there is a clear elevation in activity rates amongst treatment rats 15-30 minutes following their injection. Or is it clear? While the locomotion pattern amongst the control rats is quite uniform (homogenous), their is much greater range of locomotion response at all time points amongst the treatment rats. Is this response just more variable, or are their underlying patterns in this behavioral response that we are overlooking by only considering our treatment response.

To explore this question, we’ll visualize the data from the treatment rats using a data mechnics plots. This will first require, however, some reformatting of the data frame. Here the data is formatted to accomodate a model-based repeated measures analysis, wherein each row is an observational unit (ie - activity of one rat at one time point), and their is an indexing variable for each experimental unit (ie - each rat). Here we need each rat to be a row and their repeated observations to be aligned along the column axis. This can be done using the *unstack* function.

```
trtrats <- locomotor[locomotor$drug == 'Drug',]
locowide <- unstack(trtrats, dist~time)
rownames(locowide) <- paste('Rat',unique(trtrats$animal), sep = '')
names(locowide) <- c('15', '30','45','60','75','90')
locowide
#>          15     30     45     60    75    90
#> Rat24 0.252  2.328  6.504 10.028 9.937 4.324
#> Rat25 0.091  0.000  0.019  0.012 0.010 0.138
#> Rat26 0.235  0.591  1.429  1.901 2.409 2.421
#> Rat27 1.733  2.665  4.799  5.460 5.241 5.314
#> Rat28 0.031  0.067  0.088  0.721 1.236 0.611
#> Rat29 0.590  0.758  0.783  0.989 1.540 1.685
#> Rat30 0.511  1.975  6.186  7.044 6.244 5.995
#> Rat31 0.328  0.139  0.649  1.507 2.132 1.388
#> Rat32 0.117  0.545  1.254  1.713 2.284 2.687
#> Rat33 0.481  0.233  0.637  1.454 3.503 4.397
#> Rat34 0.161  0.321  0.333  1.167 0.756 0.865
#> Rat35 0.280  0.507  0.187  0.060 0.063 0.220
#> Rat36 0.494  0.139  0.110  0.047 0.098 0.072
#> Rat37 0.113  0.291  0.359  0.684 1.223 1.799
#> Rat38 0.917  1.718  4.764  7.153 7.669 7.705
#> Rat39 0.184  0.466  0.303  0.326 0.329 0.070
#> Rat40 1.540  6.496  8.377  3.310 3.730 2.765
#> Rat41 0.410  0.690  0.629  0.266 0.214 0.025
#> Rat42 1.000  1.429  0.133  0.560 1.202 0.988
#> Rat43 1.571  5.020  3.558  1.334 0.288 0.262
#> Rat44 1.106  9.902 10.522  0.339 0.126 0.000
#> Rat45 0.796  7.707  3.669  0.002 0.007 0.197
#> Rat46 0.066  0.070  0.880  2.541 4.329 3.323
#> Rat47 2.475 12.615  5.674  0.141 0.027 0.438
```

We can now pass this data frame directly into data mechanics plot. Note that here the only values contained in the data frame itself are locomotion values. Rat names are recorded as row names, not as a variable within the data frame, and time points are recorded as column names, which will in turn be used to label heatmap row and columns.

```
dmout <- dmplot(locowide, n_rowclusters = 3, n_colclusters = 4,
                showplot_all = F, showplot_final = T,
                plot_title = 'Rat Data Mechanics Plot')
```

Looking at this plot, we can see that colors are more uniform within- than between-blocks. This tells us that there is some deterministic feature beyond injection treament driving systematic heterogenity among observations in this data set.

Scanning from left to right across a given row, changes in color are indicative of systematic patterns across the column axis. In this example, that would mean that the locomotion patterns change over time, which we expected from our previous plot.

If we scan up and down, however, changes in color are indicative of systematic differences along the row axis. Here that means there are systematic differences remaining between rats after accounting for injection treatment. That means that residuals from our model only accounting for treatment effect will not be independently and identically distributed, which may skew our statistical inferences about drug effect. More importantly, it appears that we are potentially overlooking an important biological feature of this system that we didn’t anticipate in the original design of the experiment.

On closer inspection, however, we can see that changes in color between blocks along the time (column) axis are not the same between all groups of rats. This is indicative of a row-by-column interaction, which here means our temporal pattern is not homogenous across all rats after accounting for drug treatment. That means we cannot account for this element of signal in a model-based statistical analysis of this data set using only a fixed effect to account for change over a time variable

Instead, we see that our treatment rats are presenting three distinct locomotion patterns in response to our drug. One group of rats, starting with rat 40, demonstrate a quick surge in locomotion early in the trial and then return to being relatively inactive. Another group, starting with rat 24, take a bit longer to get moving, but then remain active throughout the duration of the trial. Our largest group, however, remain relatively inactive throughout the duration of the trial, similar to our control animals.

We can confirm the interpretations drawn from the heat map by appending the final cluster results along the row (rat) axis from the heatmap output to the data frame of treatment mice and again use the latice package to visualized their response curves

```
# converting from wide to long using gather

library(tidyr)

locowide$DMClust <- dmout$rowclust
locowide$animal <- rownames(locowide)
locotrt <- gather(locowide, 
                   time, dist, '15':'90',
                   factor_key=F)
locotrt$time <- as.numeric(locotrt$time)
locotrt[1:10, ]
#>    DMClust animal time  dist
#> 1        2  Rat24   15 0.252
#> 2        1  Rat25   15 0.091
#> 3        1  Rat26   15 0.235
#> 4        2  Rat27   15 1.733
#> 5        1  Rat28   15 0.031
#> 6        1  Rat29   15 0.590
#> 7        2  Rat30   15 0.511
#> 8        1  Rat31   15 0.328
#> 9        1  Rat32   15 0.117
#> 10       1  Rat33   15 0.481

# plot treatment subgroups using lattice

library(lattice)

xyplot(dist ~ factor(time)|DMClust, 
       data=locotrt, group=animal,
       type=c("g","l"), between=list(x=1), 
       col="black", lty=1, 
       xlab="Time (min)", ylab="Locomotor Activity",
       strip=strip.custom(bg="lightgrey"), 
       scales=list(alternating=FALSE) )
```

After a bit of data fiddling, we can confirm the results of data mechanics plot that there are three very distinct locomotion patterns amongst treatment mice. Clearly there is more going on in this system than just a uniform drug effect. Perhaps there was an issue in administering the drug. This might explain why half our treatment rats mirror our controls. Or perhaps we are seeing distinct intrinsic differences between rats with respect to metabolism of the drug, such as we might expect if multiple genetic lines or different sexes of mice were used. This might explain why in some mice the effect of the drug is more rapid but then rapidly fades away. See Lazic (2017) for further discussion of potential biological drivers.

Regardless of the source of these differences amongst treatment rats, this example clearly demonstrates the utility of data mechanics plots in fully exploring the deterministic structures of a repeated measures dataset, even in highly controlled experimental setting where we might be tempted to think our animals won’t be able to find a curve ball to throw at us.

# DM Plots with Sensor Data

Alright, we’ve seen how handy data mechanics can be on a nice small data set. Lets open up the throttle to see what it can do on a big sensor data set, and explore some of the bells and whistles that come with this plotting utility.

For this example, we’ll demonstrate how to partially reproduce the data mechanics plots generated in McVey et al 2020. This data set was collected as part of a comprehensive feed trial exploring the impact of a fat supplement on health, behavior, and prodcution outcomes in organic dairy cattle (Manriquez et al 2019). As part of this trial, entry order into the milking parlor was recorded each morning on 114 cows via a RDID reader over an 80-day observation window. During that period, cows made the transition from over-wintering in a free-stall barn with access to an outdoor drylot to spending their evenings on spring pasture.

We want to visually identify any and all non-random patterns in parlor entry position. Do cows enter to paror completely at random, or do they have consistent position within the queue. Does that position remain the same over the course of this extended trial? And do they appear to navigate this queue as individuals or is there evidence that they coordinate their movements with their friends? To explore these questions, we’ll start by visualizing the data frame containing only entry position percentiles (normalized to account for fluctuations in herd size due to reader errors and management).

## Exploring Cluster Metaparameters on a Grid

How many clusters should we divide our cows (row axis) into? How many clusters should we divide our days (column axis)? In the previous example, where we had only a handful of repeated observations and a relatively small subsample of rats, we could just fiddle with these meta-parameter values until we got a clear visualization. With this larger data set, we will need to scan through our cluster size options more systematically.

We might expect that different deterministic features will come into resolutions at different clustering grainularity. For example, if we think there is a clear binary distinction between cows that are keen to wake up andg o the parlor and cows that would perfer a few more minutes of beatuy sleep, we might only need a few row clusters to reveal clear distinctions between motivation to be milked. If we think that cows might want to travel to the parlor within their friend groups, we might need finer stratification to recover this deterministic driver of milking position.

The LIT package allows users to exlore the optimal combination of metaparameter values for row and column cluster numbers on a grid of values using the *dmplotGrid*. Users provide a vector of candidate row and column cluster size values, and dmplotGrid will generate a data mechanics plot for each combination of cluster number values on either axis.

There are two options availiable to explore these results. Where the grid being explored is quite large, we recomend users export the plots to jpeg files, and so this is the default option for this function. By defualt, plots will be exported to the current working directory (check using *getwd()*) . Alternatively, users can specify a filepath to the folder that heatmap files should be returned to as part of the filename option.

The following code will export jpeg files to a folder “Viz” within our project directory. Each jpeg file will start with the name EntryOrderPlot. Appended to the end of each filename will be the number of row and column cluster numbers used in a given plot. For example ‘EntryOrderPlot\_R4C2.jpeg’ will be the dmplot with 4 row clusters and 2 column clusters. We can adjust the width, height, and resolution of the image to acheive the proper image scale using dmplotGrid options. Any additional plotting options that can be passed to *dmplot()* can also be passed to *dmplotGrid*. In addition to exporting jpeg files to the specified directory, *dmplotGrid* will also return a list containing the output for each dmplot fit on the grid.

```
dmgrid_out <- dmplotGrid(eodat, 
                          n_rowclusters = 1:10, 
                          n_colclusters = 1:10,
                          filename = 'Viz/EntryOrderPlot',
                          imwidth = 10, 
                          imheight = 15, 
                          imres = 300)
```

Alternatively, when the number of metadata parameters to consider is not too large, the results can be printed directly to the screen using the *showplots* option

```
dmgrid_out <- dmplotGrid(eodat, 
                          n_rowclusters = c(3,6), 
                          n_colclusters = c(2,3),
                          export_plots = F,
                          showplots = T)
```

Scanning up and down along the row (cow) axis of these plots, there is clear differences in color values between blocks. That tells us that cows are not going into the parlor randomly, but that individual cows are more likely to be found in certain sections of the queue than others. The blue band, which corresponds to cows at the front of the queue, appears particularly uniform in color (homogenous). This might reflect a clear consistent preference for queue position amongst these cows. Alternatively, these cows might maintain a stable social position within this closed group of animals wherein they are expected to lead the herd.

Whith finer stratification along the row axis, a clear group of straggler (red) cows also emerges that are also reasonably consistent in their entry position. The remaining cows occupy sections of the middle of the queue, but the greater variability in color value suggest that these positions are not as clearly defined.

Scanning left to right along the column axis, with a cluster size of only two, the colors seem relatively uniform within each row cluster. As we increase the stratification to three columns, we see that when row cluster are also more finely stratified, there is at least one group of cows whose entry positions change over time from nearer the end of the queue to closer to the front of the middle section of the queue. While the other bands remain reasonably homogenouss across time, it is clear from this last plot that entry order patterns are not stationary for all subgroups within this herd! Further, that this is a cluster of cows that appears to have moved from one position in the queue to another at roughly the same time might provide evidence that cows are not moving as individuals but as part of smaller cliques within the larger herd.

## Adding Auxilliary Data to Row and Colum Axes

We’ve identified some nonstationarity amongst a subset of our cows using data mechanics visualizations, but what factors are driving this pattern of behavior? Is this shift a response to a change in their environment? And are there intrinsic factors about this collection of cows priming them to respond to these external factors while other animals in the herd remain fairly stationary in their queue position?

On a working dairy, it is not only impossible to hold all environmental factors constant over such an extended window of observation, but it is even difficult to measure all uncontrolled variables that may be influencing the response. The beauty of data mechanics visualizations is that we can identify systematic heterogenity in a data set even when we have failed to quantify its source. But if we do have access to some control variables, we certainly would want to include this information in our data mechanics visualizations. This will help us identify any patterns in our response that might be partially explained by control variables, information that we may want to incorperate into any subsequent model-based evaluations of the biological system at hand.

The *pheatmap* package used to create heatmap visualizations for our data mechancis plots offers a range of plotting options that can be used to add color annotations to observations on both the row and column axis. Here we will demonstrate how these options can be passed through the *dmplot()* function to augment our plots. To do so we will need two addtional data frames.

We’ll begin with *dateaux*. This data frame contains two variables. The first is a continous integer variable indicating the day on which a milking order observation was made relative to the date that all cows had entered the milking herd. The second variable is a factor with two levels indicating whether cows left for the parlor from their home pen or from their spring pasture. **It is critical that the row names given to the data frame containing the auxiliary column information matches the column names of the data frame being passed into *dmplot()*.**

```
str(dateaux)
#> 'data.frame':    80 obs. of  2 variables:
#>  $ Period     : Factor w/ 2 levels "Pasture","Pen": 2 2 2 2 2 2 2 2 2 2 ...
#>  $ DaysOnTrial: int  1 2 3 4 5 6 7 9 10 11 ...
names(eodat)[1:10]
#>  [1] "Day 1"  "Day 2"  "Day 3"  "Day 4"  "Day 5"  "Day 6"  "Day 7"  "Day 9" 
#>  [9] "Day 10" "Day 11"
dateaux[1:10,]
#>        Period DaysOnTrial
#> Day 1     Pen           1
#> Day 2     Pen           2
#> Day 3     Pen           3
#> Day 4     Pen           4
#> Day 5     Pen           5
#> Day 6     Pen           6
#> Day 7     Pen           7
#> Day 9     Pen           9
#> Day 10    Pen          10
#> Day 11    Pen          11
```

Next we have *cowaux*. Again each row of the auxiliary data frame is given the same name as the corresponding obsevervation (row) in the data frame passed to dmplot(). Here, we’ve got some information on additional cows not considered in these analyse, but *pheatmap* will match the entery order data to the correct cow information using the matching row names.

```
str(cowaux)
#> 'data.frame':    136 obs. of  2 variables:
#>  $ Treatment  : Factor w/ 2 levels "Control","Organilac": 1 1 2 1 1 1 2 2 2 2 ...
#>  $ MilkYield95: num  81.8 80.5 86.8 101 73.1 ...
rownames('eodat')[1:10]
#> NULL
cowaux[1:10,]
#>       Treatment MilkYield95
#> 1025    Control      81.760
#> 10318   Control      80.530
#> 10837 Organilac      86.835
#> 1090    Control     101.000
#> 10985   Control      73.075
#> 11298   Control      70.925
#> 1135  Organilac      88.720
#> 11690 Organilac      69.425
#> 13077 Organilac      72.335
#> 13267 Organilac      71.100
```

Again, we’ve got one variable formatted as a factor with two levels to indicate the feed treatment that a cow recieved. The other variable is continous, and is the 95th percentile of a cows daily milk yeild, which is here used to approximate peak production. We could visualize this as a continous variable, but in performing a quick histogram visualization, we see that this variable is multimodal and contains outliers.

```
qplot(cowaux$MilkYield95, geom="histogram", xlab = 'Peak Milk Yeild', ylab = '', main = 'Distribution of Herd Productivity', col=I("black"), fill=I("blue"), alpha=I(.5), bins = 30) 
#> Warning: Removed 1 rows containing non-finite values (stat_bin).
```

With such a wide nominal range, this finer multimodal structure (and any unmeasured deterministic factors driving this departure from a simple bell curve) can be lost visually. To extract the finer geometric feature of this histogram, we will instead demonstrate how to discretize this data into an ordinal variable with cutoffs that we will determine empirically using a hclust tree.

```
# create data frame with Cow ID and continous milk yield variable
tempMY <- data.frame(CowID = rownames(cowaux), MilkYield95 = cowaux$MilkYield95) 
rownames(tempMY) <- rownames(cowaux) # add row names
tempMY <- tempMY[!is.na(tempMY$MilkYield95),] # remove missing values

# create hclust tree of milk yield values
hcout <- hclust(dist(tempMY$MilkYield95), method = 'ward.D2')
plot(hcout, xlab = 'Peak Milk Yeild')
```

```
# discretize data
nclust = 7
tempMY$MilkYield95C <- cutree(hcout, nclust)

#Create a intuitive category name using max and min milk yeild values for each cluster 
for(i in 1:nclust){
  
  temp2 <- subset(tempMY, tempMY$MilkYield95C == i)
  stringtemp <- paste(round(min(temp2$MilkYield95),1), 'lb-', 
                      round(max(temp2$MilkYield95),1), 'lb', sep = '')
  
  tempMY$MilkYield95C[tempMY$MilkYield95C == i] <- stringtemp
  
}

# order categorical variable

tempMY <- tempMY[order(tempMY$MilkYield95),]
tempMY$PeakYield <- factor(tempMY$MilkYield95C, 
                           levels =   unique(tempMY$MilkYield95C),
                           ordered = T)

# add categorical variable to cowaux
cowaux$CowID <- rownames(cowaux)
cowaux <- merge(cowaux, tempMY[,c('CowID','PeakYield')], by = 'CowID', all.x = T)
rownames(cowaux) <- cowaux$CowID
cowaux <- cowaux[, c('Treatment', 'PeakYield')]

cowaux[1:10, ]
#>       Treatment     PeakYield
#> 1025    Control 74.6lb-82.9lb
#> 10318   Control 74.6lb-82.9lb
#> 10837 Organilac 84.5lb-93.5lb
#> 1090    Control    95lb-101lb
#> 10985   Control 52.8lb-73.1lb
#> 11298   Control 52.8lb-73.1lb
#> 1135  Organilac 84.5lb-93.5lb
#> 11690 Organilac 52.8lb-73.1lb
#> 13077 Organilac 52.8lb-73.1lb
#> 13267 Organilac 52.8lb-73.1lb
```

Alright, now that we’ve done all that data wrangling, lets make some data mechanics plots! We can directly control the colors used to annotate the row and column axes by creating a named list with each of the auxiliary variables being considered, which must match the names used in the corresponding auxiliary data frame. To each variable label is attributed a vector of color values. If the variable is a factor, vector components must have names corresponding to all levels of the variable. We can create such vectors with these color-label associations ourselves, as shown here with Treatment and Period. Where a variable is divided into a larger number of levels, we can use *RColorBrewer* to assign color values to each level of the scale using predefined pallets, as whown here with PeakYield. For continous variables *RColorViewer* can also be used to create a continous color scale from predefined palletes, as shown here with the Days variable. For additional information on adding collor annotations to row and column margins see ?pheatmap

```
library(RColorBrewer)
col_list <- list(PeakYield = 
                setNames(
                  brewer.pal(nlevels(cowaux$PeakYield),"Reds"),
                  levels(cowaux$PeakYield)),
                Treatment  = c('Control' = 'cornflowerblue',
                               'Organilac' = 'gold'),
                Period = c('Pen' = 'cornflowerblue', 
                           'Pasture' = 'darkolivegreen1'),
                DaysOnTrial  = brewer.pal(dateaux$DaysOnTrial, "Purples")
                       )
#> Warning in if (n < 3) {: the condition has length > 1 and only the first element
#> will be used
#> Warning in brewer.pal(dateaux$DaysOnTrial, "Purples"): minimal value for n is 3, returning requested palette with 3 different levels

dmout <- dmplot(eodat,
                n_rowclusters = 7,
                n_colclusters = 4,
                export_plot_final = F,
                plot_title = 'Entry Order DM Plot',
                annotation_row = cowaux, 
                annotation_col = dateaux,
                annotation_colors = col_list)
```

In contrasting the patterns recovered from the data mechanics plots with our auxiliary variables, we see that there is quite a bit of structure on the time (column) axis. Looking first at the Period variable, there appears to be a clear if imperfect division between entery order observations recorded while the cows remained over-wintered in their free stall barn and those recorded after they were granted overnight access to pasture. Contrasting this pattern along the column axis with the row cluster beginning with Cow 45724, which demonstrates a nonstationary entry position, we see that cows in this group tended to come in towards the end of the herd when being sent to the parlor from their home pen, but during the pasture period moved closer to the front of the herd.

With this pattern in mind, we look at the control variables for these animals. We see that all but one of the animals in this group are on the fat supplement treatment, and that overall these are cows demonstrate moderate-to-low peak yeild values. Perhaps these animals are somewhat less energy deficient? In the pen they might have been less motivated by the promise of a post-milking ration to wake up, whereas during the pasture period they may have had more pep in their step to reach the parlor ahead of their herdmates? Or perhaps the lower yields are indicative of physically smaller animals, which could not effectively jockey for position in the mob coming from their pen, but may have had the time and space to manuver into a more preferable during the long walk in from the pasture. Or perhaps the low peak yeild is indicative of subacute tansition diseases that never manifested into a full-blown detectable illness with the higher plane of nutrition availiable to treatment cows. Such a subacute condition might explain why these animals were reluctant to rise and move into the parlor in the morning during the first few weeks of the trial, and explain why their motivation to be milked increased later in the trial when energy deficits were less severe.

Such explanations are ultimately only speculations. Any and none of these explanations might contribute to the nonstationary behavior of this anomolous group of animals with nonstationary entry positions. But they do indicate that caution might be warranted in incorperating these animals into models that seek to explain drivers of entry position across the broader herd.

Looking also at the Days on Trial variable, we see quite a bit of heterogenity across column clusters, and even some color gradients within clusters. This pattern is detectable not only across the pen-to-pasture transition, but also within these subperiods. This seems to indicate that the entry order assumed by the herd on a given day is much more similar to the entry position observed the next day than several days removed, and that this loss of similarity appears to be progressive. This may indicate that there is a degree of memory in this system at the herd level (though there is no overwhelming evidence of autocorrelation at the individual animal level). This result, combined with the heterogenous entry patterns across clusters, likely underscores the nonindepenence of animals as they move into the parlor. While it may seem intutive that social interactions would be important as cows jockey for queue positions, the highly systematic nature has serious implications for the statistical inferences drawn from linear modeling analyses of this system, which are discussed at greater length in McVey et al (2020).

# References

Hsieh F, Liu S-Y, Hsieh Y-C, McCowan B. From patterned response dependency to structured covariate dependency: entropy based categorical-pattern-matching. PLoS ONE. (2018) 13:1–28. doi: 10.1371/journal.pone.0198253

McCowan B, Beisner B, Bliss-Moreau E, Vandeleest J, Jin J, Hannibal D, et al. Connections matter: social networks and lifespan health in primate translational models. Front Psychol. (2016) 7:433. doi: 10.3389/fpsyg.2016.00433

McVey C, Hsieh F, Manriquez D, Pinedo P and Horback K (2020) Mind the Queue: A Case Study in Visualizing Heterogeneous Behavioral Patterns in Livestock Sensor Data Using Unsupervised Machine Learning Techniques. Front. Vet. Sci. 7:523. doi: 10.3389/fvets.2020.00523

MANRIQUEZ, D., CHEN, L., MELENDEZ, P. and PINEDO, P. (2019). The effect of an organic rumen-protected fat supplement on performance, metabolic status, and health of dairy cows. BMC Veterinary Research 15 450. https://doi.org/10.1186/s12917-019-2199-8
