# Supplementary material for: Livestock Informatics Toolkit: A Case Study in Visually Characterizing Complex Behavioral Patterns across Multiple Sensor Platforms, Using Novel Unsupervised Machine Learning and Information Theoretic Approaches
Source: Sensors (Basel). 2021 Dec 21;22(1):1. doi: 10.3390/s22010001 (PMC8747447; doi:10.3390/s22010001)
Supplement: Supplementary file 1 [file sensors-22-00001-s001.zip › sensors-1463895-supplementary/OverallTB/LIT_0.0.0.9001.tar.gz!/LIT/inst/doc/Tubeplots.html]

Visualizing Temporally Complex Behavioral Patterns Using Tubeplots


# Visualizing Temporally Complex Behavioral Patterns Using Tubeplots

#### *Catie McVey*

#### *2021-10-20*

```
library(LIT)

library(plotly)
library(dplyr)
library(ggplot2)
```

# Introduction

When behaviors are recorded by direct observation, there are practical limitations on the number of observations that can be generated (ie - grad students have to grduate eventually). As a result, where such studies must also consider the influence of time on the expression of animal behaviors, researchers are typically forced to focus on either cyclical patterns or longitudinal trends, but seldom are able to study both concurrently.

With livestock sensor technologies, observations may be logged at high granularity, on some platforms as frequently as every minute, over extended time periods that may last weeks, months, or possibly even years. With such datasets it becomes possible to not only incorperate both temporal axes into statistical analysis of behaviors, but also to explore potential interactions between these two factors.

Indeed, on a working farm environment where sensor technologies are often employed as a less invasive means of collecting behavioral data than an omnipresent grad student lurking in odd places, we should not expect that environmental and managerial factors can be held constant over extended periods of time. That means we also should automatically assume that cyclical patterns in behavior will remain stationary. Failure to incorperate such an interaction effect where one exists could certainly serve to obscure interesting behavioral patterns. Where time is used as a control variable for other unmeasured facets of the environment, ommitting such an interaction from statistical analyses could also lead to under-powered or misleading results.

But how can we effectively visualize such complex temporal dynamics when we have thousands of data points on every animal? In this vignette we’ll show how tube plots, in conjunction with more conventional marginal mean plots, can be used to understand the temporal dynamics of a large collection of behavioral observations from an on-animal sensor.

# Univariate Analysis of an Individual Animal

We’ll first explore the eating patterns of a single cow recorded via a HerdManager ear tag accelerometer (Agis Automatisering BV, Harmelen, Netherlands). This data was collected as part of a large comrehensive feed trial, procedural details for which can be found in Manriquez et al (2019). For more comprehensive analysis of this data in the context of the larger herd level, see McVey et al (2020).

To use tubeplot, the data frame should be formated so that each row represents an observation at a unique timepoint. One column should contain the cyclical time index of each observation as a numeric value. One column should contain the longitudinal time index of each observation as a numeric value. And one column should contain the observed value, which can be either numeric or categorical.

```
str(cowdat)
#> 'data.frame':    1008 obs. of  6 variables:
#>  $ TimeStampF   : Factor w/ 1008 levels "2017-03-12 00:00:00",..: 1 2 3 4 5 6 7 8 9 10 ...
#>  $ TimeDiff     : num  0 3600 7200 10800 14400 18000 21600 25200 28800 32400 ...
#>  $ Date         : int  17237 17237 17237 17237 17237 17237 17237 17237 17237 17237 ...
#>  $ Hour         : int  0 1 2 3 4 5 6 7 8 9 ...
#>  $ MinEat       : int  0 3 2 0 0 6 18 6 0 4 ...
#>  $ HourCorrected: num  19 20 21 22 23 0 1 2 3 4 ...

cowdat[1:10,]
#>             TimeStampF TimeDiff  Date Hour MinEat HourCorrected
#> 1  2017-03-12 00:00:00        0 17237    0      0            19
#> 2  2017-03-12 01:00:00     3600 17237    1      3            20
#> 3  2017-03-12 02:00:00     7200 17237    2      2            21
#> 4  2017-03-12 03:00:00    10800 17237    3      0            22
#> 5  2017-03-12 04:00:00    14400 17237    4      0            23
#> 6  2017-03-12 05:00:00    18000 17237    5      6             0
#> 7  2017-03-12 06:00:00    21600 17237    6     18             1
#> 8  2017-03-12 07:00:00    25200 17237    7      6             2
#> 9  2017-03-12 08:00:00    28800 17237    8      0             3
#> 10 2017-03-12 09:00:00    32400 17237    9      4             4
```

## Visualizing Marginal Temporal Trends

To begin getting a feel for this data, we’ll start by visualizing the marginal cyclical and longitudinal trends in eating patterns. If we wanted a more exacting visualization not only of the observed pattern but also of our confidence in estiamted temporal effects, we could use a linear modeling approach and visualize the marginal effects as in McVey et al (2020). For repeated measures on a large number of animals, such an approach is a more tratable means of generating summary information and identifying overall trends.

Here, with data from only a single animal and subsequently a more manageable number of observations, we are able to plot all of the availiable data to look for trends. We do this here using ggplot2 package to generate a scatter plot, and add a loess curve to help visualize the overall trend.

```
ggplot(cowdat, aes(x = HourCorrected, y = MinEat)) + 
  geom_point() + 
  geom_smooth(formula = y ~ x, method = 'loess') + 
  ggtitle('Cyclical Eating Pattern: Cow 47575') + 
  ylab('Minutes Spent Eating') + xlab('Hour')
```

```
ggplot(cowdat, aes(x = Date, y = MinEat)) + 
  geom_point() + 
  geom_smooth(formula = y ~ x, method = 'loess') + 
  ggtitle('Longitudinal Eating Pattern: Cow 47575') + 
  ylab('Minutes Spent Eating')
```

From these graphs several patterns emerge. As we might expect, there appears to be three peaks in eating time throughout the day, which correspond to the times that cows are expected to return to their home pen from their three daily trips to the milking parlor. This spike is perhaps the most pronounced after the evening milking, after which we expect cows to lay down and sleep, with more spikes in eating obsered following the morning and afternoon milkings.

The longitudinal trend is perhaps less defined, but there do appear to be longer eating bouts occuring in the later half of the observation window, which corresponds to the middle of the cows lactation when here appetite should be at its highest.

It is also readily apparent from these graphs, however, neither the cyclical or longitudinal axis are able to explain the bulk of the variability in eating time on their own. Daily flucutations and the enviornoment and management schedule will surely contribute to the noise of this system, but could there be other behavioral patterns present but unseen between these two temporal axes?

## Tubeplot Visualizations

To explore this question, we will generate a few tubeplots to visualize cyclical and longitudinal patterns conncurrently. While a single plot can be interactively manipulated so that the entire plot can be inspected, to more clearly highlight key patterns in this graphic, we will also demonstrate how the camera orientation option can be used to control vantage point of the figure that is initially shown, which is also handy when generating exporting still images to jpeg.

```
tubeplot(cowdat$HourCorrected, cowdat$Date, cowdat$MinEat, 
         plot_title = 'Eating Pattern of Cow 45757', 
         camera_orient = list(eye = list(x = 1.5, y = 0.5, z = 0))
         )
#> Warning: `arrange_()` is deprecated as of dplyr 0.7.0.
#> Please use `arrange()` instead.
#> See vignette('programming') for more help
#> This warning is displayed once every 8 hours.
#> Call `lifecycle::last_warnings()` to see where this warning was generated.
```

Here can see both a clear cyclical and longitudinal pattern. Looking around the radius of the tube plot, we can see cyclical patterns in eating time. We see that in the middle of the lounging periods, this cow is only occasionally inclided to spend time eating. Instead, most of this cow’s evening feed intake is occuring in the 2-3 hour windown after reutnring from the final milking of the day.

Longitudinal patterns can also be viewed by looking down the shaft of the tube. Here we see that, as the marginal means plots suggested, the times spent eating are growing in magnitude over the duration of the trial, but only during this 2-3 hour window of concentrated feed intake. For the overnight feeding period, there appears to be a relatively constant gradual increase time spent eating.

Finally, interaction effects can be visualized as shifts in the cyclical pattern along different segments of the tubes shaft. For this eating period, we see that in the first half of the trial eating times were low and always in the first hour or two following milking. After the half-way point of the trial, however, eating times not only become larger, but begin to extend to 3 and even four hours after returning from the parlor. Near the end of the trial, this pattern fades again, with feed intake again occuring directly after milking.

```
tubeplot(cowdat$HourCorrected, cowdat$Date, cowdat$MinEat, 
         plot_title = 'Eating Pattern of Cow 45757', 
         camera_orient = list(eye = list(x = -1.3, y = 1, z = 0))
         )
```

In the morning milking, we again see a clear cyclical pattern, with a narrow 3-4 hour window of concentrated feed intake. We also again see a clear longitudinal pattern were time spent eating appears to gradually increase over the course of the trial.

Here, however, we see an even clearer interaction effect between the cyclical and longitudinal axes. As eating time first begins to increase at around the middle of the shaft, the periods with highest minutes spend eating consistently occur between 8am and 9am. During the last half of the trial, however, these periods of elevated eating activity progressively shift towards 9am and then 10am, such that by the very end of the trial very little time is spent eating at 8am. This could indicate a progressive shift in the management schedule, such as delaying returning to the parlor, or perhaps the cow is prioritizing another behavior after returning to her home pen.

```
tubeplot(cowdat$HourCorrected, cowdat$Date, cowdat$MinEat, 
         plot_title = 'Eating Pattern of Cow 45757',     
         camera_orient = list(eye = list(x = -0.5, y = -1.8, z = 0))
         )
```

Looking at the afternoon milking, we see the same cyclical and lognitudinal patterns as in the previous graphs. Eating time is clearly concentrated to the hour or so following her return from the milking, and longer eating times are more prevalent during the later half of the trial.

In this case, however, we can also very clearly see that these pattern is far less defined than before. This cow tends to show spikes in eating time around 5pm, but we also see days where she appears to wait several hours after eating to visit the feed bunk. And while here eating times are extended in the later half of the trial, we don’t see a clear progression along the shaft of the tube as before, with several days of extended feeding time after milking often followed by several days of little or no feed intake.

**Summary:** Tube plots have reflect the cyclical and lognitudinal trends we anticipated from previous visualizations of the marginal effects. Tube plots also allow us to visualizae nonstationary cyclical trends along the longitudinal axis. They can also help visualize the relative strength of observed patterns.

## Exploring Plotting Options

Tubeplots in the LIT package are generated using plotly. In this section we’ll go over several commonly-used ploting options built into tubeplot() as an arguement. We’ll also show how users can pass additional visualization options supported by plotly.

First, in the previous plots, we used 0-23 to label the hour index. While this suffices to identify the timepoints for this data set, there may be other use scenarios the user may want to define different cyclic labels. For example, the variable used to define the cyclic indeces may be ordinal but only approximated by a numeric vector, in which case the category label may be more informative. In this data set, the sensors were temporally synced across cows, but were not aligned with the local time. Instead of transforming the variable Hour to the corect 0-23 hour index, we’ll show here how to set up a data frame to assign a text label with the corresponding time index.

```
myannotationkey <- data.frame(
  H = c(0, 1, 2, 3, 4, 5, 6, 7, 8, 9, 10, 11, 12, 13, 14, 15, 16, 17, 18, 19, 20, 21, 22, 23),
  text = c("7pm",  "8pm",  "9pm",  "10pm", "11pm", "12pm", "1am",  "2am",  "3am",  "4am",  "5am",  "6am",  "7am",  "8am",  "9am",  "10am", "11am", "12am", "1pm",  "2pm",  "3pm",  "4pm",  "5pm",  "6pm")
  )

tubeplot(cowdat$Hour, cowdat$Date, cowdat$MinEat, 
         plot_title = 'Eating Pattern of Cow 45757',
         annotationkey = myannotationkey,
         camera_orient = list(eye = list(x = 0.3, y = 2, z = 0))
         )
```

While the interactive plots are easy to manipulate in the RStudio environment, they may not always be convient. You may want to share these plots with non-R users (shun the nonbelievers). Or you may want to save them to inspect later without re-running your code. In either case, you can export interactive plotly graphics to an html, and tubplot() arguements will allow you to specify the directory and name of the file. In this case we’ll export a file names EatTube\_Cow45757.html to the subdirectory of our current working directory called Viz. *Note: you do not need to use the file extension* In some use cases, we may want to generate these plots in a large loop and save them directly to html files without bringing them up in the viewer - for example, if we wanted to generate tubeplots for each cow, and we have a herd of 200 cows. This can be done by simply using showplot = F

```
tubeplot(cowdat$Hour, cowdat$Date, cowdat$MinEat, 
         plot_title = 'Eating Pattern of Cow 45757',
         annotationkey = myannotationkey,
         camera_orient = list(eye = list(x = 0.3, y = 2, z = 0)),
         showplot = F,
         export_html = T,
         filename = '/Viz/EatTube_Cow45757'
         )
```

While the interactive features are good fun, your journal of choice may be a spoil sport and demand boring still images. In this case, we can use camera\_orient option to bring the most interesting part of the tube into focus, and export to jpeg using the orca options. *Note: to avoid compatibility issues when using this option, we suggest opening your RStudio through conda* To achieve the desired resolution, the orcascale option can be used to change the scale of the image (default value is 10).

```
tubeplot(cowdat$Hour, cowdat$Date, cowdat$MinEat, 
         plot_title = 'Eating Pattern of Cow 45757',
         annotationkey = myannotationkey,
         camera_orient = list(eye = list(x = 0.3, y = 2, z = 0)),
         showplot = F,
         export_html = T,
         filename = '/Viz/EatTube_Cow45757',
         orcascale = 5
         )
```

Finally, the plotly package has a seemingly endless array of ploting options to take advantage of, and users can use any of them to customize their tubplot output. We’ll show here how to tweak the marker size to generate a denser plot, and also how to change the color scheme

```
tubeplot(cowdat$Hour, cowdat$Date, cowdat$MinEat, 
         plot_title = 'Eating Pattern of Cow 45757',
         radius = 2,
         annotationkey = myannotationkey,
         camera_orient = list(eye = list(x = 0.3, y = 2, z = 0)),
         marker = list(size = 12),
         colors = 'YlOrRd'
         )
```

# Univariate Analysis of an Groups of Animals

We’ve shown how tubeplots can bring into greater detail complex temporal patterns in behaviors for a single animal. Most statistical analyses, however, center around comparisons between groups. For a more complete example of how tube plots can be integrated into a comprehensive analytical pipeline used to compare complex patterns both between groups of animals, see McVey et al (2020). Here we will focus on demonstrating their utility in exploratory data analyses.

In the Categorical Exploratory Data Analysis (CEDA) vignette, analysis of time budgets generated generated from ear tag accelerometer records revealed two small but distinct groups of cattle with respect to eating and rumination patterns. **Group 1** consists of 4 animals who spent a larger proportion of their time eating and relatively less time ruminating. **Group 2** consists of 8 cows domonstrating elevated rates of rumination but very little time eating.

Armed with such distinctive results for the overall time budget, we’ll now use tube plots to delve deeper into the temporal dynamics of this data to identify any distinctive patterns in eating and rumination patterns between the two groups.

First we’ll take a look at eating patterns. Because these groups are relatively small and seemingly quite homogenous, we’ll use mean to summarize observations across animals. For larger subsamples that may contain extreme values, we recomend using the median values.

```
groupdat_eat[1:10,]
#>             TimeStampF TimeDiff       Date Hour Cow645 Cow1135 Cow2219 Cow13482
#> 1  2017-03-12 00:00:00        0 2017-03-12    0     10       0       0        0
#> 2  2017-03-12 01:00:00     3600 2017-03-12    1     10       0       2        0
#> 3  2017-03-12 02:00:00     7200 2017-03-12    2      0      28       1        0
#> 4  2017-03-12 03:00:00    10800 2017-03-12    3      0      12       0        5
#> 5  2017-03-12 04:00:00    14400 2017-03-12    4      0      18       0       11
#> 6  2017-03-12 05:00:00    18000 2017-03-12    5      1       0       3        0
#> 7  2017-03-12 06:00:00    21600 2017-03-12    6      4      10       9        7
#> 8  2017-03-12 07:00:00    25200 2017-03-12    7      3       1       4        1
#> 9  2017-03-12 08:00:00    28800 2017-03-12    8      1      14       3        0
#> 10 2017-03-12 09:00:00    32400 2017-03-12    9      0       0       0        0
#>    Cow13956 Cow18649 Cow34360 Cow45757 Cow49670 Cow55482 Cow62002 Cow97907
#> 1         0       54       10        0        2        4       24        0
#> 2         6       26       24        3        1        1       13        6
#> 3        57        6       50        2        2        0       26        1
#> 4        18       30       39        0        7        0       21        5
#> 5         0       26       15        0        2        0       27        5
#> 6         3        8       30        6        3        6       31        6
#> 7        37       51       41       18        9        5       32        2
#> 8        30       27       21        6       14        1       29        0
#> 9        10        0       11        0        0        0       21        0
#> 10        0        1       19        4        0        0       19        3

eatout <- groupTubeplot(groupdat_eat$Hour, groupdat_eat$Date,
              groupdat_eat[,5:ncol(groupdat_eat),],
              c(2,2,2,2,1,1,1,2,2,2,1,2),
              func = 'mean',
              plot_title = 'Eating Pattern', 
              makediffplot = T,
              returnstats = T,
              annotationkey = myannotationkey,
              camera_orient = list(eye = list(x = 0.3, y = 2, z = 0))
              )

eatout$plots[[1]] # Group 1
```

```
eatout$plots[[3]] # Group 2
```

```
eatout$plots[[2]] # Diff Plot
```

```
eatout$summarydata[1:10,] # summary data for each group
#>    Cyclical_Index Longitudinal_Index Group_2 Group_1
#> 1               0                  1   50.25  50.250
#> 2               0                  1    0.00   0.000
#> 3               0                  1   22.00   2.000
#> 4               1                  1   17.25   2.875
#> 5               2                  1   34.75   4.250
#> 6               3                  1   27.00   3.625
#> 7               4                  1   17.00   4.500
#> 8               5                  1   18.00   3.125
#> 9               6                  1   40.25   8.000
#> 10              7                  1   26.75   3.750
```

The tube plots lend further weight to the strong distinctions between the two groups. The plot of the difference between groups shows that Group 2 cows consistently spent less time eating than Group 1 cows at virtually all timepoints. Group 2 cows spent some time eating upon their return from the milk parlor, but these spikes in eating activity were much lower in magnitude than the Group 1 cows. Additionally, eating activity amongst Group 2 animals is largely restricted to the first hour or two following their return from the milking parlor. Group 1 animals, on the other hand, logged some minutes spent eating throughout the morning and afternoon lounging periods, though this behavior was less persistent overnight.

Now we’ll take a look at rumination patterns

```
groupdat_rum[1:10,]
#>             TimeStampF TimeDiff       Date Hour Cow645 Cow1135 Cow2219 Cow13482
#> 1  2017-03-12 00:00:00        0 2017-03-12    0     14      22      36       16
#> 2  2017-03-12 01:00:00     3600 2017-03-12    1     39      30      11       39
#> 3  2017-03-12 02:00:00     7200 2017-03-12    2     43      25      16       17
#> 4  2017-03-12 03:00:00    10800 2017-03-12    3      0      19      31       43
#> 5  2017-03-12 04:00:00    14400 2017-03-12    4     32      24      33       32
#> 6  2017-03-12 05:00:00    18000 2017-03-12    5      9      21      21       11
#> 7  2017-03-12 06:00:00    21600 2017-03-12    6     32      16      15       20
#> 8  2017-03-12 07:00:00    25200 2017-03-12    7     46      45      40       39
#> 9  2017-03-12 08:00:00    28800 2017-03-12    8      5      23      29        6
#> 10 2017-03-12 09:00:00    32400 2017-03-12    9     24      30      22       24
#>    Cow13956 Cow18649 Cow34360 Cow45757 Cow49670 Cow55482 Cow62002 Cow97907
#> 1        24        0        9       38       33       11        0       26
#> 2        16       30       26       35       12       13        6       29
#> 3         2       16        3        9       39       27        4       44
#> 4        30       11       10       52       34       37        0       23
#> 5        22       30       12        4       32       35        2       15
#> 6        16        8        8        8       19        0        0       20
#> 7         8        5        4       28       34       34        3       34
#> 8        26       26        1       31       25       13        1       31
#> 9        27       13       21       45       56       42        1       23
#> 10       32       28       17       18       22       14        9       41

rumout <-groupTubeplot(groupdat_rum$Hour, groupdat_rum$Date,
              groupdat_rum[,5:ncol(groupdat_rum),],
              c(2,2,2,2,1,1,1,2,2,2,1,2),
              func = 'mean',
              plot_title = 'Rumination Pattern', 
              makediffplot = T,
              returnstats = T,
              annotationkey = myannotationkey,
              camera_orient = list(eye = list(x = 0.3, y = 2, z = 0))
              )

rumout$plots[[1]] # Group 1
```

```
rumout$plots[[3]] # Group 2
```

```
rumout$plots[[2]] # Diff Plot
```

```
rumout$summarydata[1:10,] # summary data for each group
#>    Cyclical_Index Longitudinal_Index Group_2 Group_1
#> 1               0                  1   43.75  43.750
#> 2               0                  1    0.25   0.250
#> 3               0                  1    8.25  24.500
#> 4               1                  1   19.50  26.000
#> 5               2                  1    6.25  27.500
#> 6               3                  1   12.75  29.875
#> 7               4                  1   16.50  25.875
#> 8               5                  1    8.00  13.625
#> 9               6                  1    5.00  26.625
#> 10              7                  1   13.50  33.750
```

The differences in rumination are equally distinct amongst groups. Group 2 cows seem to spend at least half their time ruminating throughout the day, with only slight dips seen in the hour where cows are being brought into the parlor to be milked. Group 1 cows, on the other hand, have a more distinct cyclical trend in their rumination patterns. They do not ruminate during their commute to the parlor, nor immediately upon their return. They show moderate levels of rumination throughout the morning and afternoon lounging periods, but rumination rates spike overnight.

While it is always possible that such stark contrasts between small groups of animals could be attributable to isolated issues with sensor placement or calibration, these visualizations do raise interesting questions about differences in mastications patterns adopted across the herd. As these animals were understocked with respect to feed bunk space, it seems unlikely that these divergent patterns reflect competition for a scarce resource. Possibly Group 1 cows are the picky eaters of the herd, and spend significant amounts of time at the feed face so that they can sorting out the more desirable bits of the TMR, while Group 2 cows simply hoover their food upon their return from the parlor before making a b-line for a nice freestall wherein to rest and ruminate. To be sure, there are complex temporal trends in such sensor data that can be easily visualizaed and explored using tube plot tools.

# References

MCVEY, C., HSIEH, F., MANRIQUEZ, D., PINEDO, P. and HORBACK, K. (2020). Mind the Queue: A Case Study in Visualizing Heterogeneous Behavioral Patterns in Livestock Sensor Data Using Unsupervised Machine Learning Techniques. Front. Vet. Sci. 7: 523. doi: 10.3389/fvets.2020.00523

MANRIQUEZ, D., CHEN, L., MELENDEZ, P. and PINEDO, P. (2019). The effect of an organic rumen-protected fat supplement on performance, metabolic status, and health of dairy cows. BMC Veterinary Research 15 450. https://doi.org/10.1186/s12917-019-2199-8
